# Supplementary material for: Harnessing the β-boron effect for regioselective Ru-catalyzed hydrosilylation of internal alkynes
Source: Nat Commun. 2025 May 14;16:4469. doi: 10.1038/s41467-025-59823-x (PMC12078650; doi:10.1038/s41467-025-59823-x)
Supplement: Supplementary file 1 — Supplementary Information [file 41467_2025_59823_MOESM1_ESM.pdf]

# Supplementary Information

## Harnessing the β-Boron Effect for Regioselective Ru-Catalyzed Hydrosilylation of Internal Alkynes

## Table of Contents

1. General information ........................................ 2
2. General procedure for preparation of the starting materials ........................................ 2
3. General procedure for the synthesis of product ........................................ 22
4. Synthetic Utilities ........................................ 60
5. Mechanism studies ........................................ 67
6. Computational Studies ........................................ 79
   6.1 Computational Details ........................................ 79
   6.2 Computational Discussions ........................................ 80
7. NMR spectra ........................................ 85
8. Reference ........................................ 206

## 1. General information

All the reactions were performed under nitrogen atmosphere in an oven-dried reaction tube. THF was dried over Na with benzophenone-ketyl intermediate as indicator. For chromatography, 200-300 mesh silica gel (Qingdao, China) was employed. All NMR–spectra were recorded on Bruker Avance III–400M or Ascend$^{TM}$ 500M in solvents as indicated under 25 °C. Chemical shifts are reported in ppm using tetramethylsilane as internal standard when using $CDCl_3$, $CD_3CN$, $(CD_3)_2CO$, $(CD_3)_2SO$ as the solvent, and coupling constants (*J*) were in Hertz (Hz). Multiplicities are reported using the following abbreviations: s = singlet, d = doublet, t = triplet, q = quartet, m = multiplet. The carbon directly attached to the boron atom was not detectable by $^{13}$C NMR, likely due to quadropolar relaxation. High–resolution mass spectra (HRMS) were recorded on a Bruker VPEXII spectrometer with ESI mode unless otherwise stated. Unless otherwise noted, all commercially available materials were used without further purification. PE: petroleum ether; EA: ethyl acetate; DCM, dichloromethane; DCE: 1,2-dichloroethane; DMF: *N, N*-dimethylformamide; THF: tetrahydrofuran.

## 2. General procedure for preparation of the starting materials

Silanes and ruthenium catalysts were purchased from commercial sources and used without further purification. The propargylic MIDA boronates were prepared according to the following procedure and spectroscopic data of known compounds were consistent with the reported values. **S-1**, **S-2**, **S-4**, **S-5**, **S-7**, **S-8**, **S-10**, **S-11** **S-13**, **S-14**, **S-16**, **S-17**, **S-18**, **S-19**, **S-20**, **S-21**, **S-25**, **S-27**, **S-28**, **S-36**, **S-38**, **S-39**, **S-40**, **S-43** are known compounds[1-3]. Other compounds were prepared according to the general procedure shown in Supplementary Figure 1.

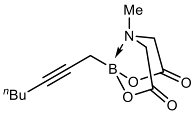

**S-1**

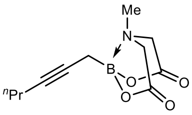

**S-2**

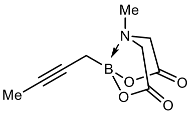

**S-3**

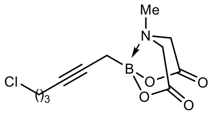

**S-4**

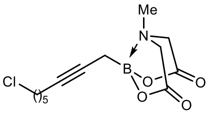

**S-5**

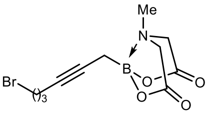

**S-6**

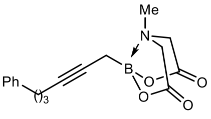

**S-7**

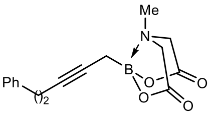

**S-8**

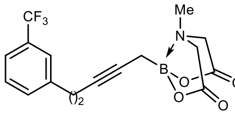

**S-9**

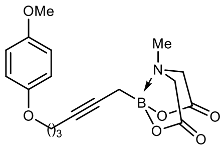

**S-10**

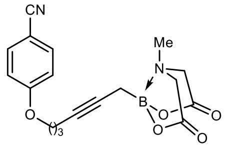

**S-11**

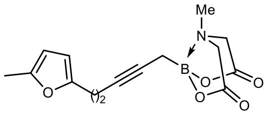

**S-12**

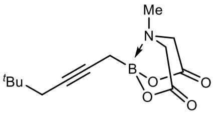

**S-13**

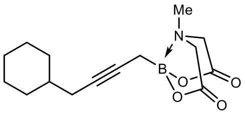

**S-14**

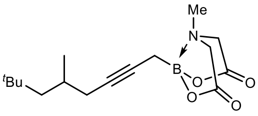

**S-15**

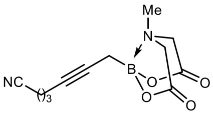

**S-16**

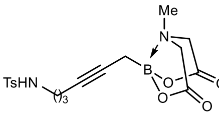

**S-17**

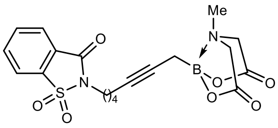

**S-18**

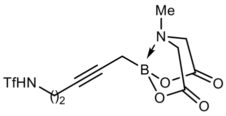

**S-19**

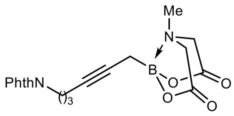

**S-20**

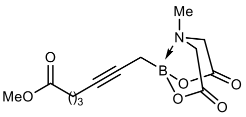

**S-21**

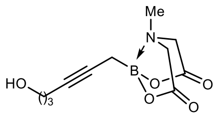

**S-22**

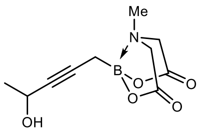

**S-23**

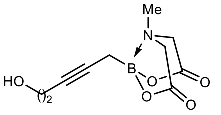

**S-24**

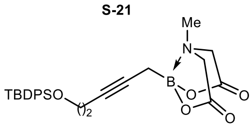

**S-25**

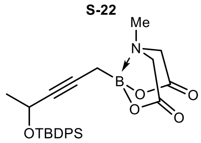

**S-26**

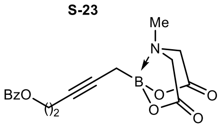

**S-27**

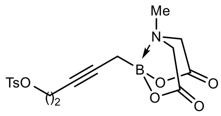

**S-28**

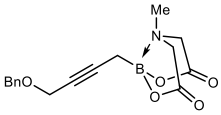

**S-29**

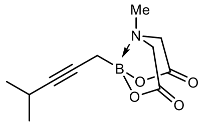

**S-30**

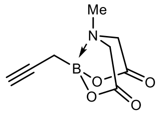

**S-31**

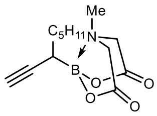

**S-32**

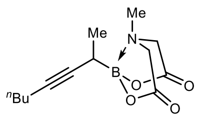

**S-33**

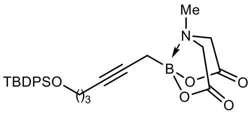

**S-34**

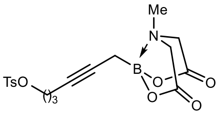

**S-35**

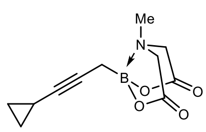

**S-36**

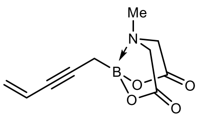

**S-37**

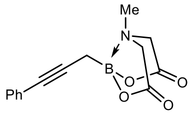

**S-38**

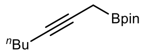

**S-39**

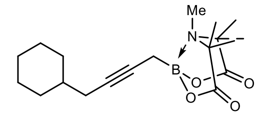

**S-40**

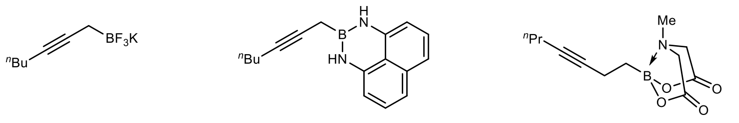

S-41 S-42 S-43

**Supplementary Figure 1: starting materials.**

## General procedure A

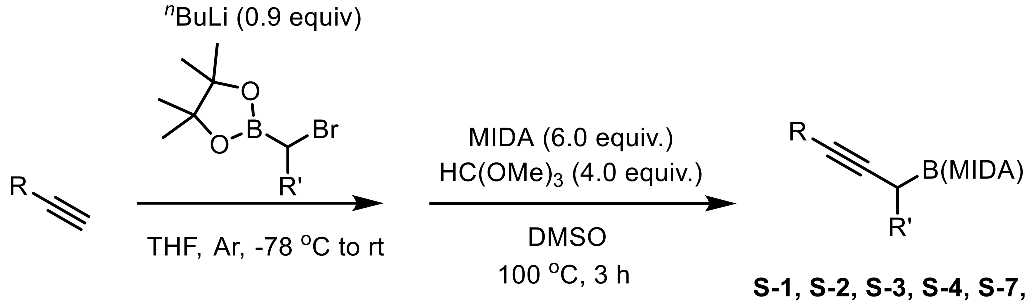

**S-1, S-2, S-3, S-4, S-7, S-8, S-10, S-11, S-13, S-14, S-16, S-21, S-25, S-26, S-27, S-29, S-30, S-33, S-34, S-36, S-38, S-39, S-40**

Step 1: To a stirred solution of THF (10 mL) containing terminal alkyne (5 mmol), ${}^{n}$butyl lithium in hexane (2.5 M, 1.8 mL, 4.5 mmol) was added dropwise at -78 °C. The resulting solution was stirred at the same temperature for 1 h, and then α-bromo-boronate (commercially available or prepared by known literature[4]) was added dropwise and the mixture was allowed to stir at room temperature for 2 h. After that, saturated $NH_4Cl$ (aq.) was added to the reaction mixture at 0 °C and the solution was stirred for 20 min. The reaction mixture was mixed with water and the product was extracted with dichloromethane (DCM). The organic phase was separated, and the aqueous layer was extracted with DCM (30 mL) for three times. The combined organic layer was dried over anhydrous $Na_2SO_4$ and concentrated under reduced pressure.

Step 2: Anhydrous DMSO (5 mL) was added to dissolve the solid (or viscous oil). Then *N*-methyliminodiacetic acid (MIDA, 6.0 equiv.) and $CH(OMe)_3$ (4.0 equiv.) was added. The resulting mixture was stirred at 100 °C until the propargyl boronic esters was used up as monitored by GC-MS. After cooling to room temperature, the reaction mixture was diluted with ethyl acetate (10 mL) and water (10 mL). The organic phase was separated, and the aqueous layer was extracted with ethyl acetate (20 mL) for three

times. The combined organic layer was dried over anhydrous $Na_2SO_4$ and concentrated under reduced pressure. The resulting crude product was purified by flash chromatography on silica gel with an appropriate solvent as eluent to afford the pure product.

**General procedure B**

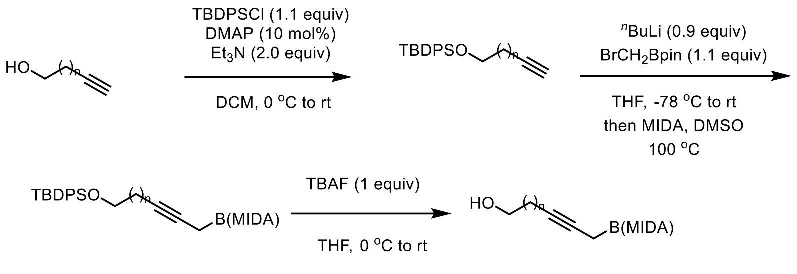


**S-25**, **S-26**, **S-35** **S-22**, **S-23**, **S-24**

Step 1: To a solution of alcohol (1.5 mL, 20 mmol, 1.0 equiv) and imidazole (3.4 g, 50 mmol, 2.5 equiv) in THF (40 mL), was added TBDPSCl (6.2 mL, 24 mmol, 1.2 equiv) dropwise at room temperature. The reaction mixture was stirred at for 4 h. After that, the reaction mixture was quenched with saturated $NH_4Cl$ solution and was extracted with $Et_2O$. The combined organic layer was dried over anhydrous $Na_2SO_4$ and concentrated under reduced pressure. The resulting crude product was purified by flash chromatography on silica gel to afford the pure product.

Step 2: To a stirred solution of THF (17 mL) containing terminal alkynes (17 mmol), $^n$butyl lithium in hexane (2.5 M, 6.1 mL, 15.3 mmol) was added dropwise at -78 °C. The resulting solution was stirred at the same temperature for 1 h, and then (bromomethyl)pinacolborane ($BrCH_2Bpin$, 3.76 g, 17 mmol) was added dropwise and the mixture was allowed to stir at room temperature for 2 h. After that, saturated $NH_4Cl$ (aq.) was added to the reaction mixture at 0 °C and the solution was stirred for 20 min. The reaction mixture was mixed with water and the product was extracted with dichloromethane (DCM). The organic phase was separated and the aqueous layer was extracted with DCM (30 mL) for three times. The combined organic layer was dried over anhydrous $Na_2SO_4$ and concentrated under reduced pressure.

Step 3: Anhydrous DMSO (40 mL) was added to dissolve the solid (or viscous oil). Then *N*-methyliminodiacetic acid (MIDA, 6.0 equiv.) and $CH(OMe)_3$ (4.0 equiv.) was added. The resulting mixture was stirred at 100 °C for 3 h. After cooling to room temperature, the reaction mixture was diluted with ethyl acetate (50 mL) and water (50 mL). The organic phase was separated and the aqueous layer was extracted with ethyl acetate (50 mL) for three times. The combined organic layer was dried over anhydrous $Na_2SO_4$ and concentrated under reduced pressure. The resulting crude product was purified by flash chromatography on silica gel with an appropriate solvent as eluent to afford the pure product.

Step 4: To a stirred solution of THF (7.5 ml) containing the product (2.0 mmol) of previous step, TBAF (1.0 M in THF, 2 mL) was added at 0 °C. The reaction mixture was stirred at 0 °C for 30 min. Then the reaction mixture was allowed to warm to room temperature and stirred at that temperature for 4 h. The reaction mixture was directly purified by flash chromatography on silica gel to afford the pure product (eluent = ethyl acetate/acetonitrile v/v 5:1).

**Synthesis of S-19**

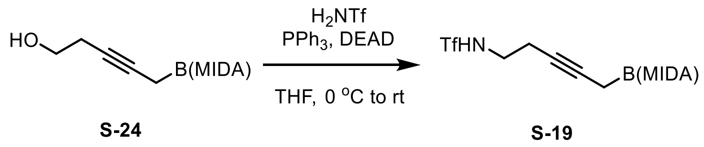


Under argon atmosphere, a Schlenk tube equipped with a magnetic stir bar was charged with **S-24** (47.8 mg, 0.2 mmol), trifluoromethane sulfonamide (35.8 mg, 1.2 equiv.) and $PPh_3$ (54.2 mg, 2.0 equiv.) in anhydrous THF (1 mL). The reaction mixture was cooled to 0 °C. DEAD (34.8 mg, 2.0 equiv.) was added slowly to the reaction mixture. The reaction mixture was stirred at room temperature for 1 h. The reaction mixture was directly purified by flash chromatography on silica gel to afford the pure product **S-19** (eluent = ethyl acetate/petroleum ether v/v 1:1 to 4:1).

**Synthesis of S-6**

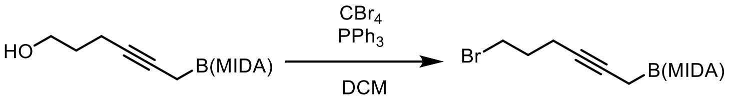

S-22 S-6

Under argon atmosphere, a Schlenk tube equipped with a magnetic stir bar was charged with **S-22** (50.6 mg, 0.2 mmol), and $CBr_4$ (79.5 mg, 1.2 equiv.) in 1 mL anhydrous DCM. The reaction mixture was cooled to 0 °C. $PPh_3$ (62.9 mg, 1.2 equiv.) in 0.5 mL anhydrous DCM was added slowly to the reaction mixture. The reaction mixture was stirred at room temperature for 1 h. The reaction mixture was directly purified by flash chromatography on silica gel to afford the pure product **S-6**.

**Synthesis of S-28, S-35**

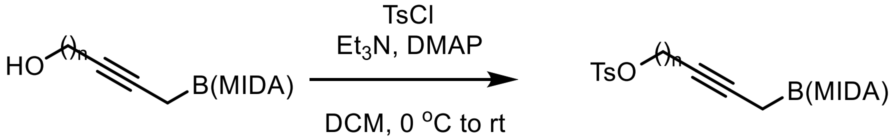

(n = 2 or 3) **S-28, S-35**

Under an argon atmosphere, a Schlenk tube equipped with a magnetic stir bar was charged with **S-22** or **S-24** (0.2 mmol, 1.0 equiv.) and DCM (2 mL, 0.1 M). The reaction mixture was cooled to 0 °C. $Et_3N$ (40.4 mg, 0.4 mmol, 2.0 equiv.) was added to the reaction mixture. The resulting mixture was stirred at 0 °C for 30 min. TsCl (45.8 mg, 0.24 mmol, 1.2 equiv.), DMAP (2.2 mg, 0.02 mmol, 10 mol%) and DCM were added to the reaction mixture. Then the reaction mixture was allowed to warm to room temperature and stirred overnight. Until the complete consumption of **S-22** as monitored by TLC analysis. The reaction mixture was diluted with ethyl acetate and water. The organic phase was separated and the aqueous layer was extracted with ethyl acetate and acetone for three times. The combined organic layer was dried over anhydrous $Na_2SO_4$ and concentrated under reduced pressure. The resulting crude product was purified by flash chromatography on silica gel to afford the pure product.

**General procedure C**

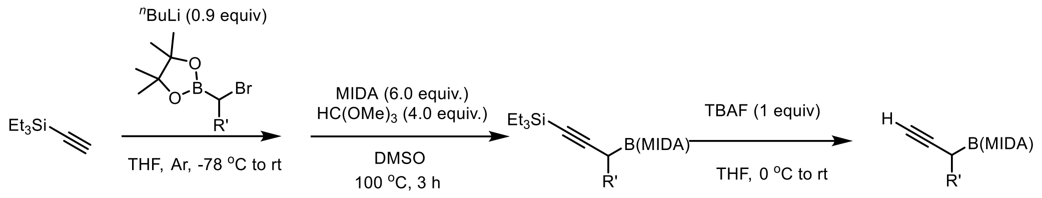


**S-31**, **S-32**

Step 1: To a stirred solution of THF (10 mL) containing triethyl(ethynyl)silane (5 mmol), $^n$butyl lithium in hexane (2.5 M, 1.8 mL, 4.5 mmol) was added dropwise at -78 °C. The resulting solution was stirred at the same temperature for 1 h, and then α-bromo-boronates was added dropwise and the mixture was stirred at room temperature overnight. After the reaction, saturated $NH_4Cl$ (aq.) was added to the reaction mixture at 0 °C and the solution was stirred for 20 min. The reaction mixture was mixed with water and the product was extracted repeatedly with dichloromethane (DCM). The organic phase was separated, and the aqueous layer was extracted with DCM (30 mL) for three times. The combined organic layer was dried over anhydrous $Na_2SO_4$ and concentrated under reduced pressure.

Step 2: Anhydrous DMSO (5 mL) was added to dissolve the solid (or viscous oil) which was then added via syringe to a suspension of *N*-methyliminodiacetic acid (MIDA, 6.0 equiv.) and $CH(OMe)_3$ (4.0 equiv.) in DMSO (10 mL). The resulting mixture was stirred at 100 °C for 3 h. After cooling to room temperature, the reaction mixture was diluted with ethyl acetate (10 mL) and water (10 mL). The organic phase was separated, and the aqueous layer was extracted with ethyl acetate (20 mL) for three times. The combined organic layer was dried over anhydrous $Na_2SO_4$ and concentrated under reduced pressure. The resulting crude product was purified by flash chromatography on silica gel with an appropriate solvent as eluent to afford the pure product.

Step 3: To a stirred solution of THF (7.5 ml) containing the product (2.0 mmol) of previous step, TBAF (1.0 M in THF, 2 mL) was added at 0 °C. The reaction mixture was stirred at 0 °C for 30 min. Then the reaction mixture was allowed to warm to room temperature and stirred at that temperature for 2 h. The reaction mixture was directly purified by flash chromatography on silica gel with an appropriate solvent as eluent to afford the pure product.

**General procedure D**

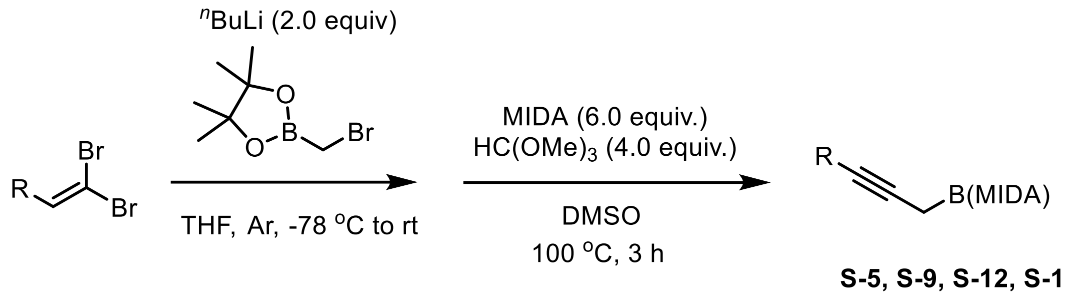


**S-5, S-9, S-12, S-15**

To a stirred solution of THF (10 mL) containing dibromoolefin (5 mmol, 1.0 equiv.), *n*-butyl lithium in hexane (2.5 M, 4.0 mL, 10 mmol) was added dropwise at -78 °C. The resulting solution was stirred at the same temperature for 1 h, and then $\alpha$-bromo boronic esters (5 mmol, 1.0 eq) was added dropwise and the mixture was stirred at room temperature for 2 h. After the reaction, Saturated $NH_4Cl$ (aq.) was added to the reaction mixture at 0 °C and the solution was stirred for 20 min. The reaction mixture was mixed with water and the product was extracted repeatedly with DCM. The organic phase was separated and the aqueous layer was extracted with DCM (30 mL) for three times. The combined organic layer was dried over anhydrous $Na_2SO_4$ and concentrated under reduced pressure. Anhydrous DMSO (5 mL) was added to dissolve the solid (or viscous oil) which was then added via syringe to a suspension of *N*-methyliminodiacetic acid (MIDA, 6.2 equiv.) and $CH(OMe)_3$ (4.0 equiv) in DMSO. The resulting mixture was stirred at 100 °C in an oil bath until the propargyl boronic esters was used up by GCMS monitoring. After cooling to room temperature, the reaction mixture was diluted with ethyl acetate (10 mL) and water (10 mL). The organic phase was separated and the aqueous layer was extracted with ethyl acetate (10 mL) for three times. The combined organic layer was dried over anhydrous $Na_2SO_4$ and concentrated under reduced pressure. The resulting crude product was purified by flash chromatography on silica gel with an appropriate solvent as eluent to afford the pure product.

**Synthesis of S-17, S-18, S-20**

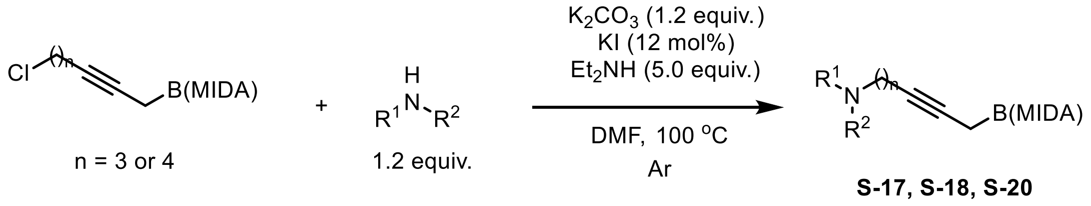


In a Schlenk tube charged with a magnetic stir bar, the alkyl chloride substitute compound (2.0 mmol, 1.0 equiv.) was dissolved in DMF (4 mL). Subsequently, amide (1.2 equiv.) was added in one portion followed by of $K_2CO_3$ (330 mg, 2.4 mmol), and potassium iodide (43 mg, 0.24 mmol). The yellowish suspension was heated at 100 °C for 24 hours. The reaction was then cooled to room temperature. $H_2O$ (10 mL) was introduced to the reaction and extracted with DCM (3 x 15 mL). The combined organics were then washed with brine (3 x 10 mL) to remove the DMF. Combined organics were dried over anhydrous $Mg_2SO_4$, filtered and then concentrated to afford crude followed by flash chromatography on silica gel with an appropriate solvent as eluent to afford the pure product. Desired product was afforded as a white solid.

**Synthesis of S-37**

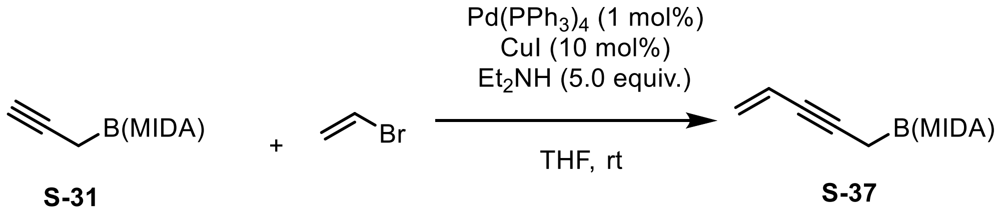


In an oven-dried Schlenk tube equipped with a stirring bar, $Pd(PPh_3)_4$ (1 mol%) and CuI (10 mol%) and **S-31** (1 mmol, 195 mg, 1 equiv,) were charged under argon, then dry THF (2 mL, 0.5 M) was added. The suspension was cooled to 0 °C, then $Et_2NH$ (365 mg, 5 mmol, 5 equiv) was added, followed by vinyl bromide (1.5 mL, 1 M in THF solution, 1.5 equiv). The reaction was warmed at room temperature and stirred for 1 h, then the reaction was filtered through a short pad of celite, rinsing thoroughly with ethyl acetate. The filtrate was dried *in vacuo*, then the crude mixture were purified by column chromatography to afford the pure **S-37**.

**Synthesis of S-41**

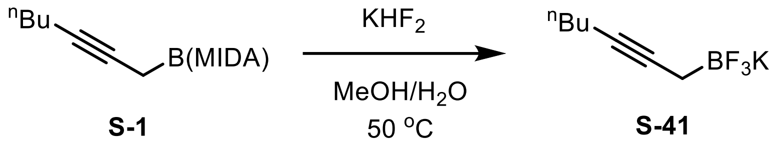


To a stirred solution of **S-1** (50.2 mg, 0.2 mmol) in MeOH (2.0 mL) was added saturated $KHF_2/H_2O$ (1.0 mL). The reaction was allowed to stirred at 60 °C overnight. After the reaction was completed, solvent was removed under reduced pressure. The crude mixture was resolved in acetone and filtered. Solution was collected and removed solvent under reduced pressure. Product was recrystallization in acetone/$Et_2O$ to provide pure **S-41**.

**Synthesis of S-42**

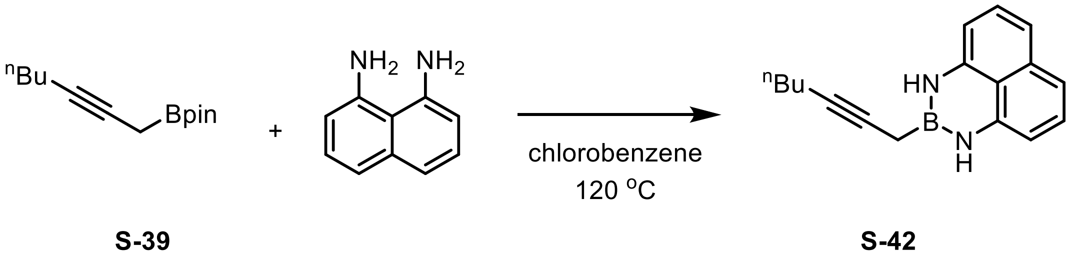


To a stirred solution of **S-39** (1.3 g, 6 mmol) in chlorobenzene (6.0 mL) was added 1,8-diaminonaphthalene (1.9 g, 12 mmol, 2.0 equiv.). The reaction was allowed to stirred at 120 °C for 3 h. The solvent was removed under reduced pressure and purified by flash chromatography on silica gel to afford the pure **S-42**.

**Synthesis of S-43**

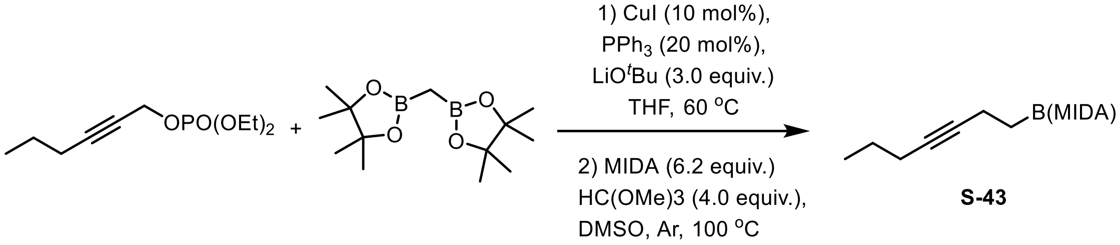


In air, CuI (100mg, 10 mol%), $^t$BuOLi (1.27 g, 3 equiv.), $PPh_3$ (289 mg, 20 mol%), were added to a Schlenk flask equipped with a stir bar. The vessel was evacuated and

filled with argon (three cycles). THF (25 mL) was added in turn by syringe under argon atmosphere at room temperature, and then stirred at 60 oC for 10 min. Then diborylmethane (2.84 g) was added in turn under an argon atmosphere and stirred at 60 oC for 10 min. And propargyl phosphates (1.23 g, 5.3 mmol) were added in turn under an argon atmosphere. The reaction mixture was stirred at 60 $^{o}$C for 24 h. The reaction mixture was then diluted with EtOAc, filtered through silica gel with copious washings (EtOAc), concentrated, and afforded a colorless oil. Anhydrous DMSO (5 mL) was added to dissolve the oil which was then added via syringe to a suspension of *N*methyliminodiacetic acid (MIDA, 6.2 equiv) and $CH(OMe)_3$ (4.0 equiv) in DMSO. The resulting mixture was stirred at 100 $^{o}$C until the boronic esters was used up by GC-MS monitoring. After cooling to room temperature, the reaction mixture was diluted with ethyl acetate (10 mL) and water (10 mL). The organic phase was separated and the aqueous layer was extracted with ethyl acetate (10 mL) for three times. The combined organic layer was dried over anhydrous $Na_2SO_4$ and concentrated under reduced pressure. The resulting crude product was purified by flash chromatography on silica gel with an eluent (petroleum ether/ EtOAc = 1:2 to 1:5, v/v) afford the desired product as white solid.

**2-(but-2-yn-1-yl)-6-methyl-1,3,6,2-dioxazaborocane-4,8-dione (S-3)**

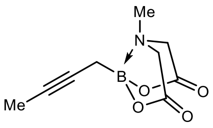


**S-3**

Following the **General procedure A**, the product **S-3** was obtained in 44% yield (with prop-1-yne in THF (1.0 M), 2 steps) as a white solid after column chromatography (eluent = ethyl acetate/petroleum ether v/v 4:1).

**$^{1}$H NMR (400 MHz, Acetone-*d*$_6$)** δ 4.24 (d, *J* = 16.8 Hz, 2H), 4.06 (d, *J* = 16.8 Hz, 2H), 3.25 (s, 3H), 1.69 (t, *J* = 2.7 Hz, 3H), 1.57 (s, 2H).

**$^{13}$C NMR (101 MHz, Acetone-*d*$_6$)** δ 168.5, 77.9, 75.5, 63.3, 46.2, 3.3.

**ESI-MS**: calculated for $C_9H_{12}BNNaO_4^+$ $[M+Na]^+$, 232.0572; Found, 232.0576.

**2-(6-bromohex-2-yn-1-yl)-6-methyl-1,3,6,2-dioxazaborocane-4,8-dione (S-6)**

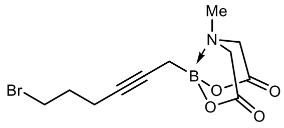

**S-6**

The product **S-6** was obtained in 65% yield (from **S-22**) as a white solid after column chromatography (eluent = ethyl acetate/petroleum ether v/v 1:1 to 2:1).

**$^{1}$H NMR (400 MHz, DMSO-*d*$_6$)** δ 4.28 (d, *J* = 17.0 Hz, 2H), 3.96 (d, *J* = 17.1 Hz, 2H), 3.55 (t, *J* = 6.5 Hz, 2H), 2.99 (s, 3H), 2.24 (tt, *J* = 6.9, 2.6 Hz, 2H), 1.89 (tt, *J* = 6.7, 6.8 Hz, 2H), 1.55 (t, *J* = 2.6 Hz, 2H).

**$^{13}$C NMR (101 MHz, DMSO-*d*$_6$)** δ 169.0, 80.0, 77.8, 62.7, 46.3, 34.4, 32.0, 17.5.

**ESI-MS**: calculated for $C_{11}H_{15}BBrNNaO_4^+$ $[M+Na]^+$, 338.0170; Found, 338.0176.

**6-methyl-2-(5-(3-(trifluoromethyl)phenyl)pent-2-yn-1-yl)-1,3,6,2-dioxazaborocane-4,8-dione (S-9)**

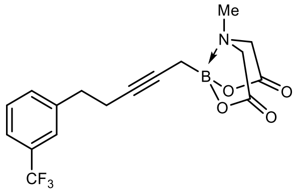

**S-9**

Following the **General procedure D**, the product **S-9** was obtained in 40% yield (from 1-(4,4-Dibromo-3-buten-1-yl)-3-(trifluoromethyl)benzene, 2 steps) as a white solid after column chromatography (eluent = ethyl acetate/petroleum ether v/v 2:1).

**$^{1}$H NMR (400 MHz, DMSO-*d*$_6$)** δ 7.61 (s, 1H), 7.58 – 7.52 (m, 3H), 4.22 (d, *J* = 17.1 Hz, 2H), 3.83 (d, *J* = 17.0 Hz, 2H), 2.87 (s, 3H), 2.79 (t, *J* = 7.3 Hz, 2H), 2.44 (tt, *J* = 7.4, 2.5 Hz, 2H), 1.49 (t, *J* = 2.5 Hz, 2H).

**$^{13}$C NMR (101 MHz, DMSO-*d*$_6$)** δ 168.6, 142.2, 132.7, 129.2, 129.0 (q, *J* = 31.0 Hz), 127.3 (q, *J* = 272.2 Hz), 125.0 (q, *J* = 3.9 Hz), 122.9 (d, *J* = 4.0 Hz), 79.4, 78.3, 62.1, 45.6, 34.1, 19.8.

**$^{19}$F NMR (376 MHz, DMSO-*d*$_6$)** δ -60.92.

**ESI-MS**: calculated for $C_{17}H_{17}BF_3NNaO_4^+$ $[M+Na]^+$, 390.1095; Found, 390.1087.

**6-methyl-2-(5-(5-methylfuran-2-yl)pent-2-yn-1-yl)-1,3,6,2-dioxazaborocane-4,8-dione (S-12)**

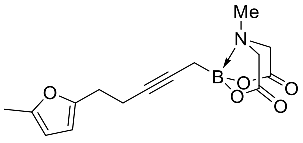


**S-12**

Following the **General procedure D**, the product **S-12** was obtained in 43% yield (from 2-(4,4-dibromobut-3-en-1-yl)-5-methylfuran, 2 steps) as a white solid after column chromatography (eluent = ethyl acetate/petroleum ether v/v 2:1).

**$^{1}$H NMR (400 MHz, Acetone-$d_6$)** δ 5.98 (d, $J$ = 2.9 Hz, 1H), 5.89 (d, $J$ = 2.9 Hz, 1H), 4.20 (d, $J$ = 16.8 Hz, 2H), 3.93 (d, $J$ = 16.8 Hz, 2H), 3.18 (s, 3H), 2.70 (t, $J$ = 7.2 Hz, 2H), 2.42 (tt, $J$ = 7.4, 2.6 Hz, 2H), 2.21 (s, 3H), 1.58 (s, 2H).

**$^{13}$C NMR (101 MHz, Acetone-$d_6$)** δ 168.5, 153.7, 151.2, 107.2, 107.0, 79.7, 79.3, 63.3, 46.2, 28.5, 18.4, 13.5.

**ESI-MS**: calculated for $C_{15}H_{18}BNNaO_5^+$ [M+Na]$^+$, 326.1170; Found, 326.1171.

**6-methyl-2-(5,7,7-trimethyloct-2-yn-1-yl)-1,3,6,2-dioxazaborocane-4,8-dione (S-15)**

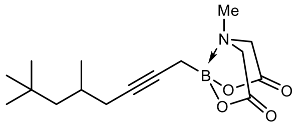


**S-15**

Following the **General procedure D**, the product **S-15** was obtained in 39% yield (1,1-dibromo-4,6,6-trimethylhept-1-ene, 2 steps) as a white solid after column chromatography (eluent = ethyl acetate/petroleum ether v/v 2:1).

**$^{1}$H NMR (400 MHz, DMSO-$d_6$)** δ 4.28 (dd, $J$ = 17.0, 1.3 Hz, 2H), 3.93 (d, $J$ = 17.0 Hz, 2H), 3.00 (s, 3H), 2.09 – 1.92 (m, 2H), 1.64 – 1.56 (m, 1H), 1.54 (t, $J$ = 2.6 Hz, 2H), 1.28 (dd, $J$ = 13.9, 4.1 Hz, 1H), 1.01 (dd, $J$ = 13.9, 6.3 Hz, 1H), 0.93 (d, $J$ = 6.7 Hz, 3H), 0.87 (s, 9H).

**$^{13}$C NMR (101 MHz, DMSO-$d_6$)** δ 169.0, 79.6, 78.4, 62.6, 50.0, 46.2, 31.1, 30.3, 29.4, 28.5, 22.7.

**ESI-MS**: calculated for $C_{16}H_{26}BNNaO_4^+$ [M+Na]$^+$, 330.1847; Found, 330.1849.

**1,1,1-trifluoro-*N*-(5-(6-methyl-4,8-dioxo-1,3,6,2-dioxazaborocan-2-yl)pent-3-yn-1-yl)methanesulfonamide (S-19)**

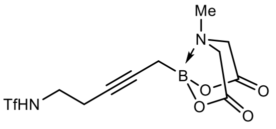


**S-19**

The product **S-19** was obtained in 64% yield (from **S-24**) as a white solid after column chromatography (eluent = ethyl acetate/petroleum ether v/v 1:1 to 2:1).

**$^1$H NMR (400 MHz, Acetone-*d*$_6$)** δ 4.26 (d, *J* = 17.0 Hz, 2H), 4.08 (d, *J* = 16.9 Hz, 2H), 3.39 (t, *J* = 7.0 Hz, 2H), 3.26 (s, 3H), 2.47 (tt, *J* = 7.0, 2.6 Hz, 2H), 1.64 (t, *J* = 2.6 Hz, 2H).

**$^{13}$C NMR (101 MHz, Acetone-*d*$_6$)** δ 168.7, 120.83 (q, *J* = 320.9 Hz), 81.4, 76.1, 63.3, 46.4, 44.2, 21.4.

**$^{19}$F NMR (376 MHz, Acetone-*d*$_6$)** δ -78.75.

**ESI-MS**: calculated for $C_{11}H_{14}BF_3N_2NaO_6S^+$ [M+Na]$^+$, 393.0510; Found, 393.0507.

**2-(6-hydroxyhex-2-yn-1-yl)-6-methyl-1,3,6,2-dioxazaborocane-4,8-dione** (**S-22**)

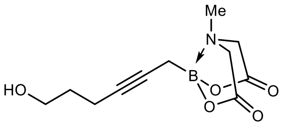


**S-22**

Following the **General procedure B**, the product **S-22** was obtained in 52% yield (from **S-34**) as a white solid after column chromatography (eluent = ethyl acetate/acetonitrile v/v 10:1 to 5:1).

**$^1$H NMR (400 MHz, $CD_3CN$)** δ 4.00 (d, *J* = 16.9 Hz, 2H), 3.88 (d, *J* = 16.9 Hz, 2H), 3.51 (t, *J* = 6.2 Hz, 2H), 2.54 (t, *J* = 5.3 Hz, 1H), 2.21 – 2.11 (m, 2H), 1.64 – 1.53 (m, 4H).

**$^{13}$C NMR (101 MHz, $CD_3CN$)** δ 168.9, 80.4, 78.9, 63.4, 61.4, 46.6, 32.8, 15.8.

**ESI-MS**: calculated for $C_{11}H_{16}BNNaO_5^+$ [M+Na]$^+$, 276.1014; Found, 276.1015.

**2-(4-hydroxypent-2-yn-1-yl)-6-methyl-1,3,6,2-dioxazaborocane-4,8-dione (S-23)**

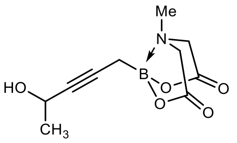

**S-23**

Following the **General procedure B**, the product **S-23** was obtained in 64% yield (from **S-26**) as a white solid after column chromatography (eluent = ethyl acetate/acetonitrile v/v 10:1 to 5:1).

**$^{1}$H NMR (500 MHz, $CD_3CN$)** δ 4.41 – 4.32 (m, 1H), 4.01 (d, *J* = 16.9 Hz, 2H), 3.90 (dd, *J* = 17.0, 3.1 Hz, 2H), 3.11 (d, *J* = 5.3 Hz, 1H), 3.05 (s, 3H), 1.65 (s, 2H), 1.27 (d, *J* = 6.5 Hz, 3H).

**$^{13}$C NMR (126 MHz, $CD_3CN$)** δ 168.9, 83.9, 82.3, 63.5, 63.5, 58.4, 46.7, 25.1.

**ESI-MS**: calculated for $C_{10}H_{14}BNNaO_5^+$ $[M+Na]^+$, 262.0857; Found, 262.0862.

**2-(5-hydroxypent-2-yn-1-yl)-6-methyl-1,3,6,2-dioxazaborocane-4,8-dione (S-24)**

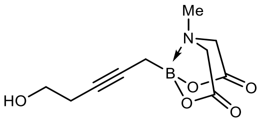

**S-24**

Following the **General procedure B**, the product **S-24** was obtained in 58% yield (from **S-26**) as a white solid after column chromatography (eluent = ethyl acetate/acetonitrile v/v 10:1 to 5:1).

**$^{1}$H NMR (400 MHz, Acetone-*d*$_6$)** δ 4.23 (d, *J* = 16.8 Hz, 2H), 4.17 (d, *J* = 16.9 Hz, 2H), 3.81 (s, 1H), 3.58 (t, *J* = 6.6 Hz, 2H), 3.28 (s, 3H), 2.28 (tt, *J* = 6.6, 2.6 Hz, 2H), 1.59 (t, *J* = 2.6 Hz, 2H).

**$^{13}$C NMR (101 MHz, Acetone-*d*$_6$)** δ 168.7, 80.0, 78.2, 63.4, 61.8, 46.1, 23.7.

**ESI-MS**: calculated for $C_{10}H_{14}BNNaO_5^+$ $[M+Na]^+$, 262.0857; Found, 262.0859.

**2-(4-((tert-butyldiphenylsilyl)oxy)pent-2-yn-1-yl)-6-methyl-1,3,6,2-dioxazaborocane-4,8-dione (S-26)**

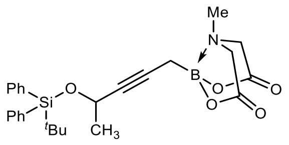

**S-26**

Following the **General procedure A**, the product **S-26** was obtained in 39% yield (from 3-butyn-2-ol, 3 steps) as a white solid after column chromatography (eluent = ethyl acetate/petroleum ether v/v 2:1).

**$^{1}$H NMR (500 MHz, DMSO-*d*$_6$)** δ 7.70 – 7.58 (m, 4H), 7.51 – 7.36 (m, 6H), 4.44 (tq, *J* = 6.5, 3.9 Hz, 1H), 4.26 (dd, *J* = 17.0, 12.8 Hz, 2H), 3.89 (dd, *J* = 27.9, 17.0 Hz, 2H), 2.89 (s, 3H), 1.55 (s, 2H), 1.26 (d, *J* = 6.3 Hz, 3H), 1.00 (s, 9H).

**$^{13}$C NMR (126 MHz, DMSO-*d*$_6$)** δ 168.5, 168.5, 135.3, 135.2, 129.9, 129.8, 127.8, 127.8, 83.0, 81.4, 62.1, 62.1, 59.9, 45.6, 26.7, 25.5, 18.8.

**ESI-MS**: calculated for $C_{26}H_{32}BNNaO_5Si^+$ $[M+Na]^+$, 500.2035; Found, 500.2042.

**2-(4-(benzyloxy)but-2-yn-1-yl)-6-methyl-1,3,6,2-dioxazaborocane-4,8-dione (S-29)**

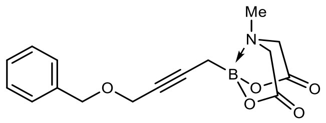

**S-29**

Following the **General procedure A**, the product **S-29** was obtained in 32% yield (from ((prop-2-yn-1-yloxy)methyl)benzene, 2 steps) as a white solid after column chromatography (eluent = ethyl acetate/petroleum ether v/v 3:1).

**$^{1}$H NMR (400 MHz, DMSO-*d*$_6$)** δ 7.39 – 7.24 (m, 5H), 4.47 (s, 2H), 4.29 (d, *J* = 17.1 Hz, 2H), 4.11 (t, *J* = 2.4 Hz, 2H), 3.99 (d, *J* = 17.0 Hz, 2H), 3.00 (s, 3H), 1.67 (t, *J* = 2.5 Hz, 2H).

**$^{13}$C NMR (101 MHz, DMSO-*d*$_6$)** δ 168.5, 137.9, 128.2, 127.7, 127.5, 85.7, 75.4, 70.4, 62.1, 57.4, 45.8.

**ESI-MS**: calculated for $C_{16}H_{18}BNNaO_5^+$ $[M+Na]^+$, 338.1170; Found, 338.1171.

**6-methyl-2-(4-methylpent-2-yn-1-yl)-1,3,6,2-dioxazaborocane-4,8-dione (S-30)**

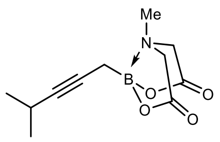

S-30

Following the **General procedure A**, the product **S-30** was obtained in 32% yield (from (3-methylbut-1-yne, 2 steps) as a white solid after column chromatography (eluent = ethyl acetate/petroleum ether v/v 2:1).

**$^1$H NMR (400 MHz, DMSO-*d*$_6$)** δ 4.29 (d, *J* = 17.0 Hz, 2H), 3.93 (d, *J* = 17.0 Hz, 2H), 3.00 (s, 3H), 2.48 – 2.40 (m, 1H), 1.51 (s, 2H), 1.04 (d, *J* = 7.0 Hz, 6H).

**$^{13}$C NMR (101 MHz, DMSO-*d*$_6$)** δ 168.6, 85.0, 77.7, 62.3, 45.8, 23.2, 19.9.

**ESI-MS**: calculated for $C_{11}H_{16}BNNaO_4^+$ [M+Na]$^+$, 260.1065; Found, 260.1063.

**6-methyl-2-(prop-2-yn-1-yl)-1,3,6,2-dioxazaborocane-4,8-dione (S-31)**

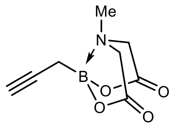

S-31

Following the **General procedure C**, the product **S-31** was obtained in 8% yield (from triethylsilylacetylene, 3 steps) as a white solid after column chromatography (eluent = ethyl acetate/petroleum ether v/v 4:1).

**$^1$H NMR (400 MHz, Acetone-*d*$_6$)** δ 4.28 (d, *J* = 16.8 Hz, 2H), 4.09 (d, *J* = 16.8 Hz, 2H), 3.28 (s, 3H), 2.16 (t, *J* = 2.9 Hz, 1H), 1.67 (s, 2H).

**$^{13}$C NMR (101 MHz, Acetone-*d*$_6$)** δ 168.4, 83.9, 69.4, 63.4, 46.4.

**ESI-MS**: calculated for $C_8H_{10}BKNO_4{}^+$ [M+K]$^+$, 234.0334; Found, 234.0334.

**6-methyl-2-(oct-1-yn-3-yl)-1,3,6,2-dioxazaborocane-4,8-dione (S-32)**

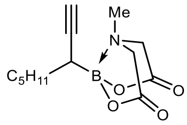

S-32

Following the **General procedure C**, the product **S-32** was obtained in 16% yield (from triethylsilylacetylene, 3 steps) as a white solid after column chromatography (eluent = ethyl acetate/petroleum ether v/v 4:1).

**¹H NMR (500 MHz, DMSO-$d_6$)** δ 4.35 (d, *J* = 17.3 Hz, 1H), 4.21 (d, *J* = 16.8 Hz, 1H), 4.01 (d, *J* = 17.3 Hz, 1H), 3.93 (d, *J* = 16.8 Hz, 1H), 3.04 (s, 3H), 2.67 (d, *J* = 2.5 Hz, 1H), 1.76 (dt, *J* = 10.4, 3.3 Hz, 1H), 1.62 – 1.17 (m, 8H), 0.87 (t, *J* = 6.8 Hz, 3H).

**¹³C NMR (126 MHz, DMSO-$d_6$)** δ 168.9, 168.3, 87.3, 71.8, 62.5, 62.3, 45.6, 31.0, 29.0, 28.0, 22.1, 14.0.

**ESI-MS**: calculated for $C_{13}H_{20}BNNaO_4^+$ $[M+Na]^+$, 288.1378; Found, 288.1382.

**6-methyl-2-(oct-3-yn-2-yl)-1,3,6,2-dioxazaborocane-4,8-dione (S-33)**

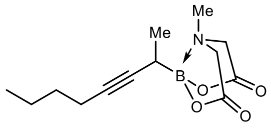


**S-33**

Following the **General procedure A**, the product **S-33** was obtained in 30% yield (from (1-hexyne, 2 steps) as a white solid after column chromatography (eluent = ethyl acetate/petroleum ether v/v 2:1).

**¹H NMR (400 MHz, DMSO-$d_6$)** δ 4.36 (d, *J* = 17.3 Hz, 1H), 4.22 (d, *J* = 16.8 Hz, 1H), 3.99 (d, *J* = 17.3 Hz, 1H), 3.85 (d, *J* = 16.7 Hz, 1H), 3.03 (s, 3H), 2.08 (dt, *J* = 6.8, 3.4 Hz, 2H), 1.82 – 1.72 (m, 1H), 1.40 – 1.27 (m, 4H), 1.07 (d, *J* = 7.2 Hz, 3H), 0.85 (t, *J* = 7.2 Hz, 3H).

**¹³C NMR (101 MHz, DMSO-$d_6$)** δ 169.0, 168.2, 83.9, 80.0, 62.5, 62.4, 45.5, 30.7, 21.4, 17.8, 15.8, 13.5.

**ESI-MS**: calculated for $C_{13}H_{20}BNNaO_4^+$ $[M+Na]^+$, 288.1378; Found, 288.1380.

**2-(6-((tert-butyldiphenylsilyl)oxy)hex-2-yn-1-yl)-6-methyl-1,3,6,2-dioxazaborocane-4,8-dione (S-34)**

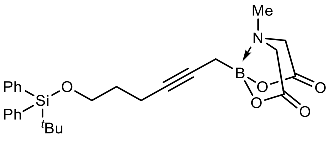


**S-34**

Following the **General procedure A**, the product **S-34** was obtained in 40% yield (from 4-pentyn-1-ol, 3 steps) as a white solid after column chromatography (eluent = ethyl

acetate/petroleum ether v/v 2:1).

**$^{1}$H NMR (400 MHz, DMSO-*d*$_6$)** δ 7.65 – 7.57 (m, 4H), 7.49 – 7.39 (m, 6H), 4.27 (d, *J* = 17.0 Hz, 2H), 3.92 (d, *J* = 17.0 Hz, 2H), 3.69 (t, *J* = 6.1 Hz, 2H), 2.96 (s, 3H), 2.20 (tt, *J* = 7.1, 2.6 Hz, 2H), 1.63 (tt, *J* = 6.6, 6.7 Hz, 2H), 1.52 (t, *J* = 2.5 Hz, 2H), 0.99 (s, 9H).

**$^{13}$C NMR (101 MHz, DMSO-*d*$_6$)** δ 168.5, 135.0, 133.2, 129.8, 127.9, 78.6, 78.5, 62.3, 62.1, 45.7, 31.6, 26.6, 18.8, 14.7.

**ESI-MS**: calculated for $C_{27}H_{34}BNNaO_5Si^+$ $[M+Na]^+$, 514.2192; Found, 514.2199.

**6-(6-methyl-4,8-dioxo-1,3,6,2-dioxazaborocan-2-yl)hex-4-yn-1-yl 4-methylbenzenesulfonate (S-35)**

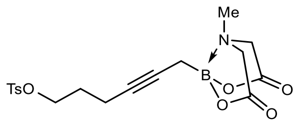


**S-35**

The product **S-35** was obtained in 74% yield (from **S-22**) as a white solid after column chromatography (eluent = ethyl acetate/petroleum ether v/v 2:1).

**$^{1}$H NMR (400 MHz, $CD_3CN$)** δ 7.81 – 7.75 (m, 2H), 7.48 – 7.40 (m, 2H), 4.05 (t, *J* = 6.1 Hz, 2H), 3.99 (d, *J* = 17.0 Hz, 2H), 3.85 (d, *J* = 17.0 Hz, 2H), 2.99 (s, 3H), 2.44 (s, 3H), 2.12 (tt, *J* = 7.0, 2.6 Hz, 2H), 1.76 – 1.67 (m, 2H), 1.53 (t, *J* = 2.6 Hz, 2H).

**$^{13}$C NMR (101 MHz, $CD_3CN$)** δ 168.9, 146.4, 133.8, 131.0, 128.8, 79.9, 78.7, 70.7, 63.4, 46.7, 29.0, 21.7, 15.5.

**ESI-MS**: calculated for $C_{18}H_{22}BNNaO_7S^+$ $[M+Na]^+$, 430.1102; Found, 430.1102.

**6-methyl-2-(pent-4-en-2-yn-1-yl)-1,3,6,2-dioxazaborocane-4,8-dione (S-37)**

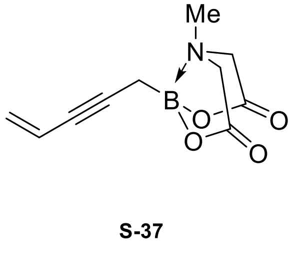


**S-37**

The product **S-37** was obtained in 74% yield (from **S-31**) as a yellow solid after column chromatography (eluent = ethyl acetate/petroleum ether v/v 3:2).

**$^1$H NMR (500 MHz, Acetone-*d*$_6$)** δ 5.81 – 5.73 (m, 1H), 5.44 (dd, *J* = 17.5, 2.3 Hz, 1H), 5.33 (dd, *J* = 11.1, 2.3 Hz, 1H), 4.29 (d, *J* = 16.9 Hz, 2H), 4.07 (d, *J* = 16.9 Hz, 2H), 3.27 (s, 3H), 1.80 (s, 2H).

**$^{13}$C NMR (101 MHz, Acetone-*d*$_6$)** δ 168.4, 125.4, 118.8, 90.5, 79.8, 63.3, 46.5.

**ESI-MS**: calculated for $C_{10}H_{12}BNNaO_4^+$ [M+Na]$^+$, 244.0752; Found, 244.0751.

**trifluoro(hept-2-yn-1-yl)borate potassium (S-41)**

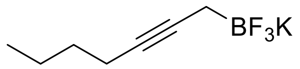


**S-41**

The product **S-41** was obtained in 73% yield (from **S-1**) as a white solid after recrystallization.

**$^1$H NMR (400 MHz, DMSO-*d*$_6$)** δ 2.05 – 1.96 (m, 2H), 1.39 – 1.25 (m, 4H), 0.89 – 0.82 (m, 3H), 0.77 (s, 2H).

**$^{13}$C NMR (101 MHz, DMSO-*d*$_6$)** δ 84.7, 74.7, 31.6, 21.5, 18.5, 13.7.

**$^{11}$B NMR (128 MHz, DMSO-*d*$_6$)** δ 3.34 (q, *J* = 56.5 Hz).

**$^{19}$F NMR (376 MHz, DMSO-*d*$_6$)** δ -136.80 (m)

**ESI-MS**: calculated for $C_7H_{11}BF_3^-$ [M-K]$^-$, 163.0911; Found, 163.0921.

**2-(hept-2-yn-1-yl)-2,3-dihydro-1H-naphtho[1,8-de][1,3,2]diazaborinine (S-42)**

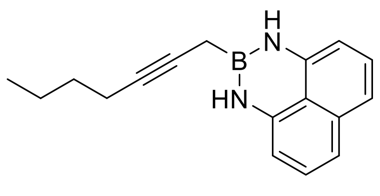


**S-42**

The product **S-42** was obtained in 92% yield (from **S-39**) as a yellow oil after column chromatography (eluent = ethyl acetate/petroleum ether v/v 1:50).

**$^1$H NMR (500 MHz, CDCl$_3$)** δ 7.12 (t, *J* = 7.8 Hz, 2H), 7.04 (d, *J* = 8.2 Hz, 2H), 6.33

(d, $J$ = 7.3 Hz, 2H), 5.82 (s, 2H), 2.25 (tt, $J$ = 7.0, 2.5 Hz, 2H), 1.87 (t, $J$ = 2.6 Hz, 2H), 1.60 – 1.44 (m, 4H), 0.98 (t, $J$ = 7.2 Hz, 3H).

**$^{13}$C NMR (126 MHz, $CDCl_3$)** δ 140.9, 136.4, 127.6, 119.8, 117.8, 105.9, 80.7, 76.7, 31.5, 22.2, 18.7, 13.8.

**ESI-MS**: calculated for $C_{17}H_{19}BN_2Na^+$ $[M+Na]^+$, 285.1534; Found, 285.1541.

## 3. General procedure for the synthesis of product

### 3.1 General procedure E for the synthesis of products in *Z* isomers:

In the glove box, to an oven-dried 15 mL vial were added the propargylic MIDA boronate (0.10 mmol), the silane (0.20 mmol), $[Cp^*Ru(MeCN)_3]PF_6$ (2.5 mg, 5.0 μmol), and THF or 1,4-dioxane (1.0 mL). The vial was capped and removed from the glove box. The reaction mixture was stirred at room temperature for 30 min, and then concentrated under reduced pressure. The residue was purified by silica gel flash column chromatography (ethyl acetate–petroleum as the eluent) to give the desired product.

### 3.2 General procedure F for the synthesis of products in *E* isomers:

In a glove box, to an oven-dried 15 mL vial were added the propargylic MIDA boronate (0.10 mmol), the silane (0.20 mmol), $CpRu(PPh_3)_2Cl$ (3.6 mg, 5.0 μmol), and 1,4-dioxane (1.0 mL). The vial was capped and removed from the glove box. The reaction mixture was stirred at room temperature for 3 h, and then concentrated under reduced pressure. The residue was purified by silica gel flash column chromatography (ethyl acetate–petroleum as the eluent) to give the desired product.

**(*Z*)-6-methyl-2-(2-(triethoxysilyl)hept-2-en-1-yl)-1,3,6,2-dioxazaborocane-4,8-dione (1)**

Following the **General procedure E**, the product **1** was obtained in 95% yield (39.5

mg, from **S-1**) as a colorless oil after column chromatography (eluent = ethyl acetate/petroleum ether v/v 4:1).

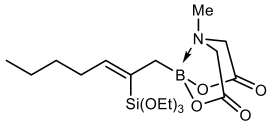


**1**

**$^{1}$H NMR (500 MHz, DMSO-*d*$_6$)** δ 6.05 (t, *J* = 7.6 Hz, 1H), 4.13 (d, *J* = 16.9 Hz, 2H), 3.88 (d, *J* = 16.9 Hz, 2H), 3.73 (q, *J* = 7.0 Hz, 6H), 2.81 (s, 3H), 2.19 – 2.09 (m, 2H), 1.48 (s, 2H), 1.32 – 1.28 (m, 4H), 1.13 (t, *J* = 7.0 Hz, 9H), 0.91 – 0.84 (m, 3H).

**$^{13}$C NMR (126 MHz, DMSO-*d*$_6$)** δ 168.8, 145.9, 128.8, 61.4, 57.5, 45.3, 31.7, 31.7, 22.0, 18.1, 18.1, 13.9.

**ESI-MS**: calculated for $C_{18}H_{34}BNNaO_7Si^+$ [M+Na]$^+$, 438.2090; Found, 438.2098.

**(*E*)-6-methyl-2-(2-(triethoxysilyl)hept-2-en-1-yl)-1,3,6,2-dioxazaborocane-4,8-dione (2)**

Following the **General procedure F**, the product **2** was obtained in 84% yield (34.9 mg, from **S-1**) as a colorless oil after column chromatography (eluent = ethyl acetate/petroleum ether v/v 4:1).

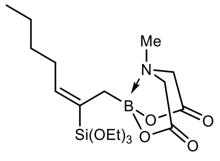


**2**

**$^{1}$H NMR (500 MHz, CD$_3$CN)** δ 5.98 (t, *J* = 6.8 Hz, 1H), 3.89 (d, *J* = 16.8 Hz, 2H), 3.82 – 3.75 (m, 8H), 2.92 (s, 3H), 2.16 – 2.10 (m, 2H), 1.64 (s, 2H), 1.41 – 1.32 (m, 4H), 1.17 (t, *J* = 7.0 Hz, 9H), 0.90 (t, *J* = 7.2 Hz, 3H).

**$^{13}$C NMR (126 MHz, CD$_3$CN)** δ 169.1, 146.3, 130.3, 62.6, 59.1, 46.2, 32.0, 29.5, 23.2, 18.5, 14.3.

**ESI-MS**: calculated for $C_{18}H_{34}BNNaO_7Si^+$ [M+Na]$^+$, 438.2090; Found, 438.2106.

**(*Z*)-6-methyl-2-(2-(triethylsilyl)hept-2-en-1-yl)-1,3,6,2-dioxazaborocane-4,8-dione (3)**

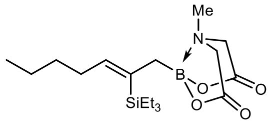

**3**

Following the **General procedure E**, the product **3** was obtained in 95% yield (34.9 mg, from **S-1**) as a colorless oil after column chromatography (eluent = ethyl acetate/petroleum ether v/v 4:1).

**$^{1}$H NMR (400 MHz, Acetone-*d*$_6$)** δ 6.07 (tt, *J* = 7.5, 1.2 Hz, 1H), 4.14 (d, *J* = 16.7 Hz, 2H), 3.98 (d, *J* = 16.8 Hz, 2H), 3.08 (s, 3H), 2.16 – 2.09 (m, 2H), 1.58 (s, 2H), 1.39 – 1.32 (m, 4H), 1.00 – 0.87 (m, 12H), 0.72 (q, *J* = 7.7,6H).

**$^{13}$C NMR (101 MHz, DMSO-*d*$_6$)** δ 168.9, 142.7, 132.6, 61.4, 45.4, 32.1, 31.6, 21.9, 14.0, 7.7, 4.0.

**$^{11}$B NMR (128 MHz, Acetone-*d*$_6$)** δ 12.28.

**ESI-MS**: calculated for $C_{18}H_{34}BNNaO_4Si^+$ [M+Na]$^+$, 390.2242; Found, 390.2249.

**(*Z*)-6-methyl-2-(2-(tributylsilyl)hept-2-en-1-yl)-1,3,6,2-dioxazaborocane-4,8-dione (4)**

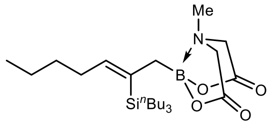

**4**

Following the **General procedure E**, the product **4** was obtained in 77% yield (34.7 mg, from **S-1**) as a colorless oil after column chromatography (eluent = ethyl acetate/petroleum ether v/v 4:1).

**$^{1}$H NMR (400 MHz, Acetone-*d*$_6$)** δ 6.05 (t, *J* = 7.5 Hz, 1H), 4.13 (d, *J* = 16.7 Hz, 2H), 3.96 (d, *J* = 16.8 Hz, 2H), 3.07 (s, 3H), 2.17 – 2.10 (m, 2H), 1.58 (s, 2H), 1.39 – 1.29 (m, 16H), 0.92 – 0.85 (m, 12H), 0.81 – 0.66 (m, 6H).

**$^{13}$C NMR (101 MHz, Acetone-*d*$_6$)** δ 168.7, 143.9, 134.4, 62.5, 46.1, 33.3, 33.0, 27.5, 27.1, 23.2, 14.4, 14.1, 14.0.

**ESI-MS**: calculated for $C_{24}H_{46}BNNaO_4Si^+$ [M+Na]$^+$, 474.3181; Found, 474.3186.

**(*Z*)-2-(2-(benzyldimethylsilyl)hept-2-en-1-yl)-6-methyl-1,3,6,2-dioxazaborocane-4,8-dione (5)**

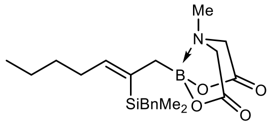


**5**

Following the **General procedure E**, the product **5** was obtained in 95% yield (38.1 mg, from **S-1**) as a colorless oil after column chromatography (eluent = ethyl acetate/petroleum ether v/v 4:1).

**$^1$H NMR (400 MHz, DMSO-*d*$_6$)** δ 7.20 – 7.14 (m, 2H), 7.06 – 6.99 (m, 3H), 5.91 (t, *J* = 7.5 Hz, 1H), 4.17 (d, *J* = 17.0 Hz, 2H), 3.96 (d, *J* = 16.9 Hz, 2H), 2.83 (s, 3H), 2.22 (s, 2H), 2.01 – 1.95 (m, 2H), 1.47 (s, 2H), 1.29 – 1.22 (m, 4H), 0.87 – 0.83 (m, 3H), 0.02 (s, 6H).

**$^{13}$C NMR (101 MHz, DMSO-*d*$_6$)** δ 168.9, 142.5, 140.4, 134.4, 128.1, 127.9, 123.7, 61.5, 45.5, 32.0, 31.7, 25.7, 21.9, 13.9, -1.7.

**ESI-MS**: calculated for $C_{21}H_{32}BNNaO_4Si^+$ $[M+Na]^+$, 424.2086; Found, 424.2099.

**(*Z*)-2-(2-(dimethyl(phenyl)silyl)hept-2-en-1-yl)-6-methyl-1,3,6,2-dioxazaborocane-4,8-dione (6)**

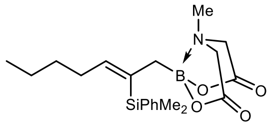


**6**

Following the **General procedure E**, the product **6** was obtained in 90% yield (35.0 mg, from **S-1**) as a white solid after column chromatography (eluent = ethyl acetate/petroleum ether v/v 4:1).

**$^1$H NMR (500 MHz, DMSO-*d*$_6$)** δ 7.54 – 7.50 (m, 2H), 7.33 – 7.28 (m, 3H), 5.96 (t, *J* = 7.5 Hz, 1H), 4.17 (d, *J* = 17.0 Hz, 2H), 3.95 (d, *J* = 17.0 Hz, 2H), 2.81 (s, 3H), 1.83-1.75 (m, 2H), 1.57 (s, 2H), 1.10 – 0.98 (m, 4H), 0.70 – 0.63 (m, 3H), 0.32 (s, 6H).

**$^{13}$C NMR (126 MHz, DMSO-*d*$_6$)** δ 168.9, 142.9, 140.5, 133.7, 133.6, 128.4, 127.5, 61.5, 45.5, 31.9, 31.5, 21.7, 13.8, -0.9.

**$^{11}$B NMR (128 MHz, Acetone-*d*$_6$)** δ 12.40.

**ESI-MS**: calculated for $C_{20}H_{30}BNNaO_4Si^+$ $[M+Na]^+$, 410.1929; Found, 410.1927.

**(*Z*)-2-(2-(ethoxydimethylsilyl)hept-2-en-1-yl)-6-methyl-1,3,6,2-dioxazaborocane-4,8-dione (7)**

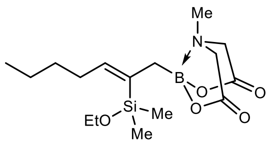

**7**

Following the **General procedure E**, the product **7** was obtained in 96% yield (34.2 mg, from **S-1**) as a white solid after column chromatography (eluent = ethyl acetate/petroleum ether v/v 4:1).

**$^1$H NMR (400 MHz, Acetone-*d*$_6$)** δ 6.04 (tt, $J$ = 7.5, 1.3 Hz, 1H), 4.14 (d, $J$ = 16.7 Hz, 2H), 3.96 (d, $J$ = 16.7 Hz, 2H), 3.64 (q, $J$ = 7.0 Hz, 2H), 3.09 (s, 3H), 2.22 (td, $J$ = 9.2, 8.4, 5.6 Hz, 2H), 1.63 (s, 2H), 1.39 – 1.32 (m, 4H), 1.13 (t, $J$ = 7.0 Hz, 3H), 0.93 – 0.86 (m, 3H), 0.22 (s, 6H).

**$^{13}$C NMR (101 MHz, Acetone-*d*$_6$)** δ 168.7, 144.4, 135.7, 62.6, 58.4, 46.3, 33.2, 32.5, 23.2, 18.8, 14.4, -0.1.

**ESI-MS**: calculated for $C_{16}H_{30}BNNaO_5Si^+$ $[M+Na]^+$, 378.1879; Found, 378.1882.

**(*Z*)-2-(2-(dimethoxy(methyl)silyl)hept-2-en-1-yl)-6-methyl-1,3,6,2-dioxazaborocane-4,8-dione (8)**

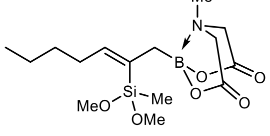

**8**

Following the **General procedure E**, the product **8** was obtained in 78% yield (27.7 mg, from **S-1**) as a colorless oil after column chromatography (eluent = ethyl acetate).

**$^1$H NMR (400 MHz, DMSO-*d*$_6$)** δ 5.98 (t, $J$ = 7.6 Hz, 1H), 4.14 (d, $J$ = 16.9 Hz, 2H), 3.90 (d, $J$ = 16.9 Hz, 2H), 3.37 (s, 6H), 2.81 (s, 3H), 2.14 (q, $J$ = 7.1 Hz, 2H), 1.49 (s, 2H), 1.33 – 1.25 (m, 4H), 0.90 – 0.84 (m, 3H), 0.15 (s, 3H).

**$^{13}$C NMR (101 MHz, DMSO-*d*$_6$)** δ 168.8, 144.2, 131.9, 61.4, 49.5, 45.3, 31.9, 31.1, 21.9, 13.9, -3.9.

**ESI-MS**: calculated for $C_{15}H_{28}BNNaO_6Si^+$ $[M+Na]^+$, 380.1671; Found, 380.1670.

**(*Z* & *E*)-2-(2-(1,1,1,3,5,5,5-heptamethyltrisiloxan-3-yl)hept-2-en-1-yl)-6-methyl-1,3,6,2-dioxazaborocane-4,8-dione (9)**

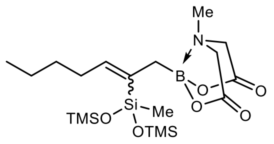


**9**

Following the **General procedure E**, the product **9** was obtained in 97% yield (45.7 mg, from **S-1**) as a colorless oil after column chromatography (eluent = ethyl acetate/petroleum ether v/v 2:1, *Z/E* = 3:1).

**$^{1}$H NMR (500 MHz, Acetone-*d*$_6$)** (*Z* isomer) δ 6.01 (t, *J* = 7.6 Hz, 1H), 3.92 (d, *J* = 16.8 Hz, 2H), 3.08 (s, 3H), 2.24 – 2.19 (m, 2H), 1.62 (s, 2H), 0.20 (s, 3H). (*E* isomer) 5.87 (t, *J* = 6.6 Hz, 1H), 4.01 (d, *J* = 16.8 Hz, 2H), 3.12 (s, 3H), 2.16 – 2.10 (m, 2H), 1.69 (s, 2H). (overlap) 4.14 (dd, *J* = 16.8, 7.6 Hz, 2H), 1.42 – 1.31 (m, 4H), 0.91 (t, *J* = 6.6 Hz, 3H), 0.17 – 0.19 (m, 18H).

**$^{13}$C NMR (126 MHz, Acetone-*d*$_6$)** δ 168.6, 168.6, 144.2, 142.0, 137.1, 136.0, 62.7, 62.4, 46.3, 46.0, 33.1, 33.1, 32.3, 23.3, 23.2, 14.4, 14.3, 2.2, 2.2, 2.1. (mixture of *Z* & *E* isomers)

**ESI-MS**: calculated for $C_{19}H_{40}BNNaO_6Si_3^+$ $[M+Na]^+$, 496.2149; Found, 496.2146.

**(*Z*)-6-methyl-2-(2-(trimethoxysilyl)hept-2-en-1-yl)-1,3,6,2-dioxazaborocane-4,8-dione (10)**

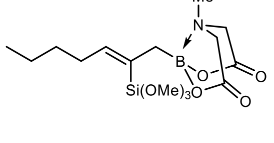


**10**

Following the **General procedure E**, the product **10** was obtained in 84% yield (31.3 mg, from **S-1**) as a colorless oil after column chromatography (eluent = ethyl acetate).

**$^1$H NMR (400 MHz, Acetone-*d*$_6$)** δ 6.18 (tt, *J* = 7.6, 1.3 Hz, 1H), 4.13 (d, *J* = 16.7 Hz, 2H), 3.93 (d, *J* = 16.6 Hz, 2H), 3.52 (s, 9H), 3.07 (s, 3H), 2.24 – 2.16 (m, 2H), 1.63 (s, 2H), 1.40 – 1.32 (m, 4H), 0.94 – 0.87 (m, 3H).

**$^{13}$C NMR (101 MHz, Acetone-*d*$_6$)** δ 168.7, 147.8, 128.9, 62.6, 62.5, 50.5, 45.9, 32.9, 32.9, 23.1, 14.3.

**ESI-MS**: calculated for $C_{15}H_{28}BNNaO_7Si^+$ $[M+Na]^+$, 396.1620; Found, 396.1620.

**(*Z*)-6-methyl-2-(2-(triisopropoxysilyl)hept-2-en-1-yl)-1,3,6,2-dioxazaborocane-4,8-dione (11)**

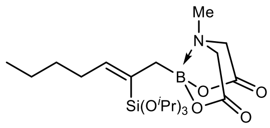


**11**

Following the **General procedure E**, the product **11** was obtained in 60% yield (27.5 mg, from **S-1**) as a colorless oil after column chromatography (eluent = ethyl acetate/petroleum ether v/v 2:1).

**$^1$H NMR (400 MHz, Acetone-*d*$_6$)** δ 6.16 (tt, *J* = 7.6, 1.3 Hz, 1H), 4.32 (hept, *J* = 6.0 Hz, 3H), 4.13 (d, *J* = 16.6 Hz, 2H), 3.89 (d, *J* = 16.6 Hz, 2H), 3.08 (s, 3H), 2.25 (q, *J* = 7.6, 7.2 Hz, 2H), 1.66 (s, 2H), 1.40 – 1.33 (m, 4H), 1.19 (d, *J* = 6.1 Hz, 18H), 0.94 – 0.86 (m, 3H).

**$^{13}$C NMR (101 MHz, Acetone-*d*$_6$)** δ 168.6, 147.1, 130.8, 65.6, 62.6, 46.2, 33.5, 32.8, 26.0, 23.4, 14.4.

**ESI-MS**: calculated for $C_{21}H_{40}BNNaO_7Si^+$ $[M+Na]^+$, 480.2559; Found, 480.2562.

**(*Z*)-6-methyl-2-(2-(triethoxysilyl)hex-2-en-1-yl)-1,3,6,2-dioxazaborocane-4,8-dione (12)**

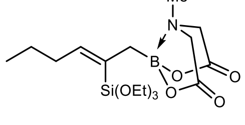


**12**

Following the **General procedure E**, the product **12** was obtained in 92% yield (36.8

mg, from **S-2**) as a colorless oil after column chromatography (eluent = ethyl acetate/petroleum ether v/v 4:1).

**$^{1}$H NMR (500 MHz, Acetone-*d$_6$*)** δ 6.17 (t, *J* = 7.7 Hz, 1H), 4.12 (d, *J* = 16.6 Hz, 2H), 3.91 (d, *J* = 16.6 Hz, 2H), 3.83 (q, *J* = 7.0 Hz, 6H), 3.08 (s, 3H), 2.23 – 2.19 (m, 2H), 1.66 (s, 2H), 1.42 – 1.36 (m, 2H), 1.18 (t, *J* = 7.0 Hz, 9H), 0.92 (t, *J* = 7.4 Hz, 3H).

**$^{13}$C NMR (126 MHz, Acetone-*d$_6$*)** δ 168.7, 147.3, 130.0, 62.6, 58.7, 46.0, 35.3, 23.8, 18.6, 14.2.

**ESI-MS**: calculated for $C_{17}H_{32}BNNaO_7Si^+$ [M+Na]$^+$, 424.1933; Found, 424.1928.

**(*Z*)-6-methyl-2-(2-(triethoxysilyl)but-2-en-1-yl)-1,3,6,2-dioxazaborocane-4,8-dione (13)**

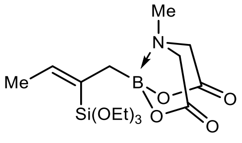


**13**

Following the **General procedure E**, the product **13** was obtained in 87% yield (32.4 mg, from **S-3**) as a colorless oil after column chromatography (eluent = ethyl acetate/petroleum ether v/v 4:1).

**$^{1}$H NMR (500 MHz, Acetone-*d$_6$*)** δ 6.23 (q, *J* = 7.0 Hz, 1H), 4.13 (d, *J* = 16.6 Hz, 2H), 3.92 (d, *J* = 16.8 Hz, 2H), 3.82 (q, *J* = 7.0 Hz, 6H), 3.07 (s, 3H), 1.81 (d, *J* = 7.0 Hz, 3H), 1.64 (s, 2H), 1.18 (t, *J* = 7.0 Hz, 9H).

**$^{13}$C NMR (126 MHz, Acetone-*d$_6$*)** δ 168.7, 141.1, 131.2, 62.5, 58.6, 45.9, 18.5, 18.4.

**$^{11}$B NMR (128 MHz, Acetone-*d$_6$*)** δ 12.33.

**ESI-MS**: calculated for $C_{15}H_{28}BNNaO_7Si^+$ [M+Na]$^+$, 396.1620; Found, 396.1616.

**(*Z*)-2-(6-chloro-2-(triethoxysilyl)hex-2-en-1-yl)-6-methyl-1,3,6,2-dioxazaborocane-4,8-dione (14)**

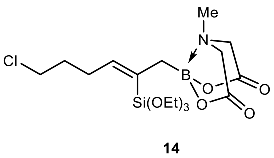


**14**

Following the **General procedure E**, the product **14** was obtained in 83% yield (36.2 mg, from **S-4**) as a colorless oil after column chromatography (eluent = ethyl acetate/petroleum ether v/v 4:1).

**$^1$H NMR (500 MHz, Acetone-*d*$_6$)** δ 6.15 (t, *J* = 7.7 Hz, 1H), 4.14 (d, *J* = 16.8 Hz, 2H), 3.93 (d, *J* = 16.6 Hz, 2H), 3.84 (q, *J* = 7.0 Hz, 6H), 3.61 (t, *J* = 6.9 Hz, 2H), 3.08 (s, 3H), 2.40 – 2.35 (m, 2H), 1.84 (tt, *J* = 6.9, 7.0 Hz, 2H), 1.67 (s, 2H), 1.19 (t, *J* = 7.0 Hz, 9H).

**$^{13}$C NMR (126 MHz, Acetone-*d*$_6$)** δ 168.7, 145.3, 131.6, 62.5, 58.8, 46.0, 45.4, 33.8, 30.6, 18.6.

**ESI-MS**: calculated for $C_{17}H_{31}BClNNaO_7Si^+$ $[M+Na]^+$, 458.1544; Found, 458.1554.

**(*Z*)-2-(8-chloro-2-(triethylsilyl)oct-2-en-1-yl)-6-methyl-1,3,6,2-dioxazaborocane-4,8-dione (15)**

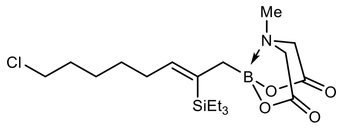


**15**

Following the **General procedure E,** the product **15** was obtained in 68% yield (28.2 mg, from **S-5**) as a colorless oil after column chromatography (eluent = ethyl acetate/petroleum ether v/v 4:1).

**$^1$H NMR (400 MHz, DMSO-*d*$_6$)** δ 5.94 (t, *J* = 7.4 Hz, 1H), 4.13 (d, *J* = 17.0 Hz, 2H), 3.94 (d, *J* = 17.0 Hz, 2H), 3.62 (t, *J* = 6.7 Hz, 2H), 2.80 (s, 3H), 2.10 – 2.02 (m, 2H), 1.71 (tt, *J* = 6.8, 6.8 Hz, 2H), 1.49 – 1.26 (m, 6H), 0.91 (t, *J* = 7.8 Hz, 9H), 0.64 (q, *J* = 7.9 Hz, 6H).

**$^{13}$C NMR (101 MHz, DMSO-*d*$_6$)** δ 168.9, 142.4, 132.9, 61.4, 45.4, 45.3, 32.1, 31.7, 29.1, 26.1, 7.7, 4.0.

**ESI-MS**: calculated for $C_{19}H_{35}BClNNaO_4Si^+$ $[M+Na]^+$, 438.2009; Found, 438.2011.

**(*Z*)-2-(6-bromo-2-(triethoxysilyl)hex-2-en-1-yl)-6-methyl-1,3,6,2-dioxazaborocane-4,8-dione (16)**

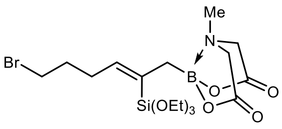


**16**

Following the **General procedure E,** the product **16** was obtained in 54% yield (26.0 mg, from **S-6**) as a colorless oil after column chromatography (eluent = ethyl acetate/petroleum ether v/v 4:1).

**$^{1}$H NMR (400 MHz, Acetone-*d*$_6$)** δ 6.16 (tt, *J* = 7.6, 1.3 Hz, 1H), 4.14 (d, *J* = 16.7 Hz, 2H), 3.93 (d, *J* = 16.7 Hz, 2H), 3.85 (q, *J* = 7.0 Hz, 6H), 2.42 – 2.35 (m, 2H), 1.98 – 1.90 (m, 2H), 1.67 (s, 2H), 1.20 (t, *J* = 7.0 Hz, 9H).

**$^{13}$C NMR (101 MHz, Acetone-*d*$_6$)** δ 168.7, 145.2, 131.7, 62.6, 58.8, 46.0, 34.3, 34.1, 31.9, 18.6.

**ESI-MS**: calculated for $C_{17}H_{31}BBrNNaO_7Si^+$ [M+Na]$^+$, 502.1038; Found, 502.1045.

**(*Z*)-6-methyl-2-(6-phenyl-2-(triethoxysilyl)hex-2-en-1-yl)-1,3,6,2-dioxazaborocane-4,8-dione (17)**

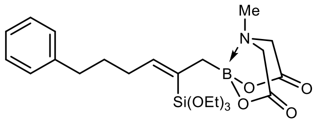


**17**

Following the **General procedure E,** the product **17** was obtained in 74% yield (34.9 mg, from **S-7**) as a colorless oil after column chromatography (eluent = ethyl acetate/petroleum ether v/v 4:1).

**$^{1}$H NMR (400 MHz, DMSO-*d*$_6$)** δ 7.26 (td, *J* = 7.3, 1.5 Hz, 2H), 7.21 – 7.11 (m, 3H), 6.09 (t, *J* = 7.6 Hz, 1H), 4.14 (d, *J* = 16.9 Hz, 2H), 3.89 (d, *J* = 16.9 Hz, 2H), 3.72 (q, *J* = 7.0 Hz, 6H), 2.82 (s, 3H), 2.62 – 2.54 (m, 2H), 2.24 – 2.16 (m, 2H), 1.66 – 1.53 (m, 2H), 1.50 (s, 2H), 1.12 (t, *J* = 7.0 Hz, 9H).

**$^{13}$C NMR (101 MHz, DMSO-*d*$_6$)** δ 168.7, 145.5, 142.3, 129.3, 128.2, 128.2, 125.6, 61.4, 57.5, 45.3, 35.1, 31.7, 31.6, 18.1.

**ESI-MS**: calculated for $C_{23}H_{36}BNNaO_7Si^+$ [M+Na]$^+$, 500.2246; Found, 500.2243.

**(*Z*)-6-methyl-2-(5-phenyl-2-(triethoxysilyl)pent-2-en-1-yl)-1,3,6,2-dioxazaborocane-4,8-dione (18)**

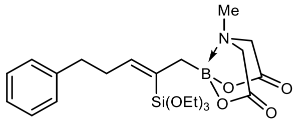


**18**

Following the **General procedure E,** the product **18** was obtained in 82% yield (38.1 mg, from **S-8**) as a colorless oil after column chromatography (eluent = ethyl acetate/petroleum ether v/v 4:1).

**$^{1}$H NMR (400 MHz, $CDCl_3$)** δ 7.36 – 7.15 (m, 5H), 6.31 (t, *J* = 7.5 Hz, 1H), 3.91 – 3.79 (m, 8H), 3.65 (d, *J* = 16.2 Hz, 2H), 2.88 (s, 3H), 2.77 – 2.69 (m, 2H), 2.66 – 2.56 (m, 2H), 1.72 (s, 2H), 1.25 (t, *J* = 7.1 Hz, 9H).

**$^{13}$C NMR (101 MHz, $CDCl_3$)** δ 167.7, 147.7, 142.1, 128.7, 128.4, 128.4, 125.8, 62.1, 58.5, 45.4, 36.0, 34.2, 18.3.

**ESI-MS**: calculated for $C_{22}H_{34}BNNaO_7Si^+$ $[M+Na]^+$, 486.2090; Found, 486.2088.

**(*Z*)-6-methyl-2-(2-(triethoxysilyl)-5-(3-(trifluoromethyl)phenyl)pent-2-en-1-yl)-1,3,6,2-dioxazaborocane-4,8-dione (19)**

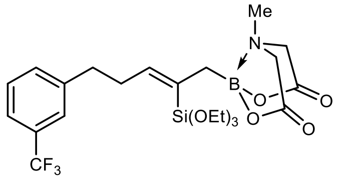


**19**

Following the **General procedure E,** the product **19** was obtained in 94% yield (49.9 mg, from **S-9**) as a colorless oil after column chromatography (eluent = ethyl acetate/petroleum ether v/v 4:1).

**$^{1}$H NMR (400 MHz, $CD_3CN$)** δ 7.57 – 7.43 (m, 4H), 6.18 (tt, *J* = 7.6, 1.3 Hz, 1H), 3.90 (d, *J* = 16.6 Hz, 2H), 3.77 (q, *J* = 7.0 Hz, 6H), 3.72 (d, *J* = 16.6 Hz, 2H), 2.83 (s, 3H), 2.78 – 2.71 (m, 2H), 2.59 – 2.48 (m, 2H), 1.59 (s, 2H), 1.16 (t, *J* = 7.0 Hz, 9H).

**$^{13}$C NMR (101 MHz, $CD_3CN$)** δ 169.0, 145.9, 144.6, 133.5, 131.5, 130.8 (q, *J* = 31.5 Hz), 130.1, 126.0 (q, *J* = 3.9 Hz), 125.6 (q, *J* = 271.4 Hz), 123.5 (q, *J* = 4.0 Hz), 62.6,

59.0, 46.3, 36.5, 35.1, 18.6.

**$^{19}$F NMR (376 MHz, $CD_3CN$)** δ -62.96.

**ESI-MS**: calculated for $C_{23}H_{34}BF_3NO_7Si^+$ $[M+H]^+$, 532.2144; Found, 532.2160.

**(*Z*)-2-(5-(4-methoxyphenoxy)-2-(triethoxysilyl)pent-2-en-1-yl)-6-methyl-1,3,6,2-dioxazaborocane-4,8-dione (20)**

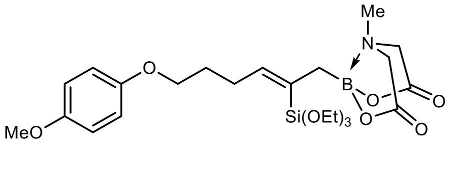


**20**

Following the **General procedure E,** the product **20** was obtained in 96% yield (50.1 mg, from **S-10**) as a colorless oil after column chromatography (eluent = ethyl acetate/petroleum ether v/v 4:1).

**$^1$H NMR (400 MHz, DMSO-*d*$_6$)** δ 6.84 – 6.82 (m, 4H), 6.09 (t, *J* = 7.6 Hz, 1H), 4.15 (d, *J* = 16.9 Hz, 2H), 3.95 – 3.85 (m, 4H), 3.74 (q, *J* = 7.0 Hz, 6H), 3.68 (s, 3H), 2.82 (s, 3H), 2.30 (q, *J* = 7.5 Hz, 2H), 1.79 – 1.67 (m, 2H), 1.51 (s, 2H), 1.13 (t, *J* = 7.0 Hz, 9H).

**$^{13}$C NMR (101 MHz, DMSO-*d*$_6$)** δ 168.7, 153.2, 152.6, 144.9, 129.7, 115.2, 114.6, 67.5, 61.4, 57.6, 55.3, 45.3, 29.2, 28.5, 18.1.

**ESI-MS**: calculated for $C_{24}H_{38}BNNaO_9Si^+$ $[M+Na]^+$, 546.2301; Found, 546.2305.

**(*Z*)-4-((6-(6-methyl-4,8-dioxo-1,3,6,2-dioxazaborocan-2-yl)-5-(triethoxysilyl)hex-4-en-1-yl)oxy)benzonitrile (21)**

Following the **General procedure E,** the product **21** was obtained in 83% yield (43.0 mg, from **S-11**) as a colorless oil after column chromatography (eluent = ethyl acetate/petroleum ether v/v 4:1).

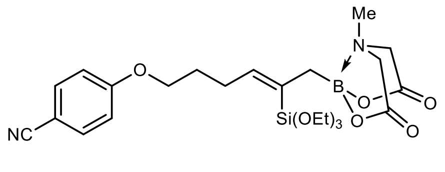

**21**

**$^1$H NMR (400 MHz, DMSO-*d*$_6$)** δ 7.74 (d, *J* = 9.0 Hz, 2H), 7.08 (d, *J* = 8.9 Hz, 2H), 6.09 (t, *J* = 7.6 Hz, 1H), 4.15 (d, *J* = 16.9 Hz, 2H), 4.07 (t, *J* = 6.6 Hz, 2H), 3.90 (d, *J* = 16.9 Hz, 2H), 3.73 (q, *J* = 7.0 Hz, 6H), 2.83 (s, 3H), 2.35 – 2.29 (m, 2H), 1.84 – 1.74 (m, 2H), 1.51 (s, 2H), 1.12 (t, *J* = 7.0 Hz, 9H).

**$^{13}$C NMR (101 MHz, DMSO-*d*$_6$)** δ 168.8, 162.1, 144.6, 134.2, 134.2, 130.1, 119.2, 115.5, 102.6, 67.6, 61.4, 57.7, 57.6, 45.3, 28.8, 28.3, 18.1, 18.0.

**ESI-MS**: calculated for $C_{24}H_{35}BN_2NaO_8Si^+$ $[M+Na]^+$, 541.2148; Found, 541.2143.

**(*Z*)-6-methyl-2-(5-(5-methylfuran-2-yl)-2-(triethylsilyl)pent-2-en-1-yl)-1,3,6,2-dioxazaborocane-4,8-dione (22)**

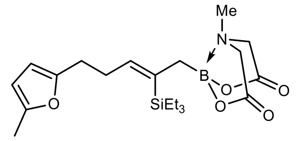

**22**

Following the **General procedure E,** the product **22** was obtained in 93% yield (39.0 mg, from **S-12**) as a colorless oil after column chromatography (eluent = ethyl acetate/petroleum ether v/v 4:1).

**$^1$H NMR (500 MHz, DMSO-*d*$_6$)** δ 5.97 (t, *J* = 7.3 Hz, 1H), 5.94 – 5.87 (m, 2H), 4.13 (d, *J* = 16.9 Hz, 2H), 3.93 (d, *J* = 16.9 Hz, 2H), 2.80 (s, 3H), 2.56 (t, *J* = 7.6 Hz, 2H), 2.36 – 2.31 (m, 2H), 2.19 (s, 3H), 1.45 (s, 2H), 0.90 (t, *J* = 7.9 Hz, 9H), 0.65 (q, *J* = 7.9 Hz, 6H).

**$^{13}$C NMR (101 MHz, DMSO-*d*$_6$)** δ 168.8, 153.2, 149.6, 141.1, 134.2, 106.1, 105.7, 61.4, 45.4, 30.6, 28.2, 13.2, 7.6, 3.9.

**ESI-MS**: calculated for $C_{21}H_{34}BNNaO_5Si^+$ $[M+Na]^+$, 442.2192; Found, 442.2193.

**(*Z*)-2-(5,5-dimethyl-2-(triethoxysilyl)hex-2-en-1-yl)-6-methyl-1,3,6,2-dioxazaborocane-4,8-dione (23)**

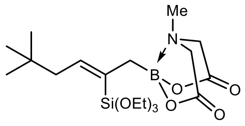

23

Following the **General procedure E,** the product **23** was obtained in 95% yield (40.6 mg, from **S-13**) as a colorless oil after column chromatography (eluent = ethyl acetate/petroleum ether v/v 4:1).

**$^{1}$H NMR (400 MHz, Acetone-*d*$_6$)** δ 6.28 (t, *J* = 7.6 Hz, 1H), 4.13 (d, *J* = 16.7 Hz, 2H), 3.93 (d, *J* = 16.7 Hz, 2H), 3.83 (q, *J* = 7.0 Hz, 6H), 3.08 (s, 3H), 2.18 (d, *J* = 7.4 Hz, 2H), 1.67 (s, 2H), 1.18 (t, *J* = 7.0 Hz, 9H), 0.93 (s, 9H).

**$^{13}$C NMR (101 MHz, DMSO-*d*$_6$)** δ 168.8, 143.1, 130.1, 61.4, 57.5, 45.3, 45.3, 30.8, 29.3, 18.1, 18.1.

**$^{11}$B NMR (128 MHz, Acetone-*d*$_6$)** δ 12.41.

**ESI-MS**: calculated for $C_{19}H_{36}BNNaO_7Si^+$ $[M+Na]^+$, 452.2246; Found, 452.2249.

**(*Z*)-2-(4-cyclohexyl-2-(triethylsilyl)but-2-en-1-yl)-6-methyl-1,3,6,2-dioxazaborocane-4,8-dione (24)**

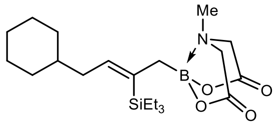

24

Following the **General procedure E,** the product **24** was obtained in 58% yield (23.6 mg, from **S-14**) as a colorless oil after column chromatography (eluent = ethyl acetate/petroleum ether v/v 4:1).

**$^{1}$H NMR (500 MHz, DMSO-*d*$_6$)** δ 5.98 (t, *J* = 7.3 Hz, 1H), 4.12 (d, *J* = 16.9 Hz, 2H), 3.94 (d, *J* = 16.9 Hz, 2H), 2.79 (s, 3H), 1.95 (t, *J* = 7.0 Hz, 2H), 1.77 – 1.58 (m, 5H), 1.42 (s, 2H), 1.29 – 1.07 (m, 4H), 0.90 (t, *J* = 7.9 Hz, 9H), 0.63 (q, *J* = 7.9 Hz, 6H).

**$^{13}$C NMR (101 MHz, DMSO-*d*$_6$)** δ 168.9, 141.6, 133.2, 61.4, 45.3, 38.6, 32.8, 26.1, 26.0, 7.7, 4.0.

**ESI-MS**: calculated for $C_{21}H_{38}BNNaO_4Si^+$ $[M+Na]^+$, 430.2555; Found, 430.2559.

**(*Z*)-6-methyl-2-(5,7,7-trimethyl-2-(triethoxysilyl)oct-2-en-1-yl)-1,3,6,2-dioxazaborocane-4,8-dione (25)**

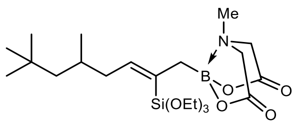


**25**

Following the **General procedure E,** the product **25** was obtained in 60% yield (28.3 mg, from **S-15**) as a colorless oil after column chromatography (eluent = ethyl acetate/petroleum ether v/v 4:1).

**$^1$H NMR (400 MHz, Acetone-*d*$_6$)** δ 6.18 (tt, *J* = 7.3, 1.3 Hz, 1H), 4.12 (d, *J* = 16.6 Hz, 2H), 3.92 (dd, *J* = 16.8, 0.9 Hz, 2H), 3.83 (q, *J* = 7.0 Hz, 6H), 3.07 (s, 3H), 2.28 – 2.08 (m, 2H), 1.70 – 1.58 (m, 3H), 1.37 (dd, *J* = 13.9, 3.4 Hz, 1H), 1.18 (t, *J* = 7.0 Hz, 9H), 1.05 (dd, *J* = 14.0, 6.9 Hz, 1H), 0.96 (d, *J* = 6.6 Hz, 3H), 0.92 (s, 9H).

**$^{13}$C NMR (101 MHz, Acetone-*d*$_6$)** δ 168.7, 146.6, 130.7, 62.5, 58.6, 51.5, 46.0, 42.6, 31.6, 30.8, 30.5, 23.0, 18.6.

**ESI-MS**: calculated for $C_{22}H_{42}BNNaO_7Si^+$ $[M+Na]^+$, 494.2716; Found, 494.2703.

**(*Z*)-7-(6-methyl-4,8-dioxo-1,3,6,2-dioxazaborocan-2-yl)-6-(triethoxysilyl)hept-5-enenitrile (26)**

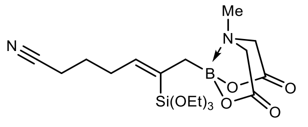


**26**

Following the **General procedure E,** the product **26** was obtained in 84% yield (35.8 mg, from **S-16**) as a colorless oil after column chromatography (eluent = ethyl acetate/petroleum ether v/v 4:1).

**$^1$H NMR (500 MHz, DMSO-*d*$_6$)** δ 6.00 (t, *J* = 7.6 Hz, 1H), 4.15 (d, *J* = 16.9 Hz, 2H), 3.90 (d, *J* = 16.9 Hz, 2H), 3.75 (q, *J* = 6.9 Hz, 6H), 2.82 (s, 3H), 2.46 (t, *J* = 7.1 Hz, 2H), 2.30 – 2.22 (m, 2H), 1.61 (tt, *J* = 7.2, 7.3 Hz, 2H), 1.50 (s, 2H), 1.14 (t, *J* = 7.0 Hz, 9H).

**$^{13}$C NMR (126 MHz, DMSO-*d*$_6$)** δ 168.8, 143.5, 131.0, 120.5, 61.4, 57.6, 45.3, 30.9, 25.3, 18.1, 15.8.

**ESI-MS**: calculated for $C_{18}H_{31}BN_2NaO_7Si^+$ [M+Na]$^+$, 449.1886; Found, 449.1887.

**(*Z*)-4-methyl-N-(6-(6-methyl-4,8-dioxo-1,3,6,2-dioxazaborocan-2-yl)-5-(triethoxysilyl)hex-4-en-1-yl)benzenesulfonamide (27)**

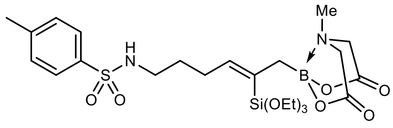


**27**

Following the **General procedure E,** the product **27** was obtained in 76% yield (43.3 mg, from **S-17**) as a colorless oil after column chromatography (eluent = ethyl acetate/petroleum ether v/v 4:1).

**$^1$H NMR (400 MHz, Acetone-*d*$_6$)** δ 7.84 – 7.79 (m, 2H), 7.50 – 7.41 (m, 2H), 6.02 (t, *J* = 7.8 Hz, 1H), 4.15 (d, *J* = 16.6 Hz, 2H), 3.93 (d, *J* = 16.6 Hz, 2H), 3.79 (q, *J* = 7.0 Hz, 6H), 3.20 – 3.14 (m, 2H), 3.06 (s, 3H), 2.44 (s, 3H), 2.35 – 2.28 (m, 2H), 1.75 – 1.65 (m, 2H), 1.63 (s, 2H), 1.14 (t, *J* = 7.0 Hz, 9H).

**$^{13}$C NMR (101 MHz, Acetone-*d*$_6$)** δ 168.7, 145.1, 144.8, 137.9, 132.5, 130.6, 128.9, 62.5, 58.8, 56.1, 46.0, 31.4, 23.8, 21.5, 18.5.

**ESI-MS**: calculated for $C_{24}H_{39}BN_2NaO_9SSi^+$ [M+Na]$^+$, 593.2131; Found, 593.2143.

**(*Z*)-2-(7-(1,1-dioxido-3-oxobenzo[d]isothiazol-2(3H)-yl)-2-(triethoxysilyl)hept-2-en-1-yl)-6-methyl-1,3,6,2-dioxazaborocane-4,8-dione (28)**

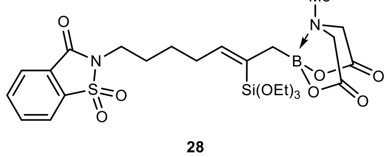


**28**

Following the **General procedure E,** the product **28** was obtained in 86% yield (51.2 mg, from **S-18**) as a colorless oil after column chromatography (eluent = ethyl acetate/petroleum ether v/v 4:1).

**$^1$H NMR (400 MHz, Acetone-*d*$_6$)** δ 8.20 – 7.97 (m, 4H), 6.18 (t, *J* = 7.6 Hz, 1H), 4.13

(d, $J$ = 16.7 Hz, 2H), 3.92 (d, $J$ = 16.6 Hz, 2H), 3.87 – 3.75 (m, 8H), 3.08 (s, 3H), 2.35 – 2.27 (m, 2H), 1.93 – 1.81 (m, 2H), 1.66 (s, 2H), 1.57 – 1.47 (m, 2H), 1.17 (t, $J$ = 7.0 Hz, 9H).

**$^{13}$C NMR (101 MHz, Acetone-*d*$_6$)** δ 168.7, 159.5, 146.7, 138.7, 136.2, 135.6, 130.7, 128.0, 125.8, 121.8, 62.6, 58.7, 46.0, 39.8, 32.7, 29.0, 27.8, 18.6.

**ESI-MS**: calculated for $C_{25}H_{37}BN_2NaO_{10}SSi^+$ [M+Na]$^+$, 619.1923; Found, 619.1920.

**(*Z*)-1,1,1-trifluoro-N-(5-(6-methyl-4,8-dioxo-1,3,6,2-dioxazaborocan-2-yl)-4-(triethoxysilyl)pent-3-en-1-yl)methanesulfonamide (29)**

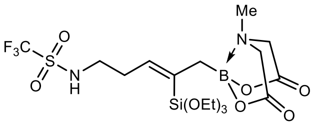


**29**

Following the **General procedure E,** the product **29** was obtained in 71% yield (37.9 mg, from **S-19**) as a colorless oil after column chromatography (eluent = ethyl acetate/petroleum ether v/v 4:1).

**$^1$H NMR (400 MHz, Acetone-*d*$_6$)** δ 6.13 (t, $J$ = 7.8 Hz, 1H), 4.16 (d, $J$ = 16.8 Hz, 2H), 3.96 (d, $J$ = 16.6 Hz, 2H), 3.87 (q, $J$ = 7.0 Hz, 6H), 3.34 (t, $J$ = 7.2 Hz, 2H), 3.09 (s, 3H), 2.61 – 2.56 (m, 2H), 1.70 (s, 2H), 1.20 (t, $J$ = 7.0 Hz, 9H).

**$^{13}$C NMR (101 MHz, Acetone-*d*$_6$)** δ 168.7, 141.5, 134.9, 121.0 (q, $J$ = 321.2 Hz), 62.6, 59.1, 46.1, 44.7, 33.5, 18.5.

**$^{19}$F NMR (376 MHz, Acetone-*d*$_6$)** δ -78.5.

**ESI-MS**: calculated for $C_{17}H_{30}BF_3N_2NaO_9SSi^+$ [M+Na]$^+$, 557.1379; Found, 557.1363.

**(*Z*)-2-(6-(1,3-dioxoisoindolin-2-yl)-2-(triethoxysilyl)hex-2-en-1-yl)-6-methyl-1,3,6,2-dioxazaborocane-4,8-dione (30)**

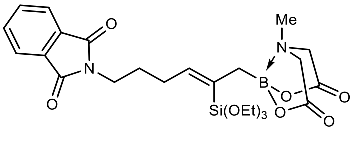


**30**

Following the **General procedure E,** the product **30** was obtained in 87% yield (47.5

mg, from **S-20**) as a colorless oil after column chromatography (eluent = ethyl acetate/petroleum ether v/v 4:1).

**$^{1}$H NMR (500 MHz, Acetone-*d*$_6$)** δ 7.88 – 7.80 (m, 4H), 6.18 (t, *J* = 7.7 Hz, 1H), 4.12 (d, *J* = 16.7 Hz, 2H), 3.92 (d, *J* = 16.7 Hz, 2H), 3.78 (q, *J* = 7.0 Hz, 6H), 3.68 (t, *J* = 7.1 Hz, 2H), 3.08 (s, 3H), 2.31 – 2.25 (m, 2H), 1.73 (tt, *J* = 7.2, 7.3 Hz, 2H), 1.66 (s, 2H), 1.14 (t, *J* = 7.0 Hz, 9H).

**$^{13}$C NMR (126 MHz, Acetone-*d*$_6$)** δ 168.7, 168.7, 145.7, 134.9, 133.2, 131.2, 123.6, 62.6, 58.7, 46.1, 38.3, 30.8, 29.6, 18.5.

**ESI-MS**: calculated for $C_{25}H_{35}BN_2NaO_9Si^+$ $[M+Na]^+$, 569.2097; Found, 569.2091.

**Methyl (*Z*)-6-(6-methyl-4,8-dioxo-1,3,6,2-dioxazaborocan-2-yl)-5-(triethoxysilyl)hex-4-enoate (31)**

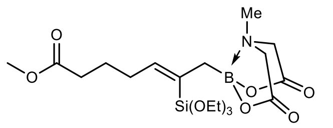


**31**

Following the **General procedure E,** the product **31** was obtained in 66% yield (30.3 mg, from **S-21**) as a colorless oil after column chromatography (eluent = ethyl acetate).

**$^{1}$H NMR (400 MHz, Acetone-*d*$_6$)** δ 6.14 (tt, *J* = 7.6, 1.3 Hz, 1H), 4.14 (d, *J* = 16.7 Hz, 2H), 3.93 (d, *J* = 16.7 Hz, 2H), 3.83 (q, *J* = 7.0 Hz, 6H), 3.61 (s, 3H), 3.08 (s, 3H), 2.37 – 2.22 (m, 4H), 1.76 – 1.62 (m, 4H), 1.19 (t, *J* = 7.0 Hz, 9H).

**$^{13}$C NMR (126 MHz, Acetone-*d*$_6$)** δ 174.1, 168.7, 146.1, 131.2, 62.5, 58.7, 51.5, 46.0, 34.0, 32.5, 25.9, 18.6.

**ESI-MS**: calculated for $C_{19}H_{34}BNNaO_9Si^+$ $[M+Na]^+$, 482.1988; Found, 482.1985.

**(*Z*)-2-(6-hydroxy-2-(triethoxysilyl)hex-2-en-1-yl)-6-methyl-1,3,6,2-dioxazaborocane-4,8-dione (32)**

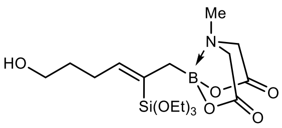


**32**

Following the **General procedure E,** the product **32** was obtained in 41% yield (17.1

mg, from **S-22**) as a colorless oil after column chromatography (eluent = ethyl acetate/acetonitrile v/v 8:1).

**$^1$H NMR (400 MHz, $CD_3CN$)** δ 6.11 (tt, *J* = 7.7, 1.3 Hz, 1H), 3.89 (d, *J* = 16.8 Hz, 2H), 3.80 (q, *J* = 7.0 Hz, 6H), 3.72 (d, *J* = 16.7 Hz, 2H), 3.51 – 3.45 (m, 2H), 2.84 (s, 3H), 2.69 (d, *J* = 5.4 Hz, 1H), 2.27 – 2.21 (m, 2H), 1.60 – 1.50 (m, 4H), 1.18 (t, *J* = 7.0 Hz, 9H).

**$^{13}$C NMR (101 MHz, $CD_3CN$)** δ 169.1, 147.2, 130.3, 62.6, 62.1, 59.0, 46.3, 33.6, 29.7, 18.5.

**ESI-MS**: calculated for $C_{17}H_{32}BNNaO_8Si^+$ $[M+Na]^+$, 440.1882; Found, 440.1877.

**(*Z*)-2-(4-hydroxy-2-(triethylsilyl)pent-2-en-1-yl)-6-methyl-1,3,6,2-dioxazaborocane-4,8-dione (33)**

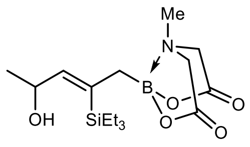


**33**

Following the **General procedure E,** the product **33** was obtained in 45% yield (16.0 mg, from **S-23**) as a colorless oil after column chromatography (eluent = ethyl acetate/acetonitrile v/v 8:1).

**$^1$H NMR (400 MHz, $CD_3CN$)** δ 5.95 (dt, *J* = 9.5, 1.2 Hz, 1H), 4.35 (dq, *J* = 9.6, 6.1 Hz, 1H), 3.91 (dd, *J* = 16.9, 1.2 Hz, 2H), 3.75 (dd, *J* = 17.0, 2.1 Hz, 2H), 2.84 (s, 3H), 2.52 (s, 1H), 1.52 (s, 2H), 1.13 (d, *J* = 6.1 Hz, 3H), 0.96 (t, *J* = 7.8 Hz, 9H), 0.76 – 0.65 (m, 6H).

**$^{13}$C NMR (101 MHz, $CD_3CN$)** δ 169.1, 169.0, 148.0, 135.7, 67.7, 62.6, 62.6, 46.5, 24.4, 8.1, 5.4.

**ESI-MS**: calculated for $C_{16}H_{30}BNNaO_5Si^+$ $[M+Na]^+$, 378.1879; Found, 378.1876.

**(*Z*)-2-(5-hydroxy-2-(triethylsilyl)pent-2-en-1-yl)-6-methyl-1,3,6,2-dioxazaborocane-4,8-dione (34)**

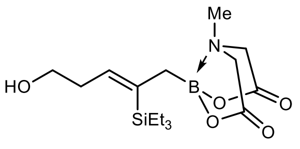

**34**

Following the **General procedure E,** the product **34** was obtained in 80% yield (28.4 mg, from **S-24**) as a colorless oil after column chromatography (eluent = ethyl acetate/acetonitrile v/v 8:1).

**$^1$H NMR (400 MHz, Acetone-*d*$_6$)** δ 6.08 (tt, *J* = 7.5, 1.2 Hz, 1H), 4.13 (d, *J* = 16.8 Hz, 2H), 3.99 (d, *J* = 16.7 Hz, 2H), 3.56 (t, *J* = 7.0 Hz, 2H), 3.09 (s, 3H), 2.39 – 2.33 (m, 2H), 1.62 (s, 2H), 0.96 (t, *J* = 7.8 Hz, 9H), 0.74 (q, *J* = 7.8 Hz, 6H).

**$^{13}$C NMR (101 MHz, Acetone-*d*$_6$)** δ 168.7, 140.2, 136.4, 62.8, 62.6, 46.2, 36.9, 8.1, 5.1.

**ESI-MS**: calculated for $C_{16}H_{30}BNNaO_5Si^+$ $[M+Na]^+$, 378.1879; Found, 378.1877.

**(*Z*)-2-(5-((tert-butyldiphenylsilyl)oxy)-2-(triethoxysilyl)pent-2-en-1-yl)-6-methyl-1,3,6,2-dioxazaborocane-4,8-dione (35)**

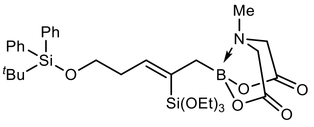

**35**

Following the **General procedure E,** the product **35** was obtained in 81% yield (51.9 mg, from **S-25**) as a colorless oil after column chromatography (eluent = ethyl acetate/petroleum ether v/v 2:1).

**$^1$H NMR (400 MHz, DMSO-*d*$_6$)** δ 7.66 – 7.59 (m, 4H), 7.49 – 7.37 (m, 6H), 6.11 (t, *J* = 7.5 Hz, 1H), 4.14 (d, *J* = 16.9 Hz, 2H), 3.88 (d, *J* = 16.9 Hz, 2H), 3.71 (q, *J* = 7.0 Hz, 6H), 3.65 (t, *J* = 6.9 Hz, 2H), 2.81 (s, 3H), 2.50 – 2.46 (m, 2H), 1.50 (s, 2H), 1.09 (t, *J* = 7.0 Hz, 9H), 0.98 (s, 9H).

**$^{13}$C NMR (126 MHz, DMSO-*d*$_6$)** δ 168.7, 141.2, 135.0, 133.3, 131.6, 129.8, 127.9, 63.7, 61.4, 57.6, 45.3, 35.4, 26.7, 18.8, 18.0.

**ESI-MS**: calculated for $C_{32}H_{48}BNNaO_8Si_2^+$ $[M+Na]^+$, 664.2904; Found, 664.2910.

**(*Z*)-2-(4-((tert-butyldiphenylsilyl)oxy)-2-(triethoxysilyl)pent-2-en-1-yl)-6-methyl-1,3,6,2-dioxazaborocane-4,8-dione (36)**

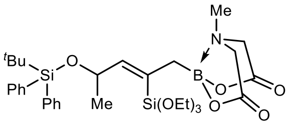


**36**

Following the **General procedure E,** the product **36** was obtained in 68% yield (43.6 mg, from **S-26**) as a colorless oil after column chromatography (eluent = ethyl acetate/petroleum ether v/v 2:1).

**$^1$H NMR (400 MHz, $CD_3CN$)** δ 7.76 – 7.72 (m, 4H), 7.47 – 7.39 (m, 6H), 6.35 (dt, *J* = 8.2, 1.3 Hz, 1H), 4.83 (dq, *J* = 8.2, 6.1 Hz, 1H), 3.95 (dd, *J* = 16.8, 1.4 Hz, 2H), 3.77 (d, *J* = 16.6 Hz, 2H), 3.65 – 3.50 (m, 6H), 2.88 (s, 3H), 1.58 (s, 2H), 1.16 (d, *J* = 6.1 Hz, 3H), 1.04 (s, 9H), 0.99 (t, *J* = 7.0 Hz, 9H).

**$^{13}$C NMR (101 MHz, $CD_3CN$)** δ 169.1, 169.0, 151.9, 136.8, 136.7, 135.5, 135.5, 130.6, 130.4, 128.6, 128.5, 70.8, 62.6, 58.9, 46.3, 27.4, 25.6, 19.7, 18.4.

**ESI-MS**: calculated for $C_{32}H_{48}BNNaO_8Si_2^+$ $[M+Na]^+$, 664.2904; Found, 664.2913.

**(*Z*)-5-(6-methyl-4,8-dioxo-1,3,6,2-dioxazaborocan-2-yl)-4-(triethoxysilyl)pent-3-en-1-yl benzoate (37)**

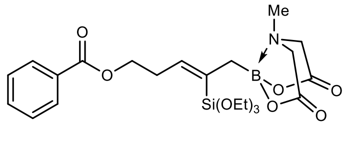


**37**

Following the **General procedure E,** the product **37** was obtained in 81% yield (41.1 mg, from **S-27**) as a colorless oil after column chromatography (eluent = ethyl acetate/petroleum ether v/v 4:1).

**$^1$H NMR (400 MHz, DMSO-*d*$_6$)** δ 8.01 – 7.94 (m, 2H), 7.69 – 7.60 (m, 1H), 7.51 (t, *J* = 7.7 Hz, 2H), 6.15 (t, *J* = 7.5 Hz, 1H), 4.32 – 4.24 (m, 2H), 4.15 (d, *J* = 16.9 Hz, 2H), 3.90 (d, *J* = 17.0 Hz, 2H), 3.75 (q, *J* = 7.0 Hz, 6H), 2.83 (s, 3H), 2.66 – 2.60 (m, 2H), 1.55 (s, 2H), 1.12 (t, *J* = 7.0 Hz, 9H).

**$^{13}$C NMR (126 MHz, DMSO-*d*$_6$)** δ 168.8, 165.8, 140.5, 133.2, 132.7, 129.9, 129.2, 129.2, 128.7, 64.4, 61.4, 57.7, 45.3, 31.4, 18.0.

**ESI-MS**: calculated for $C_{23}H_{34}BNNaO_9Si^+$ $[M+Na]^+$, 530.1988; Found, 530.1977.

**(*Z*)-5-(6-methyl-4,8-dioxo-1,3,6,2-dioxazaborocan-2-yl)-4-(triethoxysilyl)pent-3-en-1-yl 4-methylbenzenesulfonate (38)**

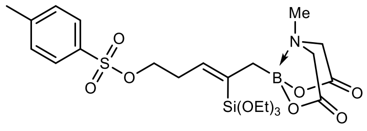


**38**

Following the **General procedure E,** the product **38** was obtained in 74% yield (41.2 mg, from **S-28**) as a colorless oil after column chromatography (eluent = ethyl acetate/petroleum ether v/v 4:1).

**$^1$H NMR (500 MHz, Acetone-*d*$_6$)** δ 7.78 (d, *J* = 8.0 Hz, 2H), 7.45 (d, *J* = 7.9 Hz, 2H), 5.99 (t, *J* = 7.6 Hz, 1H), 4.11 (d, *J* = 16.8 Hz, 2H), 4.00 (t, *J* = 7.0 Hz, 2H), 3.90 (d, *J* = 16.7 Hz, 2H), 3.75 (q, *J* = 7.0 Hz, 6H), 3.03 (s, 3H), 2.58 – 2.53 (m, 2H), 2.42 (s, 3H), 1.62 (s, 2H), 1.11 (t, *J* = 6.9 Hz, 9H).

**$^{13}$C NMR (126 MHz, Acetone-*d*$_6$)** δ 168.7, 145.7, 139.8, 135.3, 134.3, 130.9, 128.7, 71.0, 62.5, 58.8, 46.0, 32.6, 21.5, 18.5.

**ESI-MS**: calculated for $C_{23}H_{36}BNNaO_{10}SSi^+$ $[M+Na]^+$, 580.1814; Found, 580.1820.

**(*Z*)-2-(4-(benzyloxy)-2-(triethoxysilyl)but-2-en-1-yl)-6-methyl-1,3,6,2-dioxazaborocane-4,8-dione (39)**

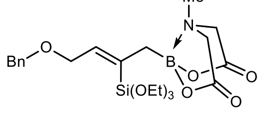


**39**

Following the **General procedure E,** the product **39** was obtained in 82% yield (39.3 mg, from **S-29**) as a colorless oil after column chromatography (eluent = ethyl acetate/petroleum ether v/v 4:1).

**$^1$H NMR (400 MHz, $CD_3CN$)** δ 7.40 – 7.25 (m, 5H), 6.26 (tt, *J* = 6.8, 1.3 Hz, 1H), 4.49 (s, 2H), 4.15 (d, *J* = 6.7 Hz, 2H), 3.91 (d, *J* = 16.8 Hz, 2H), 3.83 – 3.70 (m, 8H), 2.85 (s, 3H), 1.65 (s, 2H), 1.16 (t, *J* = 7.0 Hz, 9H).

**$^{13}$C NMR (101 MHz, $CD_3CN$)** δ 169.0, 143.3, 140.0, 134.3, 129.3, 128.8, 128.4, 73.0,

70.9, 62.6, 59.1, 46.3, 18.5.

**ESI-MS**: calculated for $C_{22}H_{34}BNNaO_8Si^+$ $[M+Na]^+$, 502.2039; Found, 502.2045.

**6-methyl-2-(4-methyl-2-(triethoxysilyl)pent-2-en-1-yl)-1,3,6,2-dioxazaborocane-4,8-dione (40)**

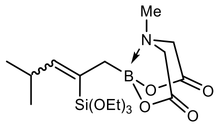


**40**

Following the **General procedure E,** the product **40** was obtained in 55% yield (22.1 mg, from **S-30**) as a yellow oil after column chromatography (mixture of *Z* & *E* isomers, *Z/E* = 1:1.2, eluent = ethyl acetate/petroleum ether v/v 4:1).

**$^1$H NMR (400 MHz, DMSO-*d*$_6$)** (*Z* isomer) δ 5.84 (d, *J* = 10.2 Hz, 1H), 4.14 (d, *J* = 16.9 Hz, 2H), 3.86 (d, *J* = 16.8 Hz, 2H), 3.75 (q, *J* = 7.0 Hz, 6H), 2.82 (s, 3H), 2.67 – 2.57 (m, 1H), 1.46 (s, 2H), 1.14 (t, *J* = 7.0 Hz, 9H), 0.90 (d, *J* = 6.5 Hz, 6H). (*E* isomer) δ 5.66 (d, *J* = 9.5 Hz, 1H), 4.14 (d, *J* = 16.9 Hz, 2H), 3.72 (q, *J* = 7.0 Hz, 6H), 2.88 (s, 3H), 2.75 – 2.68 (m, 1H), 1.54 (s, 2H), 1.13 (t, *J* = 7.0 Hz, 9H), 0.91 (d, *J* = 6.6 Hz, 6H).

**ESI-MS**: calculated for $C_{17}H_{32}BNNaO_7Si^+$ $[M+Na]^+$, 424.1933; Found, 424.1938.

**6-methyl-2-(2-(triethoxysilyl)allyl)-1,3,6,2-dioxazaborocane-4,8-dione (41)**

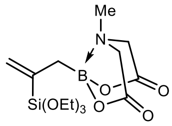


**41**

Following the **General procedure E,** the product **41** was obtained in 42% yield (15.1 mg, from **S-31**) as a colorless oil after column chromatography (eluent = ethyl acetate/petroleum ether v/v 2:1).

**$^1$H NMR (400 MHz, Acetone-*d*$_6$)** δ 5.81 (dt, *J* = 3.2, 1.5 Hz, 1H), 5.60 (dt, *J* = 3.5, 1.0 Hz, 1H), 4.15 (d, *J* = 16.7 Hz, 2H), 3.96 (d, *J* = 16.6 Hz, 2H), 3.83 (q, *J* = 7.0 Hz, 6H), 3.13 (s, 3H), 1.71 (s, 2H), 1.19 (t, *J* = 7.0 Hz, 9H).

**$^{13}$C NMR (101 MHz, Acetone-*d*$_6$)** δ 168.7, 142.1, 130.4, 62.8, 59.1, 46.3, 18.6.

**ESI-MS**: calculated for $C_{14}H_{26}BNNaO_7Si^+$ $[M+Na]^+$, 382.1464; Found, 382.1466.

**6-methyl-2-(2-(triethoxysilyl)oct-1-en-3-yl)-1,3,6,2-dioxazaborocane-4,8-dione (42)**

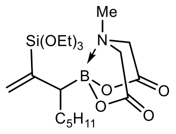


**42**

Following the **General procedure E,** the product **42** was obtained in 46% yield (19.7 mg, from **S-32**) as a colorless oil after column chromatography (eluent = ethyl acetate/petroleum ether v/v 2:1).

**$^1$H NMR (400 MHz, $CD_3CN$)** δ 5.81 (dd, $J$ = 2.9, 0.7 Hz, 1H), 5.67 (d, $J$ = 2.9 Hz, 1H), 3.93 – 3.72 (m, 9H), 3.65 (d, $J$ = 16.5 Hz, 1H), 2.92 (s, 3H), 1.86 (dd, $J$ = 10.9, 3.5 Hz, 1H), 1.64 – 1.45 (m, 2H), 1.40 – 1.24 (m, 6H), 1.18 (t, $J$ = 7.0 Hz, 9H), 0.87 (t, $J$ = 7.1 Hz, 3H).

**$^{13}$C NMR (101 MHz, $CD_3CN$)** δ 169.4, 168.9, 148.0, 130.1, 63.4, 63.4, 59.4, 46.7, 32.8, 32.6, 29.2, 23.4, 18.5, 14.4.

**ESI-MS**: calculated for $C_{19}H_{36}BNNaO_7Si^+$ $[M+Na]^+$, 452.2246; Found, 452.2236.

**6-methyl-2-(3-(triethoxysilyl)oct-3-en-2-yl)-1,3,6,2-dioxazaborocane-4,8-dione (43)**

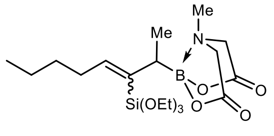


**43**

Following the **General procedure E,** the product **43** was obtained in 83% yield (35.6 mg, from **S-33**) as a yellow oil after column chromatography (mixture of *Z* & *E* isomers, *Z/E* = 4.5:1, eluent = ethyl acetate/petroleum ether v/v 2:1).

**$^1$H NMR (400 MHz, Acetone-$d_6$)** δ *Z* isomer (major) 6.29 (t, $J$ = 7.6 Hz, 1H), 4.16 – 4.09 (m, 2H), 3.97 -3.91 (m, 1H) , 3.84 (q, $J$ = 7.0 Hz, 6H), 3.75 (d, $J$ = 16.4 Hz, 1H), 3.12 (s, 3H), 2.29 – 2.22 (m, 2H), 2.01 (q, $J$ = 7.4 Hz, 1H), 1.38 – 1.32 (m, 4H), 1.19 (t, $J$ = 6.9 Hz, 9H), 1.08 (d, $J$ = 7.4 Hz, 3H), 0.92 – 0.88 (m, 3H). *E* isomer (minor) 5.93 (t, $J$ = 6.8 Hz, 1H), 1.18 (t, $J$ = 7.0 Hz, 9H), 1.15 (d, $J$ = 7.4 Hz, 3H). (overlap) 4.16 – 4.09 (m, 2H), 3.97 -3.91 (m, 1H), 3.84 (q, $J$ = 7.0 Hz, 6H), 3.75 (d, $J$ = 16.4 Hz, 1H),

3.12 (s, 3H), 2.29 – 2.22 (m, 2H), 2.01 (q, $J$ = 7.4 Hz, 1H), 1.38 – 1.32 (m, 4H), 0.92 – 0.88 (m, 3H).

**$^{13}$C NMR (101 MHz, Acetone-$d_6$)** δ 169.1, 168.4, 147.0, 145.8, 137.6, 63.3, 63.2, 59.0, 58.8, 46.7, 46.4, 33.2, 32.8, 32.4, 23.3, 18.8, 18.6, 18.5, 17.2, 14.3, 14.3.(mixture of *Z* & *E* isomers)

**ESI-MS**: calculated for $C_{19}H_{36}BNNaO_7Si^+$ $[M+Na]^+$, 452.2246; Found, 452.2238.

**(*Z*)-6-methyl-2-(2-(triethoxysilyl)penta-2,4-dien-1-yl)-1,3,6,2-dioxazaborocane-4,8-dione (44)**

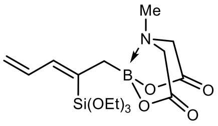


**44**

Following the **General procedure E,** the product **44** was obtained in 76% yield (29.3 mg, from **S-37**) as a colorless oil after column chromatography (eluent = ethyl acetate/petroleum ether v/v 2:1).

**$^1$H NMR (500 MHz, Acetone-$d_6$)** δ 6.87 – 6.79 (m, 1H), 6.72 (d, $J$ = 11.2 Hz, 1H), 5.15 – 5.03 (m, 2H), 4.16 (d, $J$ = 16.7 Hz, 2H), 3.96 (d, $J$ = 16.7 Hz, 2H), 3.82 (q, $J$ = 7.0 Hz, 6H), 3.10 (s, 3H), 1.77 (s, 2H), 1.17 (t, $J$ = 7.0 Hz, 9H).

**$^{13}$C NMR (126 MHz, Acetone-$d_6$)** δ 168.6, 145.7, 138.1, 136.4, 117.0, 62.5, 58.8, 46.0, 18.5.

**ESI-MS**: calculated for $C_{16}H_{28}BNNaO_7Si^+$ $[M+Na]^+$, 408.1620; Found, 408.1615.

**(*E*)-6-methyl-2-(2-(triethylsilyl)hept-2-en-1-yl)-1,3,6,2-dioxazaborocane-4,8-dione (46)**

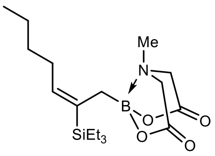


**46**

Following the **General procedure F**, the product **46** was obtained in 91% yield (33.5 mg, from **S-1**) as a colorless oil after column chromatography (eluent = ethyl

acetate/petroleum ether v/v 4:1).

**$^{1}$H NMR (500 MHz, DMSO-*d*$_6$)** δ 5.55 (t, *J* = 6.6 Hz, 1H), 4.13 (d, *J* = 16.9 Hz, 2H), 4.00 (d, *J* = 16.9 Hz, 2H), 2.84 (s, 3H), 2.12 – 2.05 (m, 2H), 1.49 (s, 2H), 1.34 – 1.27 (m, 4H), 0.91 – 0.83 (m, 12H), 0.57 (q, *J* = 7.9 Hz, 6H).

**$^{13}$C NMR (126 MHz, DMSO-*d*$_6$)** δ 168.9, 140.1, 133.7, 61.3, 45.3, 31.4, 28.1, 22.0, 14.0, 7.5, 2.7.

**ESI-MS**: calculated for $C_{18}H_{34}BNNaO_4Si^+$ [M+Na]$^+$, 390.2242; Found, 390.2249.

**(*E*)-2-(2-(benzyldimethylsilyl)hept-2-en-1-yl)-6-methyl-1,3,6,2-dioxazaborocane-4,8-dione (47)**

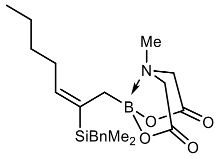


**47**

Following the **General procedure F**, the product **47** was obtained in 94% yield (37.8 mg, from **S-1**) as a colorless oil after column chromatography (eluent = ethyl acetate/petroleum ether v/v 4:1).

**$^{1}$H NMR (500 MHz, CD$_3$CN)** δ 7.23 – 7.12 (m, 2H), 7.11 – 6.97 (m, 3H), 5.64 (t, *J* = 6.6 Hz, 1H), 3.94 (d, *J* = 16.9 Hz, 2H), 3.81 (d, *J* = 16.9 Hz, 2H), 2.86 (s, 3H), 2.20 (s, 2H), 2.11 – 2.06 (m, 2H), 1.60 (s, 2H), 1.38 – 1.25 (m, 4H), 0.90 (t, *J* = 6.9 Hz, 3H), 0.00 (s, 6H).

**$^{13}$C NMR (126 MHz, CD$_3$CN)** δ 169.1, 142.0, 142.0, 136.5, 129.3, 128.8, 124.6, 62.5, 46.4, 32.3, 29.5, 26.4, 23.2, 14.4, -2.7.

**ESI-MS**: calculated for $C_{21}H_{32}BNNaO_4Si^+$ [M+Na]$^+$, 424.2086; Found, 424.2099.

**(*E*)-2-(2-(dimethyl(phenyl)silyl)hept-2-en-1-yl)-6-methyl-1,3,6,2-dioxazaborocane-4,8-dione (48)**

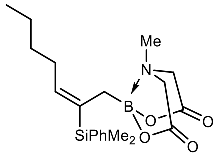


**48**

Following the **General procedure F**, the product **48** was obtained in 76% yield (29.4 mg, from **S-1**) as a colorless oil after column chromatography (eluent = ethyl acetate/petroleum ether v/v 4:1).

**$^1$H NMR (500 MHz, DMSO-*d*$_6$)** δ 7.53 – 7.47 (m, 2H), 7.35 – 7.29 (m, 3H), 5.61 (t, *J* = 6.6 Hz, 1H), 4.15 (d, *J* = 17.1 Hz, 2H), 3.98 (d, *J* = 16.9 Hz, 2H), 2.81 (s, 3H), 2.08 (q, *J* = 6.8 Hz, 2H), 1.54 (s, 2H), 1.32 – 1.22 (m, 4H), 0.85 (t, *J* = 6.8 Hz, 3H), 0.30 (s, 6H).

**$^{13}$C NMR (126 MHz, DMSO-*d*$_6$)** δ 168.8, 141.2, 139.8, 135.6, 133.9, 128.5, 127.5, 61.3, 45.3, 31.2, 28.3, 22.0, 14.0, -2.0.

**ESI-MS**: calculated for $C_{20}H_{30}BNNaO_4Si^+$ [M+Na]$^+$, 410.1929; Found, 410.1927.

**(*E*)-2-(2-(1,1,1,3,5,5,5-heptamethyltrisiloxan-3-yl)hept-2-en-1-yl)-6-methyl-1,3,6,2-dioxazaborocane-4,8-dione (49)**

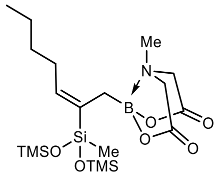


**49**

Following the **General procedure F,** the product **49** was obtained in 86% yield (40.7 mg, from **S-1**) as a waxy solid after column chromatography (eluent = ethyl acetate/petroleum ether v/v 2:1).

**$^1$H NMR (400 MHz, Acetone-*d*$_6$)** δ 5.87 (tt, *J* = 6.7, 1.3 Hz, 1H), 4.15 (d, *J* = 16.8 Hz, 2H), 4.01 (d, *J* = 16.7 Hz, 2H), 3.12 (s, 3H), 2.16 – 2.11 (m, 2H), 1.69 (s, 2H), 1.43 – 1.29 (m, 4H), 0.90 (t, *J* = 7.2 Hz, 3H), 0.16 (s, 3H), 0.12 (s, 18H).

**$^{13}$C NMR (101 MHz, Acetone-*d*$_6$)** δ 168.6, 142.0, 137.1, 62.4, 46.0, 32.3, 23.2, 14.4, 2.1, 0.8.

**ESI-MS**: calculated for $C_{19}H_{40}BNNaO_6Si_3^+$ [M+Na]$^+$, 496.2149; Found, 496.2146.

**(*E*)-6-methyl-2-(2-(triisopropoxysilyl)hept-2-en-1-yl)-1,3,6,2-dioxazaborocane-4,8-dione (50)**

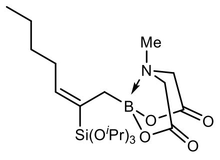

50

Following the **General procedure F**, the product **50** was obtained in 76% yield (34.7mg, from **S-1**) as a colorless oil after column chromatography (eluent = ethyl acetate/petroleum ether v/v 2:1).

**$^1$H NMR (400 MHz, $CD_3CN$)** δ 5.98 (t, *J* = 6.8 Hz, 1H), 4.27 – 4.18 (m, 3H), 3.88 (d, *J* = 16.8 Hz, 2H), 3.79 (d, *J* = 16.6 Hz, 2H), 2.93 (s, 3H), 2.16 – 2.114 (m, 2H), 1.65 (s, 2H), 1.42 – 1.31 (m, 4H), 1.16 (d, *J* = 6.1 Hz, 18H), 0.90 (t, *J* = 7.0 Hz, 3H).

**$^{13}$C NMR (101 MHz, $CD_3CN$)** δ 169.0, 145.9, 131.7, 65.9, 62.5, 46.3, 32.1, 29.6, 25.9, 25.8, 25.8, 23.2, 14.3.

**ESI-MS**: calculated for $C_{21}H_{40}BNNaO_7Si^+$ $[M+Na]^+$, 480.2559; Found, 480.2562.

**(*E*)-6-methyl-2-(2-(triethoxysilyl)hex-2-en-1-yl)-1,3,6,2-dioxazaborocane-4,8-dione (51)**

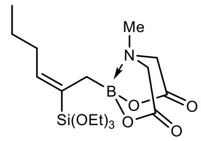

51

Following the **General procedure F,** the product **51** was obtained in 81% yield (32.5 mg, from **S-2**) as a colorless oil after column chromatography (eluent = ethyl acetate/petroleum ether v/v 4:1).

**$^1$H NMR (400 MHz, Acetone-*d*$_6$)** δ 6.01 (t, *J* = 6.8 Hz, 1H), 4.11 (d, *J* = 16.6 Hz, 2H), 3.99 (d, *J* = 16.5 Hz, 2H), 3.81 (q, *J* = 7.0 Hz, 6H), 3.16 (s, 3H), 2.20 – 2.13 (m, 2H), 1.73 (s, 2H), 1.47 – 1.39 (m, 2H), 1.18 (t, *J* = 7.0 Hz, 9H), 0.92 (t, *J* = 7.4 Hz, 3H).

**$^{13}$C NMR (101 MHz, Acetone-*d*$_6$)** δ 168.6, 145.8, 130.6, 62.5, 58.9, 45.9, 31.8, 23.0, 18.5, 14.2.

**ESI-MS**: calculated for $C_{17}H_{32}BNNaO_7Si^+$ $[M+Na]^+$, 424.1933; Found, 424.1928.

**(*E*)-6-methyl-2-(2-(triethoxysilyl)but-2-en-1-yl)-1,3,6,2-dioxazaborocane-4,8-**

**dione (52)**

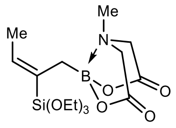


**52**

Following the **General procedure F,** the product **52** was obtained in 87% yield (32.5 mg, from **S-3**) as a colorless oil after column chromatography (eluent = ethyl acetate/petroleum ether v/v 4:1).

**$^1$H NMR (500 MHz, Acetone-*d*$_6$)** δ 6.14 (q, $J$ = 6.6 Hz, 1H), 4.12 (d, $J$ = 16.6 Hz, 2H), 4.00 (d, $J$ = 16.5 Hz, 2H), 3.81 (q, $J$ = 6.9 Hz, 6H), 3.16 (s, 3H), 1.75 – 1.70 (m, 5H), 1.18 (t, $J$ = 7.0 Hz, 9H).

**$^{13}$C NMR (126 MHz, Acetone-*d*$_6$)** δ 168.7, 139.7, 132.0, 62.5, 58.9, 45.9, 18.5, 15.3.

**ESI-MS**: calculated for $C_{15}H_{28}BNNaO_7Si^+$ [M+Na]$^+$, 396.1620; Found, 396.1616.

**(*E*)-2-(6-chloro-2-(1,1,1,3,5,5,5-heptamethyltrisiloxan-3-yl)hex-2-en-1-yl)-6-methyl-1,3,6,2-dioxazaborocane-4,8-dione (53)**

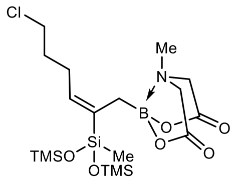


**53**

Following the **General procedure F,** the product **53** was obtained in 77% yield (38.0 mg, from **S-4**) as a colorless oil after column chromatography (eluent = ethyl acetate/petroleum ether v/v 2:1).

**$^1$H NMR (400 MHz, Acetone-*d*$_6$)** δ 5.85 (tt, $J$ = 7.0, 1.3 Hz, 1H), 4.18 (d, $J$ = 16.8 Hz, 2H), 4.03 (d, $J$ = 16.7 Hz, 2H), 3.61 (t, $J$ = 6.9 Hz, 2H), 3.13 (s, 3H), 2.29 – 2.23 (m, 2H), 1.94 – 1.82 (m, 2H), 1.72 (s, 2H), 0.17 (s, 3H), 0.12 (s, 18H).

**$^{13}$C NMR (101 MHz, Acetone-*d*$_6$)** δ 168.6, 139.7, 138.8, 62.4, 46.1, 45.5, 33.2, 27.0, 2.1, 0.7.

**ESI-MS**: calculated for $C_{18}H_{37}BClNNaO_6Si_3^+$ [M+Na]$^+$, 516.1602; Found, 516.1607.

**(*E*)-6-methyl-2-(6-phenyl-2-(triethoxysilyl)hex-2-en-1-yl)-1,3,6,2-**

**dioxazaborocane-4,8-dione (54)**

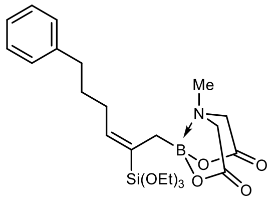

**54**

Following the **General procedure F,** the product **54** was obtained in 73% yield (34.8 mg, from **S-7**) as a colorless oil after column chromatography (eluent = ethyl acetate/petroleum ether v/v 4:1).

**$^{1}$H NMR (500 MHz, $CD_3CN$)** δ 7.31 – 7.14 (m, 5H), 6.03 (t, *J* = 6.9 Hz, 1H), 3.89 (d, *J* = 16.8 Hz, 2H), 3.82 – 3.76 (m, 8H), 2.89 (s, 3H), 2.62 (dd, *J* = 8.9, 6.8 Hz, 2H), 2.19 – 2.15 (m, 2H), 1.70 (tt, *J* = 7.5, 7.6 Hz, 2H), 1.63 (s, 2H), 1.17 (t, *J* = 6.9 Hz, 9H).

**$^{13}$C NMR (126 MHz, $CD_3CN$)** δ 169.1, 145.7, 143.8, 130.8, 129.4, 129.3, 126.6, 62.6, 59.2, 46.2, 36.2, 31.8, 29.5, 18.5.

**$^{11}$B NMR (128 MHz, Acetone-*d*$_6$)** δ 12.30.

**ESI-MS**: calculated for $C_{23}H_{26}BNNaO_7Si^+$ $[M+Na]^+$, 500.2246; Found, 500.2243.

**(*E*)-6-methyl-2-(2-(triethoxysilyl)-5-(3-(trifluoromethyl)phenyl)pent-2-en-1-yl)-1,3,6,2-dioxazaborocane-4,8-dione (55)**

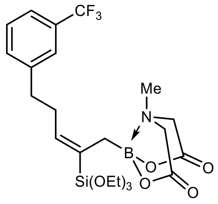

**55**

Following the **General procedure F,** the product **55** was obtained in 53% yield (28.1 mg, from **S-9**) as a colorless oil after column chromatography (eluent = ethyl acetate/petroleum ether v/v 4:1).

**$^{1}$H NMR (400 MHz, $CD_3CN$)** δ 7.61 – 7.41 (m, 4H), 5.96 (tt, *J* = 6.8, 1.3 Hz, 1H), 3.90 (d, *J* = 16.8 Hz, 2H), 3.80 (d, *J* = 16.7 Hz, 2H), 3.72 (q, *J* = 7.0 Hz, 6H), 2.91 (s, 3H), 2.82 (t, *J* = 7.6 Hz, 2H), 2.52 – 2.47 (m, 2H), 1.61 (s, 2H), 1.13 (t, *J* = 7.0 Hz, 9H).

**$^{13}$C NMR (101 MHz, $CD_3CN$)** δ 169.1, 144.7, 144.4, 133.5, 131.6, 130.8 (q, *J* = 31.5

Hz), 126.1 (q, $J$ = 3.9 Hz), 125.6 (d, $J$ = 271.8 Hz), 123.5 (q, $J$ = 4.1 Hz), 62.6, 59.1, 46.3, 35.4, 31.3, 18.5.

**$^{19}$F NMR (376 MHz, $CD_3CN$)** δ -62.92.

**ESI-MS**: calculated for $C_{23}H_{34}BF_3NO_7Si^+$ $[M+H]^+$, 532.2144; Found, 532.2160.

**(*E*)-2-(6-(4-methoxyphenoxy)-2-(triethoxysilyl)hex-2-en-1-yl)-6-methyl-1,3,6,2-dioxazaborocane-4,8-dione (56)**

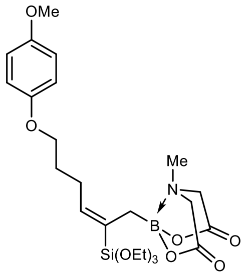


**56**

Following the **General procedure F,** the product **56** was obtained in 70% yield (36.6 mg, from **S-10**) as a colorless oil after column chromatography (eluent = ethyl acetate/petroleum ether v/v 4:1).

**$^1$H NMR (400 MHz, Acetone-$d_6$)** δ 6.94 – 6.77 (m, 4H), 6.06 (t, $J$ = 6.9 Hz, 1H), 4.13 (d, $J$ = 16.6 Hz, 2H), 4.00 (d, $J$ = 16.6 Hz, 2H), 3.95 (t, $J$ = 6.7 Hz, 2H), 3.81 (q, $J$ = 7.0 Hz, 6H), 3.72 (s, 3H), 3.15 (s, 3H), 2.39 – 2.33 (m, 2H), 1.87 (tt, $J$ = 6.9, 7.0 Hz, 2H), 1.75 (s, 2H), 1.18 (t, $J$ = 7.0 Hz, 9H).

**$^{13}$C NMR (101 MHz, Acetone-$d_6$)** δ 168.7, 154.7, 154.2, 144.9, 131.4, 116.2, 115.3, 68.6, 62.5, 58.9, 55.8, 45.9, 29.6, 26.2, 18.5.

**ESI-MS**: calculated for $C_{24}H_{38}BNNaO_9Si^+$ $[M+Na]^+$, 546.2301; Found, 546.2297.

**(*E*)-6-methyl-2-(5-(5-methylfuran-2-yl)-2-(triethoxysilyl)pent-2-en-1-yl)-1,3,6,2-dioxazaborocane-4,8-dione (57)**

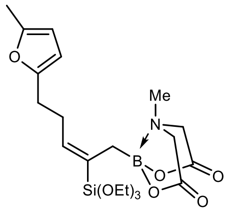


**57**

Following the **General procedure F,** the product **57** was obtained in 75% yield (35.0 mg, from **S-12**) as a colorless oil after column chromatography (eluent = ethyl acetate/petroleum ether v/v 4:1).

**$^{1}$H NMR (500 MHz, $CD_3CN$)** δ 5.98 (t, *J* = 6.6 Hz, 1H), 5.91 (d, *J* = 3.0 Hz, 1H), 5.86 (d, *J* = 4.0 Hz, 1H), 3.90 (d, *J* = 16.8 Hz, 2H), 3.80 (d, *J* = 16.8 Hz, 2H), 3.75 (q, *J* = 7.0 Hz, 6H), 2.92 (s, 3H), 2.66 (t, *J* = 7.5 Hz, 2H), 2.46 – 2.41 (m, 2H), 2.21 (s, 3H), 1.65 (s, 2H), 1.15 (t, *J* = 7.0 Hz, 9H).

**$^{13}$C NMR (126 MHz, $CD_3CN$)** δ 169.1, 155.1, 151.1, 144.7, 131.4, 106.9, 106.7, 62.6, 59.2, 46.2, 28.4, 28.0, 18.5, 13.5.

**ESI-MS**: calculated for $C_{21}H_{34}BNNaO_8Si^+$ $[M+Na]^+$, 490.2039; Found, 490.2025.

**(*E*)-2-(4-cyclohexyl-2-(triethoxysilyl)but-2-en-1-yl)-6-methyl-1,3,6,2-dioxazaborocane-4,8-dione (58)**

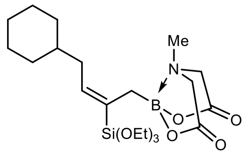


**58**

Following the **General procedure F,** the product **58** was obtained in 78% yield (35.5 mg, from **S-14**) as a colorless oil after column chromatography (eluent = ethyl acetate/petroleum ether v/v 4:1).

**$^{1}$H NMR (500 MHz, Acetone-*d*$_6$)** δ 6.05 (t, *J* = 6.7 Hz, 1H), 4.11 (d, *J* = 16.6 Hz, 2H), 3.99 (d, *J* = 16.6 Hz, 2H), 3.81 (q, *J* = 7.0 Hz, 6H), 3.15 (s, 3H), 2.10 (t, *J* = 6.8 Hz, 2H), 1.80 – 1.60 (m, 7H), 1.29 – 1.22 (m, 4H), 1.18 (t, *J* = 7.0 Hz, 9H), 0.97 (qd, *J* = 12.2, 3.3 Hz, 2H).

**$^{13}$C NMR (126 MHz, Acetone-*d*$_6$)** δ 168.6, 144.6, 131.1, 62.5, 58.9, 45.9, 39.0, 37.5, 34.0, 27.3, 27.1, 18.5.

**ESI-MS**: calculated for $C_{21}H_{38}BNNaO_7Si^+$ $[M+Na]^+$, 478.2403; Found, 478.2404.

**(*E*)-6-methyl-2-(5,7,7-trimethyl-2-(triethoxysilyl)oct-2-en-1-yl)-1,3,6,2-dioxazaborocane-4,8-dione (59)**

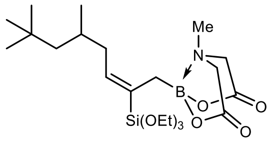


**59**

Following the **General procedure F,** the product **59** was obtained in 60% yield (28.3 mg, from **S-15**) as a colorless oil after column chromatography (eluent = ethyl acetate/petroleum ether v/v 4:1).

**$^1$H NMR (500 MHz, Acetone-*d*$_6$)** δ 6.05 (t, *J* = 6.6 Hz, 1H), 4.11 (d, *J* = 16.6 Hz, 2H), 3.99 (d, *J* = 16.6 Hz, 2H), 3.82 (q, *J* = 7.0 Hz, 6H), 3.16 (s, 3H), 2.21 – 2.07 (m, 2H), 1.73 (s, 2H), 1.70 – 1.63 (m, 1H), 1.34 (dd, *J* = 13.9, 3.5 Hz, 1H), 1.07 (dd, *J* = 14.0, 6.8 Hz, 1H), 0.96 (d, *J* = 6.6 Hz, 3H), 0.92 (s, 9H).

**$^{13}$C NMR (126 MHz, Acetone-*d*$_6$)** δ 168.6, 145.0, 131.3, 62.5, 58.9, 51.4, 45.9, 39.3, 31.6, 30.4, 30.3, 23.1, 18.6.

**ESI-MS**: calculated for $C_{22}H_{42}BNNaO_7Si^+$ [M+Na]$^+$, 494.2716; Found, 494.2703.

**(*E*)-6-(1,1,1,3,5,5,5-heptamethyltrisiloxan-3-yl)-7-(6-methyl-4,8-dioxo-1,3,6,2-dioxazaborocan-2-yl)hept-5-enenitrile (60)**

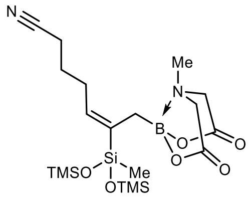


**60**

Following the **General procedure F,** the product **60** was obtained in 72% yield (34.5 mg, from **S-16**) as a colorless oil after column chromatography (eluent = ethyl acetate/petroleum ether v/v 2:1).

**$^1$H NMR (400 MHz, Acetone-*d*$_6$)** δ 5.84 (tt, *J* = 7.0, 1.3 Hz, 1H), 4.19 (d, *J* = 16.8 Hz, 2H), 4.03 (d, *J* = 16.8 Hz, 2H), 3.13 (s, 2H), 2.45 (t, *J* = 7.3 Hz, 2H), 2.28 – 2.23 (m, 2H), 1.82 – 1.69 (m, 4H), 0.17 (s, 3H), 0.12 (s, 18H).

**$^{13}$C NMR (101 MHz, Acetone-*d*$_6$)** δ 168.6, 139.4, 139.2, 120.8, 62.4, 46.1, 28.7, 26.0, 16.9, 2.1, 0.6.

**ESI-MS**: calculated for $C_{19}H_{37}BN_2NaO_6Si_3^+$ [M+Na]$^+$, 507.1945; Found, 507.1941.

**(*E*)-1,1,1-trifluoro-N-(5-(6-methyl-4,8-dioxo-1,3,6,2-dioxazaborocan-2-yl)-4-(triethoxysilyl)pent-3-en-1-yl)methanesulfonamide (61)**

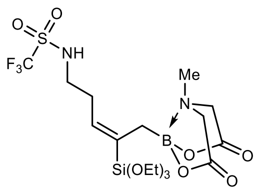


**61**

Following the **General procedure F,** the product **61** was obtained in 60% yield (32.0 mg, from **S-19**) as a colorless oil after column chromatography (eluent = ethyl acetate/petroleum ether v/v 2:1).

**$^{1}$H NMR (400 MHz, Acetone-*d*$_6$)** δ 7.93 (t, *J* = 5.8 Hz, 1H), 6.03 (tt, *J* = 6.7, 1.3 Hz, 1H), 4.16 (d, *J* = 16.7 Hz, 2H), 4.02 (d, *J* = 16.8 Hz, 2H), 3.82 (q, *J* = 7.0 Hz, 6H), 3.41 (td, *J* = 7.4, 5.7 Hz, 2H), 3.17 (s, 3H), 2.54 – 2.49 (m, 2H), 1.77 (s, 2H), 1.18 (t, *J* = 7.0 Hz, 9H).

**$^{13}$C NMR (101 MHz, Acetone-*d*$_6$)** δ 168.8, 140.2, 134.2, 120.98 (q, *J* = 321.3 Hz), 62.5, 59.0, 46.0, 44.4, 30.6, 18.5.

**$^{19}$F NMR (376 MHz, Acetone-*d*$_6$)** δ -78.59.

**ESI-MS**: calculated for $C_{17}H_{30}BF_3N_2NaO_9SSi^+$ $[M+Na]^+$, 557.1379; Found, 557.1363.

**Methyl (*E*)-6-(1,1,1,3,5,5,5-heptamethyltrisiloxan-3-yl)-7-(6-methyl-4,8-dioxo-1,3,6,2-dioxazaborocan-2-yl)hept-5-enoate (62)**

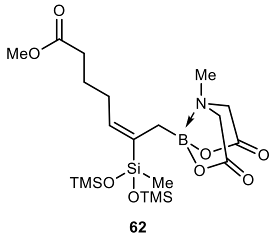


**62**

Following the **General procedure F,** the product **62** was obtained in 67% yield (34.6 mg, from **S-21**) as a colorless oil after column chromatography (eluent = ethyl acetate/petroleum ether v/v 2:1).

**$^{1}$H NMR (400 MHz, Acetone-*d*$_6$)** δ 5.74 (tt, *J* = 6.8, 1.3 Hz, 1H), 4.05 (d, *J* = 16.7 Hz,

2H), 3.91 (d, $J$ = 16.8 Hz, 2H), 3.50 (s, 3H), 3.01 (s, 3H), 2.21 (t, $J$ = 7.5 Hz, 2H), 2.07 – 2.01 (m, 2H), 1.63 – 1.54 (m, 4H), 0.05 (s, 3H), 0.01 (s, 18H).

**$^{13}$C NMR (101 MHz, Acetone-$d_6$)** δ 174.2, 168.6, 140.6, 138.3, 62.4, 51.5, 46.1, 34.0, 28.9, 25.3, 2.1, 0.7.

**ESI-MS**: calculated for $C_{20}H_{40}BNNaO_8Si_3^+$ $[M+Na]^+$, 540.2047; Found, 540.2047.

**(*E*)-2-(6-hydroxy-2-(triethoxysilyl)hex-2-en-1-yl)-6-methyl-1,3,6,2-dioxazaborocane-4,8-dione (63)**

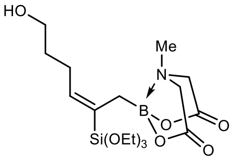


**63**

Following the **General procedure F,** the product **63** was obtained in 54% yield (23.8 mg, from **S-22**) as a colorless oil after column chromatography (eluent = ethyl acetate/acetonitrile v/v 8:1).

**$^1$H NMR (400 MHz, CD$_3$CN)** δ 5.98 (tt, $J$ = 6.8, 1.3 Hz, 1H), 3.90 (d, $J$ = 16.7 Hz, 2H), 3.84 – 3.74 (m, 8H), 3.50 (t, $J$ = 6.5 Hz, 2H), 2.93 (s, 3H), 2.20 – 2.15 (m, 2H), 1.66 (s, 2H), 1.62 – 1.54 (m, 2H), 1.17 (t, $J$ = 7.0 Hz, 9H).

**$^{13}$C NMR (101 MHz, CD$_3$CN)** δ 169.1, 145.8, 130.7, 62.6, 62.3, 59.2, 46.3, 33.0, 26.2, 18.5.

**ESI-MS**: calculated for $C_{17}H_{32}BNNaO_8Si^+$ $[M+Na]^+$, 440.1882; Found, 440.1877.

**(*E*)-2-(5-hydroxy-2-(triethylsilyl)pent-2-en-1-yl)-6-methyl-1,3,6,2-dioxazaborocane-4,8-dione (64)**

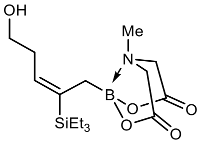


**64**

Following the **General procedure F,** the product **64** was obtained in 81% yield (28.8 mg, from **S-24**) as a colorless oil after column chromatography (eluent = ethyl acetate/acetonitrile v/v 8:1).

**$^{1}$H NMR (400 MHz, Acetone-*d*$_6$)** δ 5.74 (tt, *J* = 6.7, 1.3 Hz, 1H), 4.17 (d, *J* = 16.8 Hz, 2H), 4.06 (d, *J* = 16.8 Hz, 2H), 3.58 (t, *J* = 7.0 Hz, 2H), 3.14 (s, 3H), 2.40 – 2.35 (m, 2H), 1.69 (s, 2H), 0.92 (t, *J* = 7.8 Hz, 9H), 0.65 (q, *J* = 8.3 Hz, 6H).

**$^{13}$C NMR (101 MHz, Acetone-*d*$_6$)** δ 168.8, 137.7, 136.5, 62.5, 62.4, 46.1, 33.7, 7.9, 3.8.

**ESI-MS**: calculated for $C_{16}H_{30}BNNaO_5Si^+$ [M+Na]$^+$, 378.1879; Found, 378.1877.

**(*E*)-2-(6-((tert-butyldiphenylsilyl)oxy)-2-(triethoxysilyl)hex-2-en-1-yl)-6-methyl-1,3,6,2-dioxazaborocane-4,8-dione (65)**

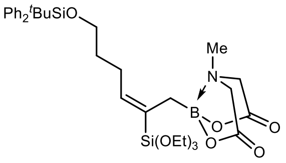


**65**

Following the **General procedure F,** the product **65** was obtained in 63% yield (41.3 mg, from **S-34**) as a colorless oil after column chromatography (eluent = ethyl acetate/petroleum ether v/v 2:1).

**$^{1}$H NMR (400 MHz, Acetone-*d*$_6$)** δ 7.75 – 7.67 (m, 4H), 7.50 – 7.39 (m, 6H), 6.02 (tt, *J* = 6.8, 1.3 Hz, 1H), 4.11 (d, *J* = 16.6 Hz, 2H), 3.98 (d, *J* = 16.6 Hz, 2H), 3.84 – 3.73 (m, 8H), 3.13 (s, 3H), 2.35 – 2.30 (m, 2H), 1.82 – 1.67 (m, 4H), 1.17 (t, *J* = 7.0 Hz, 9H), 1.05 (s, 9H).

**$^{13}$C NMR (101 MHz, Acetone-*d*$_6$)** δ 168.6, 145.3, 136.3, 134.8, 131.0, 130.5, 128.6, 64.3, 62.5, 58.9, 45.9, 32.8, 27.3, 26.1, 19.8, 18.5.

**ESI-MS**: calculated for $C_{33}H_{50}BNNaO_8Si_2^+$ [M+Na]$^+$, 678.3060; Found, 678.3064.

**(*E*)-5-(6-methyl-4,8-dioxo-1,3,6,2-dioxazaborocan-2-yl)-4-(triethoxysilyl)pent-3-en-1-yl benzoate (66)**

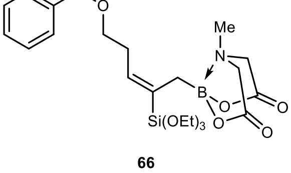


**66**

Following the **General procedure F,** the product **66** was obtained in 63% yield (31.9 mg, from **S-27**) as a colorless oil after column chromatography (eluent = ethyl acetate/petroleum ether v/v 4:1).

**$^{1}$H NMR (500 MHz, CD$_3$CN)** δ 8.02 – 7.98 (m, 2H), 7.63 – 7.58 (m, 1H), 7.48 (t, *J* = 7.8 Hz, 2H), 6.08 (t, *J* = 6.8 Hz, 1H), 4.38 (t, *J* = 6.5 Hz, 2H), 3.91 (d, *J* = 16.8 Hz, 2H), 3.82 (d, *J* = 16.9 Hz, 2H), 3.75 (q, *J* = 7.0 Hz, 6H), 2.93 (s, 3H), 2.68 – 2.61 (m, 2H), 1.71 (s, 2H), 1.13 (t, *J* = 7.0 Hz, 9H).

**$^{13}$C NMR (126 MHz, CD$_3$CN)** δ 169.0, 167.2, 140.8, 134.0, 133.7, 131.4, 130.2, 129.5, 65.1, 62.6, 59.2, 46.3, 29.4, 18.5.

**ESI-MS**: calculated for $C_{23}H_{34}BNNaO_9Si^+$ [M+Na]$^+$, 530.1988; Found, 530.1977.

**(*E*)-5-(1,1,1,3,5,5,5-heptamethyltrisiloxan-3-yl)-6-(6-methyl-4,8-dioxo-1,3,6,2-dioxazaborocan-2-yl)hex-4-en-1-yl 4-methylbenzenesulfonate (67)**

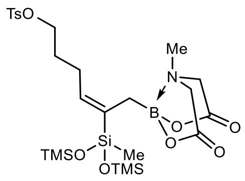


**67**

Following the **General procedure F,** the product **67** was obtained in 71% yield (44.7 mg, from **S-35**) as a colorless oil after column chromatography (eluent = ethyl acetate/petroleum ether v/v 4:1).

**$^{1}$H NMR (500 MHz, Acetone-*d*$_6$)** δ 7.84 – 7.78 (m, 2H), 7.48 (d, *J* = 8.0 Hz, 2H), 5.77 (t, *J* = 6.8 Hz, 1H), 4.17 (d, *J* = 16.8 Hz, 2H), 4.06 (t, *J* = 6.6 Hz, 2H), 4.02 (d, *J* = 16.8 Hz, 2H), 3.11 (s, 3H), 2.45 (s, 3H), 2.18 – 2.13 (m, 2H), 1.74 (tt, *J* = 6.5, 6.6 Hz, 2H), 1.67 (s, 2H), 0.14 (s, 3H), 0.10 (s, 18H).

**$^{13}$C NMR (101 MHz, Acetone-*d*$_6$)** δ 168.6, 145.6, 139.5, 138.9, 134.5, 130.8, 128.6, 71.4, 62.4, 46.1, 25.4, 21.5, 2.1, 0.6.

**ESI-MS**: calculated for $C_{25}H_{44}BNNaO_9SSi_3^+$ [M+Na]$^+$, 652.2030; Found, 652.2035.

**(*E*)-6-methyl-2-(4-methyl-2-(triethoxysilyl)pent-2-en-1-yl)-1,3,6,2-dioxazaborocane-4,8-dione (68)**

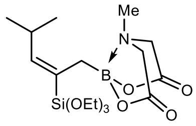

**68**

Following the **General procedure F,** the product **68** was obtained in 79% yield (31.7 mg, from **S-30**) as a colorless oil after column chromatography (reaction time: 7 h, eluent = ethyl acetate/petroleum ether v/v 2:1).

**$^{1}$H NMR (400 MHz, $CD_3CN$)** δ 5.75 (dt, *J* = 9.7, 1.3 Hz, 1H), 3.89 (d, *J* = 16.6 Hz, 2H), 3.83 – 3.74 (m, 8H), 2.92 (s, 3H), 2.78 – 2.68 (m, 1H), 1.64 (s, 2H), 1.17 (t, *J* = 7.0 Hz, 9H), 0.95 (d, *J* = 6.6 Hz, 6H).

**$^{13}$C NMR (101 MHz, Acetone-*d*$_6$)** δ 168.6, 153.0, 127.0, 62.6, 58.9, 45.9, 28.4, 22.6, 18.5.

**ESI-MS**: calculated for $C_{17}H_{32}BNNaO_7Si^+$ $[M+Na]^+$, 424.1933; Found, 424.1929.

**(*E*)-2-(3-cyclopropyl-2-(1,1,1,3,5,5,5-heptamethyltrisiloxan-3-yl)allyl)-6-methyl-1,3,6,2-dioxazaborocane-4,8-dione (69)**

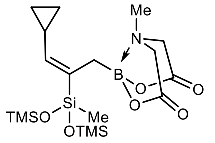

**69**

Following the **General procedure F,** the product **69** was obtained in 90% yield (41.1 mg, from **S-36**) as a colorless oil after column chromatography (eluent = ethyl acetate/petroleum ether v/v 2:1).

**$^{1}$H NMR (400 MHz, Acetone-*d*$_6$)** δ 5.19 (dt, *J* = 9.5, 1.3 Hz, 1H), 4.15 (d, *J* = 16.7 Hz, 2H), 4.00 (d, *J* = 16.7 Hz, 2H), 3.14 (s, 3H), 1.81 (s, 2H), 1.78 – 1.69 (m, 1H), 0.76 – 0.69 (m, 2H), 0.34 – 0.28 (m, 2H), 0.14 (s, 3H), 0.10 (s, 18H).

**$^{13}$C NMR (101 MHz, Acetone-*d*$_6$)** δ 168.6, 145.9, 135.1, 62.5, 46.1, 11.9, 7.6, 2.1, 0.7.

**ESI-MS**: calculated for $C_{18}H_{36}BNNaO_6Si_3^+$ $[M+Na]^+$, 480.1836; Found, 480.1842.

**(*E*)-2-(3-(1,1,1,3,5,5,5-heptamethyltrisiloxan-3-yl)oct-3-en-2-yl)-6-methyl-1,3,6,2-dioxazaborocane-4,8-dione (70)**

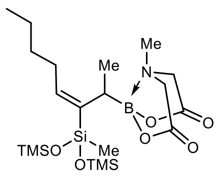


**70**

Following the **General procedure F,** the product **70** was obtained in 92% yield (44.8 mg, from **S-33**) as a colorless oil after column chromatography (reaction time: 7 h, catalyst: $[CpRu(MeCN)_3]PF_6$ (5 mol%), eluent = ethyl acetate/petroleum ether v/v 2:1).

**$^1$H NMR (400 MHz, DMSO-*d*$_6$)** δ 5.77 (t, *J* = 6.8 Hz, 1H), 4.20 (d, *J* = 17.2 Hz, 1H), 4.11 (d, *J* = 17.0 Hz, 1H), 4.01 (d, *J* = 17.0 Hz, 1H), 3.85 (d, *J* = 17.1 Hz, 1H), 2.86 (s, 3H), 2.24 – 1.94 (m, 3H), 1.36 – 1.22 (m, 4H), 0.98 (d, *J* = 7.2 Hz, 3H), 0.90 – 0.84 (m, 3H), 0.08 (d, *J* = 2.3 Hz, 21H).

**$^{13}$C NMR (101 MHz, DMSO-*d*$_6$)** δ 168.8, 168.6, 143.1, 140.4, 62.1, 61.7, 45.4, 31.3, 21.9, 16.0, 13.9, 2.0.

**$^{11}$B NMR (128 MHz, Acetone-*d*$_6$)** δ 12.82.

**ESI-MS**: calculated for $C_{20}H_{42}BNNaO6Si_3^+$ $[M+Na]^+$, 510.2305; Found, 510.2309.

## 4. Synthetic Utilities.

### 4.1 Synthesis of 73

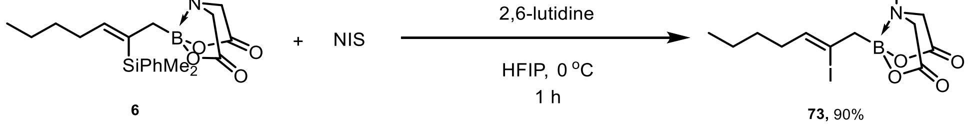


At 0 ºC, to a 4 mL vial charged with **6** (38.7 mg, 0.1 mmol, *Z:E* > 20:1) and a solution of 2,6-lutidine (7.5 mg, 0.07 mmol, 0.7 equiv.) in $(CF_3)_2CHOH$ (0.5 mL) was added *N*-iodosuccinimide (45.0 mg, 0.2 mmol, 2.0 equiv) in one portion. The reaction mixture was stirred at 0 °C for 1 h and then quenched with an aqueous solution of $Na_2S_2O_3$ (10 mL, 20 wt%). The mixture was extracted with EA (10 mL× 2). The combined organic

layers were dried over anhydrous $Na_2SO_4$ and concentrated under reduced pressure. The residue was purified by silica gel flash column chromatography (eluent = ethyl acetate/petroleum ether v/v 2:1) to give the desired product **73** as a white solid in 90% yield (34.0 mg, *Z:E* > 20:1).

**(*Z*)-2-(2-iodohept-2-en-1-yl)-6-methyl-1,3,6,2-dioxazaborocane-4,8-dione (73)**

**$^1$H NMR (400 MHz, DMSO-*d*$_6$)** δ 5.41 (t, *J* = 6.7 Hz, 1H), 4.19 (d, *J* = 17.0 Hz, 2H), 3.97 (d, *J* = 17.0 Hz, 2H), 2.85 (s, 3H), 2.25 (s, 2H), 2.02 (m, 2H), 1.37 – 1.26 (m, 4H), 0.88 (t, *J* = 6.9 Hz, 3H).

**$^{13}$C NMR (101 MHz, DMSO-*d*$_6$)** δ 169.2, 134.5, 105.0, 62.1, 46.0, 37.0, 30.6, 22.1, 14.3.

**$^{11}$B NMR (128 MHz, Acetone-*d*$_6$)** δ 11.86.

**ESI-MS**: calculated for $C_{12}H_{19}BINNaO_4^+$ $[M+Na]^+$, 402.0344; Found, 402.0340.

## 4.2 Synthesis of 74

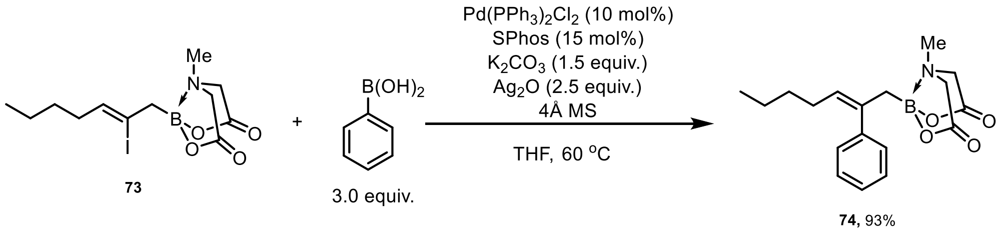


A flame-dried 15 mL Schlenk tube was charged with **73** (37.9 mg, 0.1 mmol, 1.0 equiv, *Z:E* > 20:1), $PhB(OH)_2$ (36.6 mg, 0.3 mmol, 3.0 equiv.) and $K_2CO_3$ (20.5 mg, 0.15 mmol, 1.5 equiv.), then the tube was brought into the glove box. In the glove box, $Pd(PPh_3)_2Cl_2$ (7.0 mg, 0.01 mmol, 10 mol%), SPhos (6.2 mg, 0.015 mmol, 15 mol%), $Ag_2O$ (58.0 mg, 0.25 mmol, 2.5 equiv), 4Å MS and anhydrous THF (0.1 M, 1 mL) was added in order. Then the tube was carried out of the glove box and heated to 60 °C for 5 h. After completion, the crude mixture was filtered through a pad of celite, then concentrated and purified by flash column chromatography (eluent = ethyl acetate/petroleum ether v/v 2:1) to give the desired product **74** as a light yellow oil in 93% yield (30.5 mg, *Z:E* > 20:1).

**(*Z*)-6-methyl-2-(2-phenylhept-2-en-1-yl)-1,3,6,2-dioxazaborocane-4,8-dione (74)**

**$^{1}$H NMR (400 MHz, DMSO-*d*$_6$)** δ 7.35 – 7.11 (m, 5H), 5.39 (t, *J* = 7.3 Hz, 1H), 4.13 (d, *J* = 17.0 Hz, 2H), 3.87 (d, *J* = 17.0 Hz, 2H), 2.78 (s, 3H), 1.91 – 1.78 (m, 4H), 1.33 – 1.17 (m, 4H), 0.77 (t, *J* = 7.0 Hz, 3H).

**$^{13}$C NMR (101 MHz, DMSO-*d*$_6$)** δ 168.8, 142.9, 138.0, 128.1, 127.7, 126.5, 126.0, 61.5, 45.5, 31.9, 28.4, 21.7, 13.8.

**ESI-MS:** calculated for $C_{18}H_{24}BNNaO_4^+$ $[M+Na]^+$, 352.1691; Found, 352.1692.

### 4.3 Synthesis of 75

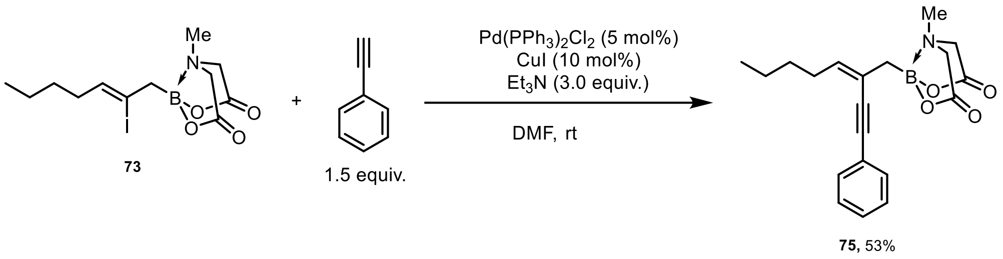


A flame-dried 15 mL Schlenk tube was charged with **75** (37.9 mg, 0.1 mmol, 1.0 equiv., *Z:E* > 20:1), then the tube was brought into the glove box. In the glove box, $Pd(PPh_3)_2Cl_2$ (3.5 mg, 0.005 mmol, 5 mol%), CuI (1.9 mg, 0.01 mmol, 10 mol%), phenylacetylene (15.0 mg, 0.15 mmol, 1.5 equiv.), $Et_3N$ (30.3 mg, 0.3 mmol, 3.0 equiv), anhydrous DMF (0.25 M, 0.4 mL) were added in order. Then the tube was carried out of the glove box and stirred at room temperature for 1 h. After completion, the crude mixture was filtered through a pad of celite, then concentrated and purified by flash column chromatography (eluent = ethyl acetate/petroleum ether v/v 2:1 to 3:1) to give the desired product **75** as a light yellow oil in 53% yield (18.6 mg, *Z:E* = 13:1).

**(*Z*)-6-methyl-2-(2-(phenylethynyl)hept-2-en-1-yl)-1,3,6,2-dioxazaborocane-4,8-dione (75)**

**$^{1}$H NMR (400 MHz, Acetone-*d*$_6$)** δ 7.52 – 7.46 (m, 2H), 7.40 – 7.32 (m, 3H), 5.83 (tt, *J* = 7.5, 1.3 Hz, 1H), 4.21 (d, *J* = 16.8 Hz, 2H), 4.00 (d, *J* = 16.6 Hz, 2H), 3.24 (s, 3H), 2.35 (q, *J* = 7.2 Hz, 2H), 1.84 (s, 2H), 1.46 – 1.31 (m, 4H), 0.92 (t, *J* = 7.2 Hz, 3H).

**$^{13}$C NMR (101 MHz, Acetone-*d*$_6$)** δ 168.6, 139.7, 132.1, 129.3, 128.8, 124.8, 120.5, 94.1, 90.6, 63.0, 46.4, 32.1, 31.2, 23.0, 14.2.

**ESI-MS**: calculated for $C_{20}H_{24}BNNaO_4^+$ $[M+Na]^+$, 376.1691; Found, 376.1698.

### 4.4 Synthesis of 76

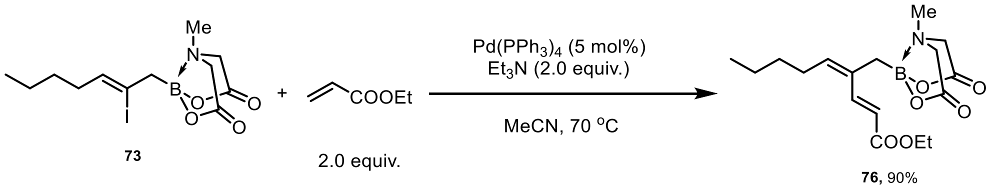


A flame-dried 15 mL Schlenk tube was charged with **73** (37.9 mg, 0.1 mmol, 1 equiv, *Z:E* > 20:1), then the tube was brought into the glove box. In the glove box, $Pd(PPh_3)_4$ (5.5 mg, 0.005 mmol, 5 mol%), ethyl acrylate (20.0 mg, 0.15 mmol, 1.5 equiv.), $Et_3N$ (20.2 mg, 0.2 mmol, 2.0 equiv.), anhydrous MeCN (0.2 M, 0.5 mL) were added in order. Then the tube was carried out of the glove box and stirred at 70 °C for 2 h. After completion, the crude mixture was filtered through a pad of celite, then concentrated and purified by flash column chromatography (eluent = ethyl acetate/petroleum ether v/v 3:1 to 4:1) to give the desired product **76** as a light-yellow oil in 90% yield (31.5 mg, *Z:E* > 20:1).

**Ethyl (2*E*,4*Z*)-4-((6-methyl-4,8-dioxo-1,3,6,2-dioxazaborocan-2-yl)methyl)nona-2,4-dienoate (76)**

**$^1$H NMR (500 MHz, Acetone-*d*$_6$)** δ 7.76 (d, *J* = 15.6 Hz, 1H), 6.00 (d, *J* = 15.6 Hz, 1H), 5.83 (t, *J* = 7.8 Hz, 1H), 4.21 – 4.13 (m, 4H), 4.06 (d, *J* = 16.8 Hz, 2H), 3.18 (s, 3H), 2.30 (q, *J* = 7.2 Hz, 2H), 1.77 (s, 2H), 1.44 – 1.31 (m, 4H), 1.25 (t, *J* = 7.2 Hz, 3H), 0.91 (t, *J* = 7.2 Hz, 3H).

**$^{13}$C NMR (126 MHz, Acetone-*d*$_6$)** δ 168.7, 167.9, 142.1, 139.5, 133.8, 119.0, 62.6, 60.4, 46.3, 32.7, 28.4, 22.8, 14.6, 14.2.

**ESI-MS**: calculated for $C_{17}H_{26}BNNaO_6^+$ $[M+Na]^+$, 374.1745; Found, 374.1744.

### 4.5 Synthesis of 77

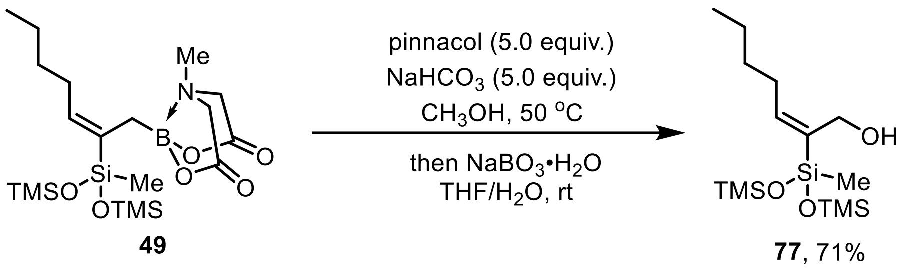

Step 1: To a 15 ml vial were added **49** (0.2 mmol, 1.0 equiv, *E/Z* > 20:1), pinacol (1.0 mmol, 5.0 equiv) and $NaHCO_3$ (1.0 mmol, 5.0 equiv). The vial was evacuated with Ar for three times, and then MeOH (4.0 mL) was injected. The mixture was stirred at 50 °C overnight. The solution was concentrated under reduced pressure and purified by flash chromatography on short silica gel to afford the silyl substituted allylic Bpin as a colorless oil.

Step 2: To a 15 ml vial were added silyl substituted allylic Bpin, $NaBO_3 \cdot H_2O$ (0.6 mmol, 3 equiv.), $THF/H_2O$ (0.5 mL/0.5 mL). The mixture was stirred at room temperature for 4 h and then quenched with an aqueous solution of $Na_2S_2O_3$ (10 mL, 20 wt%). The mixture was extracted with EA (10 mL× 2). The combined organic layers were dried over anhydrous $Na_2SO_4$ and concentrated under reduced pressure. The residue was purified by silica gel flash column chromatography (eluent = ethyl acetate/petroleum ether v/v 1:20) to give the desired product **77** as a colorless oil in 71% yield (47.4 mg, from **49**, 2 steps, *E/Z* > 20:1).

**(*E*)-2-(1,1,1,3,5,5,5-heptamethyltrisiloxan-3-yl)hept-2-en-1-ol (77)**

**$^1$H NMR (400 MHz, CDCl$_3$)** δ 8.02 – 7.96 (m, 2H), 7.55 – 7.48 (m, 2H), 6.01 (t, *J* = 7.5 Hz, 1H), 4.59 (s, 2H), 3.90 (s, 3H), 2.31 (q, *J* = 7.4 Hz, 2H), 1.51 – 1.33 (m, 4H), 0.93 (t, *J* = 7.2 Hz, 3H).

**$^{13}$C NMR (101 MHz, CDCl$_3$)** δ 167.1, 145.8, 138.1, 135.0, 129.9, 128.7, 126.2, 59.6, 52.2, 32.0, 28.4, 22.6, 14.1.

**ESI-MS**: calculated for $C_{14}H_{34}NaO_3Si_3^+$ [M+Na]$^+$, 357.1708; Found, 357.1714.

**4.6 Synthesis of 78**

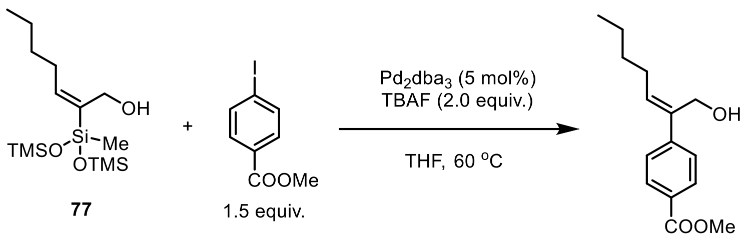


To a solution of **77** (33.4 mg, 0.1 mmol, *E/Z* > 20:1) and methyl 4-iodobenzoate (39.3 mg, 0.15 mmol) in THF (0.5 mL) at 23 °C under argon were added $Pd_2(dba)_3$ (4.6 mg, 0.005 mmol), and the mixture was stirred for 5 min. Then TBAF (0.2 mL, 1.0 M in THF, 0.2 mmol) was added dropwise. The flask was sealed, heated to 60 °C and stirred for 4 h. The mixture was extracted with EA (2 x 20 mL), at which point water (5 mL) was added, and the combined organic layers were washed with brine (30 mL) and dried over $MgSO_4$. The solvent was removed in vacuo, and the resulting residue was purified by flash chromatography (eluent = ethyl acetate/petroleum ether v/v 1:10 to 1:5) to afford **78** in 68% yield (16.9 mg, from **77**, *E/Z* > 20:1) as a light-yellow oil.

**methyl (Z)-4-(1-hydroxyhept-2-en-2-yl)benzoate (78)**

**$^1$H NMR (400 MHz, $CDCl_3$)** δ 8.02 – 7.96 (m, 2H), 7.55 – 7.48 (m, 2H), 6.01 (t, *J* = 7.5 Hz, 1H), 4.59 (s, 2H), 3.90 (s, 3H), 2.31 (q, *J* = 7.4 Hz, 2H), 1.51 – 1.33 (m, 4H), 0.93 (t, *J* = 7.2 Hz, 3H).

**$^{13}$C NMR (101 MHz, $CDCl_3$)** δ 167.1, 145.8, 138.1, 135.0, 129.9, 128.7, 126.2, 59.6, 52.2, 32.0, 28.4, 22.6, 14.1.

**ESI-MS**: calculated for $C_{15}H_{20}NaO_3^+$ $[M+Na]^+$, 271.1305; Found, 271.1308.

## 4.6 General procedure G for the synthesis of homoallylic alcohols.

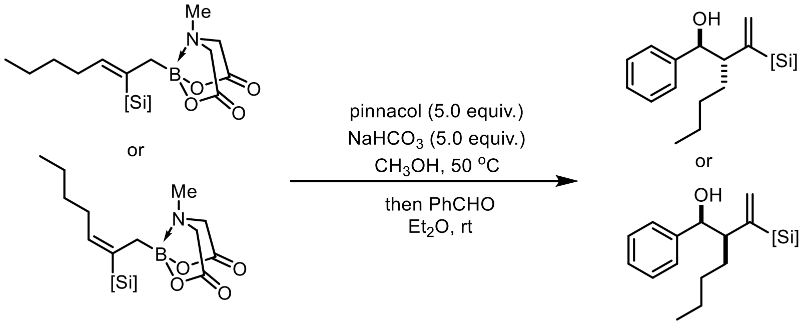


Step 1: To a 15 ml vial were added silyl-substituted allylic B(MIDA) (0.2 mmol, 1.0

equiv), pinacol (1.0 mmol, 5.0 equiv) and $NaHCO_3$ (1.0 mmol, 5.0 equiv). The vial was evacuated with Ar for three times, and then MeOH (4.0 mL) was injected. The mixture was stirred at 50 °C overnight. The solution was concentrated under reduced pressure and purified by flash chromatography on short silica gel to afford the corresponding product as a colorless oil.

Step 2: To a 15 ml vial were added silyl substituted allylic Bpin, PhCHO (1.3 equuiv.), $Et_2O$ (2 mL, 0.1 M). The mixture was stirred at room temperature for 24 h. The solution was concentrated under reduced pressure and purified by flash chromatography on short silica gel to afford the corresponding product.

***anti*-2-(1-(dimethyl(phenyl)silyl)vinyl)-1-phenylhexan-1-ol (79)**

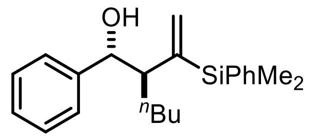


**79**

Following the **General procedure G**, the product **79** was obtained in 96% yield (64.8 mg, from **4,** 2 steps) as a colorless oil after column chromatography (eluent = ethyl acetate/petroleum ether v/v 1:50, dr > 20:1).

**$^1$H NMR (400 MHz, CDCl$_3$)** δ 7.60 – 7.51 (m, 2H), 7.39 – 7.16 (m, 8H), 5.89 (d, *J* = 2.6 Hz, 1H), 5.73 (d, *J* = 2.4 Hz, 1H), 4.36 (d, *J* = 9.0 Hz, 1H), 2.48 (td, *J* = 10.1, 3.0 Hz, 1H), 1.94 (d, *J* = 2.3 Hz, 1H), 1.20 – 0.84 (m, 6H), 0.64 (t, *J* = 6.8 Hz, 3H), 0.44 (d, *J* = 7.2 Hz, 6H).

**$^{13}$C NMR (101 MHz, CDCl$_3$)** δ 152.2, 143.1, 138.2, 134.1, 130.5, 129.3, 128.3, 128.0, 127.7, 127.3, 127.2, 77.1, 55.5, 30.9, 29.7, 22.7, 14.0, -1.9.

**ESI-MS**: calculated for $C_{22}H_{30}NaOSi^+$ $[M+Na]^+$, 361.1958; Found, 361.1945.

***anti*-1-phenyl-2-(1-(triethylsilyl)vinyl)hexan-1-ol (80)**

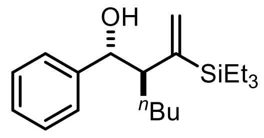


**80**

Following the **General procedure G**, the product **80** was obtained in 84% yield (53.4 mg, from **3,** 2 steps) as a colorless oil after column chromatography (eluent = ethyl acetate/petroleum ether v/v 1:50, dr > 20:1).

**$^1$H NMR (400 MHz, CDCl$_3$)** δ 7.43 – 7.16 (m, 5H), 5.93 (dd, *J* = 2.5, 0.8 Hz, 1H), 5.70 (d, *J* = 2.5 Hz, 1H), 4.55 (d, *J* = 8.9 Hz, 1H), 2.53 (td, *J* = 9.1, 3.2 Hz, 1H), 2.13 (s, 1H), 1.32 – 1.10 (m, 6H), 0.99 (t, *J* = 7.9 Hz, 9H), 0.76 (t, *J* = 7.1 Hz, 3H), 0.73 – 0.65 (m, 6H).

**$^{13}$C NMR (101 MHz, CDCl$_3$)** δ 150.7, 143.0, 129.5, 128.3, 127.7, 127.3, 77.2, 54.6, 31.1, 29.5, 22.9, 14.0, 7.5, 3.6.

**ESI-MS**: calculated for $C_{20}H_{34}NaOSi^+$ [M+Na]$^+$, 341.2271; Found, 341.2270.

***syn*-2-(1-(1,1,1,3,5,5,5-heptamethyltrisiloxan-3-yl)vinyl)-1-phenylhexan-1-ol (81)**

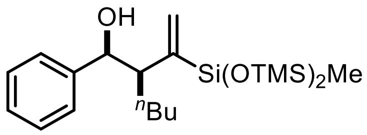


**81**

Following the **General procedure G**, the product **81** was obtained in 64% yield (54.3 mg, from **49,** 2 steps) as a colorless oil after column chromatography (eluent = ethyl acetate/petroleum ether v/v 1:50, dr > 20:1).

**$^1$H NMR (500 MHz, CDCl$_3$)** δ 7.35 – 7.30 (m, 4H), 7.25 – 7.20 (m, 1H), 5.64 (d, *J* = 2.6 Hz, 1H), 5.61 (d, *J* = 2.6 Hz, 1H), 4.80 (d, *J* = 4.3 Hz, 1H), 2.66 (dt, *J* = 11.0, 3.8 Hz, 1H), 1.61 – 1.37 (m, 2H), 1.28 – 1.11 (m, 4H), 0.79 (t, *J* = 7.0 Hz, 3H), 0.15 (d, *J* = 2.6 Hz, 18H), 0.12 (s, 3H).

**$^{13}$C NMR (126 MHz, CDCl$_3$)** δ 149.3, 141.3, 126.4, 126.0, 124.8, 124.4, 73.4, 50.5, 28.0, 24.2, 20.9, 12.1, 0.0, -1.7.

**ESI-MS**: calculated for $C_{21}H_{40}NaO_3Si_3^+$ [M+Na]$^+$, 447.2177; Found, 447.2181.

# 5. Mechanism studies

## 5.1 comparations to propargylic Bpin, BTIDA, BF$_3$K, Bdan

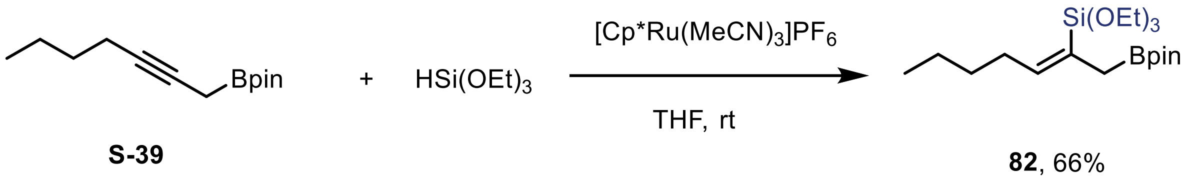


**82**, 66%
*Z/E* > 20:1, β/γ = 4.5:1

In a glove box, to an oven-dried 15 mL vial were added **S-39** (0.10 mmol), $HSi(OEt)_3$ (0.20 mmol), $[Cp^*Ru(MeCN)_3]PF_6$ (2.5 mg, 5.0 μmol), and THF (1.0 mL). The vial was capped and removed from the glove box. The reaction mixture was stirred at room temperature for 30 min, and then concentrated under reduced pressure. The residue was purified by silica gel flash column chromatography (petroleum as the eluent) to afford **82** in 66% yield (25.5 mg, β/γ = 4.5:1) as a light-yellow oil.

**(*Z*)-triethoxy(1-(4,4,5,5-tetramethyl-1,3,2-dioxaborolan-2-yl)hept-2-en-2-yl)silane (mixture of *β* & *γ* isomers)**

**$^1$H NMR (500 MHz, $CDCl_3$)** δ (β selectivity) 6.09 (t, *J* = 7.5 Hz, 1H), 2.21 (q, *J* = 7.1 Hz, 4H), 1.74 (s, 3H). (γ selectivity) 6.24 (t, *J* = 8.3 Hz, 1H), 2.08 (t, *J* = 7.6 Hz, 2H). (overlap) 3.81 (q, *J* = 6.9 Hz, 6H), 1.39 – 1.30 (m, 4H), 1.24 – 1.18 (m, 21H), 0.91 – 0.85 (m, 3H).

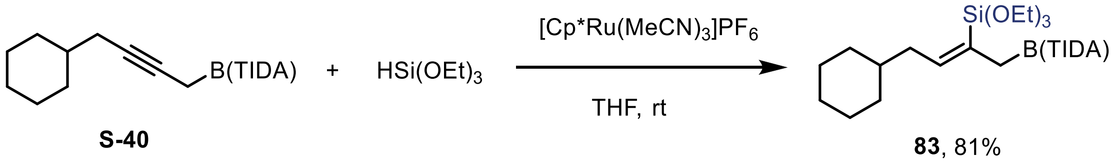


**83**, 81%
*Z/E* = 10:1, β/γ > 20:1

In a glove box, to an oven-dried 15 mL vial were added **S-40** (0.10 mmol), $HSi(OEt)_3$ (0.20 mmol), $[Cp^*Ru(MeCN)_3]PF_6$ (2.5 mg, 5.0 μmol), and THF (1.0 mL). The vial was capped and removed from the glove box. The reaction mixture was stirred at room temperature for 30 min, and then concentrated under reduced pressure. The residue was purified by silica gel flash column chromatography (eluent = ethyl acetate/petroleum ether v/v 1:1) to afford **83** in 81% yield (41.4 mg, *Z/E* = 10:1) as a white solid.

**(*Z*)-2-(4-cyclohexyl-2-(triethoxysilyl)but-2-en-1-yl)-5,5,6,7,7-pentamethyl-1,3,6,2-dioxazaborocane-4,8-dione (83)**

**$^1$H NMR (400 MHz, $CD_3CN$)** δ 6.18 (t, *J* = 7.5 Hz, 1H), 3.78 (q, *J* = 7.0 Hz, 6H), 2.54

(s, 3H), 2.10 (t, $J$ = 7.2 Hz, 2H), 1.79 – 1.53 (m, 20H), 1.28 – 1.11 (m, 14H).

**$^{13}$C NMR (101 MHz, $CD_3CN$)** δ 175.4, 146.3, 130.9, 58.8, 40.8, 39.6, 36.9, 34.0, 27.4, 27.2, 18.6.

**ESI-MS**: calculated for $C_{25}H_{46}BNNaO_7Si^+$ $[M+Na]^+$, 534.3029; Found, 534.3035.

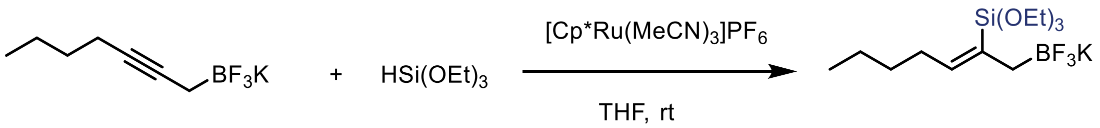


**S-41**

NR

In a glove box, to an oven-dried 15 mL vial were added **S-41** (0.10 mmol), $HSi(OEt)_3$ (0.20 mmol), $[Cp^*Ru(MeCN)_3]PF_6$ (2.5 mg, 5.0 μmol), and THF (1.0 mL). The vial was capped and removed from the glove box. The reaction mixture was stirred at room temperature for 30 min, and then concentrated under reduced pressure. The residue was directly monitored by $^1$H NMR and no reaction was observed, even we change the solvent to THF, dioxane, MeCN, acetone.

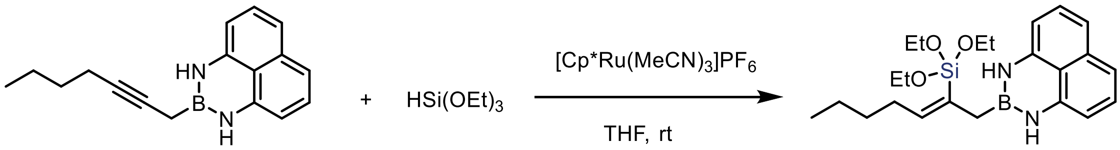


**S-42**

**85,** 15%
*Z/E* > 20:1, r.r. > 20:1

In a glove box, to an oven-dried 15 mL vial were added **S-42** (0.10 mmol), $HSi(OEt)_3$ (0.20 mmol), $[Cp^*Ru(MeCN)_3]PF_6$ (2.5 mg, 5.0 μmol), and THF (1.0 mL). The vial was capped and removed from the glove box. The reaction mixture was stirred at room temperature for 30 min, and then concentrated under reduced pressure. The product could only be detected by crude $^1$H NMR.

### 5.2 Regioselectivity of homo-propargylic B(MIDA): lack of regioselectivity

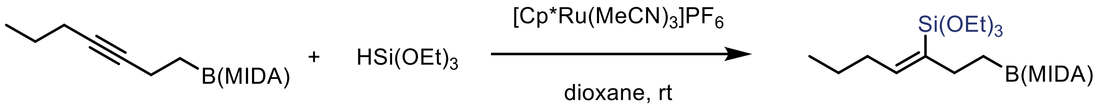


**S-43**

**86**, 99%
*Z/E* > 20:1, γ/δ = 4:1

In a glove box, to an oven-dried 15 mL vial were added **S-43** (0.10 mmol), $HSi(OEt)_3$ (0.20 mmol), $[Cp^*Ru(MeCN)_3]PF_6$ (2.5 mg, 5.0 μmol), and 1,4-dioxane (1.0 mL). The vial was capped and removed from the glove box. The reaction mixture was stirred at room temperature for 30 min, and then concentrated under reduced pressure. The residue was purified by silica gel flash column chromatography (eluent = ethyl acetate/petroleum ether v/v 2:1) to afford **86** in 99% yield (41.0 mg, *γ/δ* = 4:1) as a colorless oil.

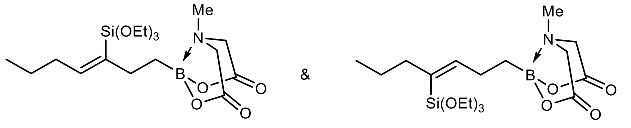


**$^1$H NMR (400 MHz, DMSO)** (mixture of *γ/δ* isomers: overlapped) δ 6.15 – 6.04 (m, 1H), 4.19 – 4.14 (m, *J* = 2H), 3.99 – 3.93 (m, 2H), 3.74 (q, *J* = 7.0 Hz, 6H), 2.85 (s, 3H), 2.19 – 2.12 (m, 2H), 2.06 – 1.95 (m, 2H), 1.41 – 1.26 (m, 2H), 1.15 (t, *J* = 6.9 Hz, 9H), 0.88 (t, *J* = 7.4 Hz, 3H), 0.67 – 0.56 (m, 2H).

**$^{13}$C NMR (101 MHz, DMSO)** (mixture of *γ/δ* isomers) δ 169.4, 169.3, 150.8, 145.3, 135.4, 130.1, 62.1, 62.0, 58.0, 46.0, 45.9, 33.9, 32.5, 23.5, 23.0, 18.6, 14.2, 14.1.

### 5.3 Regioselectivity of hex-4-yn-1-ylbenzene: lack of regioselectivity

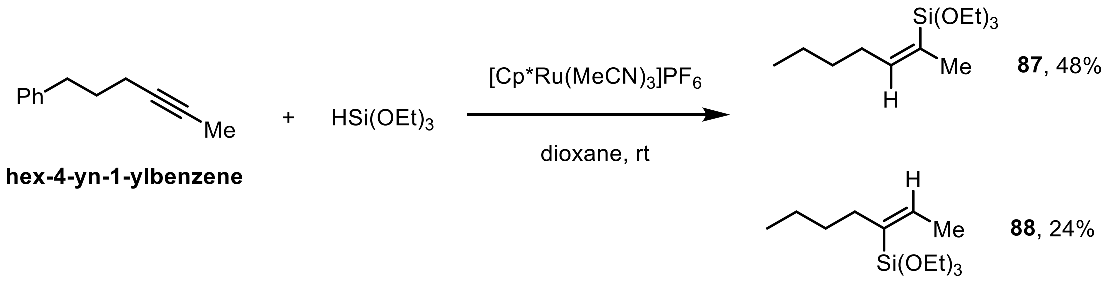


In a glove box, to an oven-dried 15 mL vial were added hex-4-yn-1-ylbenzene (0.20 mmol), $HSi(OEt)_3$ (0.40 mmol), $[Cp^*Ru(MeCN)_3]PF_6$ (5.0 mg, 10.0 μmol), and dioxane (2.0 mL). The vial was capped and removed from the glove box. The reaction mixture was stirred at room temperature for 30 min, and then concentrated under reduced pressure. The residue was purified by silica gel flash column chromatography (eluent = ethyl acetate/petroleum ether v/v 1:50) to afford mixture of **87** & **88** in 72% yield (37.4 mg, **87**/**88** = 2:1, see Supplementary Figure 2) as a colorless oil.

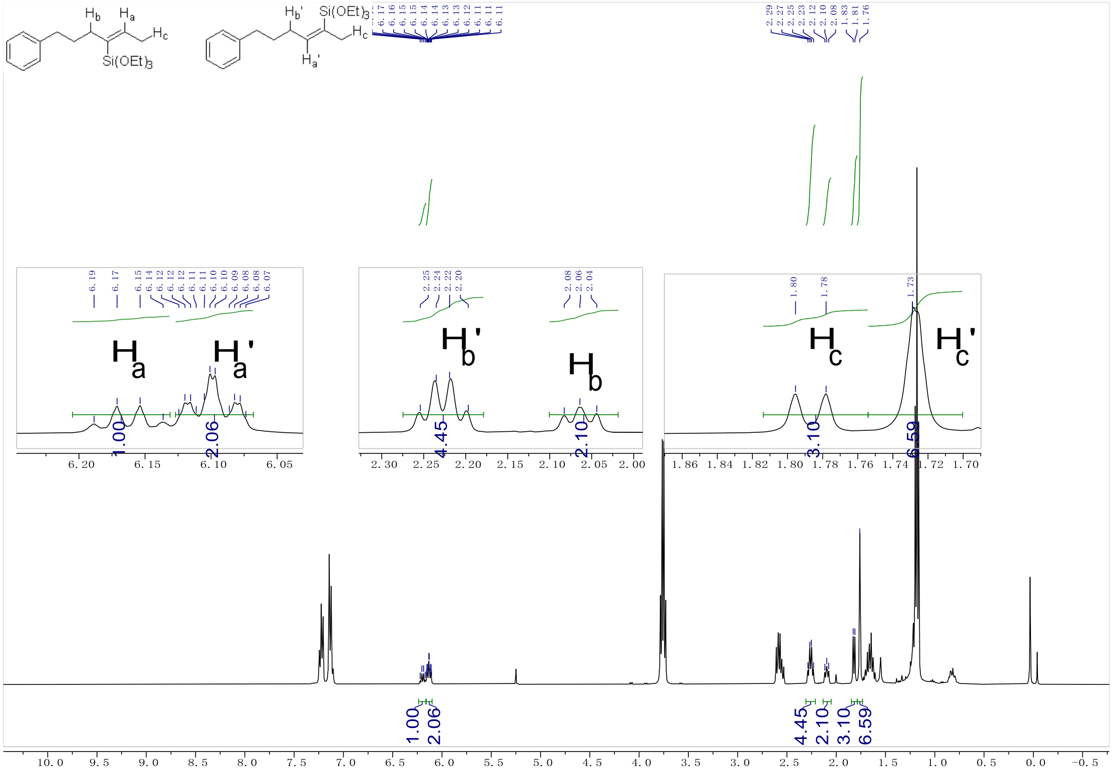

**Supplementary Figure 2: $^{1}$H NMR of mixture of 87 & 88 (400 MHz, $CDCl_3$)** δ 6.21 (q, $J$ = 7.0 Hz, 1H), 6.14 (t, $J$ = 7.6 Hz, 2H), 2.32 – 2.22 (m, 4H), 2.15 – 2.06 (m, 2H), 1.83 (d, $J$ = 7.0 Hz, 3H), 1.77 (s, 6H).

## 5.4 The ligand effect

**hydrosilylation when 5 mol% of $AgPF_6$ was added to precipitate the chloride from the catalyst.**

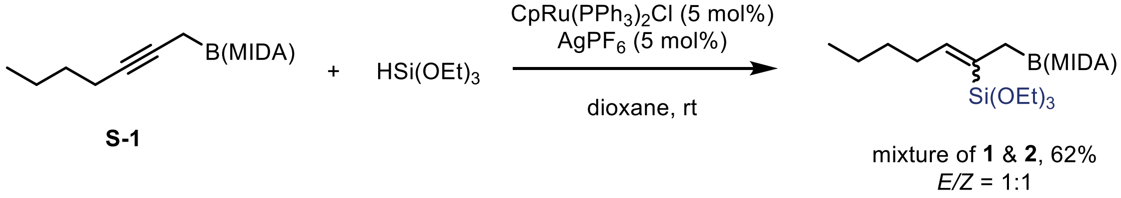


In a glove box, to an oven-dried 15 mL vial were added **S-1** (0.10 mmol), $HSi(OEt)_3$ (0.20 mmol), $CpRu(PPh_3)_2Cl$ (3.6 mg, 5.0 μmol), $AgPF_6$ (1.3 mg, 5.0 μmol) and 1,4-dioxane (1.0 mL). The vial was capped and removed from the glove box. The reaction mixture was stirred at room temperature for 3 h, and then concentrated under reduced pressure. The residue was purified by silica gel flash column chromatography (eluent = ethyl acetate/petroleum ether v/v 2:1) to afford mixture of **1 & 2** in 62% yield (25.7 mg,

*E/Z* = 1:1, see Supplementary Figure 3) as a colorless oil.

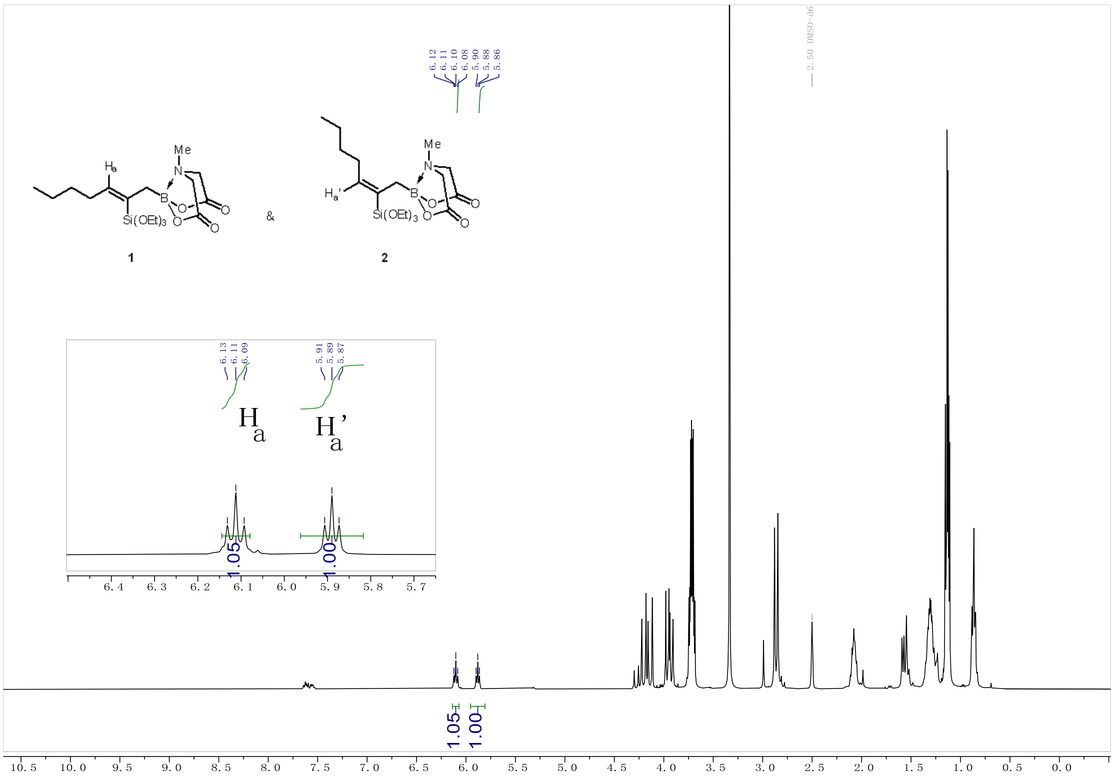

**Supplementary Figure 3: $^{1}$H NMR of mixture of 1 & 2 (400 MHz, DMSO-$d_6$)** δ 6.10 (t, *J* = 7.8 Hz, 1H), 5.88 (t, *J* = 6.7 Hz, 1H).

**hydrosilylation of S-1 when 10 mol% of $PPh_3$ was added with $[CpRu(MeCN)_3]PF_6$**

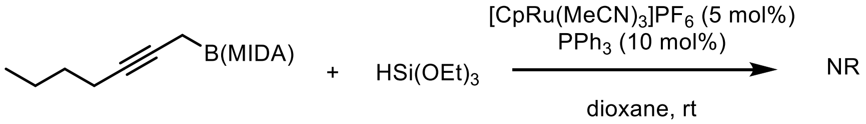


In a glove box, to an oven-dried 15 mL vial were added **S-1** (0.10 mmol), $HSi(OEt)_3$ (0.20 mmol), $[CpRu(MeCN)_3]PF_6$ (2.2 mg, 5.0 μmol), $PPh_3$ (2.6 mg, 10.0 μmol) and 1,4-dioxane (1.0 mL). The vial was capped and removed from the glove box. The reaction mixture was stirred at room temperature for 3 h, and then concentrated under reduced pressure. The residue was directly monitored by $^{1}$H NMR and no reaction was observed.

**hydrosilylation of S-1 when 10 mol% of $PPh_3$ and 1 mg LiCl was added with $[CpRu(MeCN)_3]PF_6$**

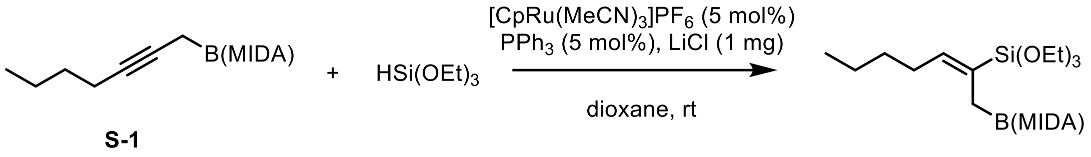


**2**, 42%
*E/Z* = 14:1, r.r. > 20:1

In a glove box, to an oven-dried 15 mL vial were added **S-1** (0.10 mmol), $HSi(OEt)_3$ (0.20 mmol), $[CpRu(MeCN)_3]PF_6$ (2.2 mg, 5.0 μmol), $PPh_3$ (2.6 mg, 10.0 μmol), LiCl (1 mg) and 1,4-dioxane (1.0 mL). The vial was capped and removed from the glove box. The reaction mixture was stirred at room temperature for 3 h, and then concentrated under reduced pressure. The residue was directly monitored by $^1$H NMR. The *E* isomer product **2** was observed in 42% yield (*E*/*Z* = 14:1, r.r. > 20:1, see Supplementary Figure 4).

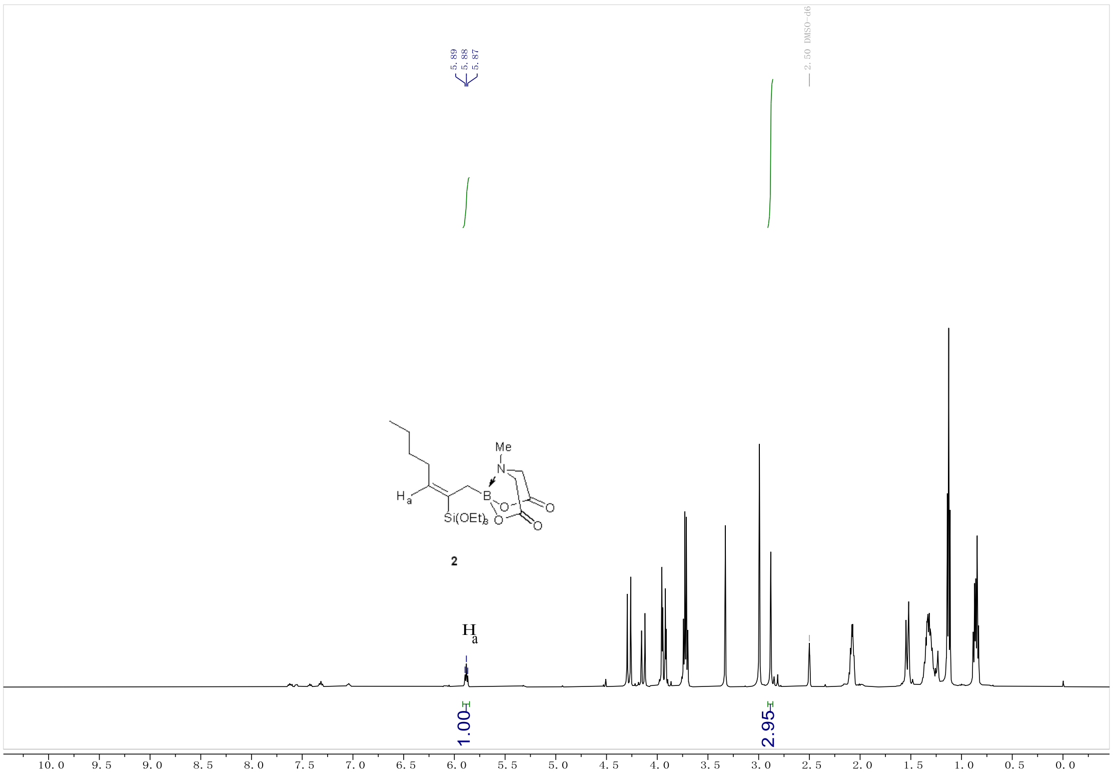

**Supplementary Figure 4: $^1$H NMR of 2 (major) (500 MHz, DMSO-$d_6$)** δ 5.88 (t, *J* = 6.7 Hz, 1H), 2.88 (s, 3H).

## 5.5 Deuteration experiments

### Synthesis of $DSiEt_3$

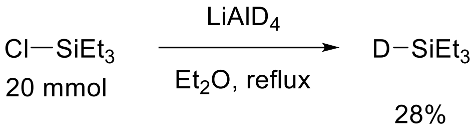


In a flame-dried round-bottomed flask under an argon atmosphere a solution of $LiAlD_4$ (680 mg, 16 mmol, 0.8 equiv.) in anhydrous $Et_2O$ (15 mL) was prepared. Then, $Et_3SiCl$ (3.0 g, 20 mmol, 1 equiv.) was added dropwise to the solution at 0 °C. The reaction was stirred under reflux at 40 °C for 16 h and then cooled to rt. The reaction was quenched by slow addition of HCl (10 mL, 2 N) at 0 °C before the mixture was extracted with $Et_2O$ (10 x 3 mL). The combined organic phases were dried over anhydrous $Na_2SO_4$ and concentrated under reduced pressure. Purification of the residue by vacuum distillation afforded $DSiEt_3$ as a colorless liquid (660 mg, 28%, >99% D).

**$^1$H NMR (500 MHz, $CDCl_3$)** δ 0.98 (t, *J* = 8.0 Hz, 9H), 0.58 (q, *J* = 7.9 Hz, 6H).

**$^{13}$C NMR (126 MHz, $CDCl_3$)** δ 8.3, 2.5.

**hydrosilylation of S-1 with $DSiEt_3$ with [Cp*Ru(MeCN)$_3$]PF$_6$**

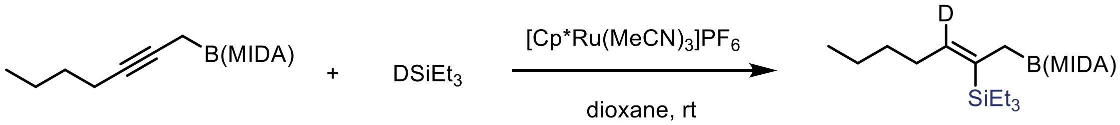


**S-1**

**3-*d***, 95%
*Z/E* > 20:1, r.r. > 20:1

In a glove box, to an oven-dried 15 mL vial were added **S-1** (0.10 mmol), $DSiEt_3$ (0.20 mmol), [Cp*Ru(MeCN)$_3$]PF$_6$ (2.5 mg, 5.0 μmol) and 1,4-dioxane (1.0 mL). The vial was capped and removed from the glove box. The reaction mixture was stirred at room temperature for 30 min, and then concentrated under reduced pressure. The residue was purified by silica gel flash column chromatography (eluent = ethyl acetate/petroleum ether v/v 2:1) to afford **3-*d*** in 95% yield (34.9 mg, D > 99%) as a colorless oil.

**$^1$H NMR (400 MHz, Acetone-*d*$_6$)** δ 4.13 (d, *J* = 16.8 Hz, 2H), 3.97 (d, *J* = 16.8 Hz, 2H), 3.08 (s, 3H), 2.12 (t, *J* = 6.9 Hz, 2H), 1.58 (s, 2H), 1.40 – 1.28 (m, 4H), 0.96 (t, *J* = 7.9 Hz, 9H), 0.94 – 0.87 (m, 3H), 0.73 (q, *J* = 8.0 Hz, 6H).

**$^{13}$C NMR (126 MHz, Acetone-*d*$_6$)** δ 168.7, 143.8 (t, *J* = 21.8 Hz), 133.5, 62.5, 46.1,

33.2, 32.8, 23.2, 14.4, 8.1, 5.1.

**ESI-MS**: calculated for $C_{18}H_{33}DBNNaO_4Si^+$ [M+Na]$^+$, 391.2305; Found, 391.2295.

### hydrosilylation of S-1 with DSiEt3 with CpRu(PPh3)2Cl

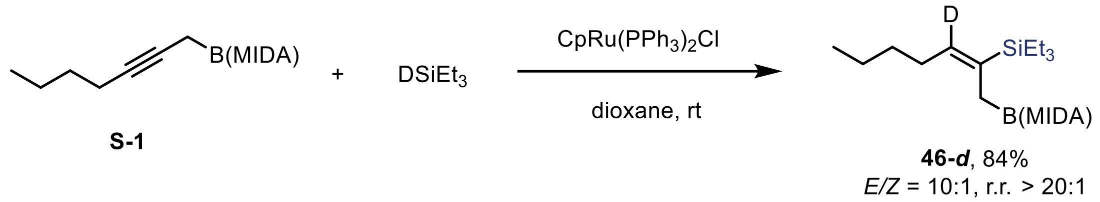


In a glove box, to an oven-dried 15 mL vial were added **S-1** (0.10 mmol), $DSiEt_3$ (0.20 mmol), $CpRu(PPh_3)_2Cl$ (3.6 mg, 5.0 µmol) and 1,4-dioxane (1.0 mL). The vial was capped and removed from the glove box. The reaction mixture was stirred at room temperature for 3 h, and then concentrated under reduced pressure. The residue was purified by silica gel flash column chromatography (eluent = ethyl acetate/petroleum ether v/v 2:1) to afford **3-*d*** in 84% yield (31.0 mg, D > 99%) as a colorless oil.

**$^1$H NMR (500 MHz, Acetone-*d*6)** δ 4.16 (d, $J$ = 16.8 Hz, 2H), 4.05 (d, $J$ = 16.8 Hz, 2H), 3.12 (s, 3H), 2.15 (t, $J$ = 7.0 Hz, 2H), 1.66 (s, 2H), 0.94 – 0.87 (m, 12H), 0.65 (q, $J$ = 7.9 Hz, 6H).

**$^{13}$C NMR (126 MHz, Acetone-*d*6)** δ 168.7, 141.2 (t, $J$ = 23.2 Hz), 134.3, 62.4, 46.0, 32.5, 29.2, 23.2, 14.4, 7.9, 3.8.

**ESI-MS**: calculated for $C_{18}H_{33}DBNNaO_4Si^+$ [M+Na]$^+$, 391.2305; Found, 391.2298.

### 5.6 intermolecular KIE experiment

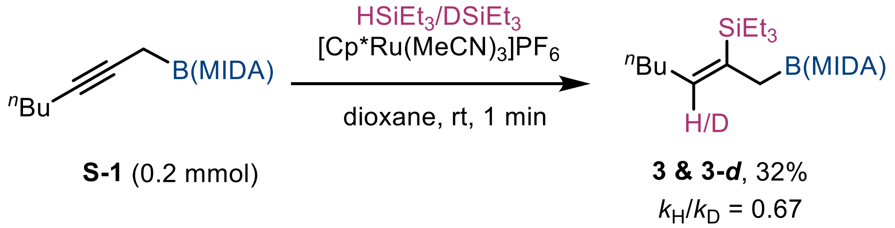


In a glove box, to an oven-dried 15 mL vial were added **S-1** (0.20 mmol), $[Cp^*Ru(MeCN)_3]PF_6$ (5.0 mg, 10.0 µmol) and 1,4-dioxane (2.0 mL). The vial was capped and removed from the glove box. $HSiEt_3/DSiEt_3$ (1:1 mixture, $HSiEt_3$ 11.6 mg, $DSiEt_3$ 11.7 mg) was then injected to the vial by microsyringe. The reaction mixture

was stirred at room temperature for 1 min, and then filtrated through a short pad of silica gel quickly. The residue was directly monitored by $^1$H NMR. The product **3 & 3-*d*** was observed in 32% yield (60% D, see Supplementary Figure 5, $k_H/k_D$ = 0.67).

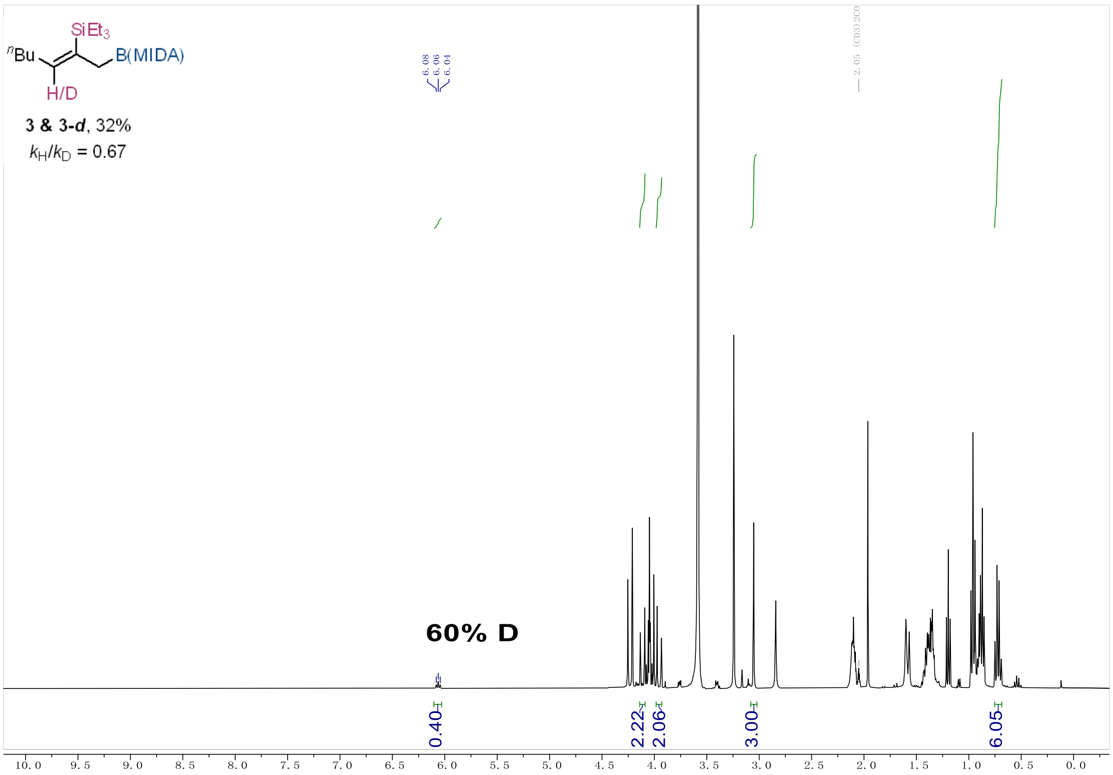


**Supplementary Figure 5: $^1$H NMR of mixture of 3 & 3-*d*. (400 MHz, Acetone-*d*$_6$)** δ 6.06 (t, *J* = 7.5 Hz, 0.4 H), 4.11 (d, *J* = 16.8 Hz, 2H), 3.95 (d, *J* = 16.8 Hz, 22H), 3.05 (s, 3H), 0.72 (q, *J* = 7.5 Hz, 6H).

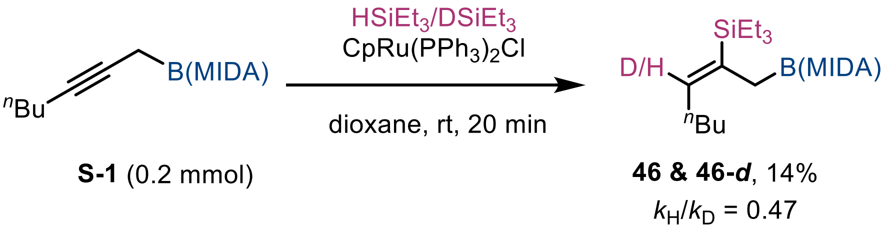


In a glove box, to an oven-dried 15 mL vial were added **S-1** (0.20 mmol), $CpRu(PPh_3)_2Cl$ (7.2 mg, 10.0 μmol) and 1,4-dioxane (2.0 mL). The vial was capped and removed from the glove box. $HSiEt_3/DSiEt_3$ (1:1 mixture, $HSiEt_3$ 11.6 mg, $DSiEt_3$ 11.7 mg) was then injected to the vial by microsyringe. The reaction mixture was stirred at room temperature for 20 min, and then filtrated through a short pad of silica gel quickly. The residue was directly monitored by $^1$H NMR. The product **46 & 46-*d*** was

observed in 14% yield (68% D, see Supplementary Figure 6, $k_H/k_D = 0.47$).

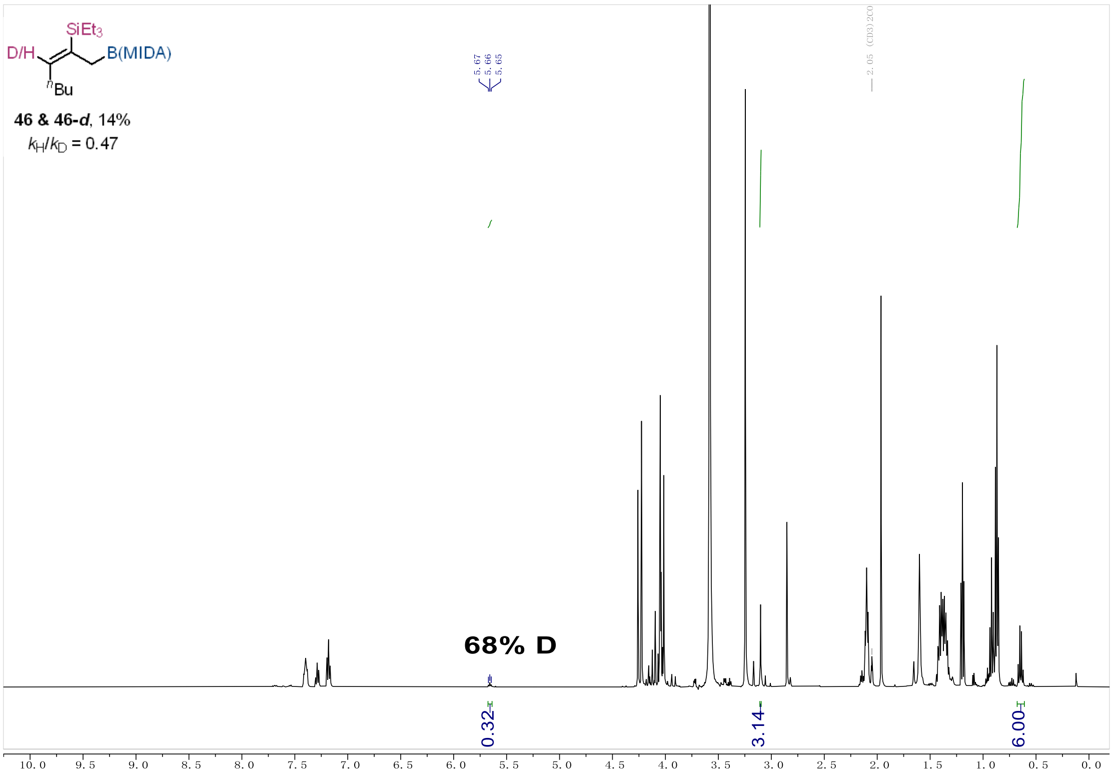


**Supplementary Figure 6: $^1$H NMR of mixture of 46 & 46-*d*. (500 MHz, Acetone-*d*$_6$)**

δ 5.66 (t, $J$ = 6.6 Hz, 1H), 3.10 (s, 8H), 0.65 (q, $J$ = 7.9 Hz, 13H).

### 5.6 parallel KIE experiment

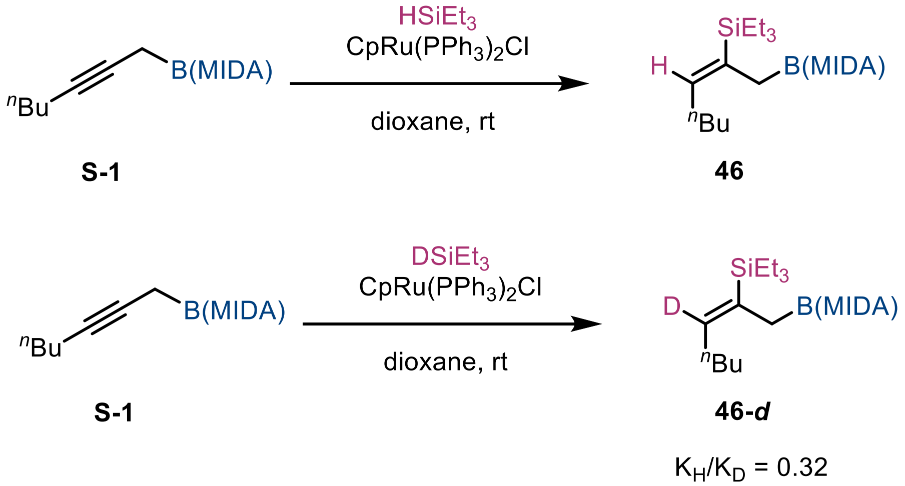


In the glove box, to an oven-dried 15 mL vial were added the propargylic MIDA boronate **S-1** (125.5 mg, 0.50 mmol), $CpRu(PPh_3)_2Cl$ (18.1 mg, 5 mol%), and 1,4-dioxane (5.0 mL). The vial was capped and removed from the glove box. $HSiEt_3$ (58.0 mg, 0.5 mmol) was then injected to the vial by microsyringe. The reaction was started

immediately at room temperature, and 1 mL of reaction solution was taken and quenched by silica gel at reaction times of 15 min, 30 min, 45 min, 60 min, and 75 min. The quenched reaction solution was then concentrated and monitored by $^1$H NMR. The yields are determined by crude $^1$H NMR with $CH_2Br_2$ as the internal standard.

In the glove box, to an oven-dried 15 mL vial were added the propargylic MIDA boronate **S-1** (125.5 mg, 0.50 mmol), $CpRu(PPh_3)_2Cl$ (18.1 mg, 5 mol%), and 1,4-dioxane (5.0 mL). The vial was capped and removed from the glove box. $DSiEt_3$ (58.5 mg, 0.5 mmol) was then injected to the vial by microsyringe. The reaction was started immediately at room temperature, and 1 mL of reaction solution was taken and quenched by silica gel at reaction times of 15 min, 30 min, 45 min, 60 min, and 75 min. The quenched reaction solution was then concentrated and monitored by $^1$H NMR. The yields are determined by crude $^1$H NMR with $CH_2Br_2$ as the internal standard.

The results were listed as following. The KIE was determined to be 0.32 using the equation: KIE = $k_H/k_D$.(see Supplementary Table 1 & Supplementary Figure 7)

| time (min) | **46** yield (%) | **46-*d*** yield (%) |
|---|---|---|
| 15 | 5.6 | 0 |
| 30 | 12.5 | 22 |
| 45 | 16 | 36 |
| 60 | 22.5 | 54 |
| 75 | 27 | 66 |

**Supplementary Table 1: parallel KIE experiment.**

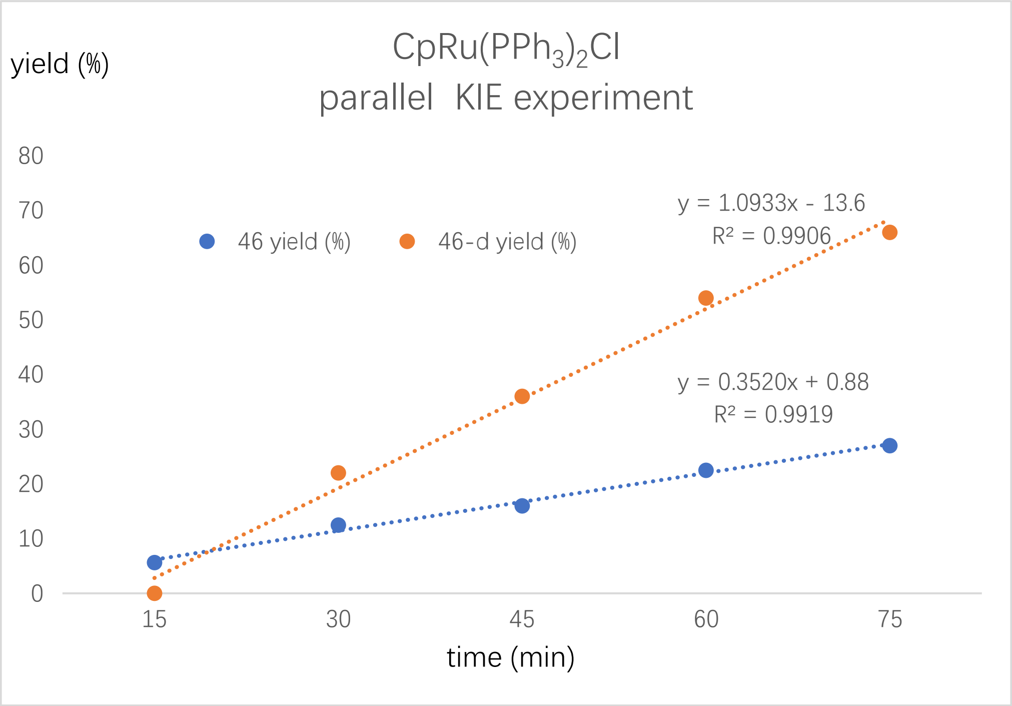


**Supplementary Figure 7: parallel KIE experiment.**

# 6. Computational Studies

## 6.1 Computational Details

The DFT calculations were performed by Gaussian 09 programs[5]. Geometry optimizations and frequency analyses were performed at the level of the B3LYP functional[6] and a standard 6-31G(d) basis set (Lanl2dz[7] for Ru) in gas phase. Single point energies were further refined at the M06 functional[8] and a standard 6-311++G(d,p) basis set (SDD[9] for Ru) level using PCM solvation model[10] (the Cp*Ru(MeCN)$_3$PF$_6$ catalyzed system, solvent = THF; the CpRu(PPh$_3$)$_2$Cl catalyzed system, solvent = 1,4-dioxane). Throughout the paper, the energies presented are the M06-calculated Gibbs free energies in the THF/1,4-dioxane solvent with B3LYP-calculated thermodynamic corrections. After optimizing the structure, we obtained the wavefuction, and the electronic structure analysis was performed with Multiwfn[11].

The Born–Oppenheimer molecular dynamics simulations[12-14] were performed with the "BOMD" keyword implemented in Gaussian 09 package. Based on the

transition states **TS-1β** and **$^{Cp}$TS-1β** optimized at B3LYP/6-31G(d) (Lanl2dz for Ru) level of theory, 100 trajectories from each transition state were initiated with their initial coordinates and momenta generated from normal mode sampling at the default temperature (**298.15 K**). All the calculated structures were visualized using CYLview[15] or VMD[16].

### 6.2 Computational Discussions

#### a. The role of the $Cl^-$ in the stereochemical outcome

We conducted additional DFT calculations. As illustrated in the Supplementary Figure 8 below, the intermediates **$^{Cp}$INT-2β-syn** and **$^{Cp}$INT-2β-anti** exhibit excellent stereochemical selectivity, with an energy difference of 8.5 kcal/mol. Upon removal of the Cl ligand from **$^{Cp}$INT-2β-syn** and **$^{Cp}$INT-2β-anti**, the resulting isomers, **$^{Cp}$INT-2β-syn-no-Cl** and **$^{Cp}$INT-2β-anti-no-Cl**, exhibit nearly identical energies, differing by only 0.4 kcal/mol, indicating poor selectivity.

The introduction of the Cl ligand increases structural crowding, particularly in **$^{Cp}$INT-2β-anti**. In the absence of the Cl ligand, the distance between the butyl chain carbon and the Si atom in **$^{Cp}$INT-2β-anti-no-Cl** is 3.94 Å. However, with the Cl ligand present, this distance decreases to 3.48 Å in **$^{Cp}$INT-2β-anti**, which is less than the sum of the van der Waals radii of C and Si (3.80 Å). This results in significant steric hindrance, leading to a higher energy for **$^{Cp}$INT-2β-anti**. These results were commented in the main text.

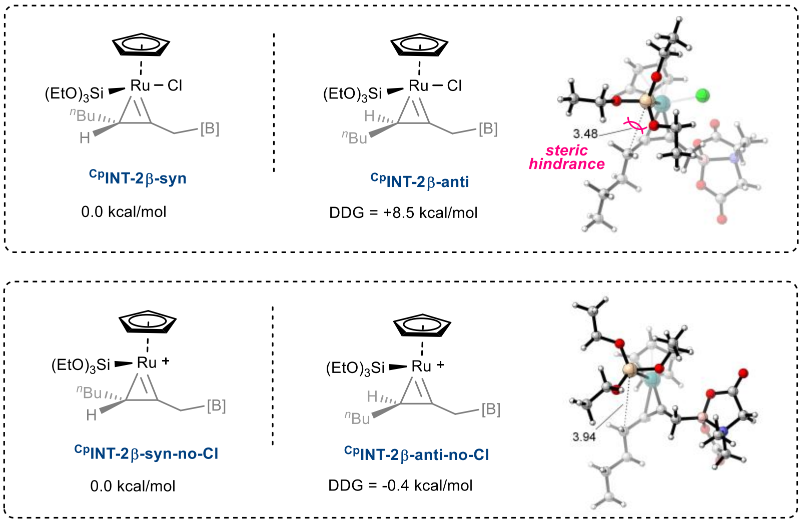

**Supplementary Figure 8. The role of the Cl ligand in the stereochemical outcome**

**b. The activation energy barriers for compounds 59, 61 and 62 by the directing effect of the B(MIDA) moiety or hydrogen bonding**

We conducted DFT calculations on compounds **59**, **61** and **62** (as shown in the Supplementary Figure 9 below), which revealed that the transition states influenced by the directing effect of the B(MIDA) moiety (**59-$^{Cp}$TS-1β**, **61-$^{Cp}$TS-1β**, and **62-$^{Cp}$TS-1β**) exhibit energies 2.7 to 4.8 kcal/mol lower than those by hydrogen bonding (**59-$^{Cp}$TS-1γ**, **61-$^{Cp}$TS-1γ**, and **62-$^{Cp}$TS-1γ**). These results align with the experimentally observed selectivity and further support the presence of the directing effect of the B(MIDA) moiety. The comment of these results was added to the reference part in the manuscript.

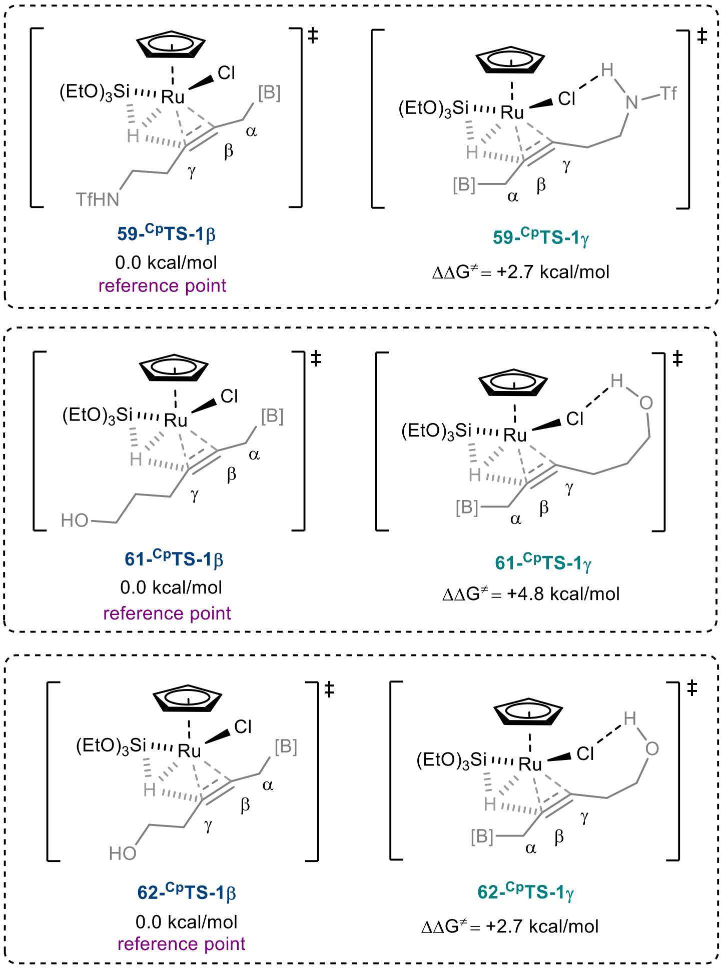


**Supplementary Figure 9. The activation energy barriers for compounds 59, 61 and 62**

### c. The activation energy barriers and free energy for the Bpin analog

As illustrated in the Supplementary Figure 10 below, when replacing B(MIDA) with $sp^2$-B Bpin moiety in transition states **TS-1β** and **TS-1γ**, the energy difference between **Bpin-TS-1β** and **Bpin-TS-1γ** drops from 1.8 kcal/mol to 0.9 kcal/mol, consistent with the experimentally observed reduction in the β/γ ratio to 4:1. Furthermore, compared to the significant energy difference (ΔΔG = 8.2 kcal/mol) between **INT-2β-anti** and **INT-2γ-anti**, the energies of **Bpin-INT-2β-anti** and **Bpin-INT-2γ-anti** are much closer (ΔΔG = 2.0 kcal/mol). Specifically, as the figure shown below, the smaller size and lower steric hindrance of Bpin may allow for greater flexibility in the transition state **Bpin-TS-1γ** (lower steric hindrance), leading to a reduced energy difference between **Bpin-TS-1β** and **Bpin-TS-1γ**. In contrast, the bulkier B(MIDA) group likely imposes stronger steric hindrance (as in **TS-1γ**), favoring the β-product more selectively.

The NBO orbital interaction analysis was also conducted to clarify the reason for this reduced energy difference. Due to the large energy difference between **Bpin-INT-2β-anti** and **Bpin-INT-2γ-anti** (compared to **Bpin-TS-1β** and **Bpin-TS-1γ**), it is inferred that their orbital interactions should also differ significantly. Therefore, we chose to perform NBO orbital interaction analysis on the ruthenium three-membered ring intermediates **Bpin-INT-2β-anti** and **Bpin-INT-2γ-anti**. For the orbital interactions between σ(C–B) and π*(C=Ru), the $E^2$ energy for **Bpin-INT-2β-anti** (6.1 kcal/mol) is lower than that for **INT-2β-anti** (7.6 kcal/mol). Additionally, for the orbital interactions between σ(C–C) and π*(C=Ru), while no $E^2$ value is observed for **INT-2γ-anti**, an $E^2$ energy of 3.4 kcal/mol is present in **Bpin-INT-2γ-anti**. Compared to **INT-2β-anti** ($E^2$ = 7.6 kcal/mol) and **INT-2γ-anti** (no $E^2$ value), the orbital interaction energy difference between **Bpin-INT-2β-anti** ($E^2$ = 6.1 kcal/mol) and **Bpin-INT-2γ-anti** ($E^2$ = 3.4 kcal/mol) is much smaller, which also indicates that B(MIDA) and Bpin exhibit different abilities to influence regioselectivity through σ(C–B) hyperconjugation.

Overall, based on the calculations results above, the significantly reduced β/γ ratio

of 4:1 with Bpin (compared to the higher ratio observed with B(MIDA)) suggests that the steric and electronic properties of the boron substituent play a critical role in determining the regioselectivity of the reaction.

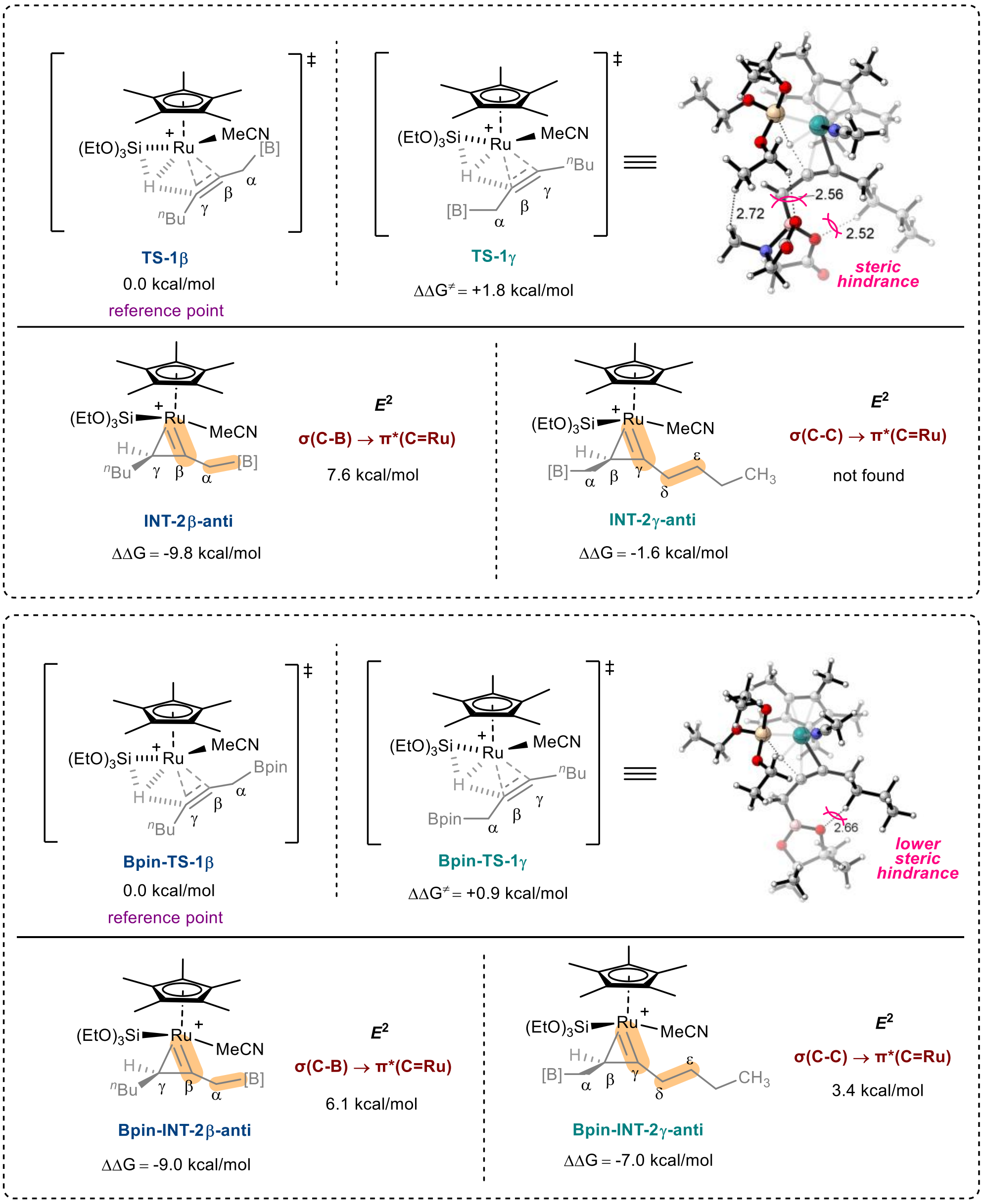


**Supplementary Figure 10. The activation energy barriers and free energy for the Bpin analog**

## d. The isomerization process from INT-2β-anti to INT-2β'-anti

The isomerization process from **INT-2β-anti** to **INT-2β'-anti** is considered. First, the MeCN ligand in **INT-2β-anti** dissociates from the Ru center to form **INT-noLigand**. Then, MeCN recoordinates (**INT-noLigand**) to form **INT-2β'-anti**. The intermediate

**INT-noLigand** has an energy of ΔG = 6.7 kcal/mol relative to **INT-2β-anti** (ΔG = 2.5 kcal/mol), indicating that the process occurs readily.

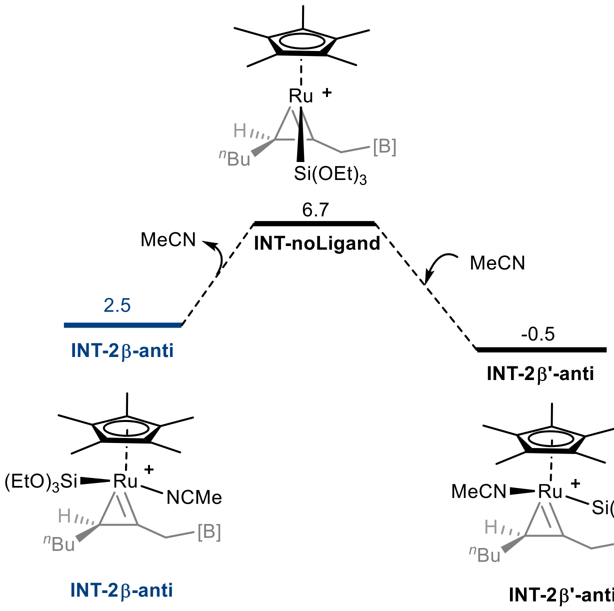


**Supplementary Figure 11. The isomerization process from INT-2β-anti to INT-2β'-anti**

## e. The dissociation energy of different ligands in $CpRu(PPh_3)_2Cl$

Based on additional DFT calculations, we found that in the $CpRu(PPh_3)_2Cl$ catalyst, the dissociation of the Cl ligand from the Ru metal requires a significantly higher energy of 48.2 kcal/mol, whereas the departure of the $PPh_3$ ligand from Ru is much more facile, with an energy of only 11.3 kcal/mol (see the Supplementary Figure 12 below).

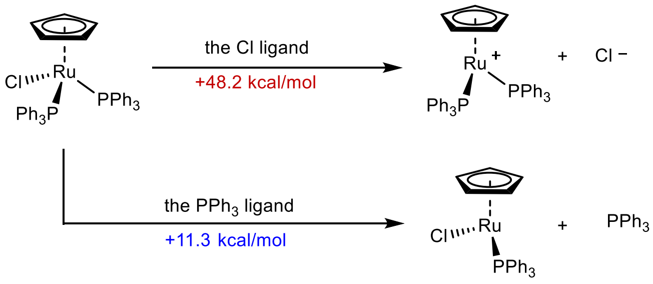


**Supplementary Figure 12. The dissociation energy of different ligands in $CpRu(PPh_3)_2Cl$**

## 7. NMR spectra

### $^{1}$H NMR of S-3 (400 MHz, Acetone-$d_6$)

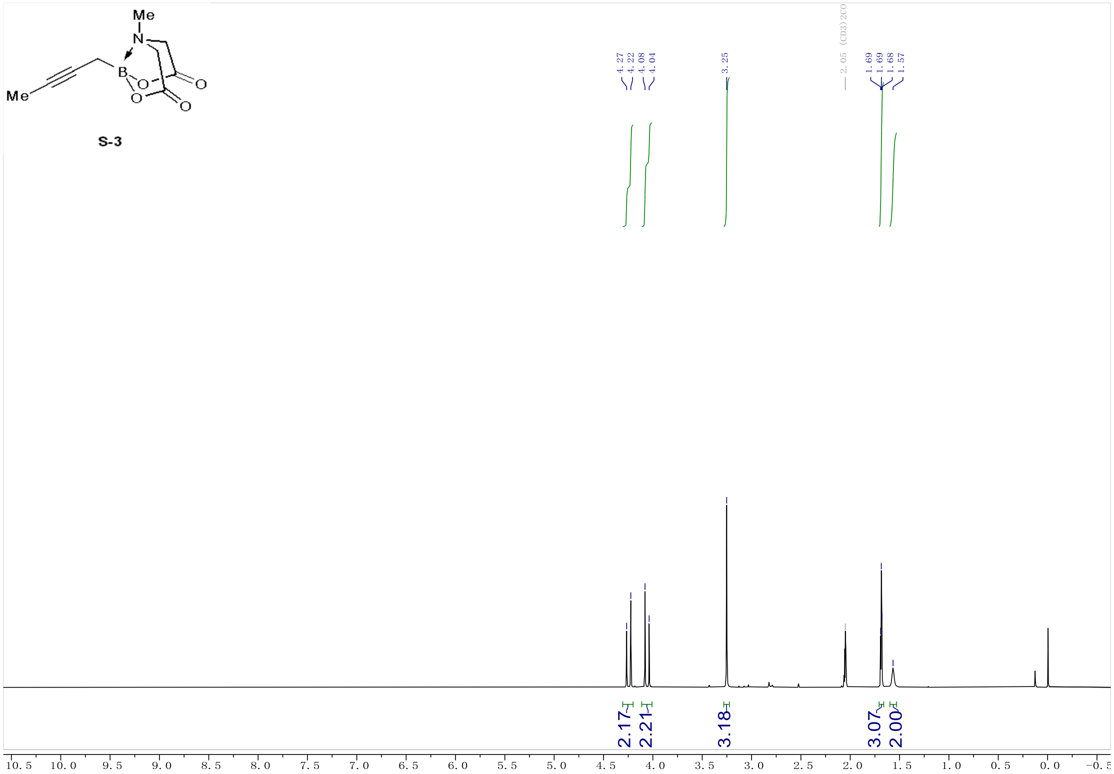

### $^{13}$C NMR of S-3 (101 MHz, Acetone-$d_6$)

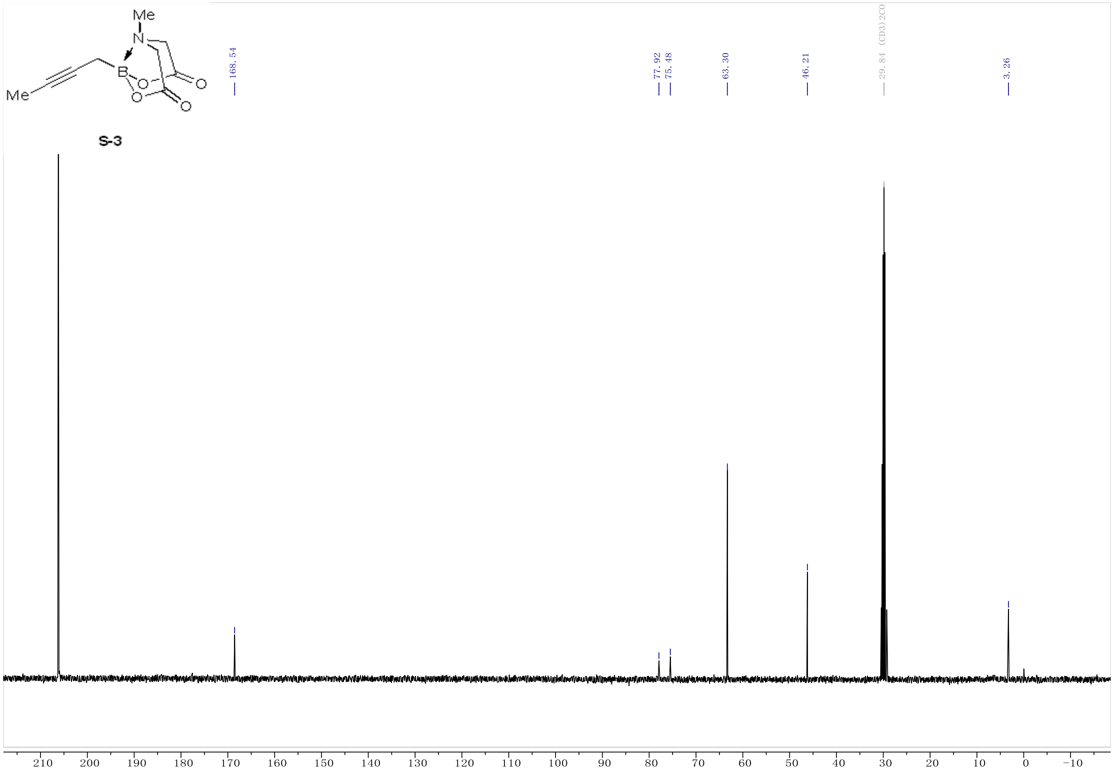


# $^{1}$H NMR of S-6 (400 MHz, DMSO-$d_6$)

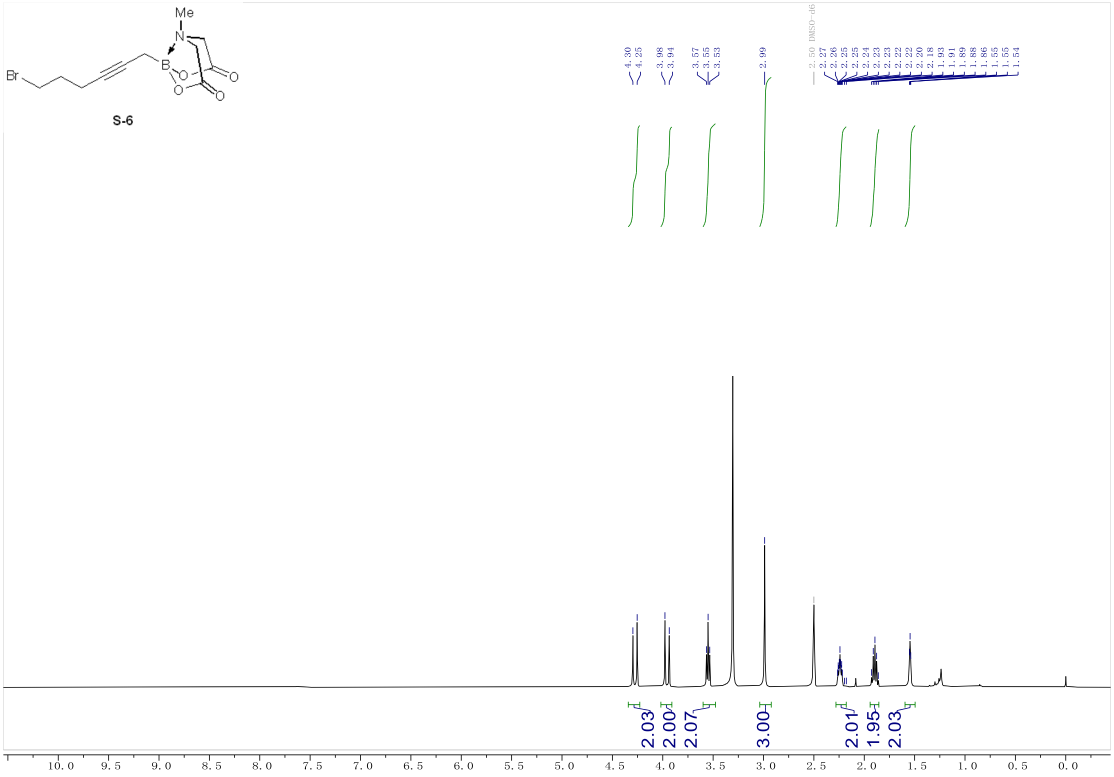

## $^{13}C$ NMR of S-6 (101 MHz, DMSO-$d_6$)

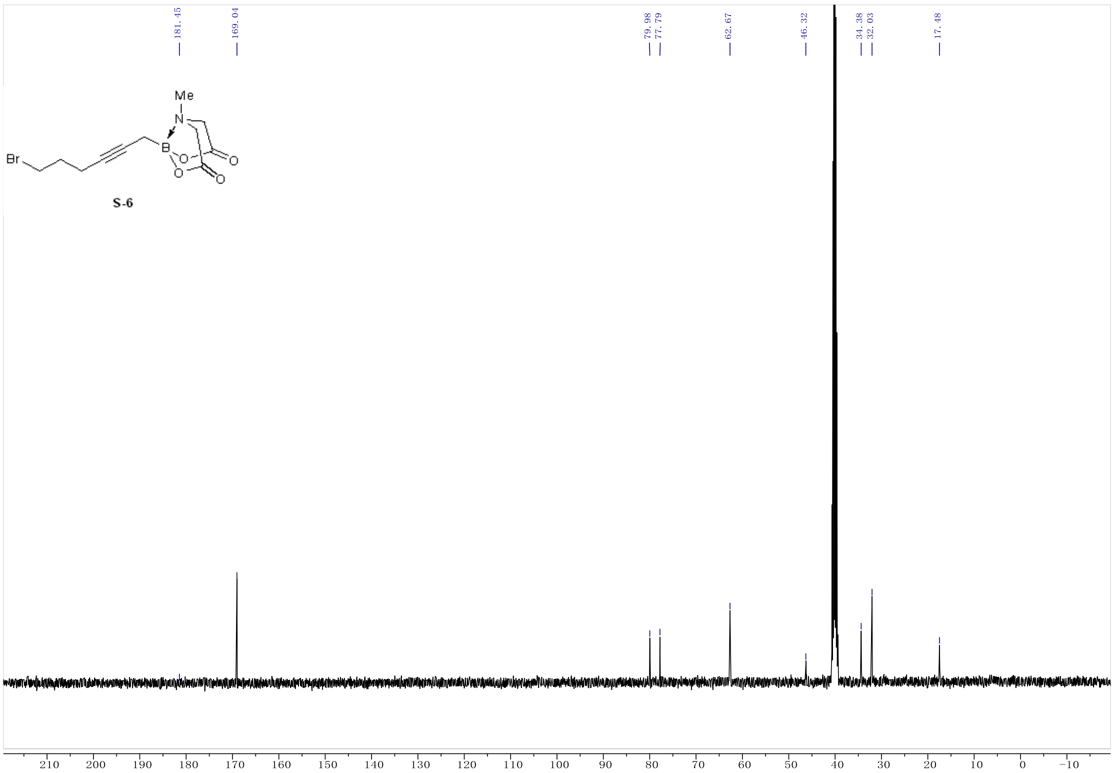

## $^{1}H$ NMR of S-9 (400 MHz, DMSO-$d_6$)

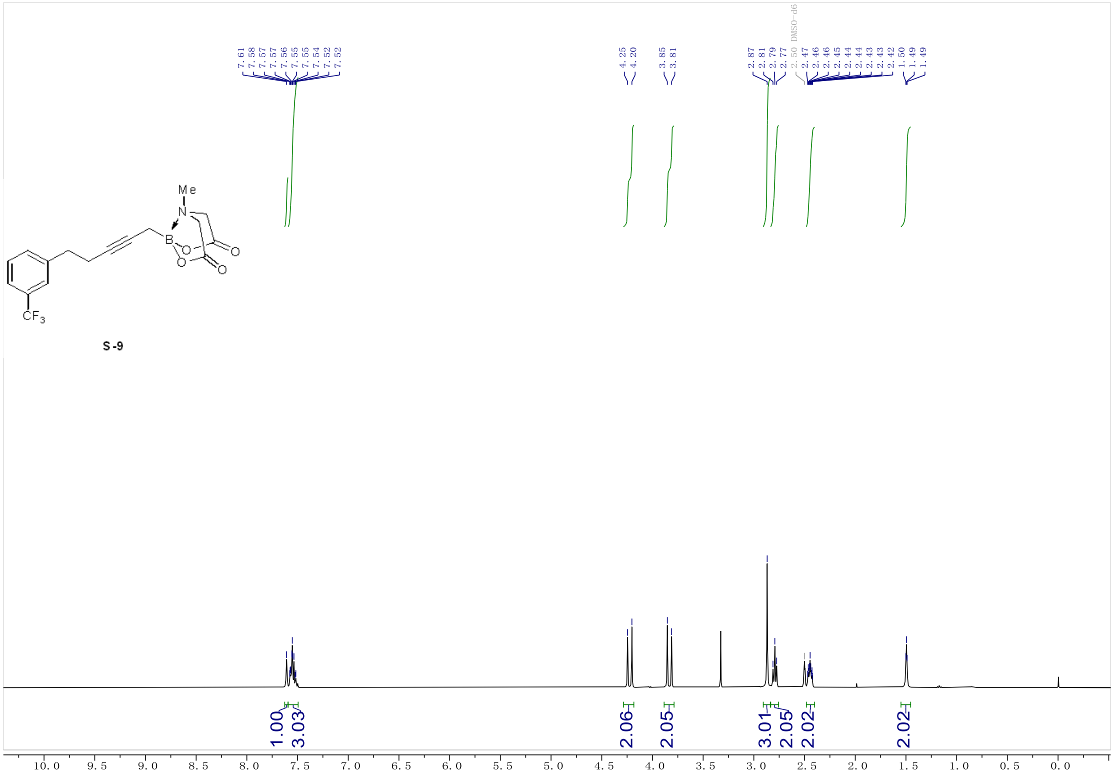

**$^{13}$C NMR of S-9 (101 MHz, DMSO-$d_6$)**

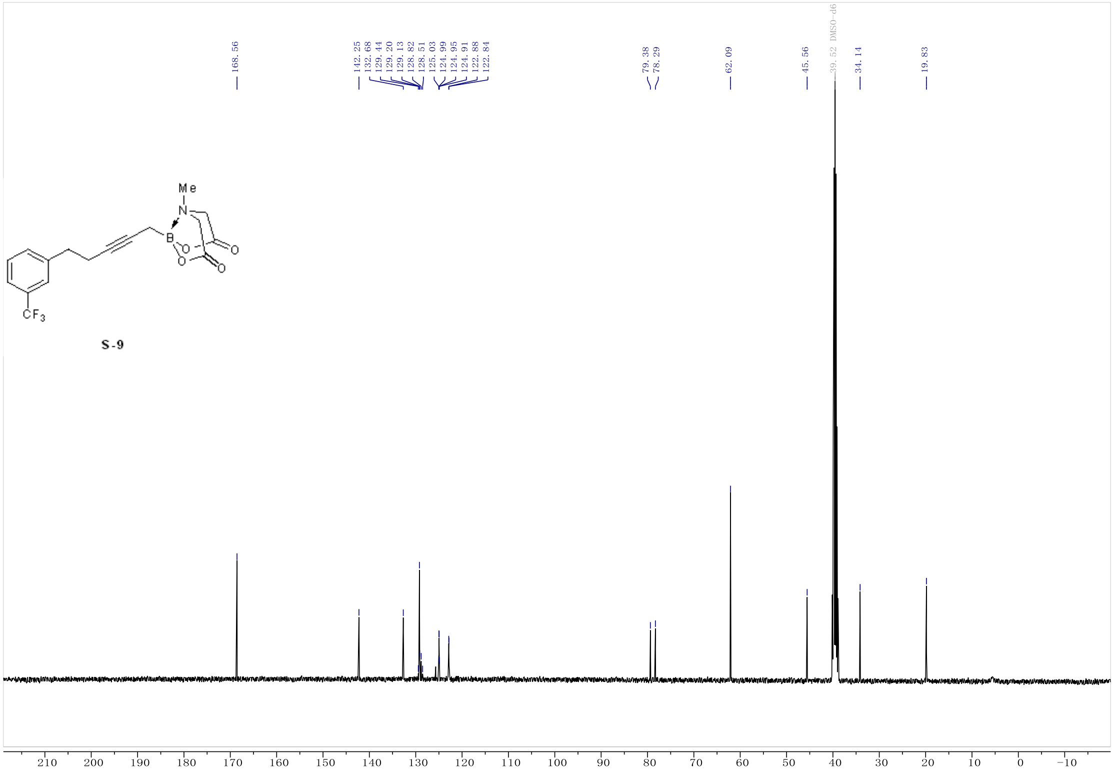


**$^{19}$F NMR of S-9 (376 MHz, DMSO-$d_6$)**

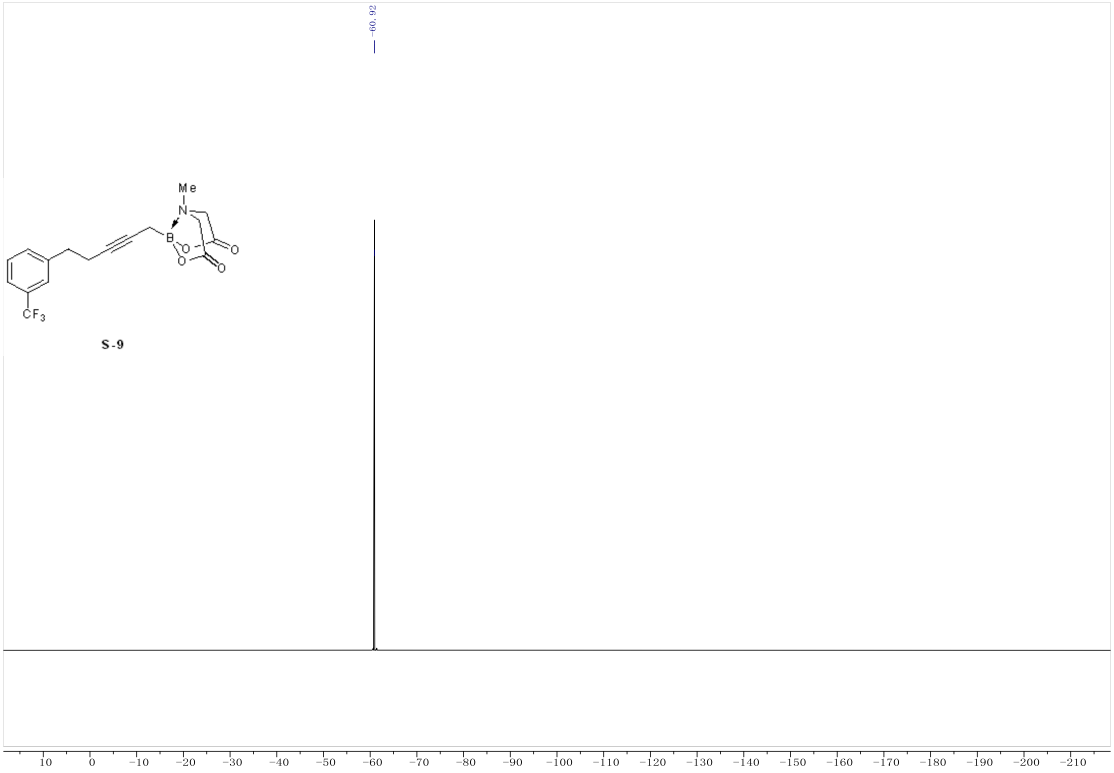

## $^{1}$H NMR of S-12 (400 MHz, Acetone-$d_6$)

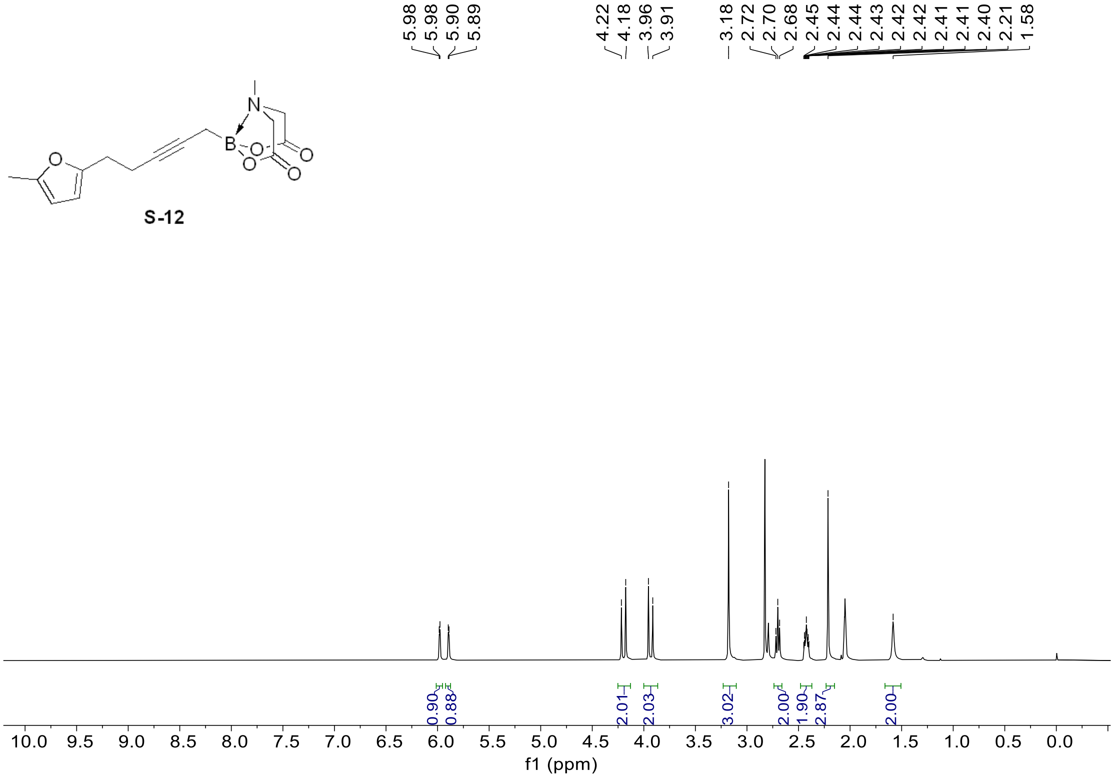


## $^{13}$C NMR of S-12 (101 MHz, Acetone-$d_6$)

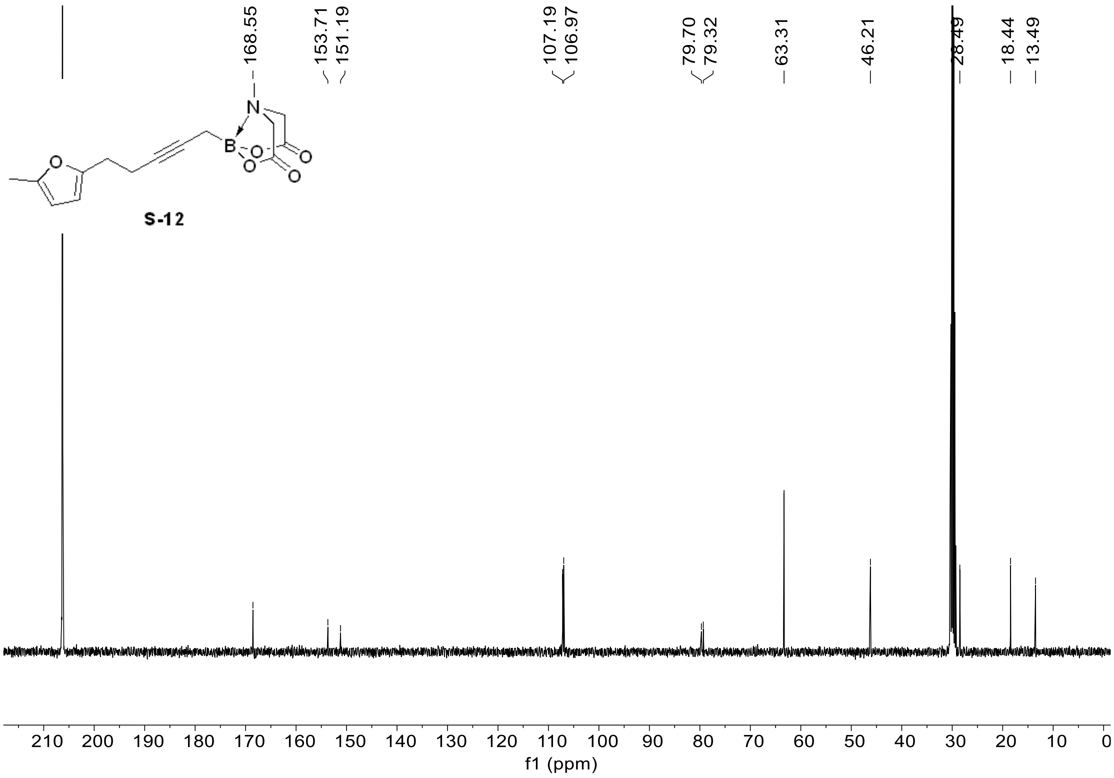

**$^{1}$H NMR of S-15 (400 MHz, DMSO-$d_6$)**

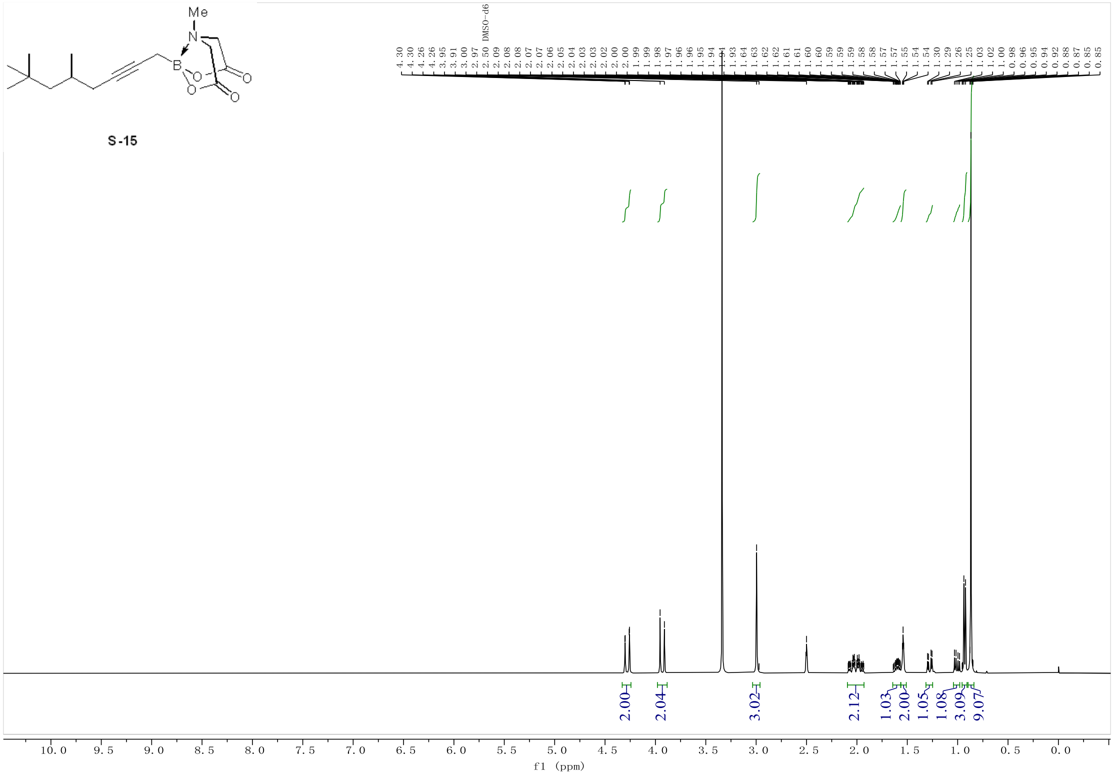

**$^{13}$C NMR of S-15 (101 MHz, DMSO-$d_6$)**

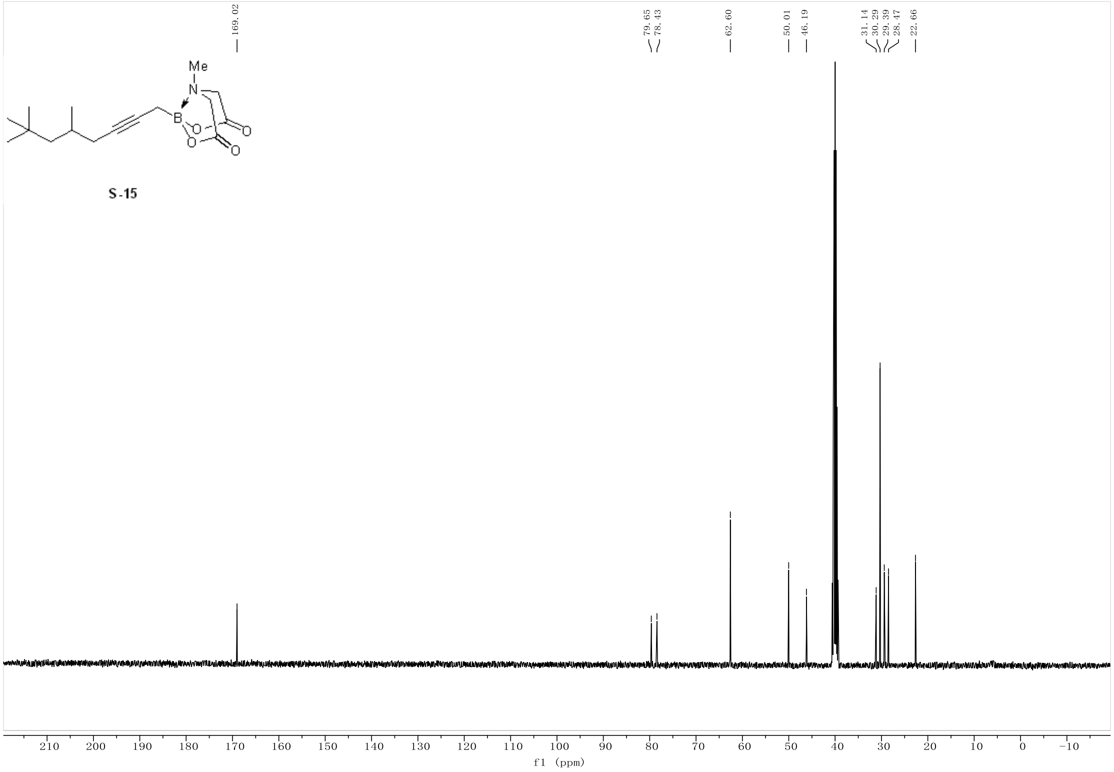

$^{1}$H NMR of S-19 (400 MHz, Acetone-$d_6$)

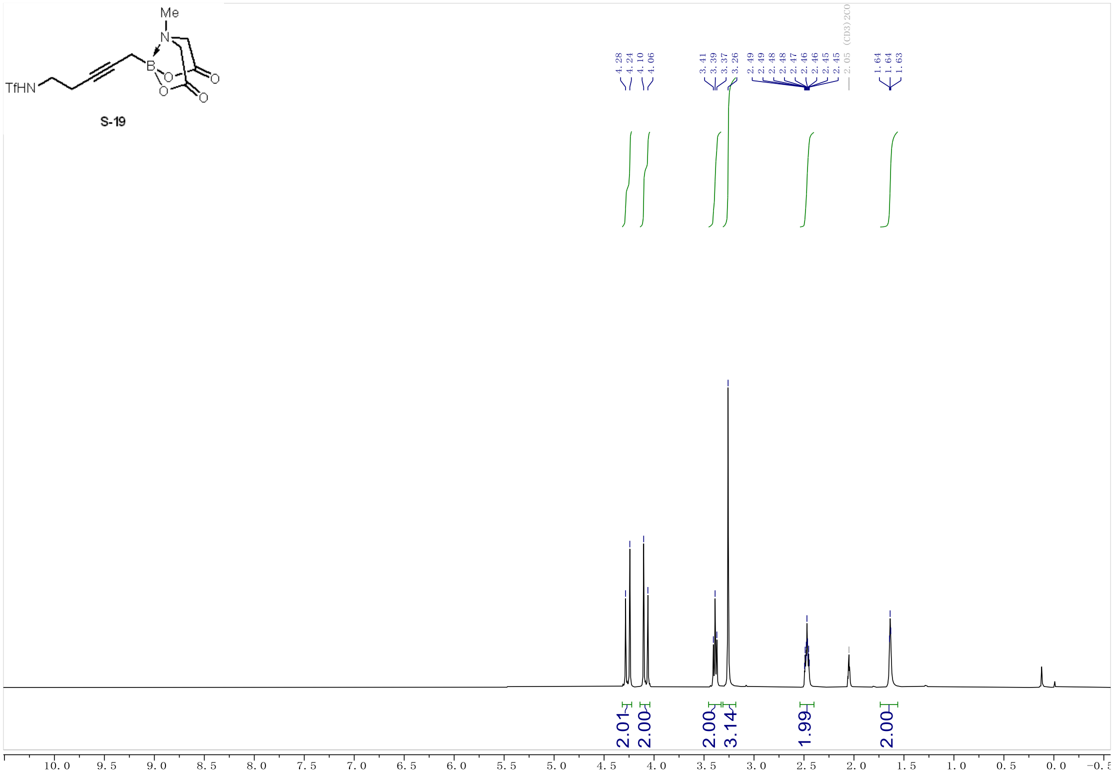

$^{13}$C NMR of S-19 (101 MHz, Acetone-$d_6$)

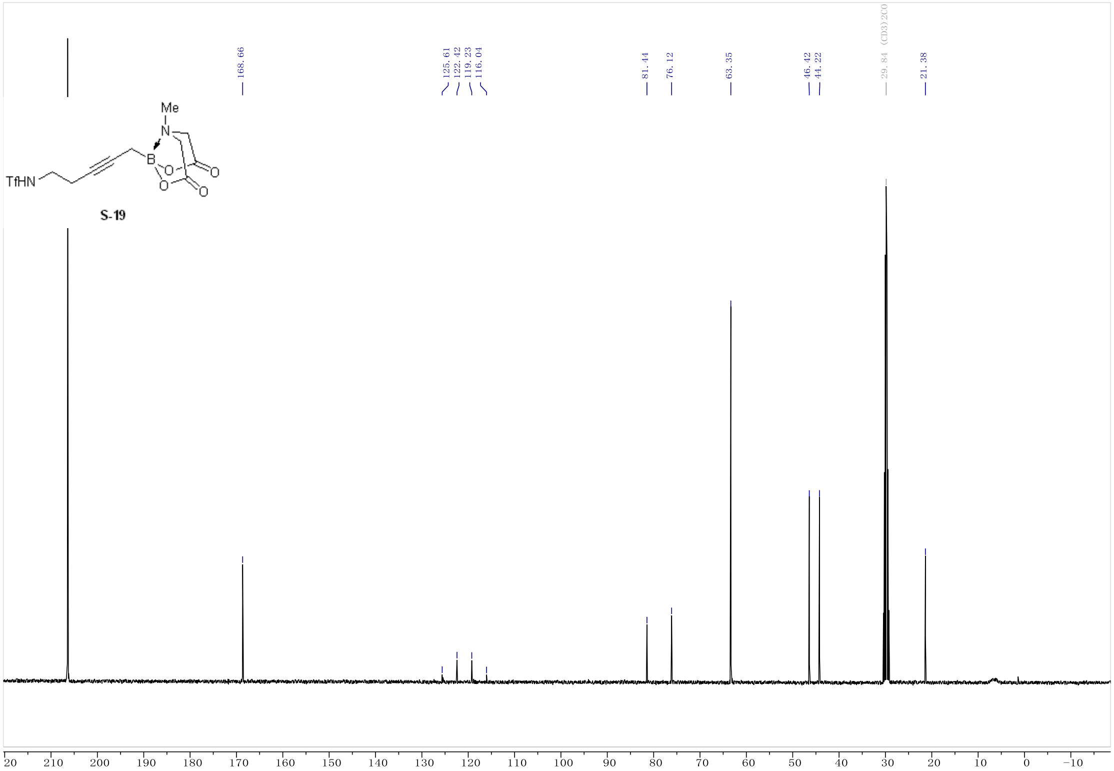

## $^{19}$F NMR of S-19 (376 MHz, Acetone-$d_6$)

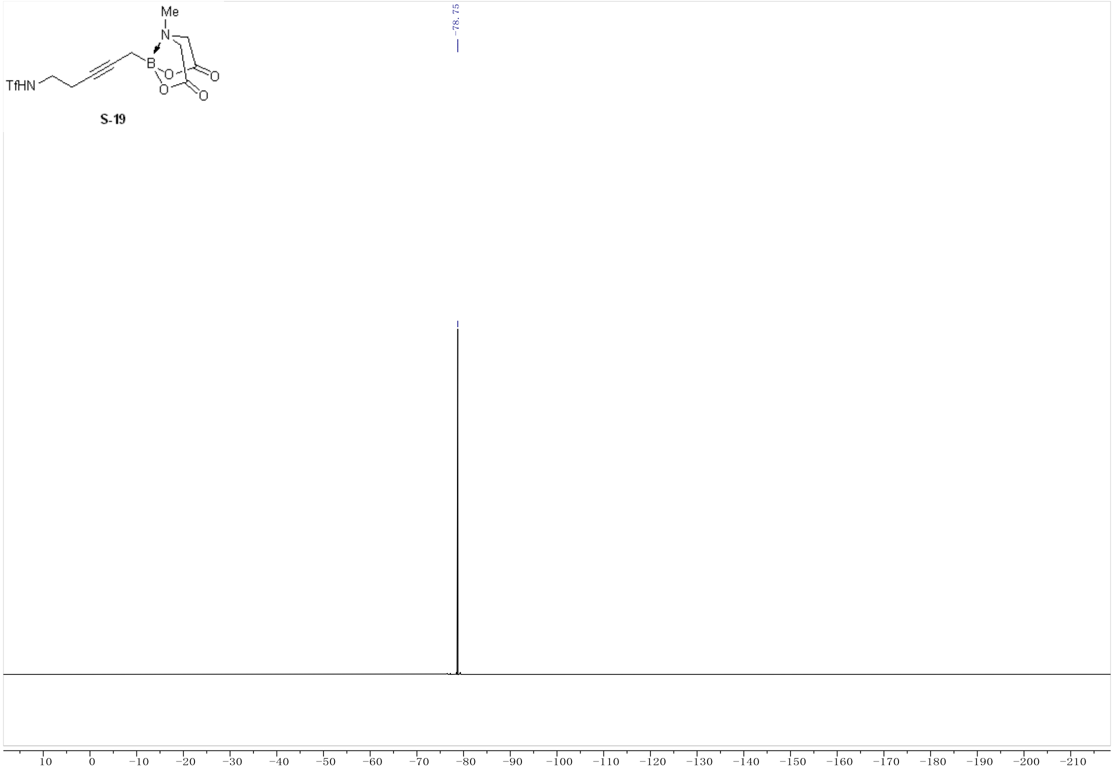


## $^{1}$H NMR of S-22 (400 MHz, $CD_3CN$)

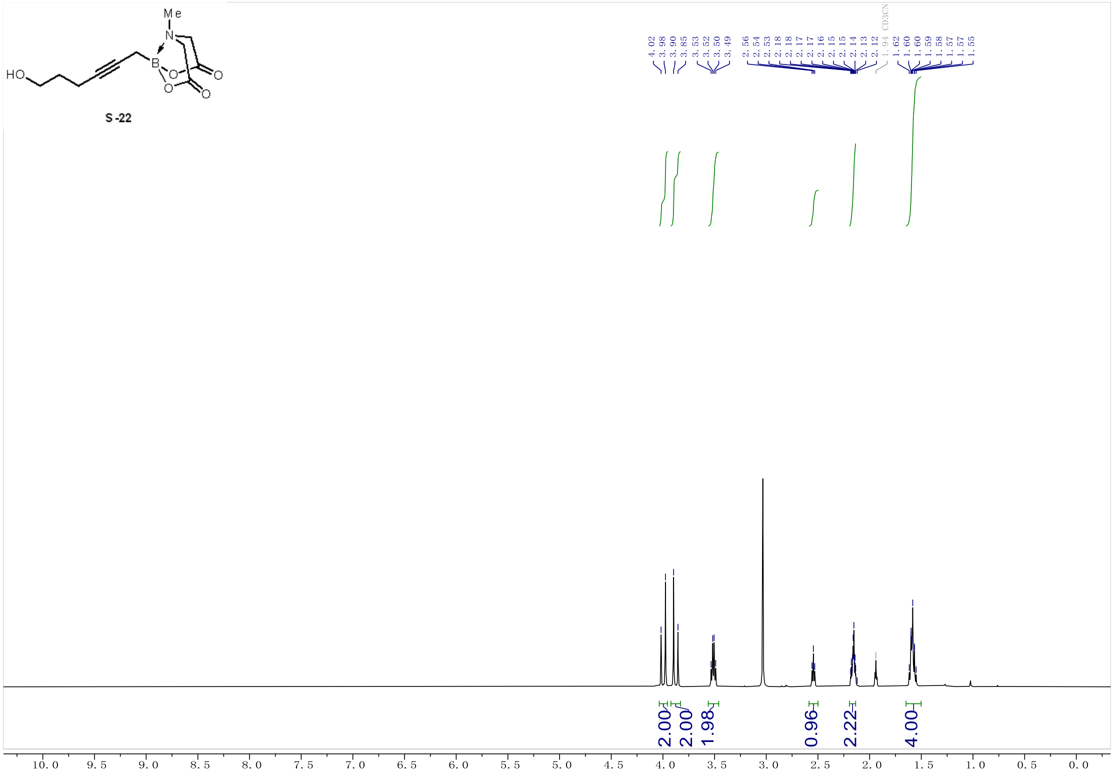

## $^{13}$C NMR of S-22 (101 MHz, $CD_3CN$)

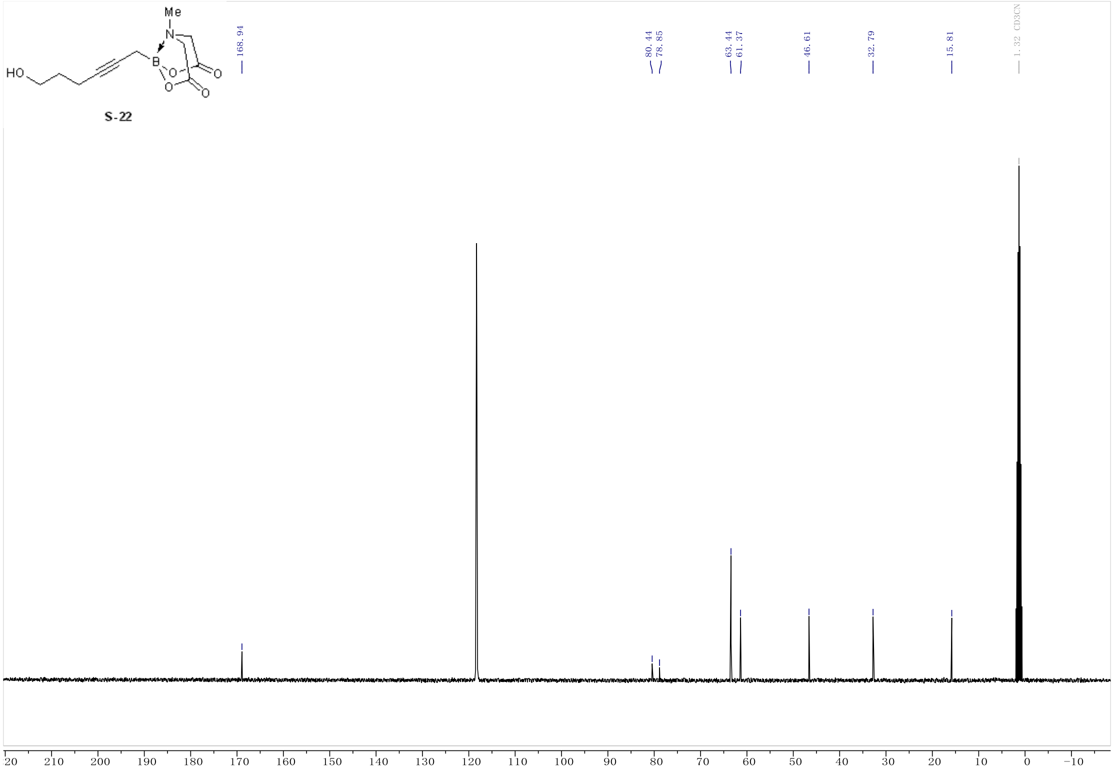


## $^{1}$H NMR of S-23 (500 MHz, $CD_3CN$)

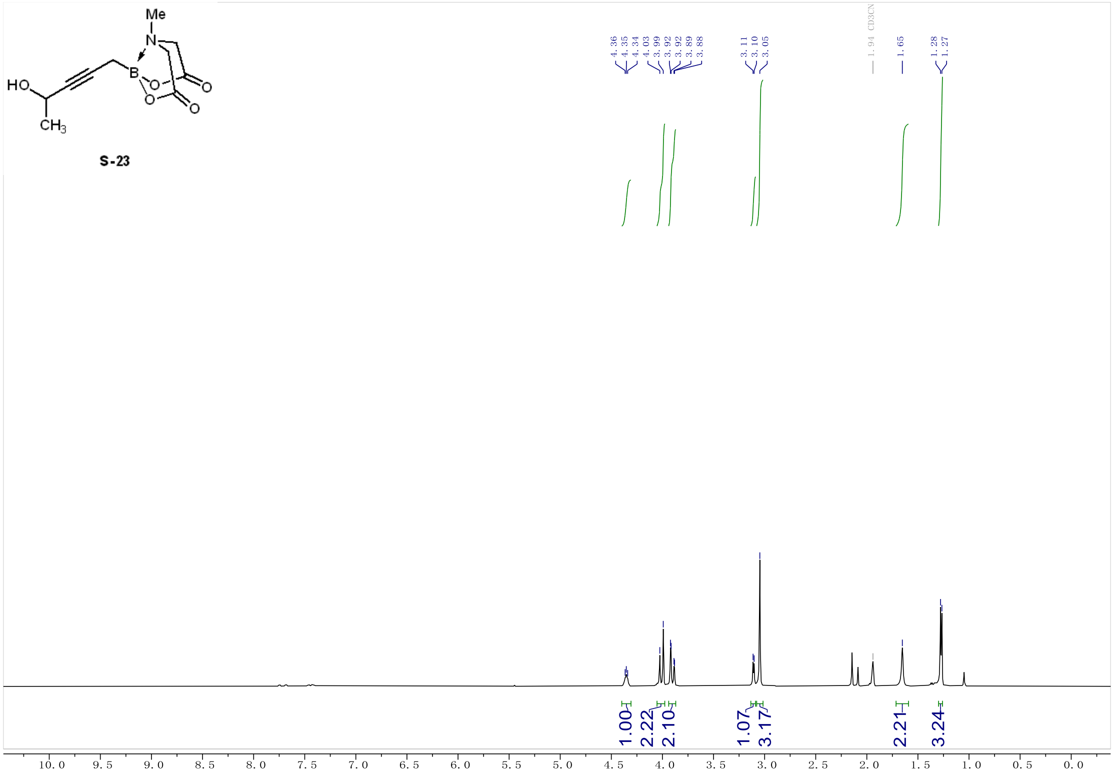

$^{13}C$ NMR of S-23 (126 MHz, $CD_3CN$)

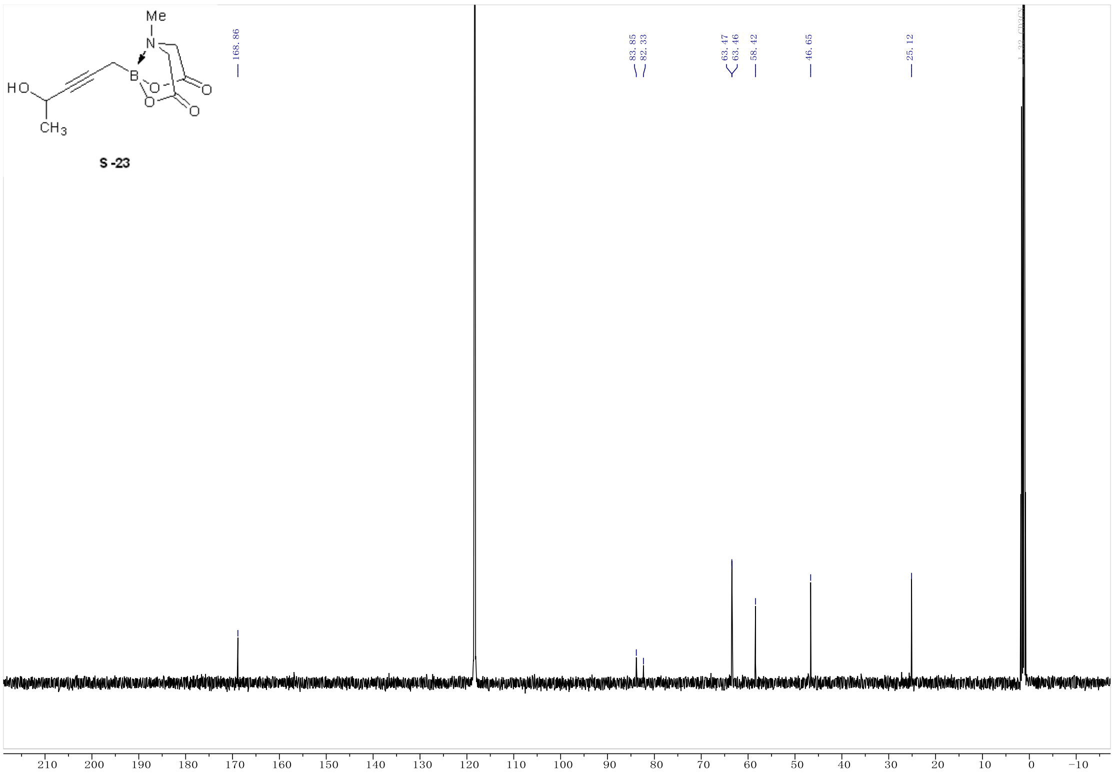


$^{1}H$ NMR of S-24 (400 MHz, Acetone-$d_6$)

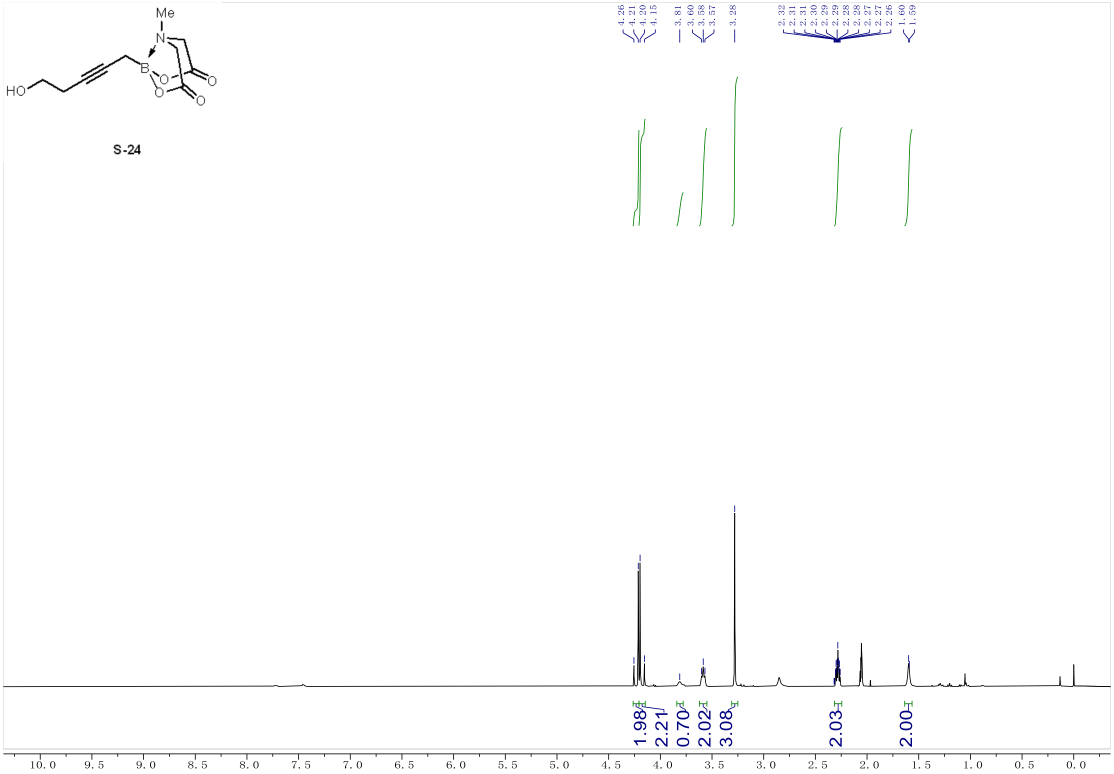

$^{13}$C NMR of S-24 (101 MHz, Acetone-$d_6$)

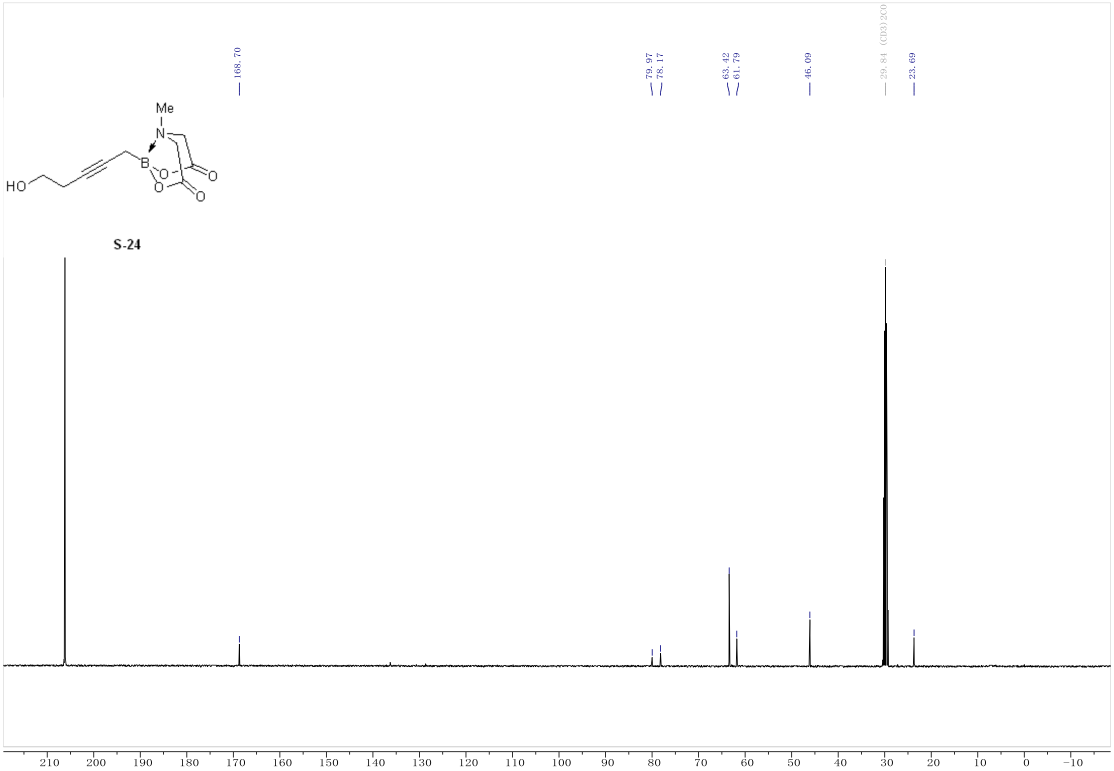

S-24

$^{1}$H NMR of S-26 (500 MHz, DMSO-$d_6$)

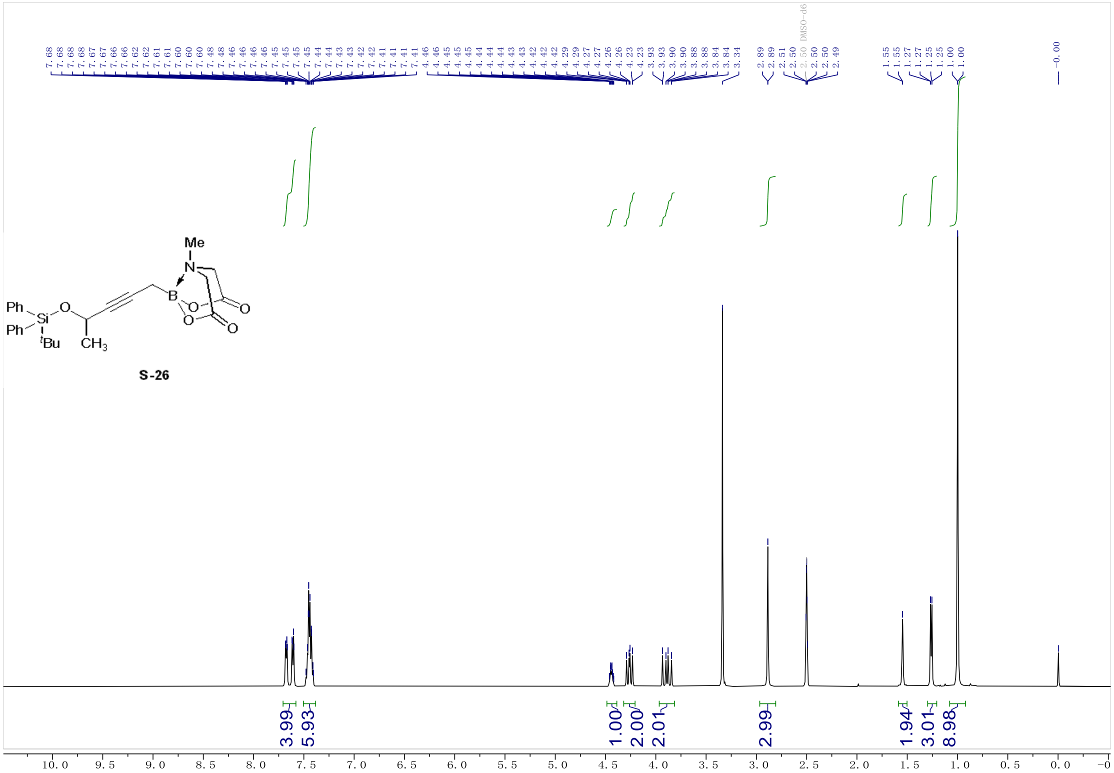

S-26

## $^{13}$C NMR of S-26 (126 MHz, DMSO-$d_6$)

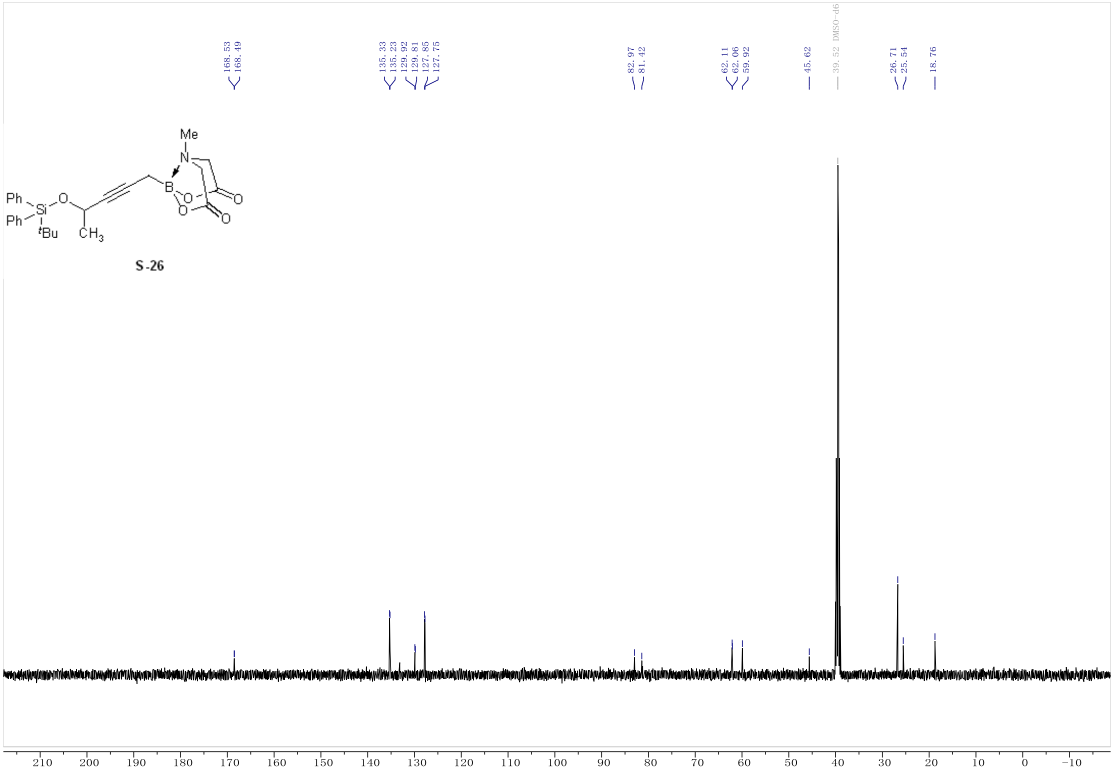


## $^{1}$H NMR of S-29 (400 MHz, DMSO-$d_6$)

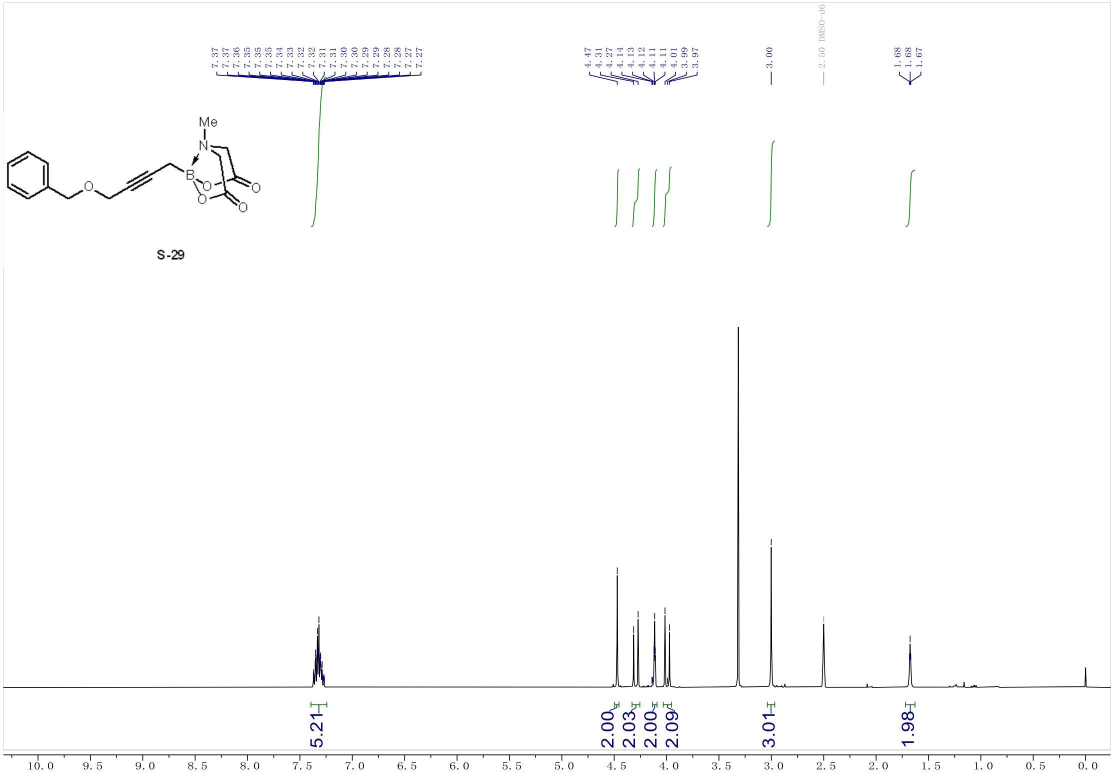

## $^{13}$C NMR of S-29 (101 MHz, DMSO-$d_6$)

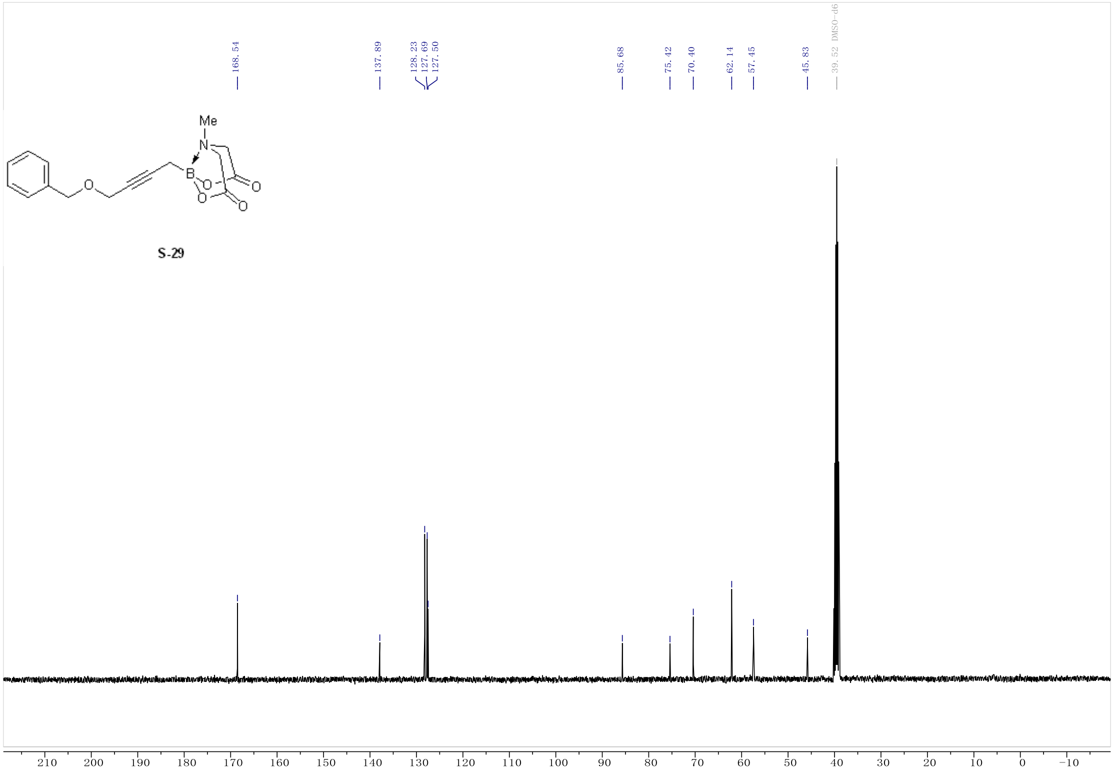


## $^{1}$H NMR of S-30 (400 MHz, DMSO-$d_6$)

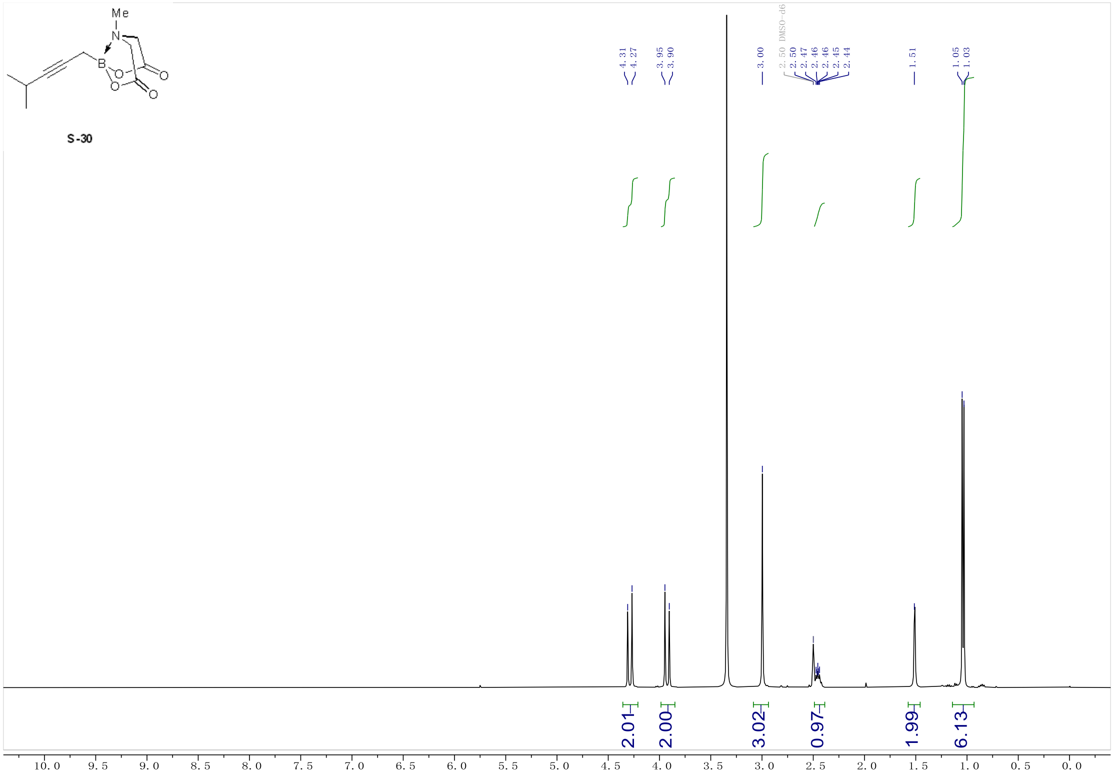

## $^{13}C$ NMR of S-30 (101 MHz, DMSO-$d_6$)

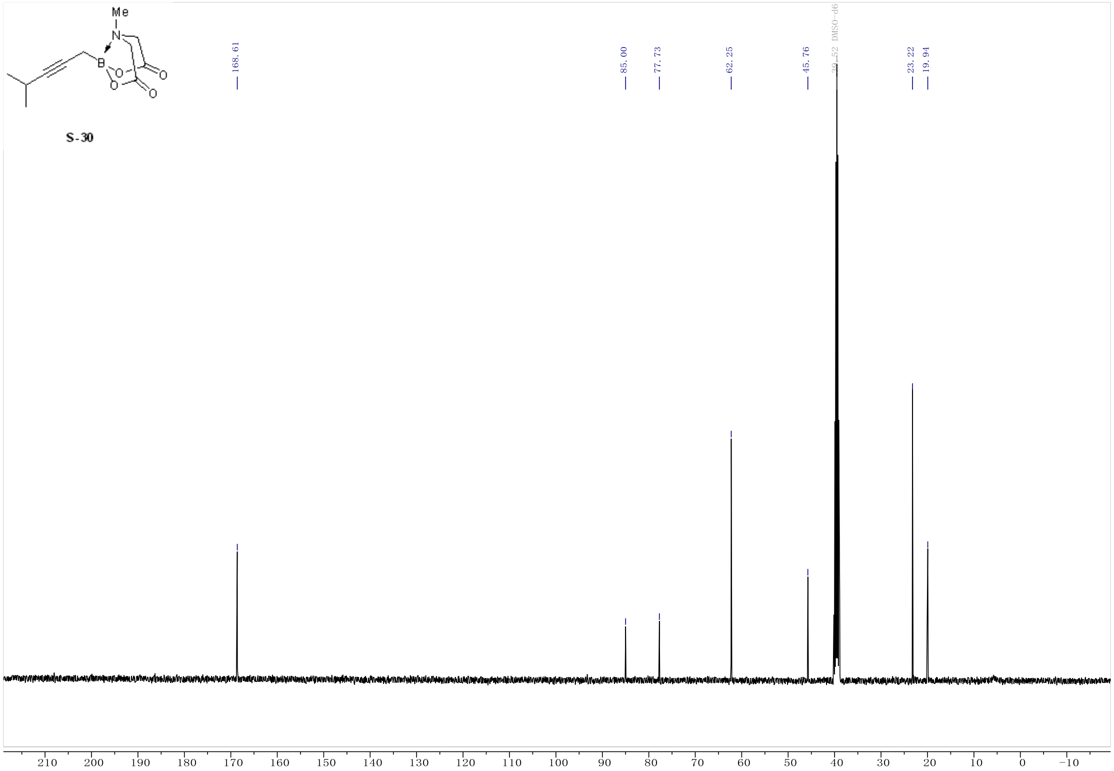

## $^{1}H$ NMR of S-31 (400 MHz, Acetone-$d_6$)

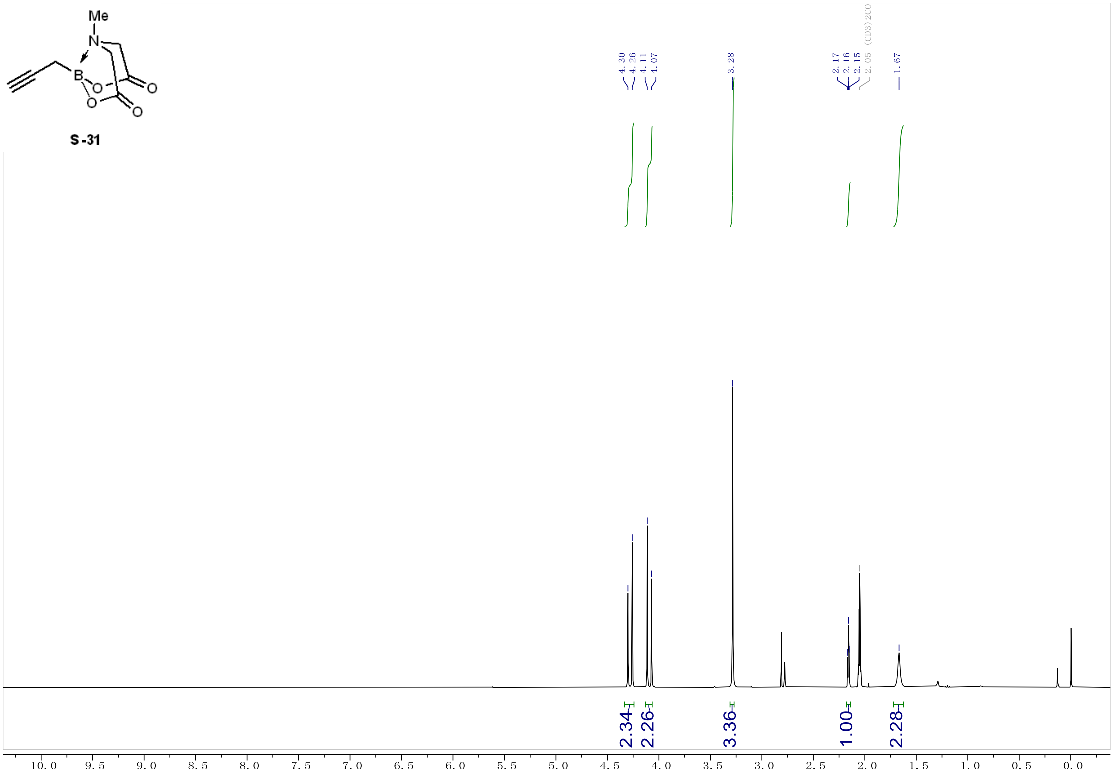

## $^{13}$C NMR of S-31 (101 MHz, Acetone-$d_6$)

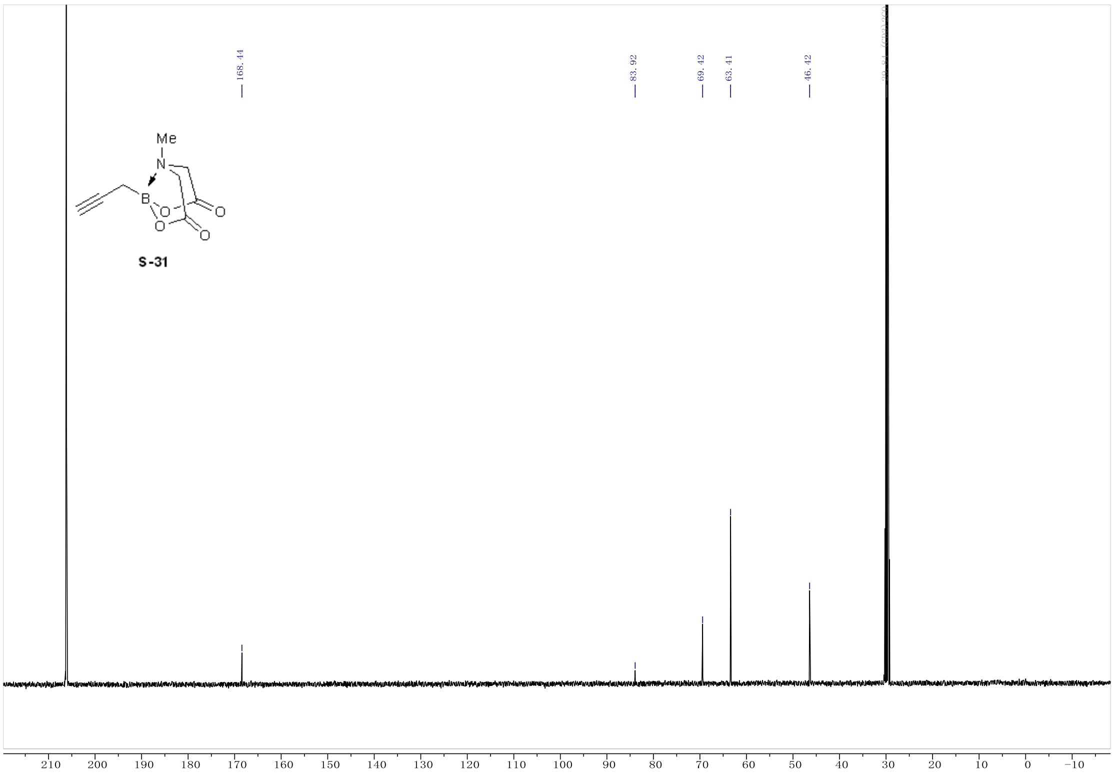


## $^{1}$H NMR of S-32 (500 MHz, DMSO-$d_6$)

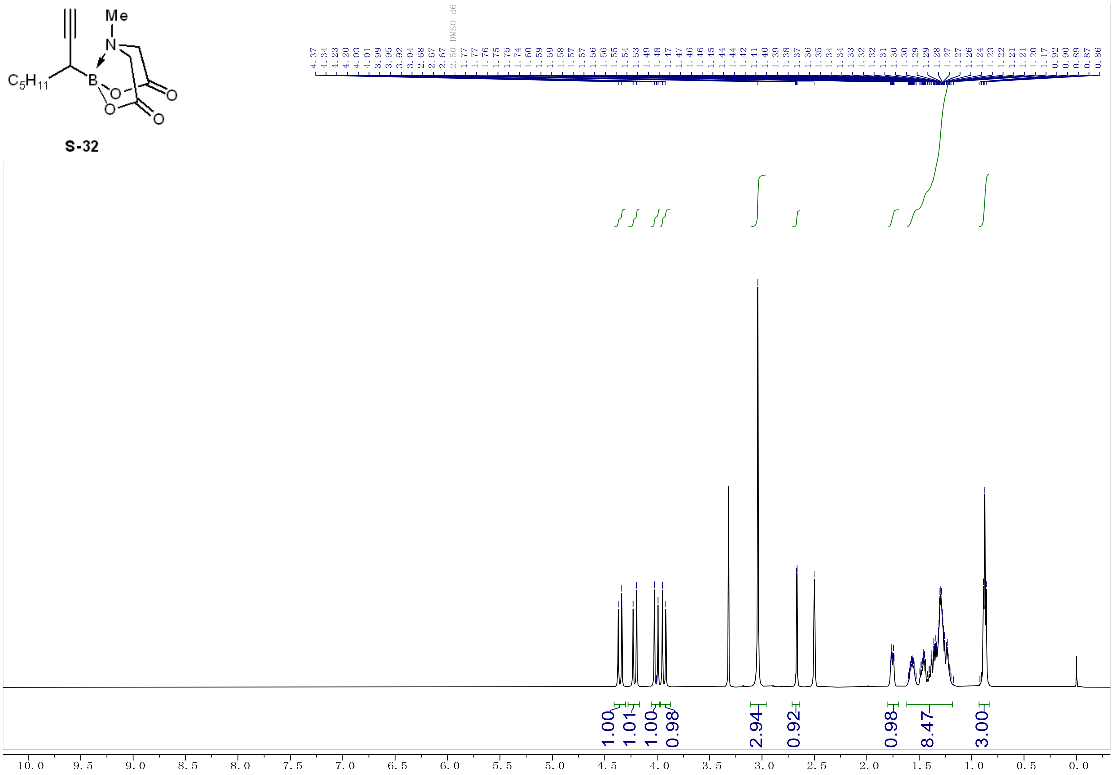

**$^{13}$C NMR of S-32 (126 MHz, DMSO-$d_6$)**

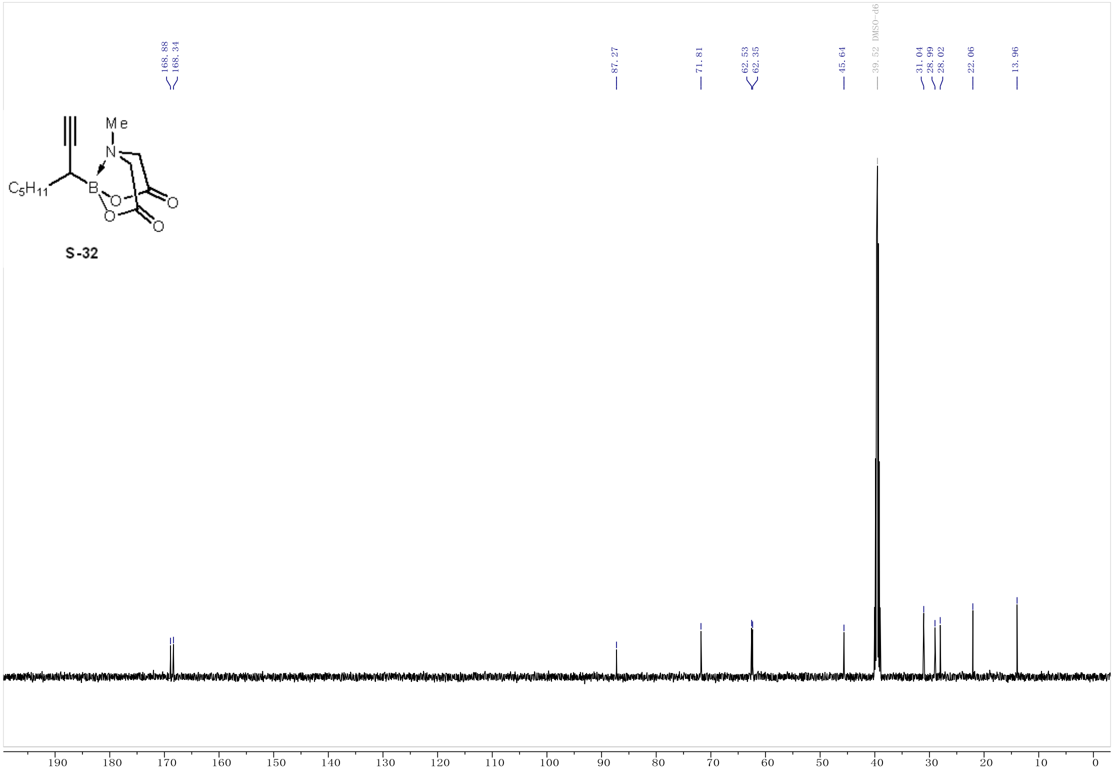


**$^{1}$H NMR of S-33 (400 MHz, DMSO-$d_6$)**

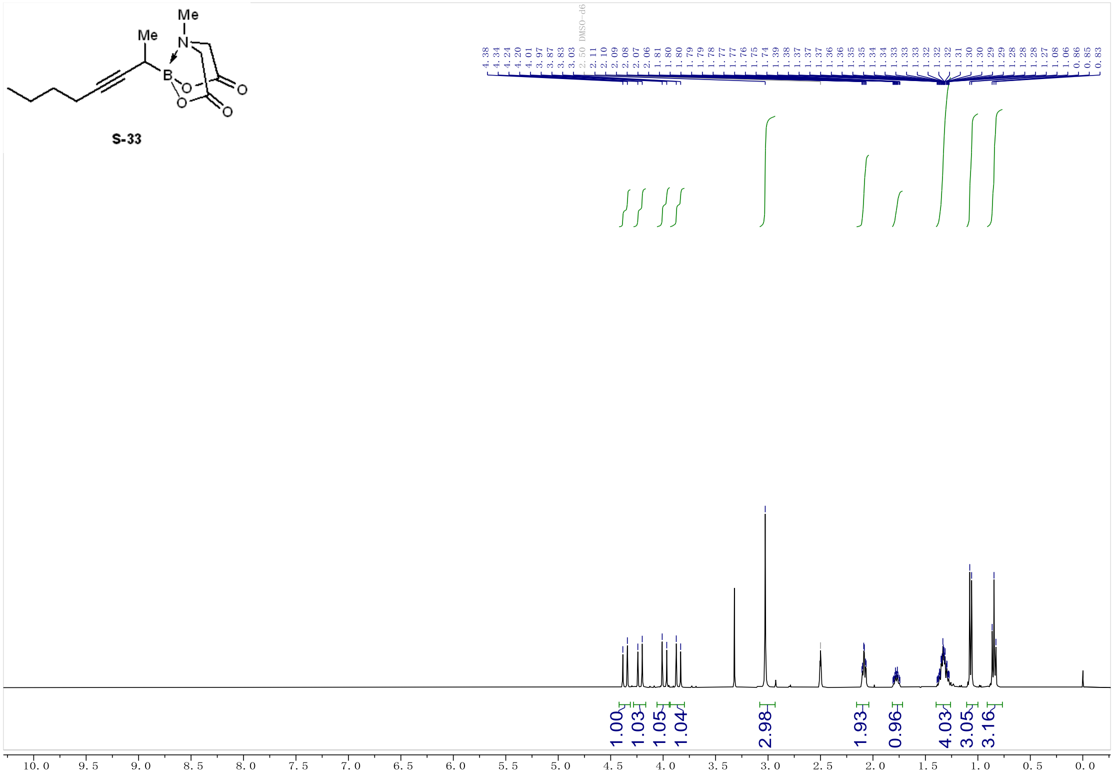

## $^{13}$C NMR of S-33 (101 MHz, DMSO-$d_6$)

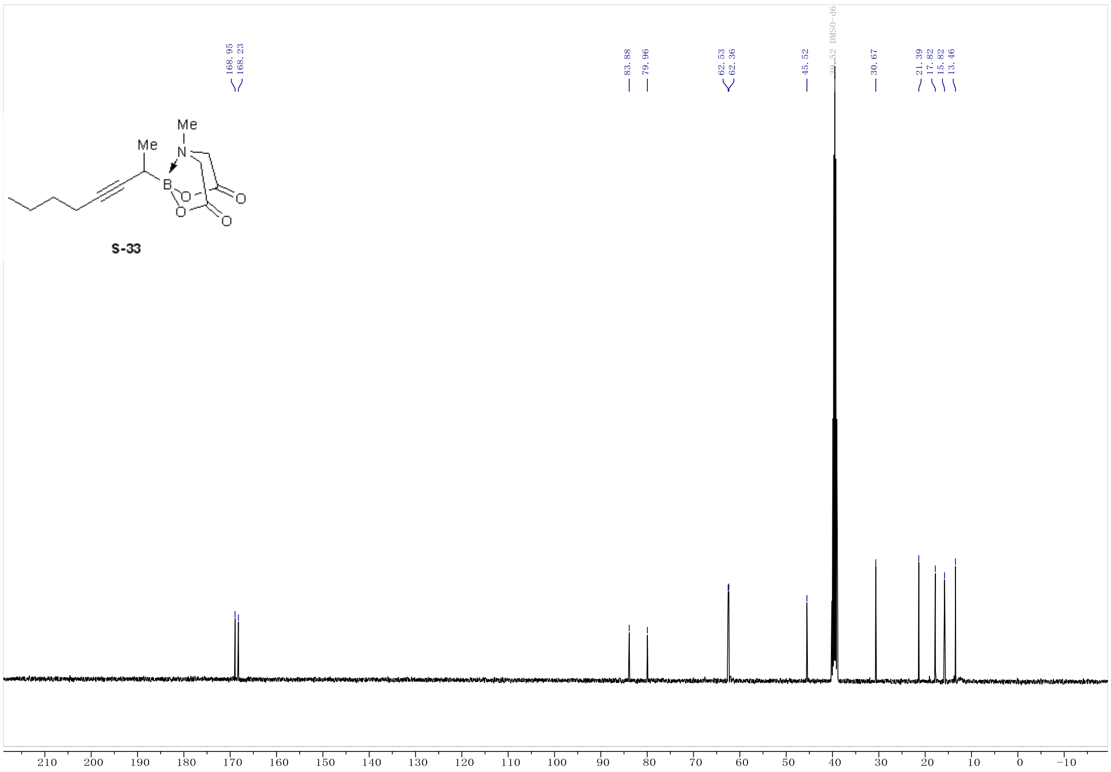


## $^{1}$H NMR of S-34 (400 MHz, DMSO-$d_6$)

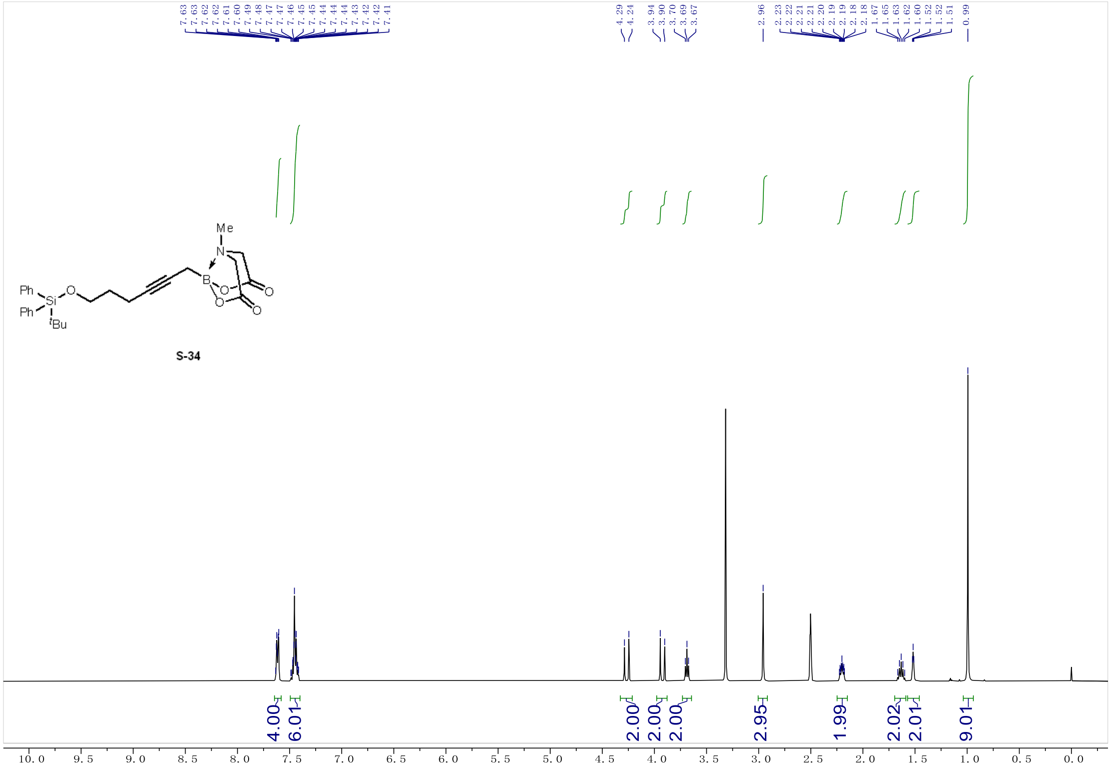

## $^{13}C$ NMR of S-34 (101 MHz, DMSO-$d_6$)

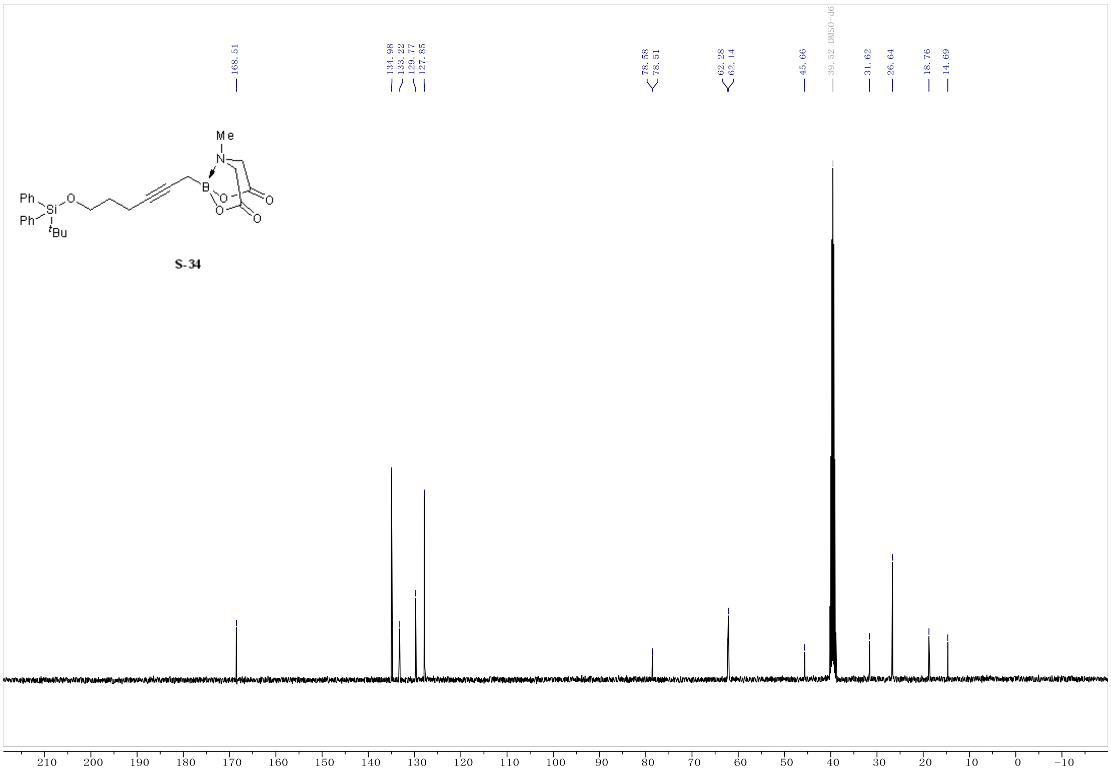

## $^{1}H$ NMR of S-35 (400 MHz, $CD_3CN$)

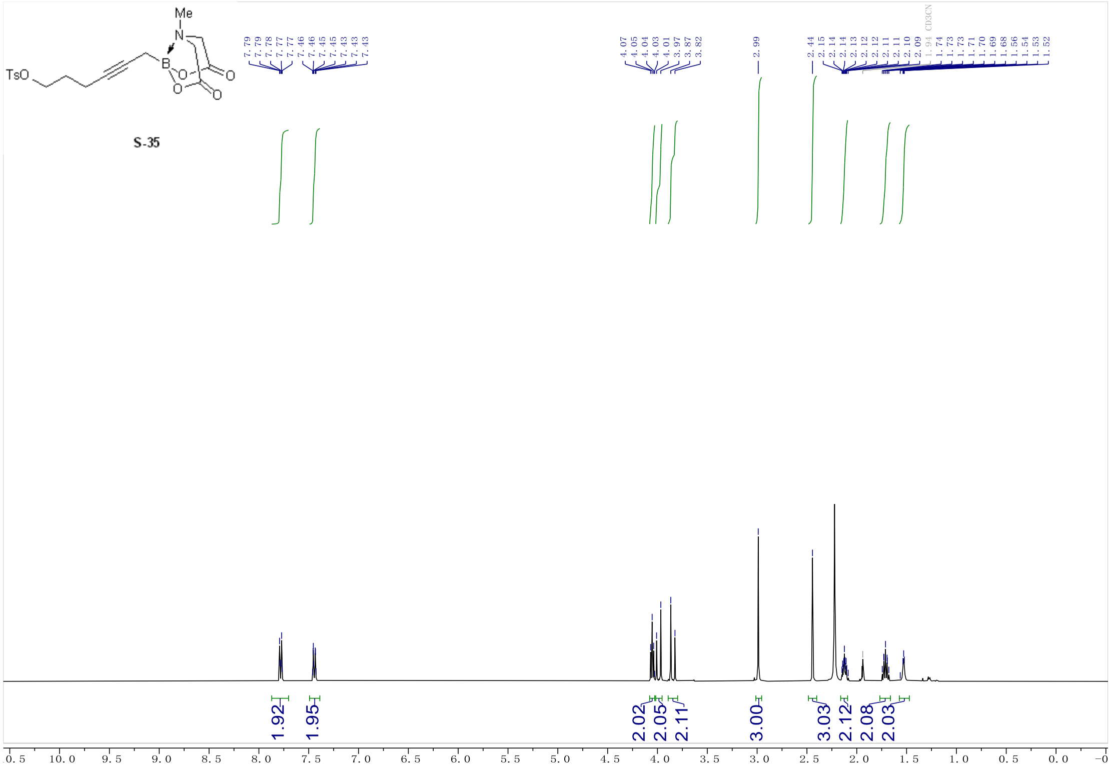

## $^{13}C$ NMR of S-35 (101 MHz, $CD_3CN$)

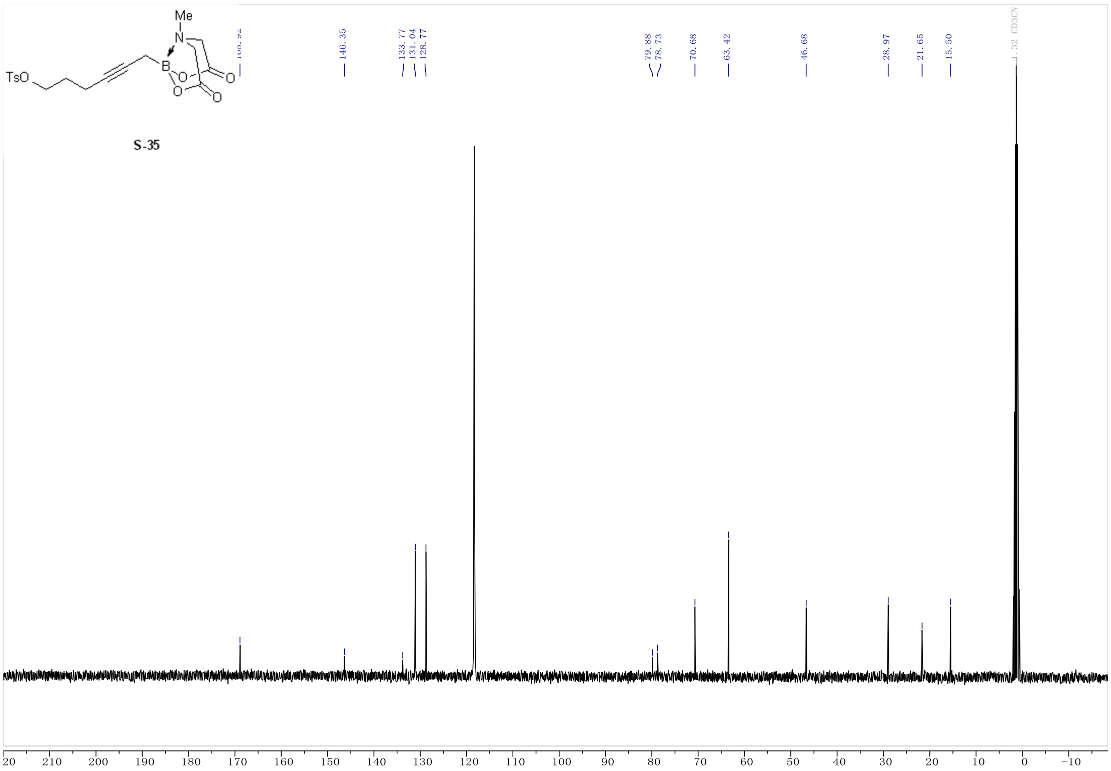

# $^{1}$H NMR of S-37 (500 MHz, Acetone-$d_6$)

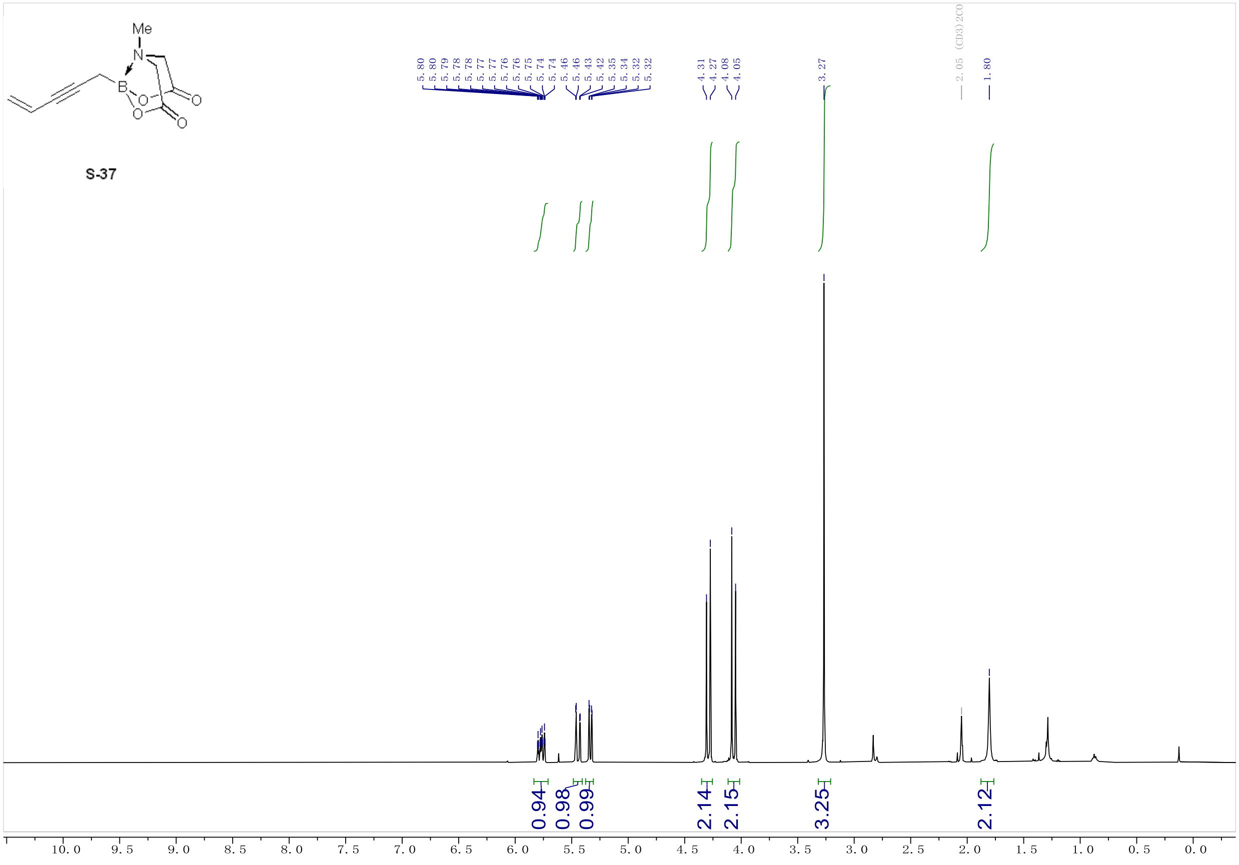

**$^{13}$C NMR of S-37 (101 MHz, Acetone-$d_6$)**

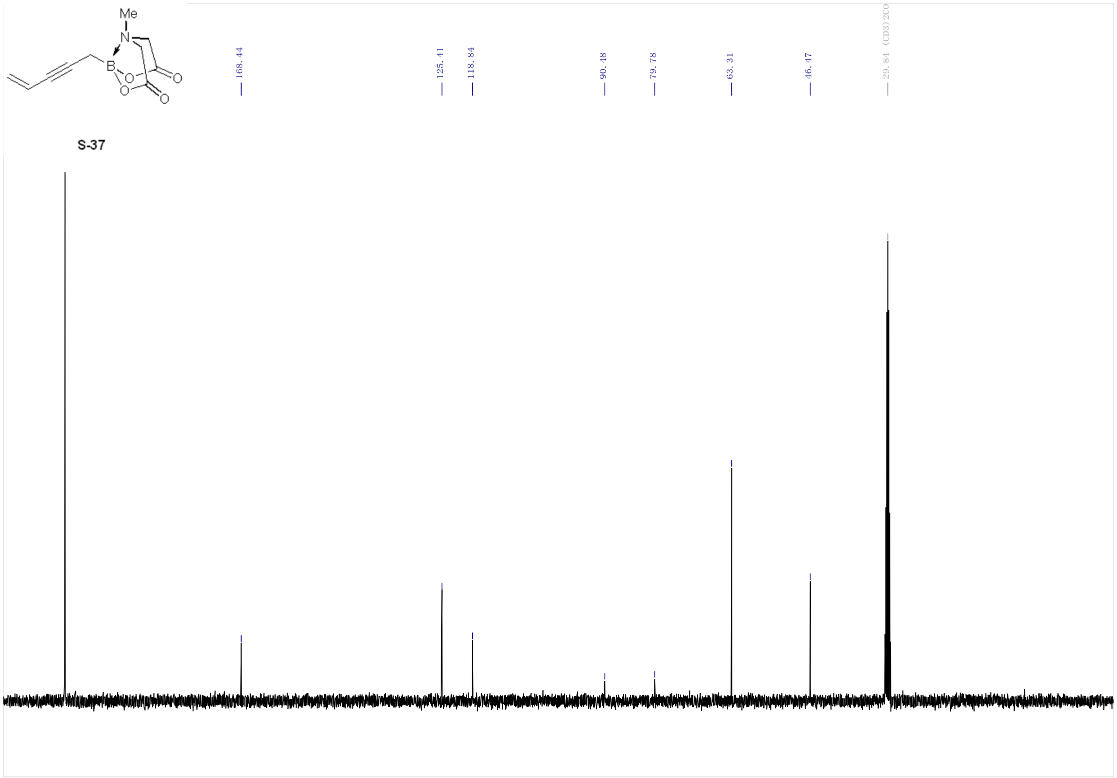


**$^{1}$H NMR of S-41 (400 MHz, DMSO-$d_6$)**

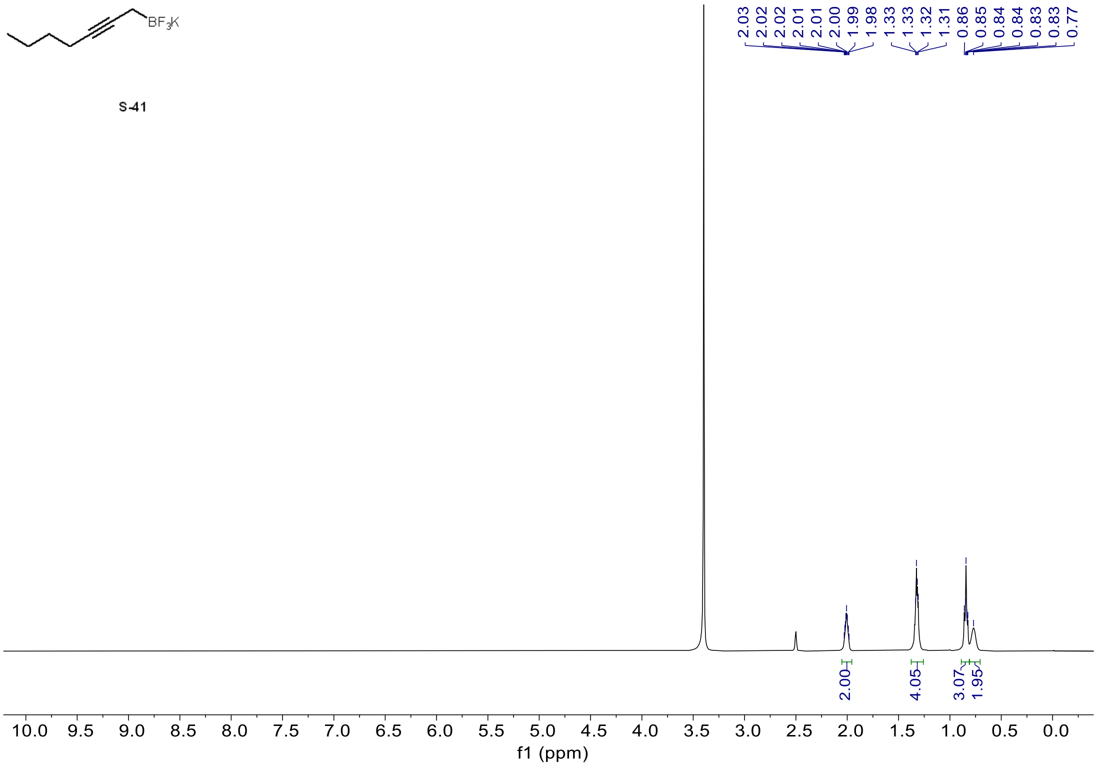

**$^{13}$C NMR of S-41 (101 MHz, DMSO-*d*$_6$)**

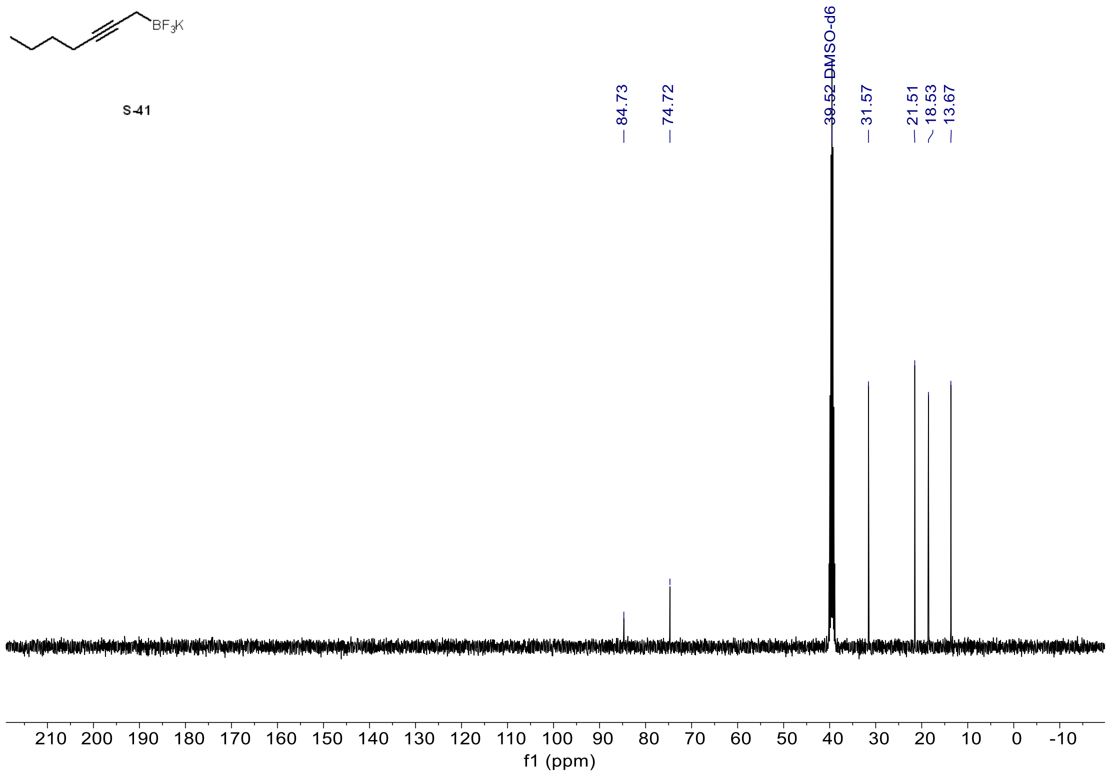


**$^{11}$B NMR of S-41 (128 MHz, DMSO-*d*$_6$)**

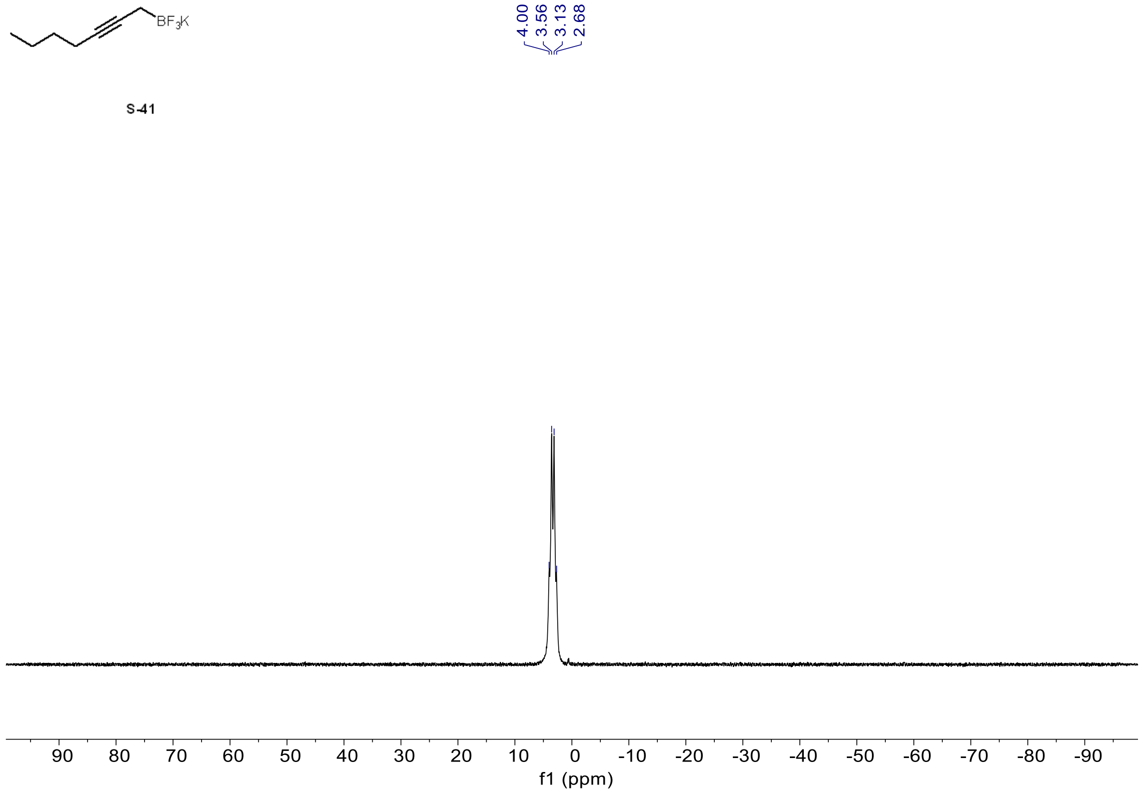

**$^{19}$F NMR of S-41 (376 MHz, DMSO-$d_6$)**

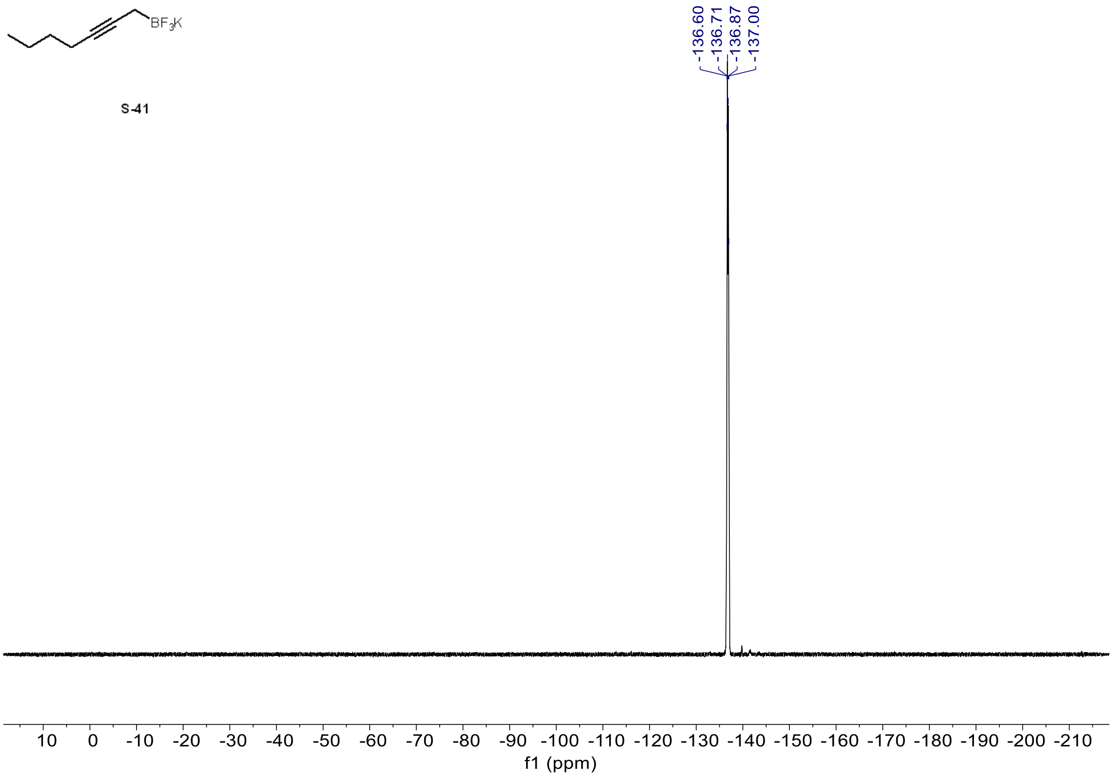


**$^{1}$H NMR of S-42 (500 MHz, CDCl$_3$)**

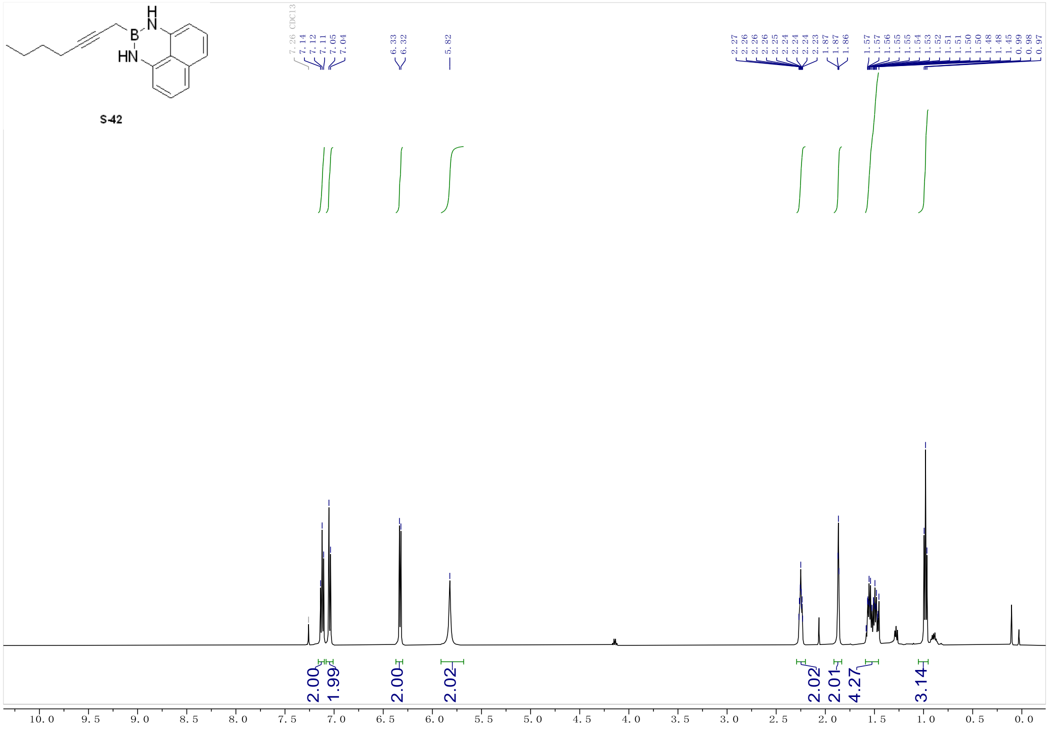

## $^{13}C$ NMR of S-42 (126 MHz, $CDCl_3$)

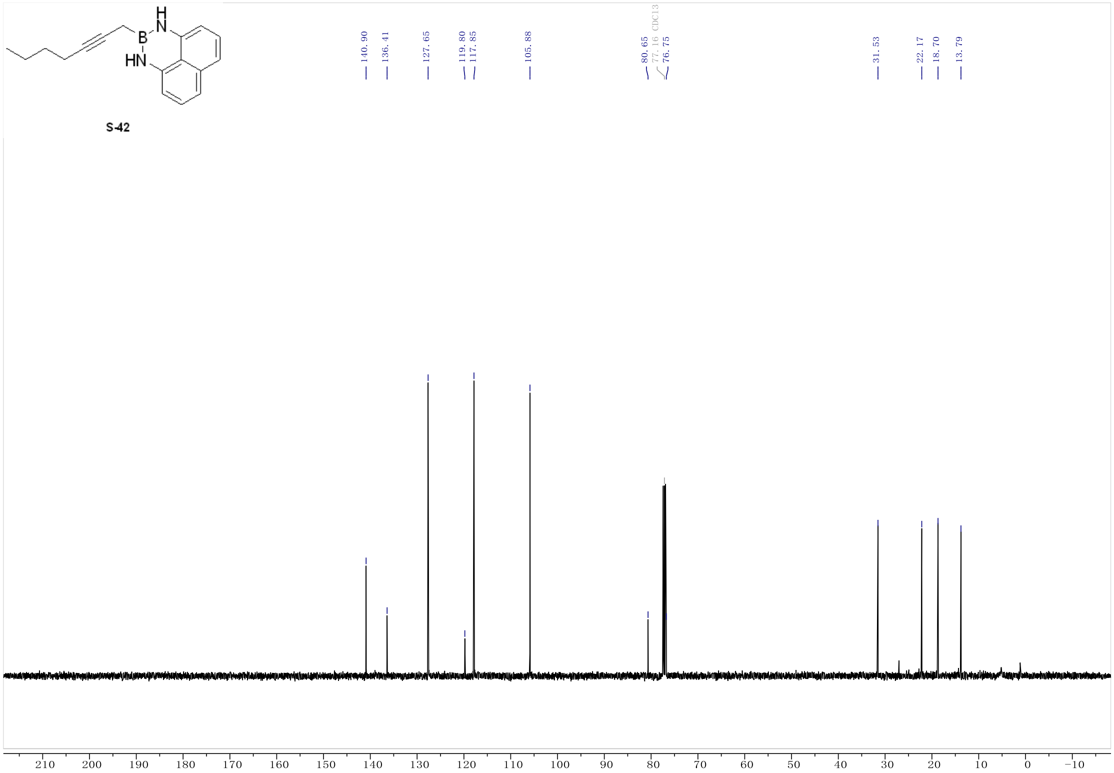

## $^{1}H$ NMR of 1 (500 MHz, DMSO-$d_6$)

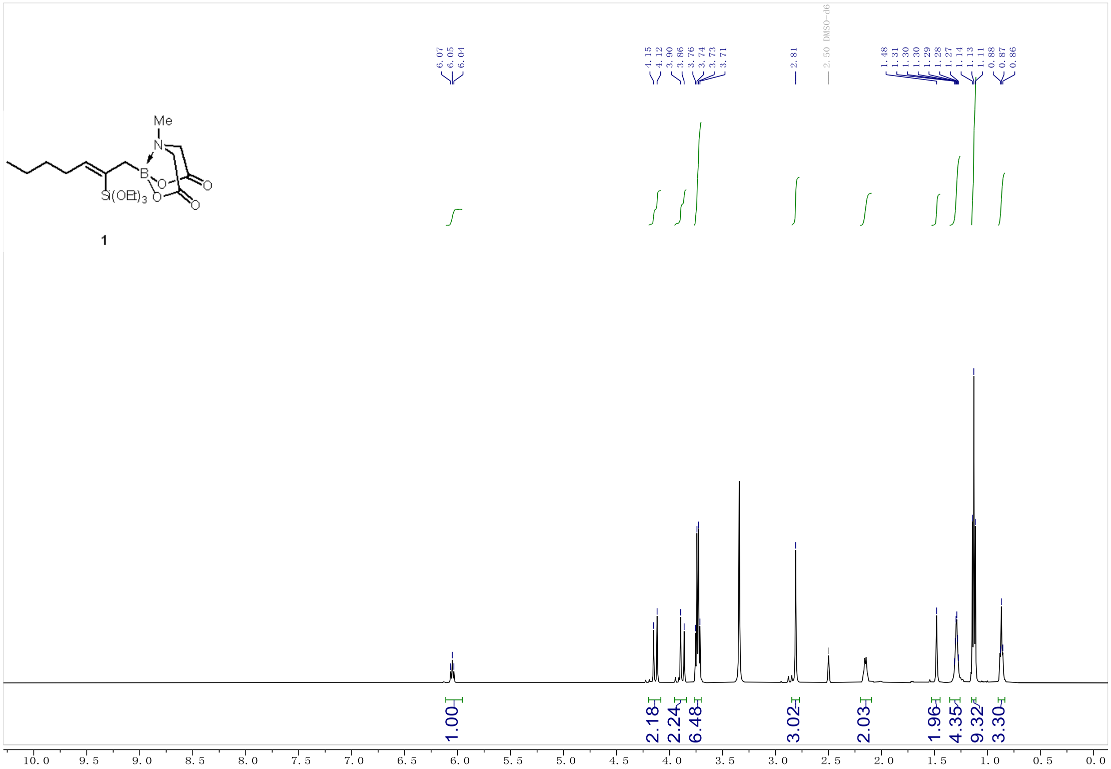

## $^{13}$C NMR of 1 (126 MHz, DMSO-$d_6$)

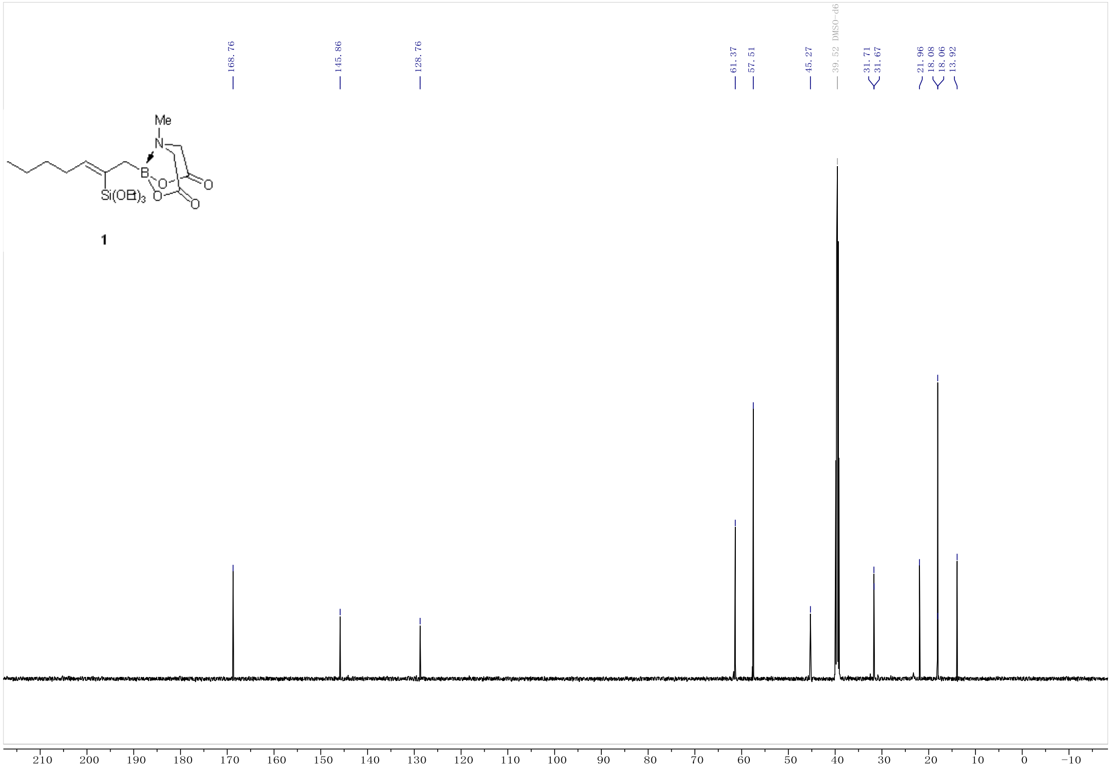


## $^{1}$H NMR of 2 (500 MHz, $CD_3CN$)

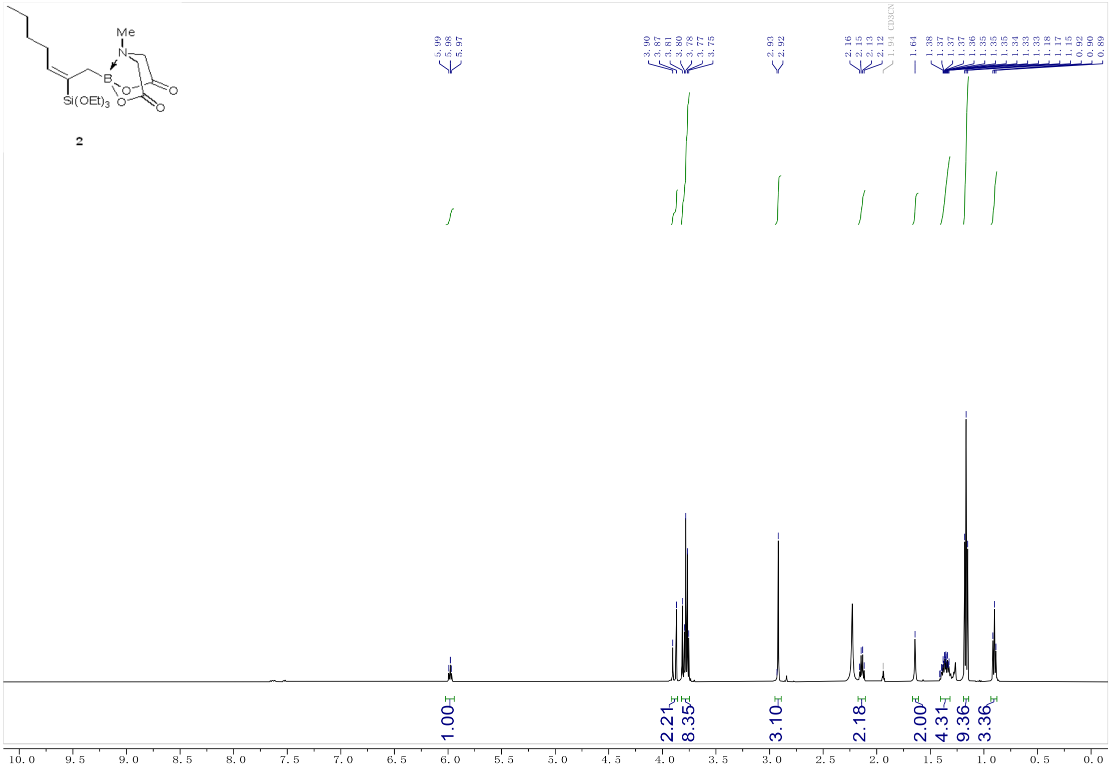

**$^{13}$C NMR of 2 (126 MHz, $CD_3CN$)**

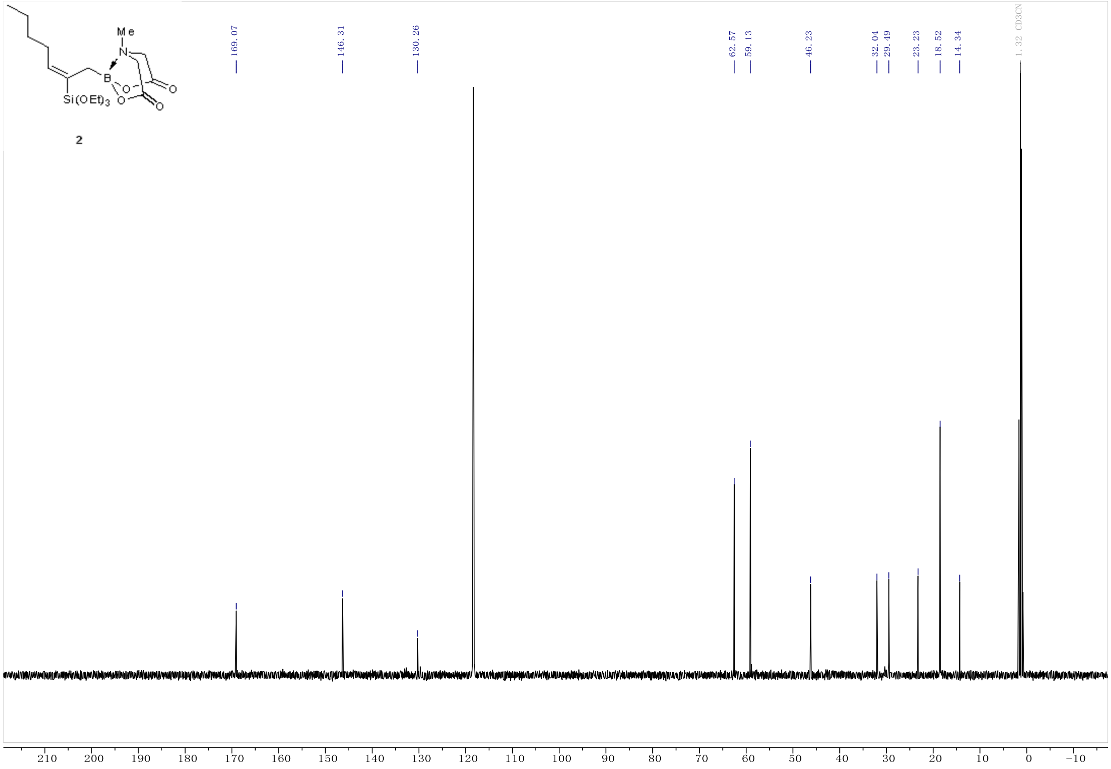

**$^{1}$H NMR of 3 (400 MHz, Acetone-$d_6$)**

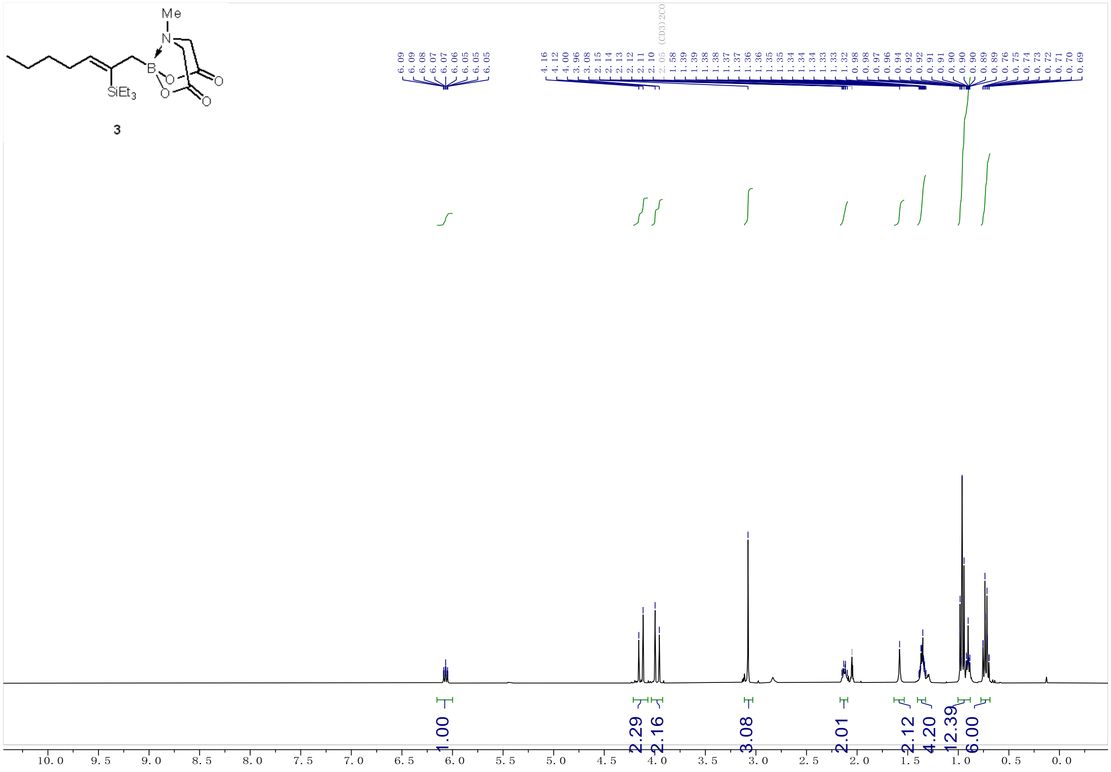

**$^{13}$C NMR of 3 (101 MHz, DMSO-$d_6$)**

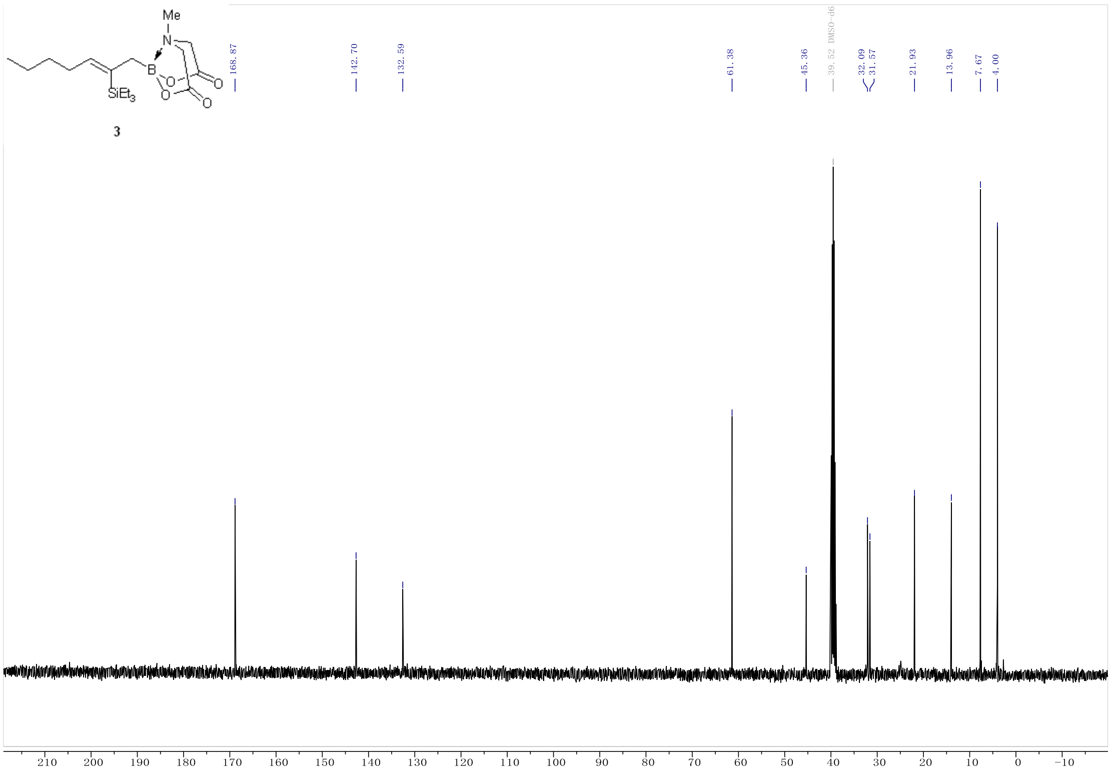


**$^{11}$B NMR of 3 (128 MHz, Acetone-$d_6$)**

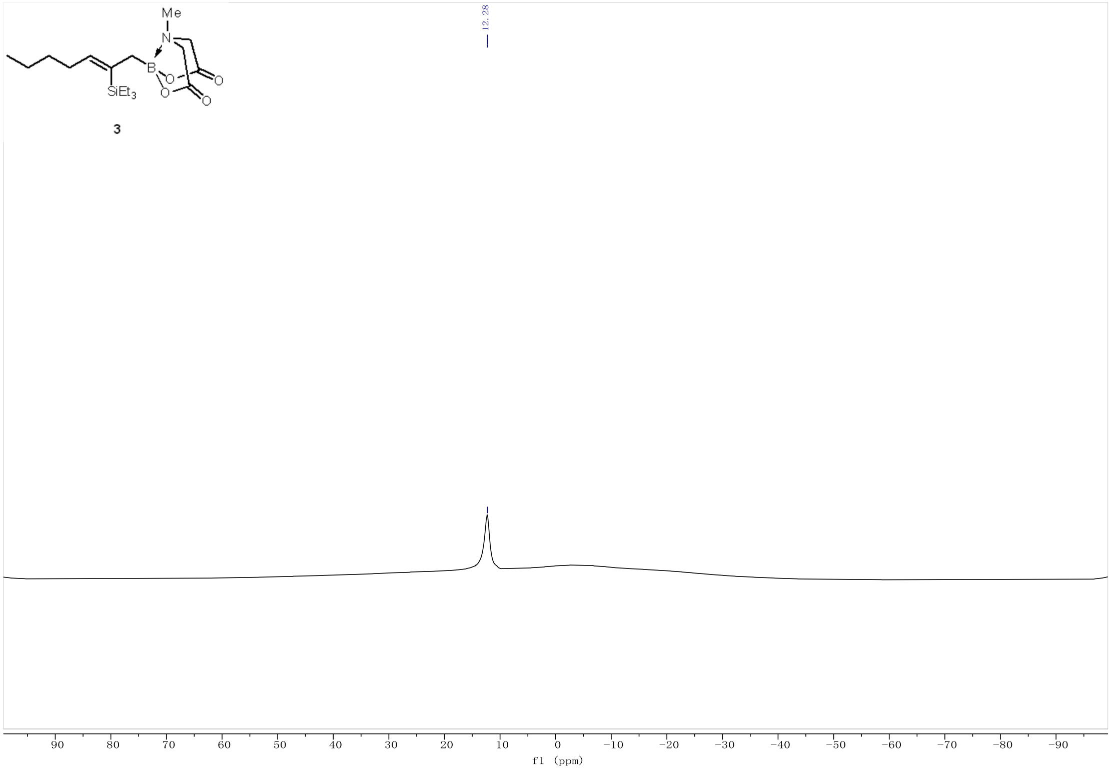

**NOE of 3**

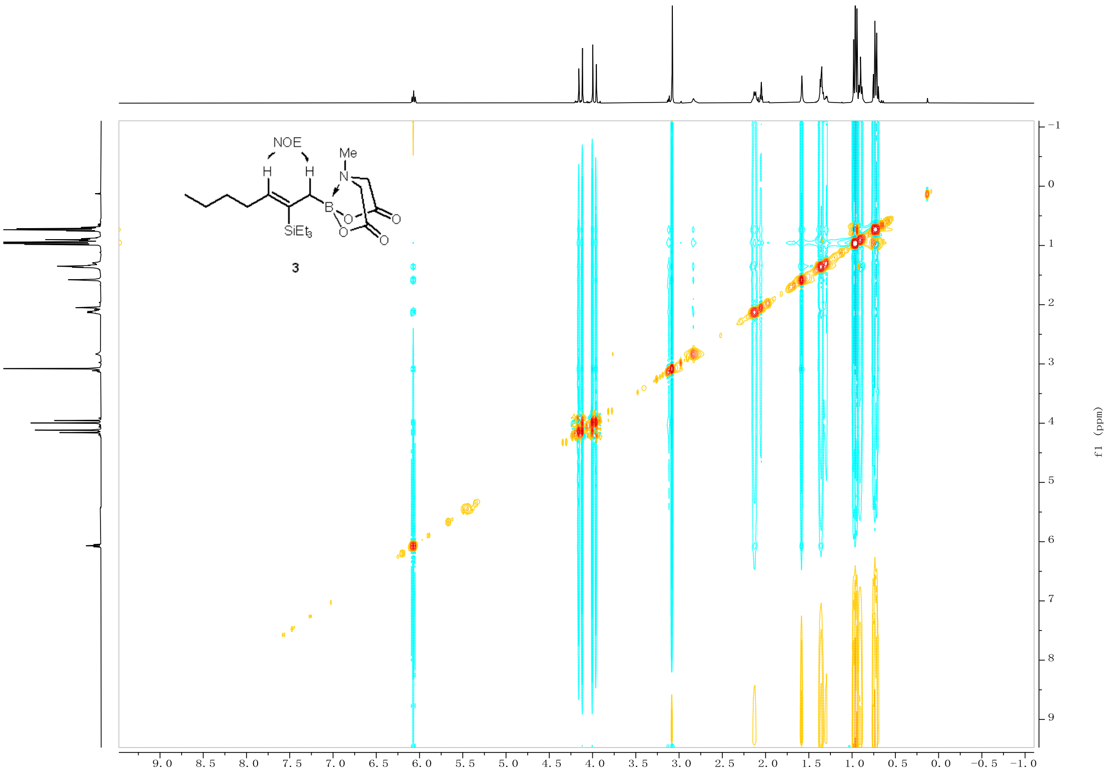

NOE
H
H
Me
N
B
O
O
O
O
SiEt3
3
9.0
8.5
8.0
7.5
7.0
6.5
6.0
5.5
5.0
4.5
4.0
3.5
3.0
2.5
2.0
1.5
1.0
0.5
0.0
-0.5
-1.0
f1 (ppm)


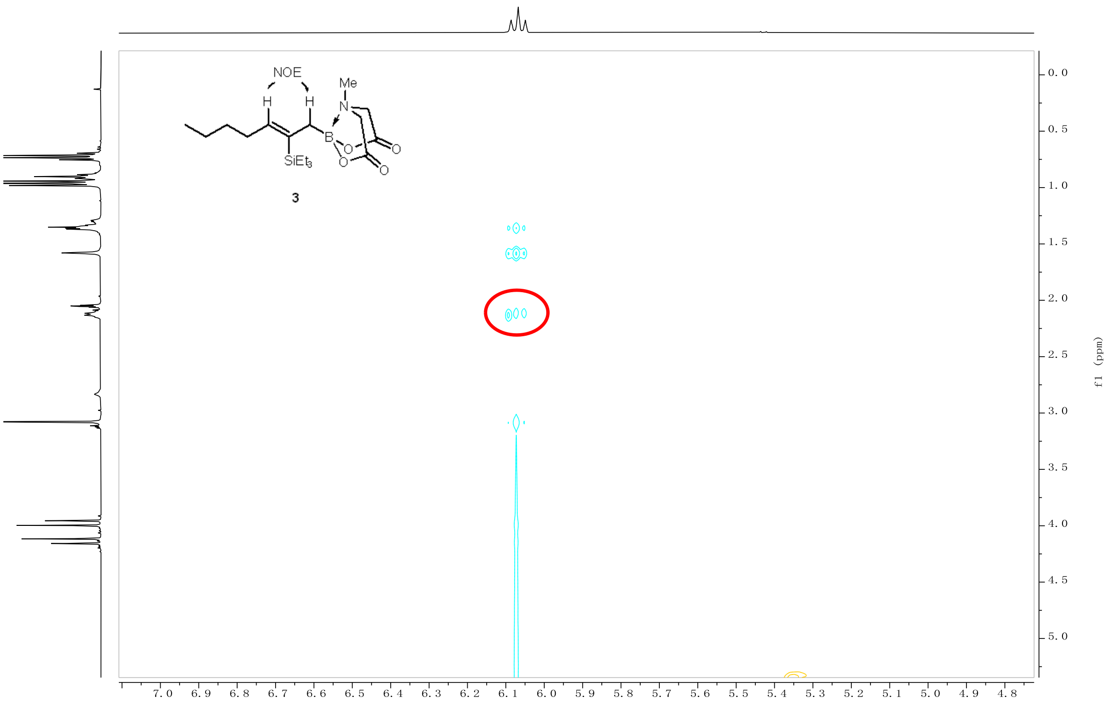

NOE
H
H
Me
N
B
O
O
O
O
SiEt3
3
7.0
6.9
6.8
6.7
6.6
6.5
6.4
6.3
6.2
6.1
6.0
5.9
5.8
5.7
5.6
5.5
5.4
5.3
5.2
5.1
5.0
4.9
4.8
f1 (ppm)

$^{1}$H NMR of 4 (400 MHz, Acetone-$d_6$)

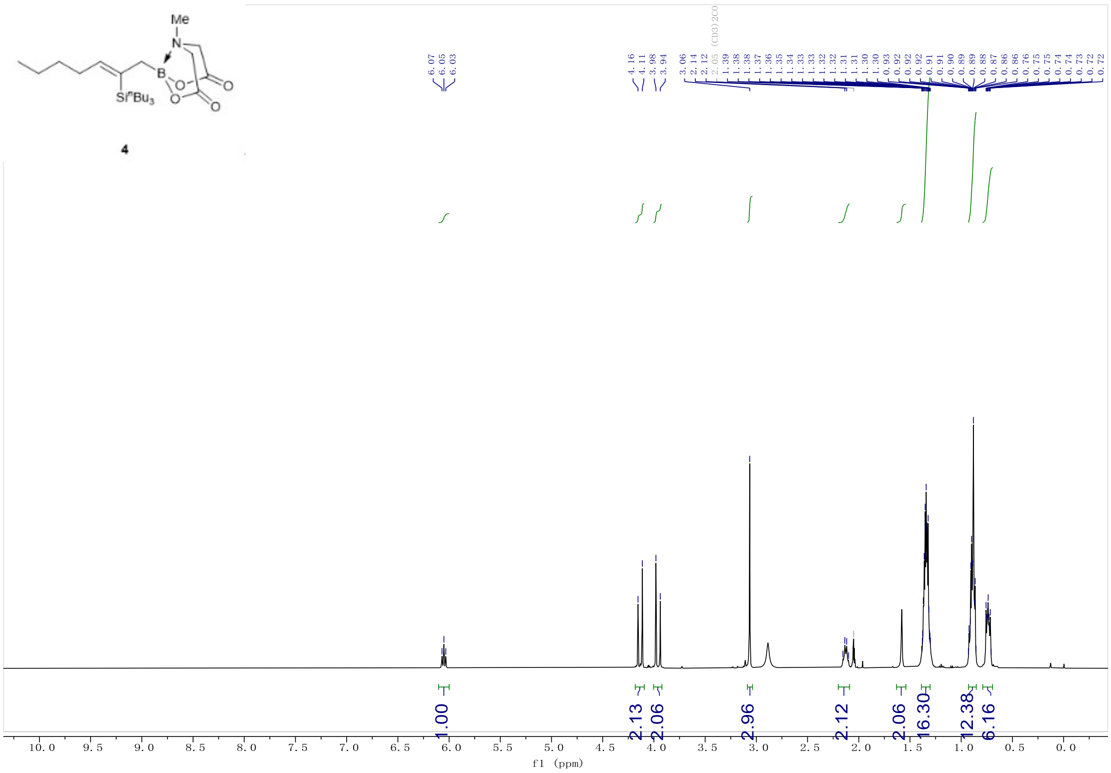

$^{13}$C NMR of 4 (101 MHz, Acetone-$d_6$)

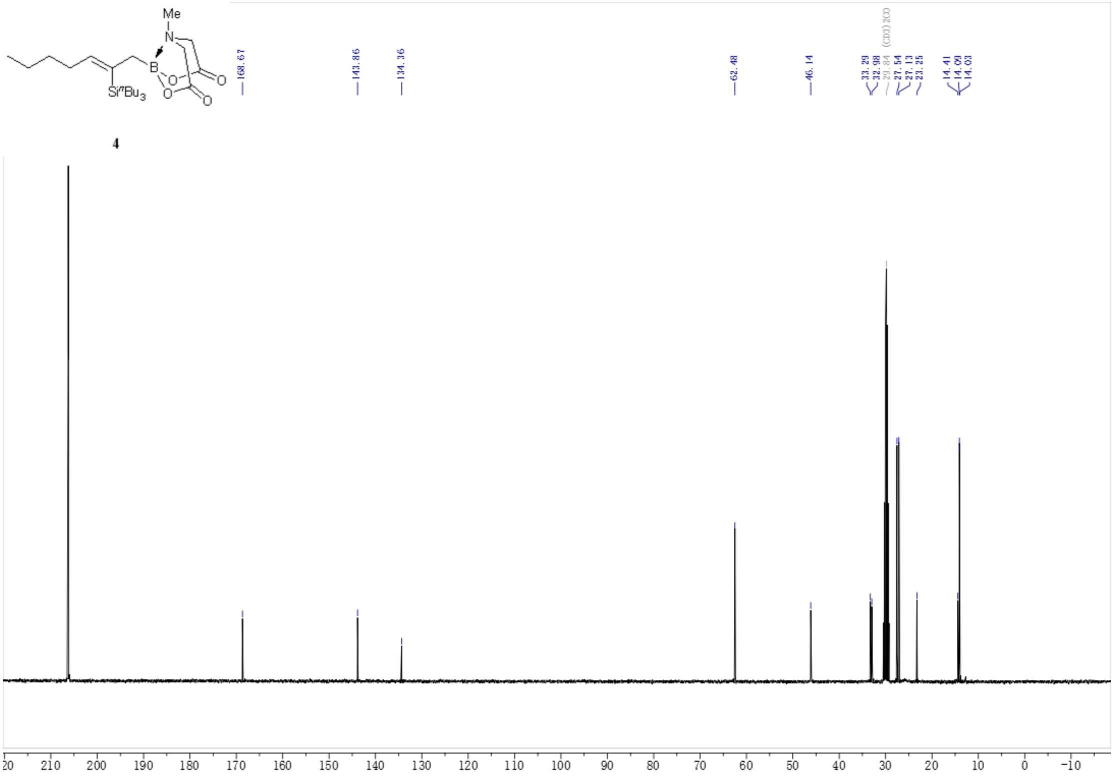

**$^{1}$H NMR of 5 (400 MHz, DMSO-$d_6$)**

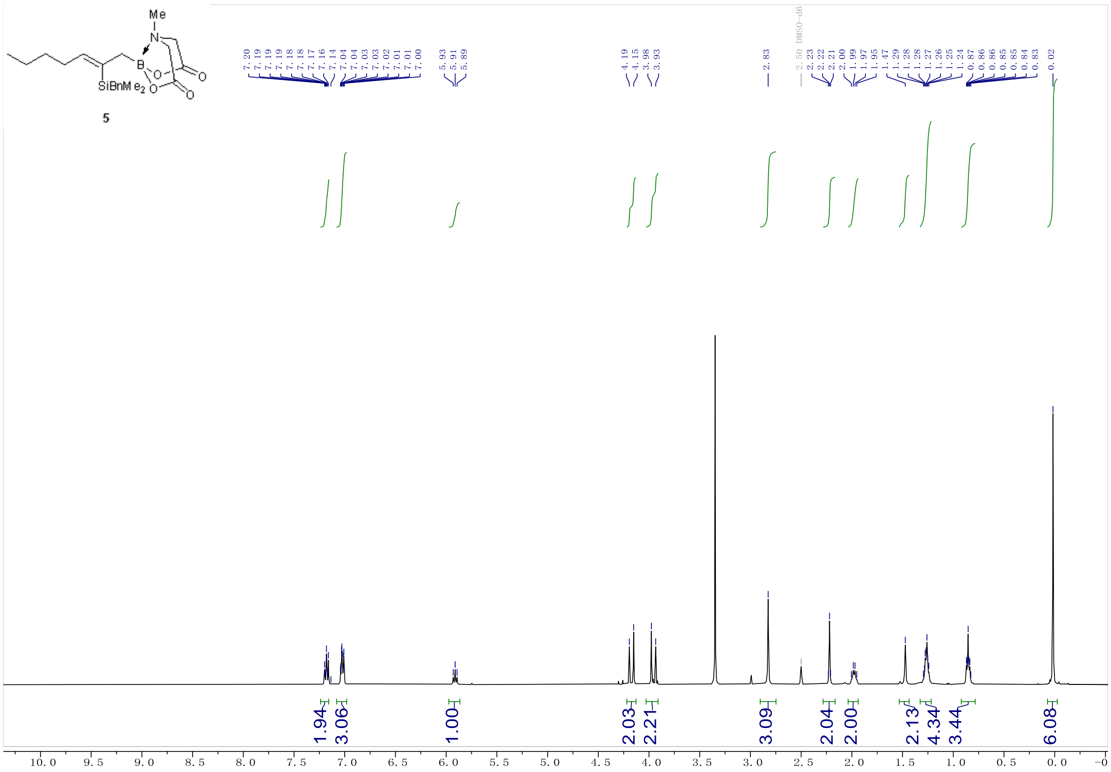

**$^{13}$C NMR of 5 (101 MHz, DMSO-$d_6$)**

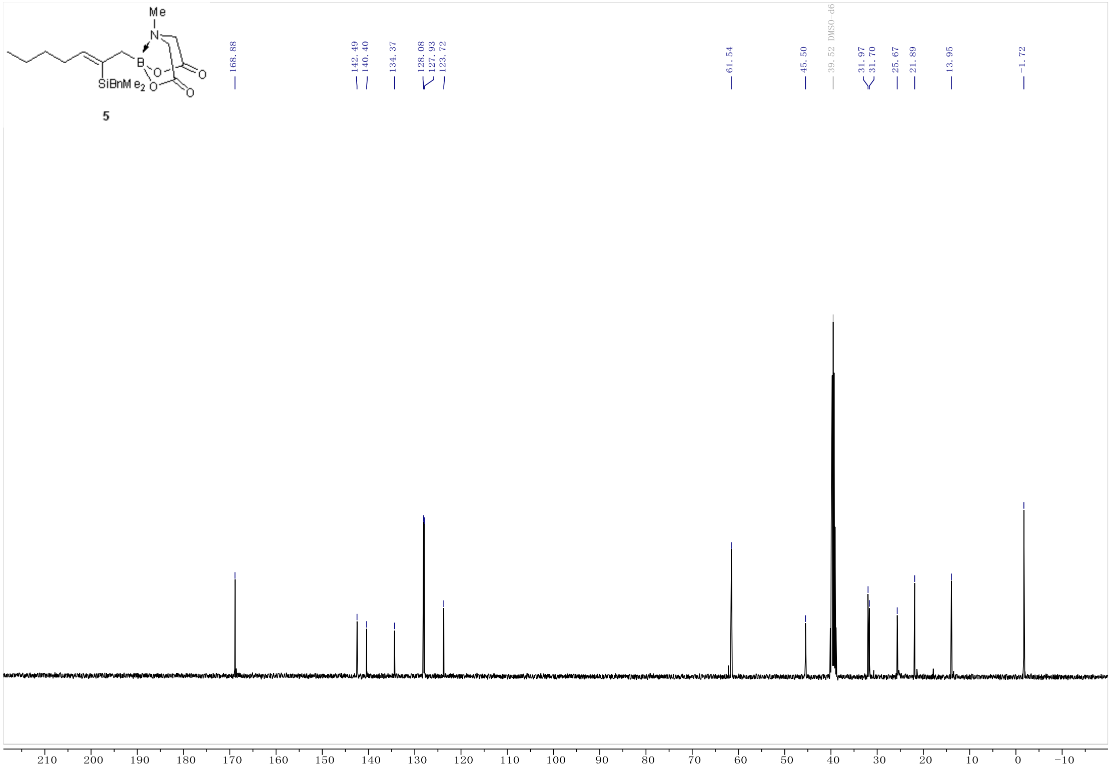

**$^{1}$H NMR of 6 (500 MHz, DMSO-$d_6$)**

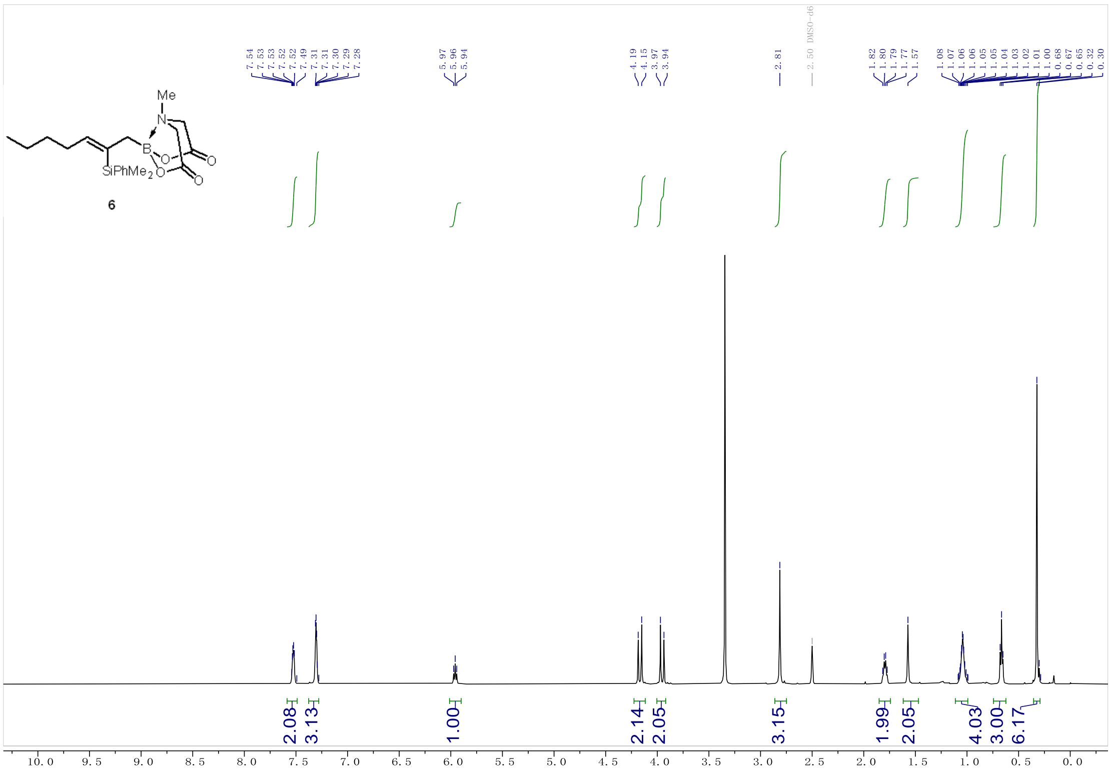


## $^{13}$C NMR of 6 (126 MHz, DMSO-$d_6$)

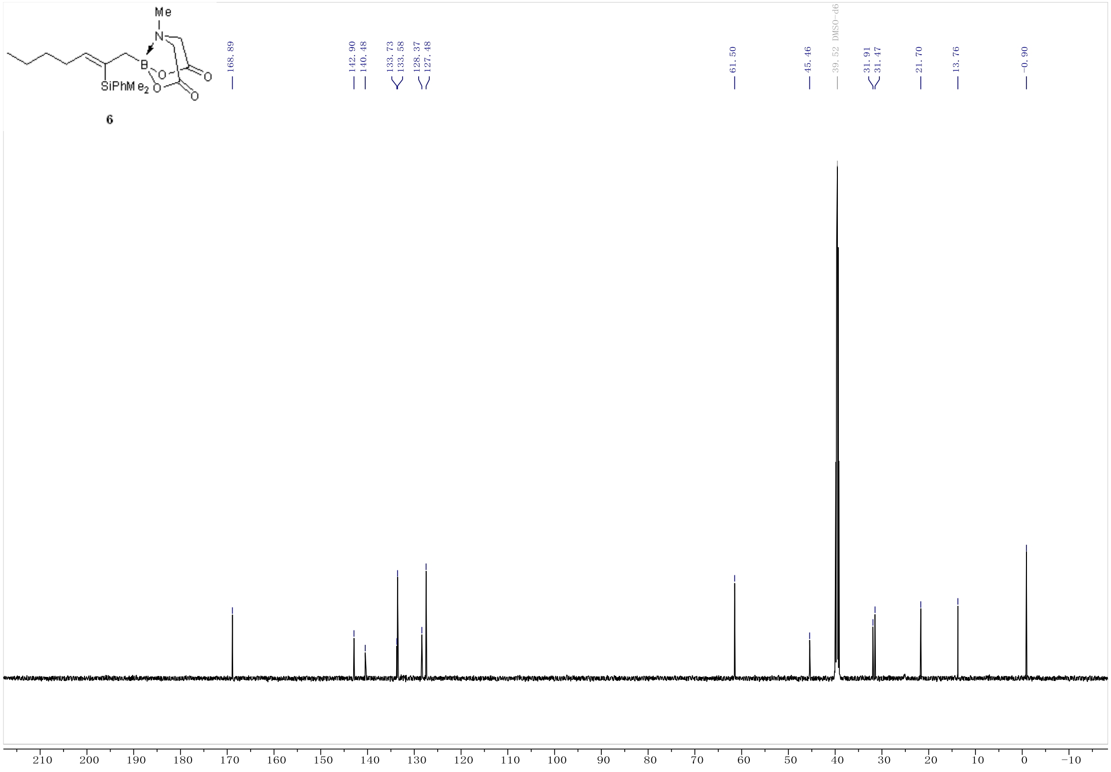

## $^{11}$B NMR of 6 (128 MHz, Acetone-$d_6$)

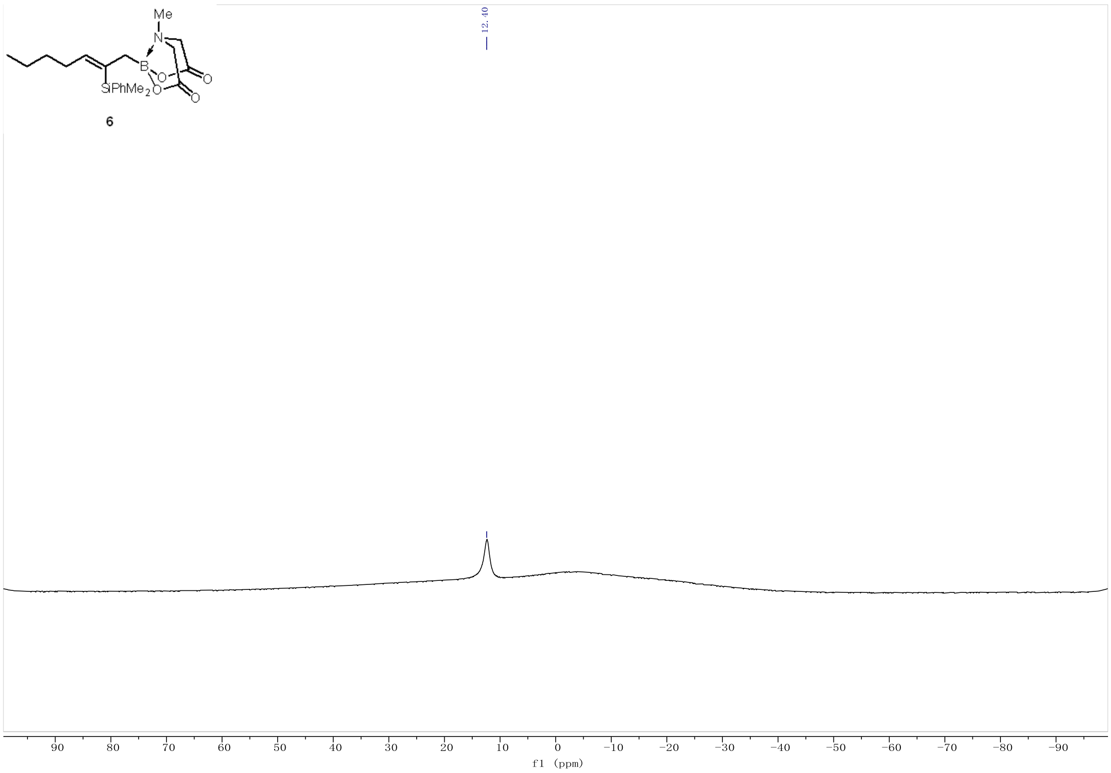

## $^{1}$H NMR of 7 (400 MHz, Acetone-$d_6$)

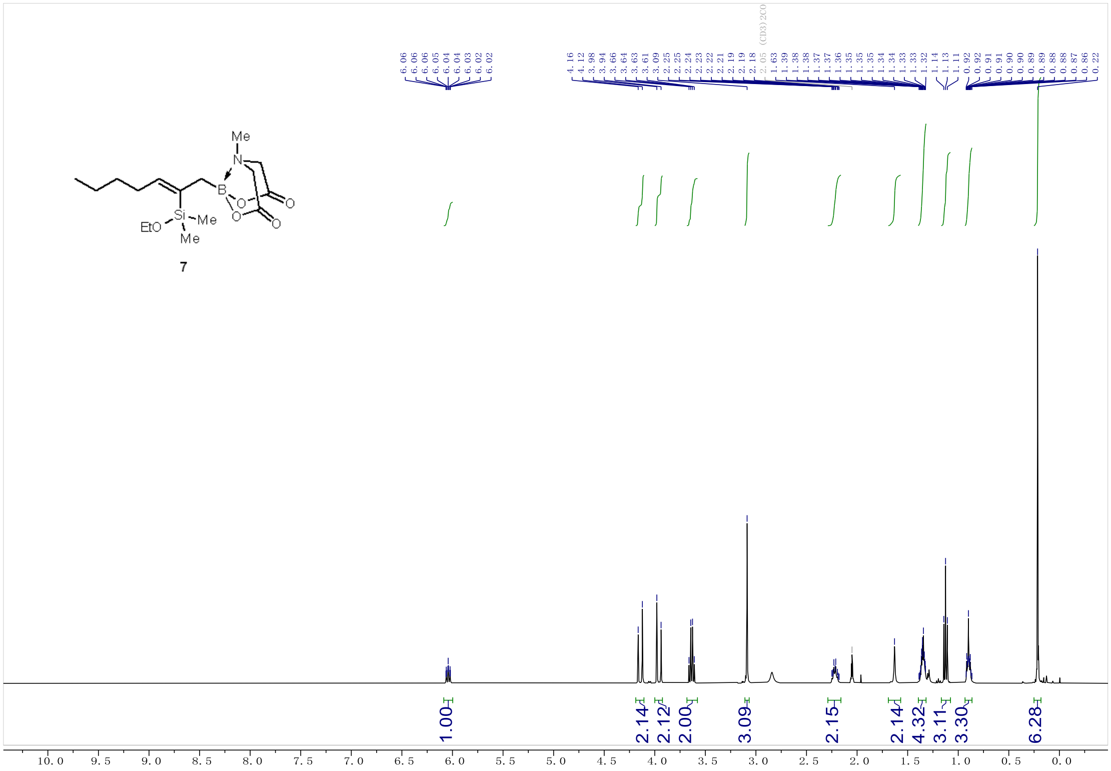

## $^{13}$C NMR of 7 (101 MHz, Acetone-$d_6$)

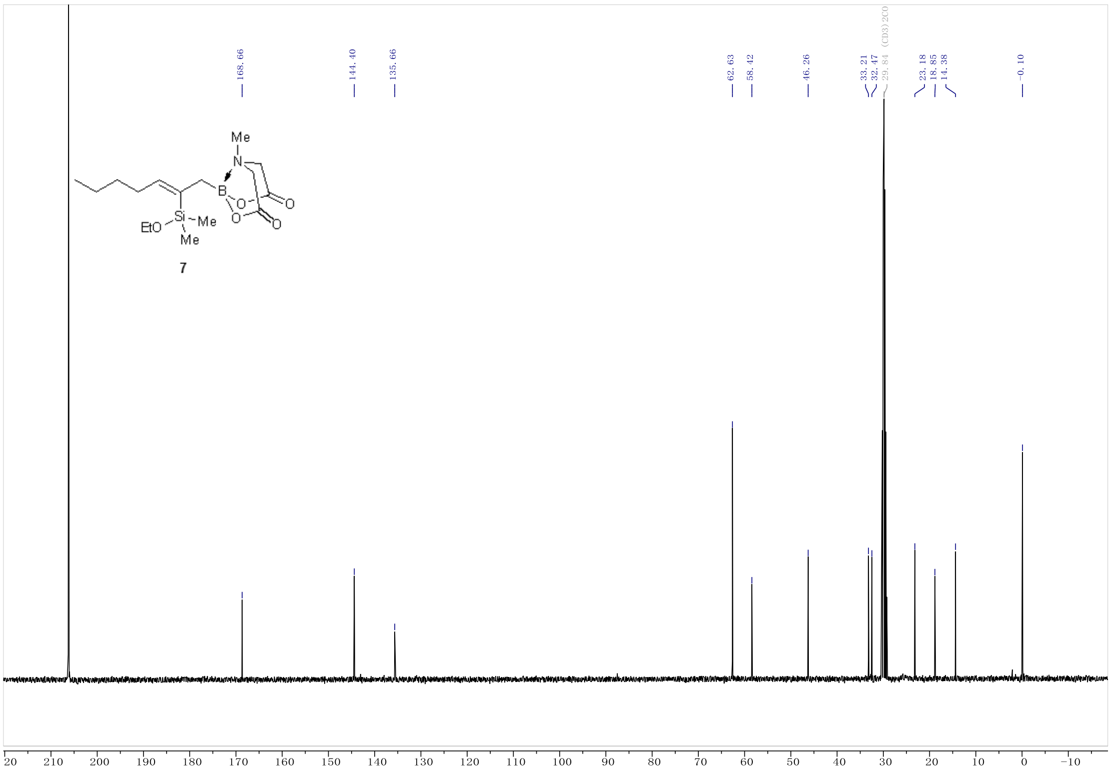


## $^{1}$H NMR of 8 (400 MHz, DMSO-$d_6$)

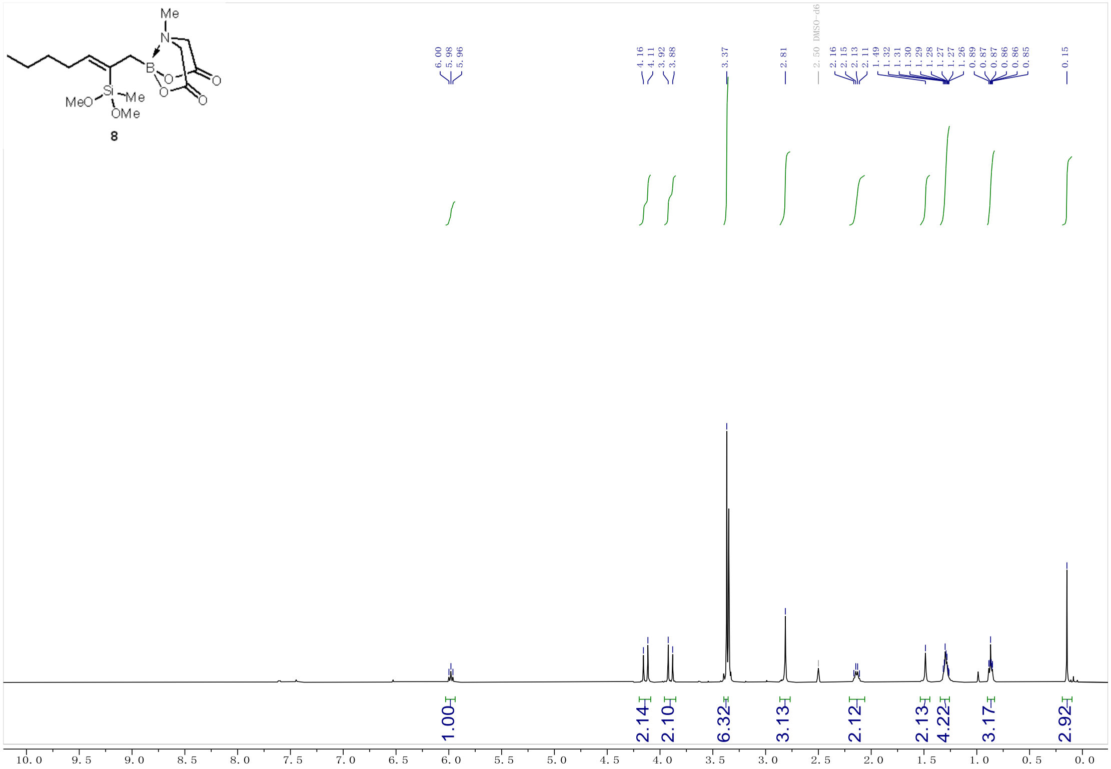


## $^{13}$C NMR of 8 (101 MHz, DMSO-$d_6$)

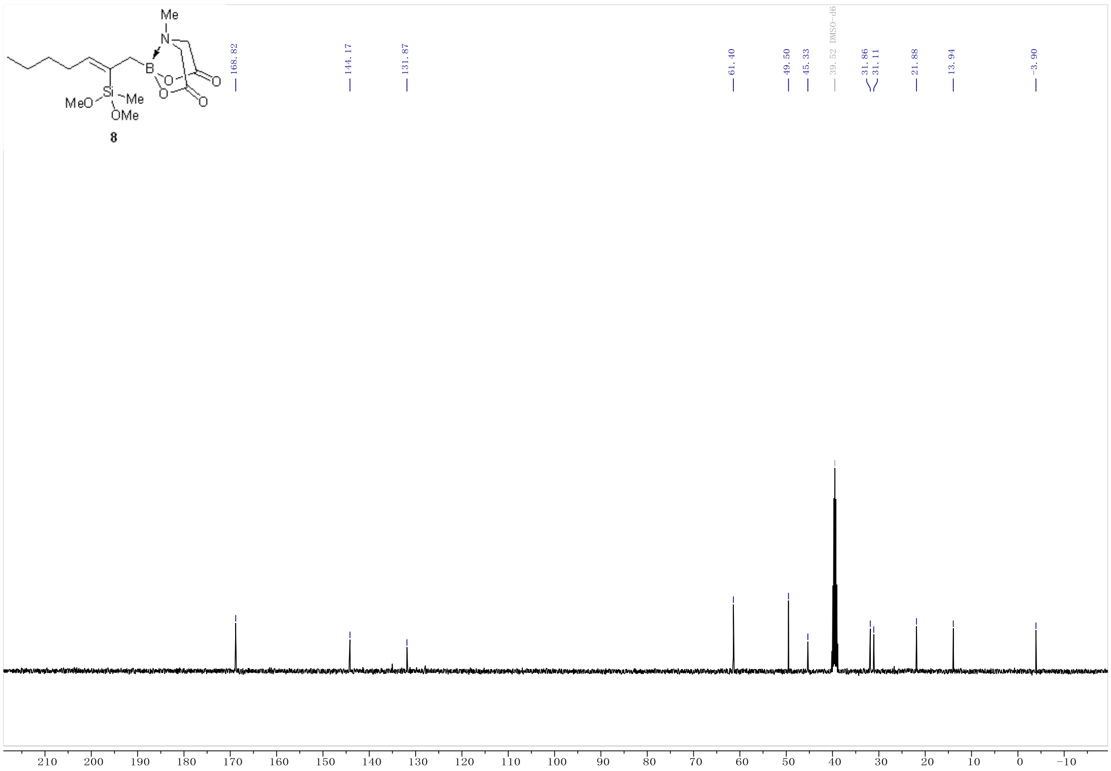

## $^1$H NMR of 9 (mixture of *Z* & *E* isomers) (500 MHz, Acetone-$d_6$)

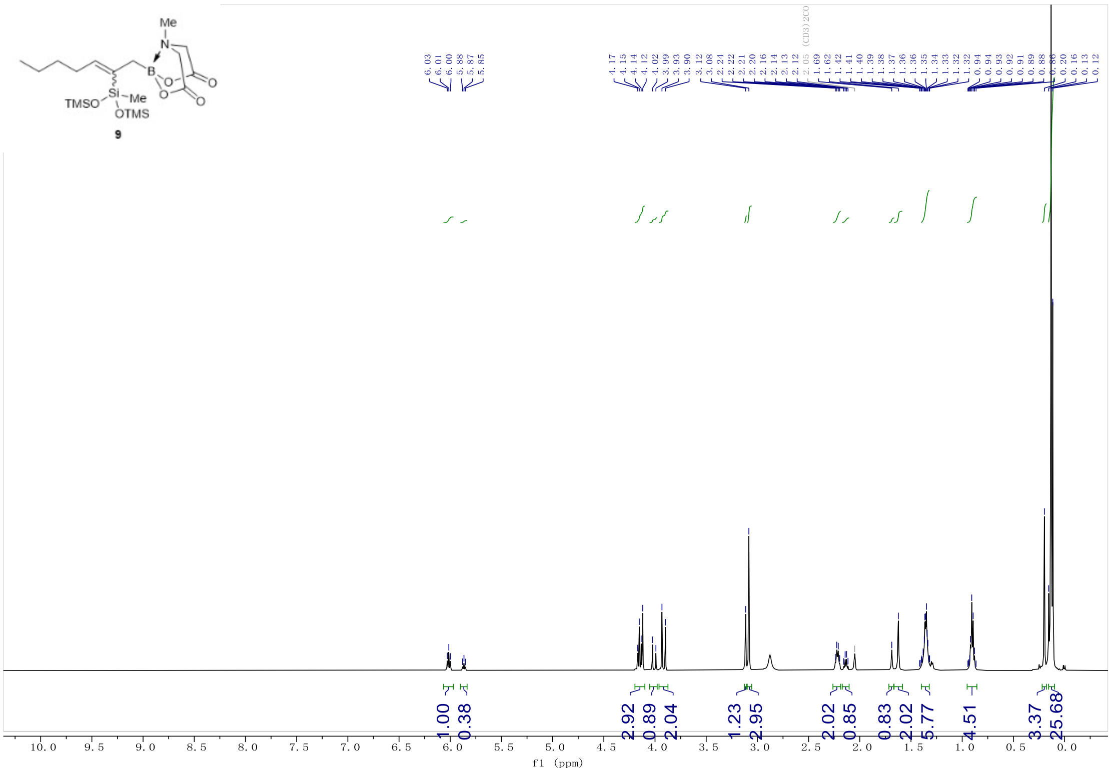

## $^{13}$C NMR of 9 (mixture of *Z* & *E* isomers) (126 MHz, Acetone-$d_6$)

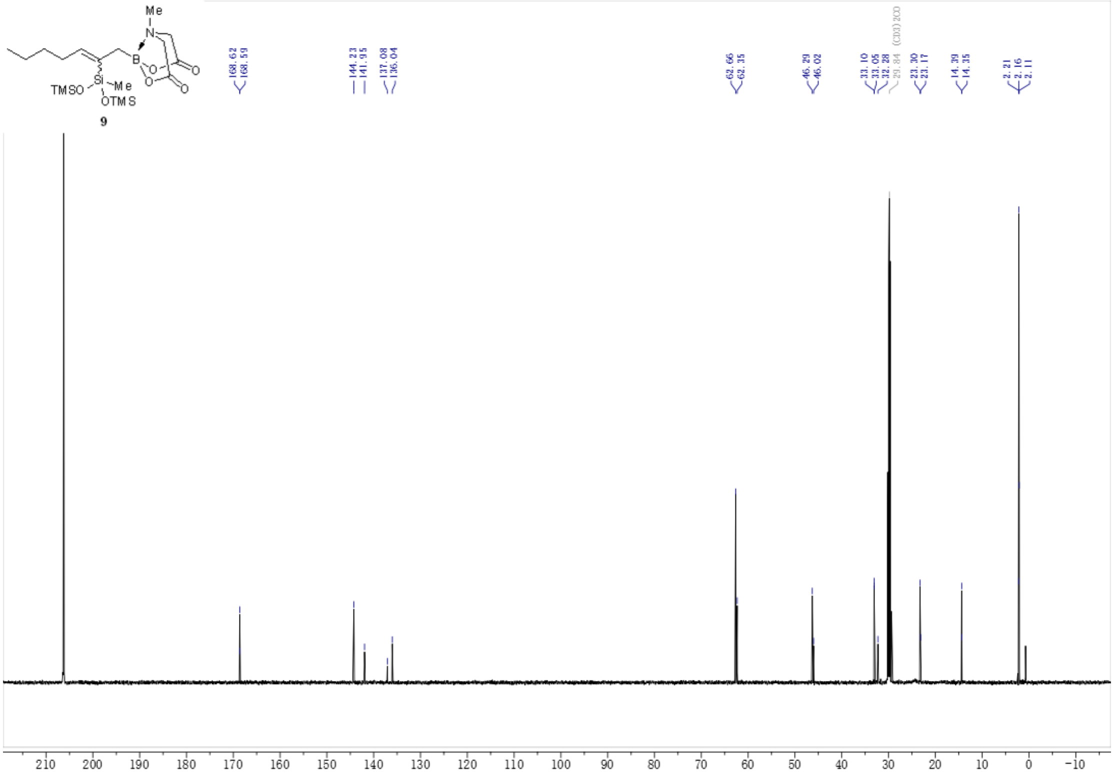


## $^{1}$H NMR of 10 (400 MHz, Acetone-$d_6$)

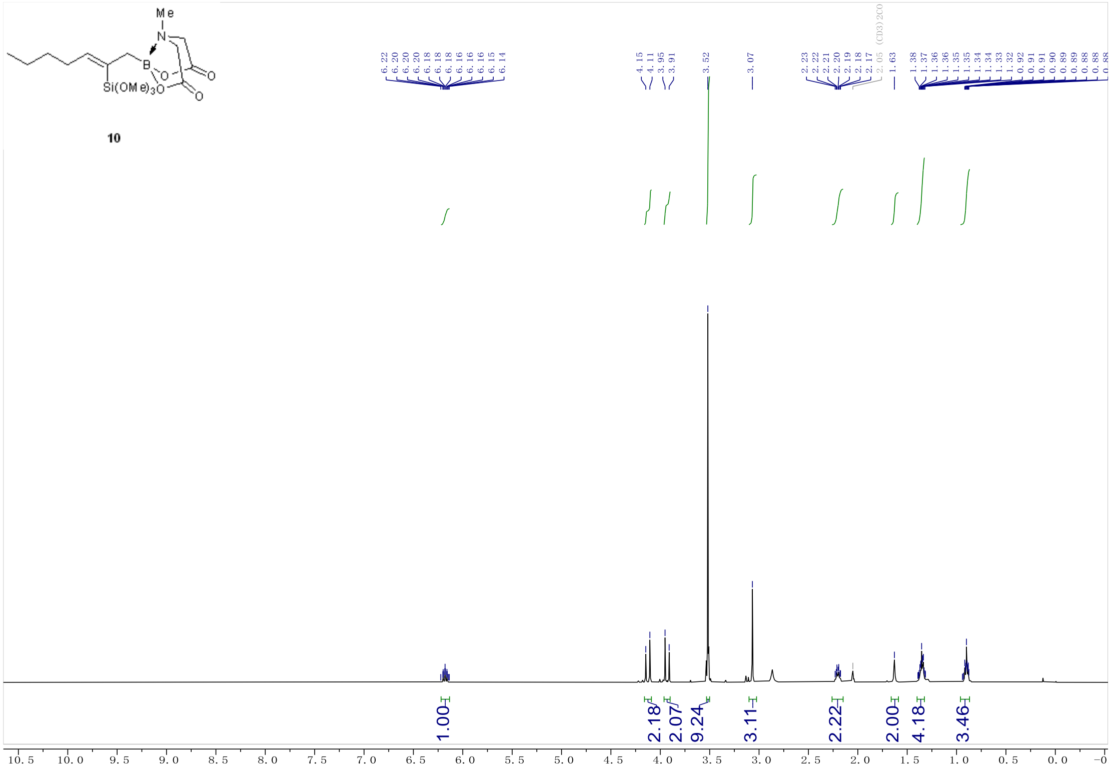


## $^{13}$C NMR of 10 (101 MHz, Acetone-$d_6$)

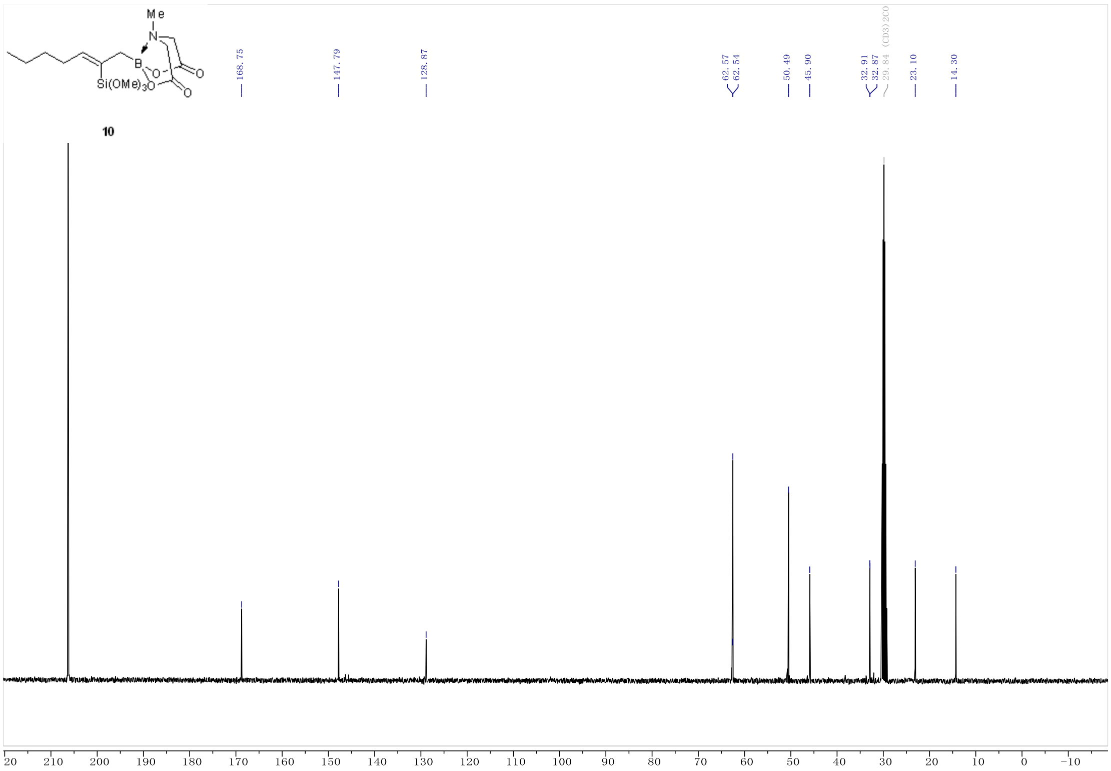

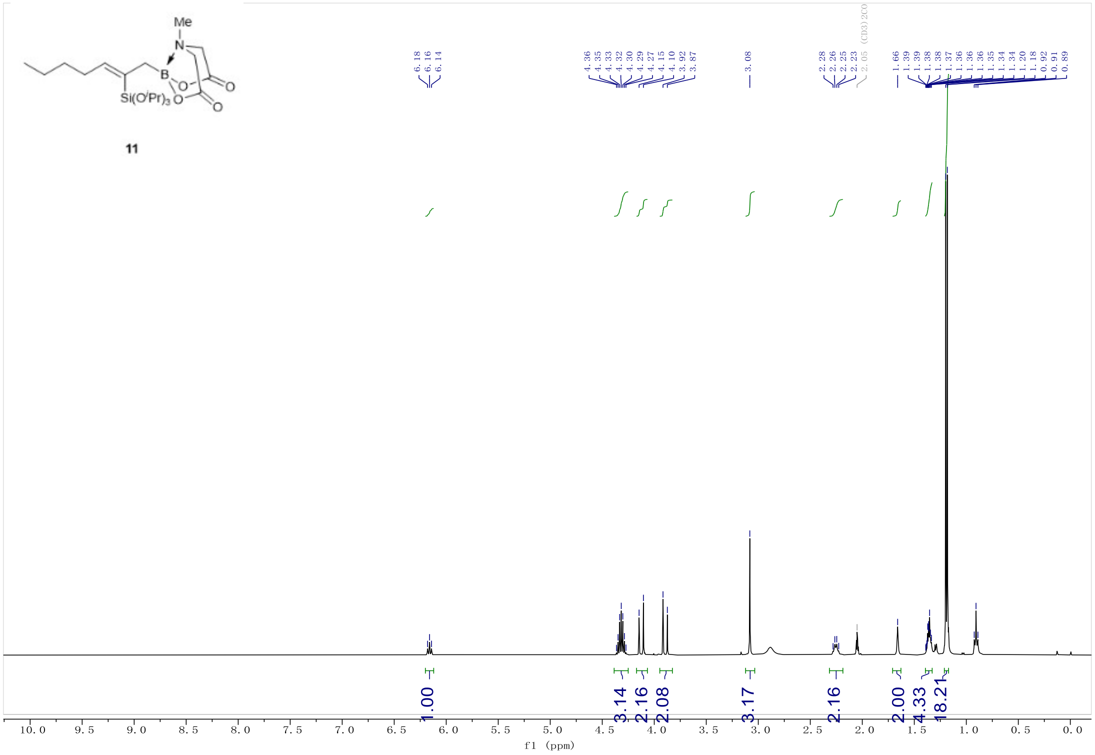

## $^{13}C$ NMR of 11 (101 MHz, Acetone-$d_6$)

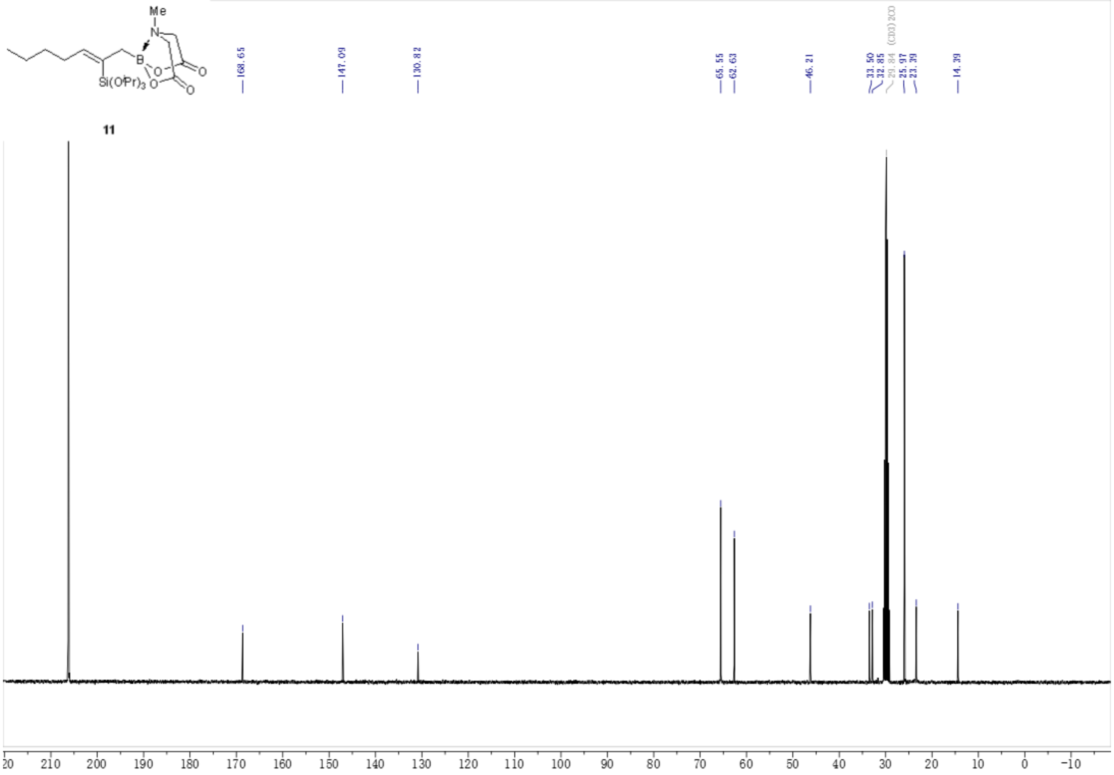


## $^{1}H$ NMR of 12 (500 MHz, Acetone-$d_6$)

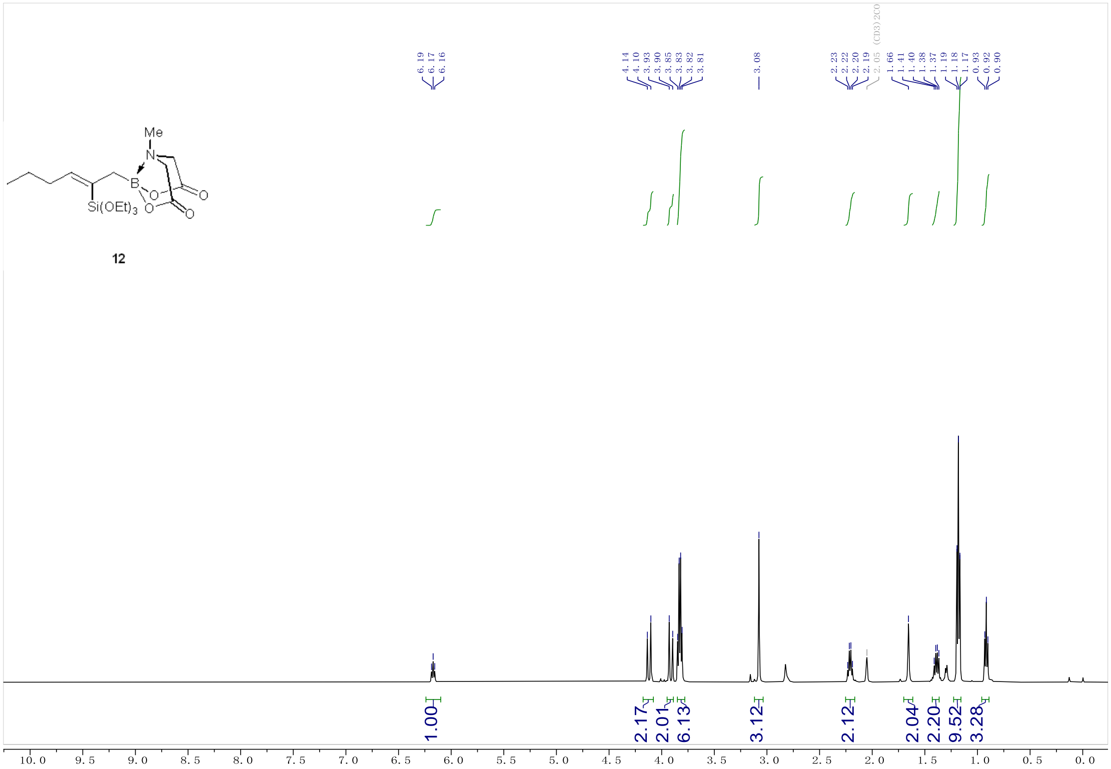

## $^{13}C$ NMR of 12 (126 MHz, Acetone-$d_6$)

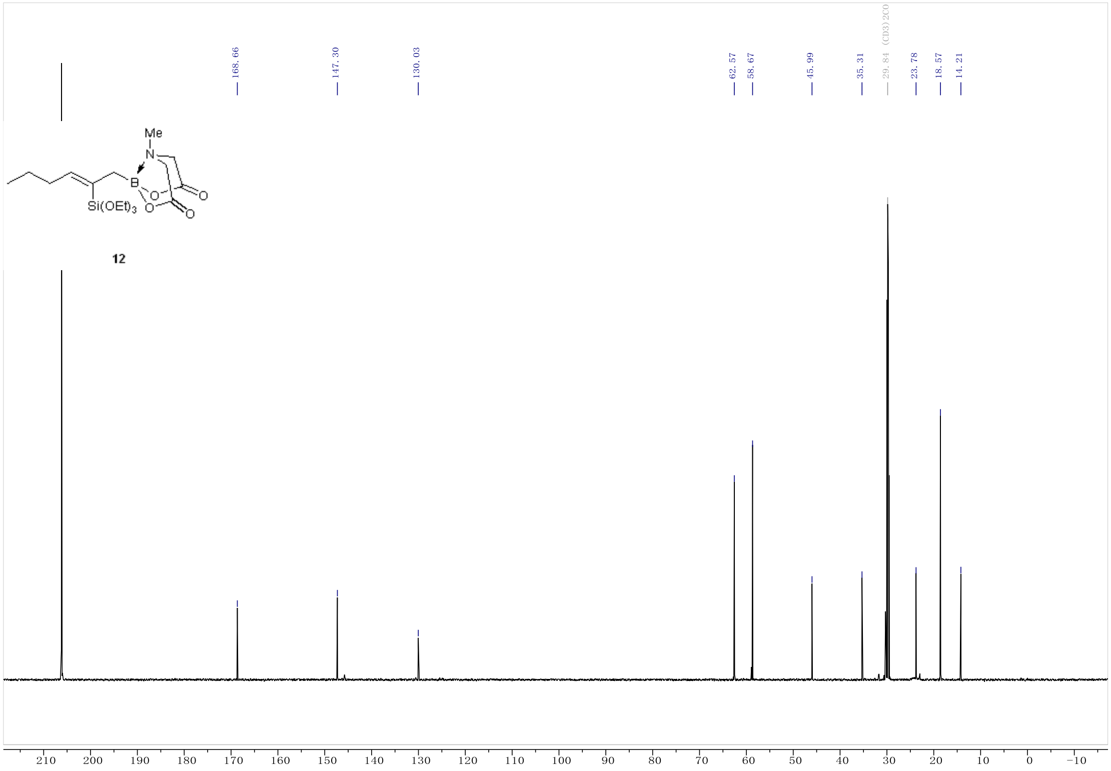


## $^{1}H$ NMR of 13 (500 MHz, Acetone-$d_6$)

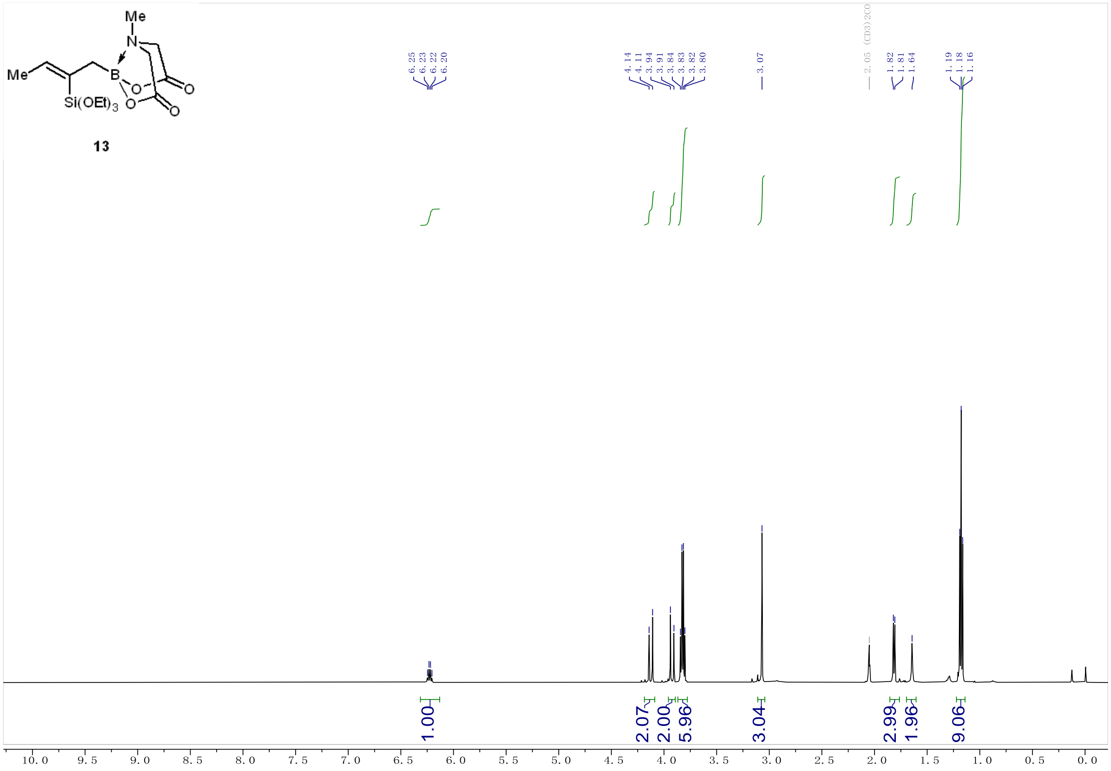

## $^{13}$C NMR of 13 (126 MHz, Acetone-$d_6$)

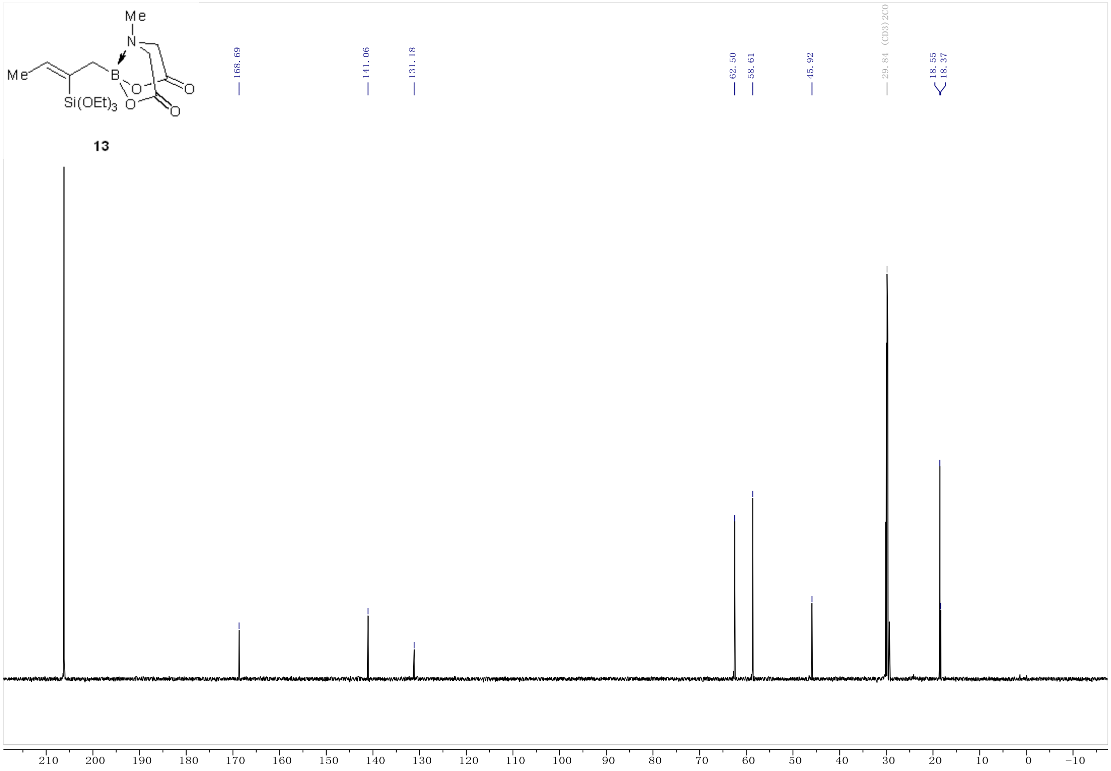


## $^{11}$B NMR of 13 (128 MHz, Acetone-$d_6$)

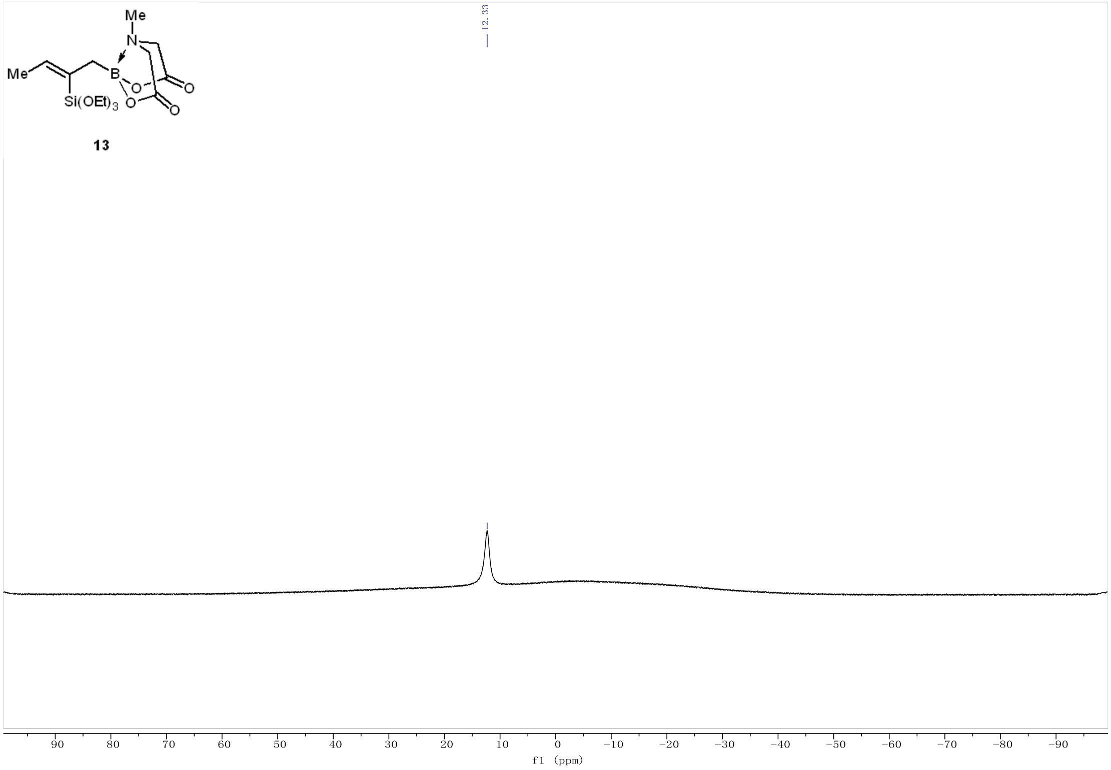

### $^{1}$H NMR of 14 (500 MHz, Acetone-$d_6$)

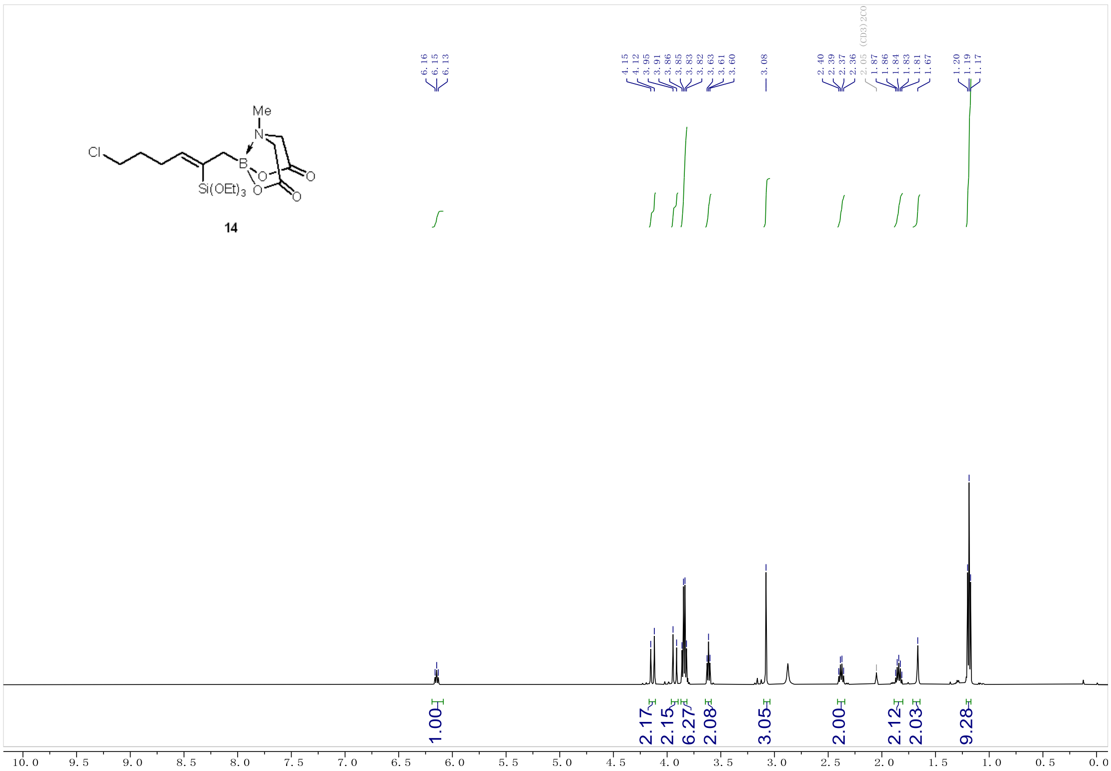

### $^{13}$C NMR of 14 (126 MHz, Acetone-$d_6$)

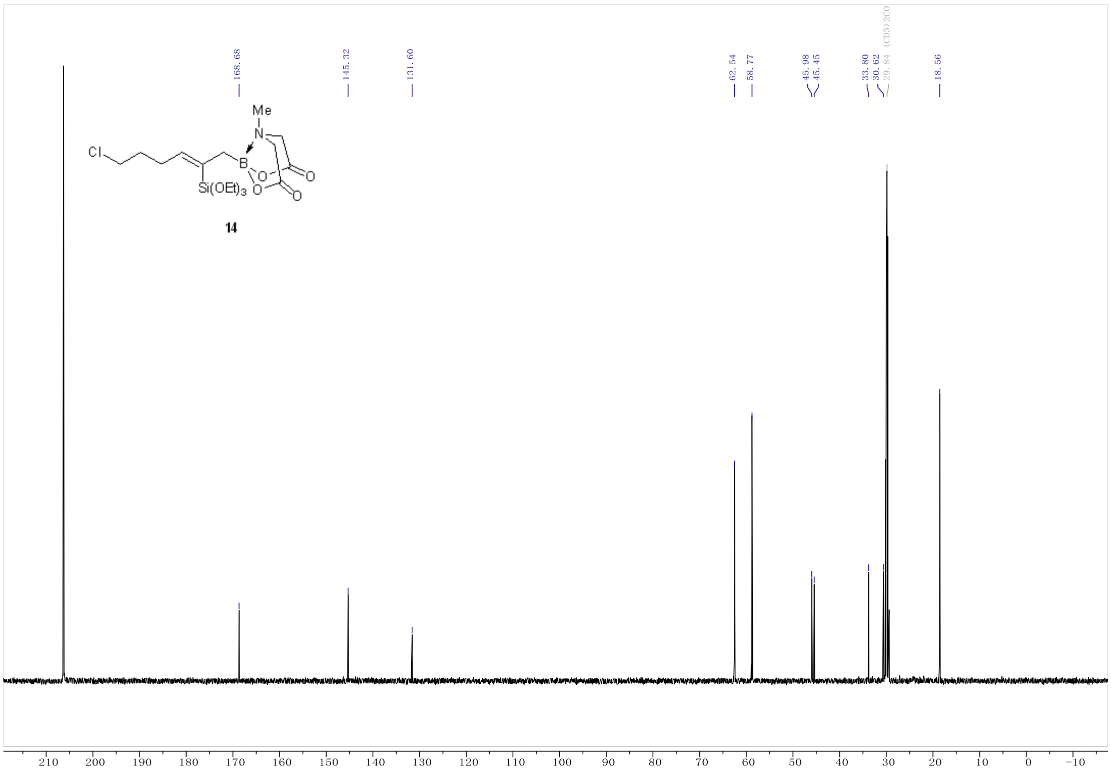

**$^{1}$H NMR of 15 (400 MHz, DMSO-$d_6$)**

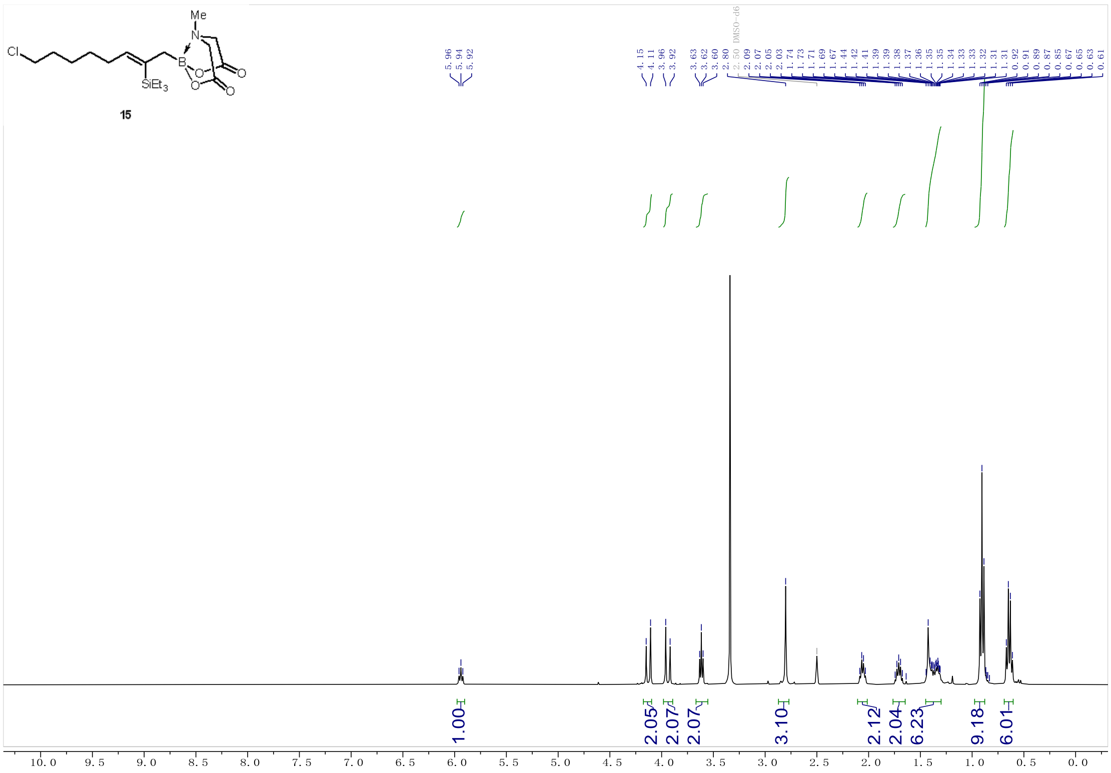

**$^{13}$C NMR of 15 (101 MHz, DMSO-$d_6$)**

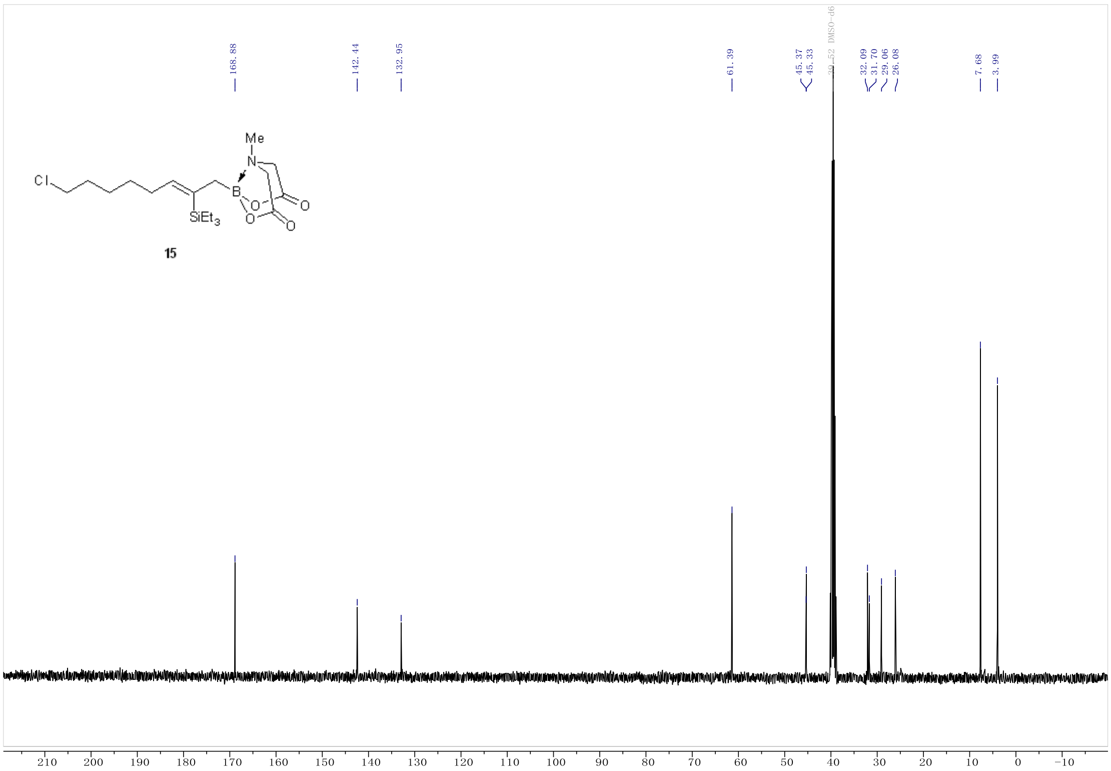

**NOE of 15**

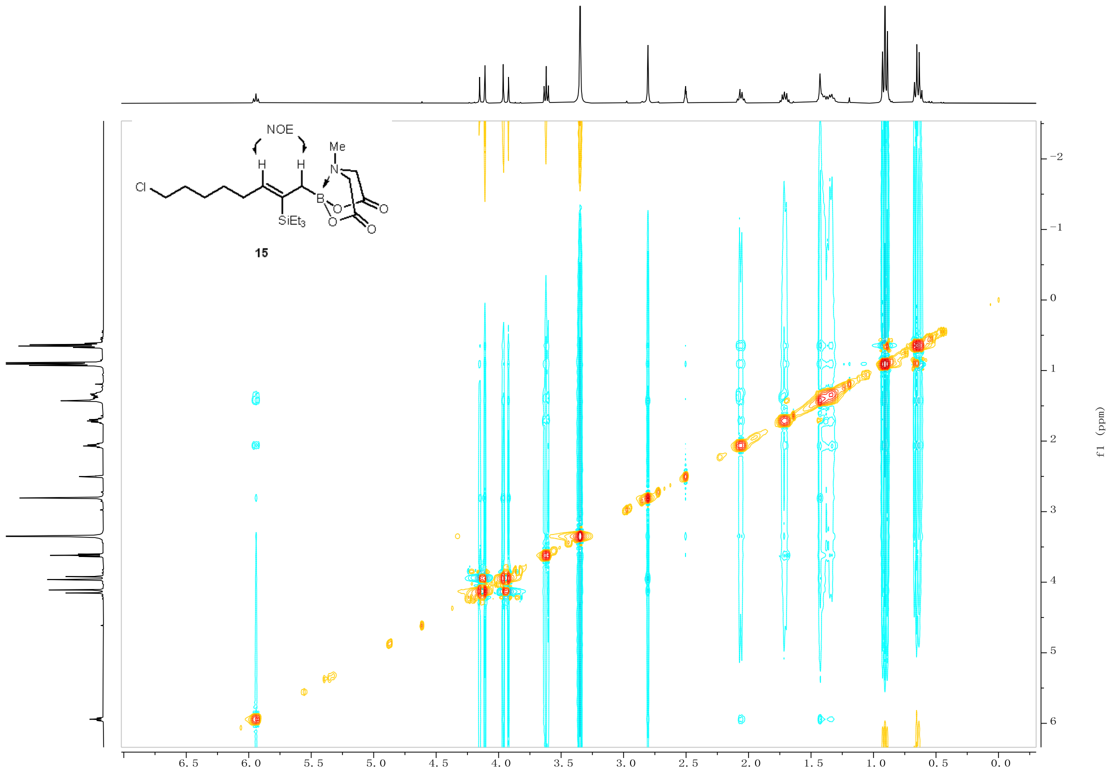


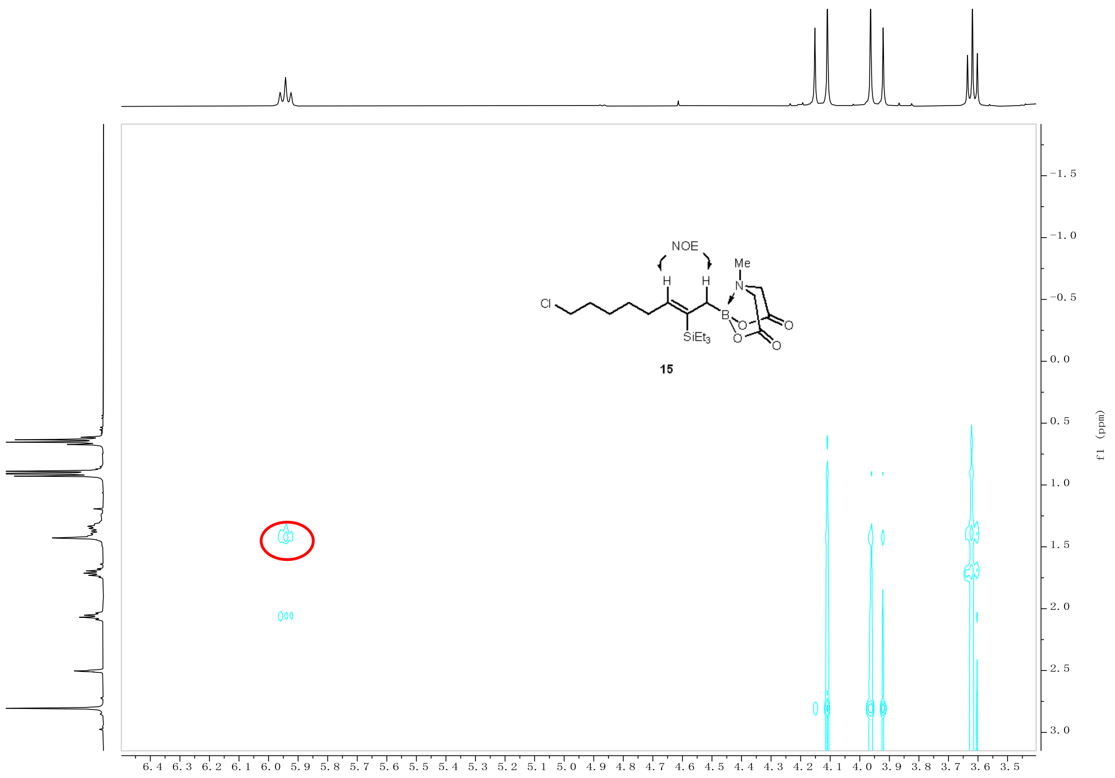


**$^1$H NMR of 16 (400 MHz, Acetone-$d_6$)**

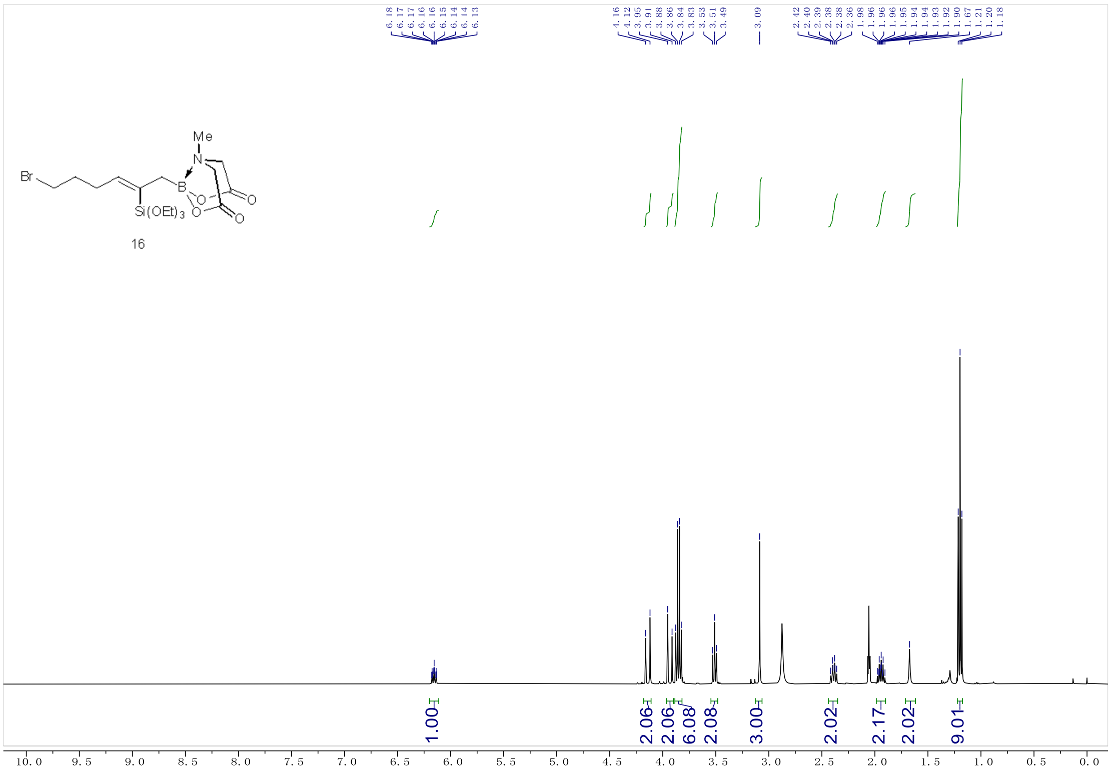


**$^{13}$C NMR of 16 (101 MHz, Acetone-$d_6$)**

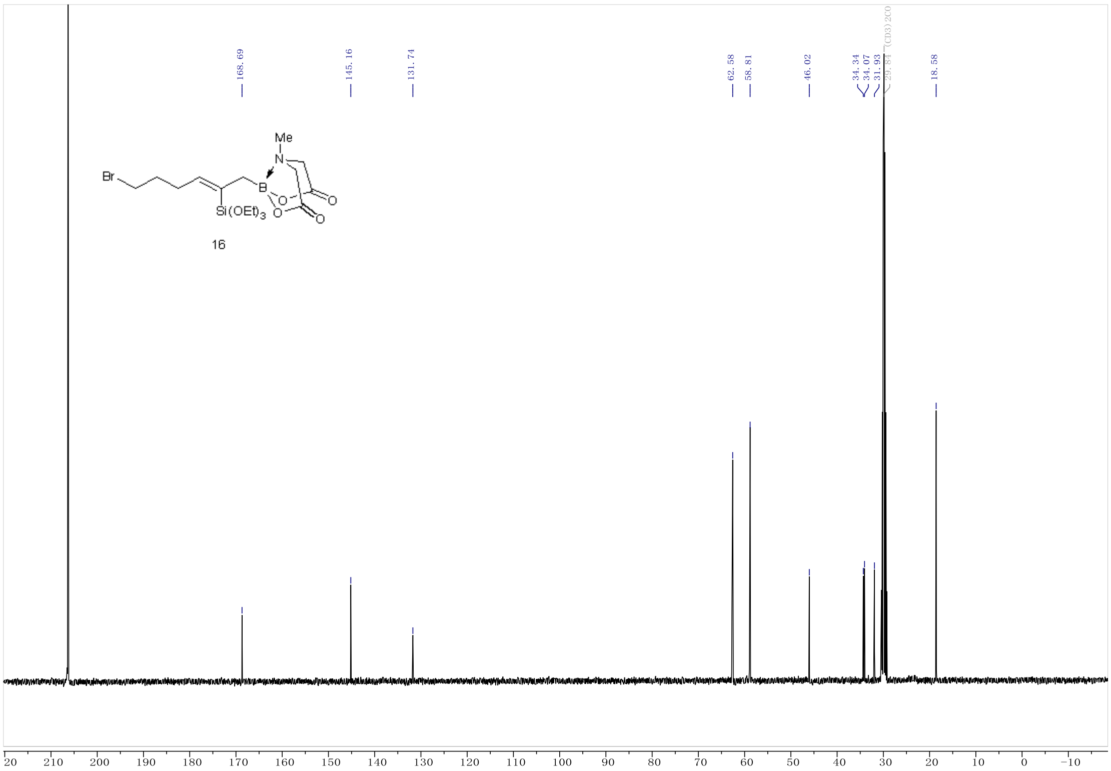

## $^{1}$H NMR of 17 (400 MHz, DMSO-$d_6$)

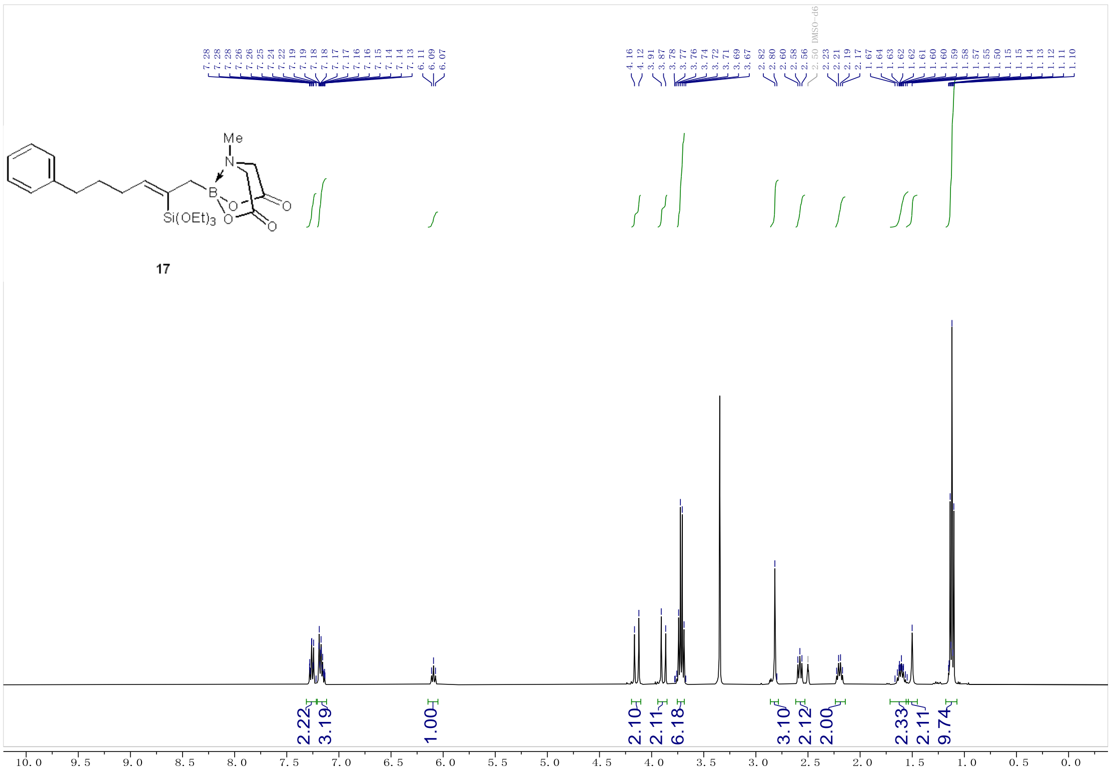


## $^{13}$C NMR of 17 (101 MHz, DMSO-$d_6$)

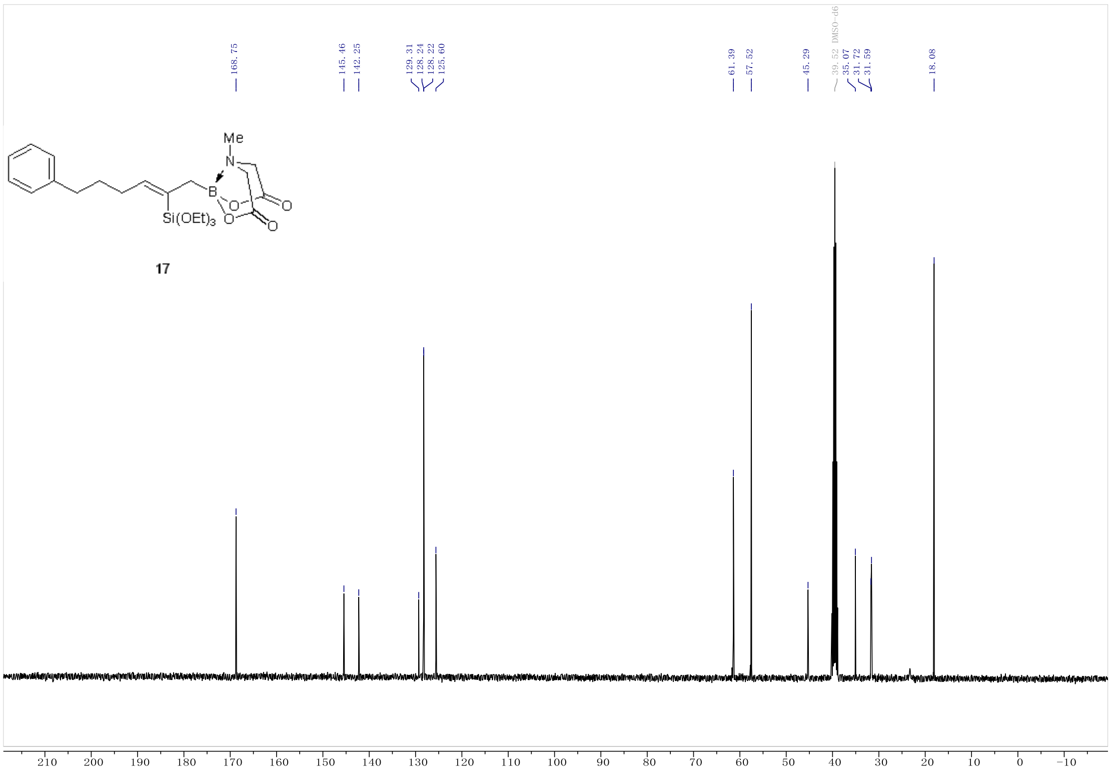

# NOE of 17

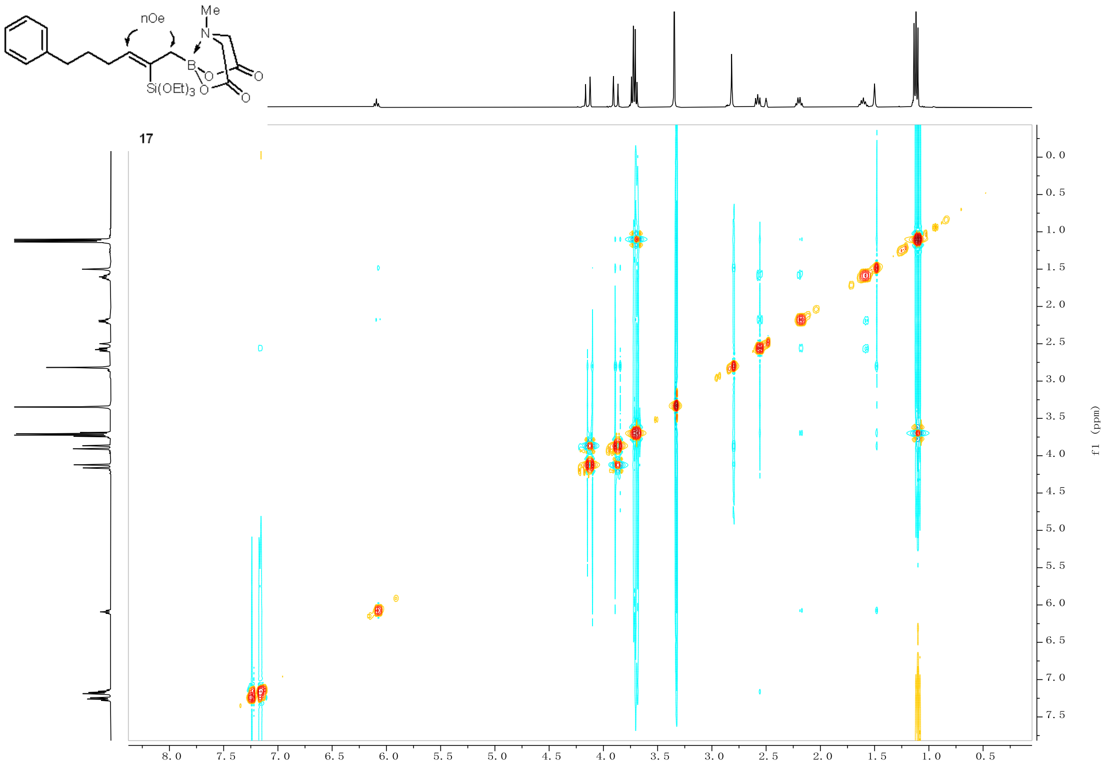
nOe
Me
N
B
O
O
O
O
Si(OEt)3
17
f1 (ppm)

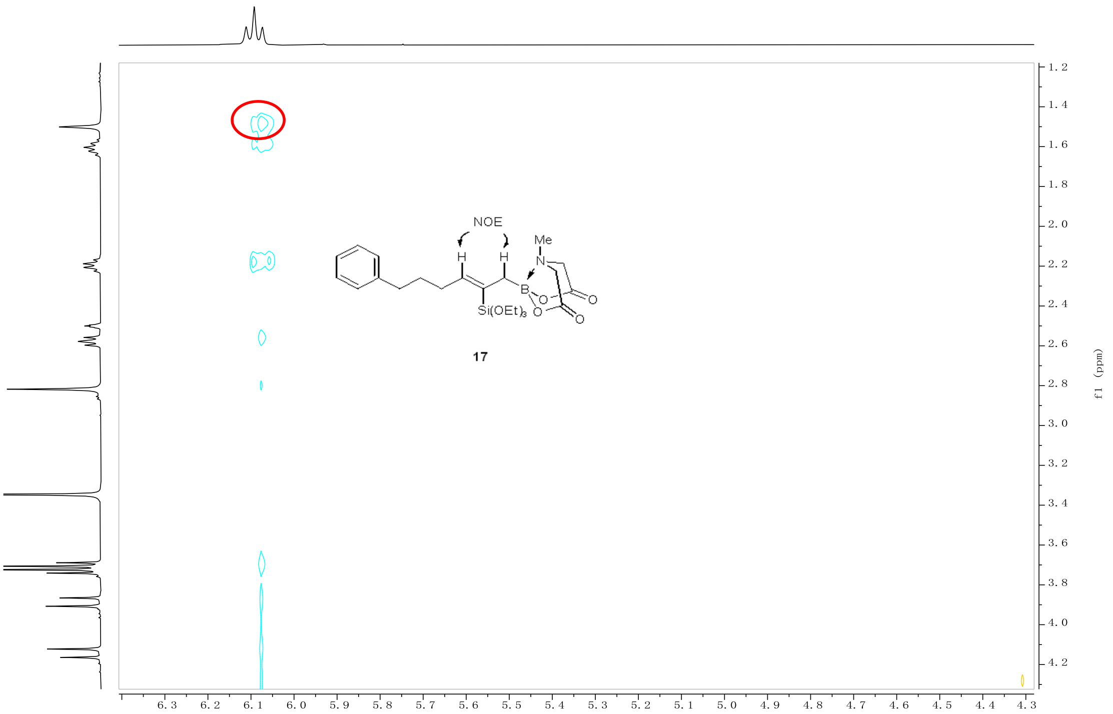
NOE
H
H
Me
N
B
O
O
O
O
Si(OEt)3
17
f1 (ppm)

## $^{1}$H NMR of 18 (400 MHz, $CDCl_3$)

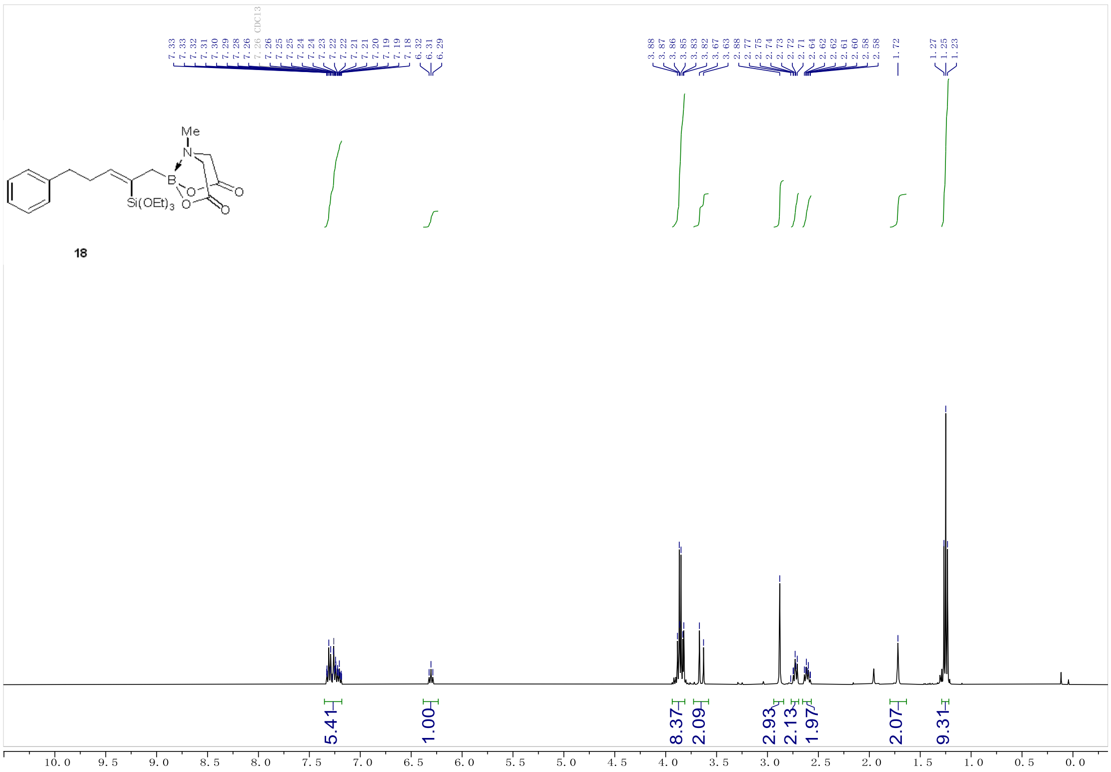

## $^{13}$C NMR of 18 (101 MHz, $CDCl_3$)

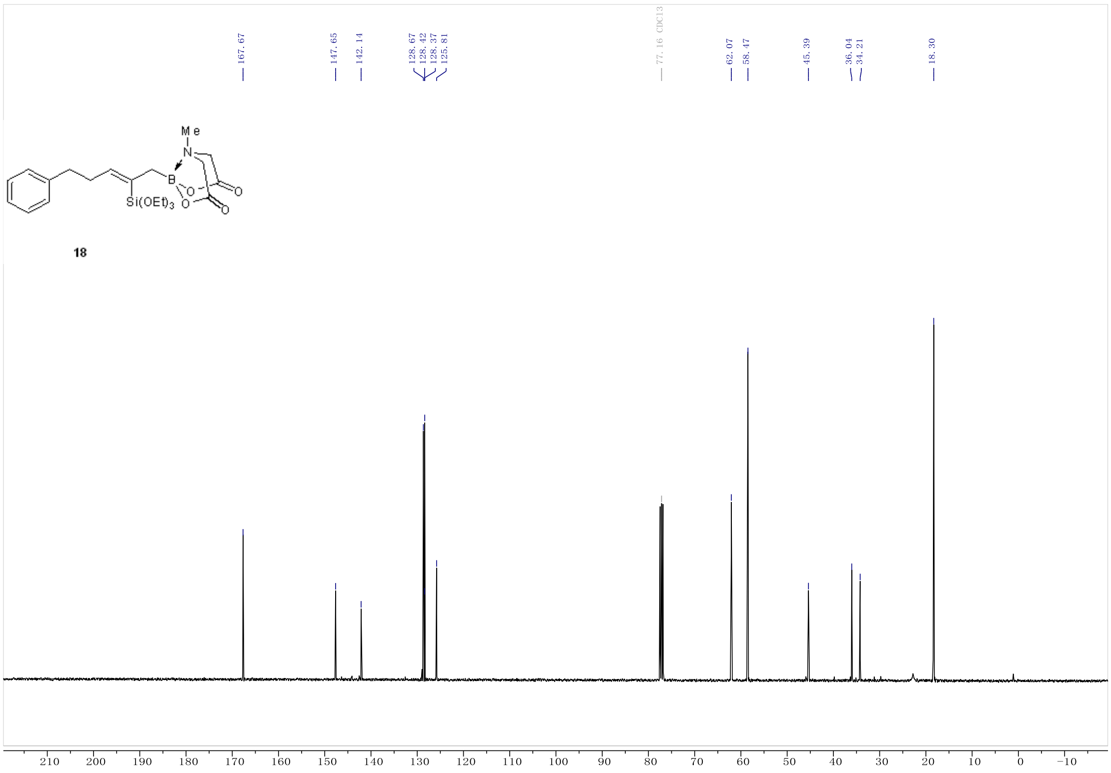

## $^{1}$H NMR of 19 (400 MHz, $CD_3CN$)

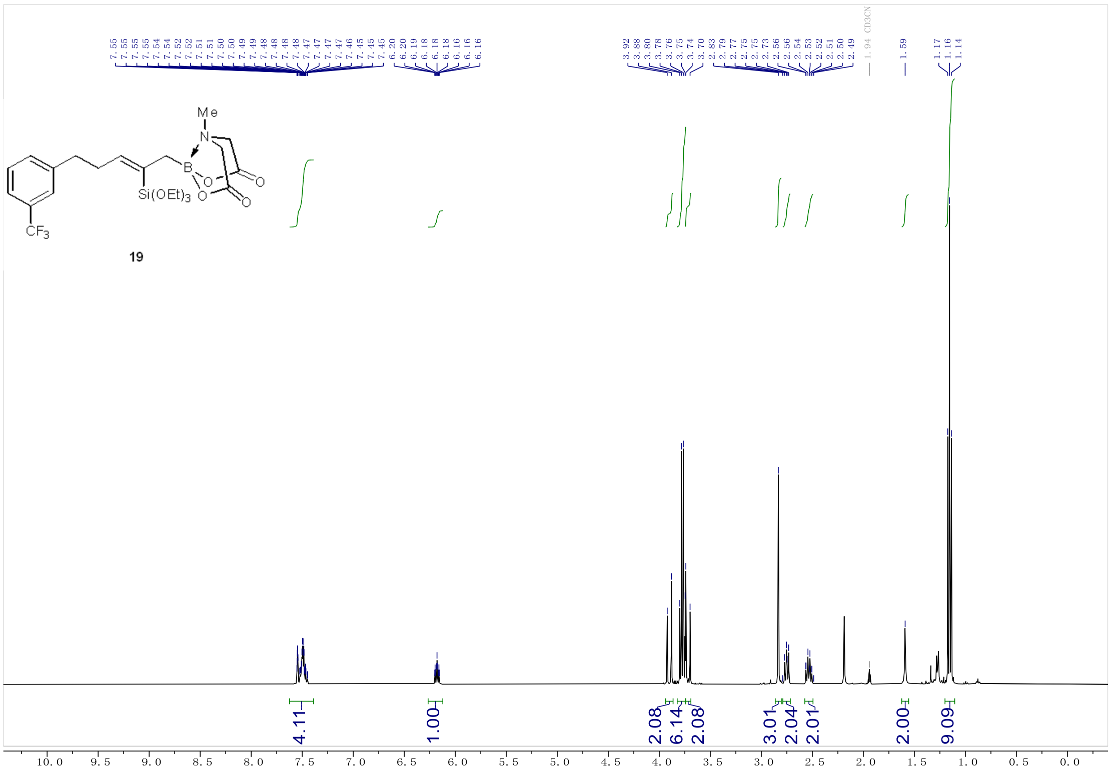

## $^{13}$C NMR of 19 (101 MHz, $CD_3CN$)

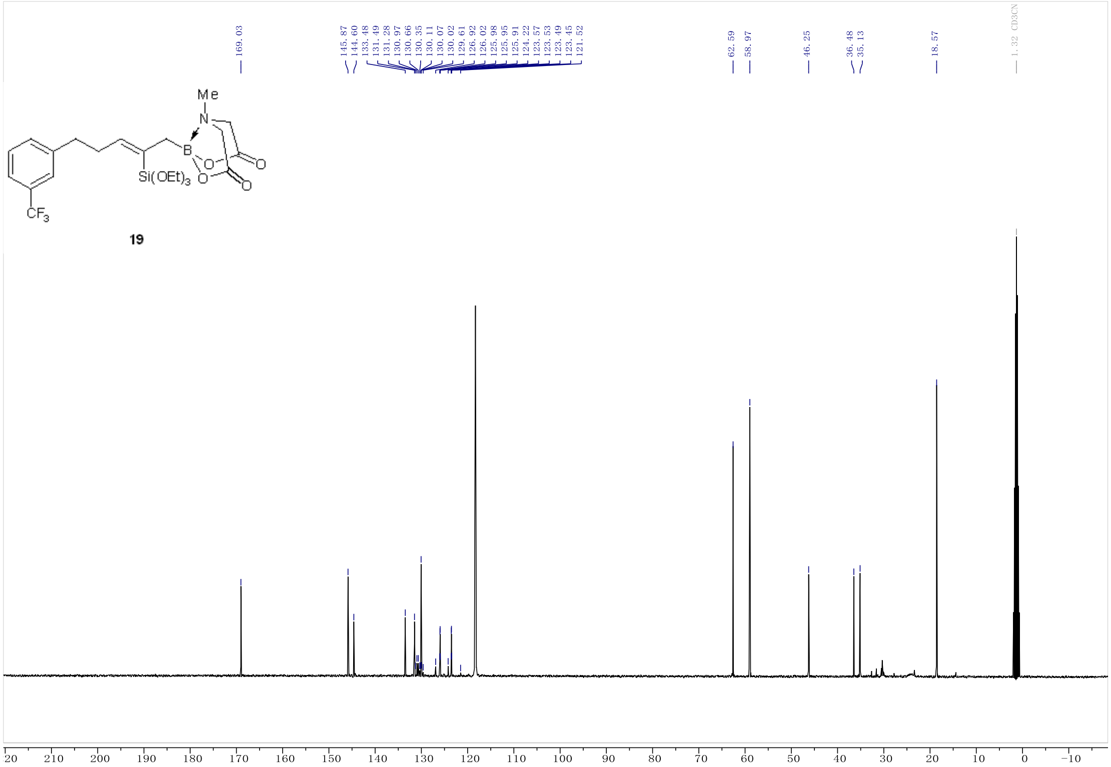

## $^{19}$F NMR of 19 (376 MHz, $CD_3CN$)

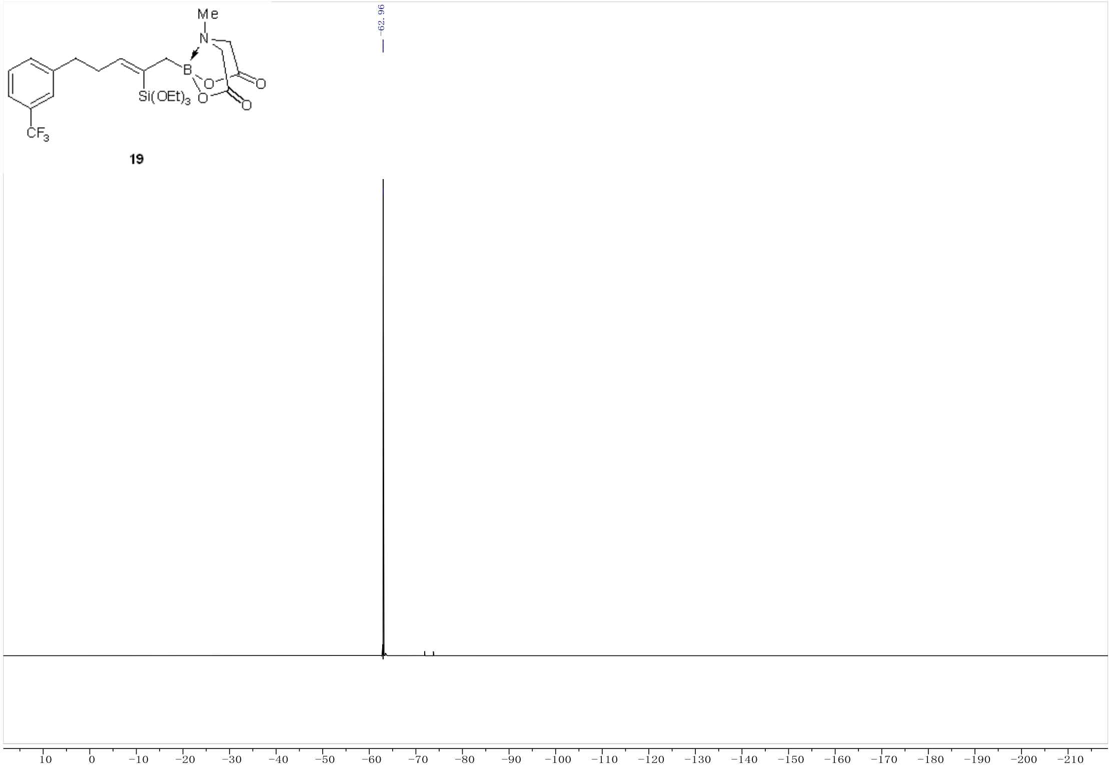

## $^{1}$H NMR of 20 (400 MHz, DMSO-$d_6$)

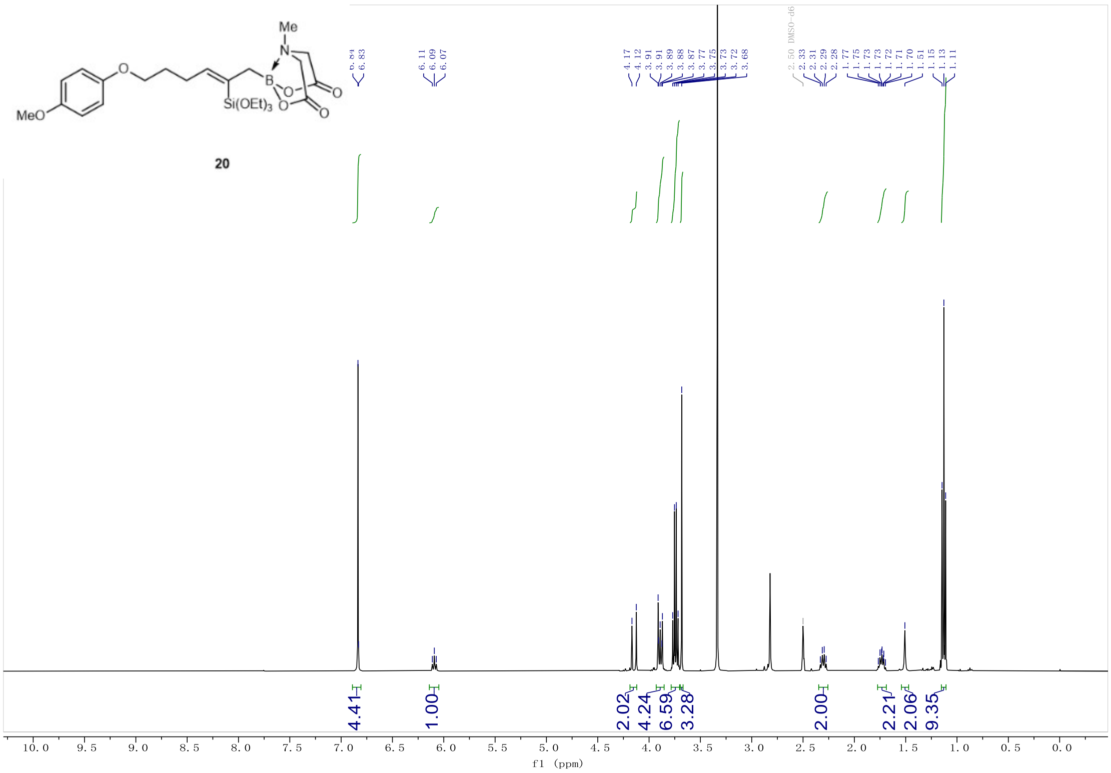

## $^{13}C$ NMR of 20 (101 MHz, DMSO-$d_6$)

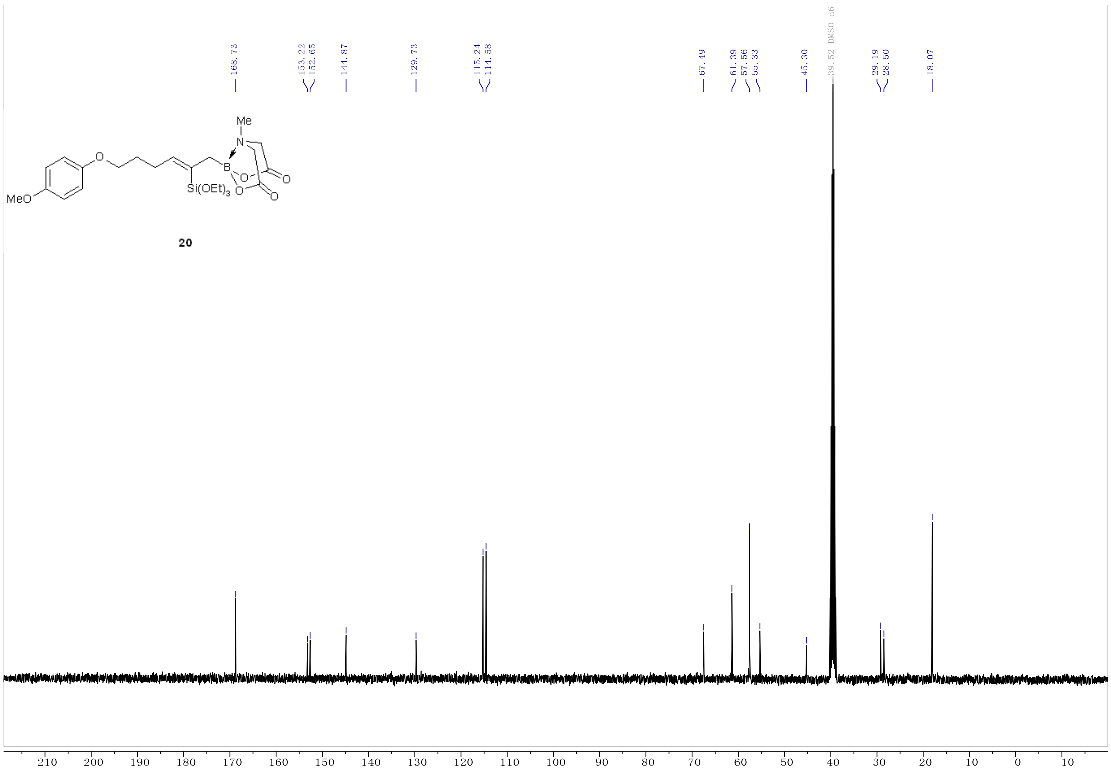


## $^{1}H$ NMR of 21 (400 MHz, DMSO-$d_6$)

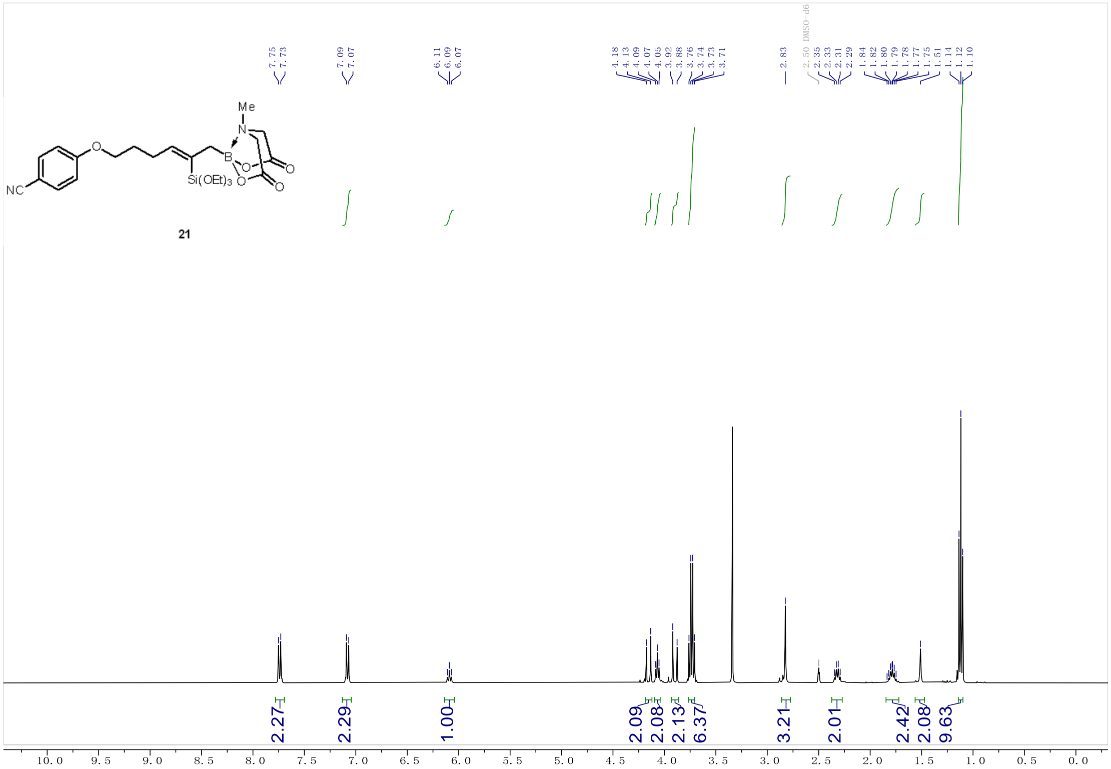

## $^{13}C$ NMR of 21 (101 MHz, DMSO-$d_6$)

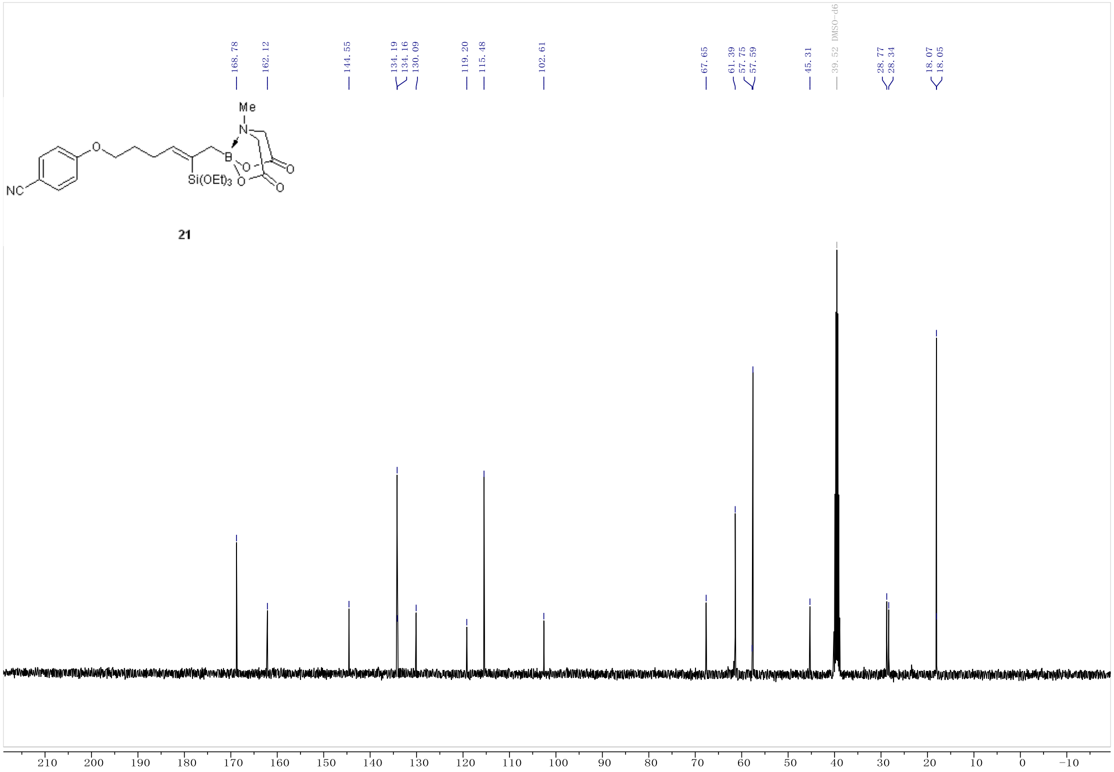


## $^{1}H$ NMR of 22 (500 MHz, DMSO-$d_6$)

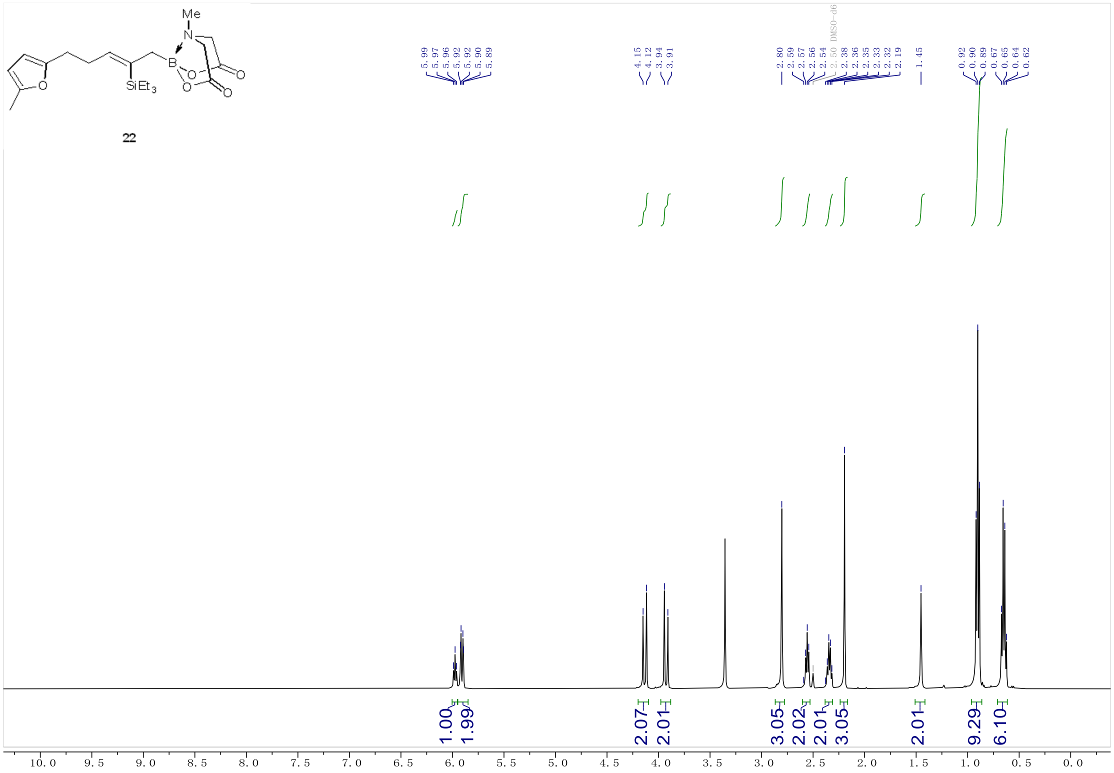

**$^{13}$C NMR of 22 (126 MHz, DMSO-$d_6$)**

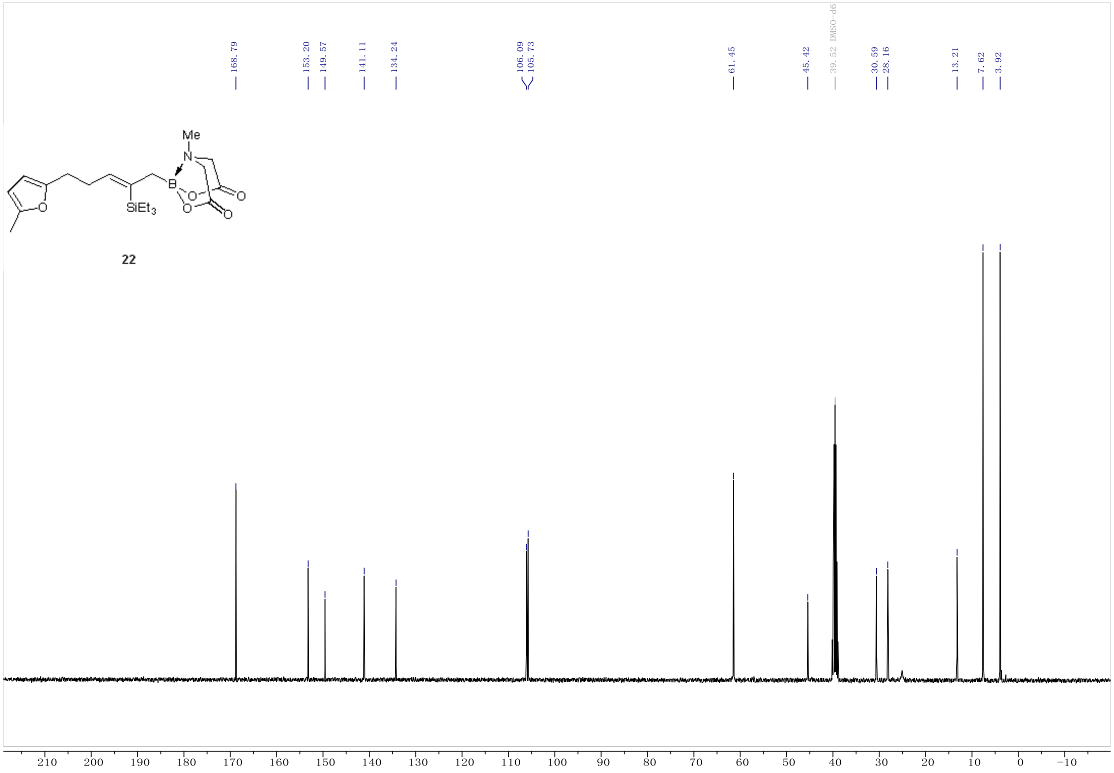


**$^{11}$B NMR of 22 (128 MHz, Acetone-$d_6$)**

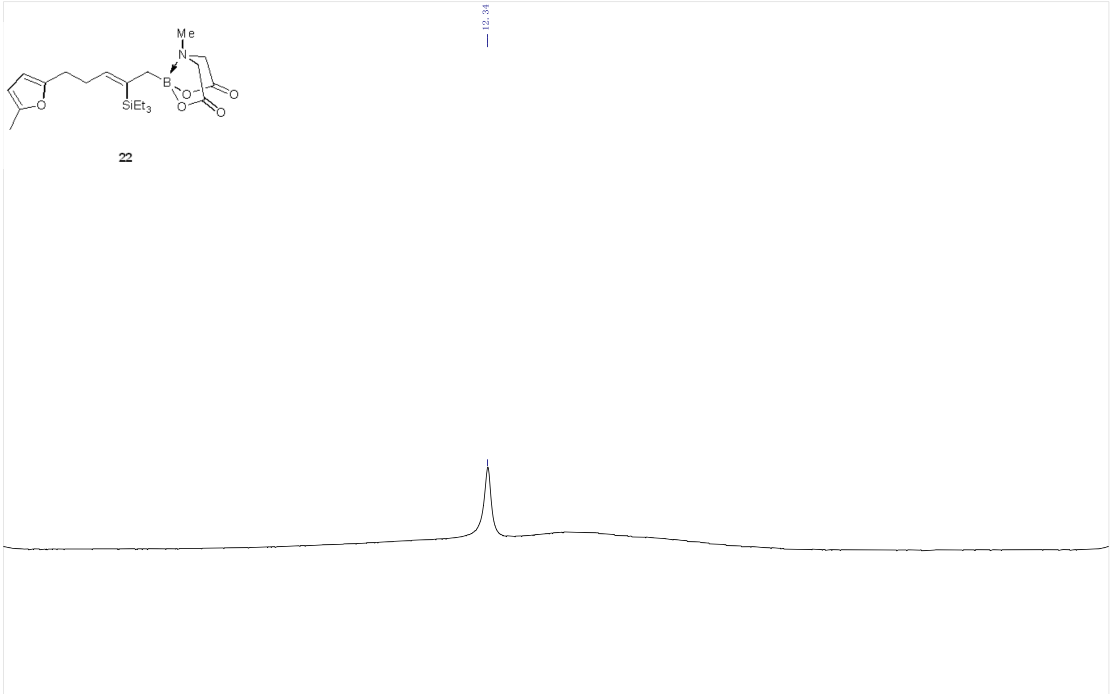


**COSY of 22**

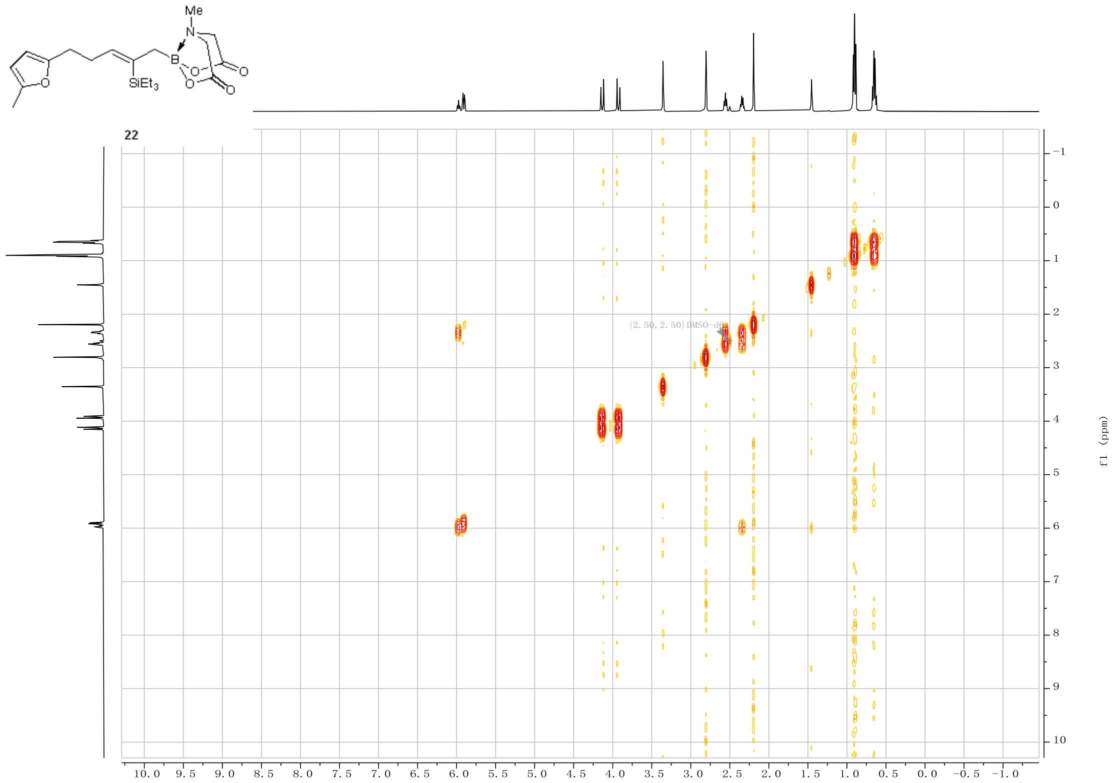


**HMBC of 22**

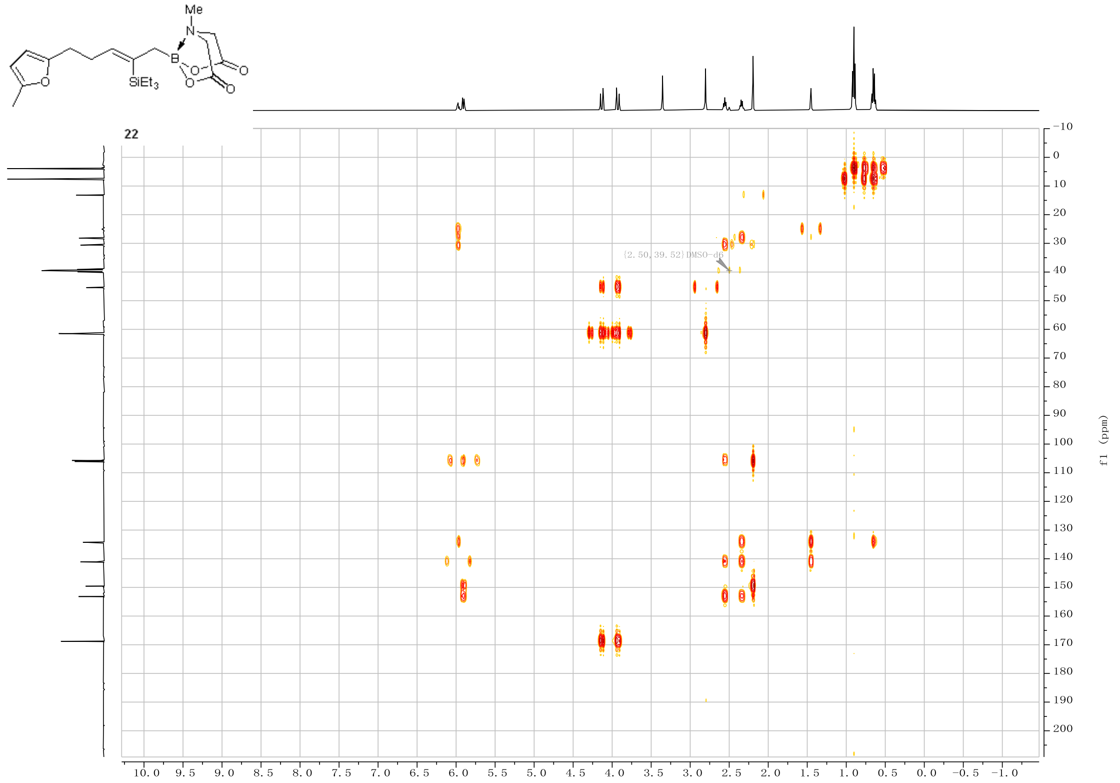


**HSQC of 22**

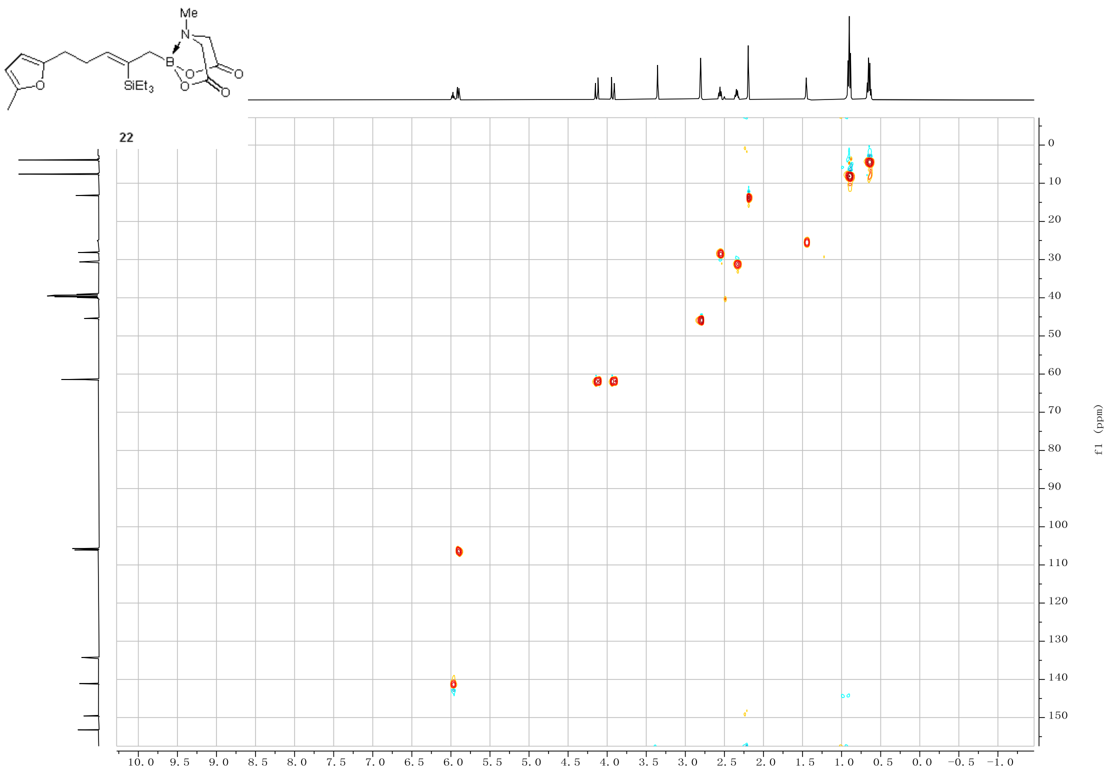


## NOE of 22

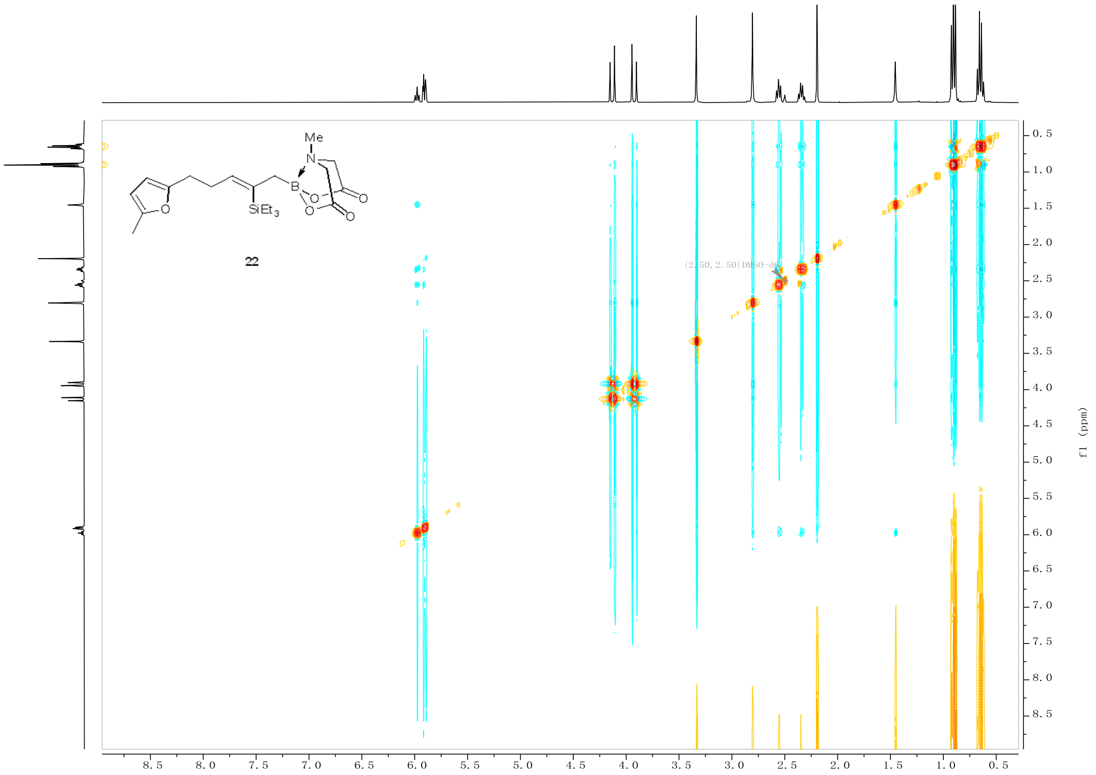

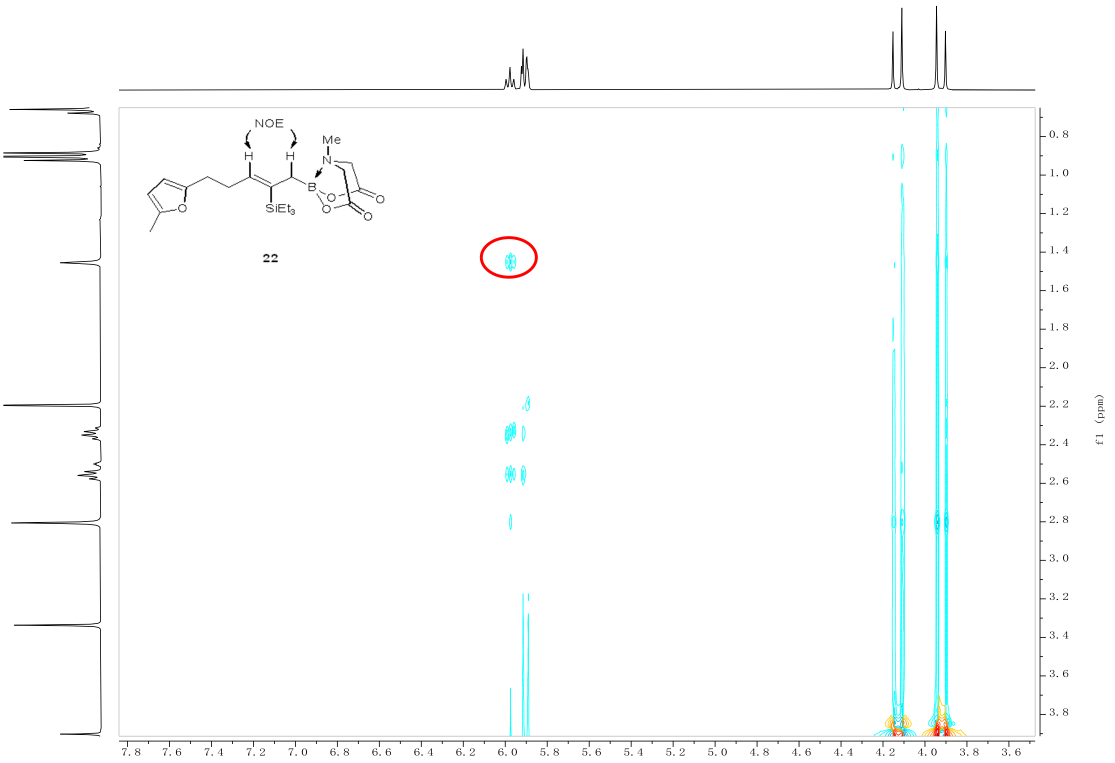


**$^{1}$H NMR of 23 (400 MHz, Acetone-$d_6$)**

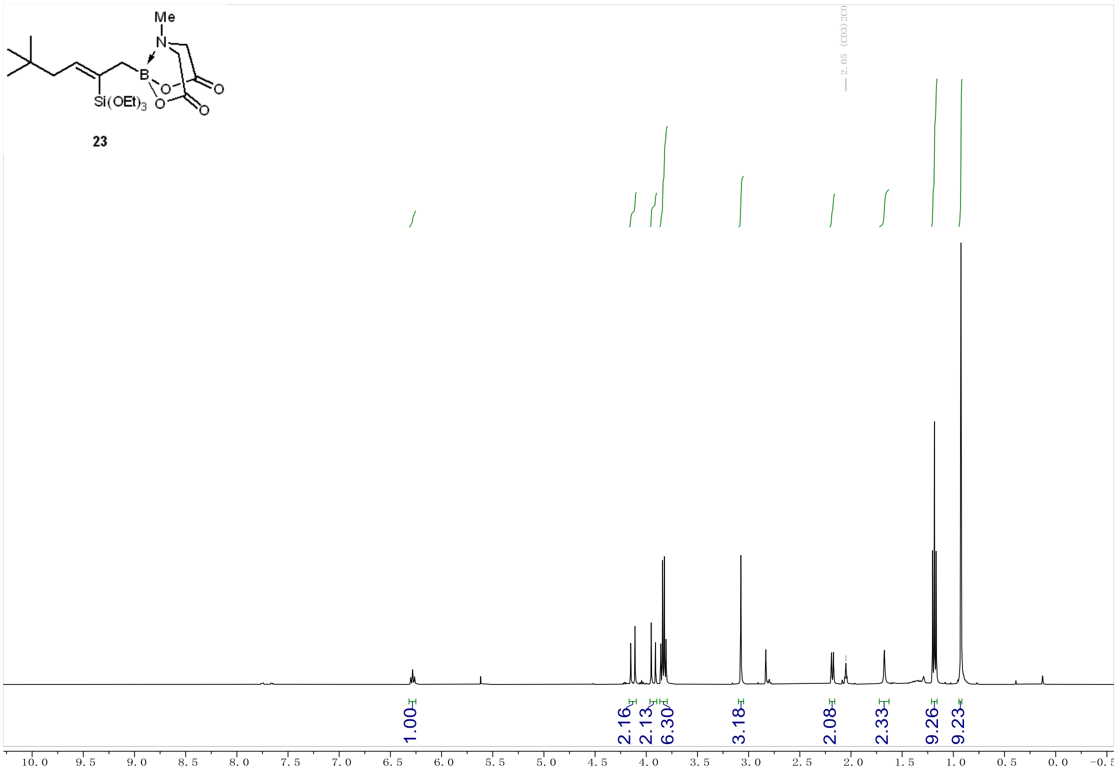

$^{13}$C NMR of 23 (101 MHz, DMSO-$d_6$)

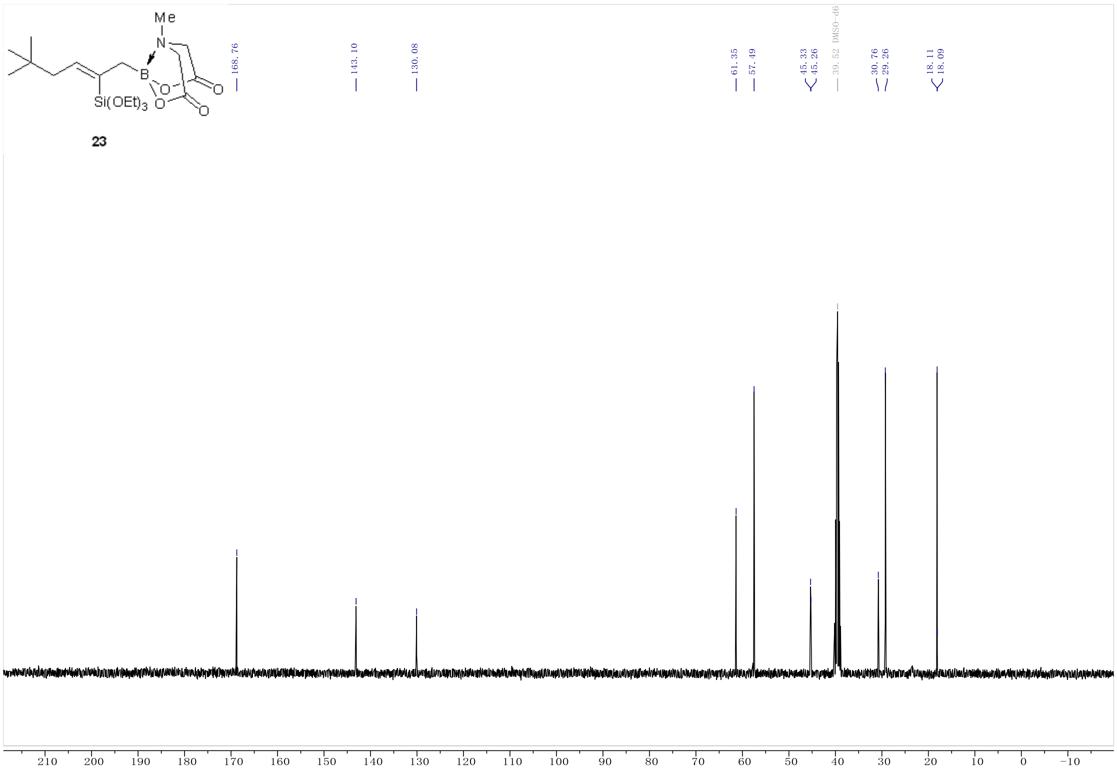


$^{11}$B NMR of 23 (128 MHz, Acetone-$d_6$)

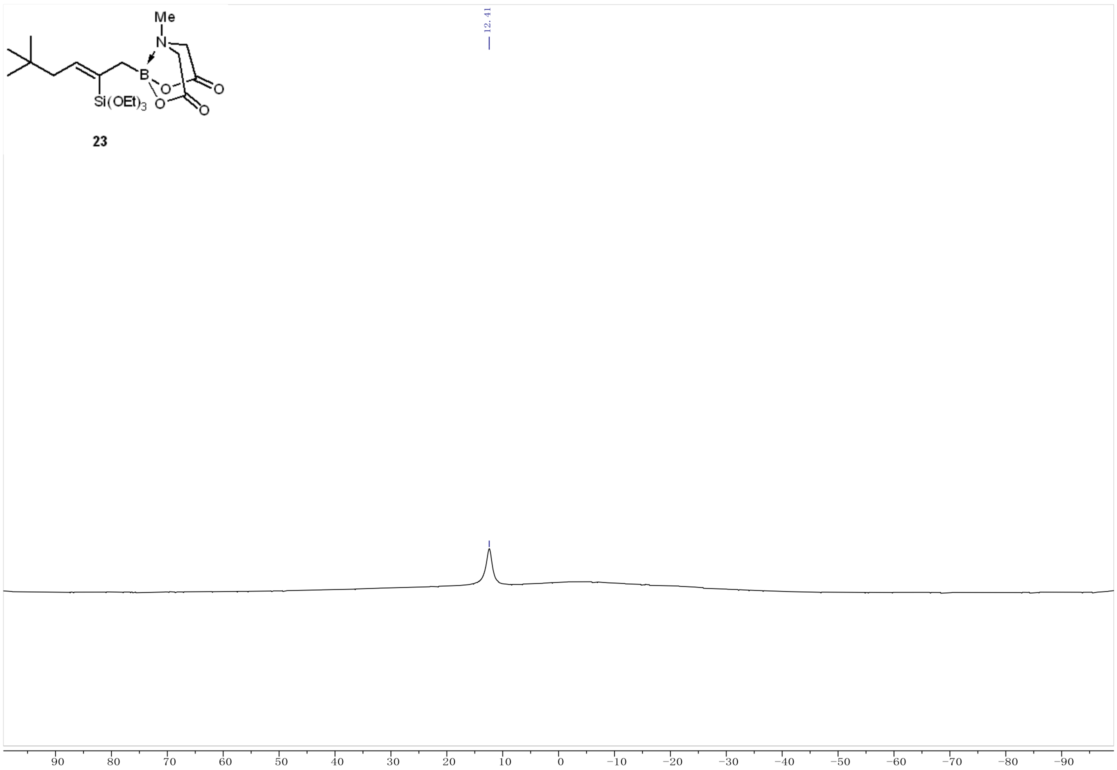


NOE of 23

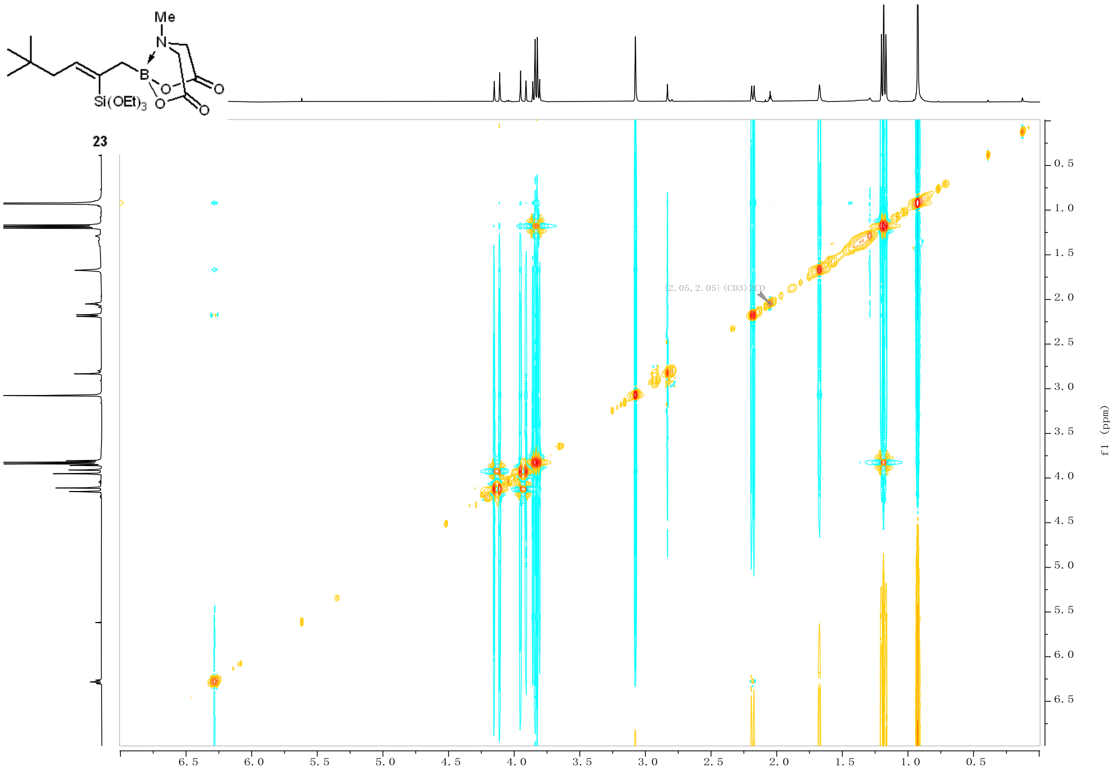


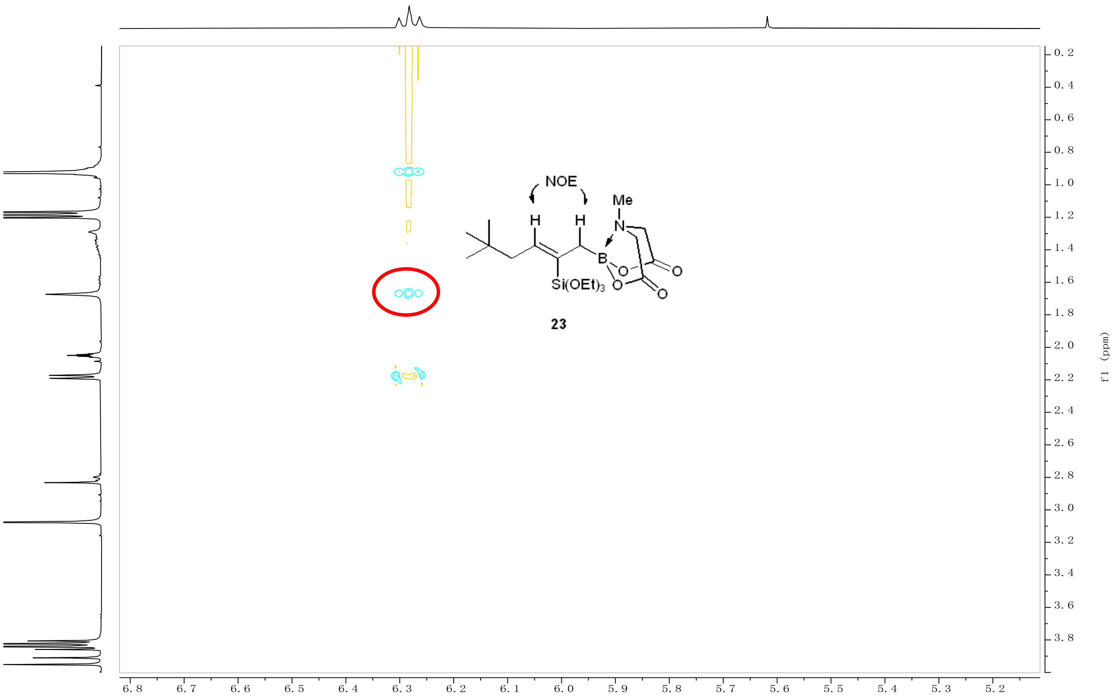


**$^{1}$H NMR of 24 (400 MHz, DMSO-$d_6$)**

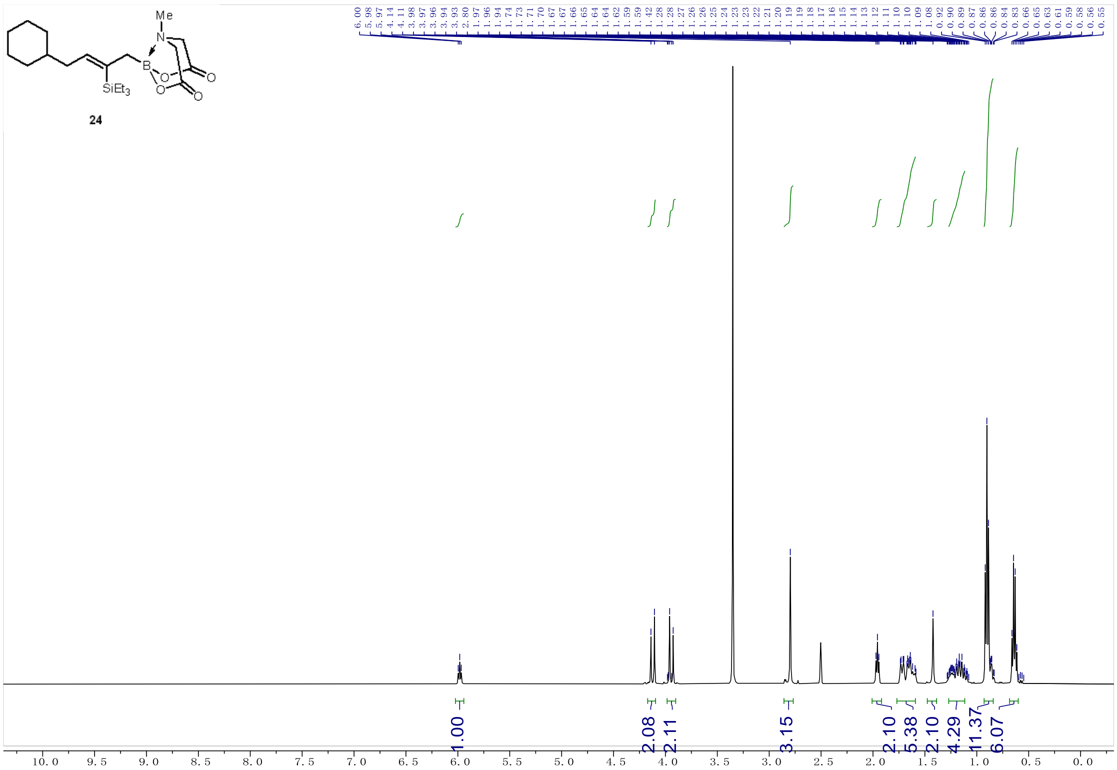

## $^{13}$C NMR of 24 (101 MHz, DMSO-$d_6$)

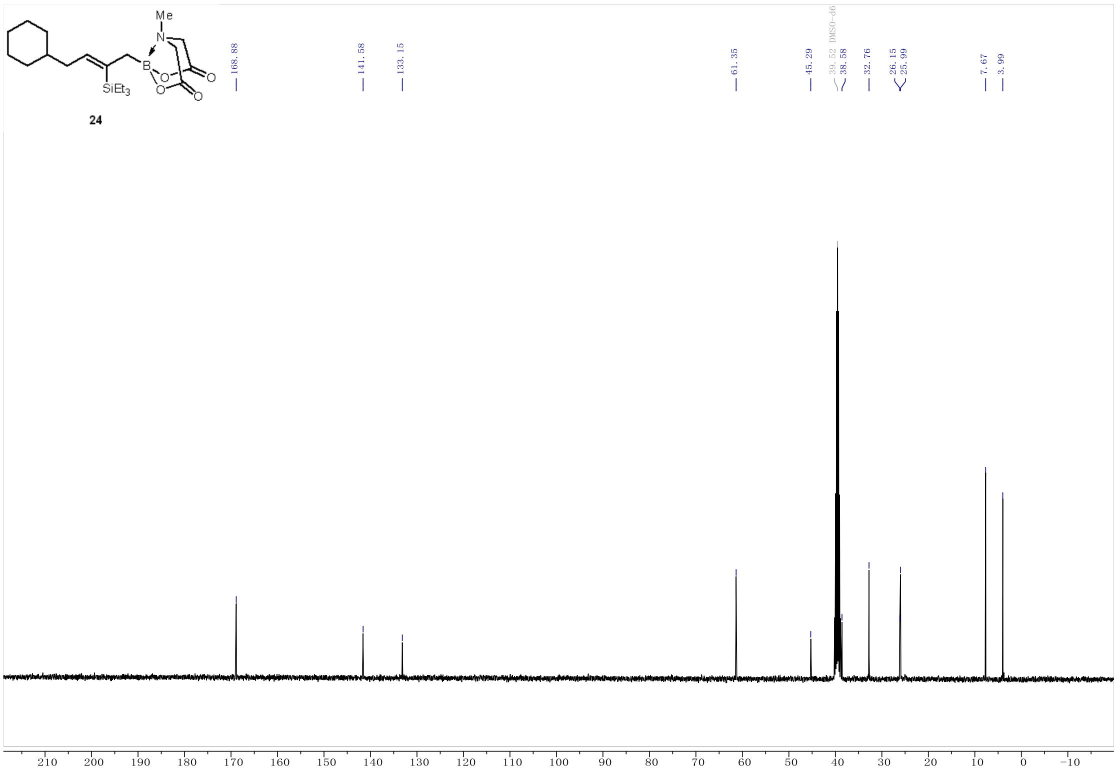

## $^1$H NMR of 25 (400 MHz, Acetone-$d_6$)

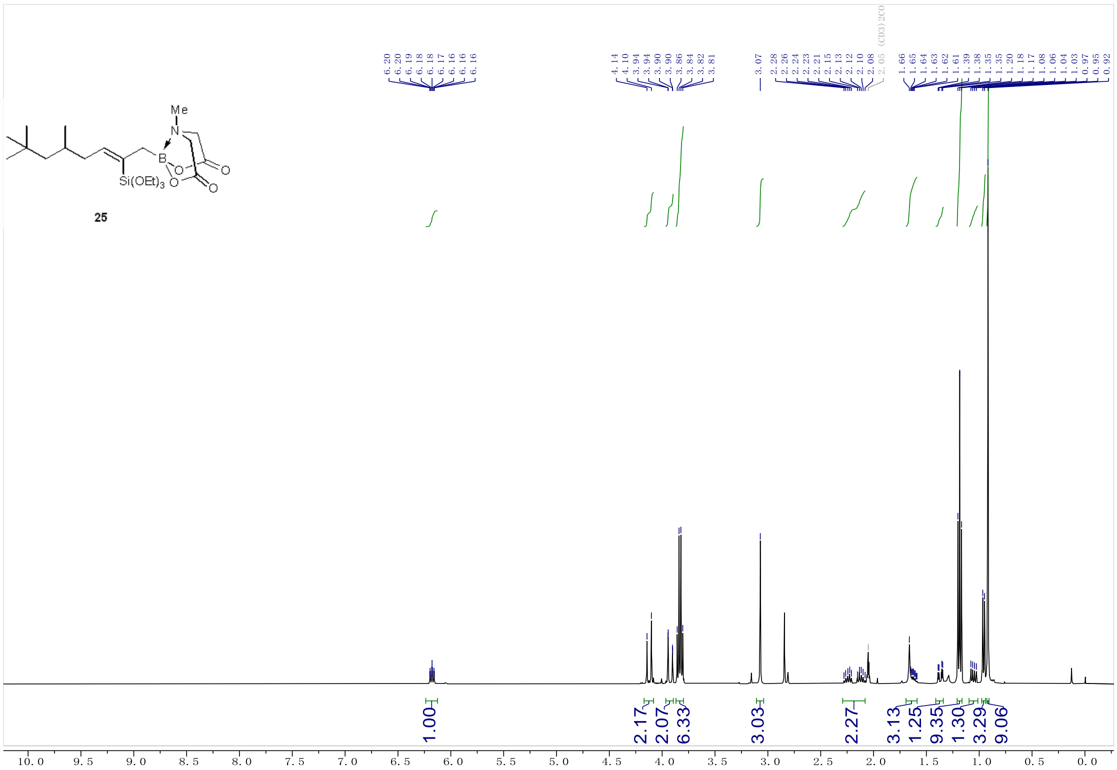

25

## $^{13}$C NMR of 25 (101 MHz, Acetone-$d_6$)

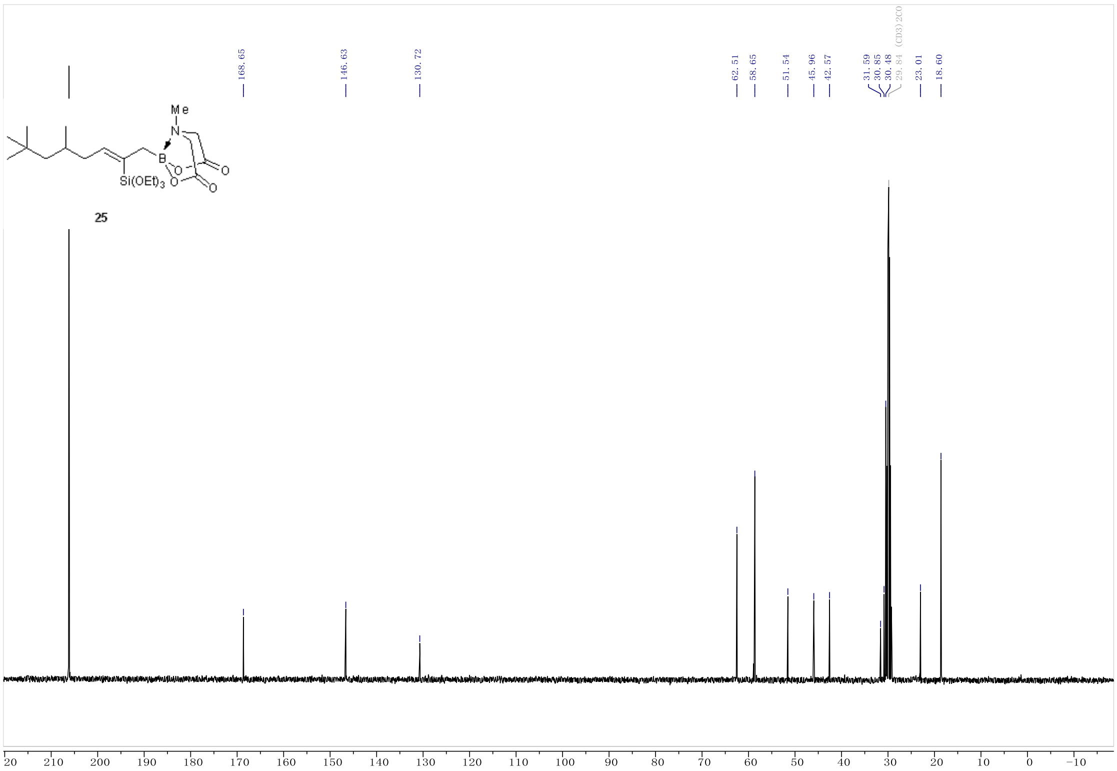

25

## $^{1}H$ NMR of 26 (500 MHz, DMSO-$d_6$)

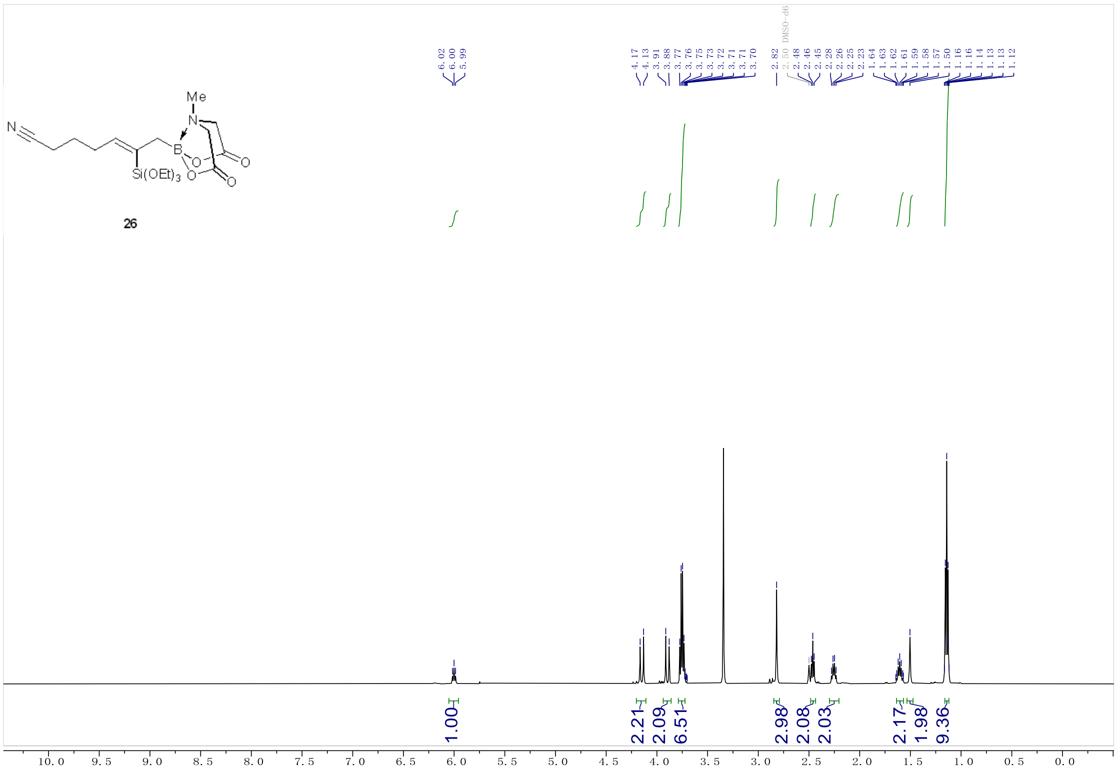


## $^{13}C$ NMR of 26 (126 MHz, DMSO-$d_6$)

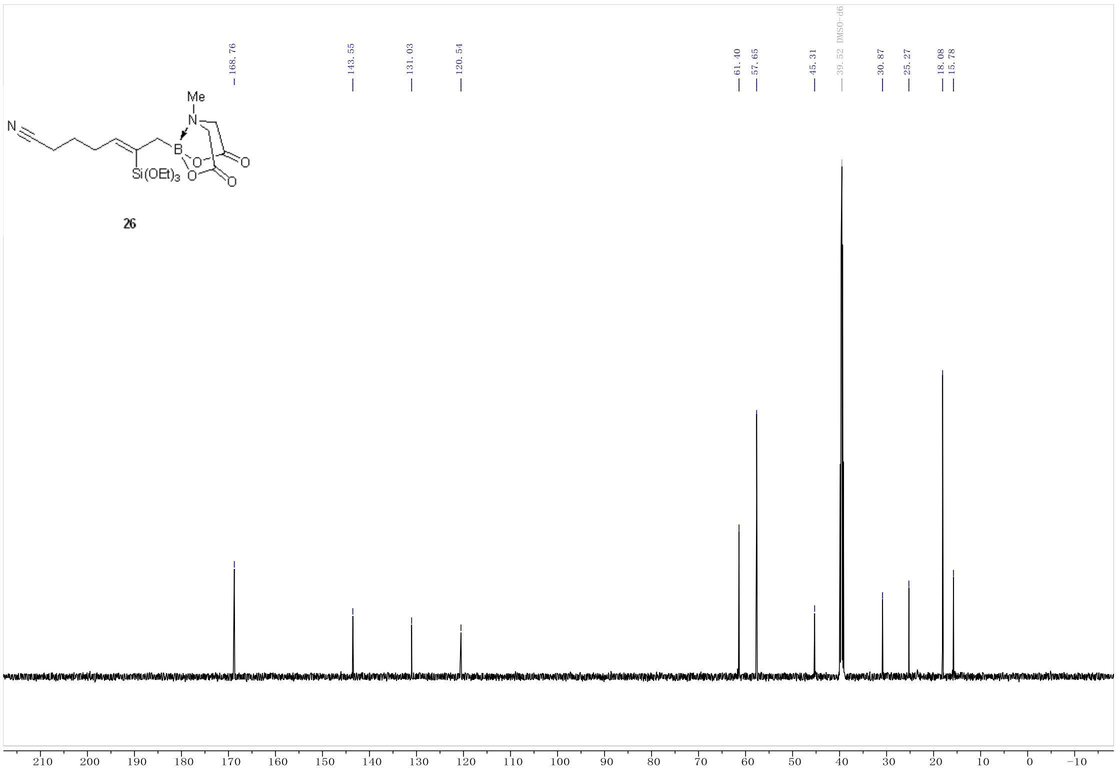

## NOE of 26

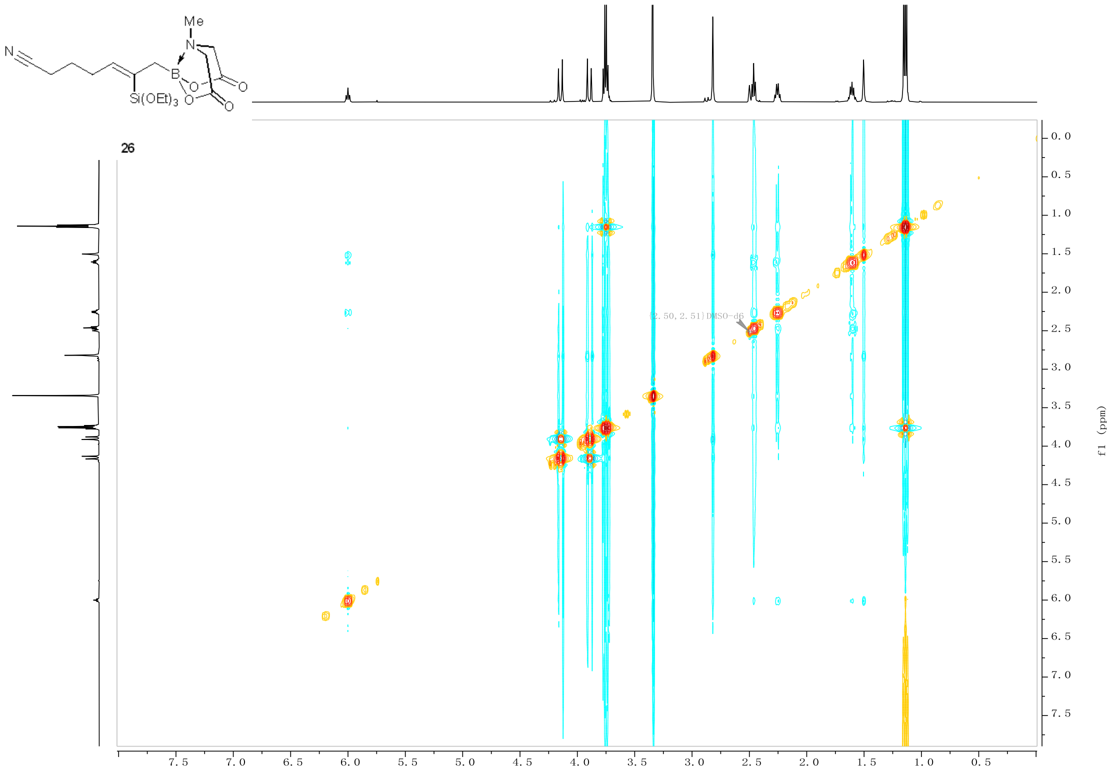

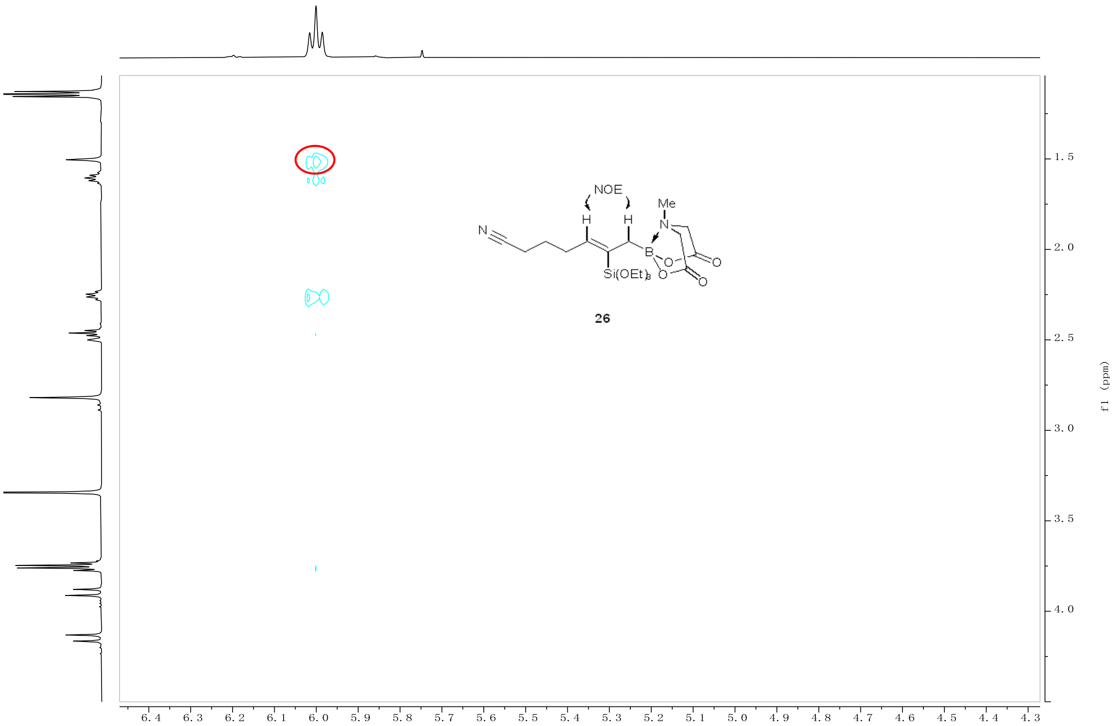

## $^1$H NMR of 27 (400 MHz, Acetone-$d_6$)

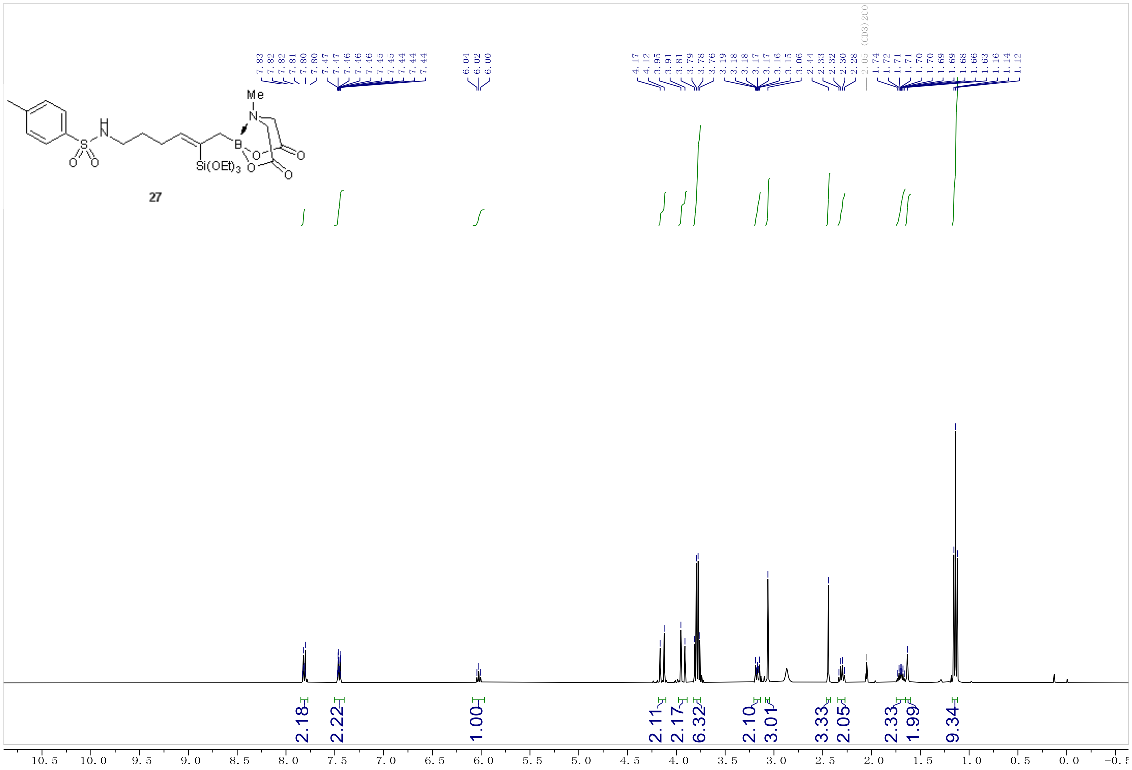

**$^{13}$C NMR of 27 (101 MHz, Acetone-$d_6$)**

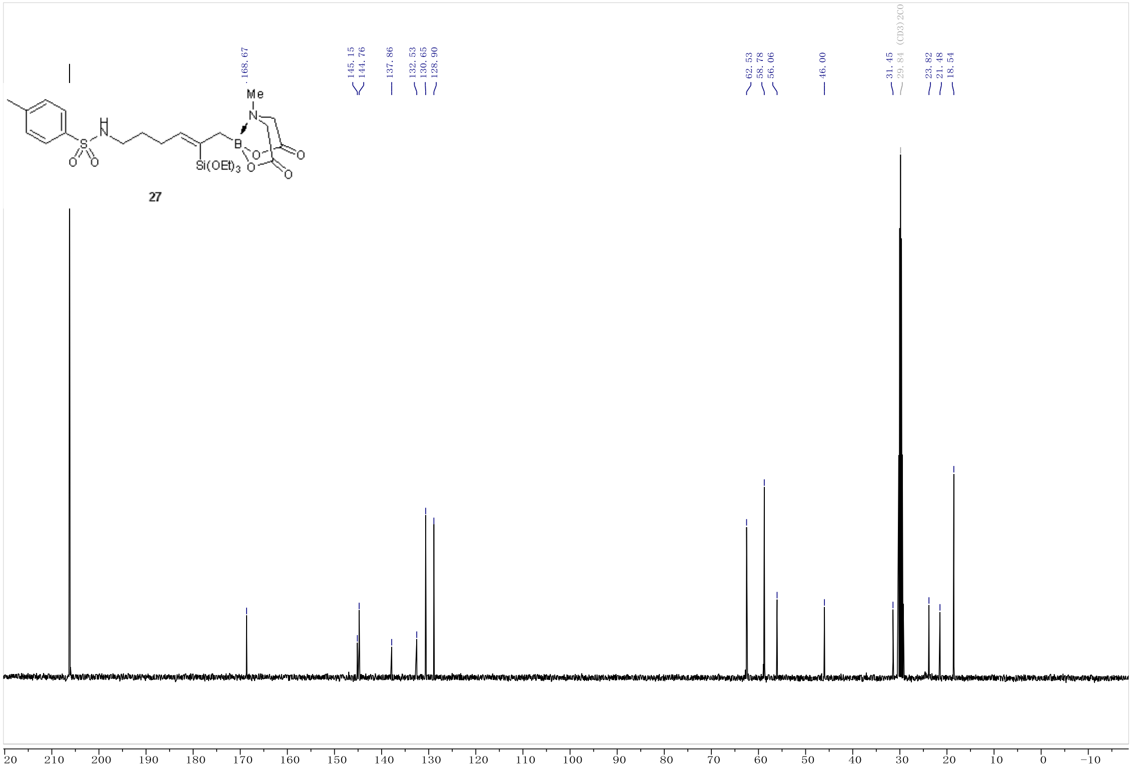

$^1$H NMR of 28 (400 MHz, Acetone-$d_6$)

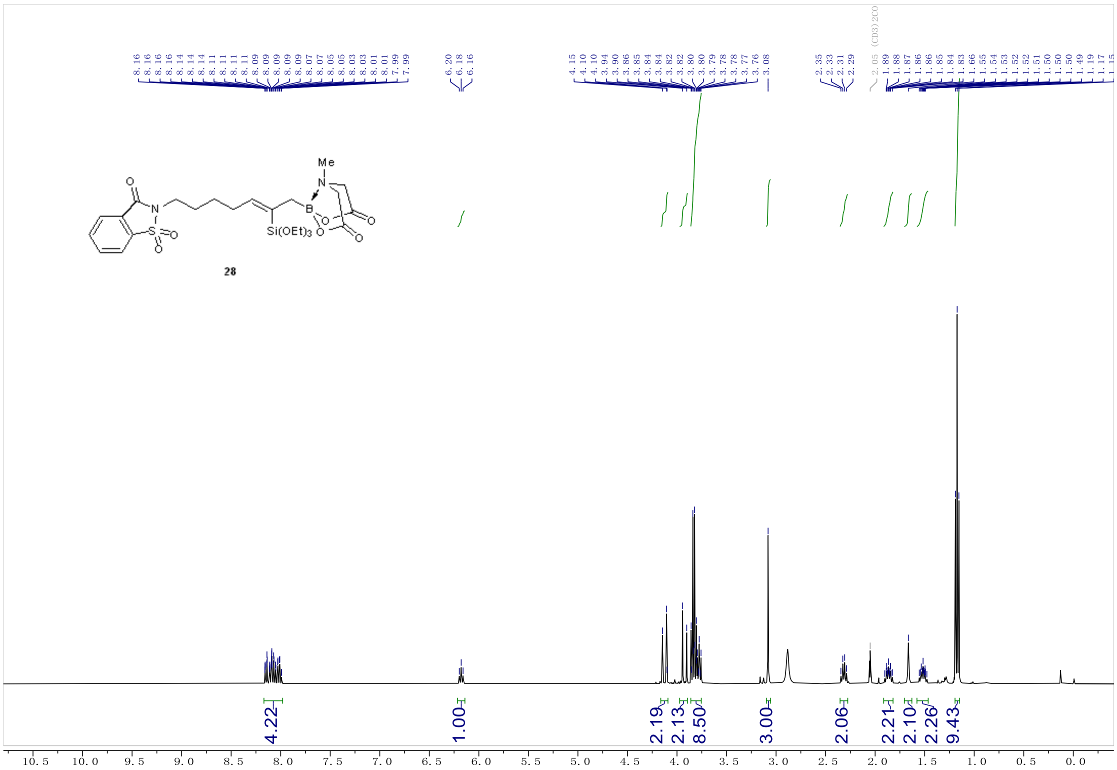

$^{13}$C NMR of 28 (101 MHz, Acetone-$d_6$)

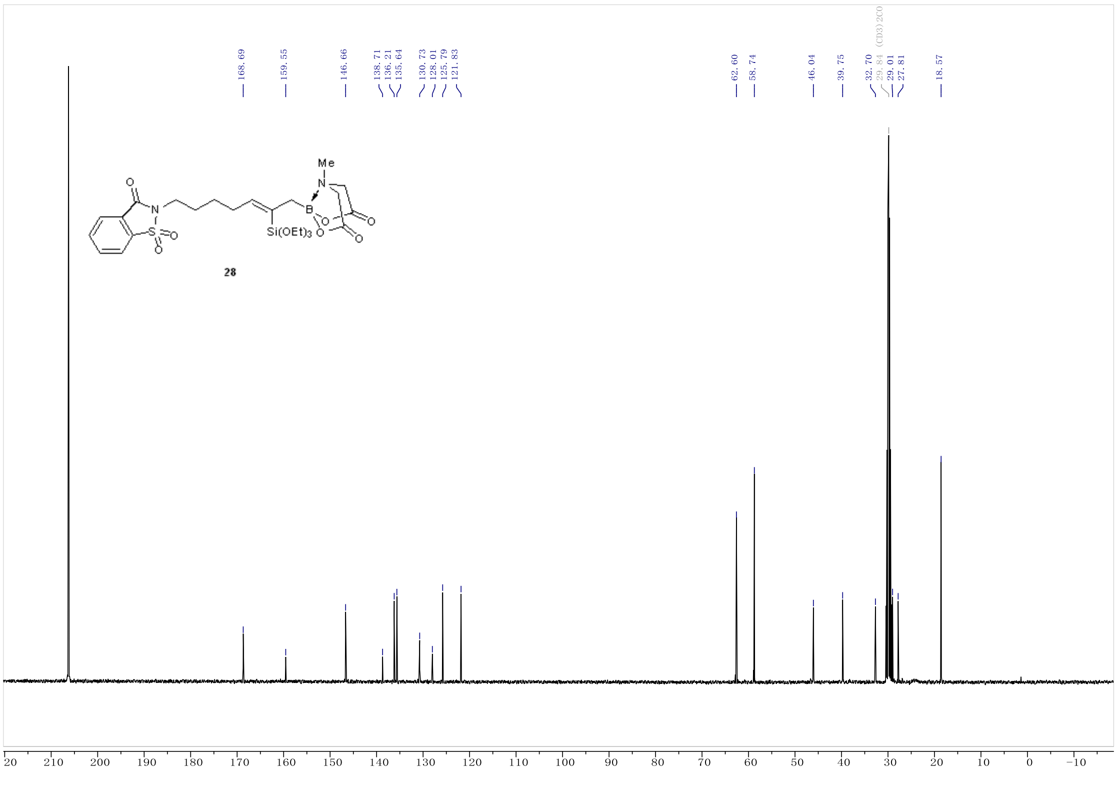

## $^{1}$H NMR of 29 (400 MHz, Acetone-$d_6$)

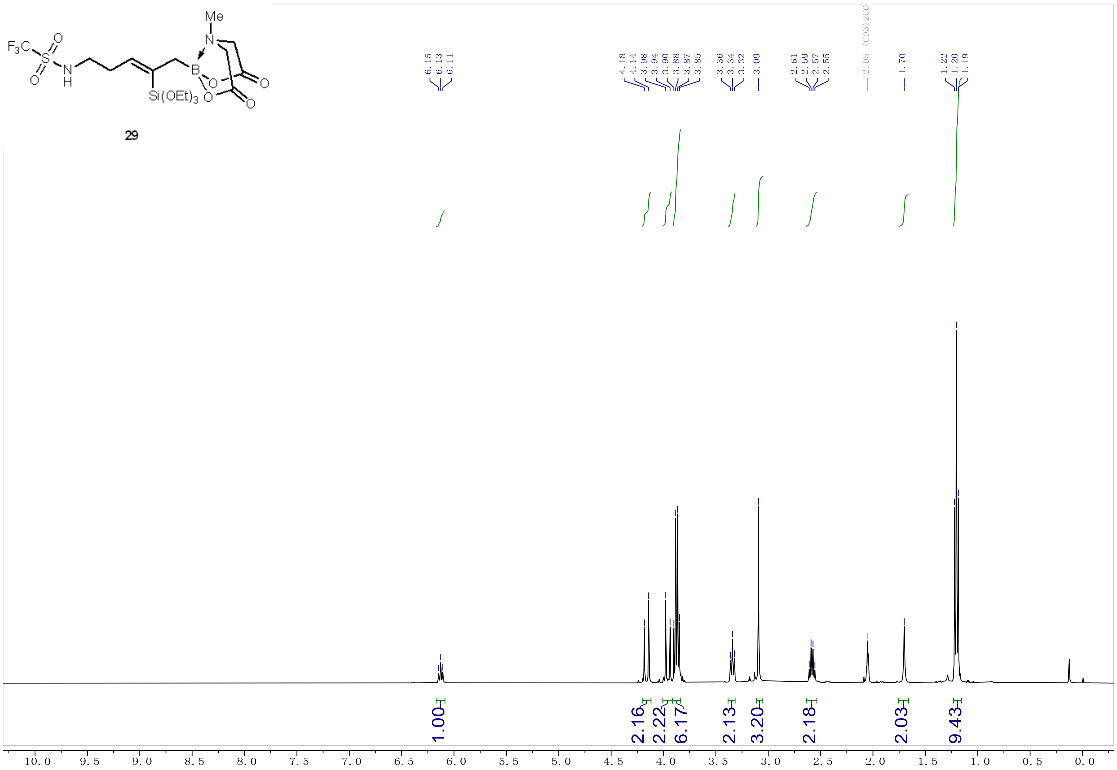


## $^{13}$C NMR of 29 (101 MHz, Acetone-$d_6$)

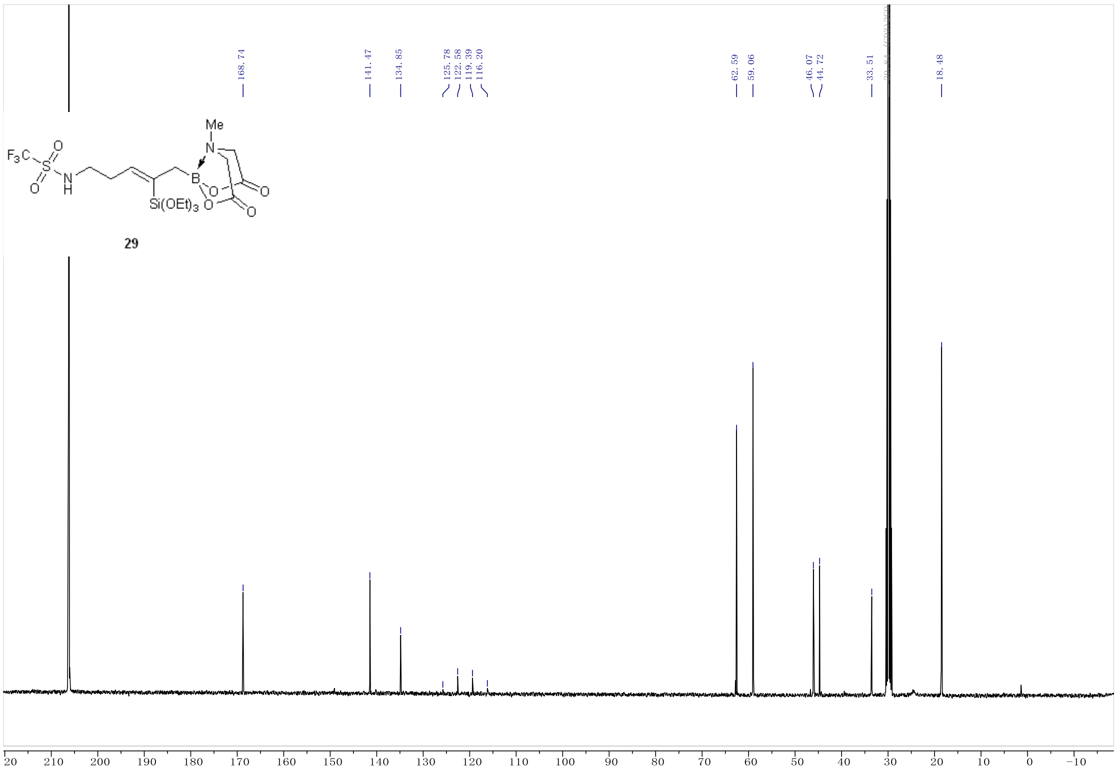

## $^{19}$F NMR of 29 (376 MHz, Acetone-$d_6$)

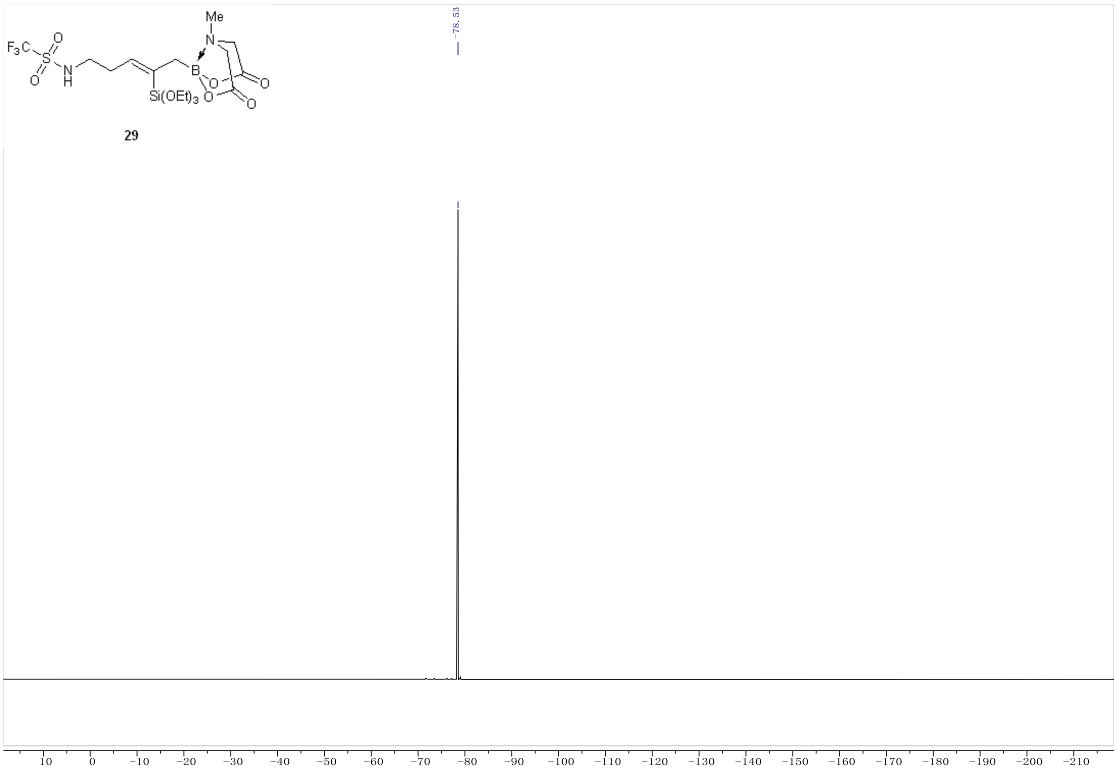


## $^1$H NMR of 30 (500 MHz, Acetone-$d_6$)

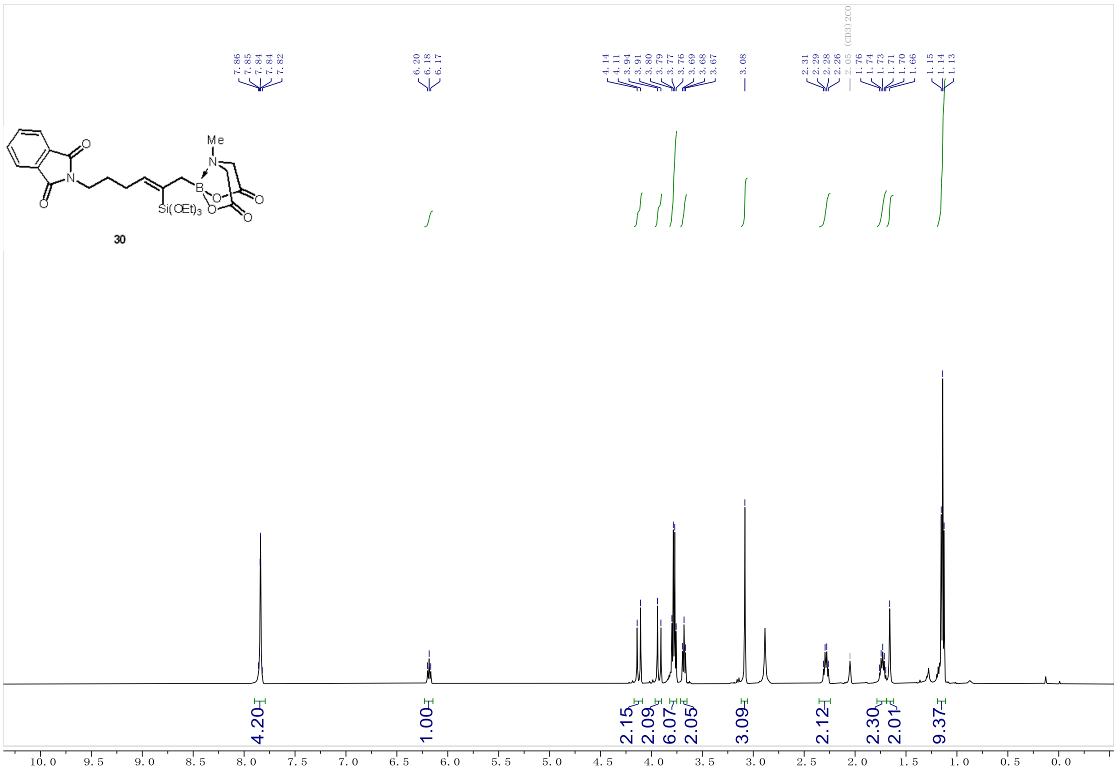

## $^{13}$C NMR of 30 (126 MHz, Acetone-$d_6$)

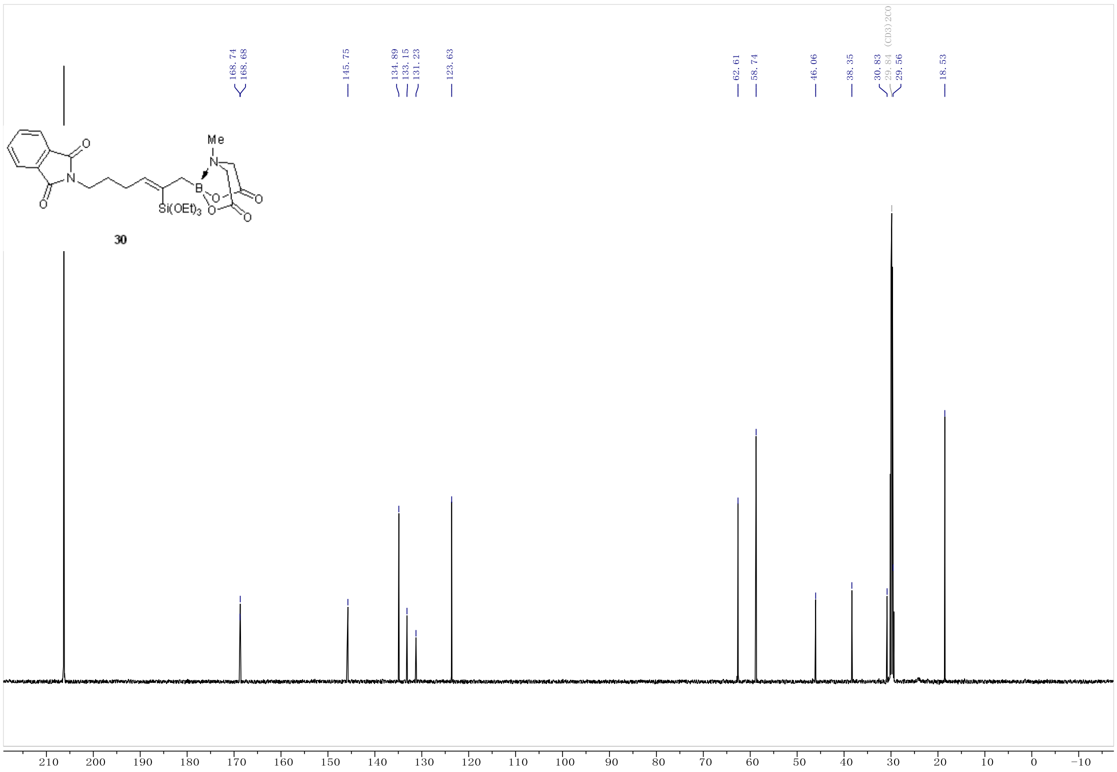


## $^{1}$H NMR of 31 (400 MHz, Acetone-$d_6$)

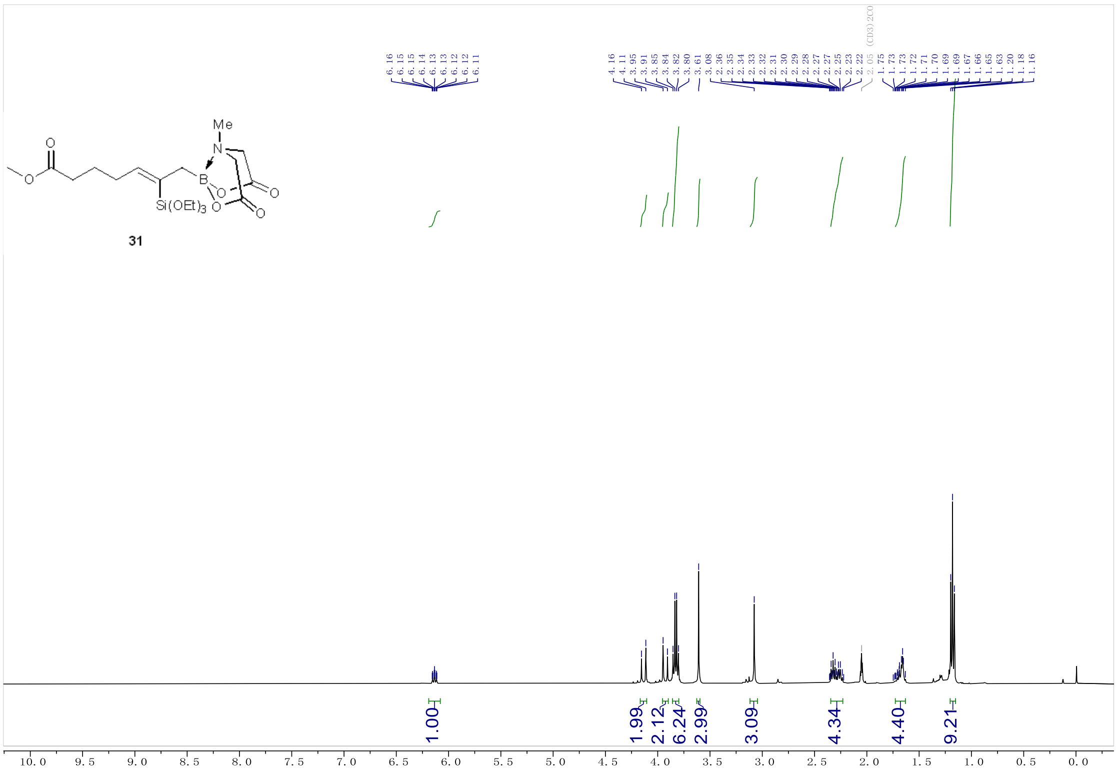

## $^{13}C$ NMR of 31 (101 MHz, Acetone-$d_6$)

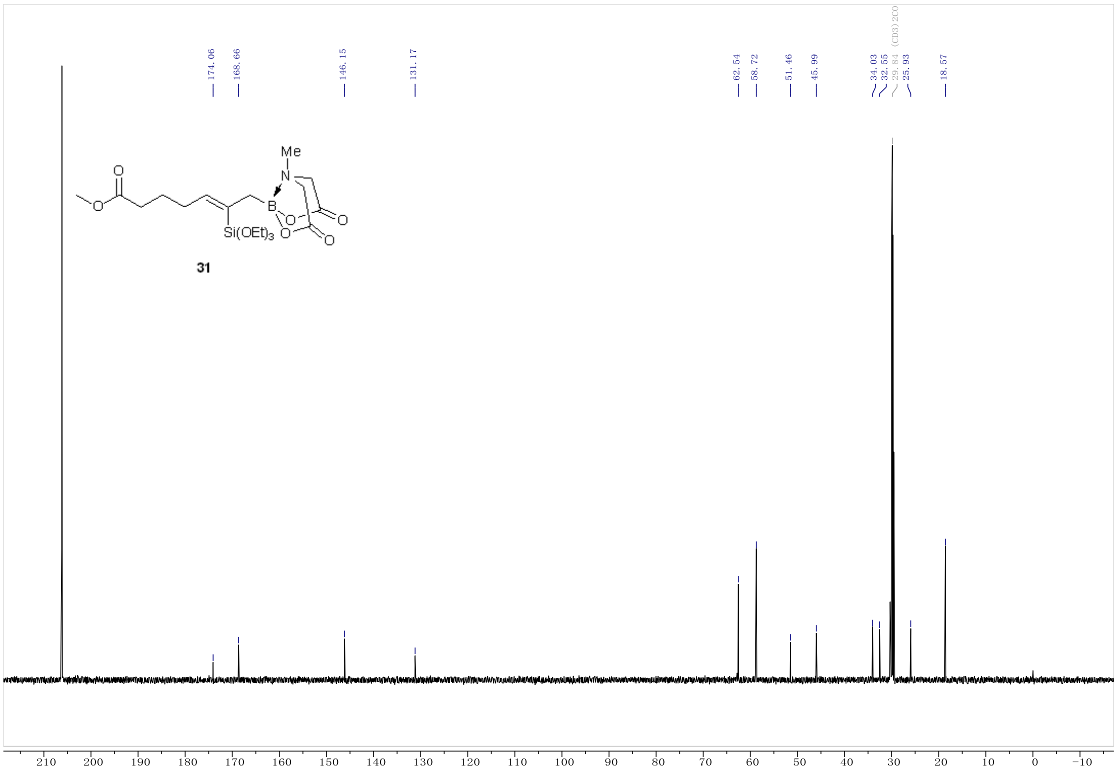

## $^{1}H$ NMR of 32 (400 MHz, $CD_3CN$)

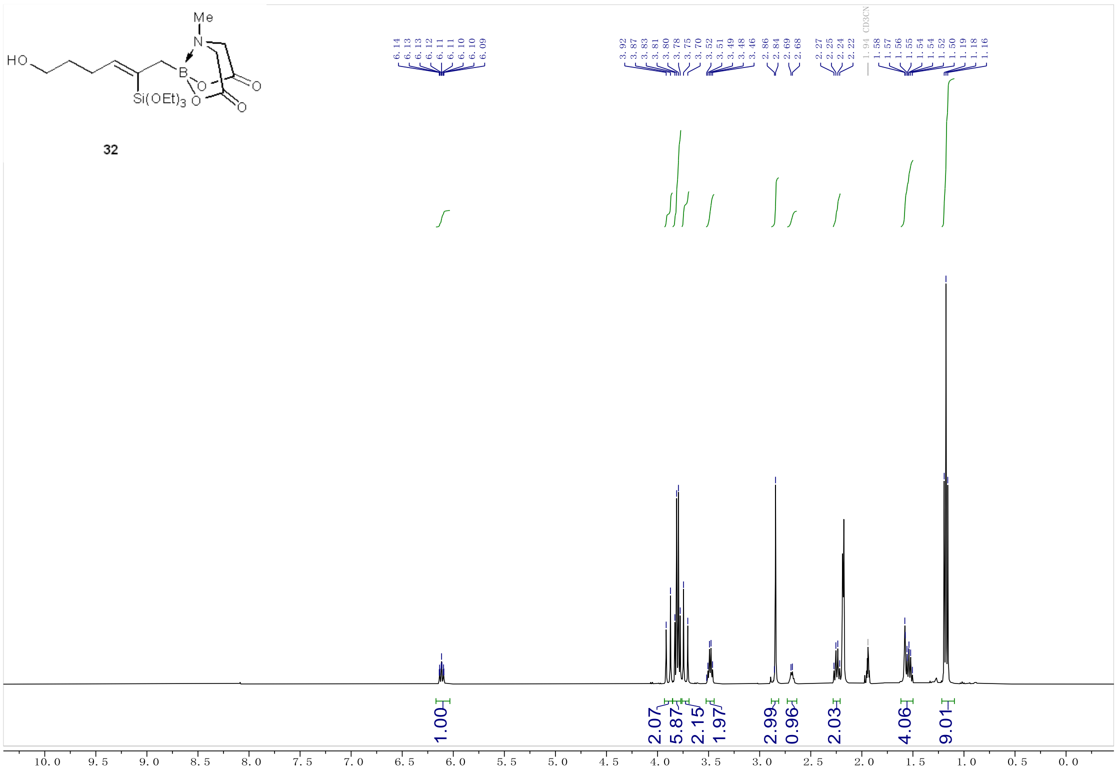

## $^{13}C$ NMR of 32 (101 MHz, $CD_3CN$)

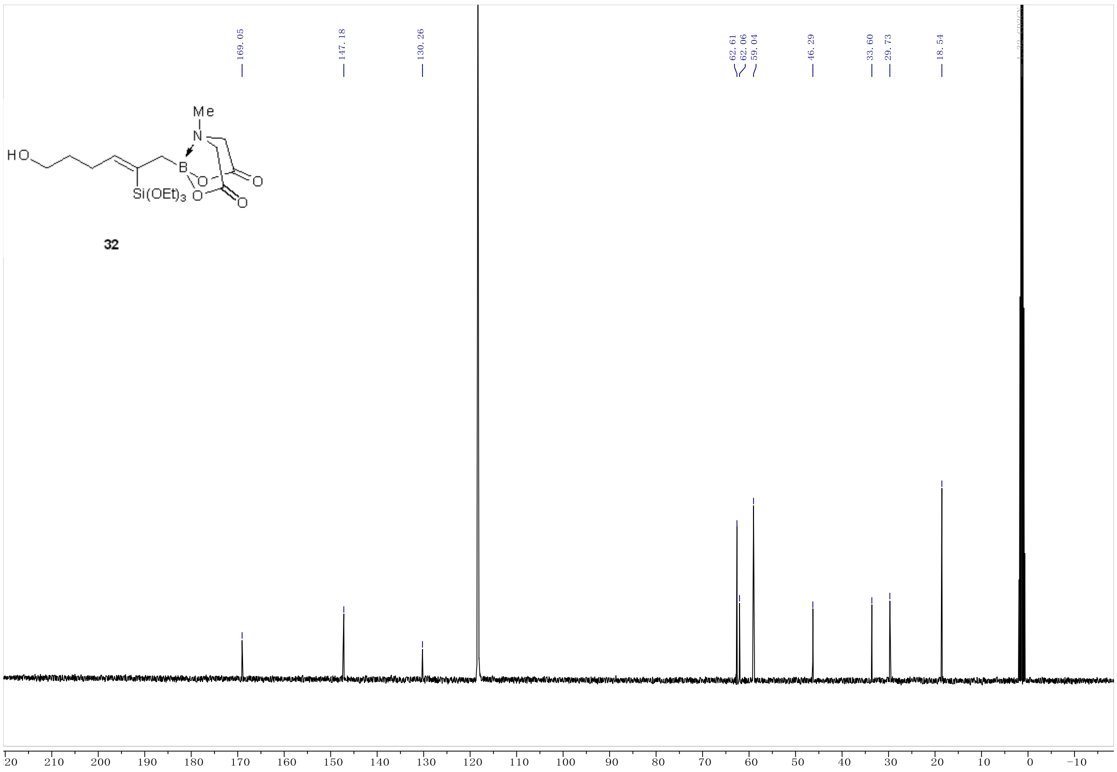

## $^{1}H$ NMR of 33 (400 MHz, $CD_3CN$)

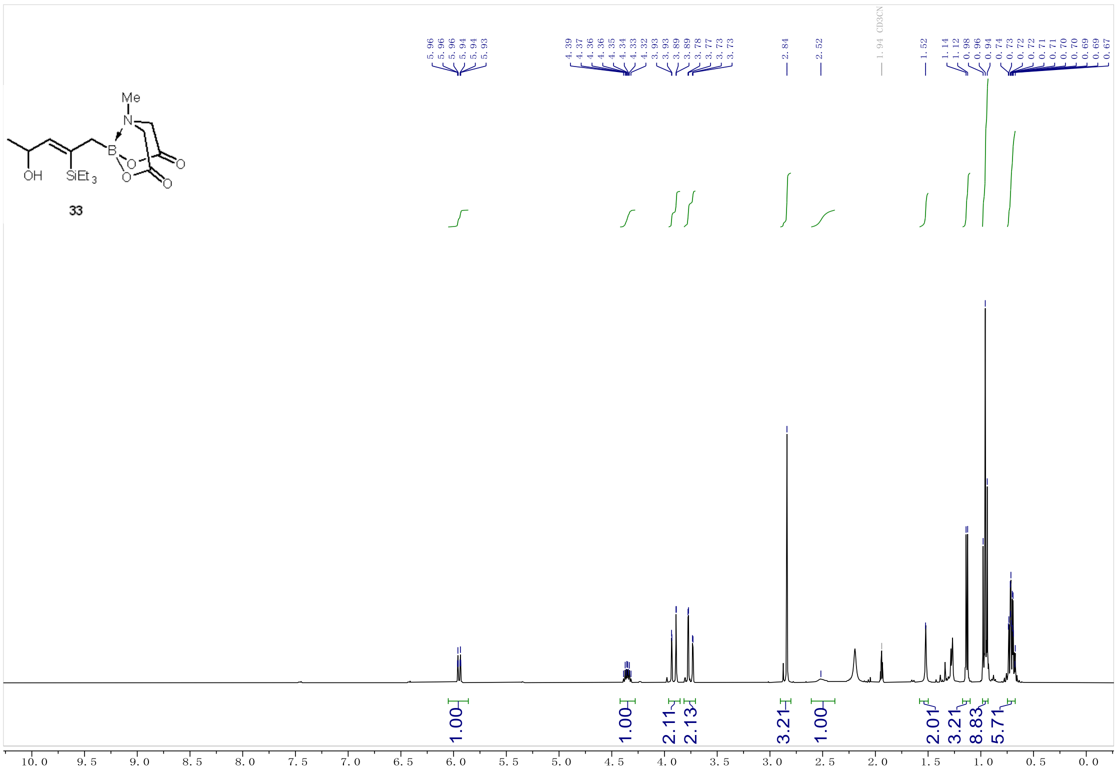

## $^{13}$C NMR of 33(101 MHz, $CD_3CN$)

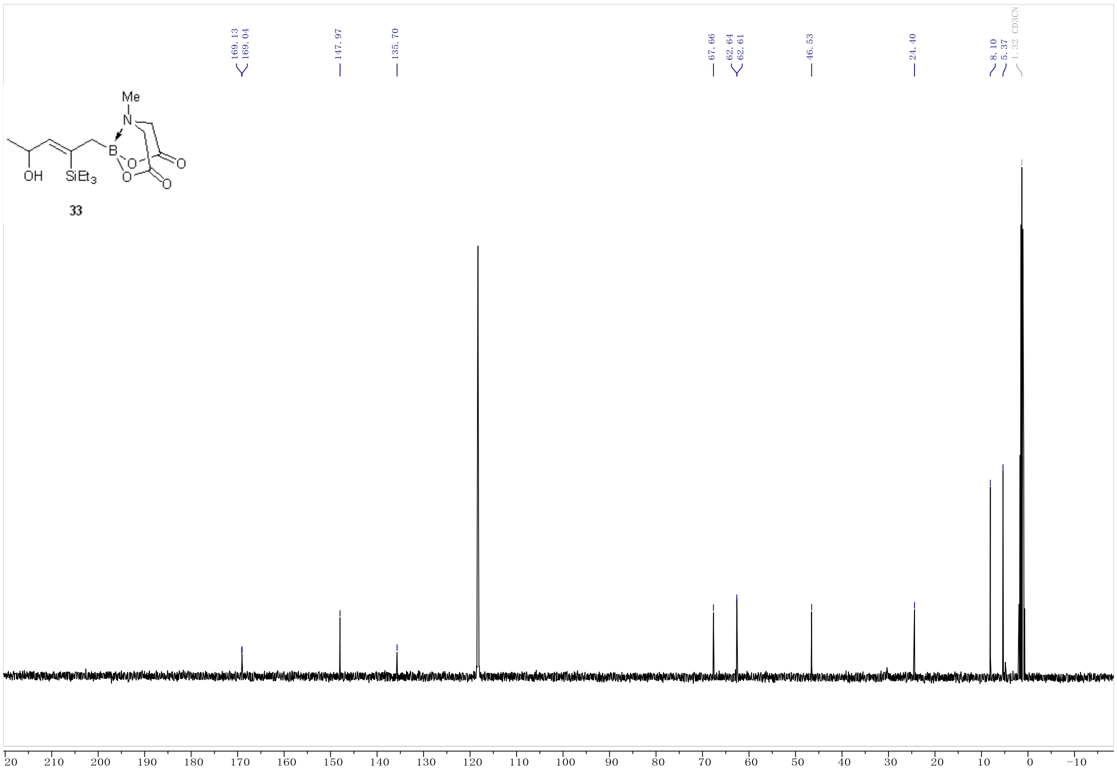


## $^{1}$H NMR of 34 (400 MHz, Acetone-$d_6$)

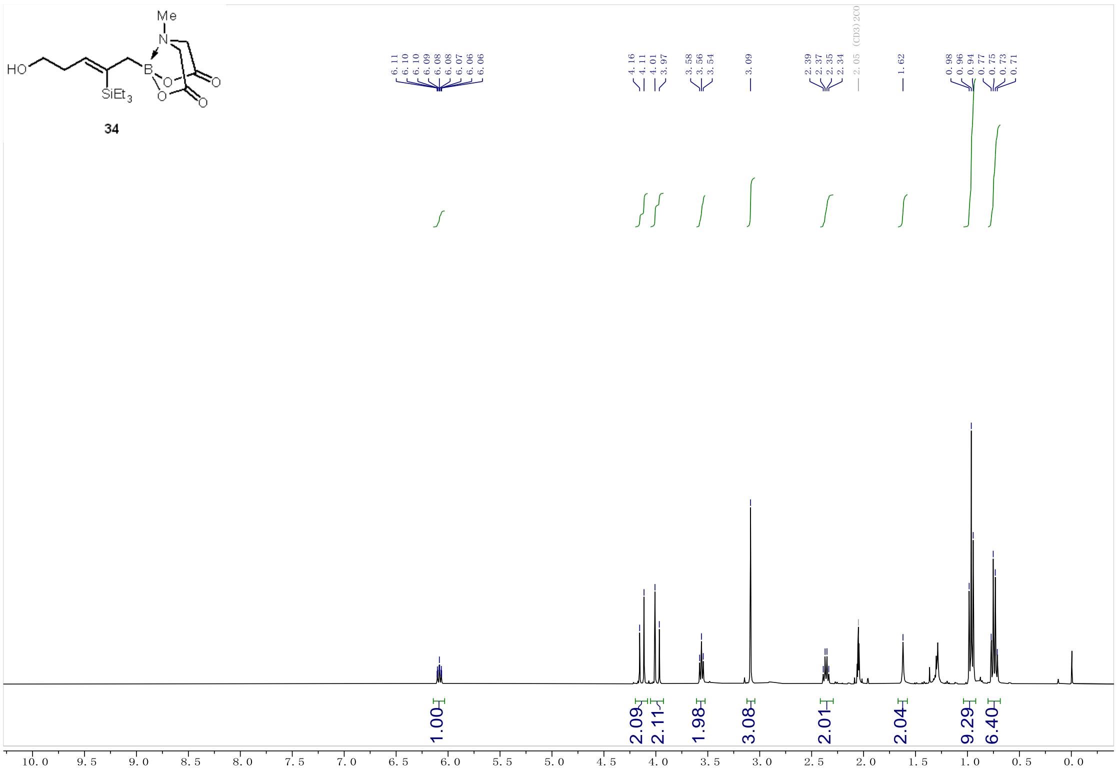

$^{13}C$ NMR of 34 (101 MHz, Acetone-$d_6$)

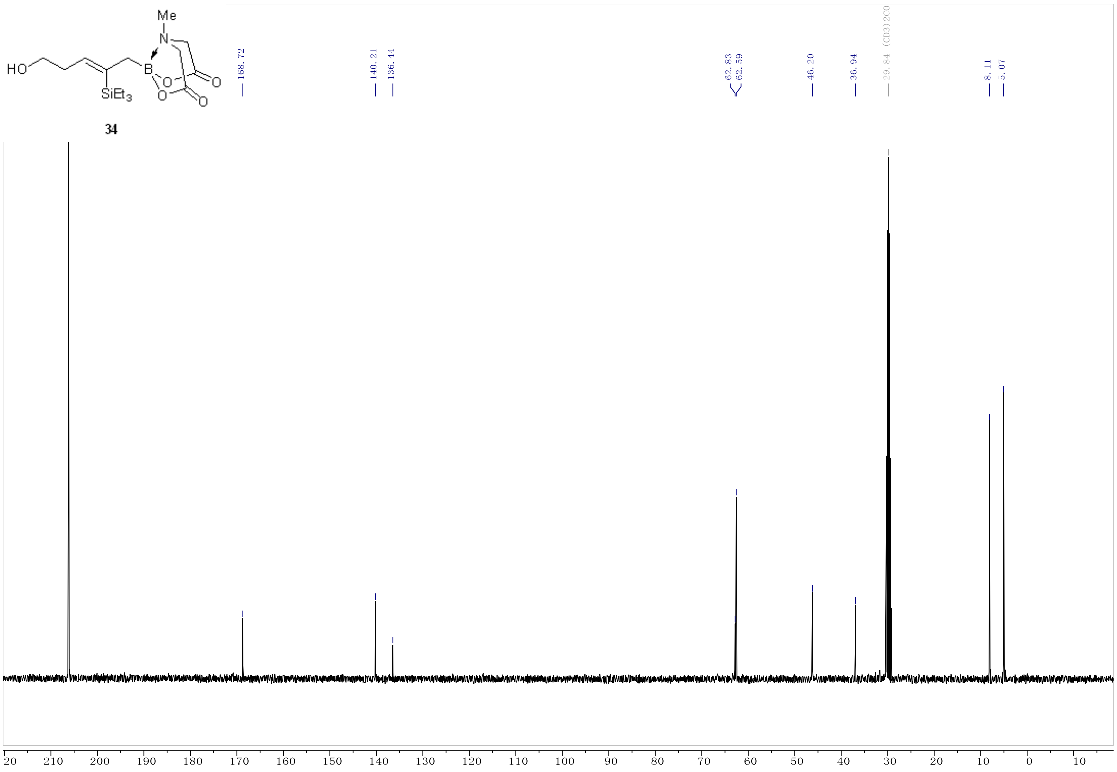

$^{1}H$ NMR of 35 (400 MHz, DMSO-$d_6$)

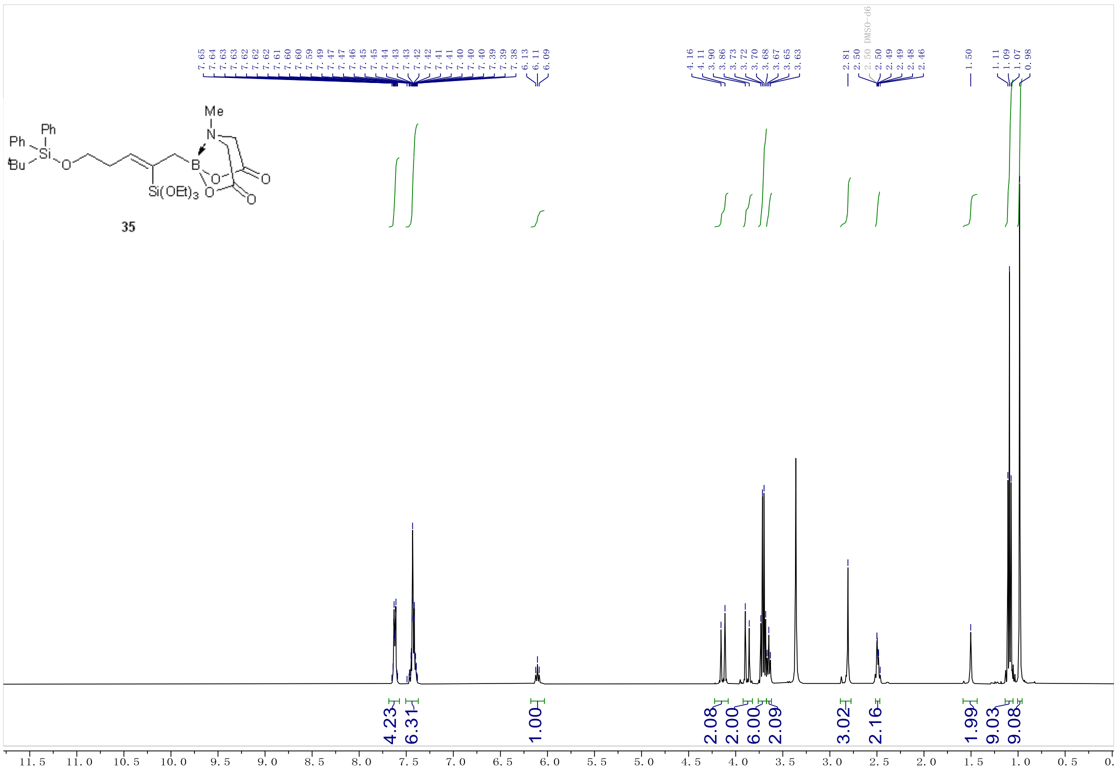

## $^{13}C$ NMR of 35 (101 MHz, DMSO-$d_6$)

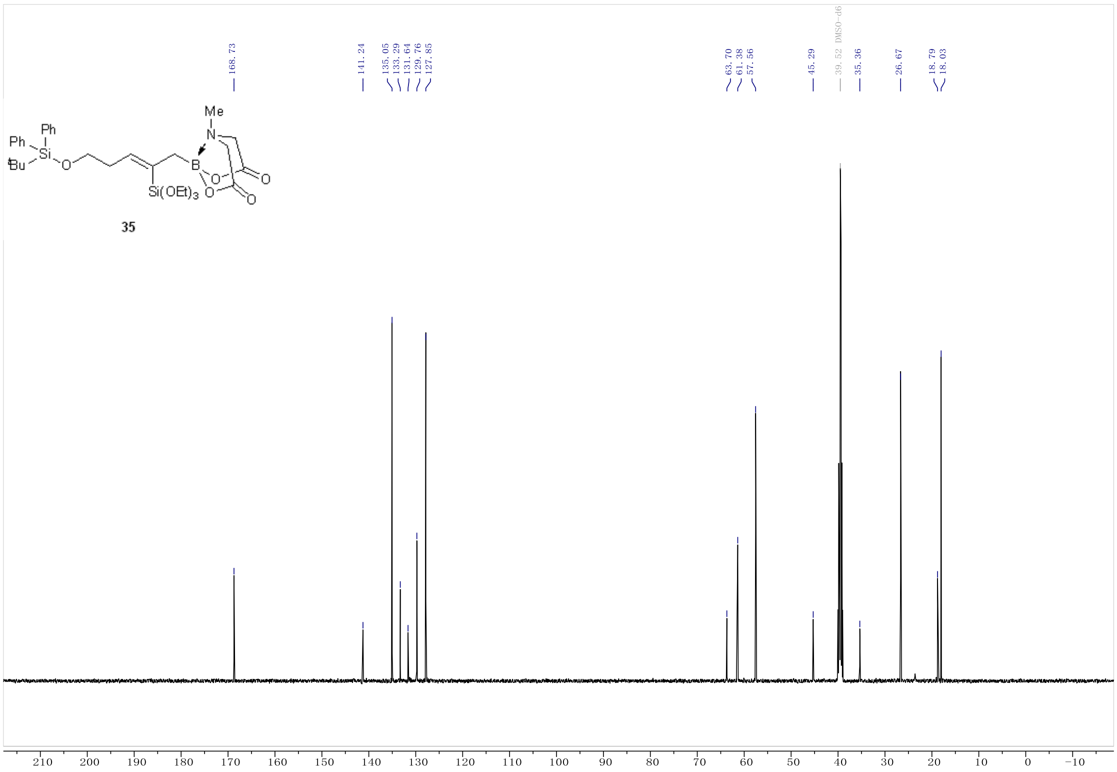

## $^{1}H$ NMR of 36 (400 MHz, $CD_3CN$)

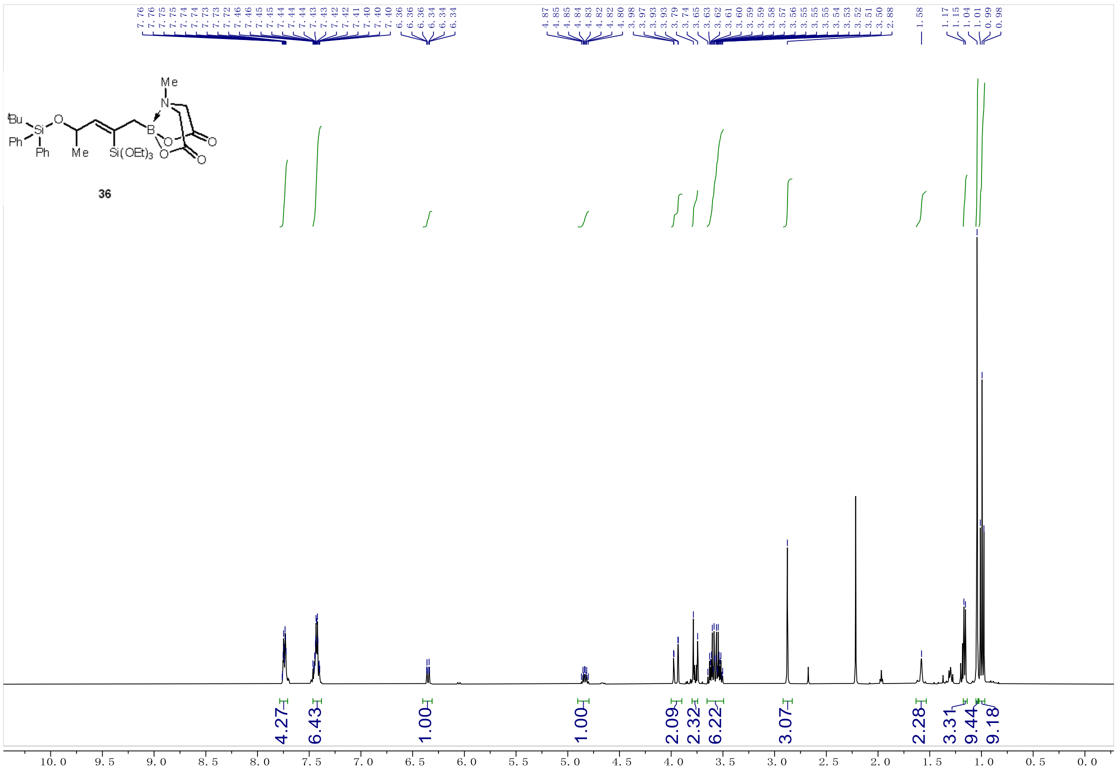

## $^{13}$C NMR of 36 (101 MHz, $CD_3CN$)

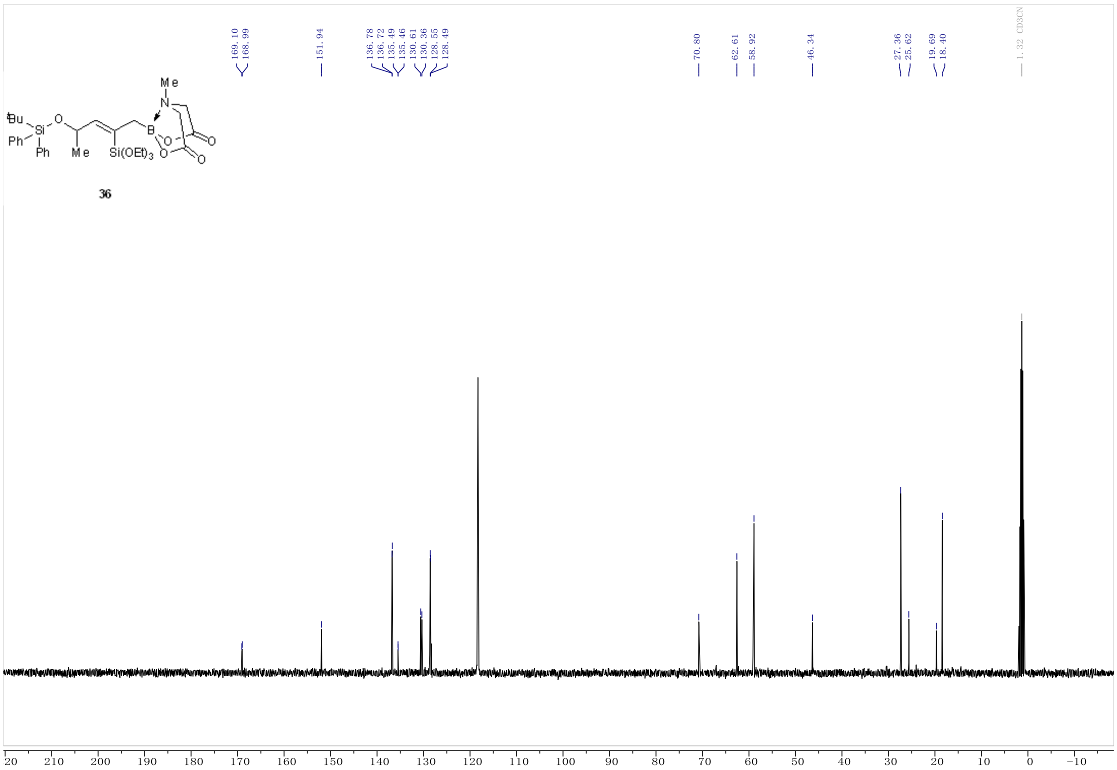

## $^{1}$H NMR of 37 (400 MHz, DMSO-$d_6$)

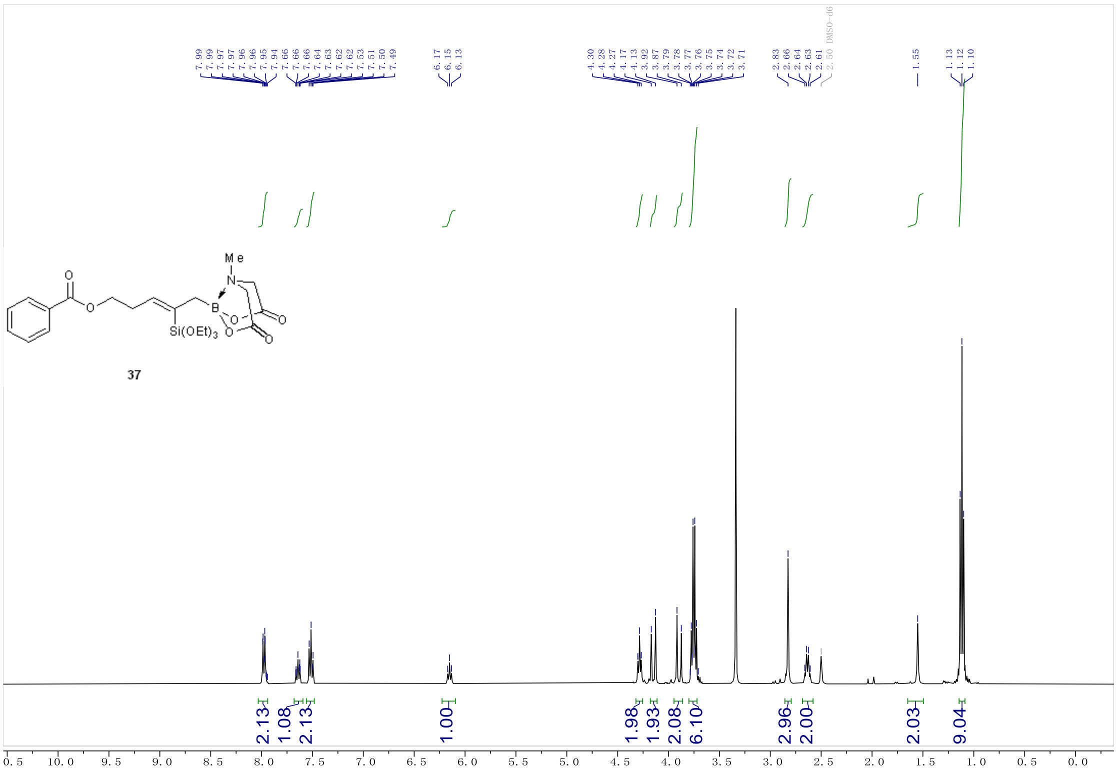

## $^{13}$C NMR of 37 (126 MHz, DMSO-$d_6$)

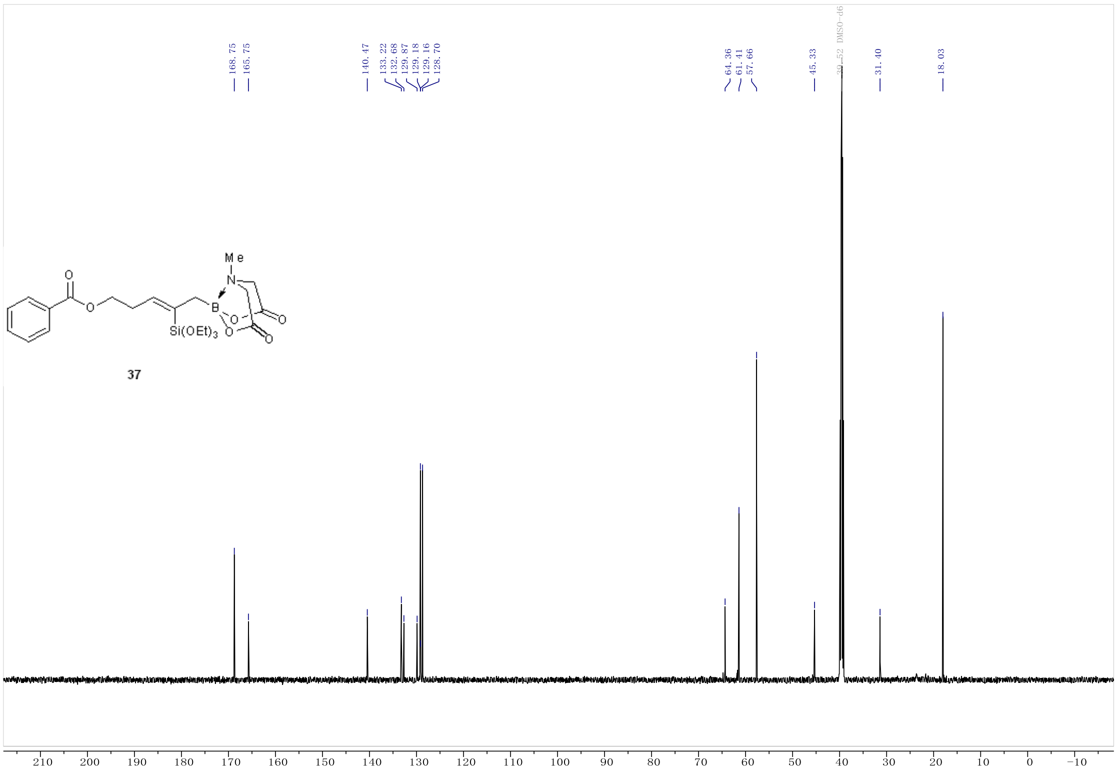

## NOE of 37

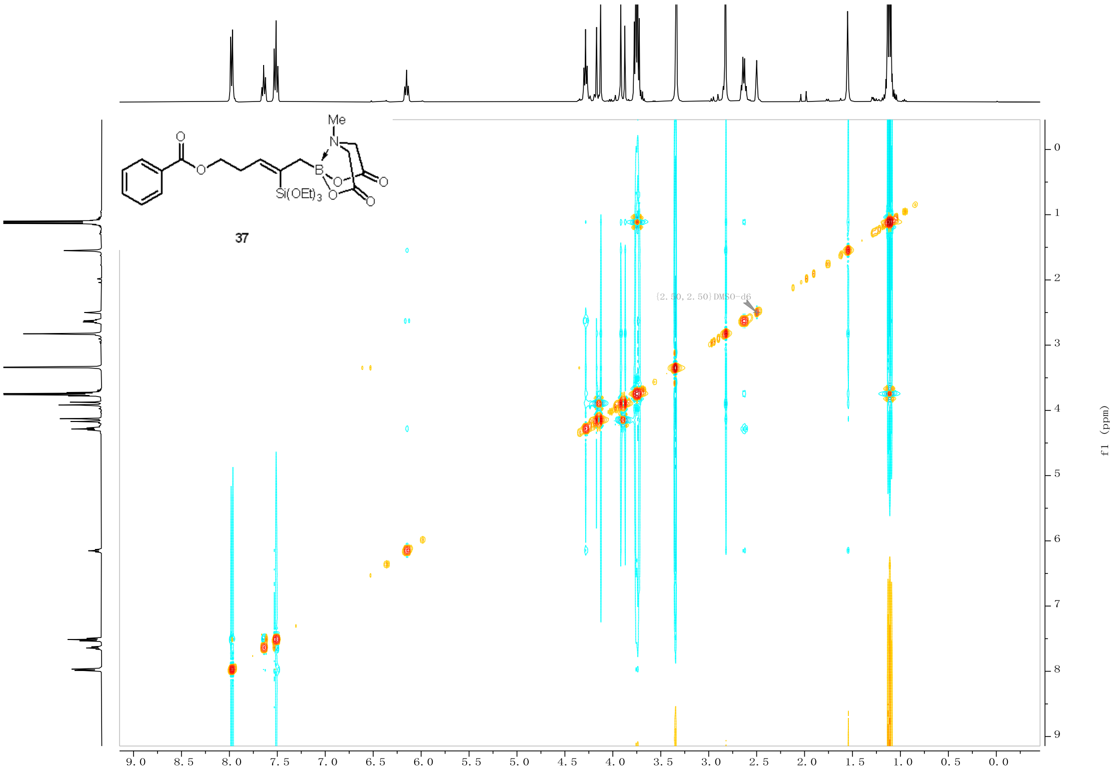

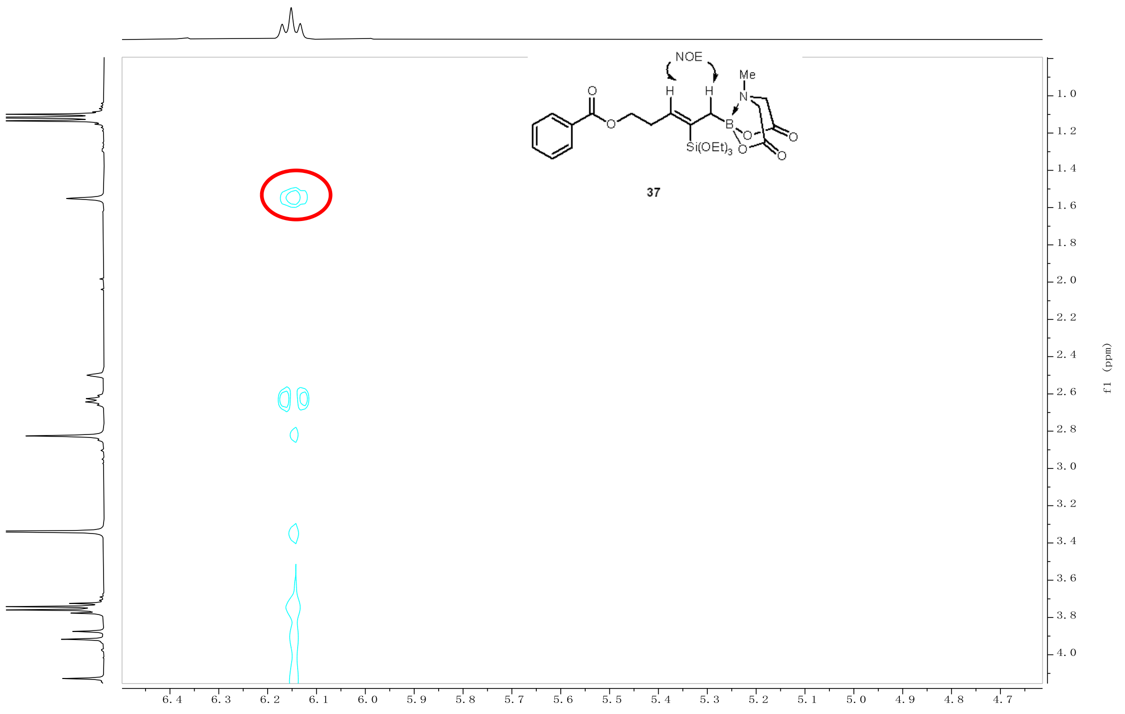


### $^{1}$H NMR of 38 (500 MHz, Acetone-$d_6$)

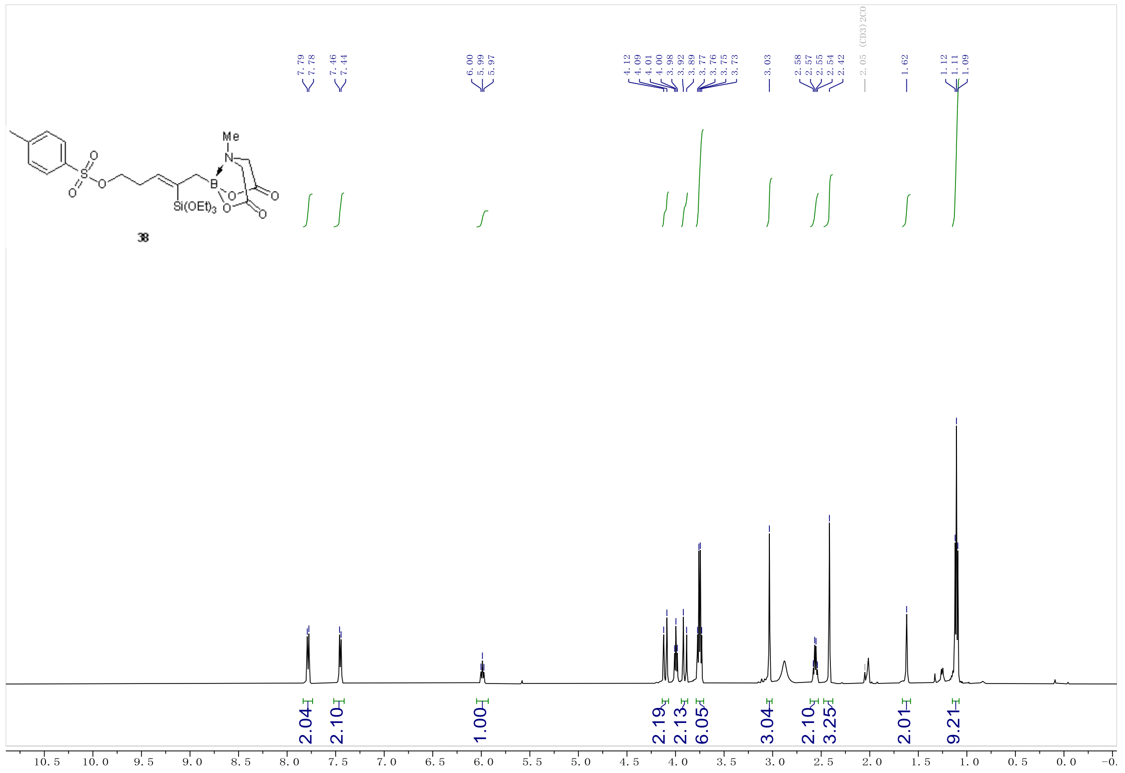

## $^{13}$C NMR of 38 (126 MHz, Acetone-$d_6$)

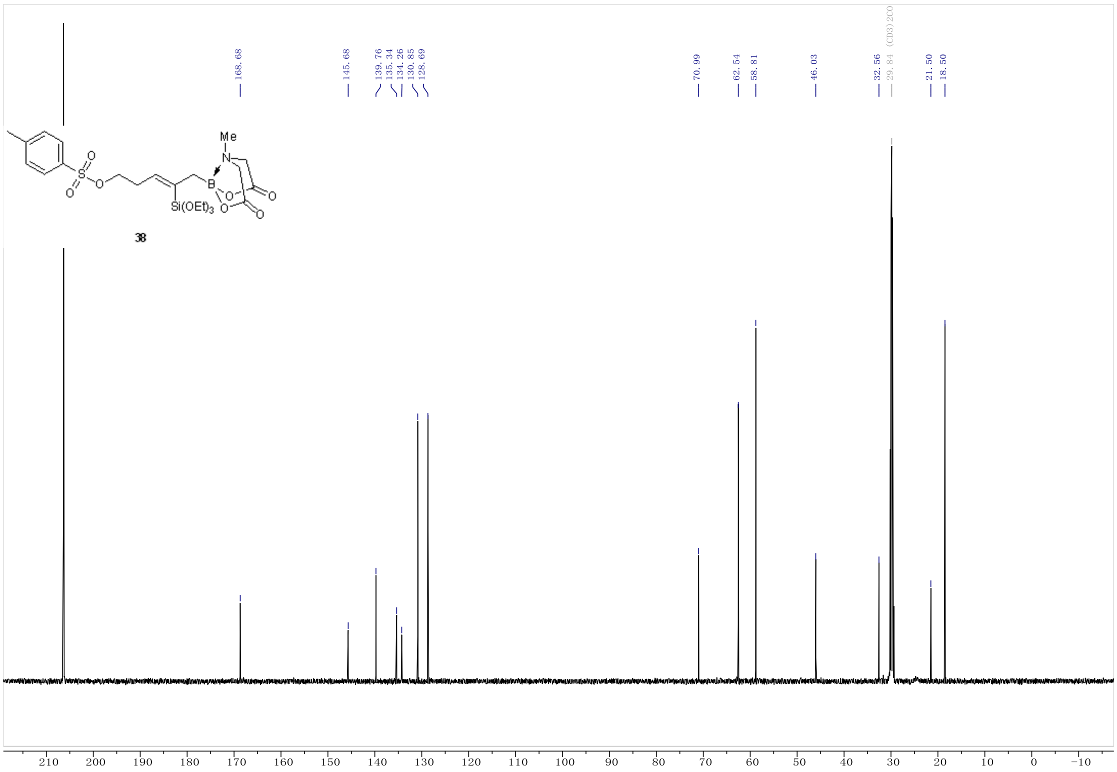


## $^{1}$H NMR of 39 (400 MHz, $CD_3CN$)

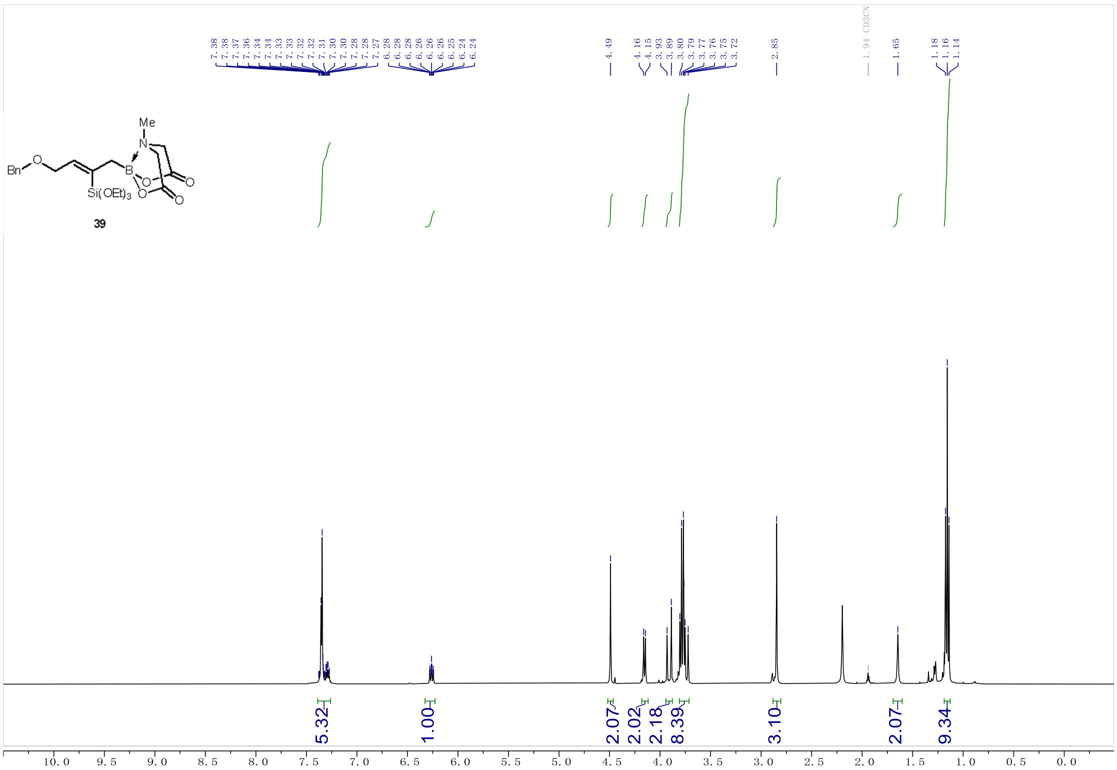

**$^{13}$C NMR of 39 (101 MHz, $CD_3CN$)**

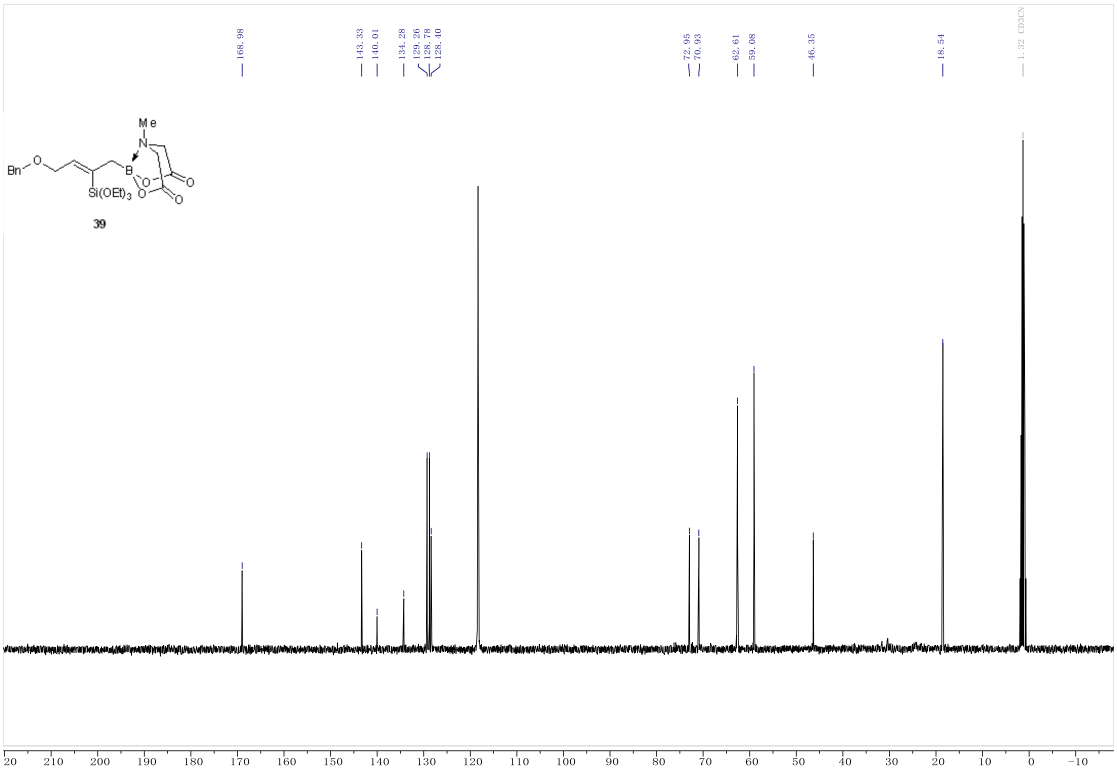

**$^{1}$H NMR of 40 (mixture of *Z* & *E* isomers) (400 MHz, DMSO-$d_6$)**

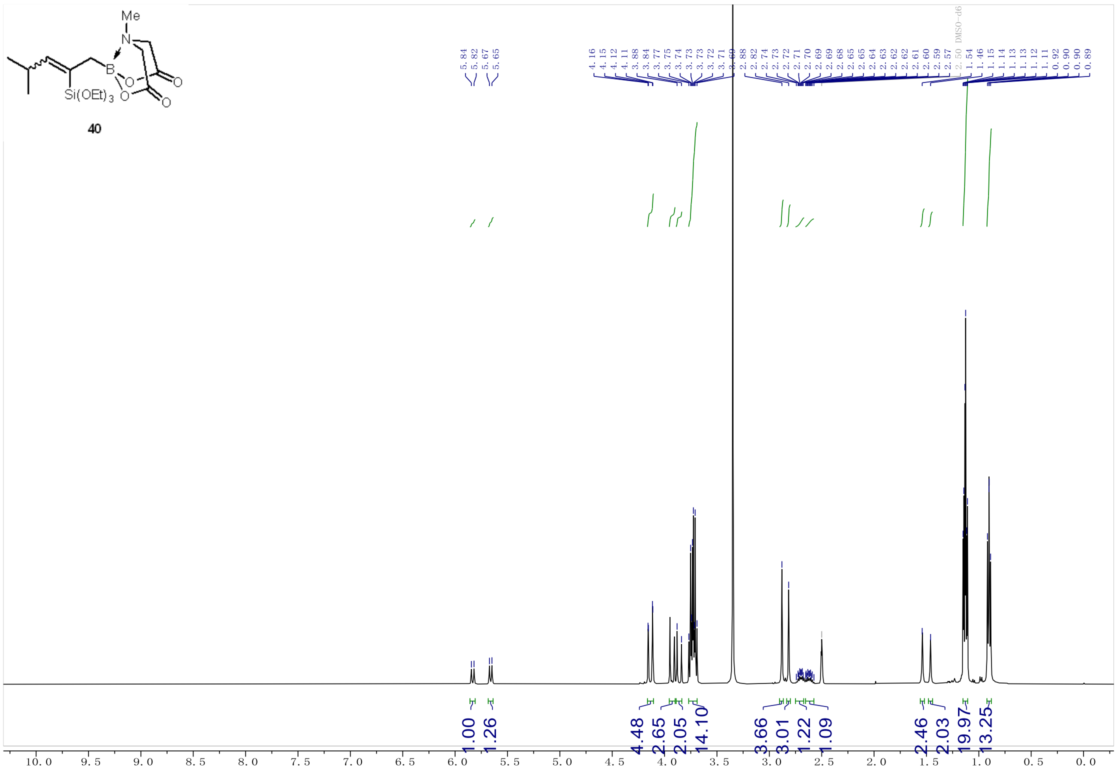

**$^{1}$H NMR of 41 (400 MHz, Acetone-$d_6$)**

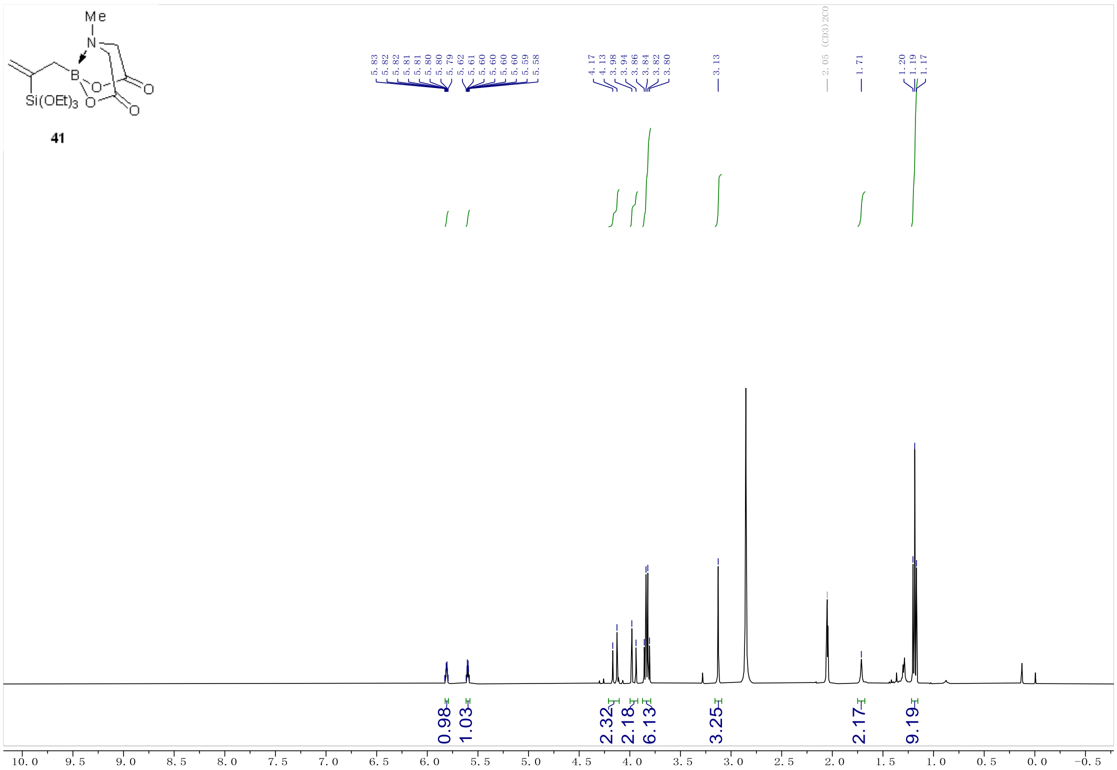


**$^{13}$C NMR of 41 (101 MHz, Acetone-$d_6$)**

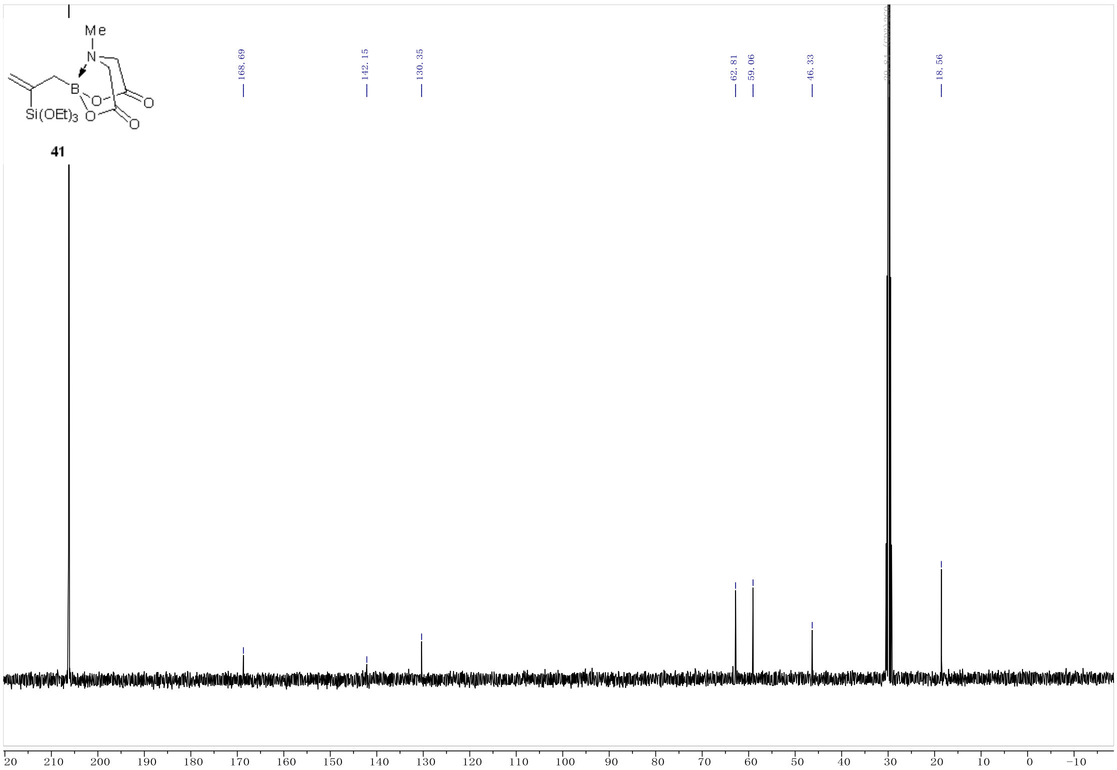

## $^{1}H$ NMR of 42 (400 MHz, $CD_3CN$)

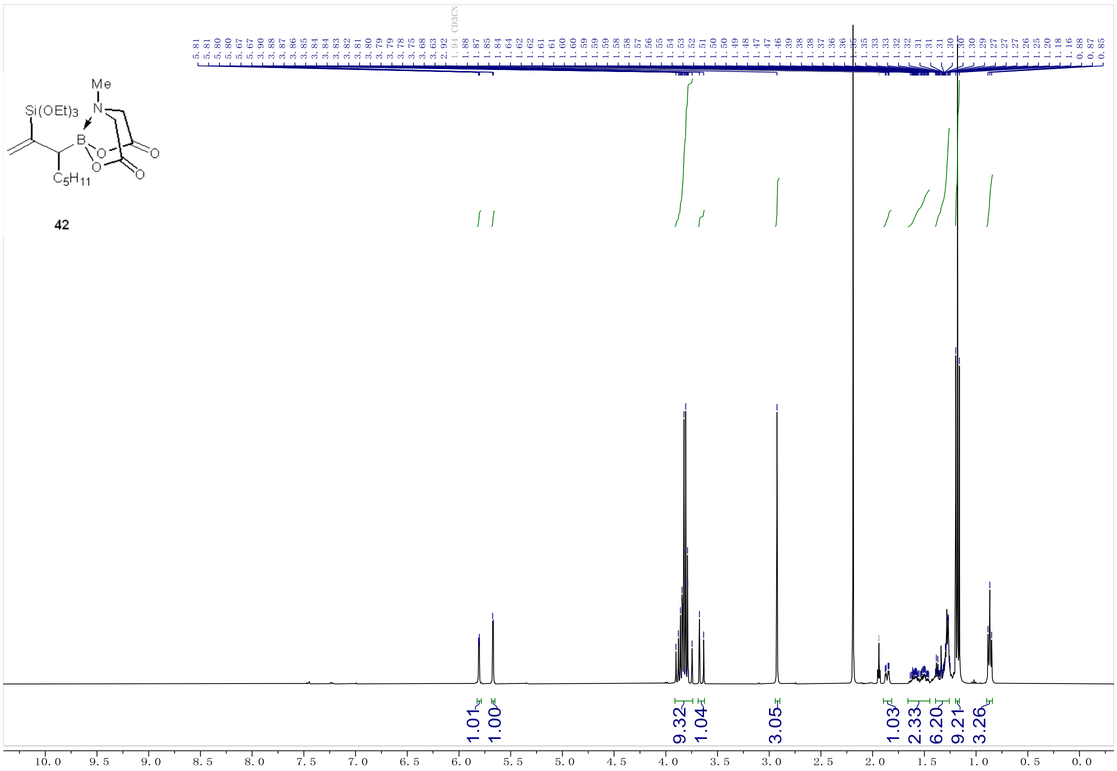


## $^{13}C$ NMR of 42 (101 MHz, $CD_3CN$)

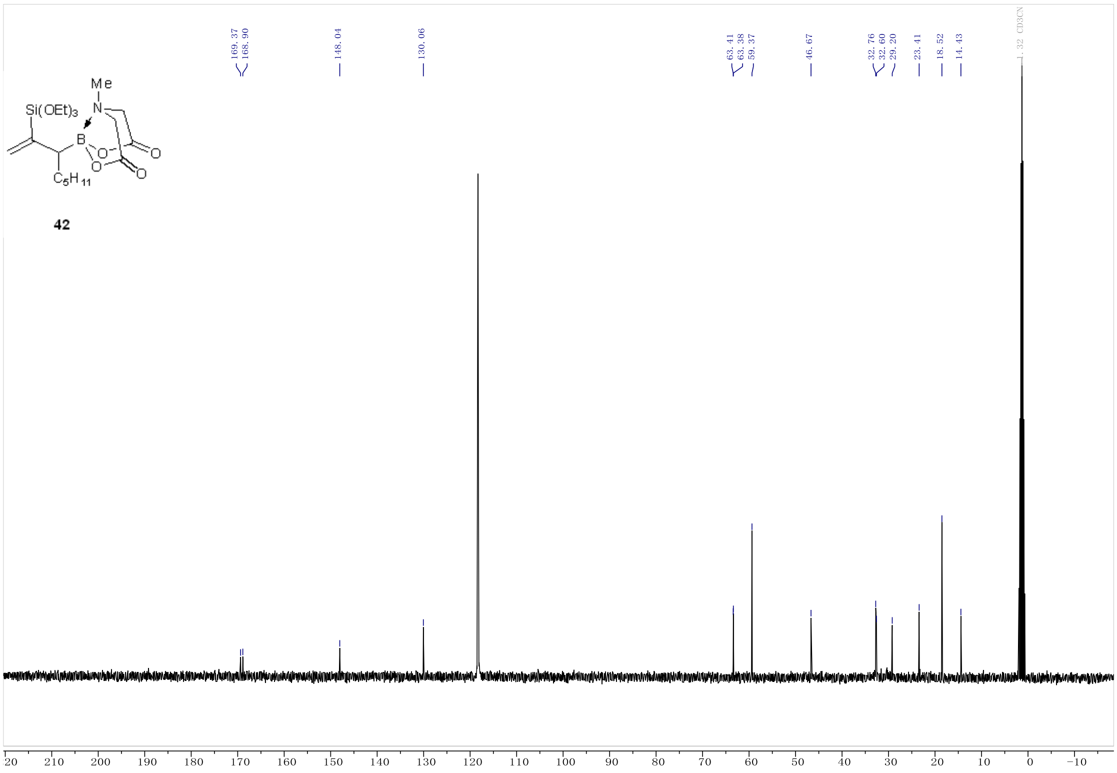

**$^{1}$H NMR of 43 (mixture of *Z* and *E* isomers) (400 MHz, Acetone-$d_6$)**

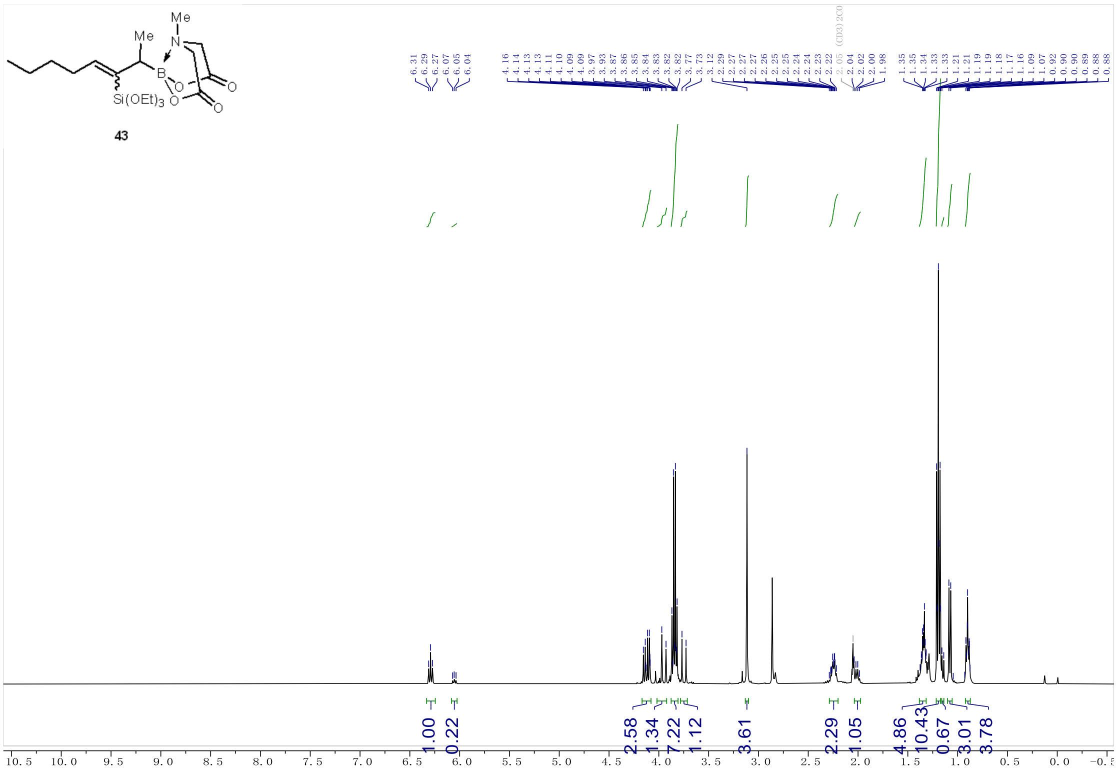

**$^{13}$C NMR of 43 (mixture of *Z* and *E* isomers) (101 MHz, Acetone-$d_6$)**

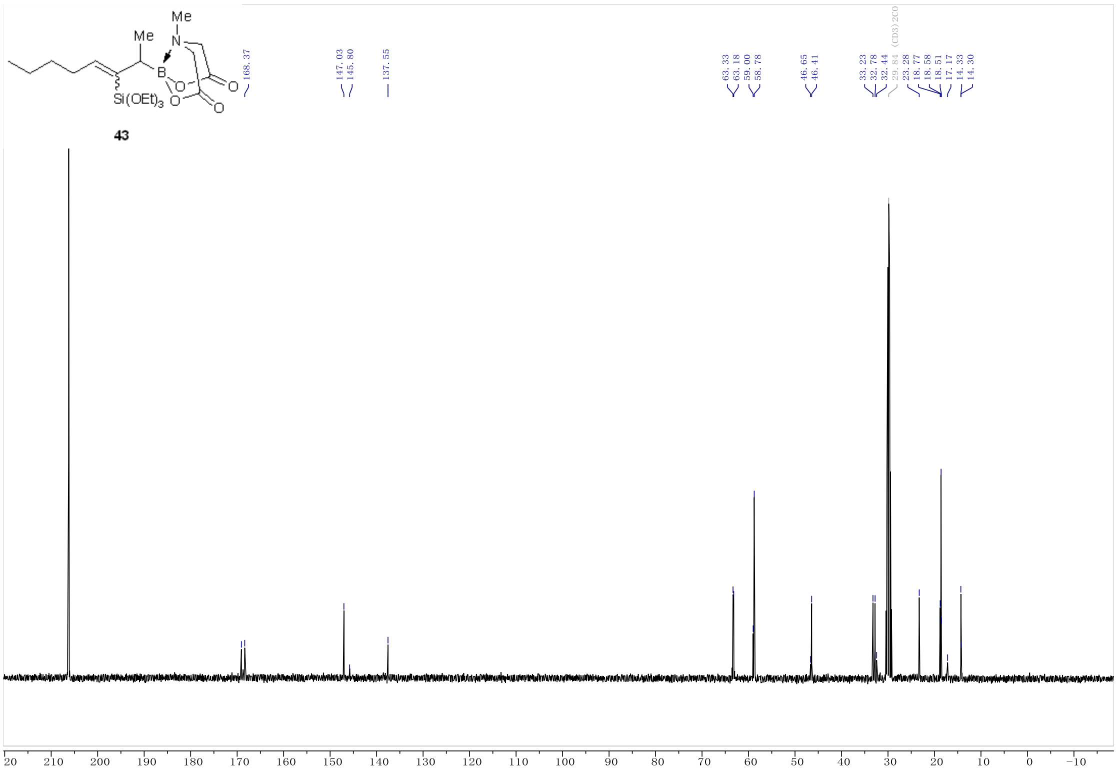

**$^{1}$H NMR of 44 (500 MHz, Acetone-$d_6$)**

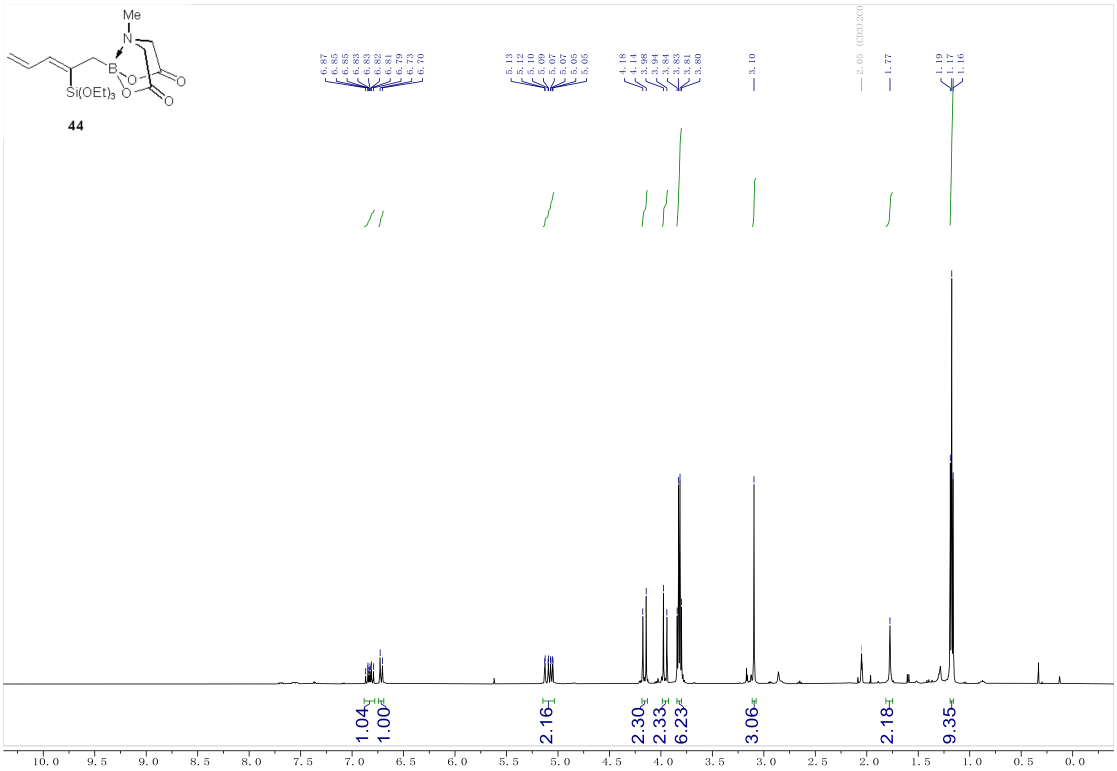

**$^{13}$C NMR of 44 (126 MHz, Acetone-$d_6$)**

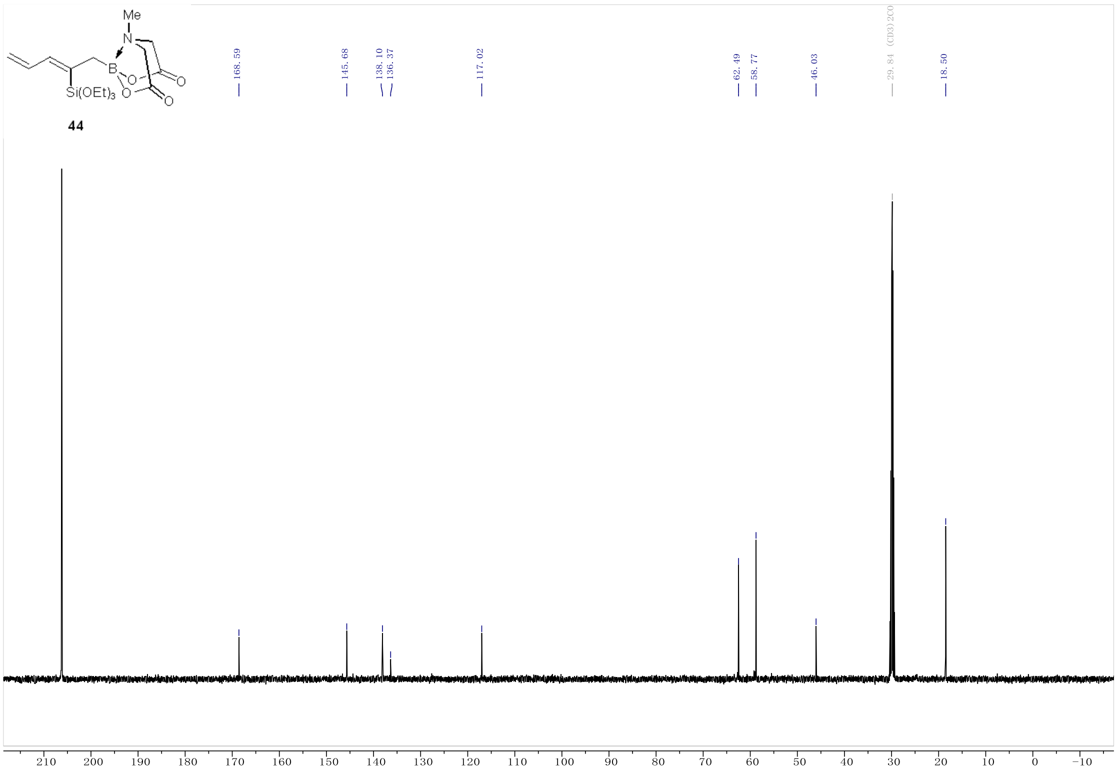

## $^{1}$H NMR of 46 (500 MHz, DMSO-$d_6$)

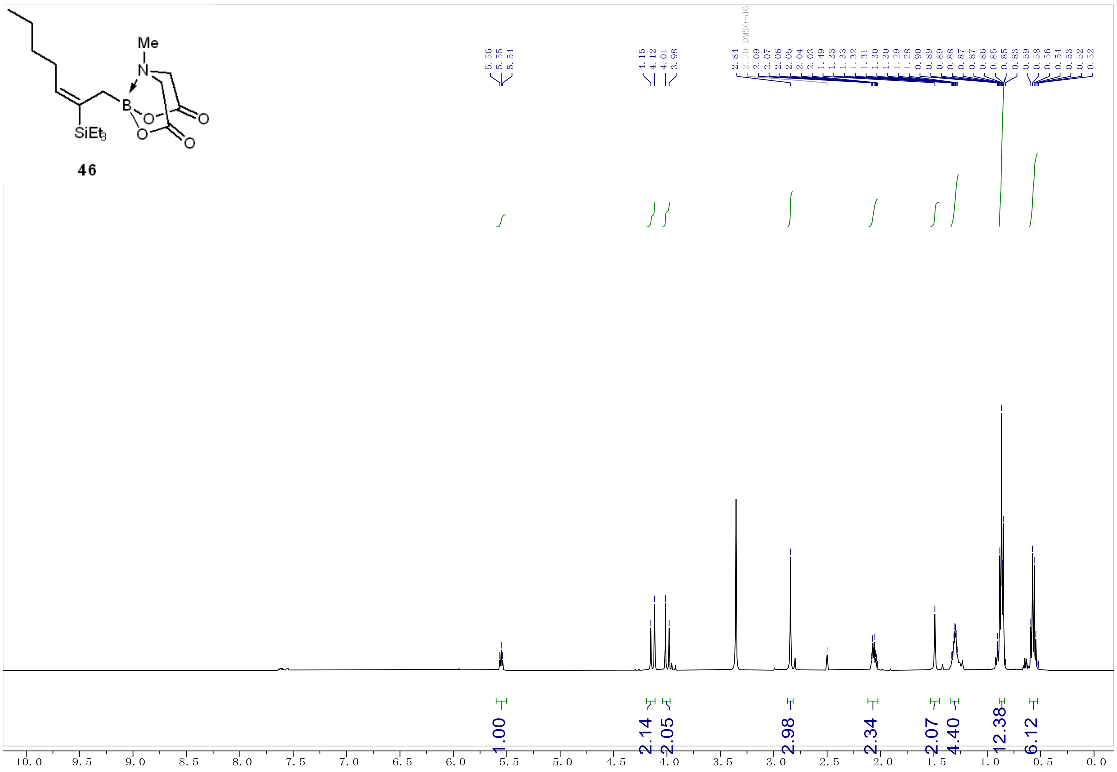

## $^{13}$C NMR of 46 (126 MHz, DMSO-$d_6$)

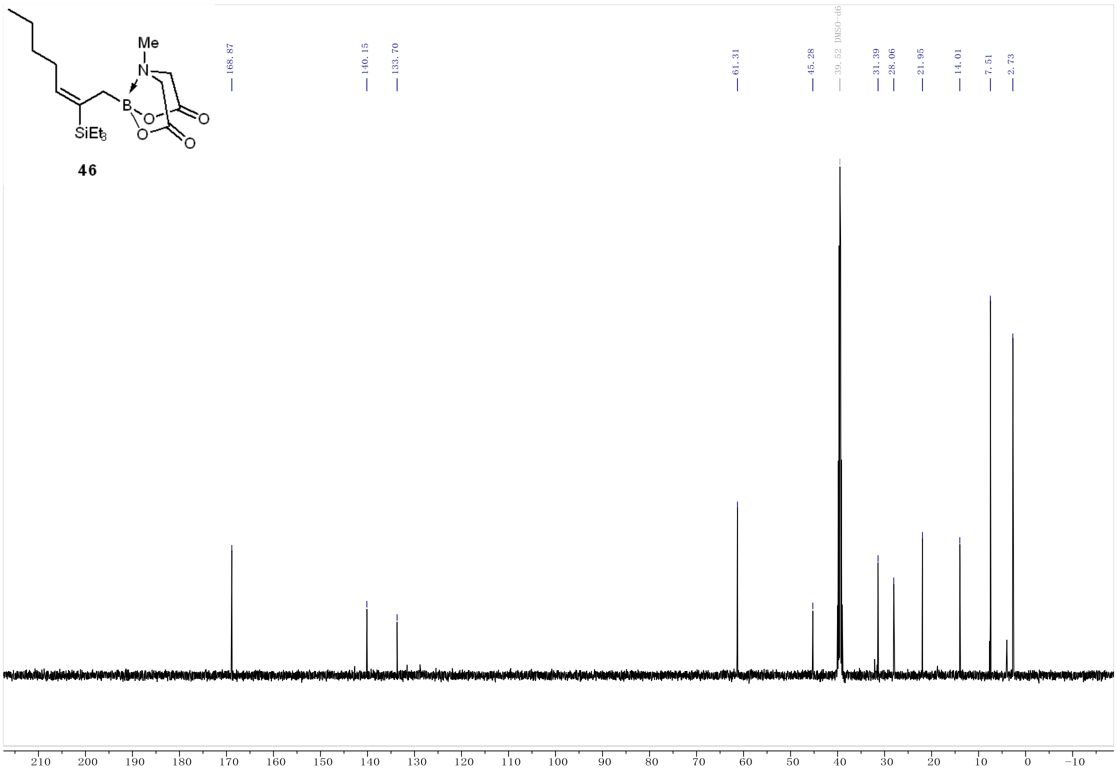

**$^{1}$H NMR of 47 (500 MHz, $CD_3CN$)**

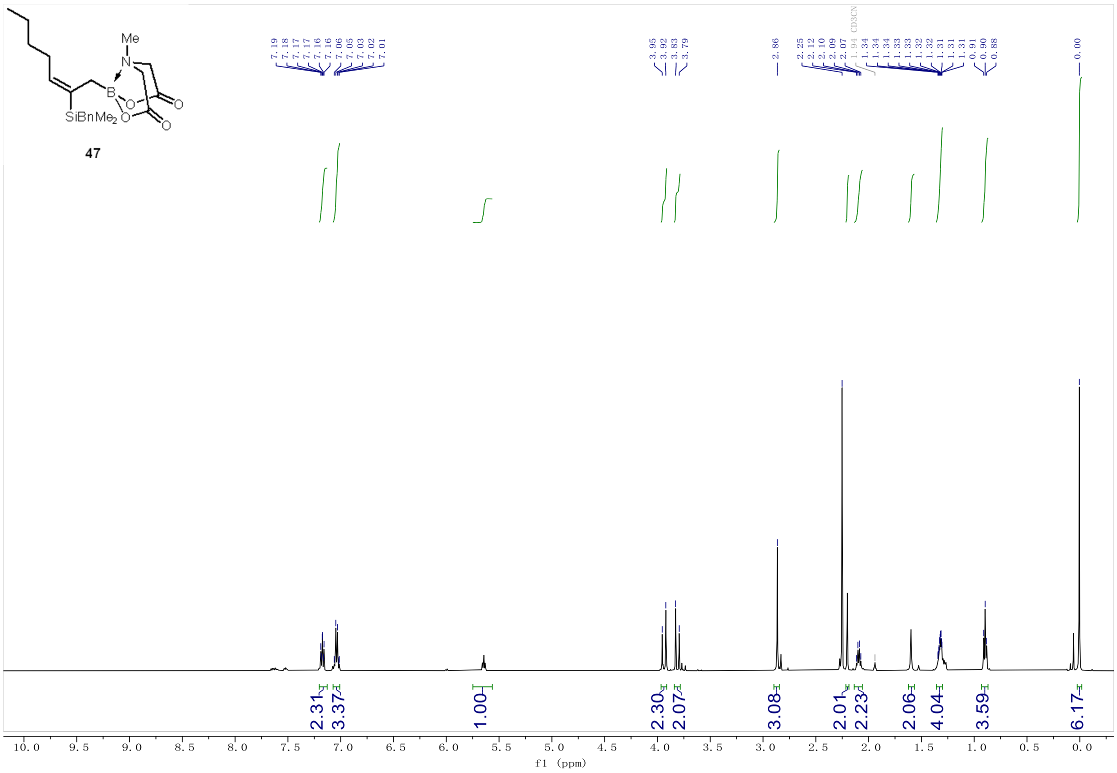

**$^{13}$C NMR of 47 (126 MHz, $CD_3CN$)**

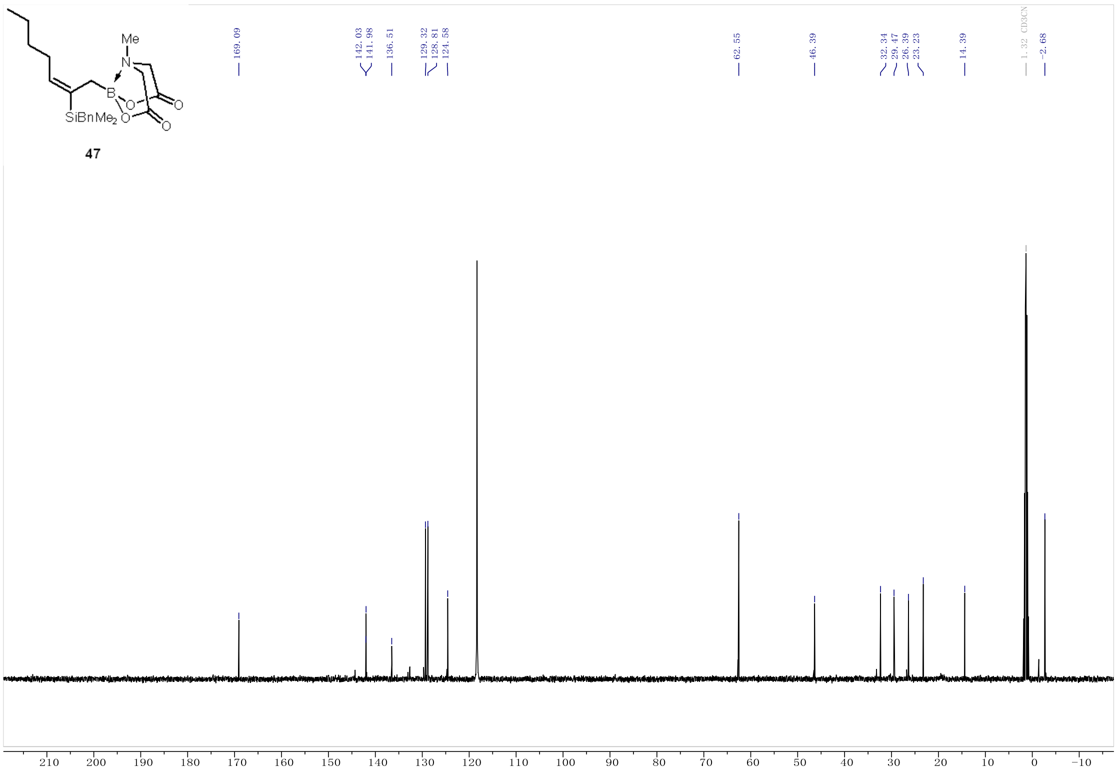

**$^{1}$H NMR of 48 (500 MHz, DMSO-$d_6$)**

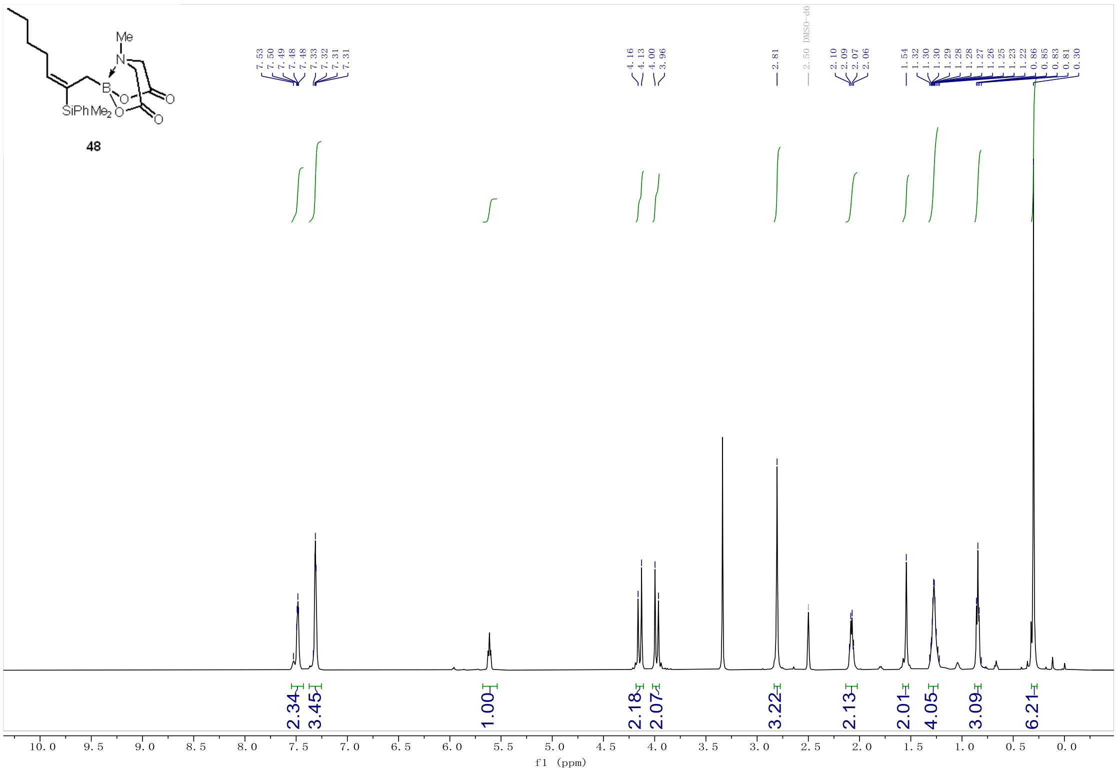

**$^{13}$C NMR of 48 (126 MHz, DMSO-$d_6$)**

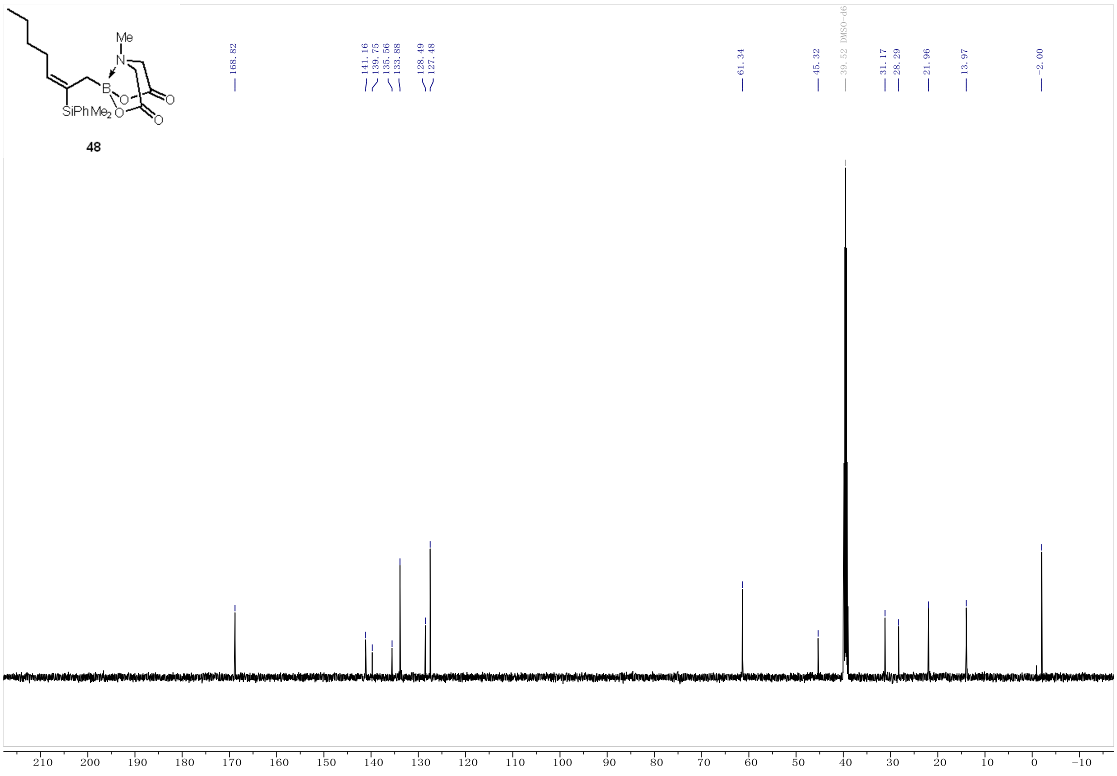

## $^1H$ NMR of 49 (400 MHz, Acetone-$d_6$)

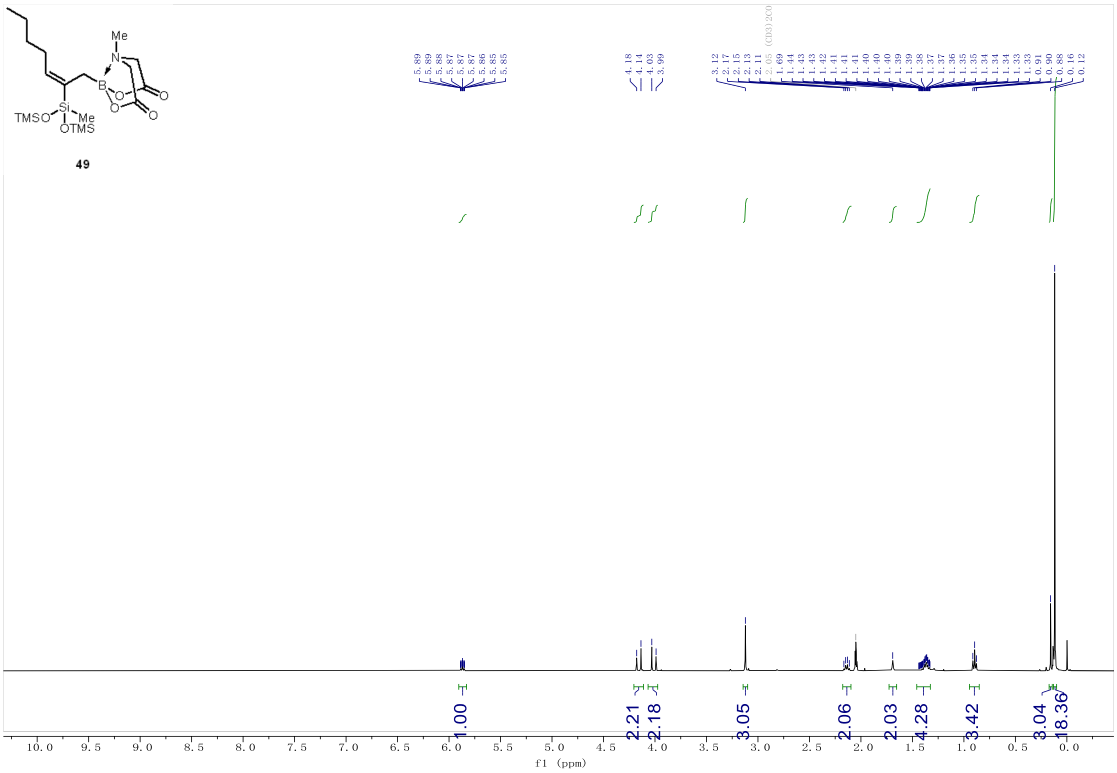

## $^{13}C$ NMR of 49 (101 MHz, Acetone-$d_6$)

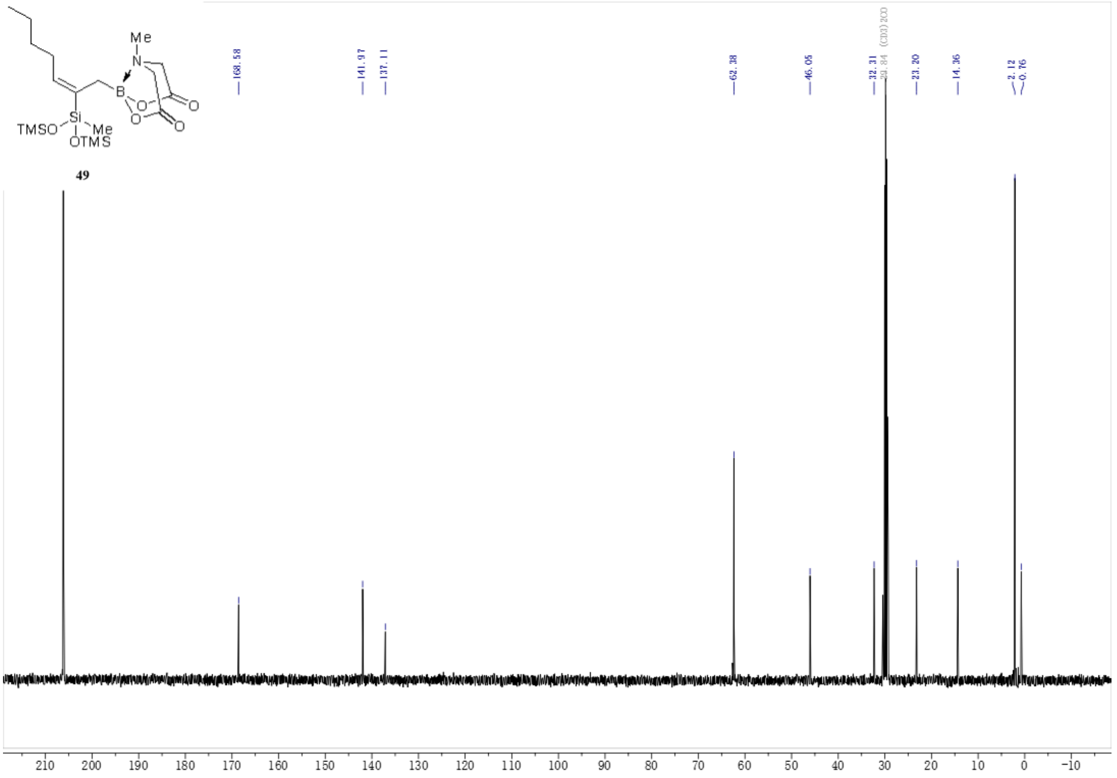

## $^{1}$H NMR of 50 (400 MHz, $CD_3CN$)

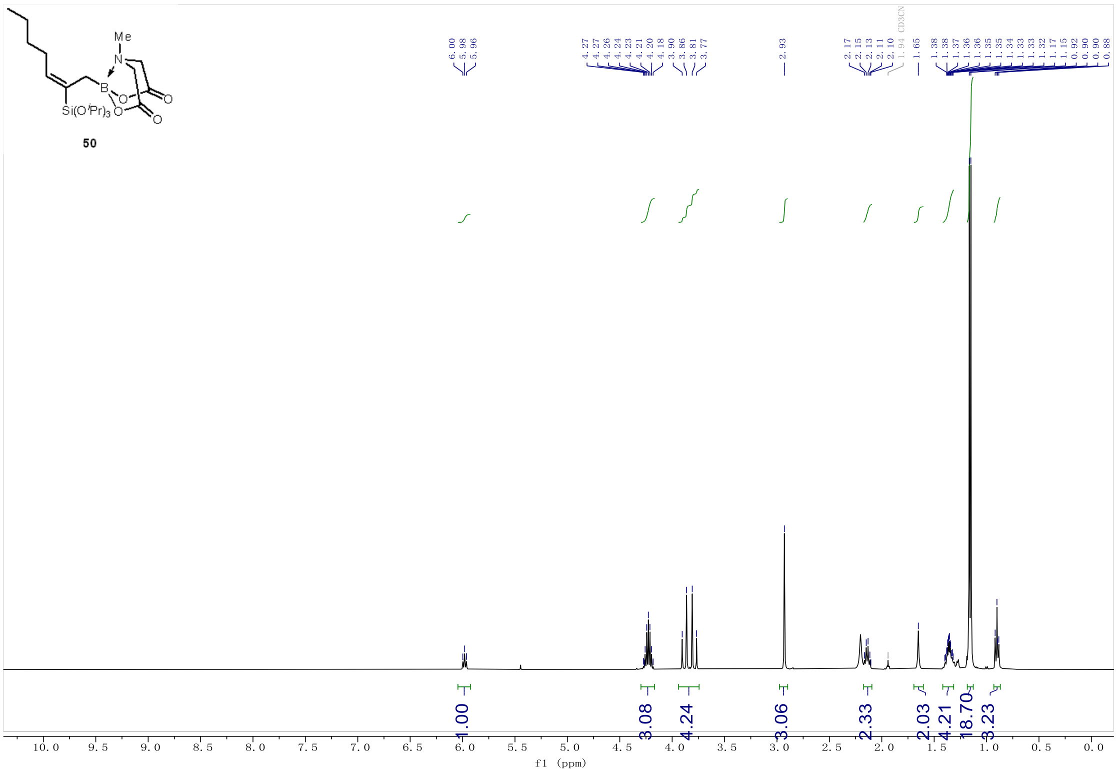

## $^{13}$C NMR of 50 (101 MHz, $CD_3CN$)

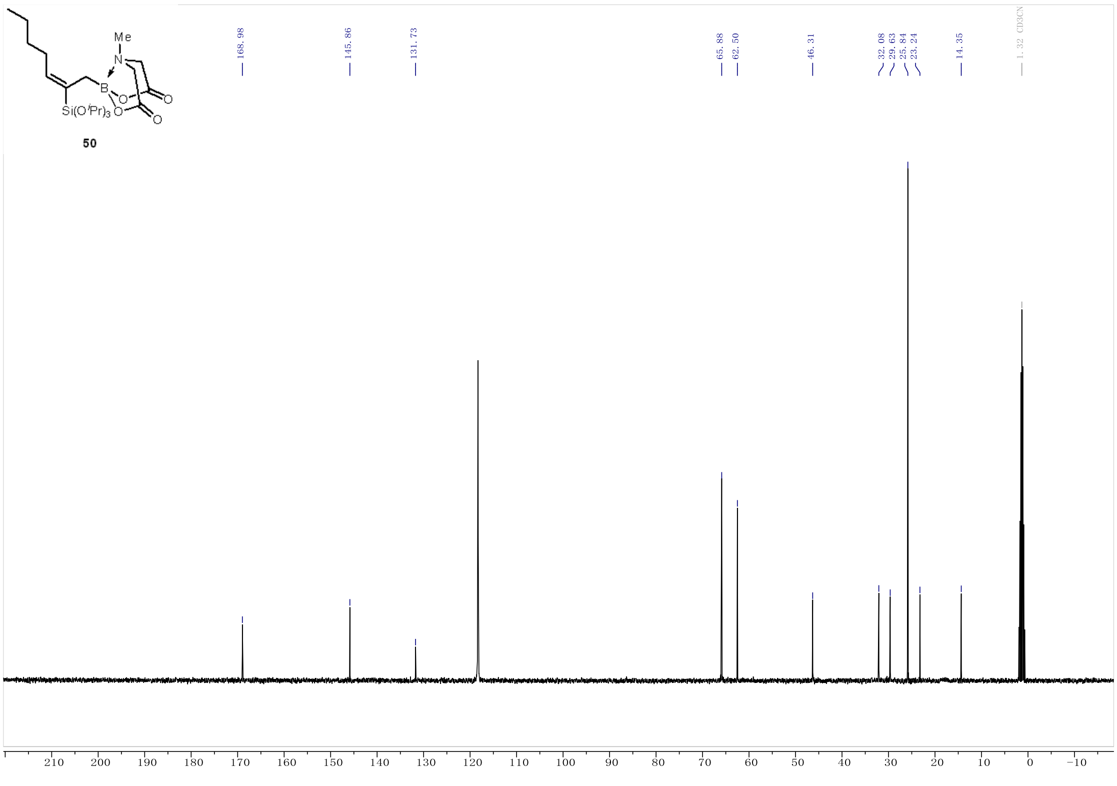

# $^{1}$H NMR of 51 (400 MHz, Acetone-$d_6$)

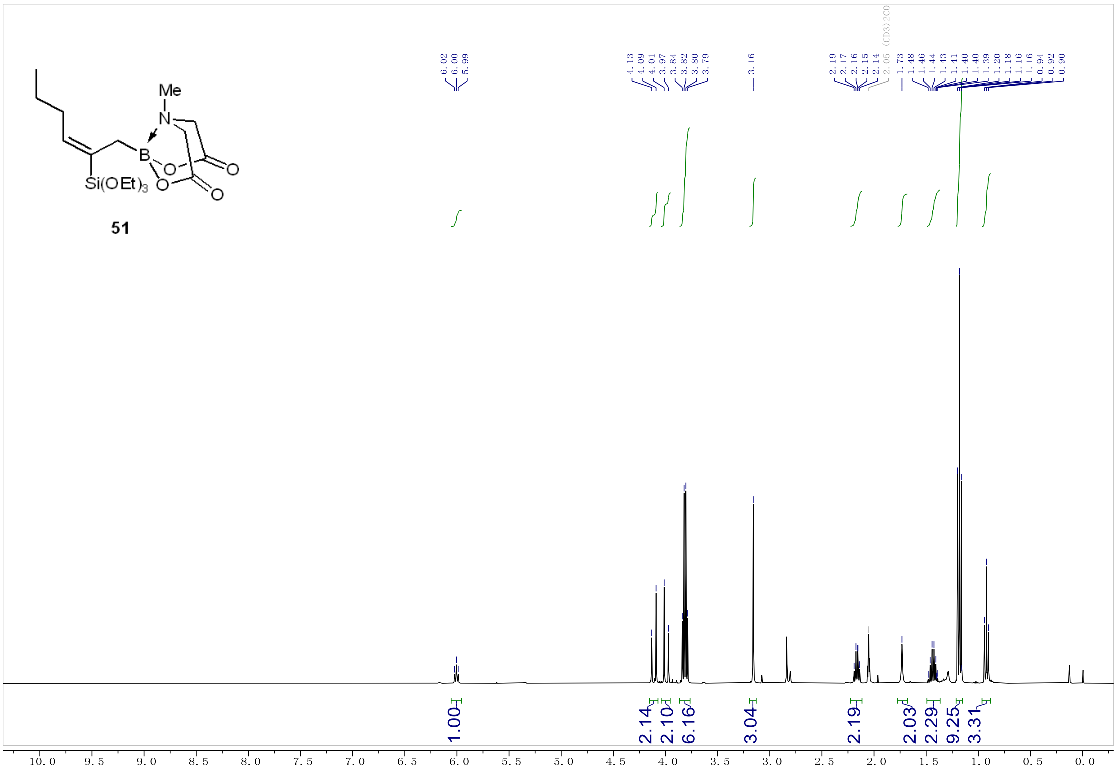


# $^{13}$C NMR of 51 (101 MHz, Acetone-$d_6$)

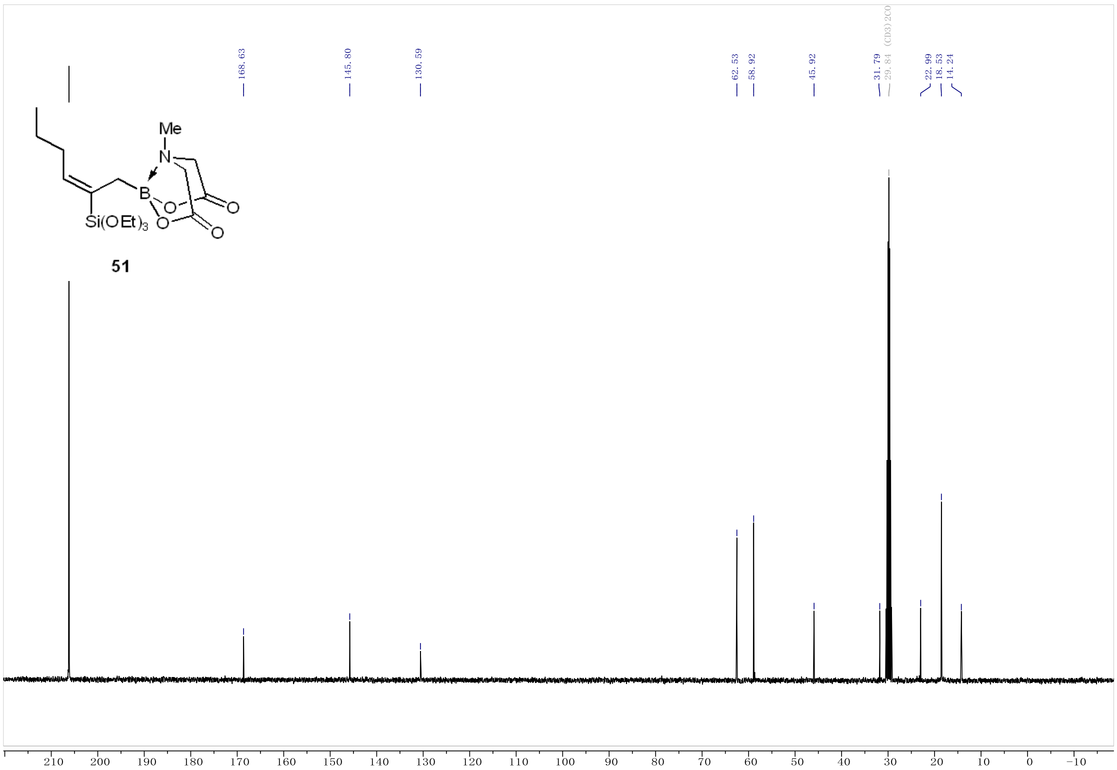

## $^1$H NMR of 52 (500 MHz, Acetone-$d_6$)

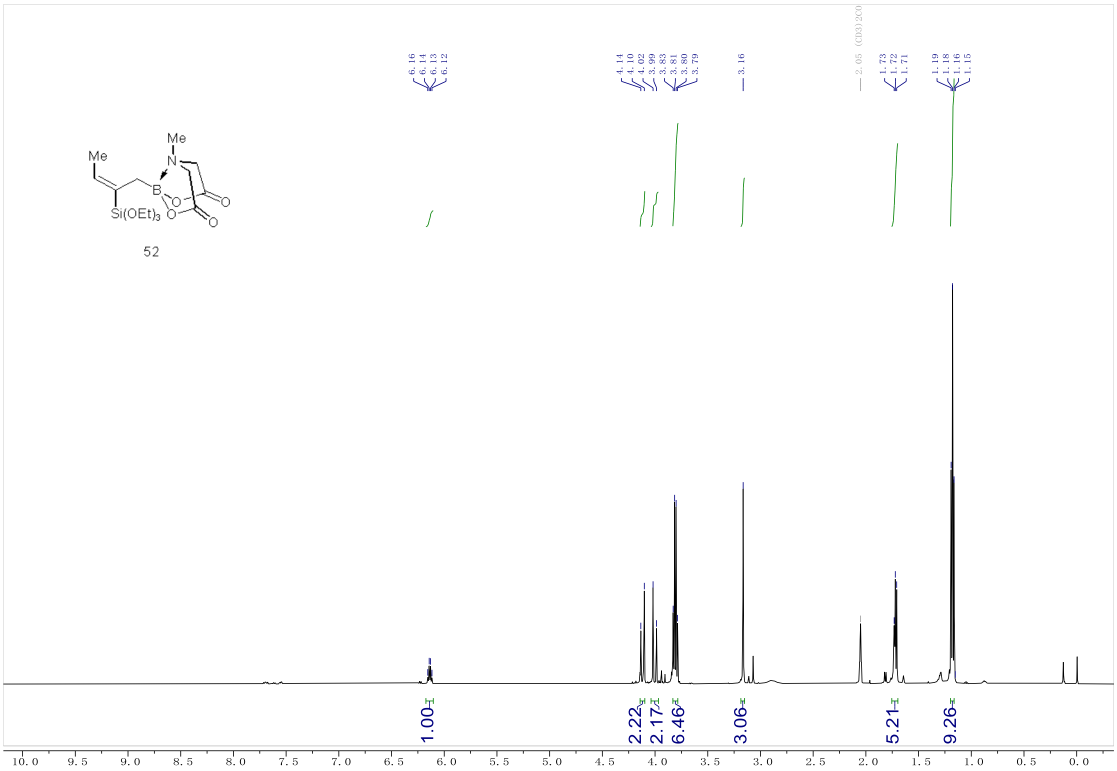


## $^{13}$C NMR of 52 (126 MHz, Acetone-$d_6$)

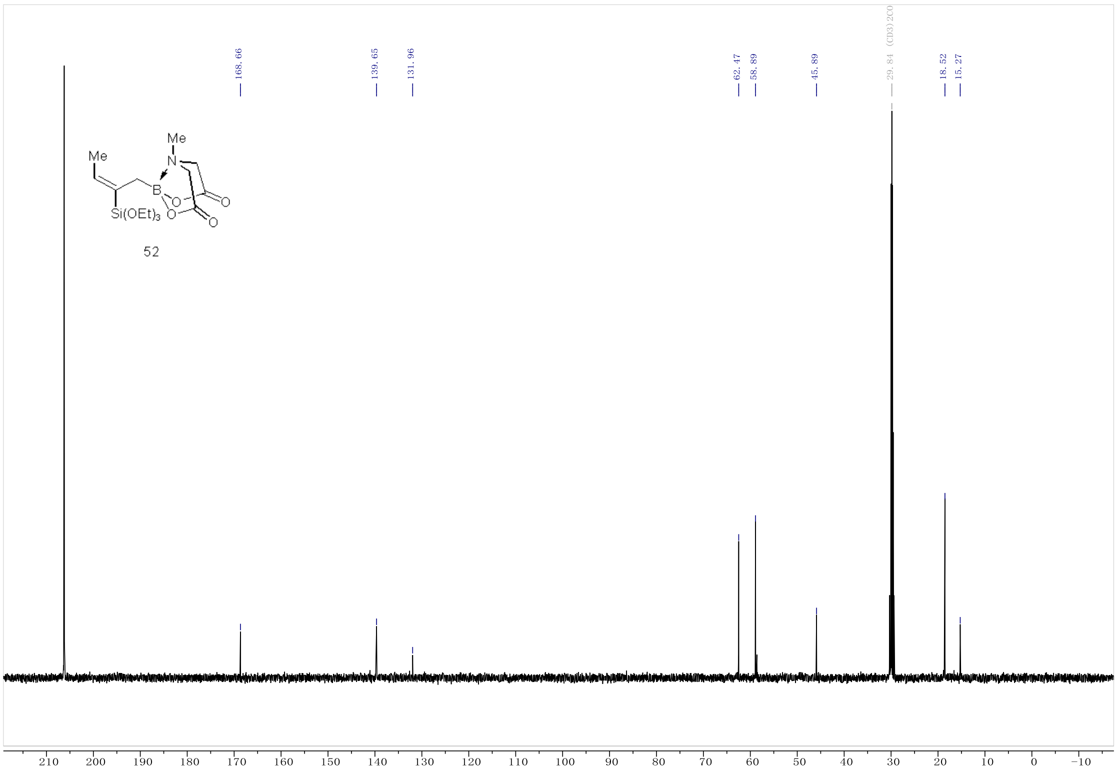

$^1$H NMR of 53 (400 MHz, Acetone-$d_6$)

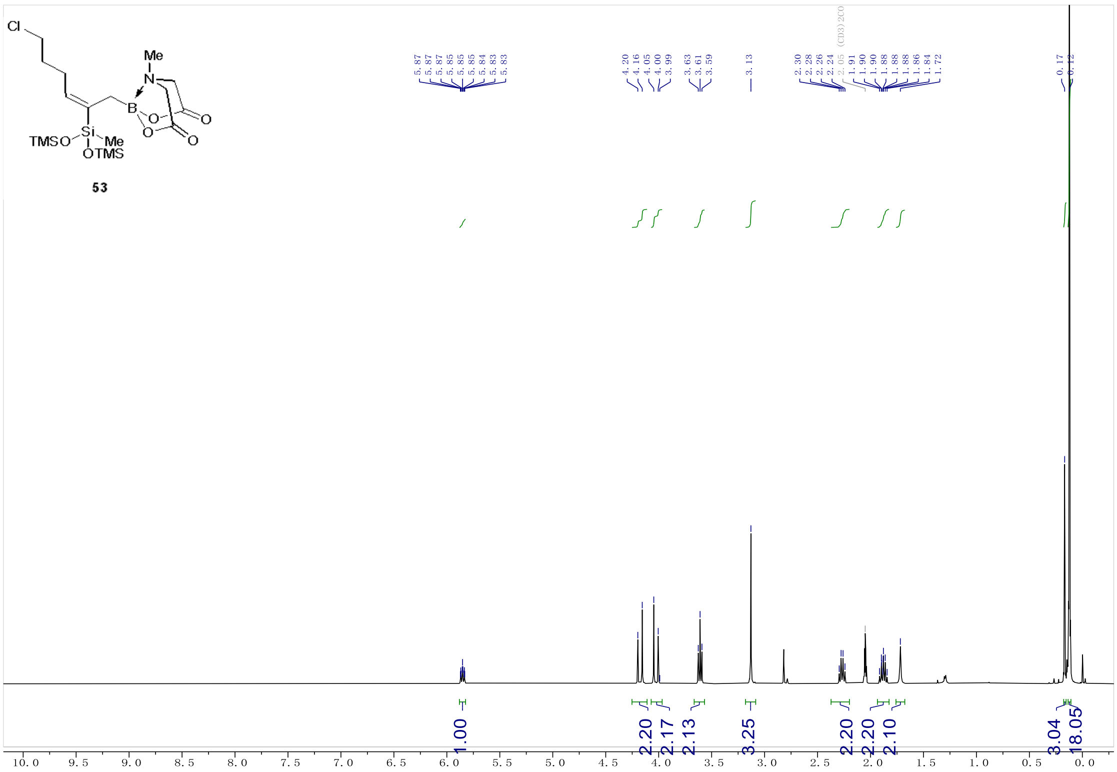

$^{13}$C NMR of 53 (101 MHz, Acetone-$d_6$)

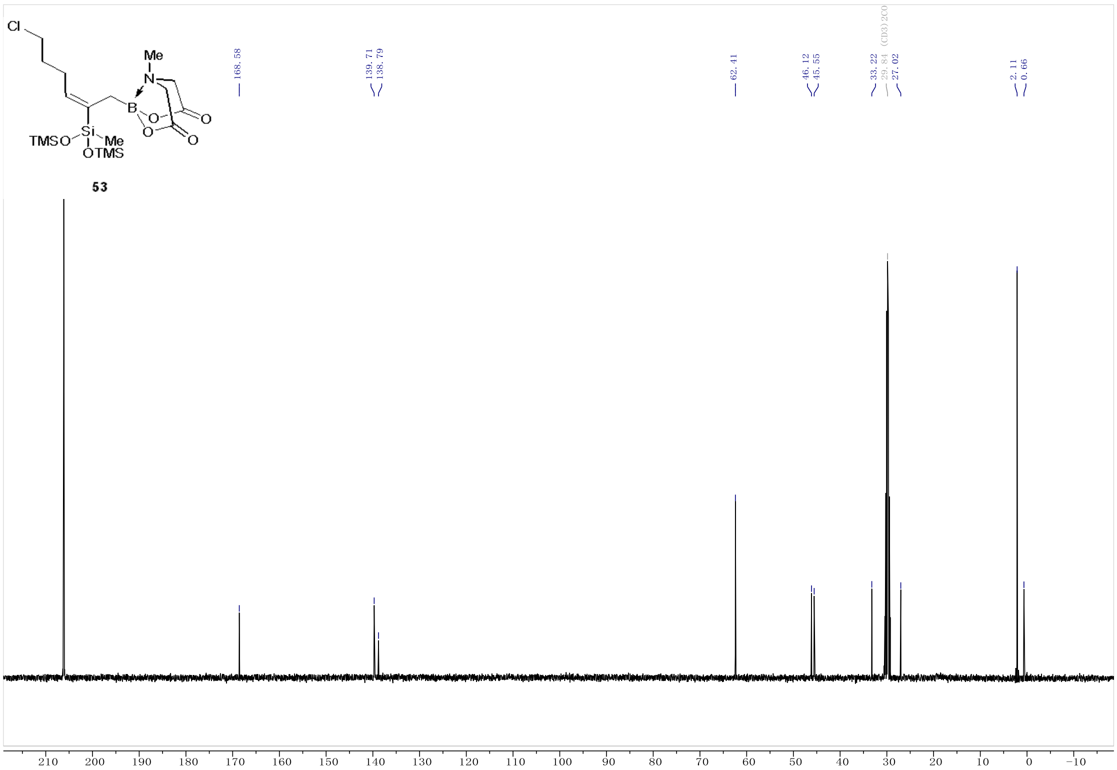

## $^{1}$H NMR of 54

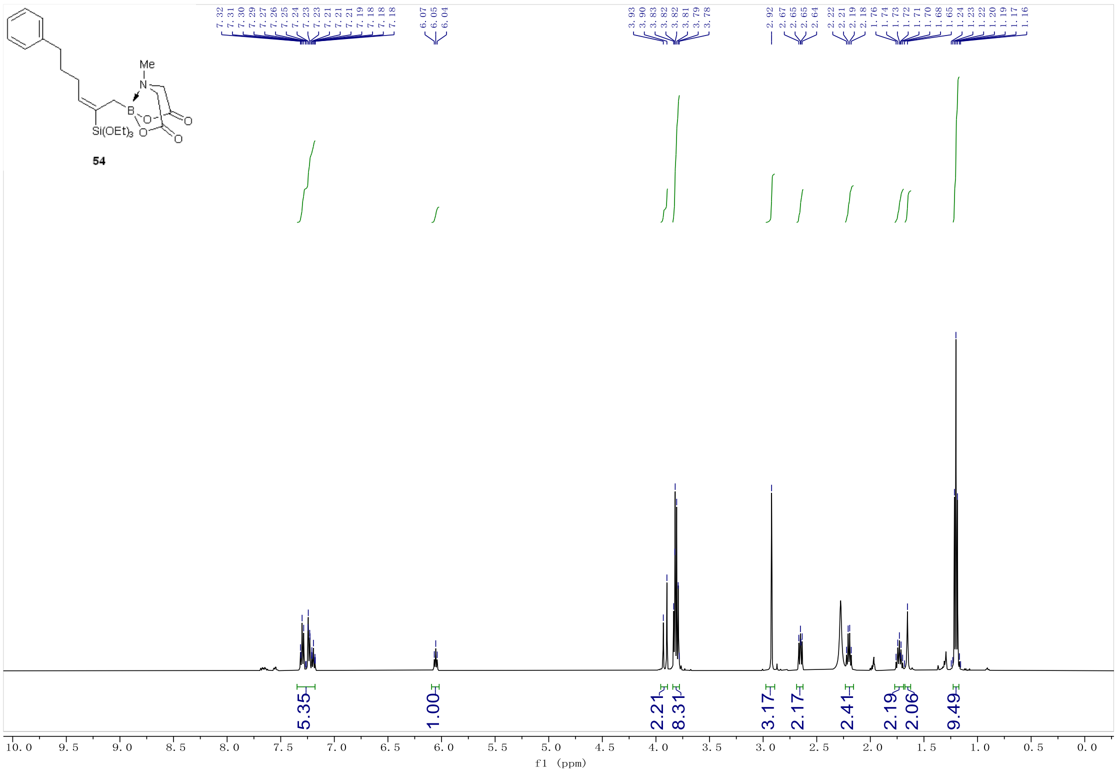

## $^{13}$C NMR of 54

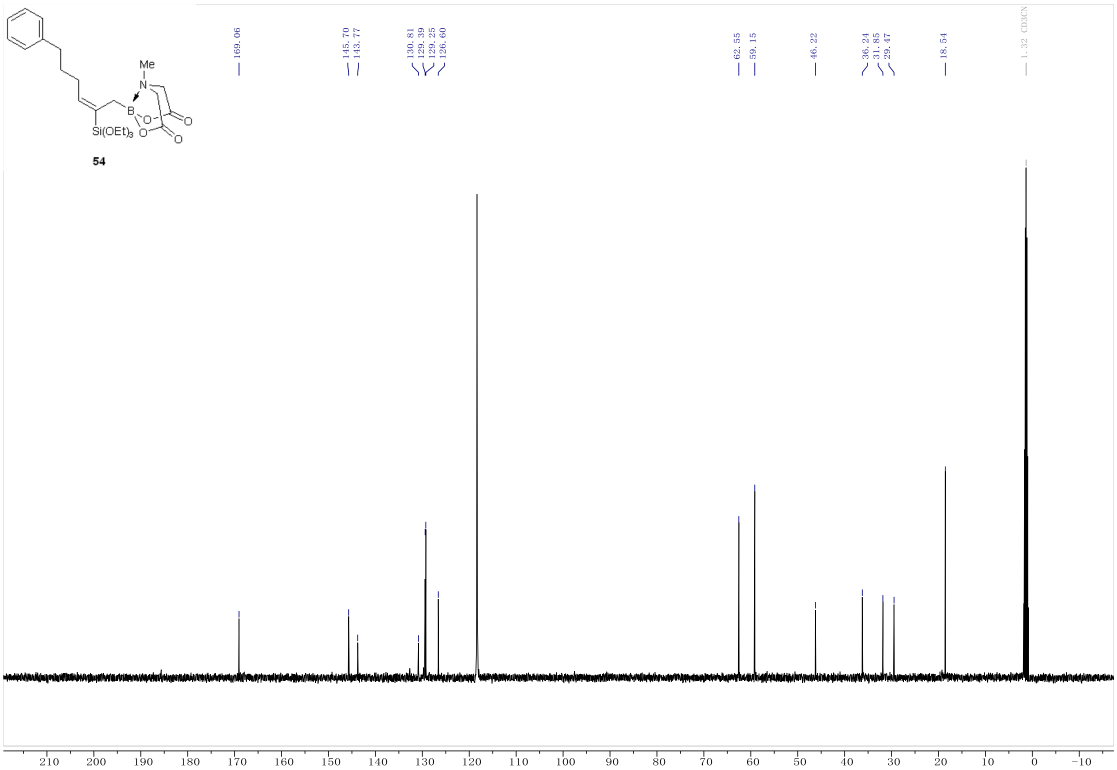

## $^{11}$B NMR of 54

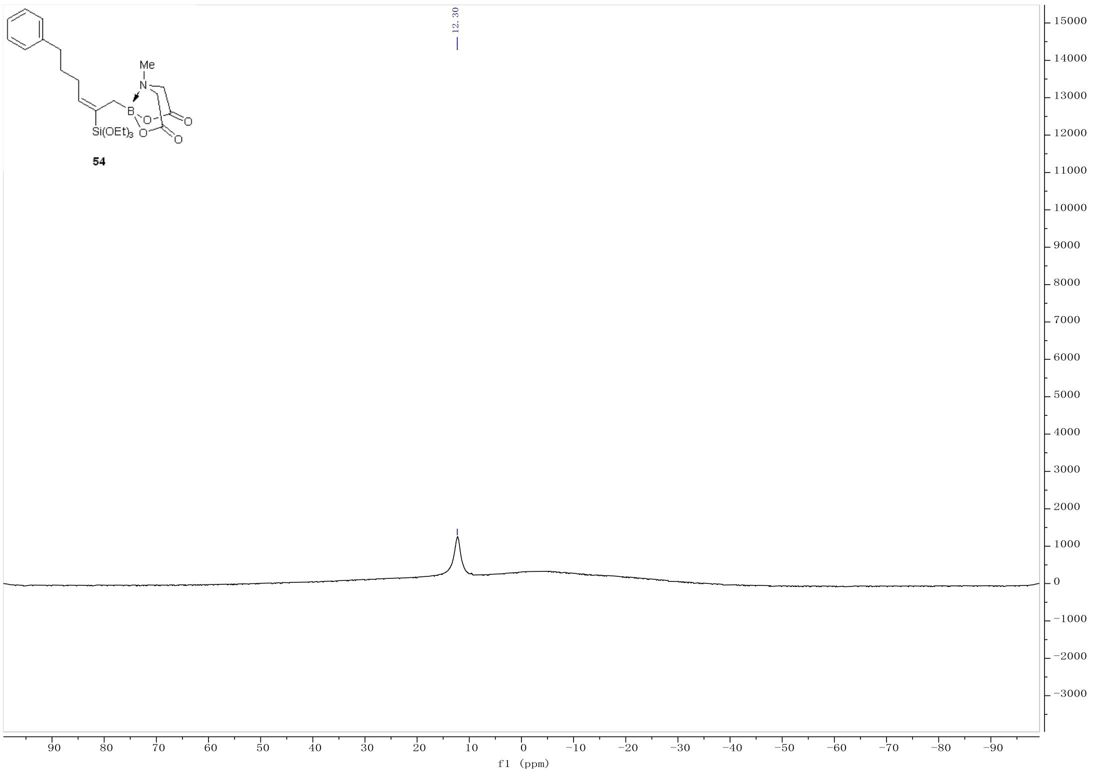

## $^{1}$H NMR of 55 (400 MHz, $CD_3CN$)

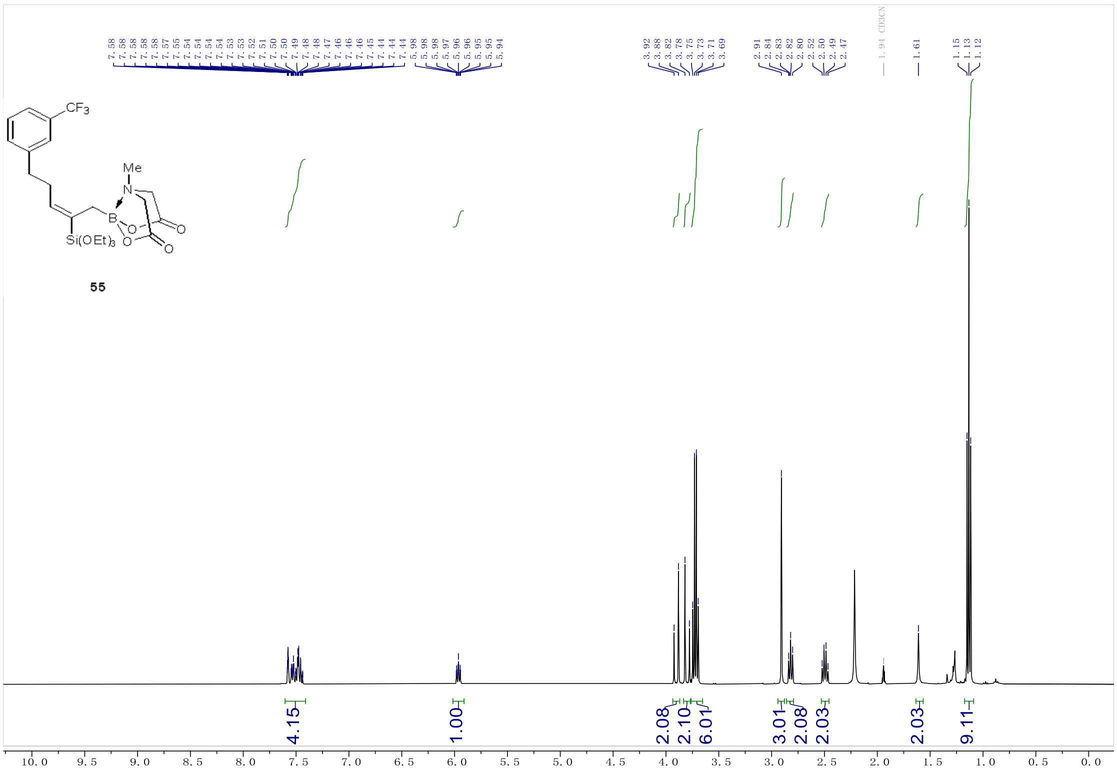

## $^{13}C$ NMR of 55 (101 MHz, $CD_3CN$)

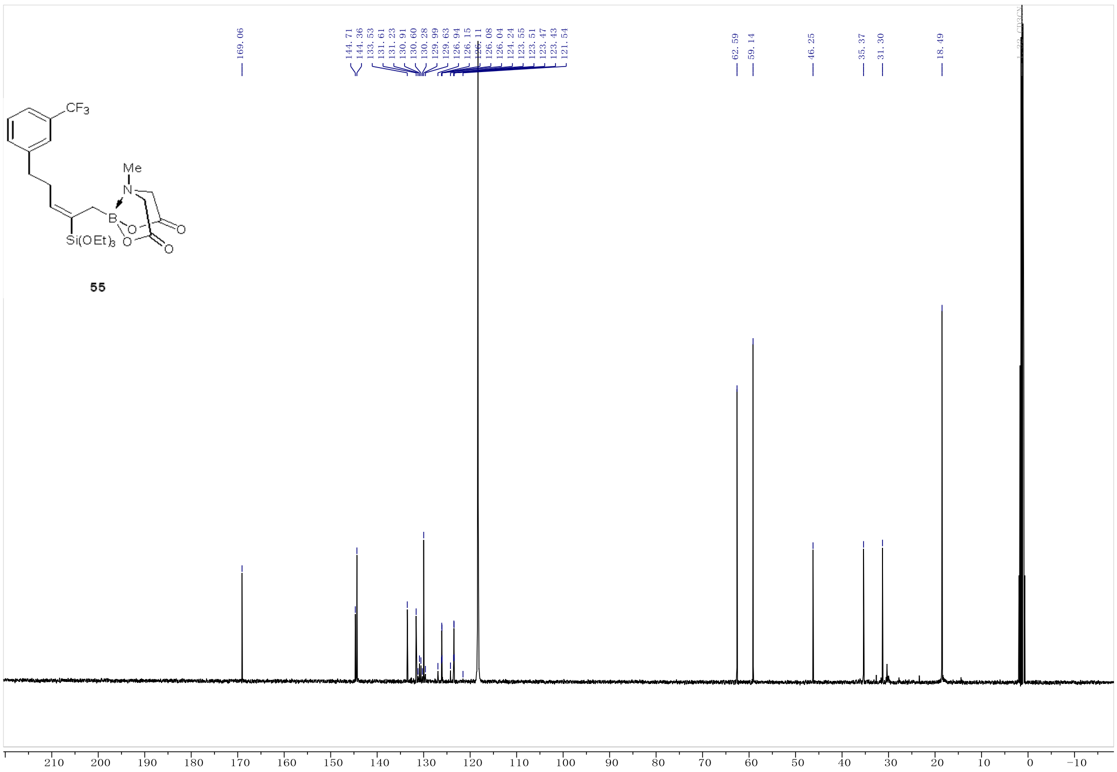


## $^{19}F$ NMR of 55 (376 MHz, $CD_3CN$)

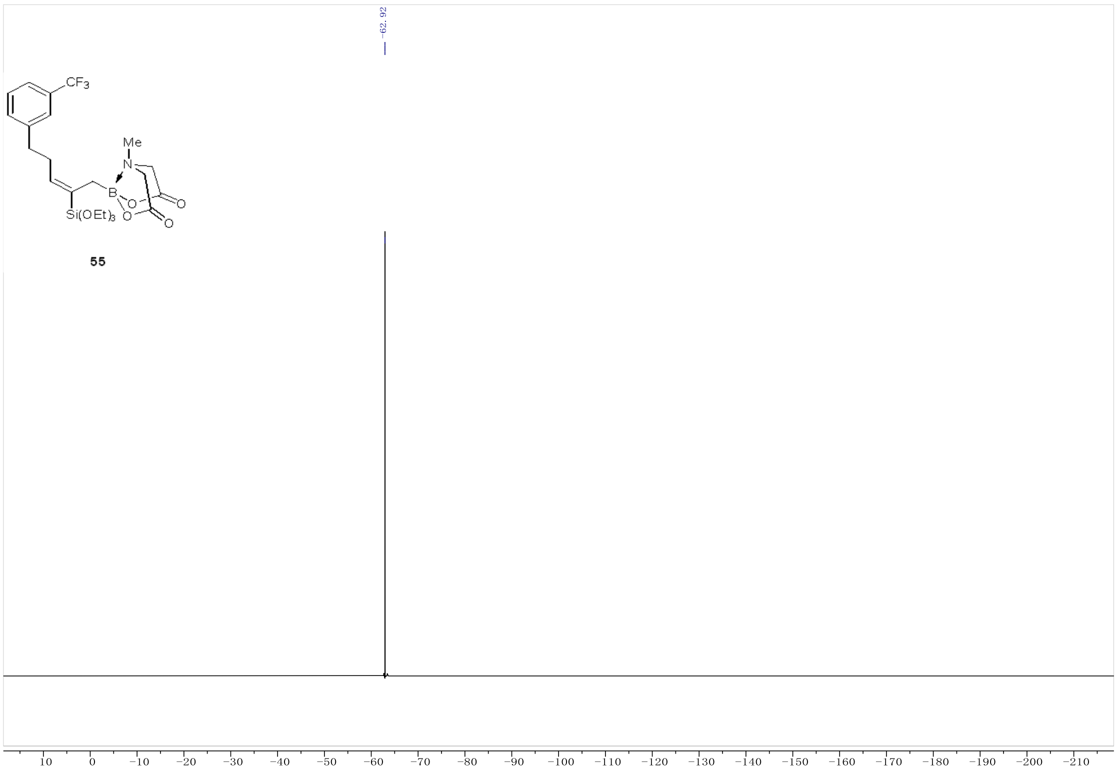

## $^1$H NMR of 56 (400 MHz, Acetone-$d_6$)

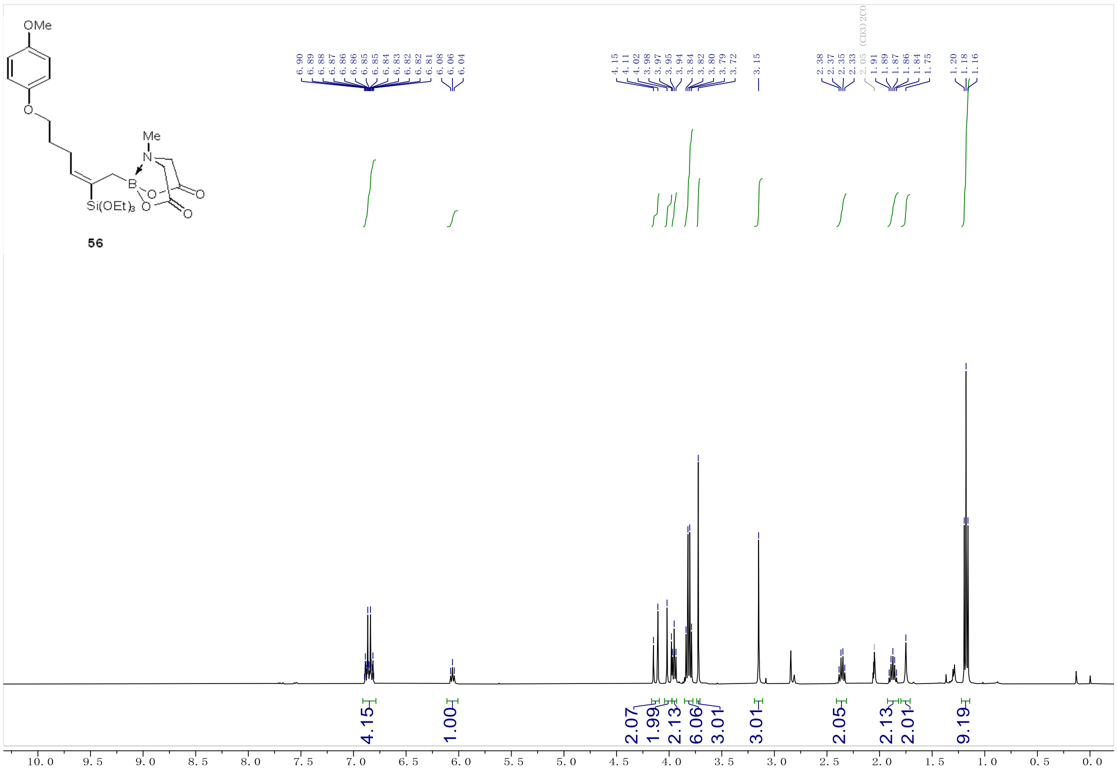


## $^{13}$C NMR of 56 (101 MHz, Acetone-$d_6$)

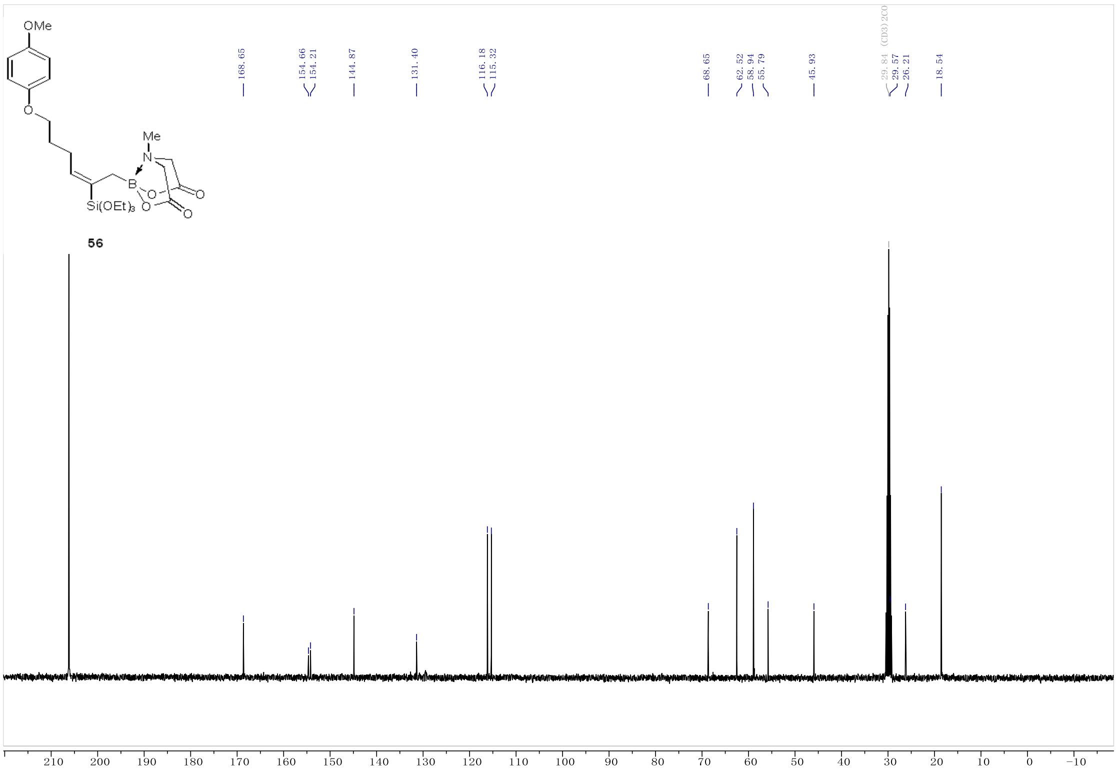

## $^{1}$H NMR of 57 (500 MHz, $CD_3CN$)

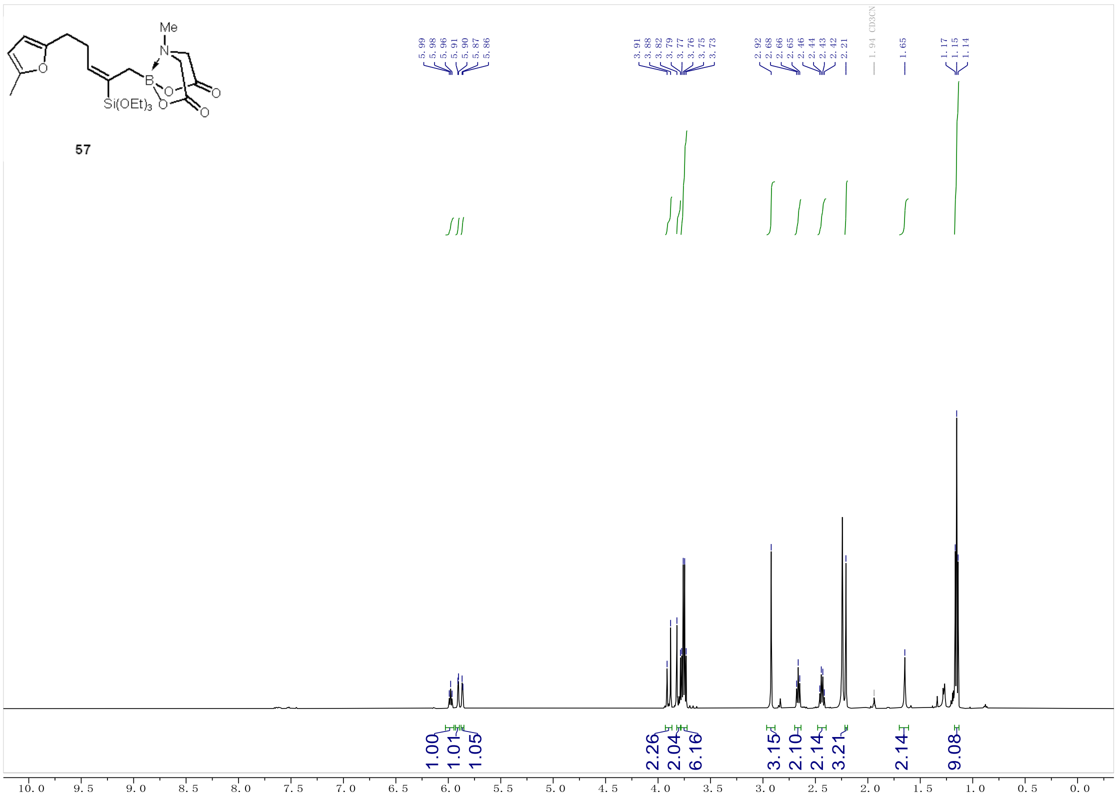


## $^{13}$C NMR of 57 (126 MHz, $CD_3CN$)

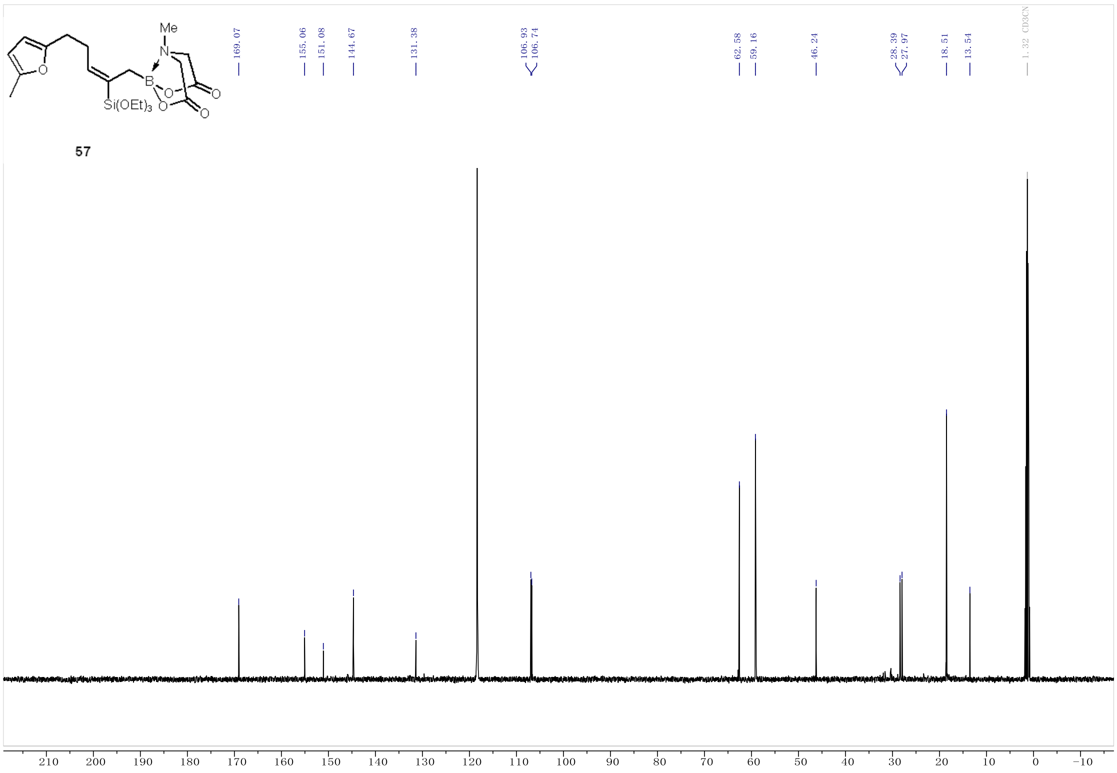

**NOE of 57**

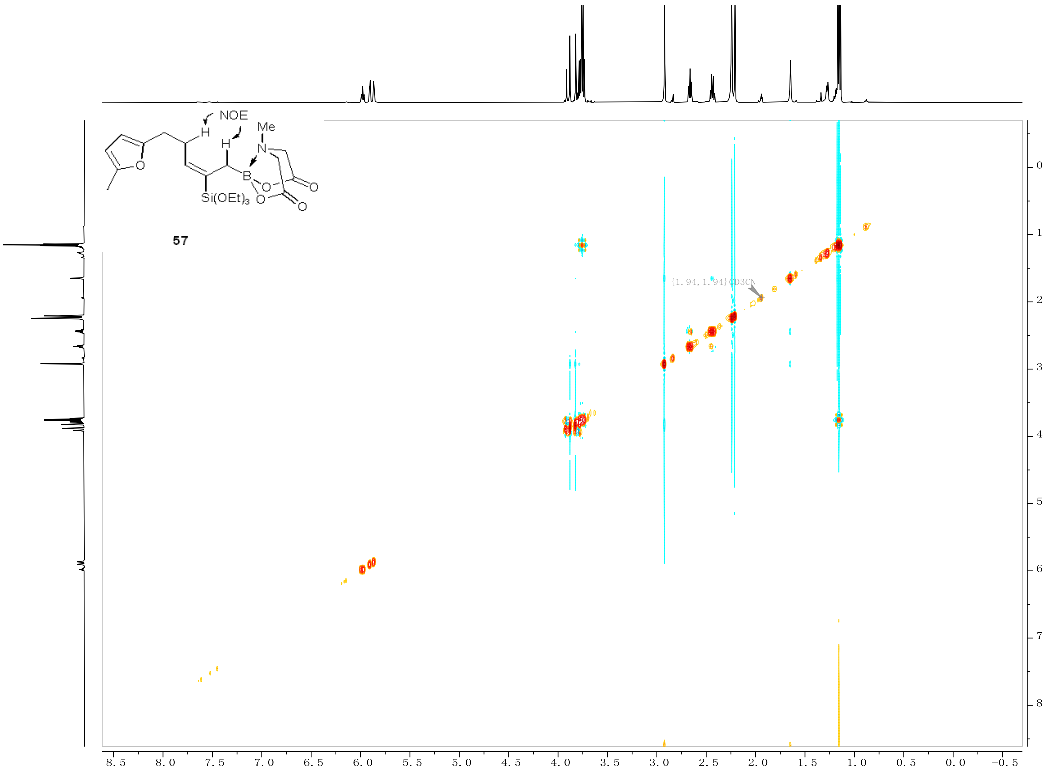

NOE
Me
N
H
H
B
O
O
O
O
Si(OEt)3
57
(1.94, 1.94) CD3CN
f1 (ppm)


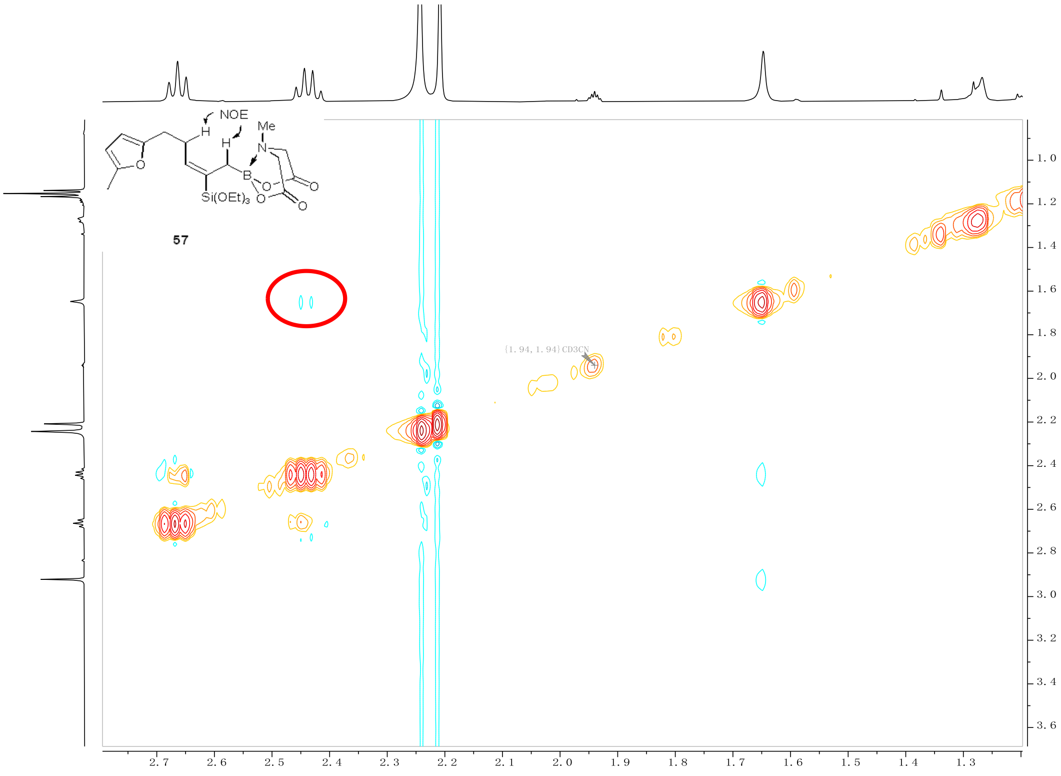

NOE
Me
N
H
H
B
O
O
O
O
Si(OEt)3
57
(1.94, 1.94) CD3CN
f1 (ppm)

## $^{1}$H NMR of 58 (500 MHz, Acetone-$d_6$)

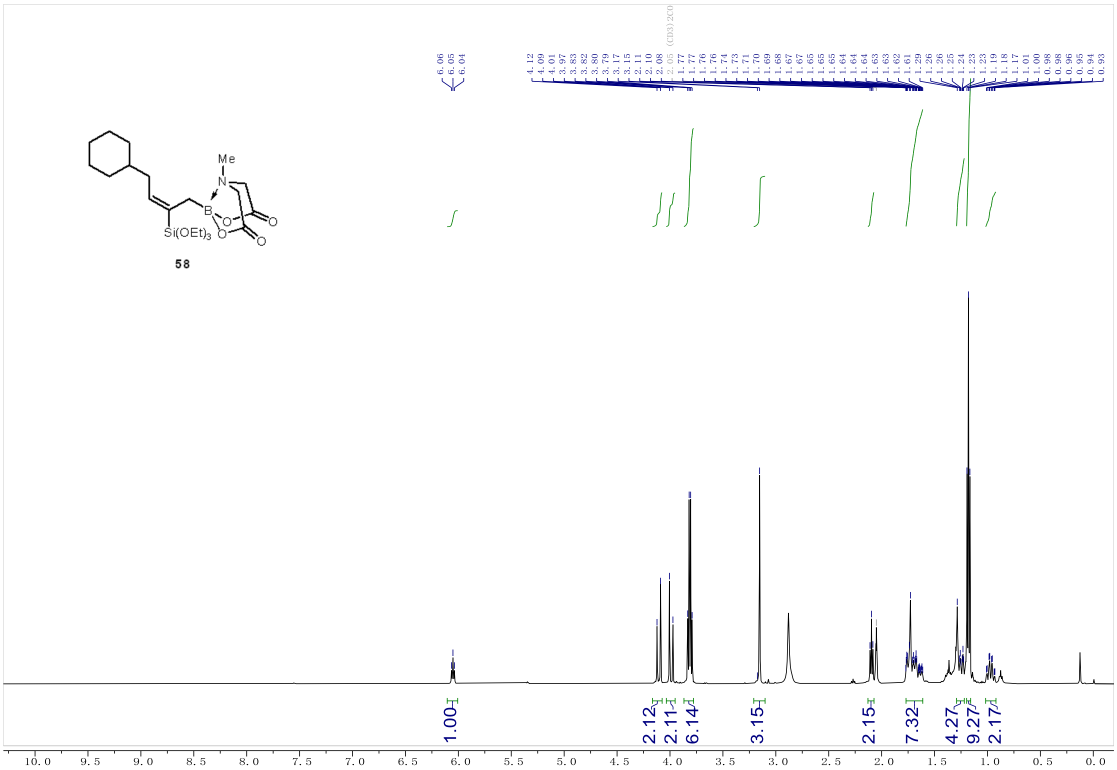

## $^{13}$C NMR of 58 (126 MHz, Acetone-$d_6$)

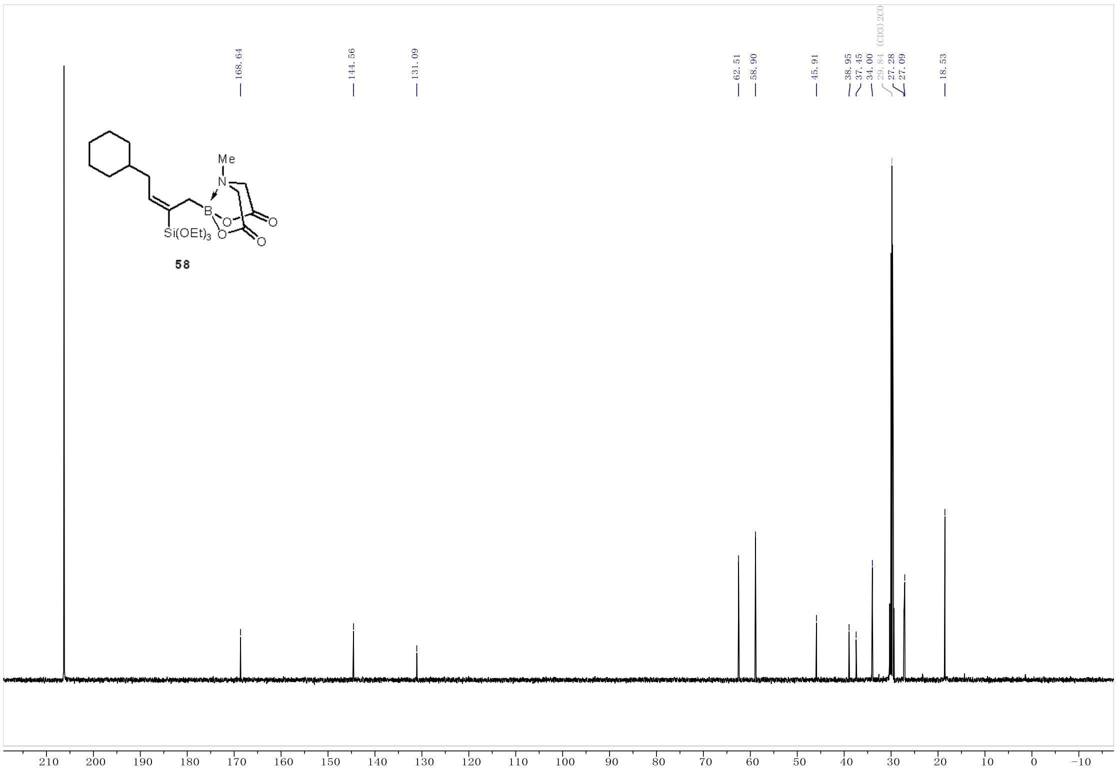

$^{1}H$ NMR of 59 (500 MHz, Acetone-$d_6$)

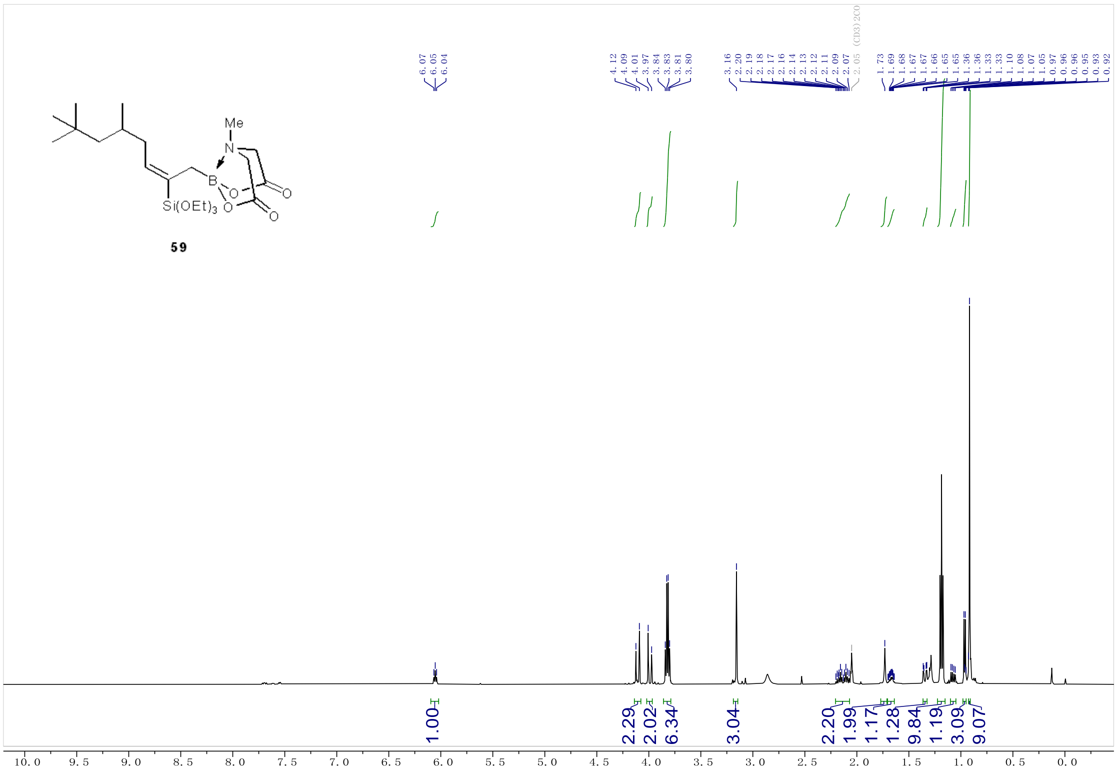

$^{13}C$ NMR of 59 (126 MHz, Acetone-$d_6$)

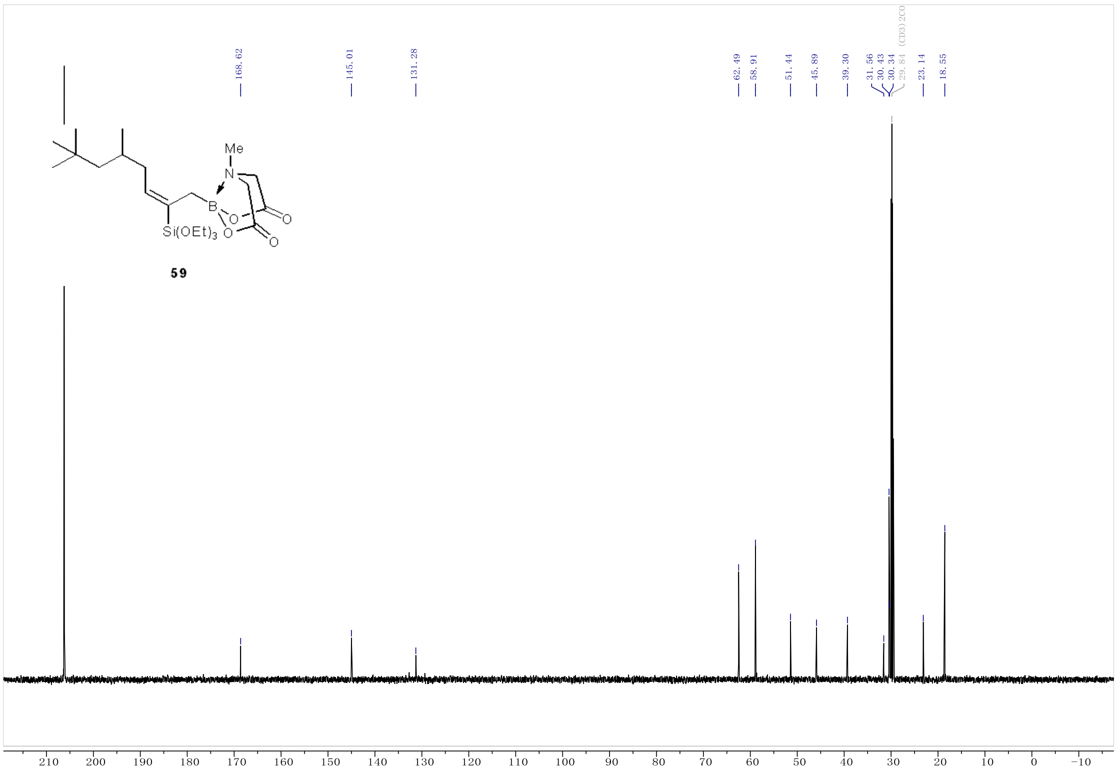

**¹H NMR of 60 (400 MHz, Acetone-$d_6$)**

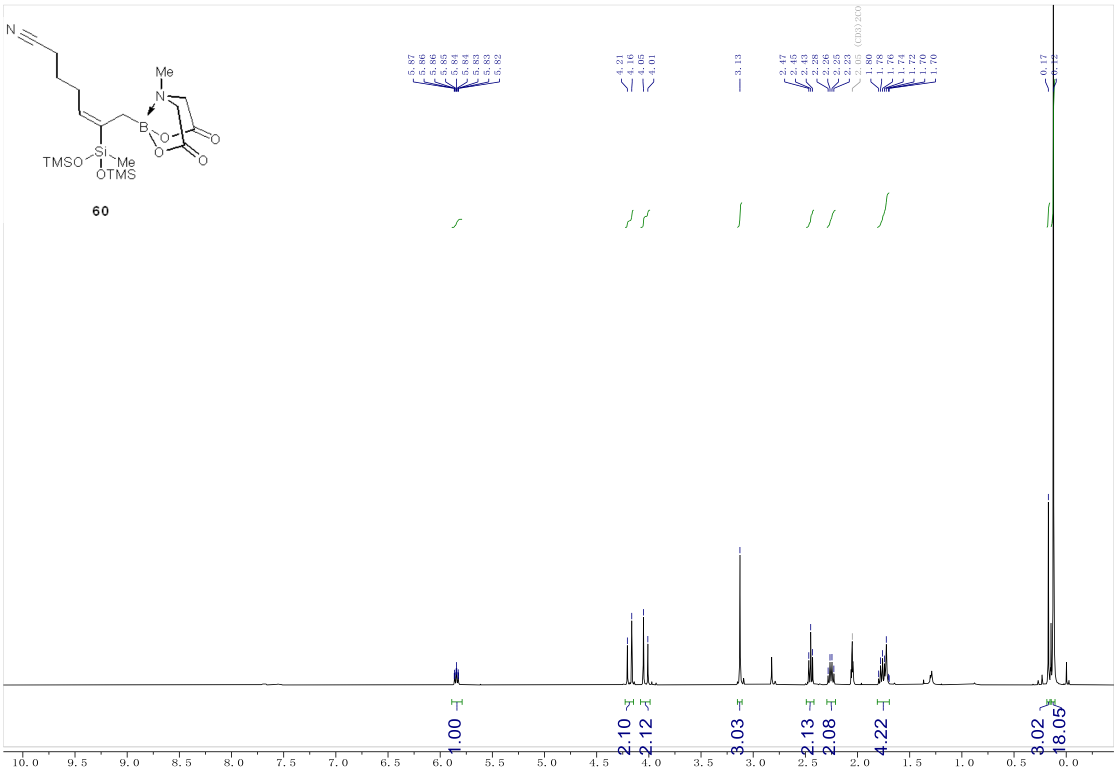

**¹³C NMR of 60 (101 MHz, Acetone-$d_6$)**

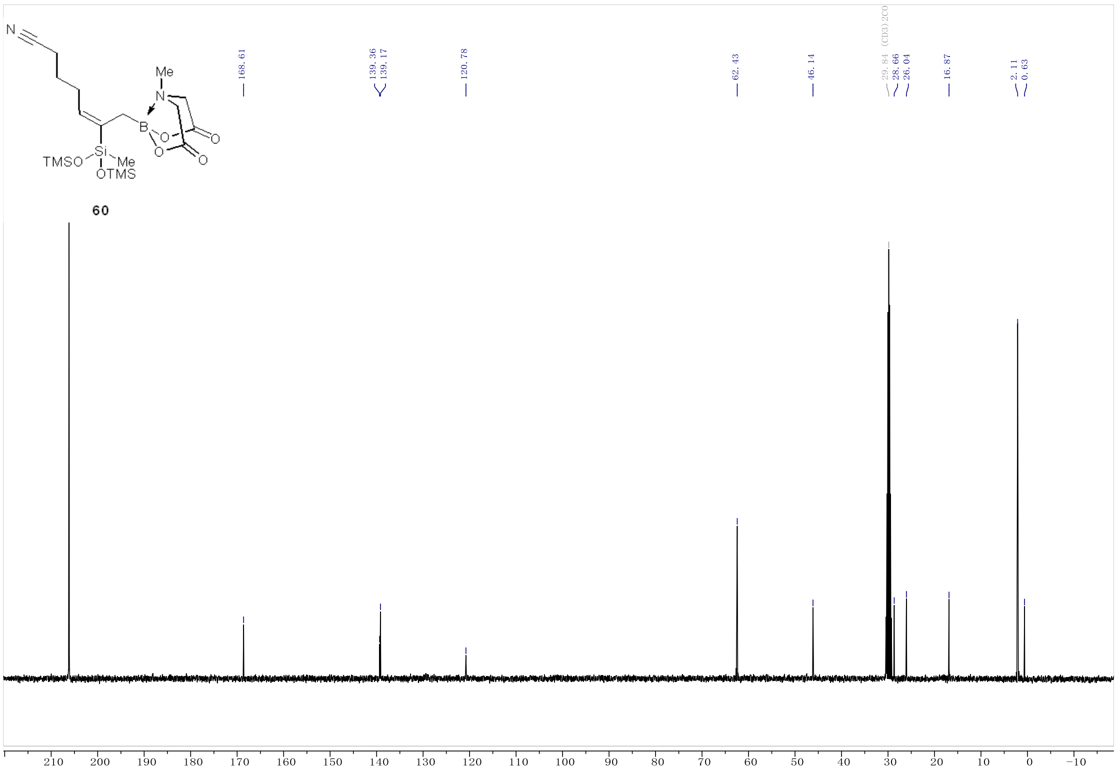

## $^{1}$H NMR of 61 (400 MHz, Acetone-$d_6$)

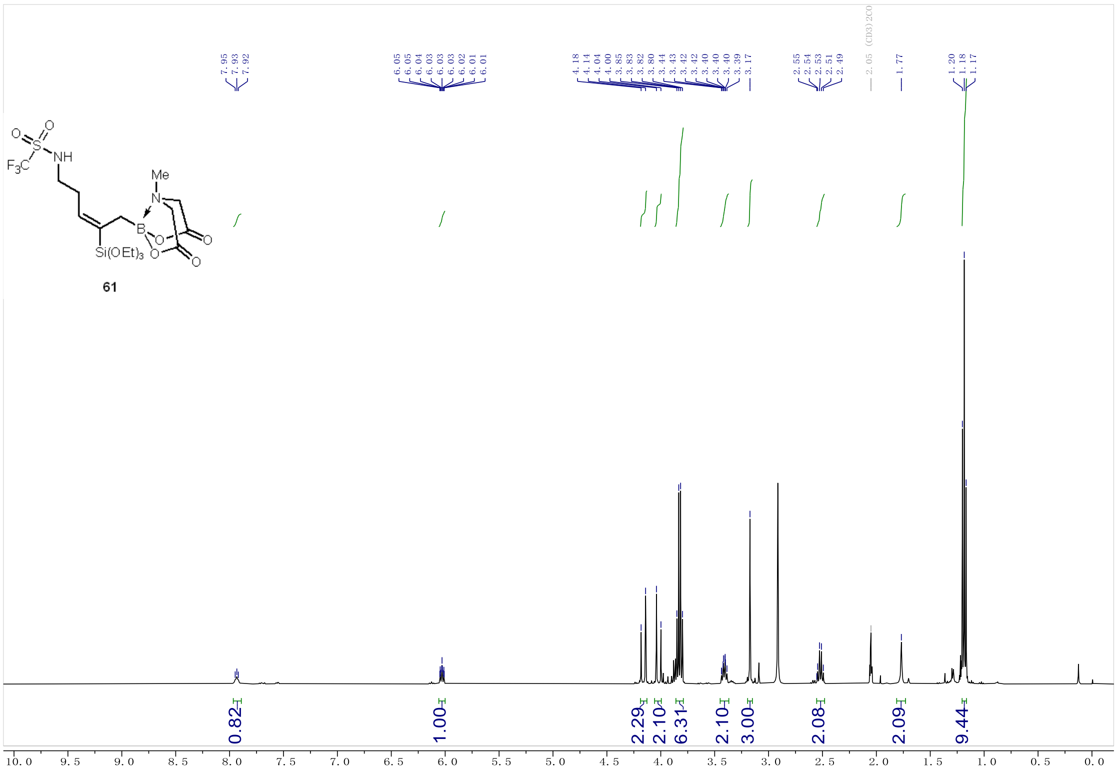

## $^{13}$C NMR of 61 (101 MHz, Acetone-$d_6$)

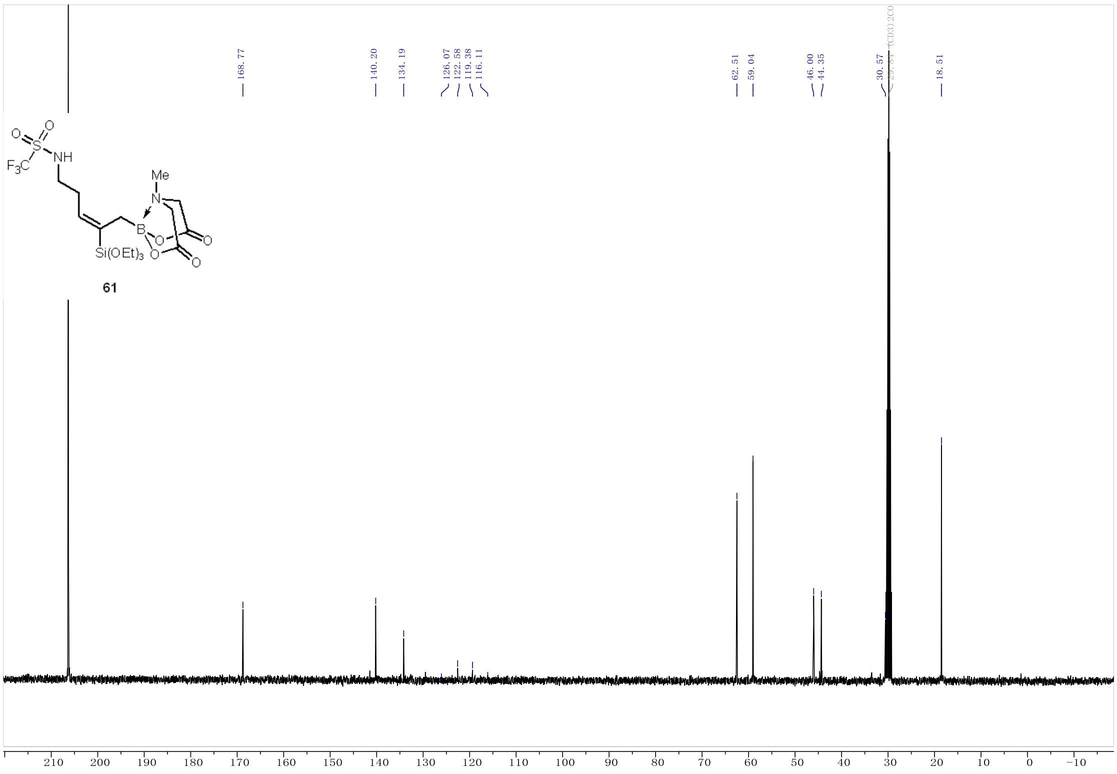

## $^{19}$F NMR of 61 (374 MHz, Acetone-$d_6$)

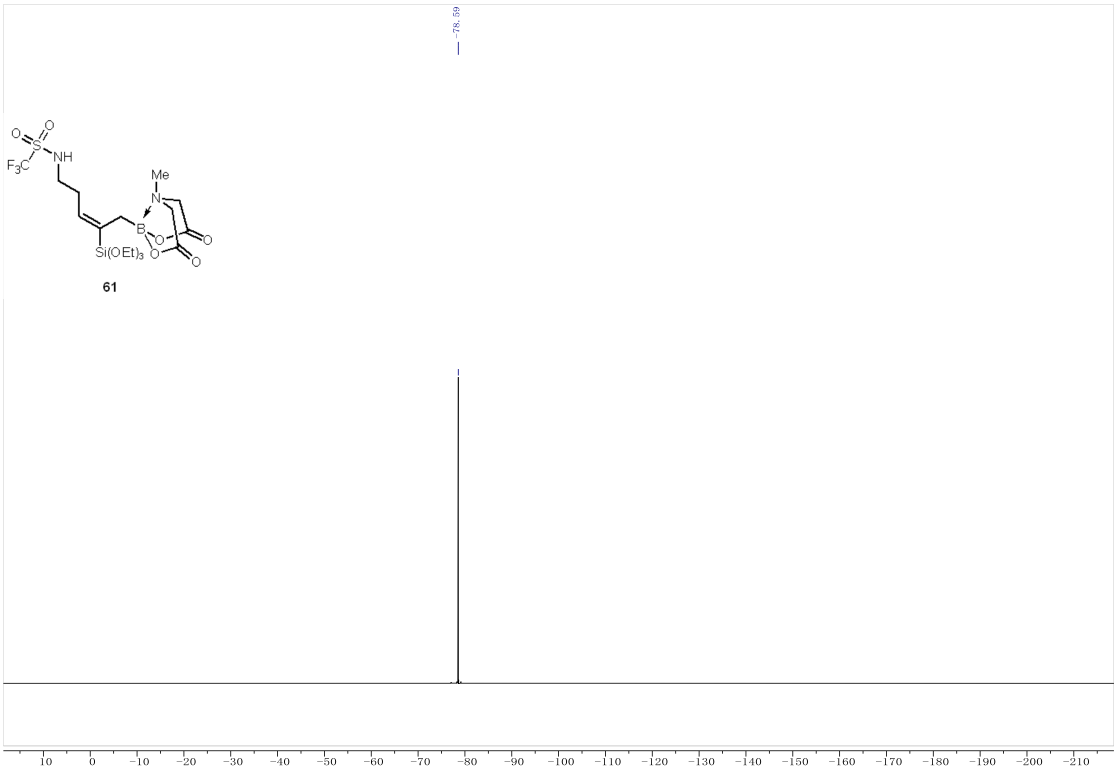


## $^{1}$H NMR of 62 (400 MHz, Acetone-$d_6$)

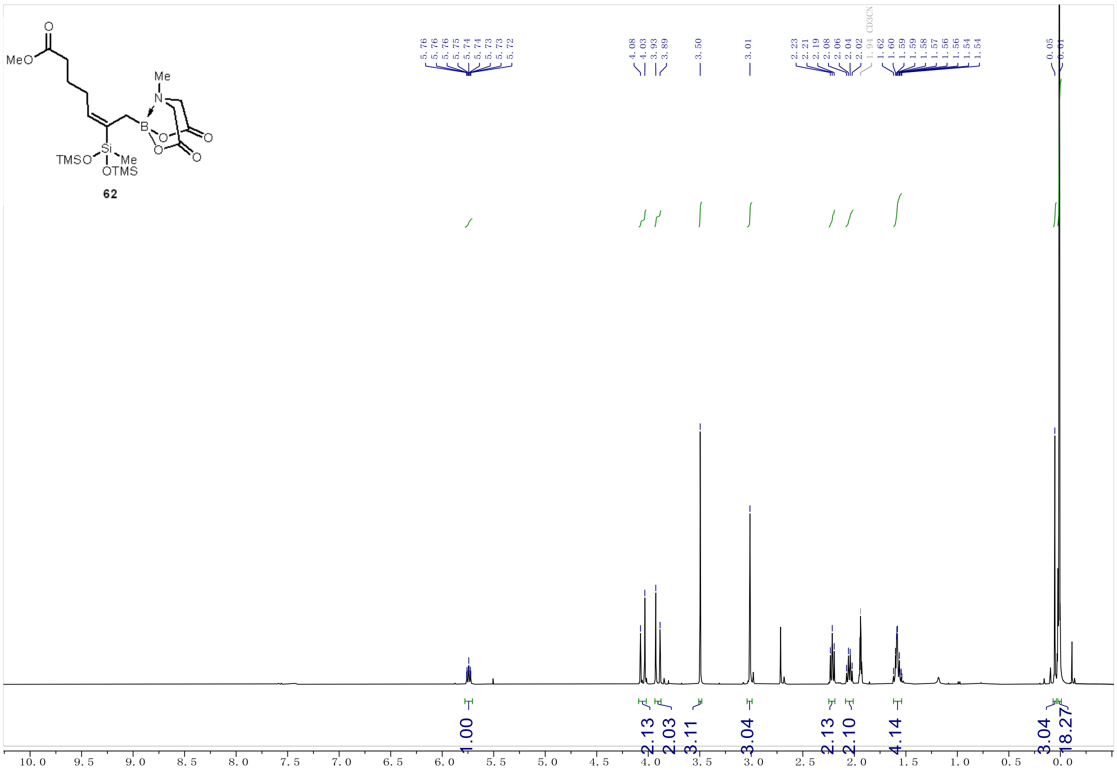

**$^{13}$C NMR of 62 (101 MHz, Acetone-$d_6$)**

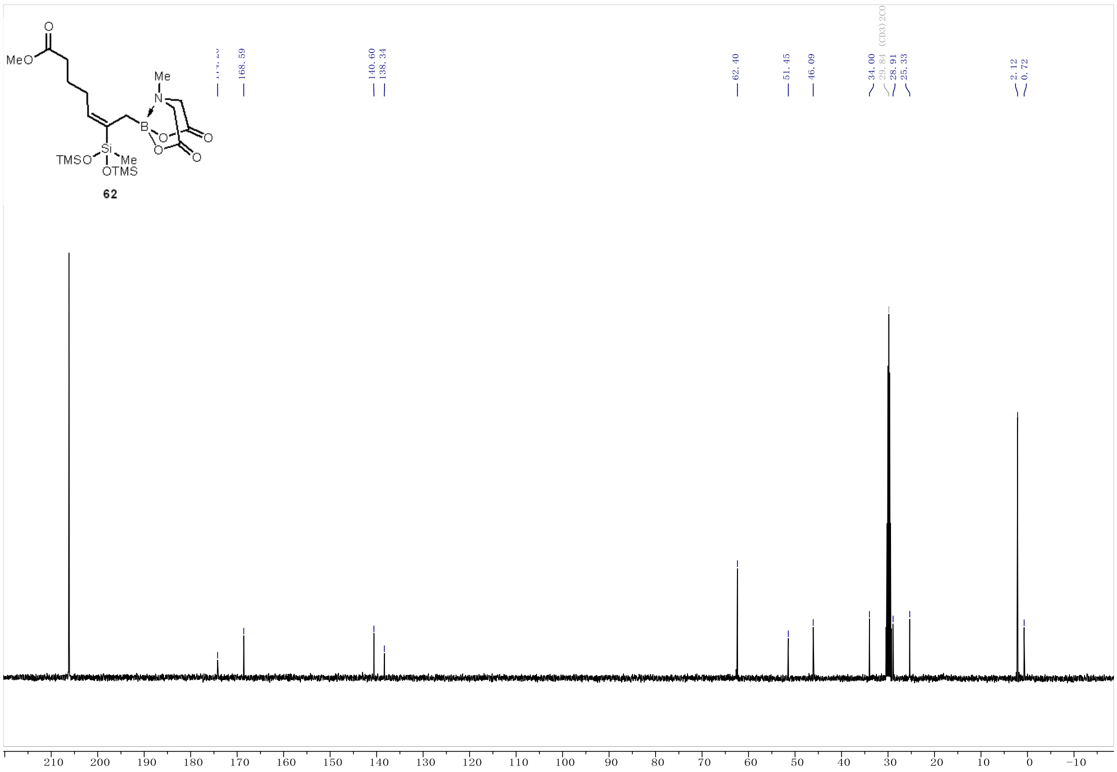


**$^{1}$H NMR of 63 (400 MHz, $CD_3CN$)**

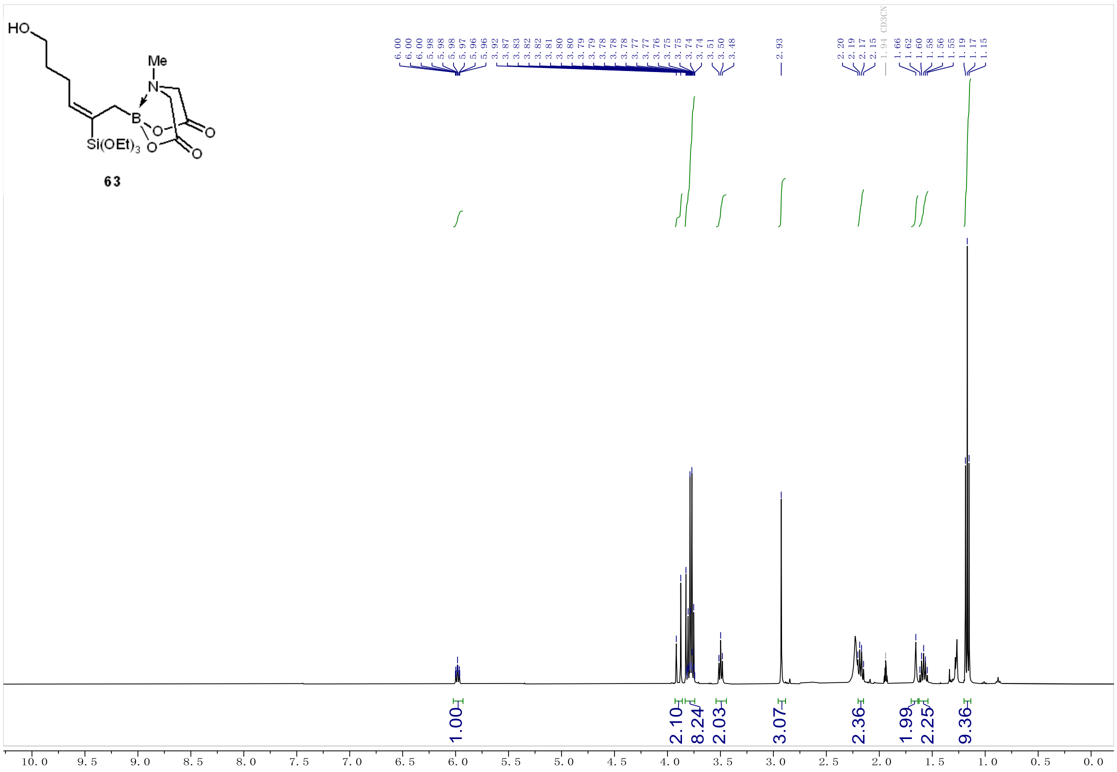

**$^{13}$C NMR of 63 (101 MHz, $CD_3CN$)**

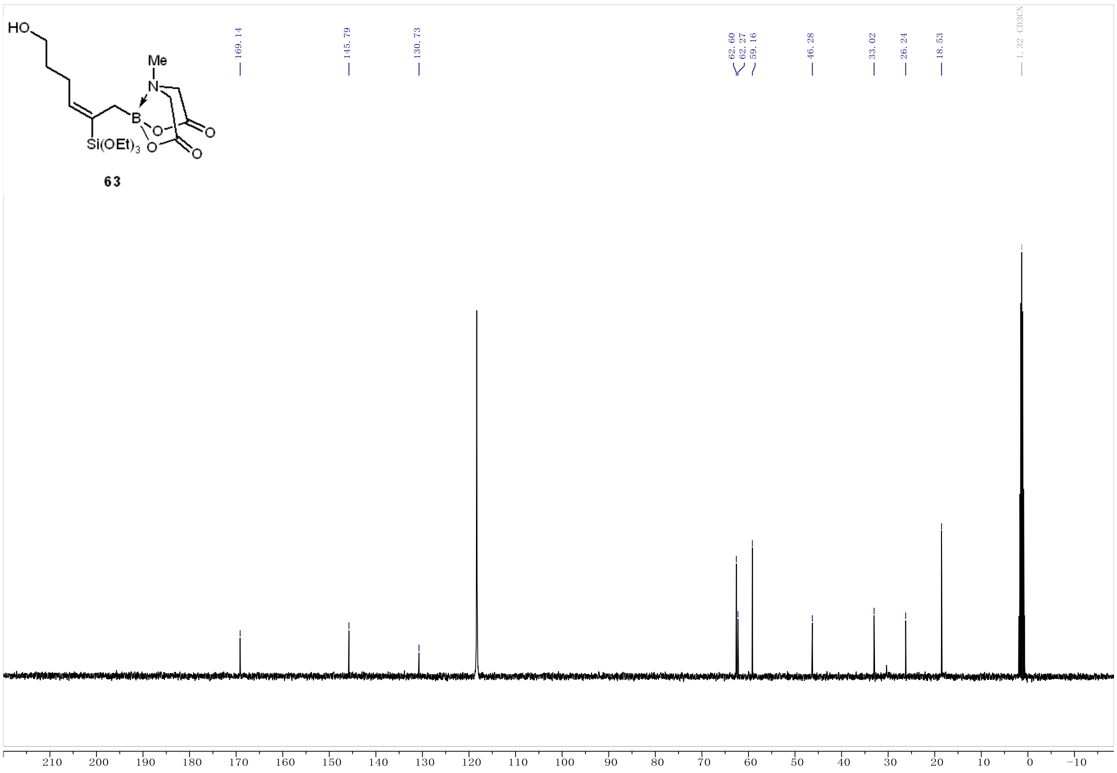

**$^{1}$H NMR of 64 (400 MHz, Acetone-$d_6$)**

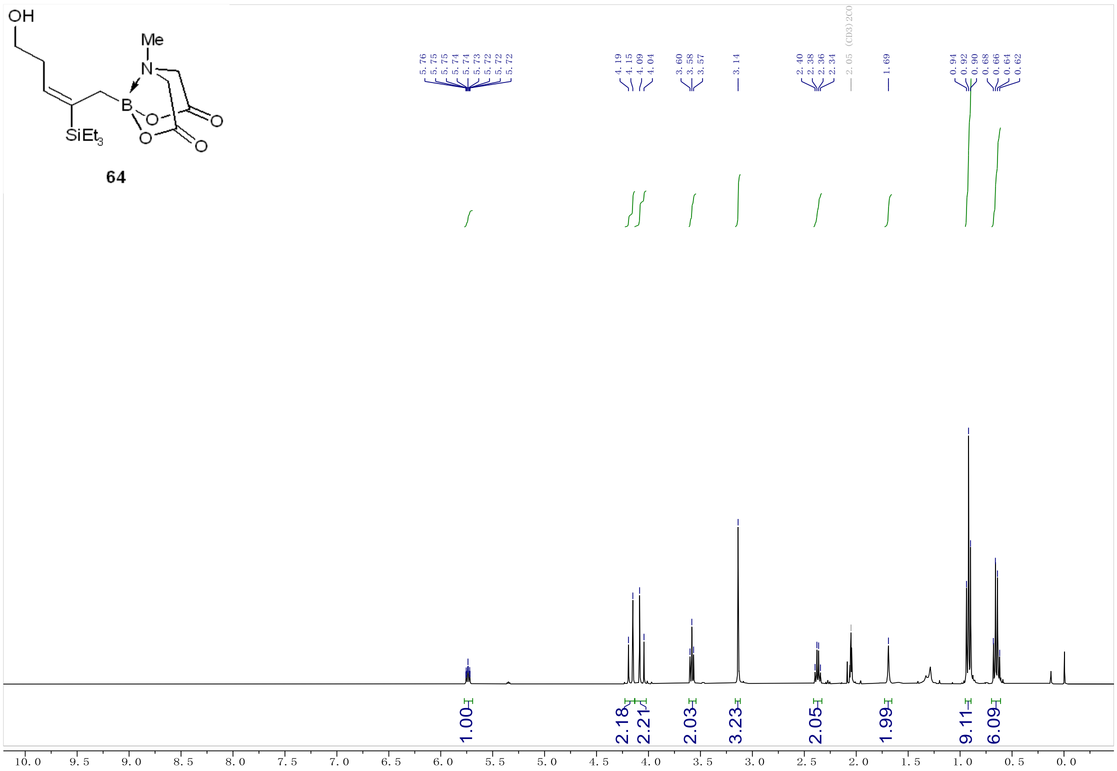


**$^{13}$C NMR of 64 (101 MHz, Acetone-$d_6$)**

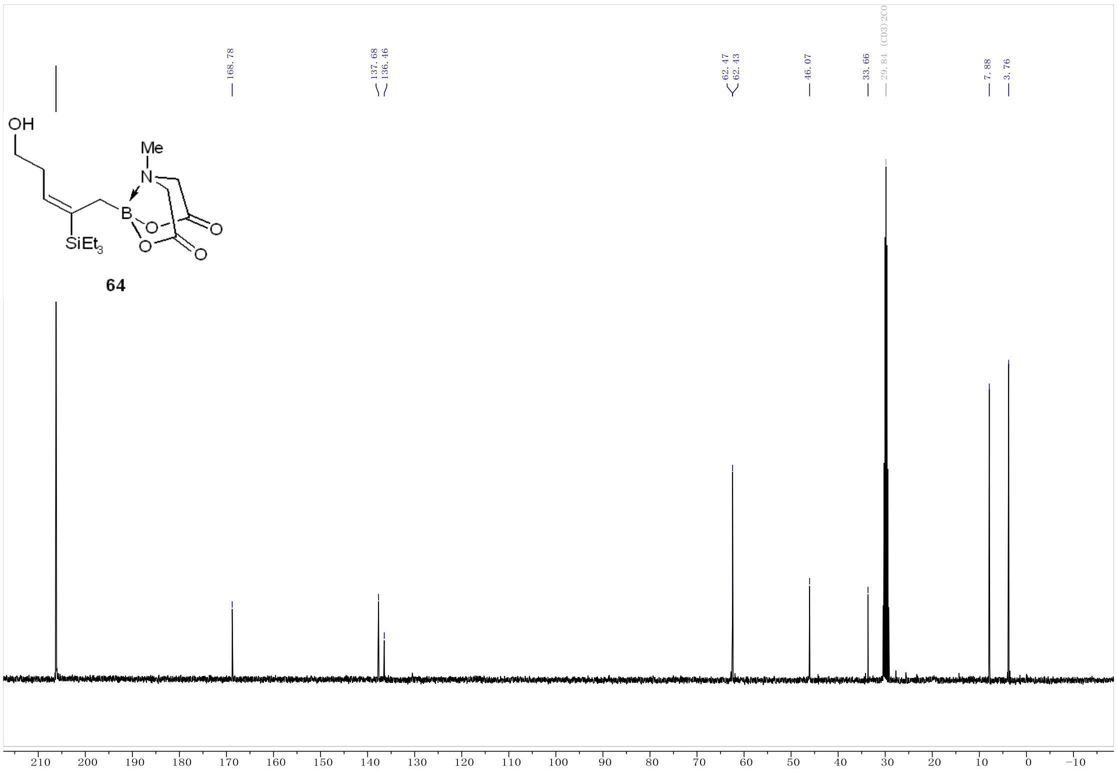


**$^{1}$H NMR of 65 (400 MHz, Acetone-$d_6$)**

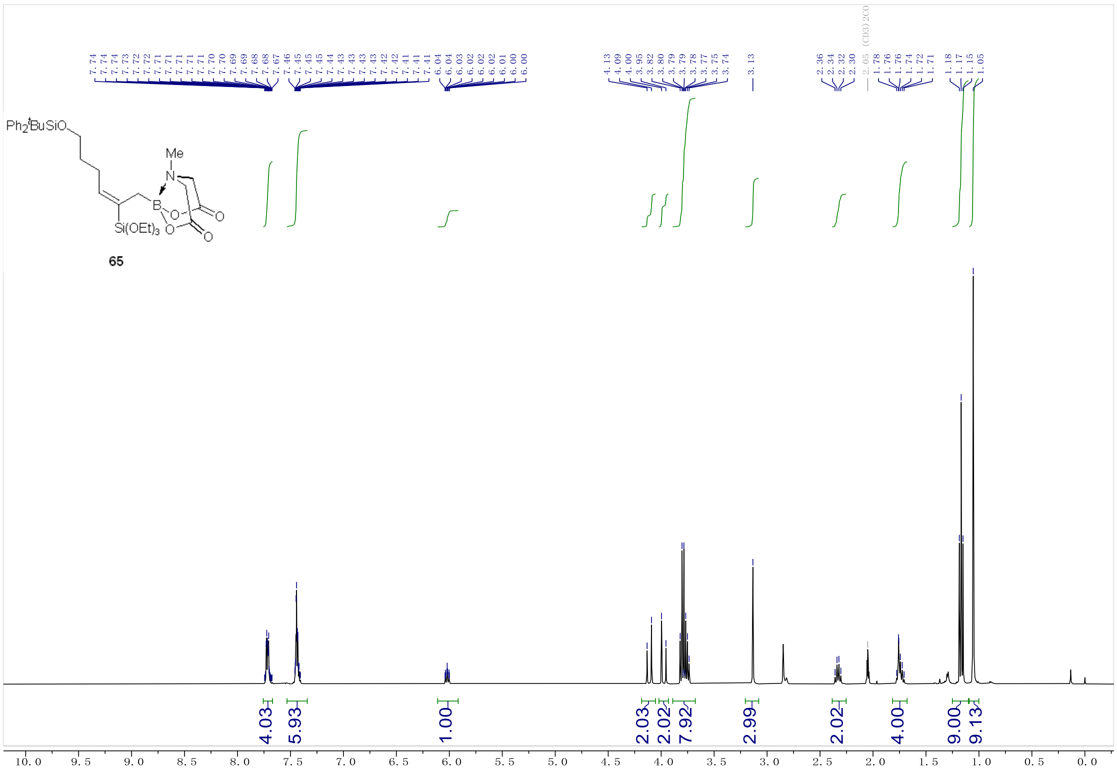


## $^{13}$C NMR of 65 (101 MHz, Acetone-$d_6$)

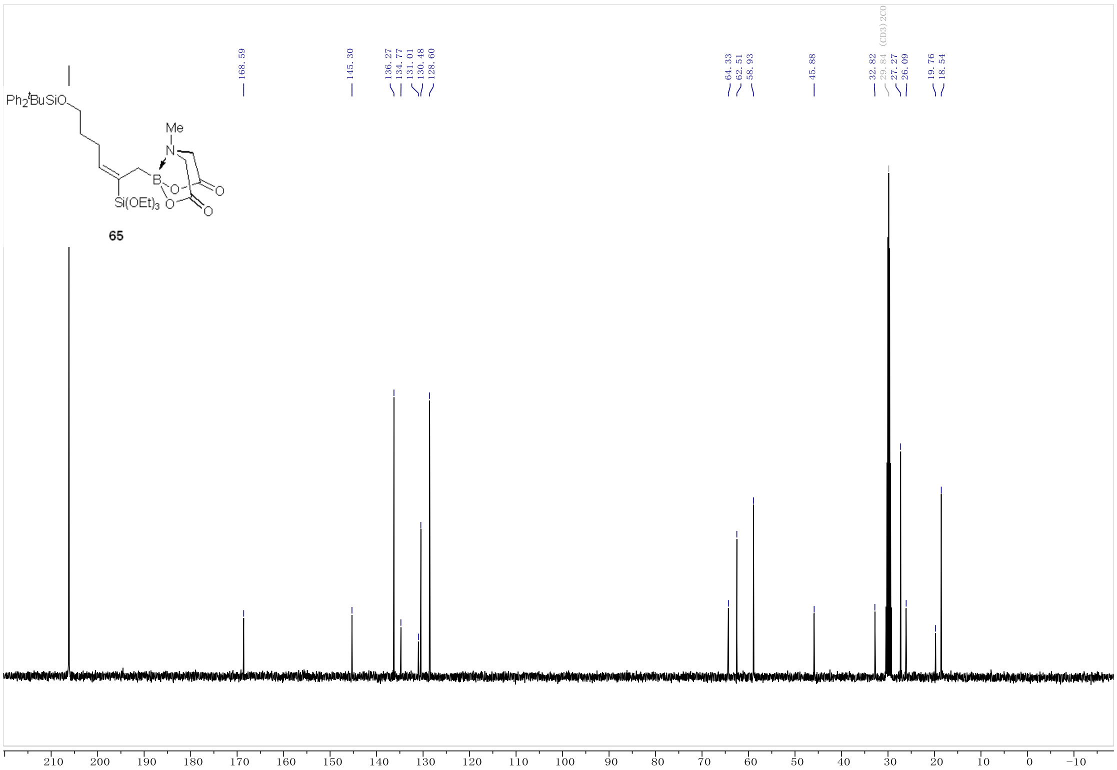

## $^{1}$H NMR of 66 (500 MHz, $CD_3CN$)

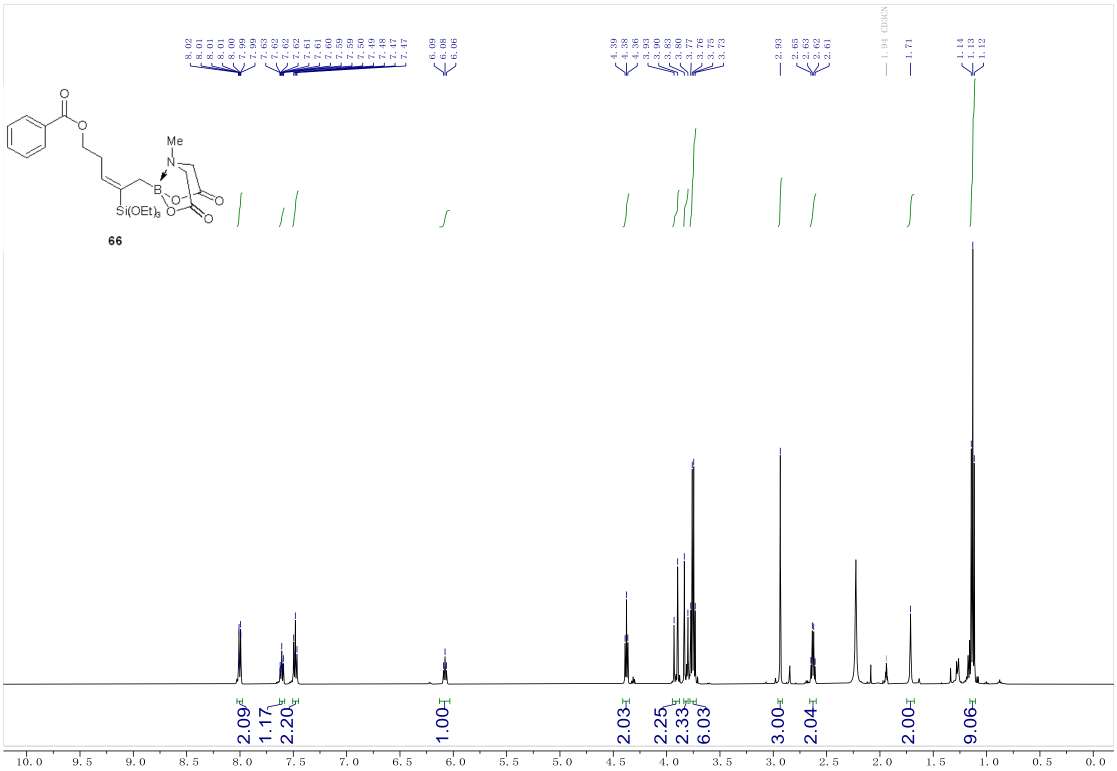


## $^{13}$C NMR of 66 (126 MHz, $CD_3CN$)

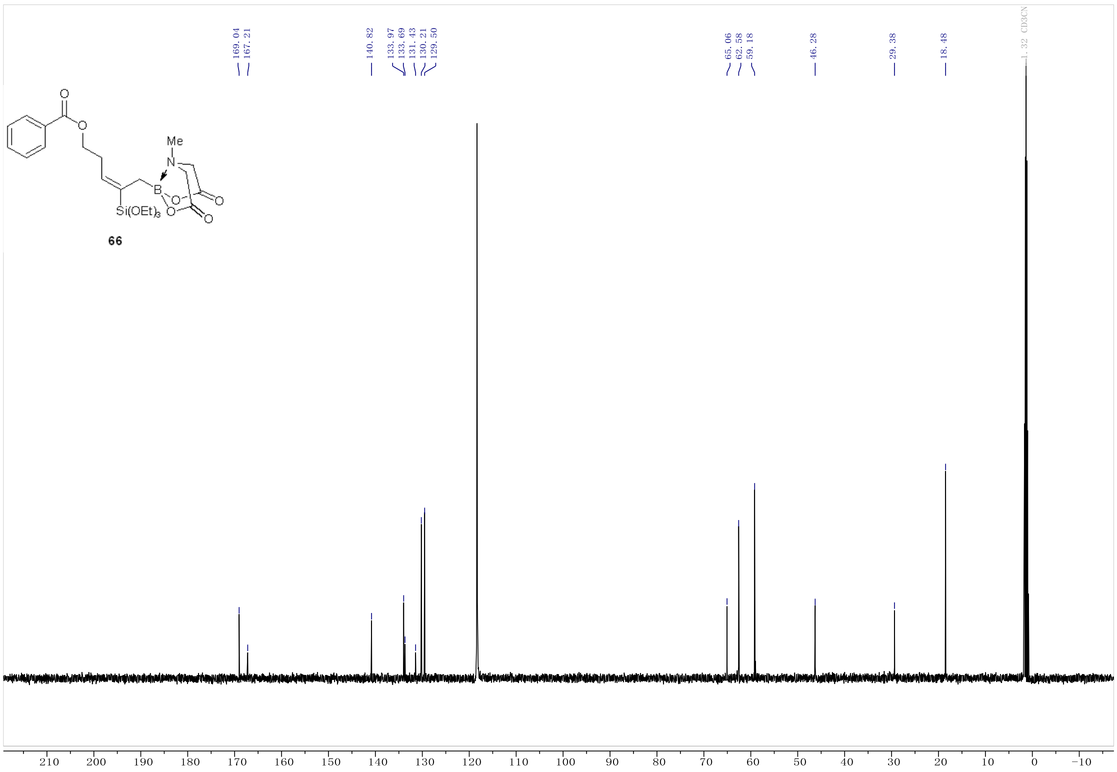

$^{1}$H NMR of 67 (500 MHz, Acetone-$d_6$)

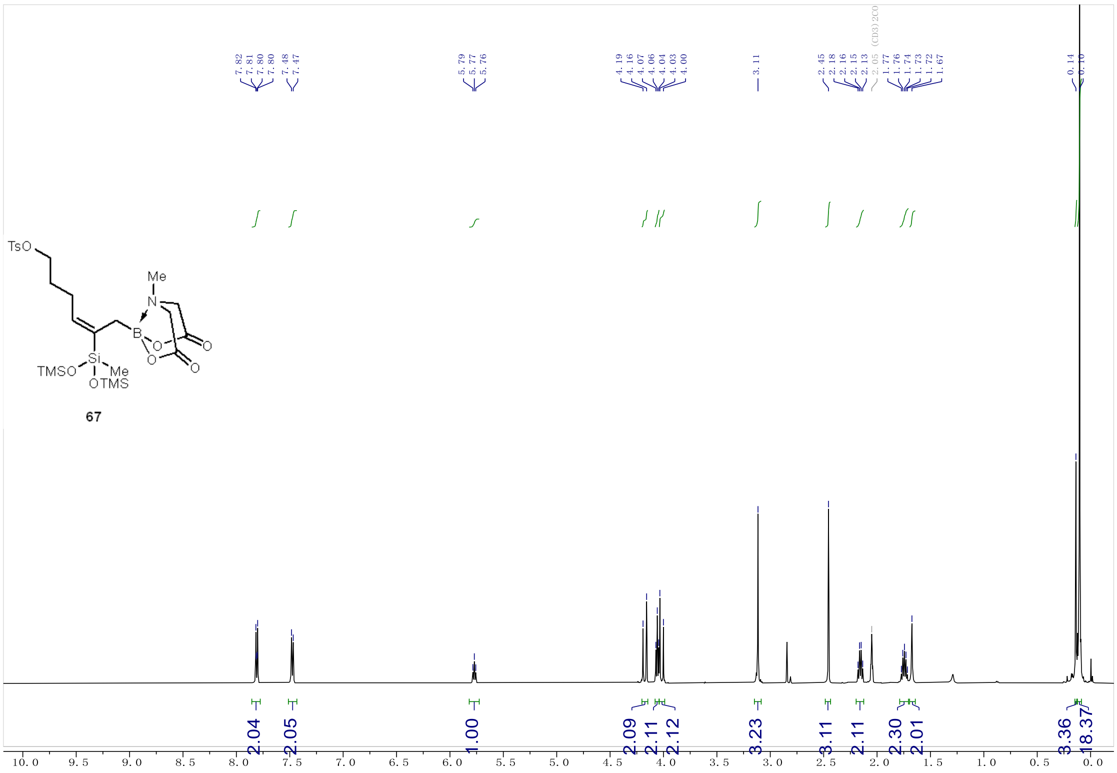

$^{13}$C NMR of 67 (101 MHz, Acetone-$d_6$)

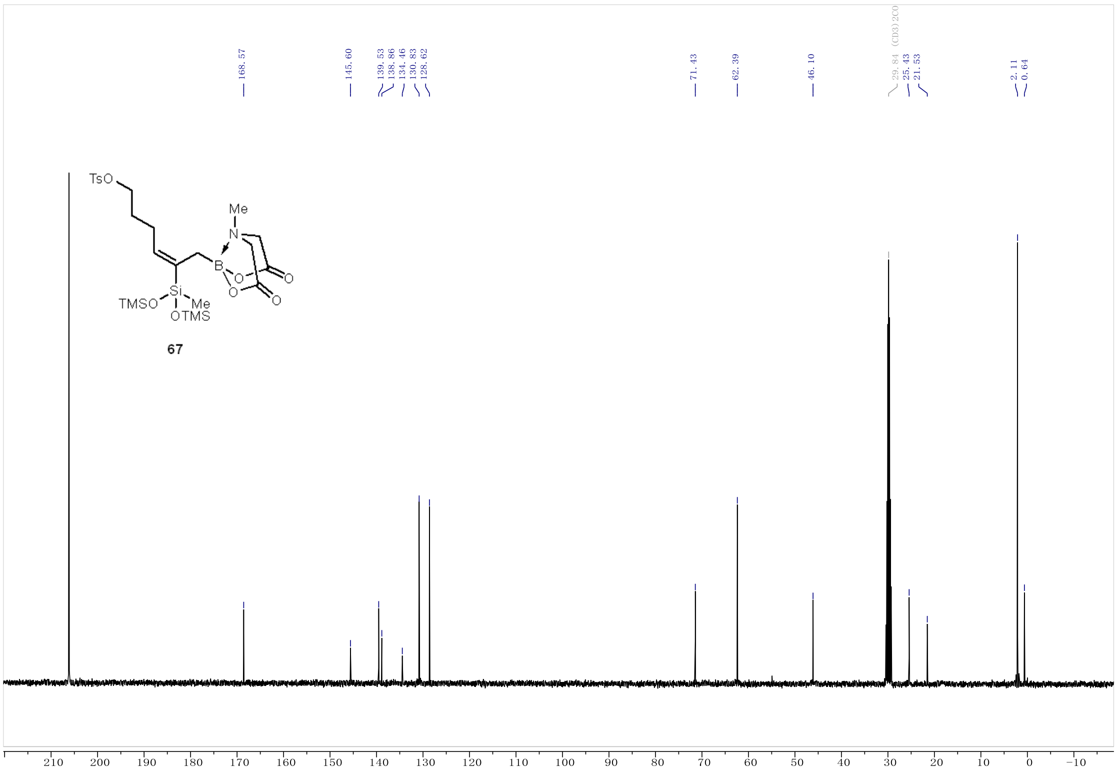

## $^{1}H$ NMR of 68 (400 MHz, $CD_3CN$)

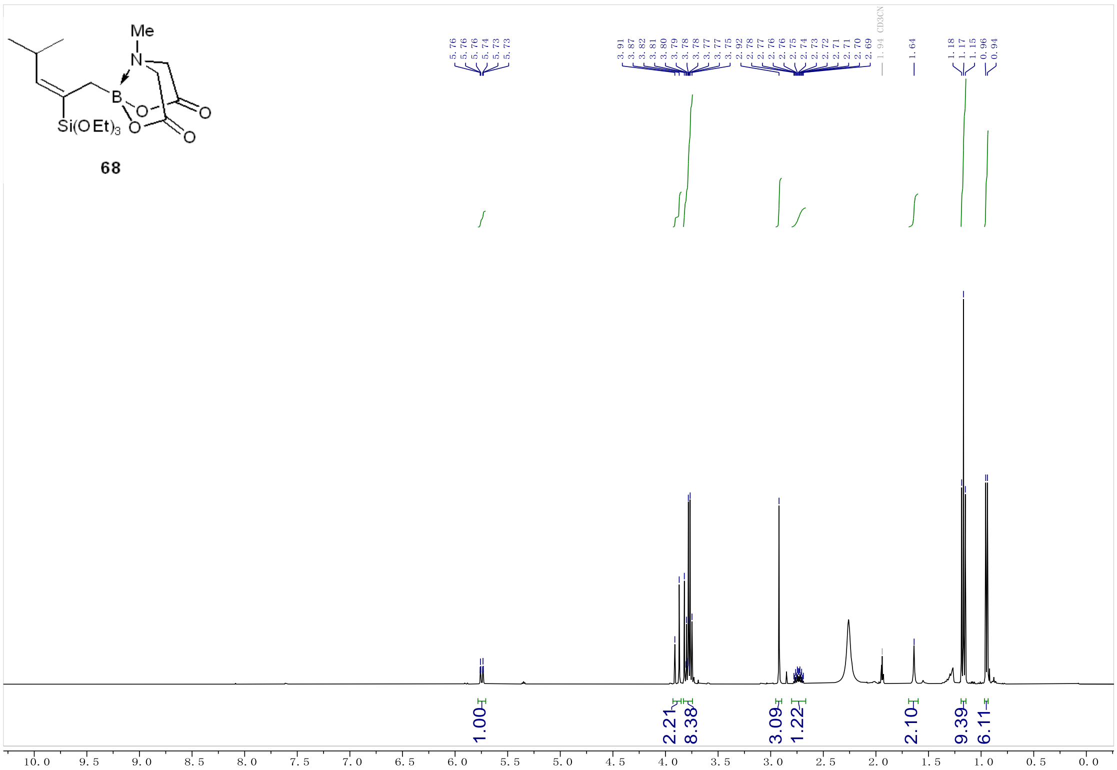

## $^{13}C$ NMR of 68 (101 MHz, Acetone-$d_6$)

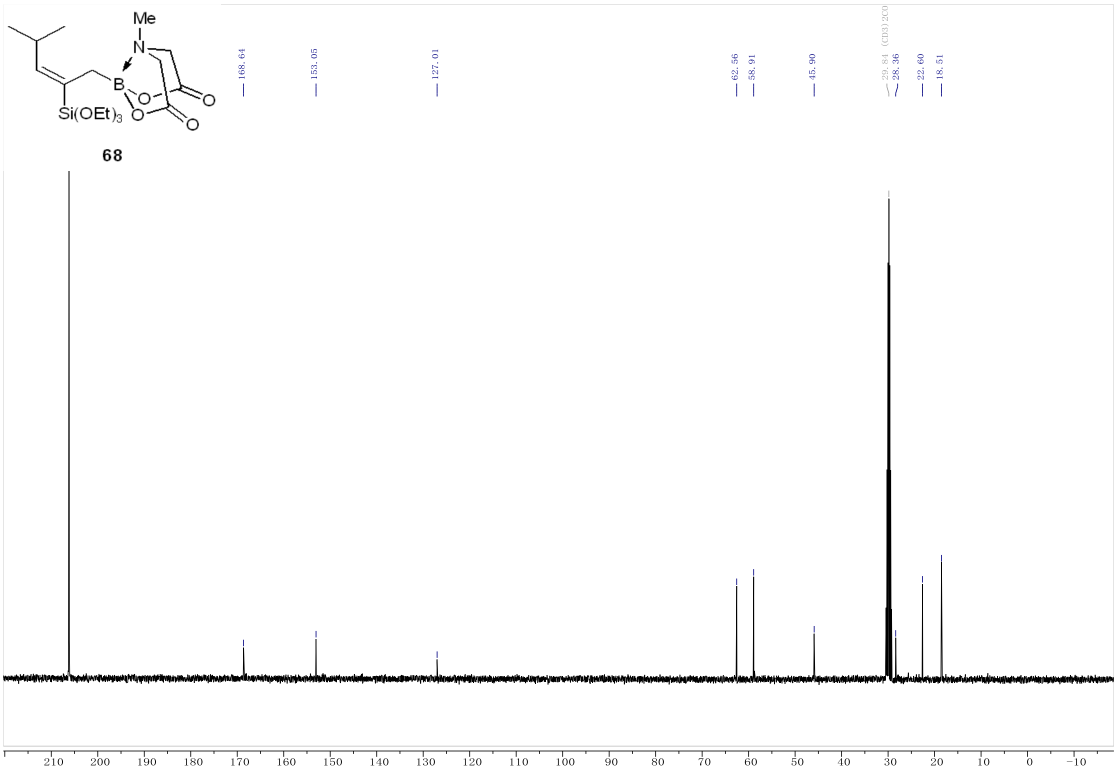

## $^{1}H$ NMR of 69 (400 MHz, Acetone-$d_6$)

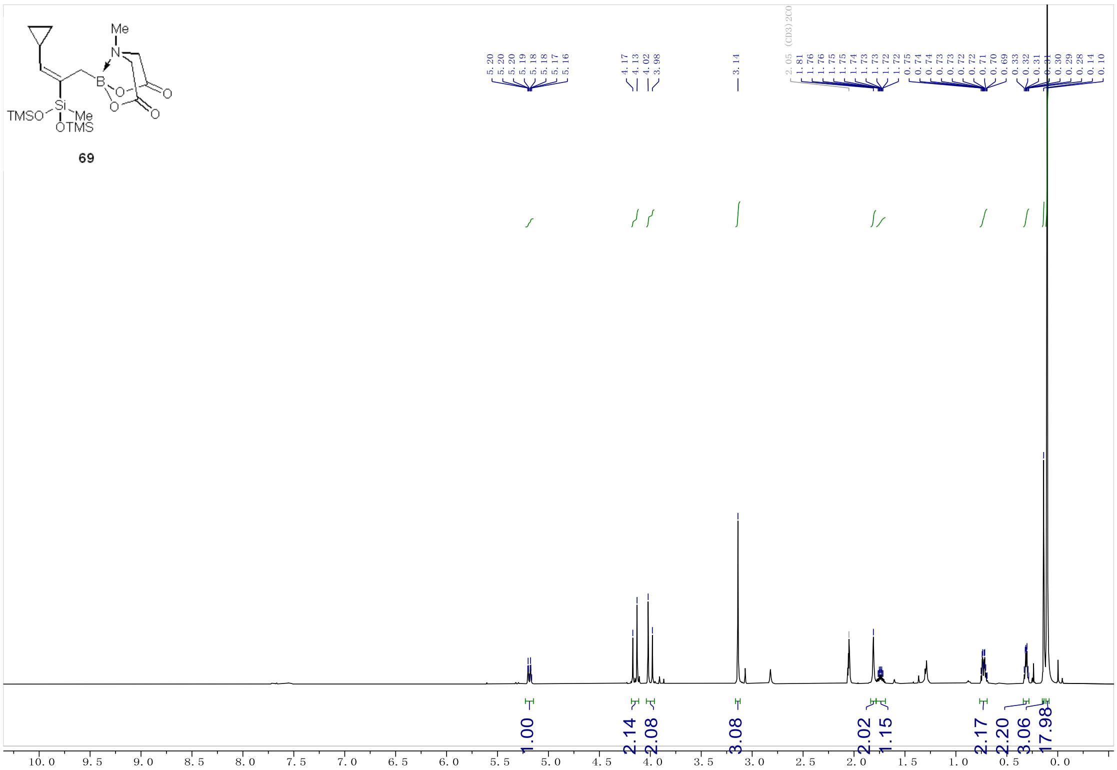


## $^{13}C$ NMR of 69 (101 MHz, Acetone-$d_6$)

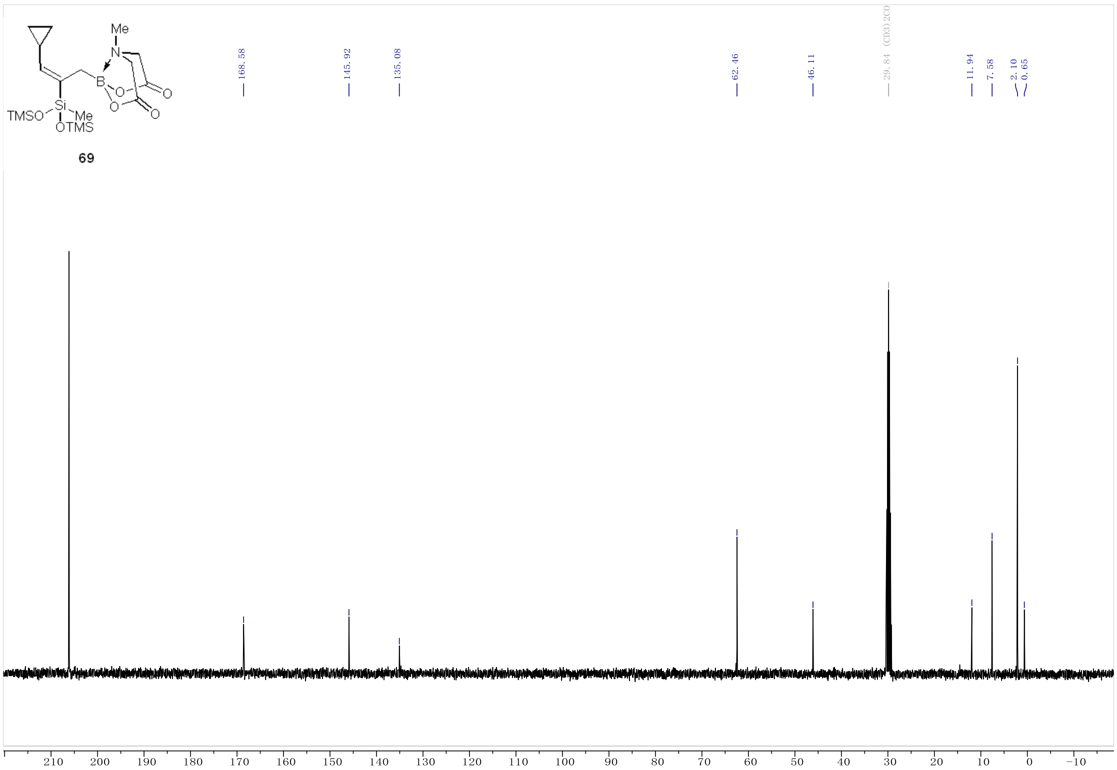

## $^{1}$H NMR of 70 (400 MHz, DMSO-$d_6$)

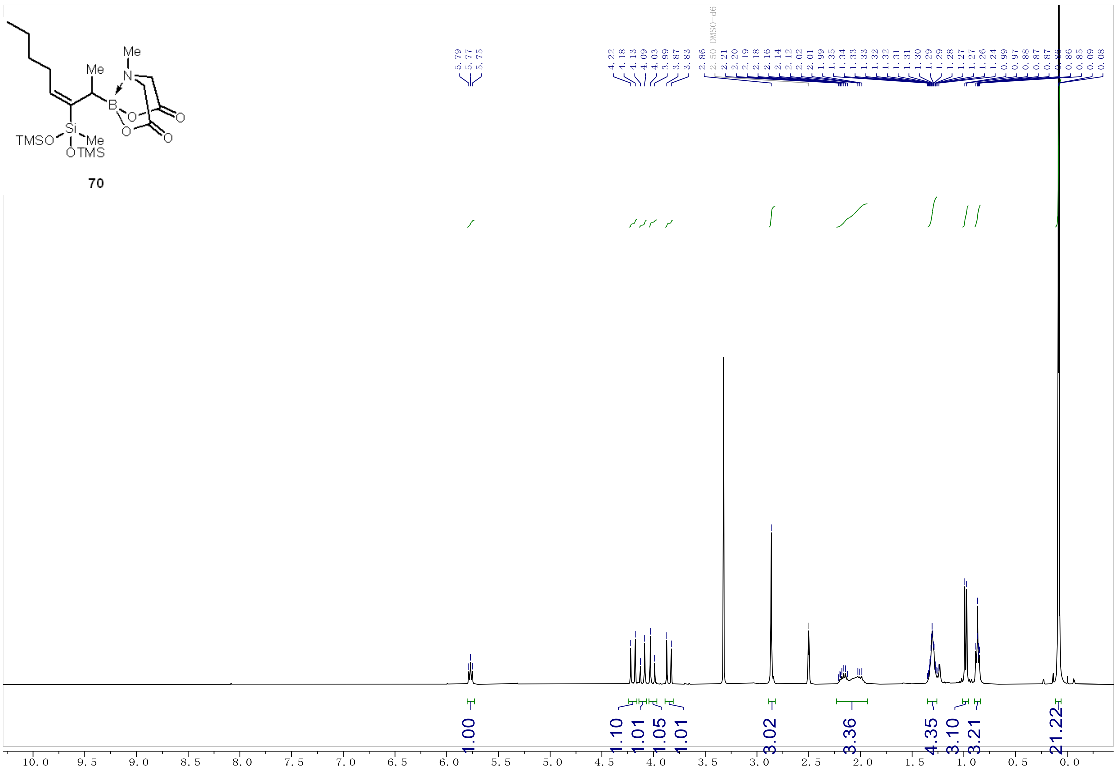

## $^{13}$C NMR of 70 (101 MHz, DMSO-$d_6$)

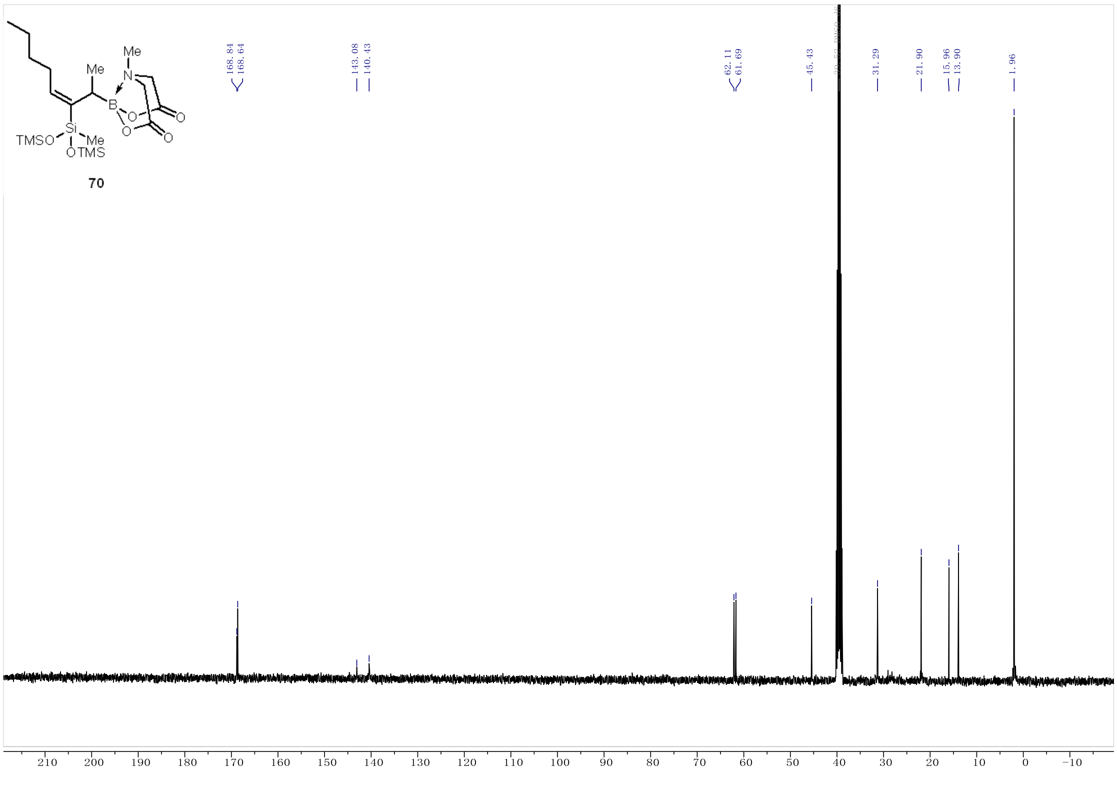

### $^{11}$B NMR of 70 (128 MHz, Acetone-$d_6$)

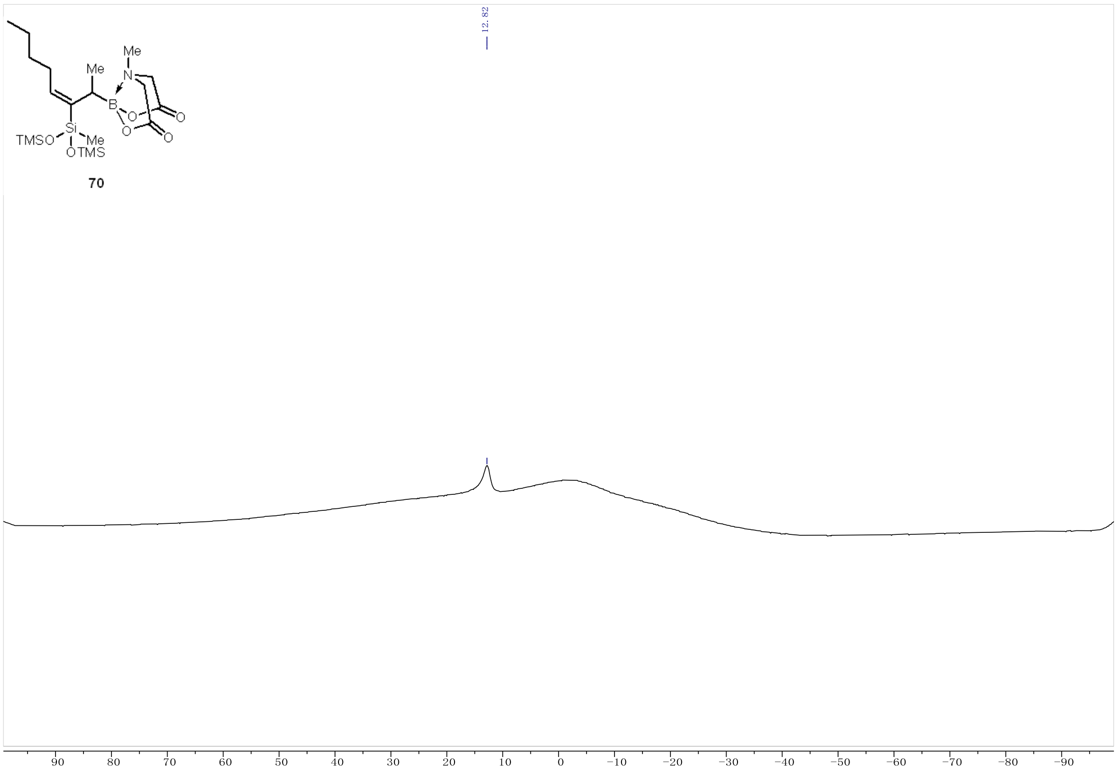


### $^{1}$H NMR of 73 (400 MHz, DMSO-$d_6$)

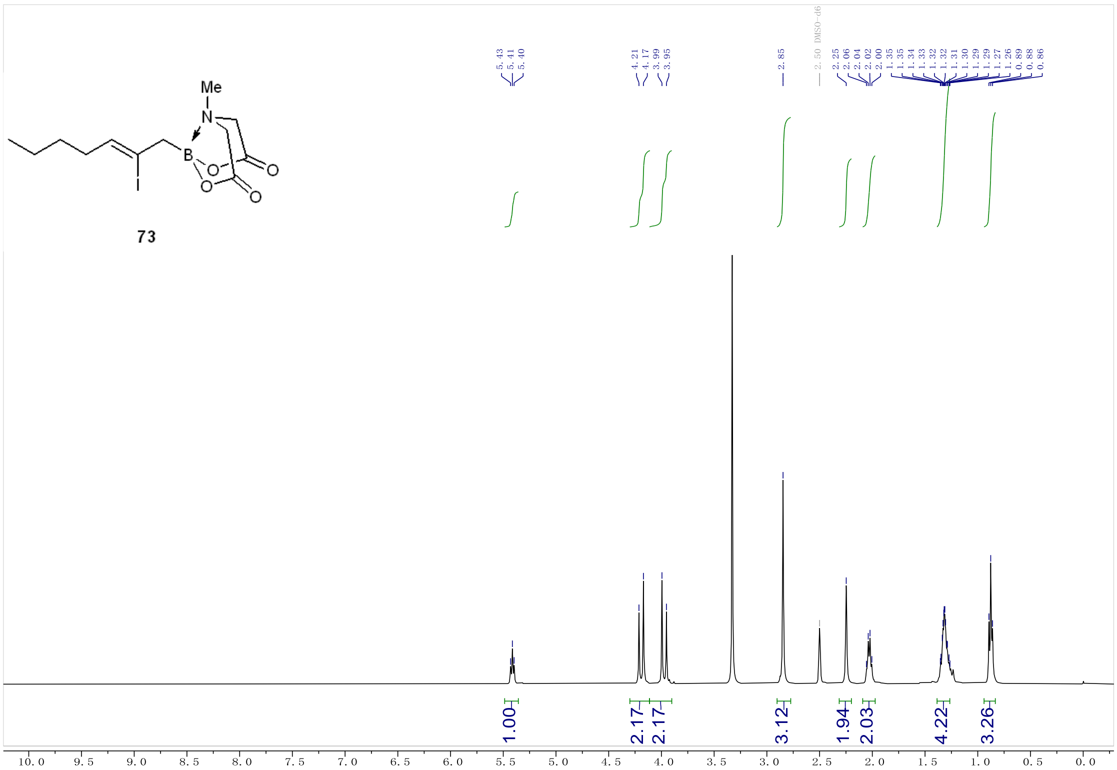

## $^{13}$C NMR of 73 (101 MHz, DMSO-$d_6$)

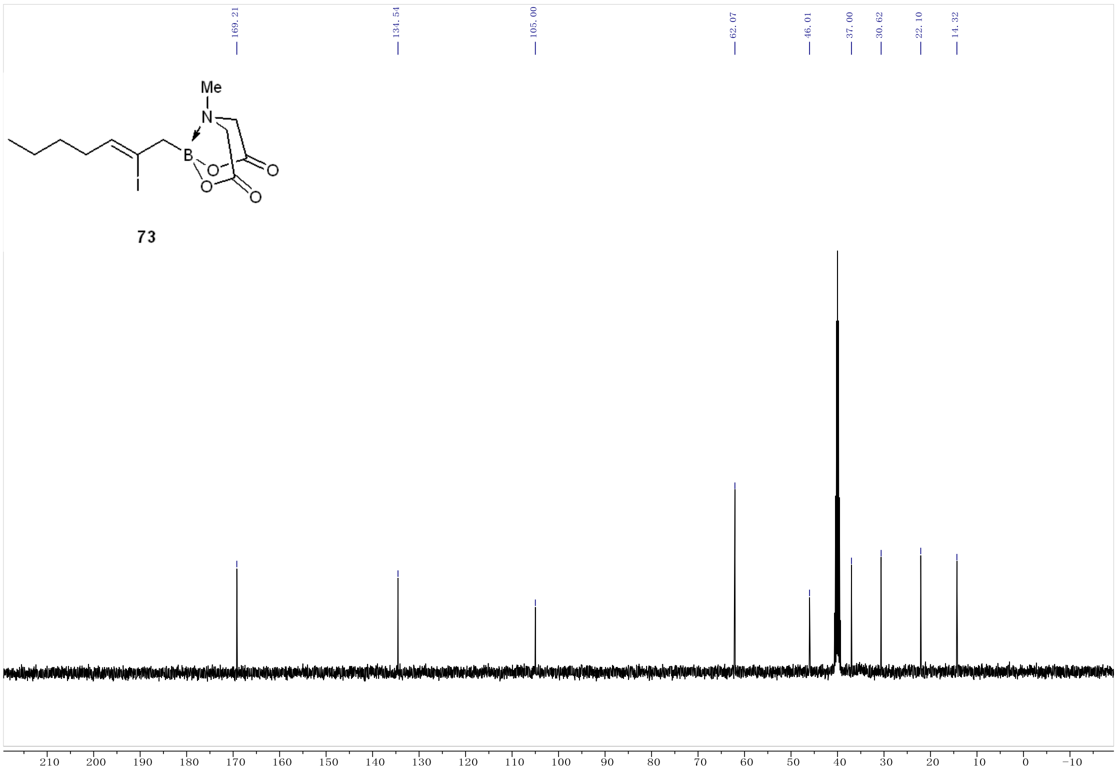


## $^{11}$B NMR of 73 (128 MHz, Acetone-$d_6$)

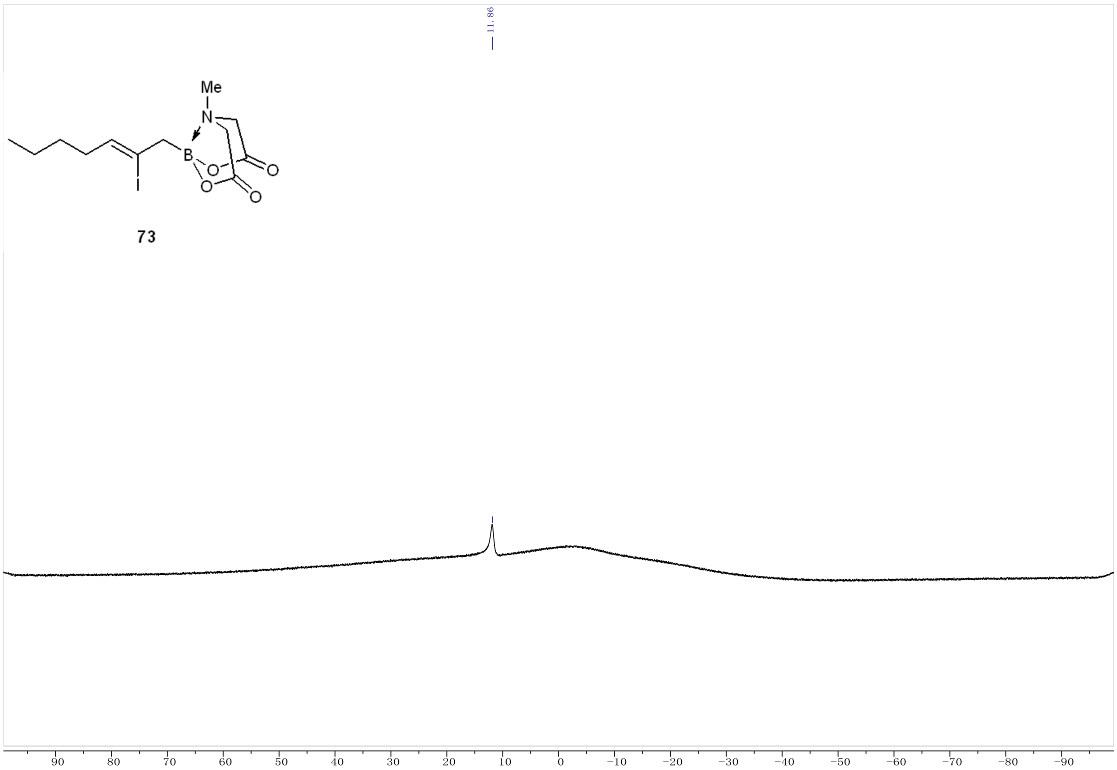

## $^{1}H$ NMR of 74 (400 MHz, DMSO-$d_6$)

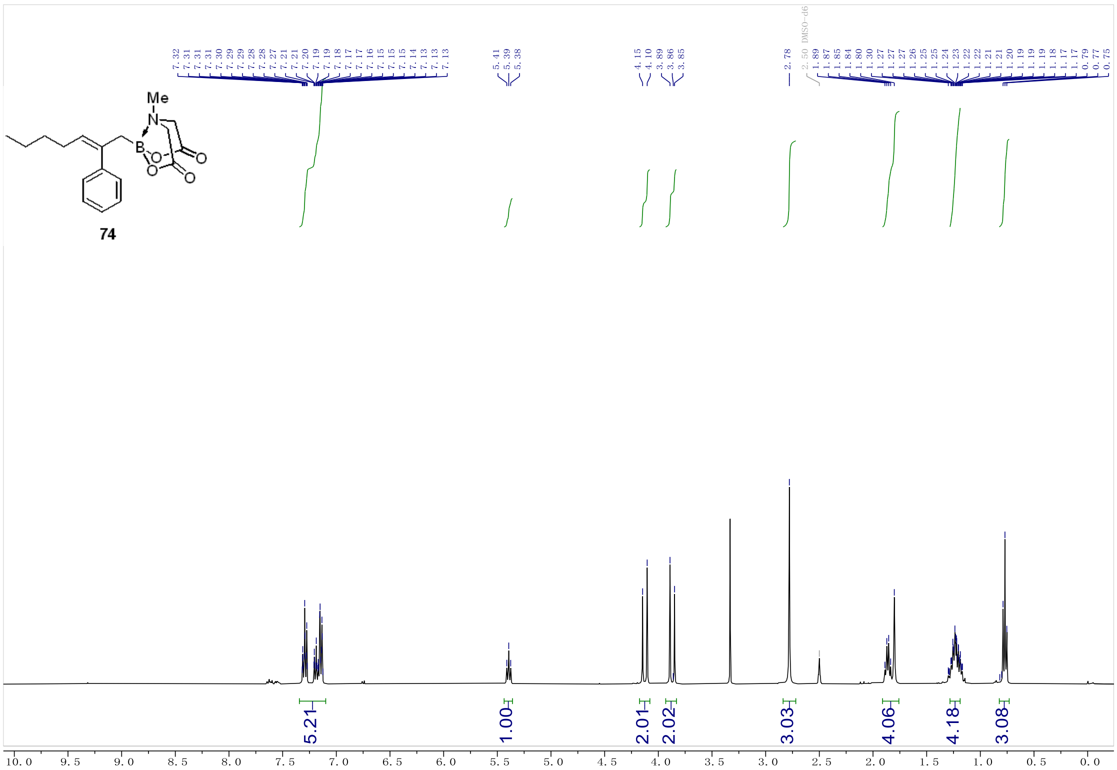


## $^{13}C$ NMR of 74 (101 MHz, DMSO-$d_6$)

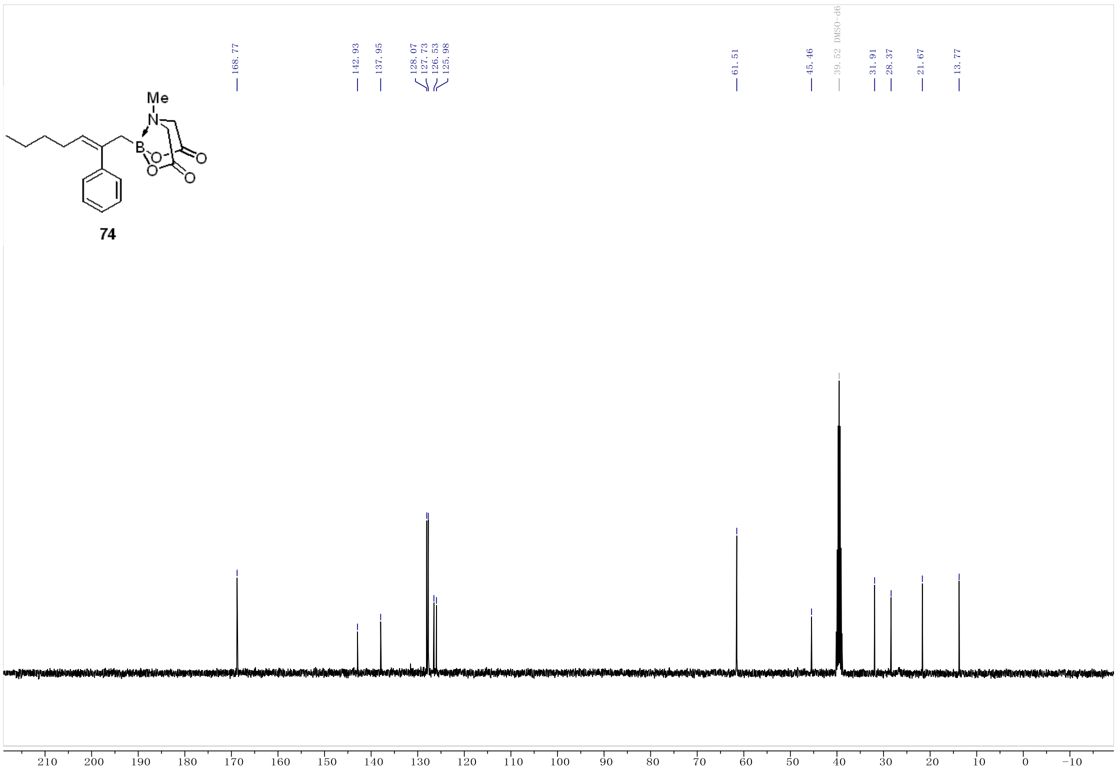

**$^1$H NMR of 75 (400 MHz, Acetone-$d_6$)**

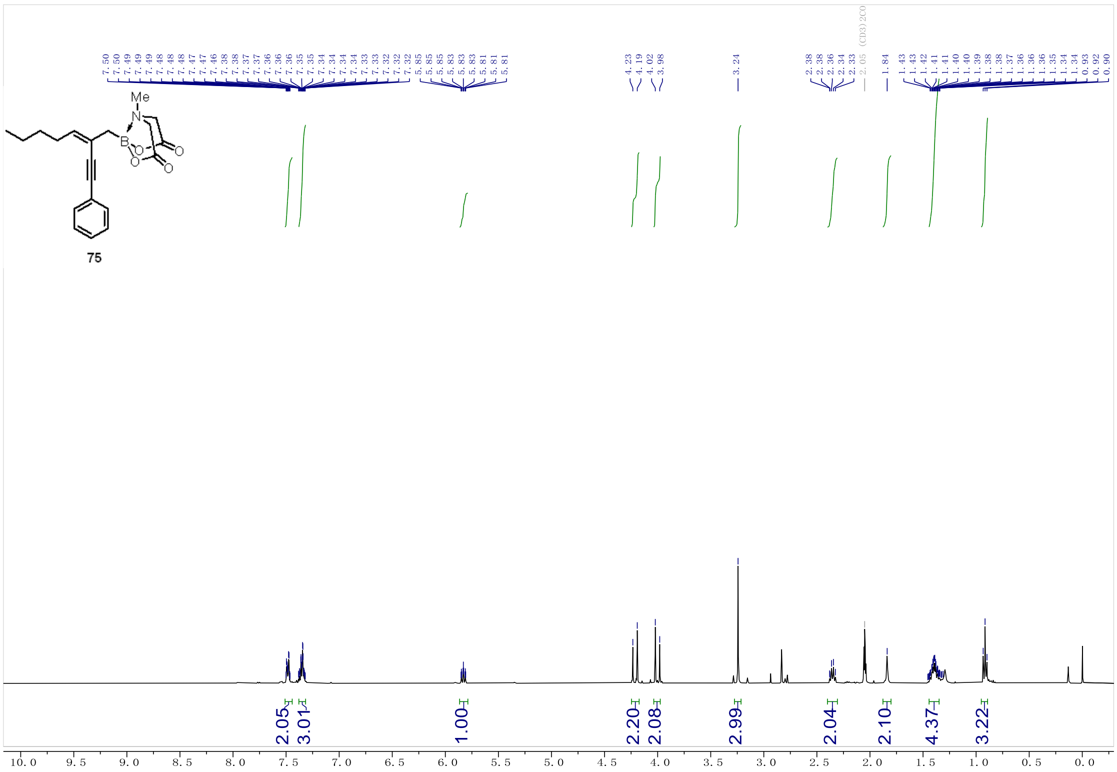

**$^{13}$C NMR of 75 (101 MHz, Acetone-$d_6$)**

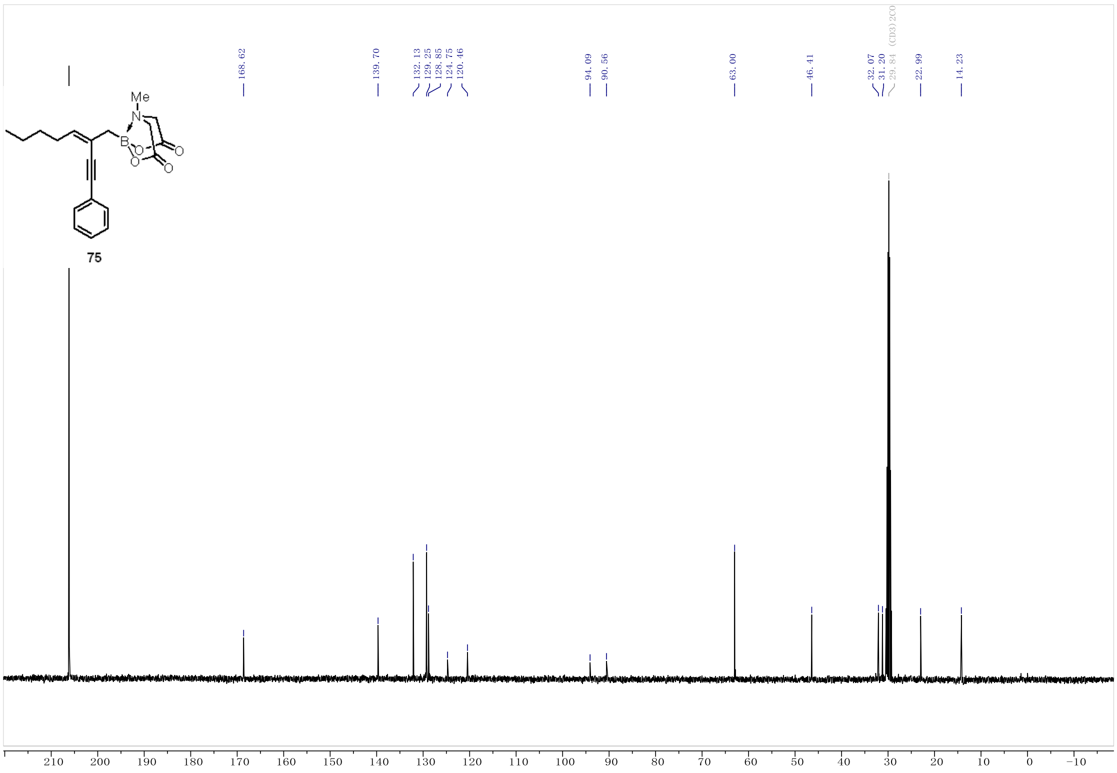

## $^{1}$H NMR of 76 (500 MHz, Acetone-$d_6$)

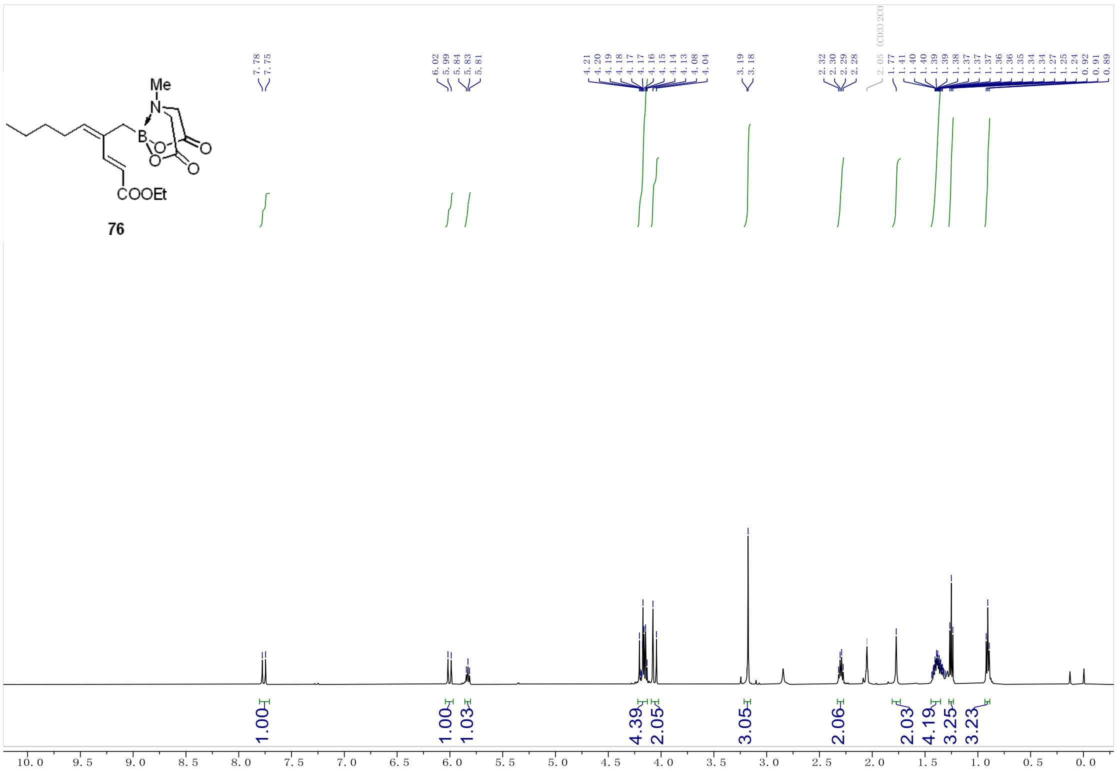


## $^{13}$C NMR of 76 (126 MHz, Acetone-$d_6$)

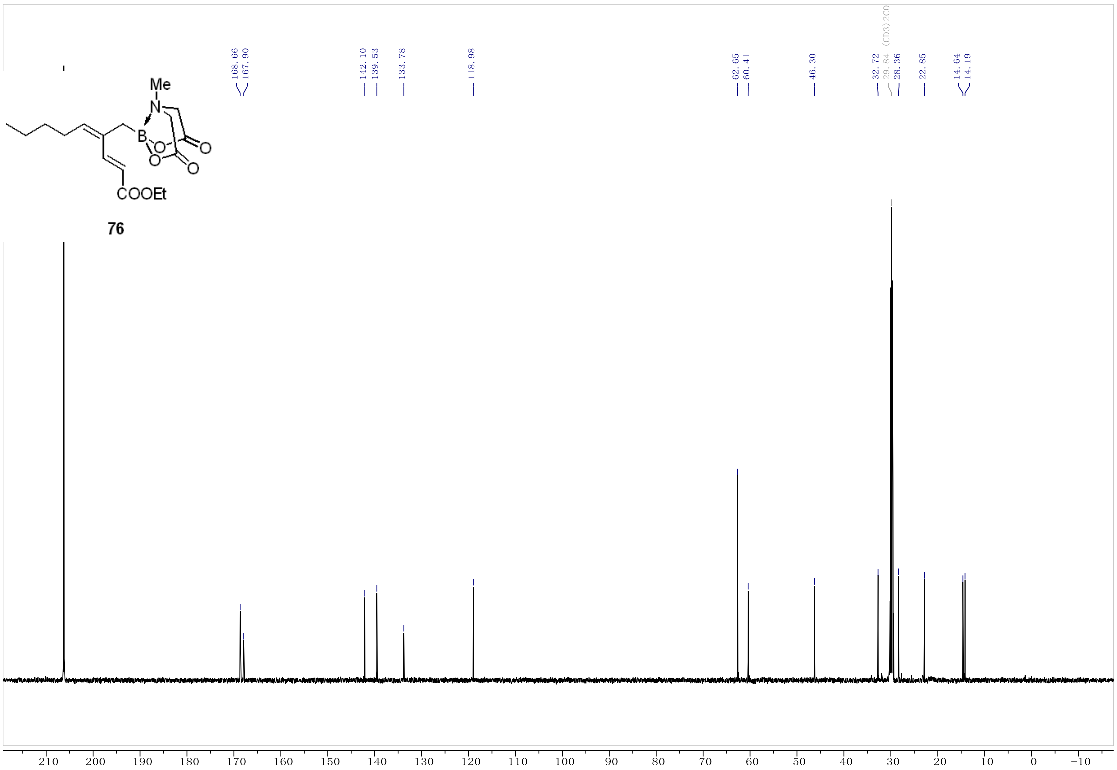

$^{1}H$ NMR of 77 (400 MHz, $CDCl_3$)

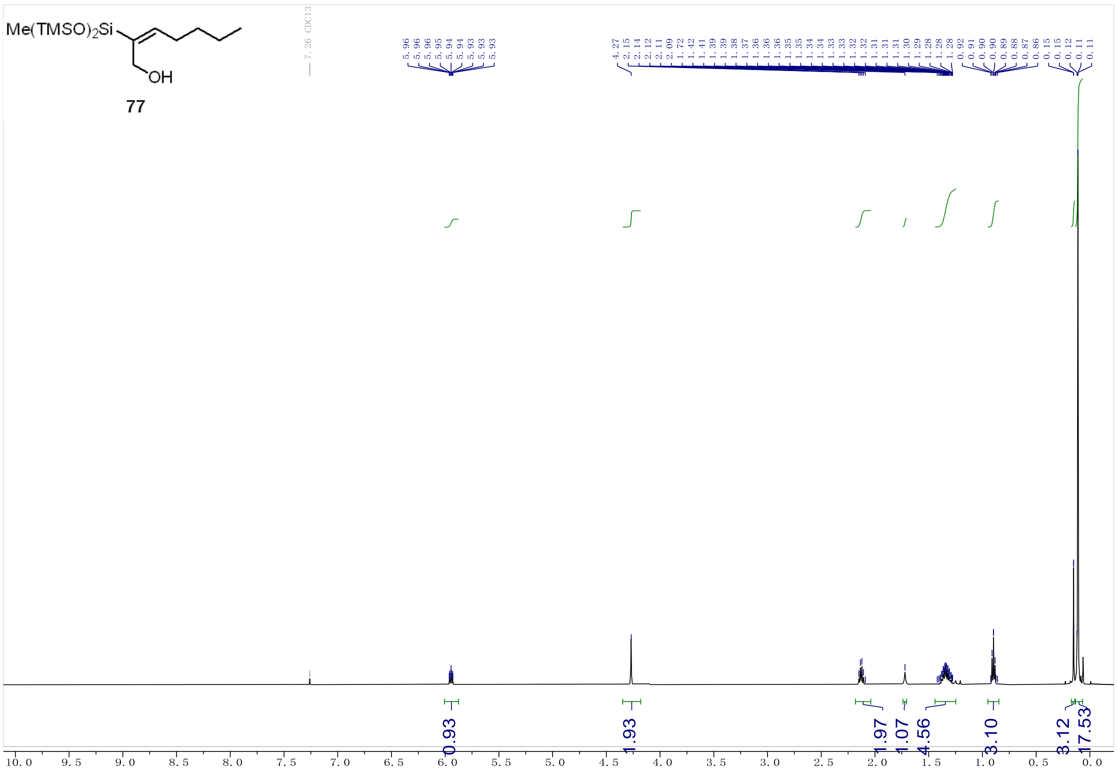


$^{13}C$ NMR of 77 (101 MHz, $CDCl_3$)

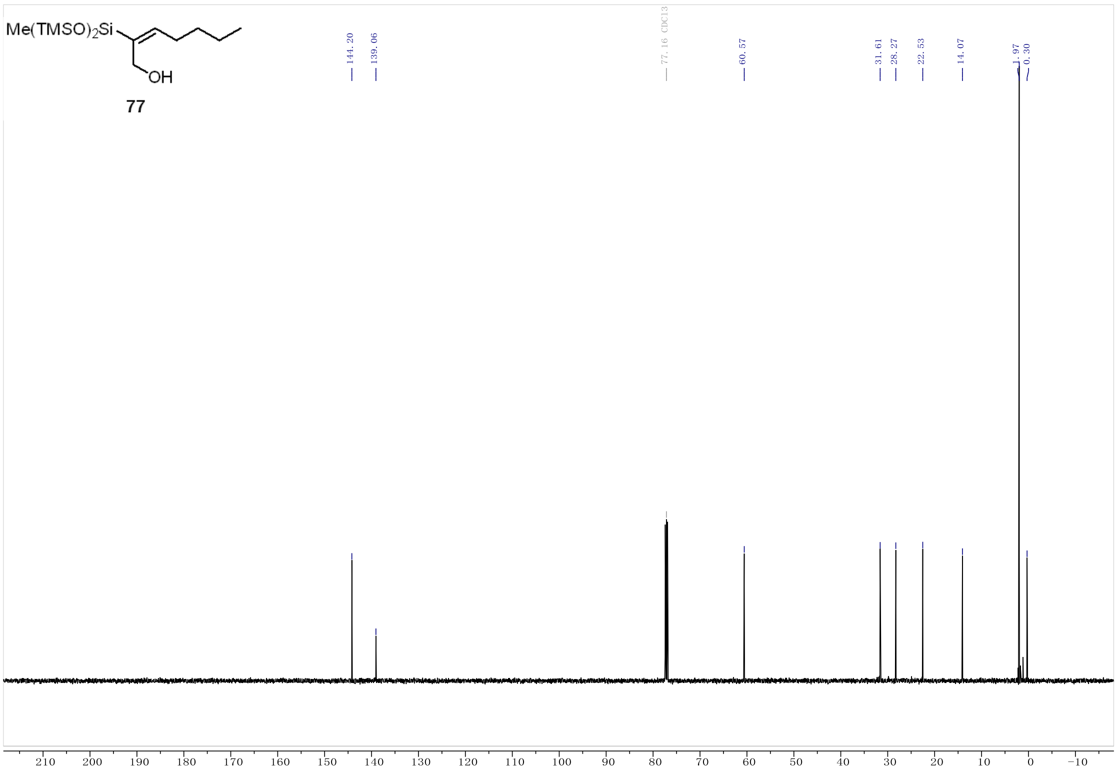

## $^1$H NMR of 78 (400 MHz, $CDCl_3$)

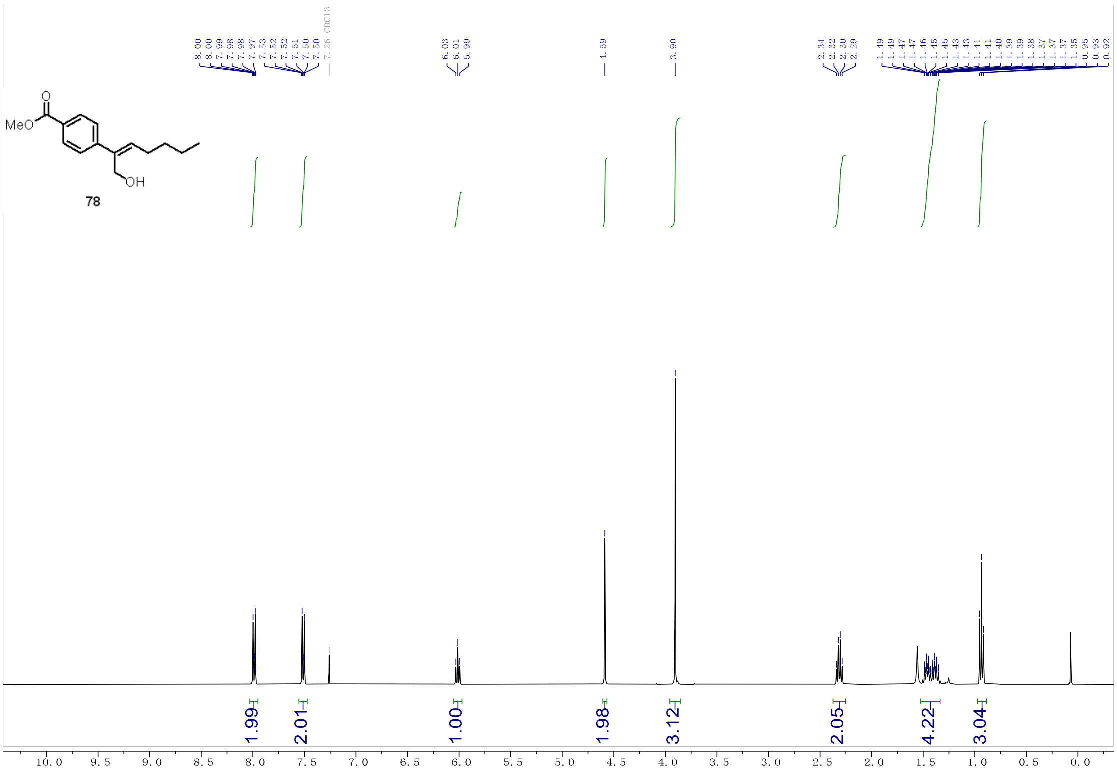


## $^{13}$C NMR of 78 (101 MHz, $CDCl_3$)

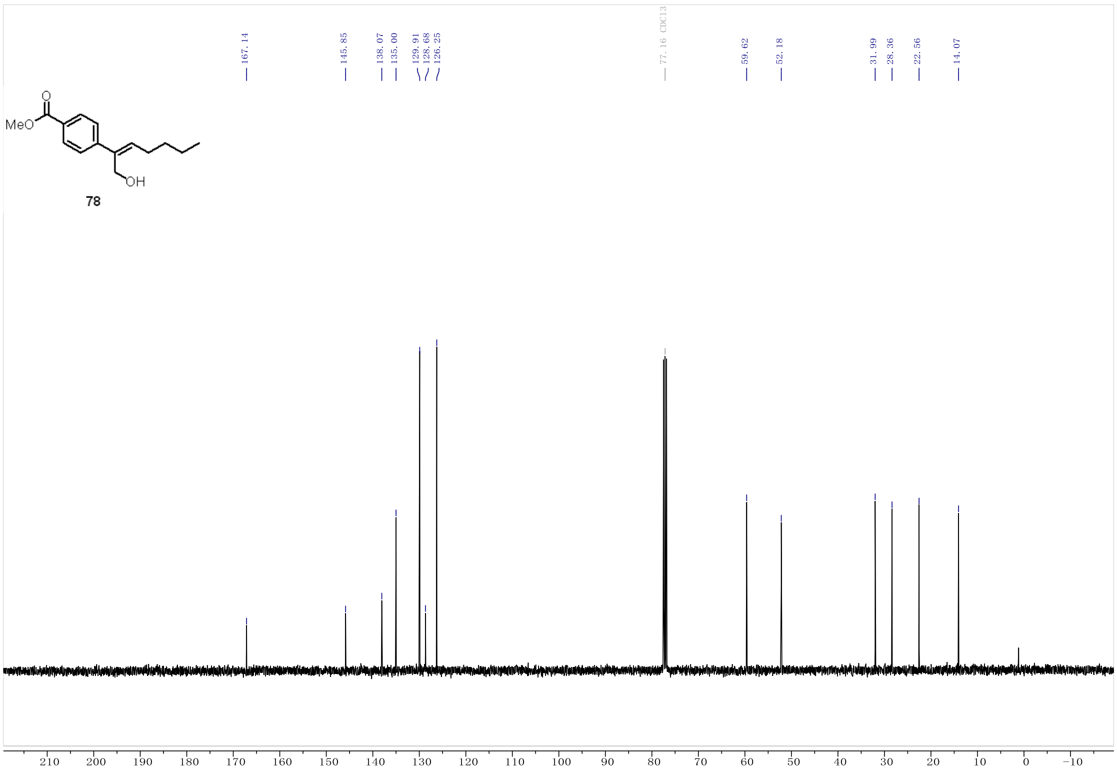

## $^{1}$H NMR of 79 (400 MHz, $CDCl_3$)

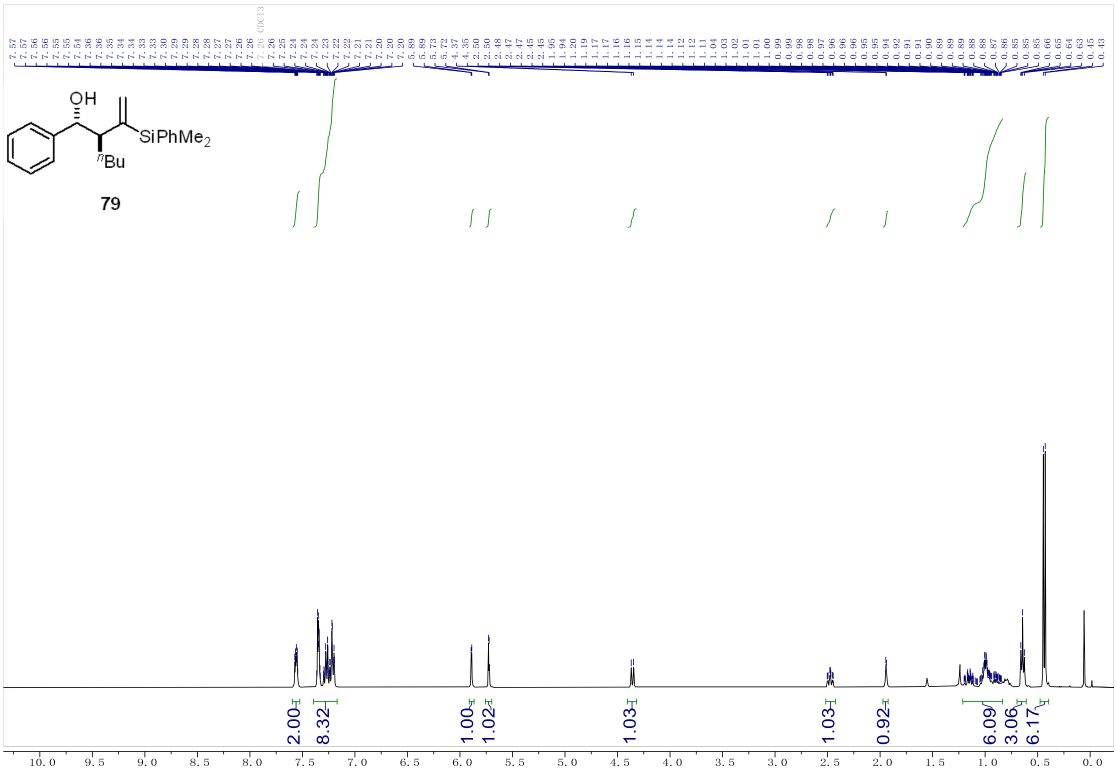

## $^{13}$C NMR of 79 (101 MHz, $CDCl_3$)

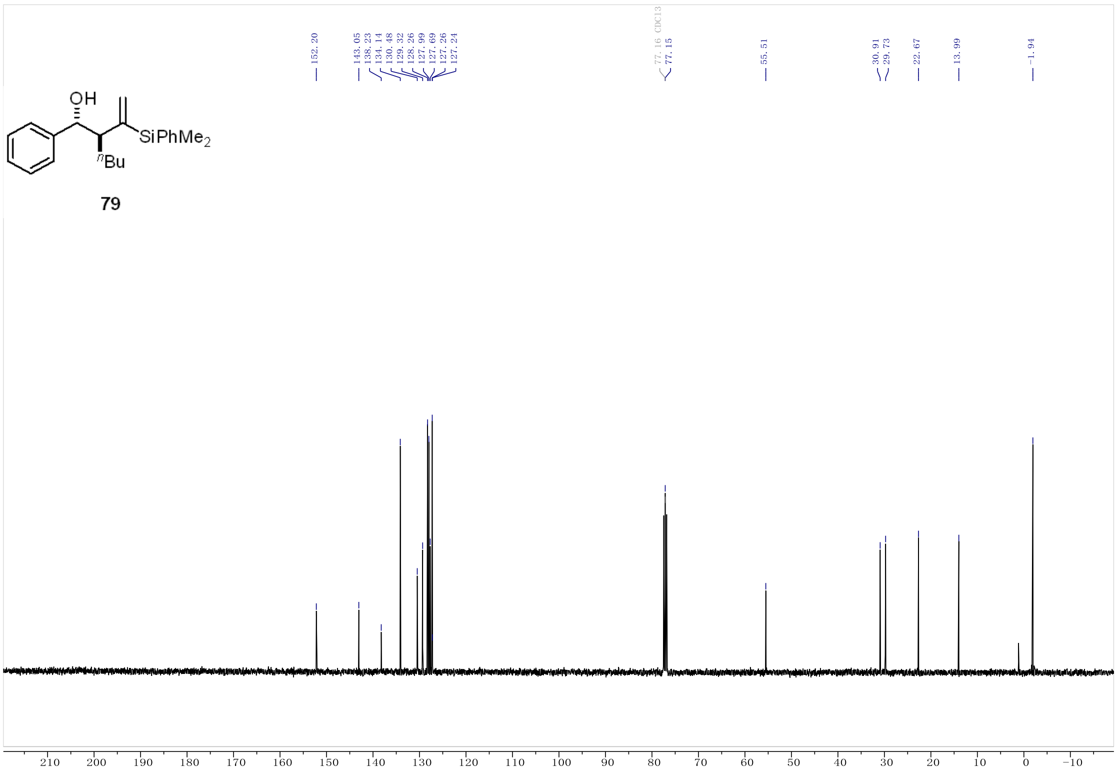

## $^{1}$H NMR of 80 (400 MHz, $CDCl_3$)

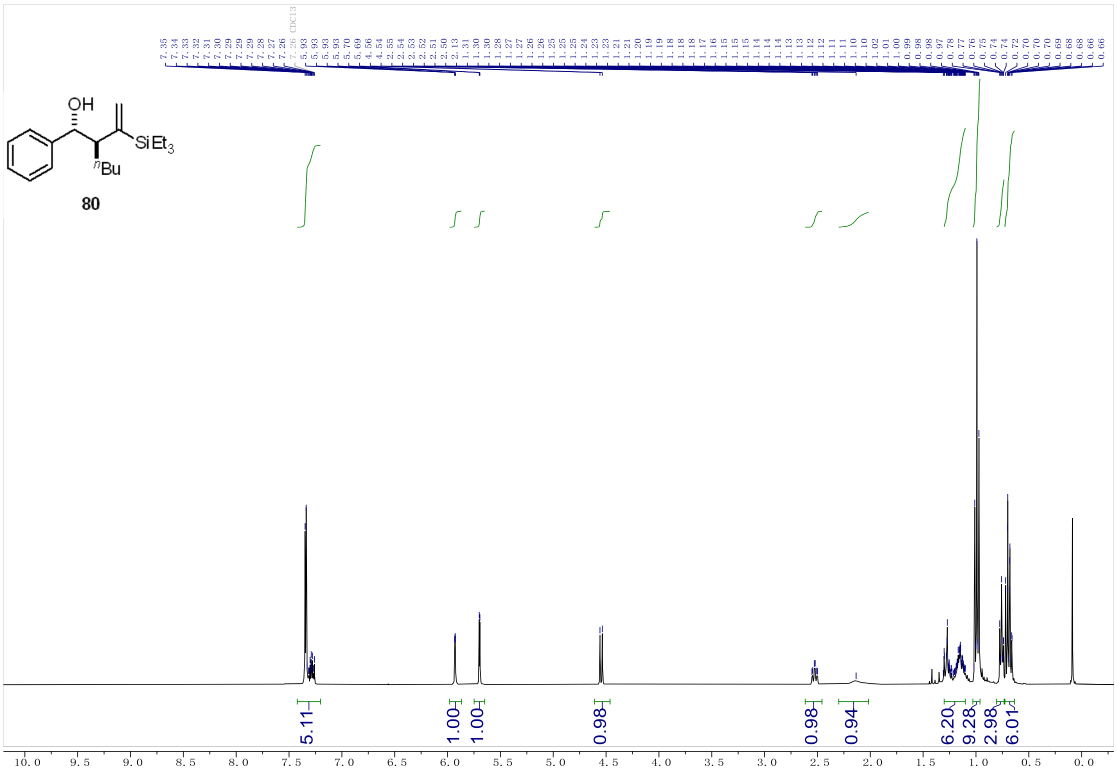

## $^{13}$C NMR of 80 (101 MHz, $CDCl_3$)

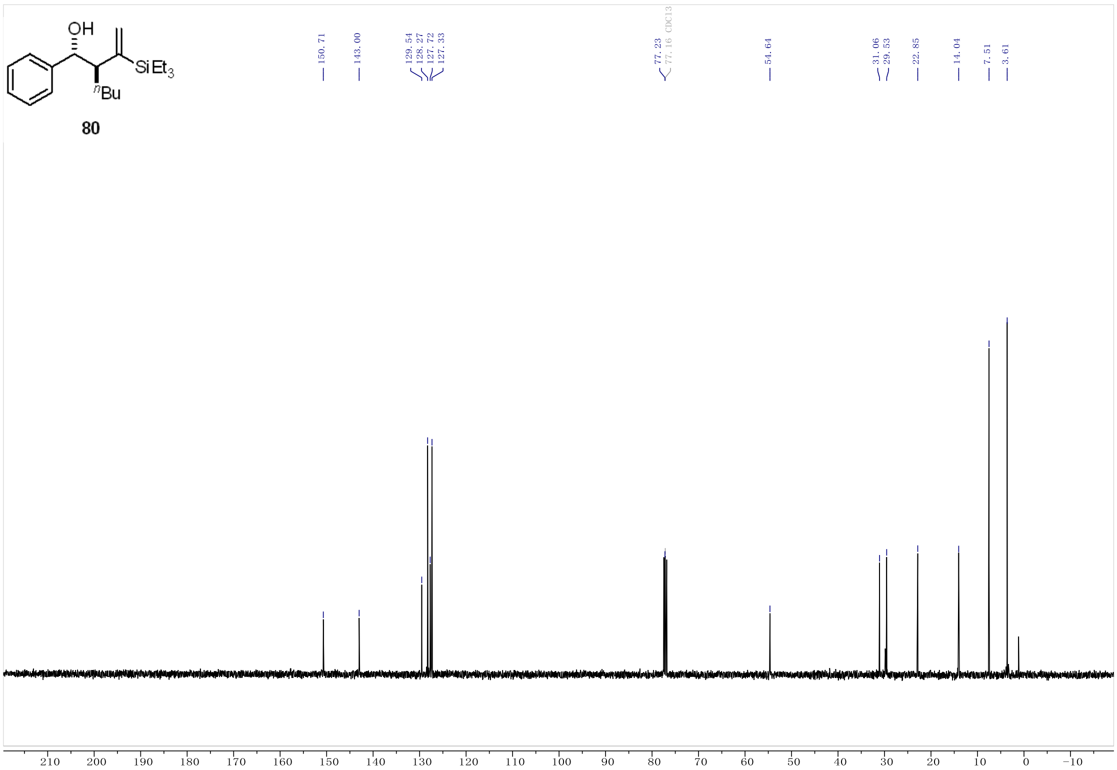

$^{1}H$ NMR of 81 (500 MHz, $CDCl_3$)

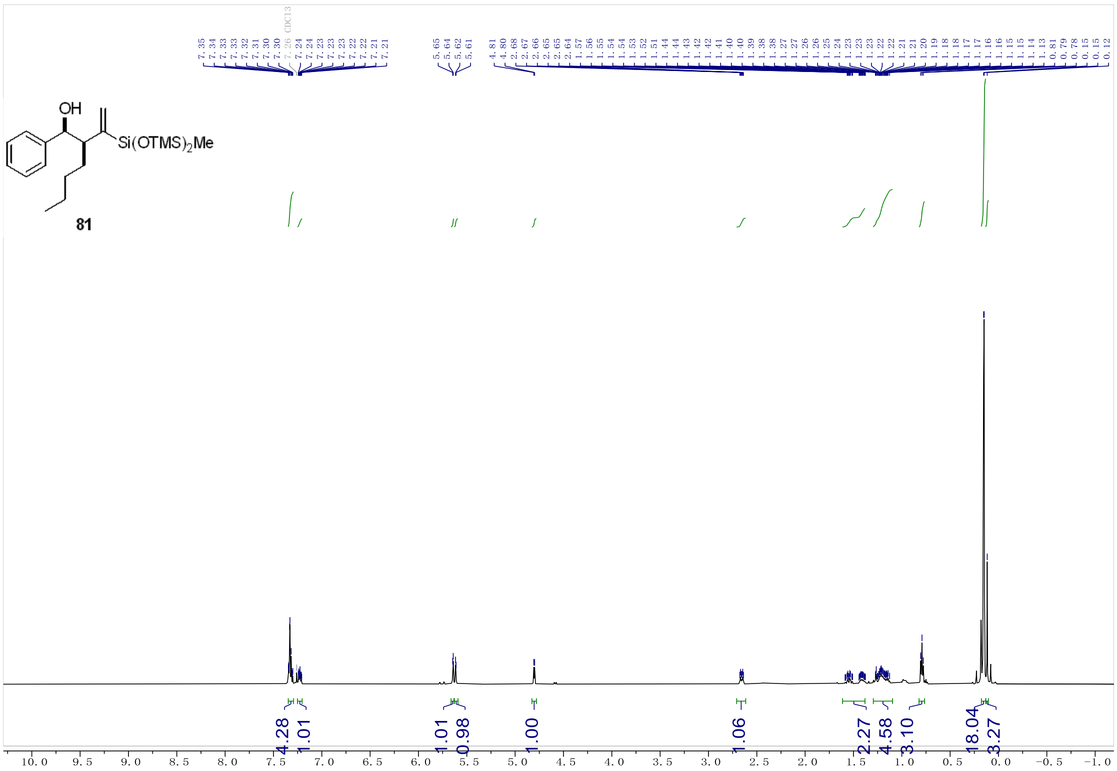

$^{13}C$ NMR of 81 (126 MHz, $CDCl_3$)

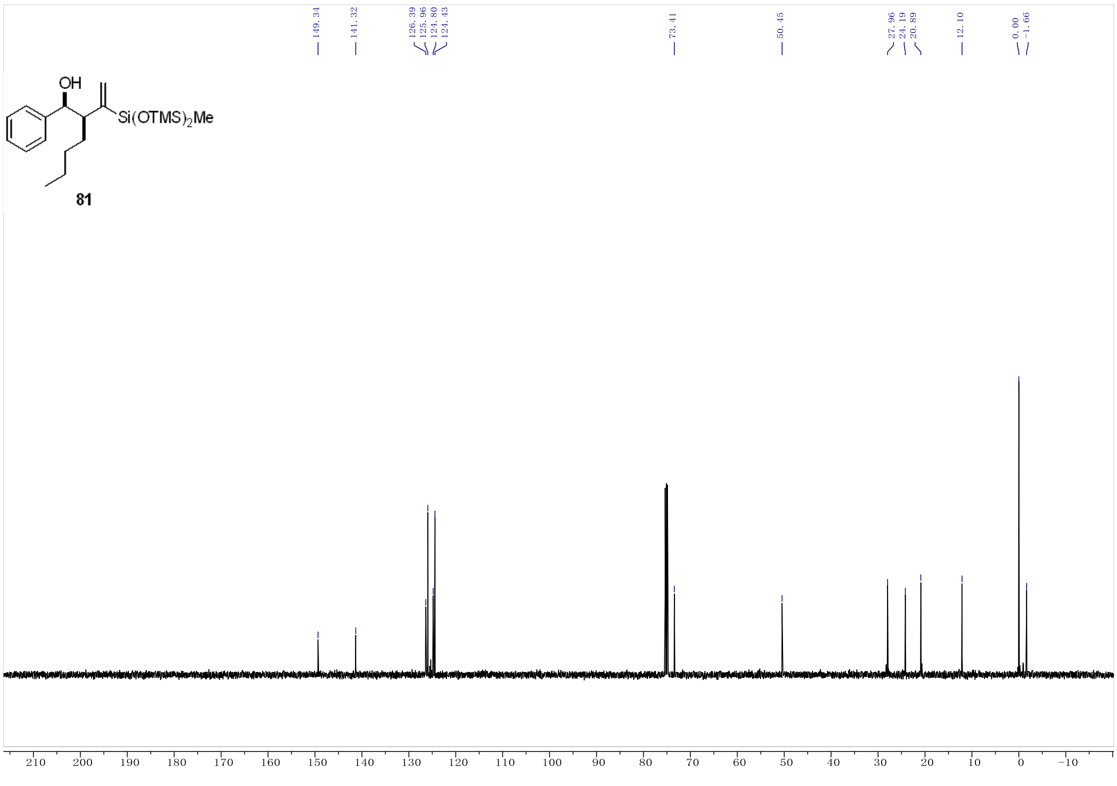

## $^1$H NMR of 78 (mixture of $\beta$ & $\gamma$ isomers) (500 MHz, $CDCl_3$)

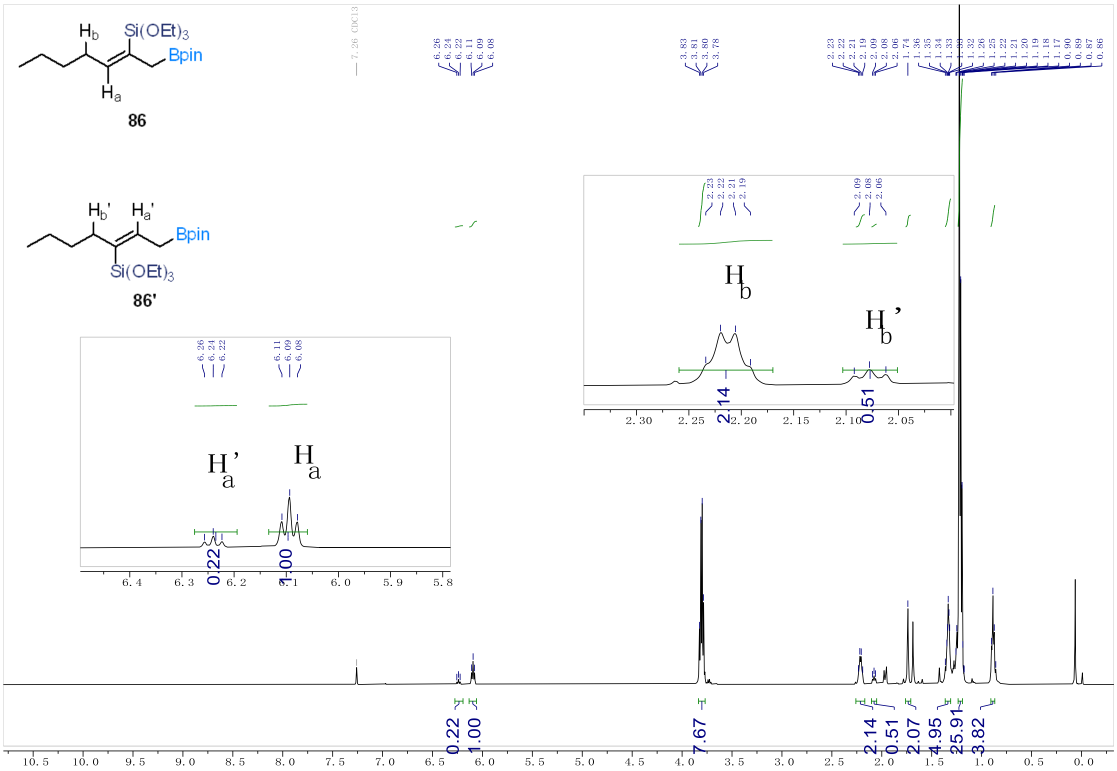

## $^1$H NMR of 83 (400 MHz, $CD_3CN$)

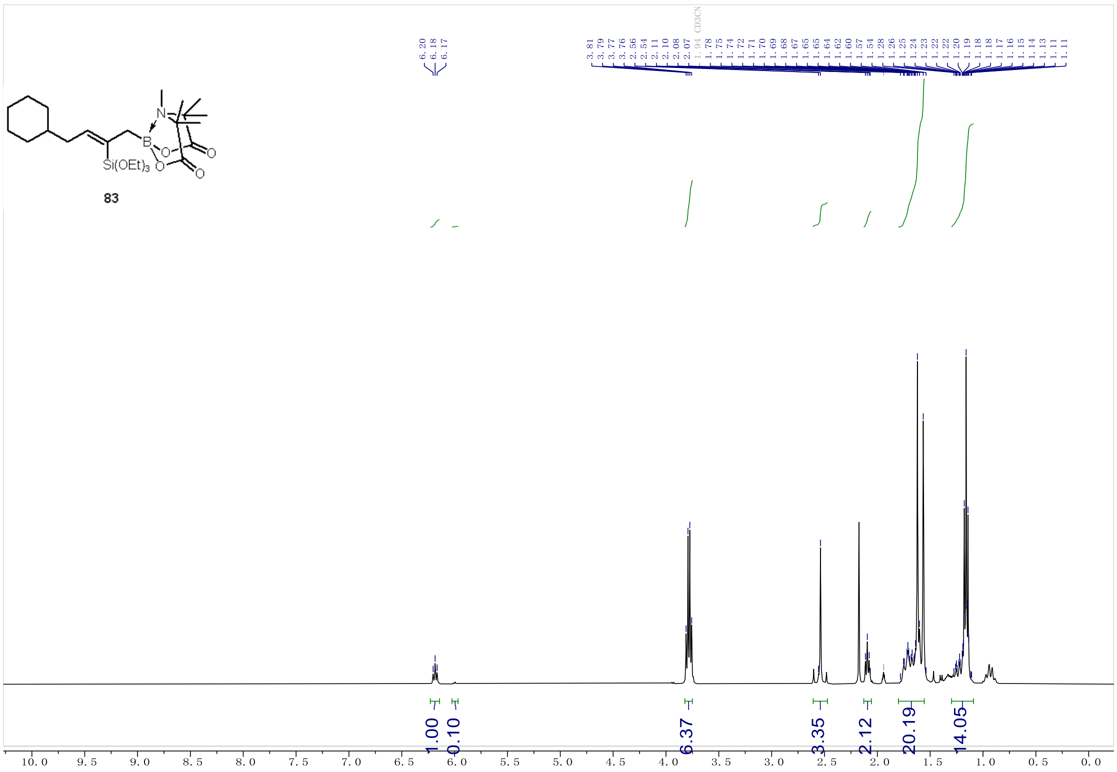

## $^{13}$C NMR of 83 (101 MHz, $CD_3CN$)

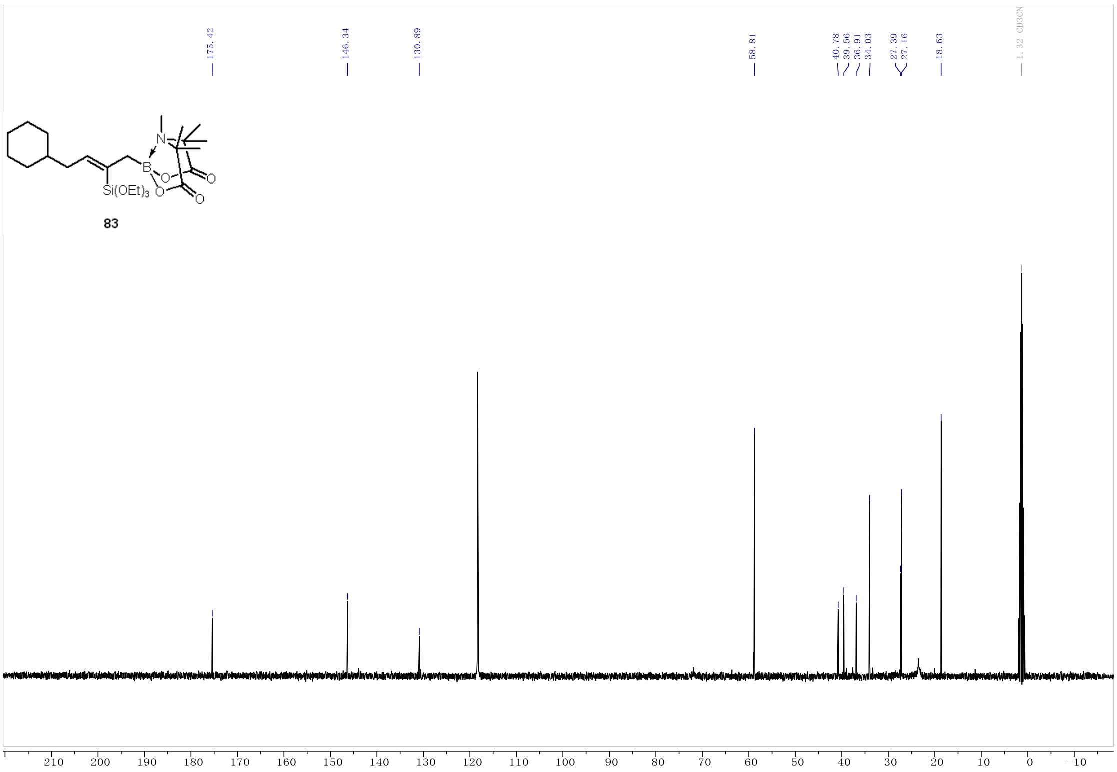


## $^{1}$H NMR of 86 (mixture of *γ/δ* isomers) (400 MHz, DMSO-$d_6$)

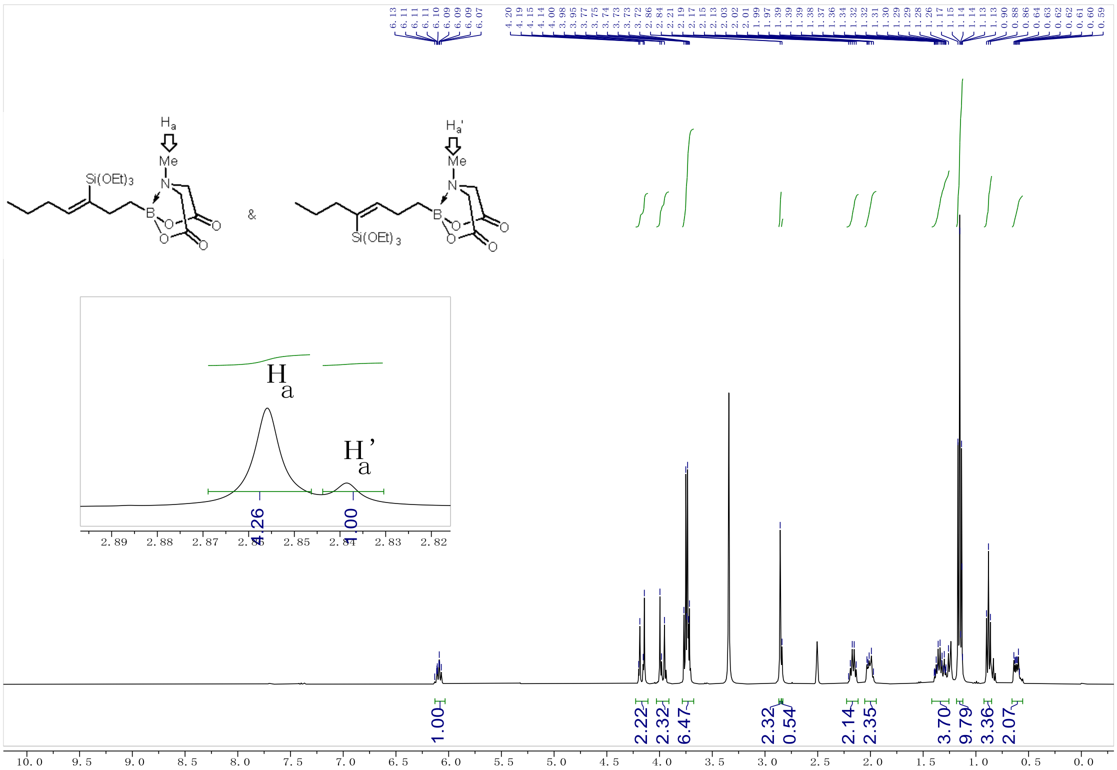

**$^{13}$C NMR of 86 (mixture of $\gamma/\delta$ isomers) (101 MHz, DMSO)**

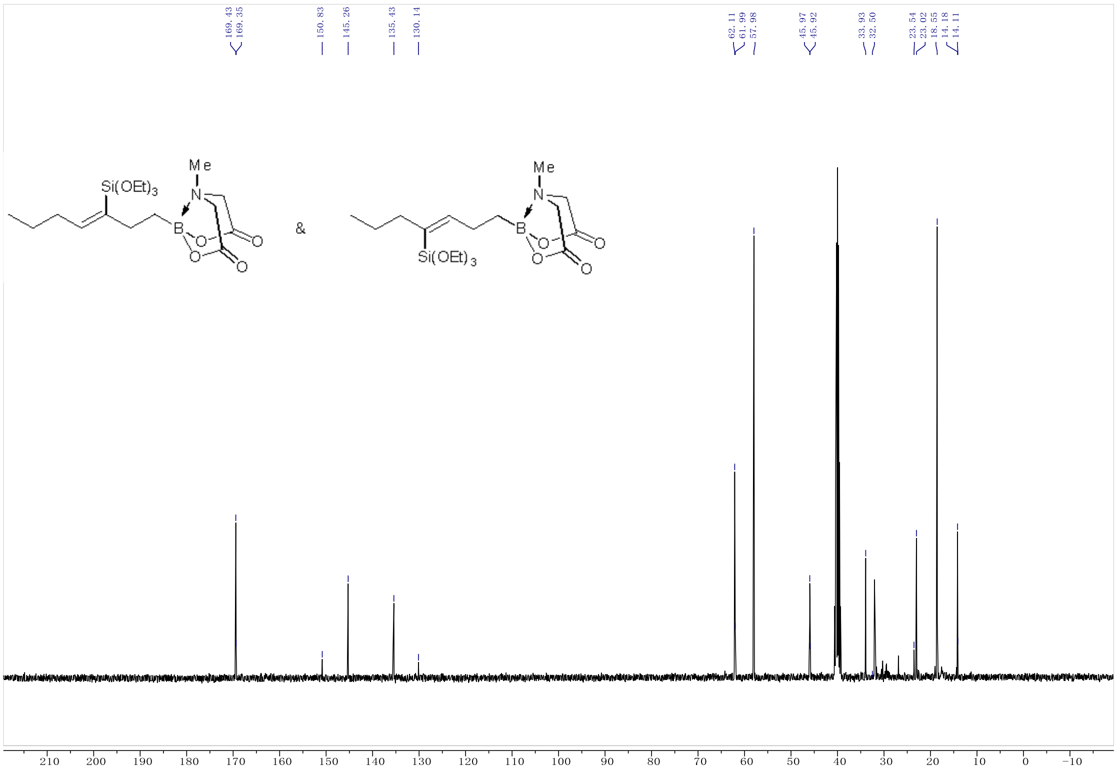

**$^{1}$H NMR of 3-*d* (400 MHz, Acetone-$d_6$)**

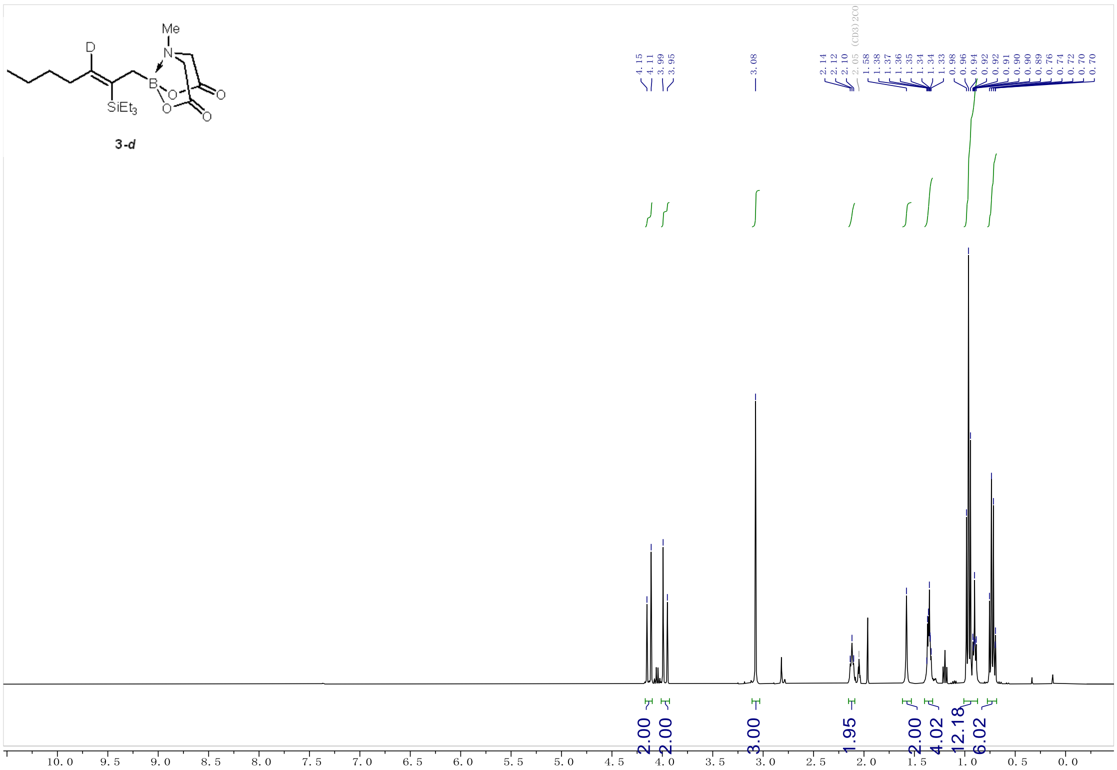

## $^{13}C$ NMR of 3-*d* (126 MHz, Acetone-$d_6$)

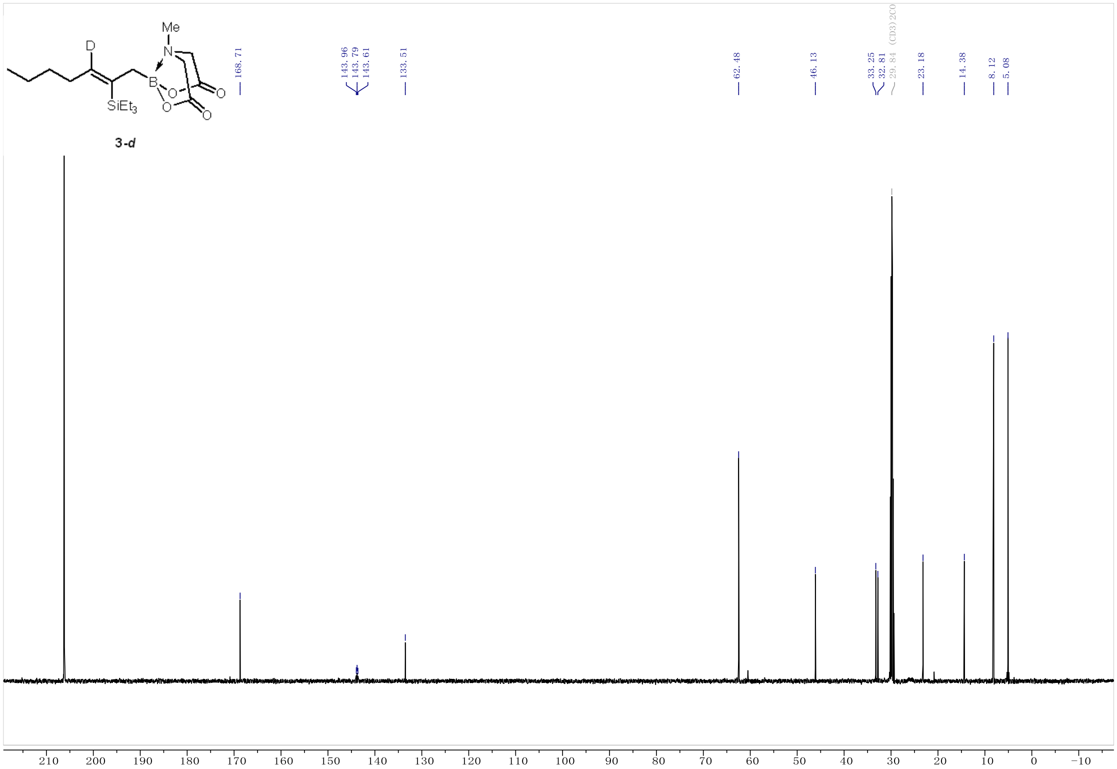


## 1H NMR of $DSiEt_3$ (500 MHz, $CDCl_3$)

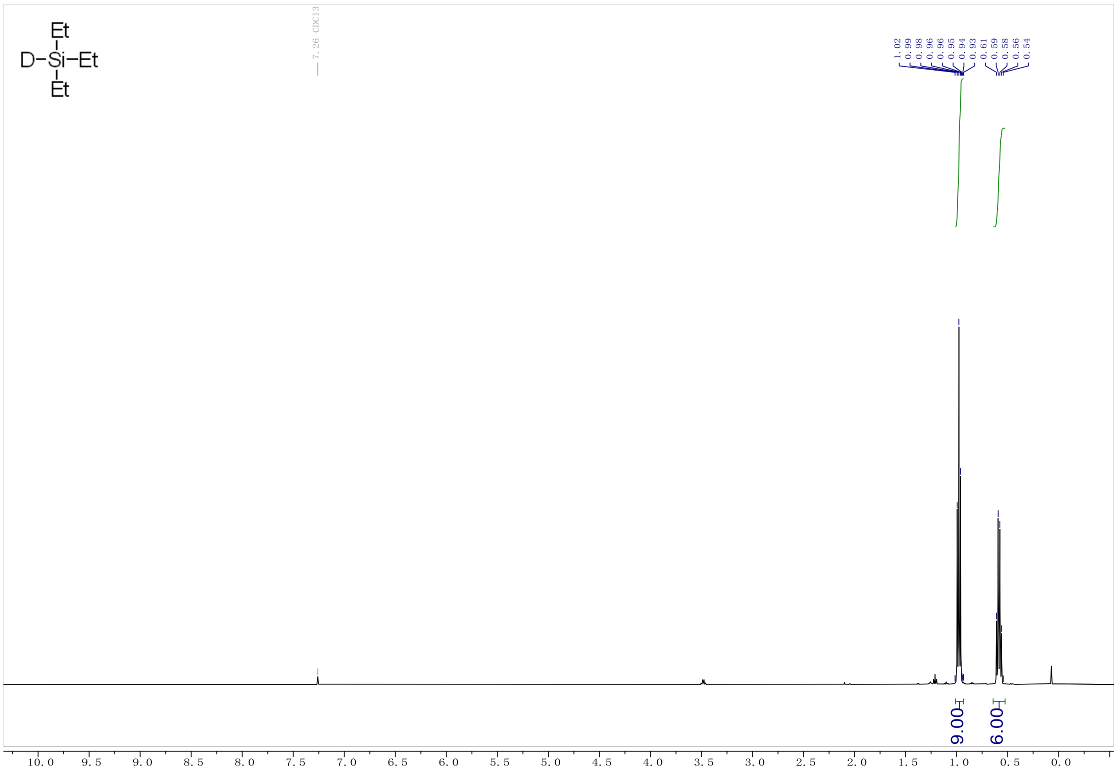

## $^{13}C$ NMR of $DSiEt_3$ (126 MHz, $CDCl_3$)

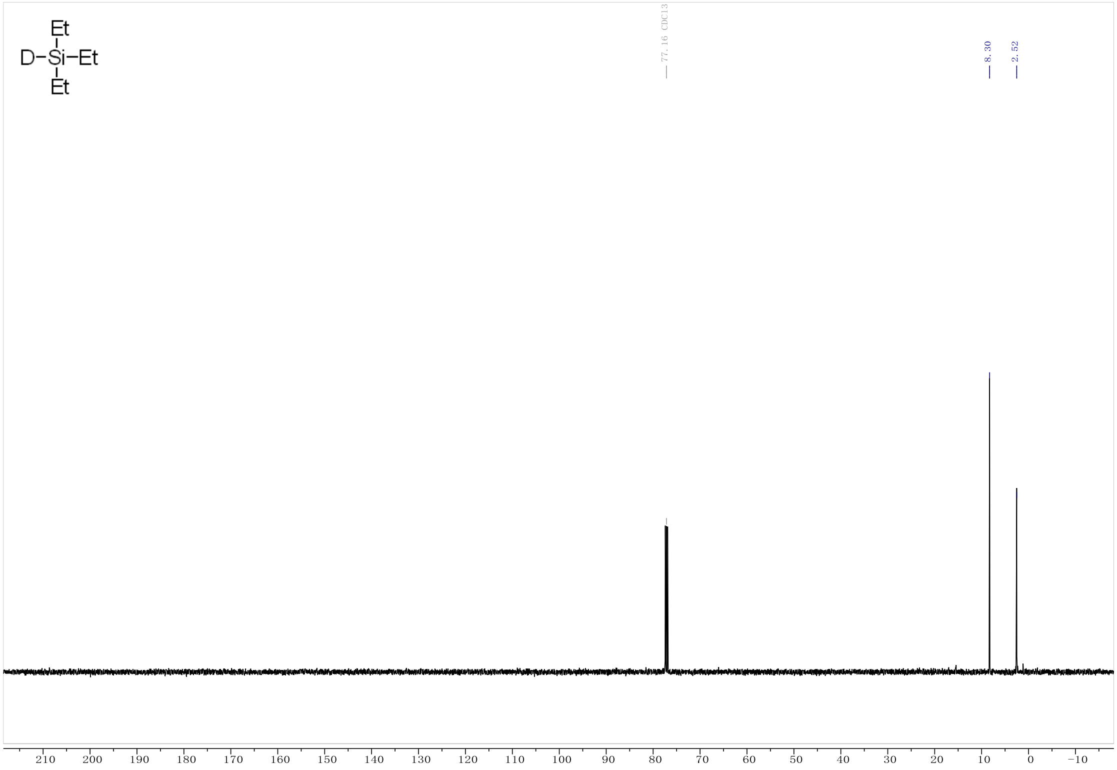


## $^{1}H$ NMR of 46-*d* (500 MHz, Acetone-$d_6$)

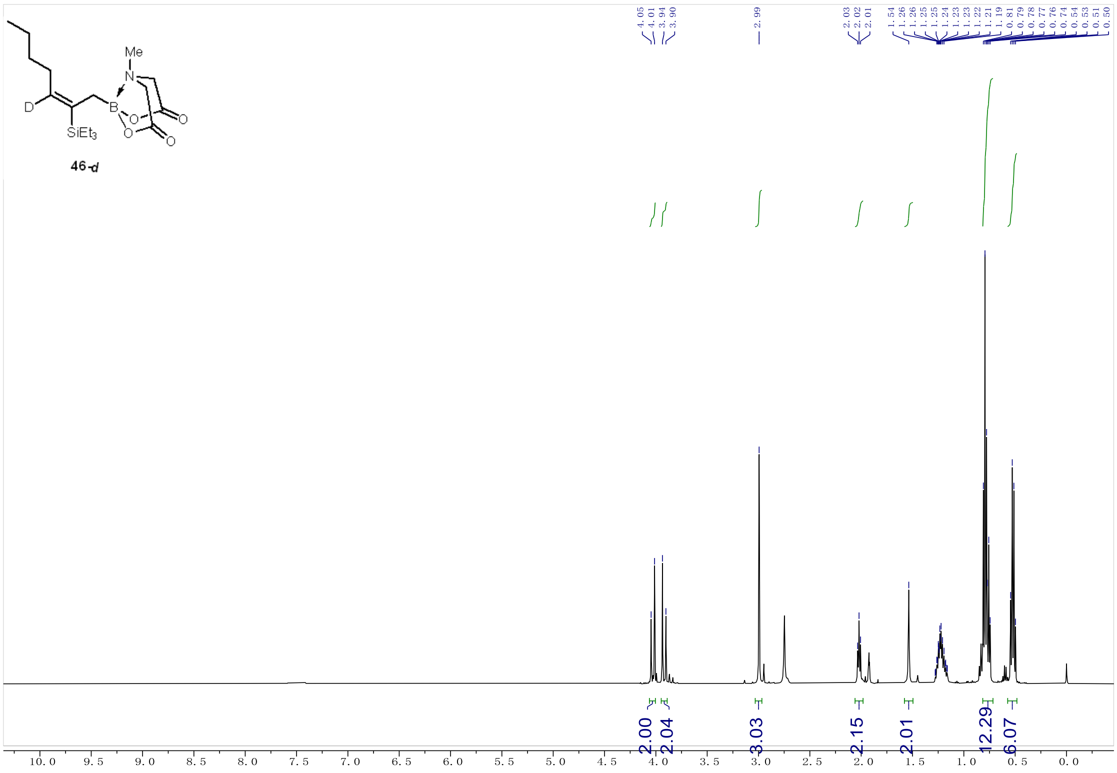


## $^{13}C$ NMR of 46-*d* (126 MHz, Acetone-$d_6$)

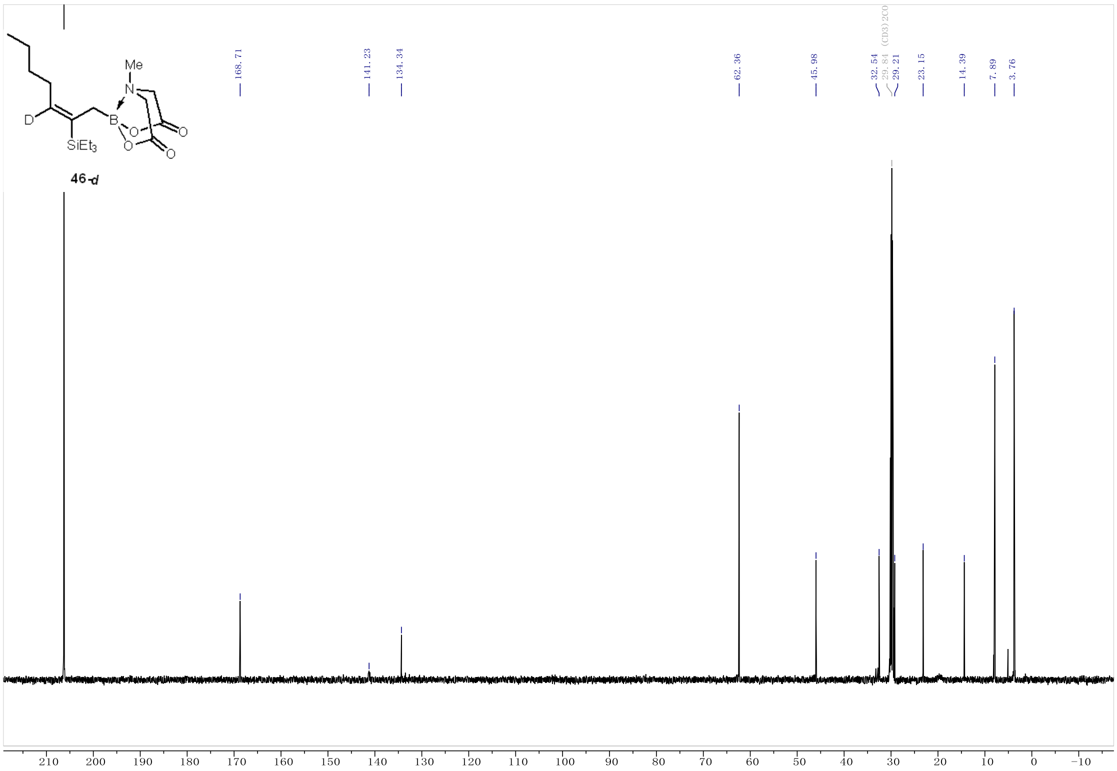

# 8. Reference


1. Li, Y.; Chen, Z.-H.; Lin, S.; Liu, Y.; Qian, J.; Li, Q.; Huang, Z.-S.; Wang, H., Regioselective electrophilic addition to propargylic B(MIDA)s enabled by β-boron effect. *Adv. Sci.* **2023,** *10* (30), 2304282.
2. Chen, Z.-H.; Su, X.-X.; Li, Q.; Wu, J.-Q.; Ou, T.-M.; Wang, H., Synthesis of α-Boryl Ketones via Hydration or Oxidation of B(MIDA)- Decorated Alkynes. *Org. Lett.* **2023,** *25* (7): 1099-1103. .
3. Liu, Y.; Chen, Z.-H.; Li, Y.; Qian, J.; Li, Q.; Wang, H., Boryl-Dictated Site-Selective Intermolecular Allylic and Propargylic C-H Amination. *J. Am. Chem. Soc.* **2022,** *144* (31), 14380-14387.
4. Wang, D.; Zhou, J.; Hu, Z.; Xu, T., Deoxygenative Haloboration and Enantioselective Chloroboration of Carbonyls. *J. Am. Chem. Soc.* **2022,** *144* (50), 22870-22876.
5. Frisch, M. J.; Trucks, G. W.; Schlegel, H. B.; Scuseria, G. E.; Robb, M. A.; Cheeseman, J. R.; Scalmani, G.; Barone, V.; Petersson, G. A.; Nakatsuji, H.; Li, X.; Caricato, M.; Marenich, A. V.; Bloino, J.; Janesko, B. G.; Gomperts, R.; Mennucci, B.; Hratchian, H. P.; Ortiz, J. V.; Izmaylov, A. F.; Sonnenberg, J. L.; Williams; Ding, F.; Lipparini, F.; Egidi, F.; Goings, J.; Peng, B.; Petrone, A.; Henderson, T.; Ranasinghe, D.; Zakrzewski, V. G.; Gao, J.; Rega, N.; Zheng, G.; Liang, W.; Hada, M.; Ehara, M.; Toyota, K.; Fukuda, R.; Hasegawa, J.; Ishida, M.; Nakajima, T.; Honda, Y.; Kitao, O.; Nakai, H.; Vreven, T.; Throssell, K.; Montgomery Jr., J. A.; Peralta, J. E.; Ogliaro, F.;

Bearpark, M. J.; Heyd, J. J.; Brothers, E. N.; Kudin, K. N.; Staroverov, V. N.; Keith, T. A.; Kobayashi, R.; Normand, J.; Raghavachari, K.; Rendell, A. P.; Burant, J. C.; Iyengar, S. S.; Tomasi, J.; Cossi, M.; Millam, J. M.; Klene, M.; Adamo, C.; Cammi, R.; Ochterski, J. W.; Martin, R. L.; Morokuma, K.; Farkas, O.; Foresman, J. B.; Fox, D. J. *Gaussian 09 Rev. D.01*, Wallingford, CT, 2013.

6. Stephens, P. J.; Devlin, F. J.; Chabalowski, C. F.; Frisch, M. J., Ab Initio Calculation of Vibrational Absorption and Circular Dichroism Spectra Using Density Functional Force Fields. *The Journal of Physical Chemistry* **1994,** *98* (45), 11623-11627.

7. Wadt, W. R.; Hay, P. J., Ab initio effective core potentials for molecular calculations. Potentials for main group elements Na to Bi. *The Journal of Chemical Physics* **1985,** *82* (1), 284-298.

8. Zhao, Y.; Truhlar, D. G., The M06 suite of density functionals for main group thermochemistry, thermochemical kinetics, noncovalent interactions, excited states, and transition elements: two new functionals and systematic testing of four M06-class functionals and 12 other functionals. *Theor. Chem. Acc.* **2008,** *120* (1), 215-241.

9. Andrae, D.; Häußermann, U.; Dolg, M.; Stoll, H.; Preuß, H., Energy-adjustedab initio pseudopotentials for the second and third row transition elements. *AcTC* **1990,** *77* (2), 123-141.

10. Scalmani, G.; Frisch, M. J., Continuous surface charge polarizable continuum models of solvation. I. General formalism. *The Journal of Chemical Physics* **2010,** *132* (11), 114110.

11. Lu, T.; Chen, F., Multiwfn: A multifunctional wavefunction analyzer. *Journal of Computational Chemistry* **2012,** *33* (5), 580-592.

12. Thompson, D. L., *Modern Methods for Multidimensional Dynamics Computations in Chemistry*. WORLD SCIENTIFIC: 1998; p 748.

13. Uggerud, E.; Helgaker, T., Dynamics of the reaction $CH_2OH$+ .fwdarw. CHO+ + H2. Translational energy release from ab initio trajectory calculations. *Journal of the American Chemical Society* **1992,** *114* (11), 4265-4268.

14. Helgaker, T.; Uggerud, E.; Jensen, H. J. A., Integration of the classical equations of motion on ab initio molecular potential energy surfaces using gradients and Hessians: application to translational energy release upon fragmentation. *Chemical Physics Letters* **1990,** *173* (2), 145-150.

15. CYLview20; Legault, C. Y., Université de Sherbrooke, 2020 (http://www.cylview.org).

16. Humphrey, W.; Dalke, A.; Schulten, K., VMD: Visual molecular dynamics. *Journal of Molecular Graphics* **1996,** *14* (1), 33-38.
