# Supplementary material for: Harnessing the β-boron effect for regioselective Ru-catalyzed hydrosilylation of internal alkynes
Source: Nat Commun. 2025 May 14;16:4469. doi: 10.1038/s41467-025-59823-x (PMC12078650; doi:10.1038/s41467-025-59823-x)
Supplement: Supplementary file 2 — Description of Additional Supplementary Files [file 41467_2025_59823_MOESM2_ESM.docx]

**File Name:** Supplementary Data 1

**Description:** Molecule Coordinates for Computational Studies.

**File Name:** Supplementary Data 2

**Description:** Cartesian Coordinates for Computational Studies.
